# Natural variation of HIV-1 group M integrase: Implications for a new class of antiretroviral inhibitors

**DOI:** 10.1186/1742-4690-5-74

**Published:** 2008-08-07

**Authors:** Soo-Yon Rhee, Tommy F Liu, Mark Kiuchi, Rafael Zioni, Robert J Gifford, Susan P Holmes, Robert W Shafer

**Affiliations:** 1 Division of Infectious Diseases, Department of Medicine, Stanford University, Stanford, CA, USA; 2 Department of Statistics, Stanford University, Stanford, CA, USA

## Abstract

HIV-1 integrase is the third enzymatic target of antiretroviral (ARV) therapy. However, few data have been published on the distribution of naturally occurring amino acid variation in this enzyme. We therefore characterized the distribution of integrase variants among more than 1,800 published group M HIV-1 isolates from more than 1,500 integrase inhibitor (INI)-naïve individuals. Polymorphism rates equal or above 0.5% were found for 34% of the central core domain positions, 42% of the C-terminal domain positions, and 50% of the N-terminal domain positions. Among 727 ARV-naïve individuals in whom the complete *pol *gene was sequenced, integrase displayed significantly decreased inter- and intra-subtype diversity and a lower Shannon's entropy than protease or RT. All primary INI-resistance mutations with the exception of E157Q – which was present in 1.1% of sequences – were nonpolymorphic. Several accessory INI-resistance mutations including L74M, T97A, V151I, G163R, and S230N were also polymorphic with polymorphism rates ranging between 0.5% to 2.0%.

## Introduction

HIV-1 integrase contains 288 amino acids encoded by the 3' end of the HIV-1 *pol *gene. It catalyzes the cleavage of the conserved 3' dinucleotide CA (3' processing) and the ligation of the viral 3'-OH ends to the 5'-DNA of host chromosomal DNA (strand transfer). Integrase also plays a role in stabilizing a pre-integration complex (PIC), which consists of the 3'-processed genome and one or more cellular co-factors involved in nuclear transfer of the PIC (reviewed in [1-4]).

HIV-1 integrase is composed of three functional domains: the N-terminal domain (NTD), which encompasses amino acids 1–50 and contains a histidine-histidine-cysteine-cysteine (HHCC) motif that coordinates zinc binding, the catalytic core domain (CCD) which encompasses amino acids 51–212 and contains the catalytic triad D64, D116, and E152, known as the DDE motif, and the C-terminal domain (CTD), which encompasses amino acids 213–288 and is involved in host DNA binding.

Crystal structures of the CCD plus CTD domains [5] and the CCD plus NTD domains [6] have been solved, but the relative conformation of the three domains and of the active multimeric form of the enzyme are not known. There is one published crystal structure of the CCD bound to an early prototype diketo acid inhibitor (5CITEP) [7] but no structures of the CCD bound to one of the integrase inhibitors (INIs) in clinical use or to a DNA template. Because of the difficulties in obtaining structures of the most biologically relevant forms of the enzyme and of most integrase-INI structures, much of the functional roles of different integrase residues have been identified through biochemical and systematic amino acid replacement studies (reviewed in [8]).

One INI, raltegravir, has been licensed for the treatment of HIV-1 infection and a second INI, elvitegravir, is in advanced clinical trials. Mutations associated with resistance to these inhibitors have been identified through *in vitro *and *in vivo *selection studies (reviewed in [9]) and through *in vitro *susceptibility testing. The purpose of this study is to supplement the structural and biochemical assessment of integrase function and INI resistance by summarizing naturally occurring variation in published sequences of group M integrase, particularly as this variation applies to positions associated with INI resistance.

## Methods

### Sequence retrieval and annotation

The HIV-1 subtype B consensus integrase amino acid published by the Los Alamos HIV Sequence Database was used to query the GenBank database V 165.0 (released on 2008-04-15) using the blastp program. Human and primate lentivirus virus sequences having an e-value of < 0.04 and containing 200 or more homologous amino acids were aligned to the query sequence using a nucleotide to amino acid alignment program [10]. Each sequence was annotated according to its primary publication, the host species from which it was obtained, the year, country, and biological source of its isolation, and the ARV drug class exposure of the individual from whom the sample was obtained. Each set of sequences from a publication was annotated according to whether the sequences were obtained from one or more than one individual in that publication. Sequences from the same individual were annotated according to whether they were obtained at the same or different times. Sequences were also characterized according to whether obtained directly from PCR-amplified material or from one or more separate clones. For the purposes of analysis, only one sequence per individual were used. For individual with multiple sequences, the first sequence was used. For integrase isolates for which multiple clones were sequenced, the consensus of the clones was used.

Insertions, deletions, and mutations were defined as differences from the HIV-1 subtype B consensus amino acid sequence. The retrovirus species and the HIV-1 group of each sequence was defined according to the sequence annotation in GenBank and confirmed through phylogenetic analysis. HIV-1 group M subtype was assigned phylogenetically by including each group M sequence in a neighbor-joining tree containing 100 sequences that had previously been characterized by full genomic sequencing including sequences belonging to subtypes A, B, C, D, F, G, H, J, and K and to the circulating recombinant forms (CRFs) 01 to 19. This set of 100 sequences included the 65 subtype-specific reference sequences assembled by the Los Alamos HIV Sequence Database [11] supplemented by 35 sequences so that a minimum of three published sequences belonging to each subtype and CRF was included. The neighbor joining tree was created from a distance matrix computed using the HKY method with a gamma distribution calculated by PAUP 4.0. Sequences that formed a clade with reference sequences belonging to the same subtype were assigned to that subtype. Sequences that did not form a clade with references belonging to the same subtype but that were within a genetic distance of 0.12 from a reference sequence were assigned to the subtype of the closest sequence.

### Sequence quality control

Four categories of sequences were excluded from analysis including (i) sequences of uncertain provenance that lacked sufficient annotation as to the sequence's origin, (ii) sequences submitted to GenBank more than once or derived from a previously submitted sequence through experimental manipulation either *in vitro *or in a primate model ("experimental sequences"), (iii) case reports of complete genomic sequences that were submitted to GenBank because of some unusual characteristic unrelated to integrase or to sequence diversity (e.g. a strain with unique tropism characteristics, or a strain associated with an epidemiologic cluster), and (iv) sequences of poor quality defined as having two or more of the following features: stop codons, frame shifts, highly ambiguous nucleotides (B, D, H, V, N), active site mutations, or unique insertions or deletions.

### Analysis of sequence heterogeneity

For most analyses, polymorphisms were defined as mutations present in ≥ 0.5% of group M sequences. However, all mutations at essential integrase positions or at known INI-resistance positions that were present in sequences from one or more individuals are also noted in the text.

To compare HIV-1 integrase heterogeneity with that of protease and RT, we assembled virus sequences from ARV-naïve individuals for which the complete *pol *gene had been sequenced. For this set of sequences, we calculated the uncorrected pair-wise amino acid differences between sequences belonging to the species HIV-1 and HIV-2, sequences belonging to the different HIV-1 groups (M, N, and O) and HIV-1_cpz _isolates, sequences belonging to the different group M HIV-1 subtypes, and within the six most common group M subtypes. For the six most common group M subtypes, we also examined the number of differences from the consensus subtype sequence and examined the distribution of these differences across each of the sequences and each of these genes.

We used an information-theoretic measure of diversity known as Shannon's entropy [12] to quantify the amount of amino acid variation at each position of protease, RT, and integrase for the set of ARV-naïve sequences for which the complete pol gene was sequenced. For each subtype, the entropy at each position of protease, RT, and integrase was calculated as:

$$\text{Entropy }(\text{X})=-\sum_{i=1}^{k}p(Ai)\cdot \log p(Ai),$$

for K different amino acids (*A*_1 _... *A*_*k*_) at position X where *p*(*A*_*i*_) is the frequency of amino acid *A*_*i*_.

To assess covariation among integrase amino acids, we analyzed sequences belonging to the six most common group M subtypes using the Jaccard similarity coefficient (J). For a given pair of mutations X and Y, the Jaccard similarity coefficient is calculated as *J *= *N*_*XY*_/(*N*_*XY *_+ *N*_*X*0 _+ *N*_0*Y*_) where N_XY _represents the number of sequences containing X and Y, N_X0 _represents the number of sequences containing X but not Y and N_0Y _represents the number of sequences containing Y but not X. To test whether observed Jaccard similarity coefficients were statistically significant, the expected value of the Jaccard similarity coefficients (J_RAND_) and its standard error (J_SE_) assuming two mutations (X and Y) occur independently were calculated for each pair of mutations. J_RAND _was calculated as the mean Jaccard similarity coefficient after 2,000 random rearrangements of the X or Y vector (containing 0 or 1 for presence or absence of a mutation). J_SE _was calculated using a jack-knifed procedure, which removed one sequence at a time, repeatedly for each sequence. The standardized score Z, *Z *= (*J *- *J*_*RAND*_)/*J*_*SE*_, indicates a significant positive association (Z > 2.56) or a significant negative association (Z < -2.56) at an unadjusted p < 0.01 [13]. To adjust for multiple comparisons, we used a false discovery rate of 0.05 to identify correlations warranting further examination [14].

## Results

### Published integrase sequences

The April 15, 2008 GenBank release contained 2,736 primate lentivirus integrase sequences with 200 or more amino acids. Twenty-nine percent of these sequences (n = 775) were excluded from analysis because they were of poor sequence quality (n = 385), contained insufficient annotation (n = 291), represented experimental sequences (n = 96), or represented case reports of viruses sequenced for phenotypic properties unrelated to integrase (n = 93). Of the remaining 1,961 sequences, 1,863 sequences belonged to HIV-1/SIVcpz, 40 sequences belonged to HIV-2/SIVsmm/SIVmac, and 58 sequences belonged to one of the remaining primate lentivvirus species.

The 1,863 HIV-1/SIVcpz sequences were obtained from 1,626 separate virus isolations from 1,581 individuals including 1,563 persons with HIV-1 and 18 chimpanzees with SIVcpz. Table 1 summarizes the taxonomic categories of the HIV-1 sequences according to the number of distinct individuals from whom sequences were obtained. Among 1,482 persons with group M viruses, sequences from 1,351 were classified as belonging to subtypes A, B, C, D, F, G, CRF01, or CRF02; whereas sequences from 131 were classified as belonging to subtypes H, J, K or one of the other CRFs (n = 87); 44 sequences could not be adequately subtyped (n = 44). Among 1,051 group M integrase sequences in the database for which the complete genome sequence had been published, the assigned subtype matched the subtype indicated in the primary publication for the integrase region in 1,045 (99.4%) sequences. Of the 1,563 persons from whom HIV-1 sequences were obtained, none had received an INI. Seven persons had received an RT and/or protease inhibitor and in 525 persons RT and protease inhibitor treatment history was not known. A file containing the nucleotides and GenBank accession numbers of the sequences in Table 2 is provided [see Additional file 1].

**Table 1:** Numbers of individuals with primate lentivirus integrase sequences > 200 amino acids by species, HIV-1 group, and subtype

**Species**	**Group**	**Subtype**	**No. individuals**
HIV-1	M	A	157
		B	367
		C	431
		CRF01_AE	130
		CRF02_AG	93
		D	82
		F	56
		G	35
		Others^†^	131
	N		5
	O		76
	CPZ		18
HIV-2			39
NHPL*			58

*NHPL: non-human primate lentiviruses exclusive of SIV_CPZ _and SIV_STM_/SIV_MAC_. SIV_CPZ _and SIV_STM_/SIV_MAC _are listed with human HIV-1 and HIV-2 isolates, respectively

^†^Others: Subtypes, H, J, K, circulating recombinant forms other than 01 and 02, as well as non-CRF recombinants, and other non-classifiable group M sequences.

**Table 2:** Integrase positions at which different subtypes have different consensus residues

**Subtype**	**No.**	**14**	**17**	**21**	**25**	**31**	**39**	**50**	**72**	**84**	**100**	**101**	**112**	**113**	**119**	**124**	**125**	**134**
		
		**K^94^**	**S^76^**	**A^95^**	**D^96^**	**V^77^**	**S^91^**	**M^83^**	**I^51^**	**I^98^**	**F^100^**	**L^56^**	**T^87^**	**I^90^**	**S^69^**	**T^54^**	**T^69^**	**G^98^**
**A**	160	R^64^				I^77^			V^89^				V^92^	V^75^		A^78^	A^96^	N^57^
**AE**	132	R^96^		T^86^		I^80^	N^78^		V^93^				V^98^			A^95^	A^98^	N^92^
**AG**	93	R^89^				I^72^						I^83^	V^91^			A^96^	A^96^	N^81^
**C**	432				E^81^	I^76^		I^58^			Y^72^	I^95^	V^92^			A^70^	A^92^	
**D**	82		N^70^					L^59^	V^82^				V^84^	V^89^		A^82^	A^78^	
**F**	57		N^81^						V^70^	L^81^		I^86^			T^46^	A^70^	A^70^	
															P^40^			
**G**	35	R^89^				I^60^						I^86^	V^75^			A^71^	A^86^	N^71^

**Subtype**	**No.**	**135**	**136**	**167**	**201**	**205**	**206**	**211**	**218**	**227**	**234**	**255**	**256**	**265**	**269**	**278**	**283**	
		
		**I^93^**	**K^97^**	**D^98^**	**V^62^**	**A^99^**	**T^86^**	**K^89^**	**T^92^**	**Y^95^**	**L^87^**	**S^97^**	**D^79^**	**A^78^**	**R^99^**	**D^98^**	**S^83^**	

**A**	160		Q^86^	E^81^	I^98^						I^70^						G^73^	
**AE**	132	V^92^	R^69^	E^93^	I^98^						I^96^						G^96^	
**AG**	93	V^78^	T^82^		I^99^		S^92^				I^98^						G^84^	
**C**	432		Q^89^		I^98^				I^64^		I^98^			V^55^	K^57^	A^94^	G^87^	
**D**	82				I^98^						I^94^		E^57^					
**F**	57		Q^89^		I^98^	S^65^		R^56^	I^72^		V^91^		E^96^				G^91^	
**G**	35		T^97^		I^97^		S^94^			F^57^	I^91^	N^94^	E^100^				G^94^	

Abbreviations: No. – number of sequences. The header shows the amino acid consensus for subtype B isolates. The individual rows indicate the amino acid positions at which specific subtypes have a consensus amino acid different from subtype B. The superscript indicates the proportion of isolates of that row's subtype which have the consensus amino acid for that subtype. Empty cells indicate that the subtype has the same consensus amino acid as the consensus for subtype B.

### HIV-1 group M amino acid polymorphisms

**Figure 1:**
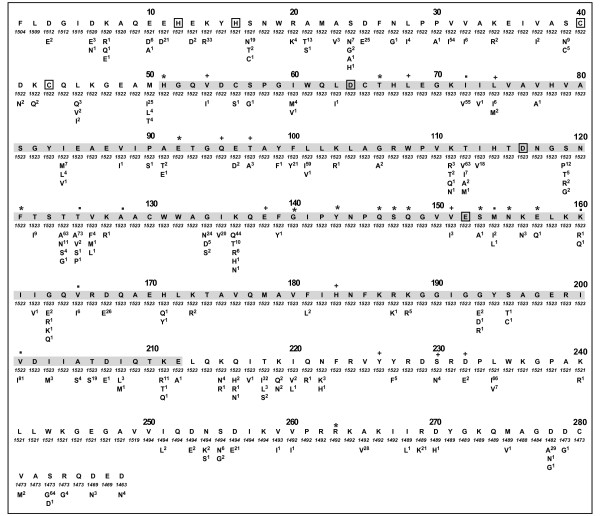
**Distribution of variants among group M HIV-1 integrase sequences.** The consensus subtype B sequence is shown at the top of each 40 amino acid section. Beneath the consensus B sequence is the number of annotated sequences containing an unambiguous amino acid at the indicated position with the number of such sequence ranging from 1183 to 1288. All variants reported at a level of ≥ 0.5% of sequences are indicated. The central core domain residues are surrounded by grey shading. The signature HHCC zinc-binding motif in the N-terminal domain and the DDE active site residues in the central core domain are indicated by boxes. Positions at which primary INI-resistance mutations for raltegravir and elvitegravir have been reported are indicated by "*". Positions at which accessory INI-resistance mutations for raltegravir and elvitegravir have been reported are indicated by "+". Positions at which INI-resistance mutations for other inhibitors have been reported are indicated by ".".

Figure 1 shows the distribution of amino acid variation among all group M integrase sequences compared with the consensus B reference sequence. Of the 288 integrase positions, 115 (39.9%) had at least one amino acid polymorphism present in 0.5% or more sequences including 41 (14.2%) at which two or more polymorphisms were present. Of the 185 polymorphisms, many resulted from highly conservative substitutions such as V↔I↔L in 32 cases, K↔R in 15 cases, A↔S↔T in 17 cases, and D↔E in 12 cases.

Table 2 summarizes the differences in the consensus amino acid sequence for each of the eight most common subtypes. For 33 (11.5%) of the 288 integrase positions, two or more subtypes had different consensus amino acids. Most of the polymorphic positions shown in Figure 1 are polymorphic in three or more subtypes [see Additional file 2]. However, at a few positions, the high level of amino acid variability shown in Figure 1 results largely from inter-subtype rather than intra-subtype variability. For example, much of the variability at the highly variable positions 112, 124, 125, 201, 234, and 283 results in part because the consensus B amino acid differs from the consensus of most other subtypes.

Likewise, variability in just one or two subtypes can explain some of the findings in Figure 1. For example, the uncommon polymorphism F139Y is due solely to the presence of this mutation in 8% of subtype A sequences. The uncommon polymorphism V151I which appears to be an accessory INI-resistance mutation is due solely to the presence of this mutation in 10% of subtype B sequences. Finally, the uncommon polymorphism K156N, another accessory INI-resistance mutation is due solely to the presence of this mutation in 9% of subtype B and 5% of subtype D sequences.

### HIV integrase, RT, and protease diversity

Among the 1,961 integrase sequences in Table 1, 1,367 were from isolates for which simultaneous protease and RT sequences were also available including 1,301 HIV-1/SIVcpz, 33 HIV-2/SIVstm and 33 NHPL isolates. For this comparative analysis, isolates from ARV-naive individuals of which the subtypes of the three genes are the same were used. When there are multiple isolates available from a same patient, only one isolate is used. Table 3 displays the extent of protease, RT, and integrase amino acid diversity by species, group, and subtype for these isolates. Integrase amino acid diversity decreased from ~40% at the species level, ~16% at the group level, to ~7% at the subtype level. The mean intra-subtype diversity was ~5%. At all levels, the extent of amino acid diversity was lower in integrase than in protease and RT, although there was no mean difference in amino acid diversity between integrase and RT between HIV-1 and HIV-2.

**Table 3:** Amino acid inter-species, inter-group, inter-subtype, and intra-subtype divergence among protease, RT, and integrase sequences

**Divergence**	**Protease**	**RT**	**Integrase**
Inter-species			
HIV-1 (789) vs HIV-2 (26)	0.51 ± 0.03	0.40 ± 0.04	0.40 ± 0.02
			
Inter-group			
group M (764) vs O (21)	0.29 ± 0.02	0.22 ± 0.01	0.18 ± 0.01
group M (764) vs N (4)	0.21 ± 0.02	0.14 ± 0.01	0.11 ± 0.01
group O (21) vs N (4)	0.30 ± 0.02	0.21 ± 0.01	0.18 ± 0.01
			
Inter-subtype			
Subtype A (71) vs B (145)	0.11 ± 0.03	0.10 ± 0.01	0.07 ± 0.01
Subtype A (71) vs C (337)	0.10 ± 0.03	0.09 ± 0.01	0.07 ± 0.01
Subtype B (145) vs C (337)	0.11 ± 0.02	0.09 ± 0.01	0.07 ± 0.01
			
Intra-subtype			
Subtype A (71)	0.05 ± 0.02	0.07 ± 0.02	0.05 ± 0.01
Subtype B (145)	0.07 ± 0.03	0.06 ± 0.01	0.05 ± 0.02
Subtype C (337)	0.06 ± 0.03	0.06 ± 0.01	0.04 ± 0.01

Divergence was defined as the mean proportion of amino acid difference between all sequence pairs. The number of sequences compared are within parentheses.

Among the 741 ARV-naïve HIV-1 group M isolates belonging to the six subtypes with the most sequences (A, B, C, D, CRF01, and CRF02), the number of differences from the subtype consensus sequence was highly correlated between all three pairs of genes (correlation coefficient ~0.34, p < 0.001). In other words, virus isolates with many differences from the subtype consensus in one gene tended to have many difference from the subtype consensus in all three genes. Nonetheless, a regression model that accounted for this factor (by using the covariance in the number of mutations among protease, RT, and integrase and the variance within each gene) and that accounted for the length of each gene confirmed that there were fewer differences from the subtype consensus in integrase compared with RT and protease.

**Figure 2:**
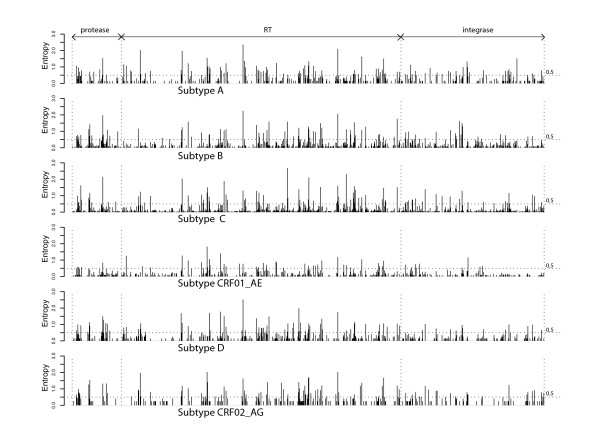
**Level of Shannon's entropy across the 99 amino acids of protease, 560 amino acids of RT, and 288 amino acids of integrase for 727 isolates from the six subtypes for which the most isolates were available.** A dotted line is drawn at an entropy level of 0.5 bits – a level at which the correct amino acid at a position could be predicted with nearly 90% certain.

Among the 741 ARV-naïve HIV-1 group M isolates belonging to the six most common subtypes, the proportion of positions with ≥ 0.5% variability relative to the consensus subtype amino acid was lower for integrase (34.7%) compared with protease (40.0%; p < 0.001) and RT (37.2%; p < 0.001). The mean level of Shannon's entropy at all positions calculated using the same 741 pol sequences was also significantly lower for integrase (0.11 ± 0.23) than for RT (0.15 ± 0.31) and protease (0.16 ± 0.31) (Figure 2). For 92.7%, 89.8%, and 88.2% of integrase, RT, and protease positions across the six most common subtypes, there was an entropy level below 0.5 bits meaning that at these positions, the correct amino acid could be predicted with approximately 90% certainty.

### Catalytic core domain (CCD)

Of the 162 amino acid catalytic core domain (CCD) residues encompassing positions 51 to 212, 108 (66%) were nonpolymorphic (prevalence ≤ 0.5%) among group M sequences. Based on the published crystallographic structure of the integrase CCD bound to prototype diketo acid active site inhibitor (5CITEP) [7], a putative integrase inhibitor binding pocket containing the active site residues and D64, C65, T66, H67, E92, D116, Q148, V151, E152, N155, K156, and K159 has been proposed [15,16]. These residues were nonpolymorphic, with the exceptions of the conservative mutations V151I, K156N, and K156R, each of which occurred in 1% of sequences (Figure 1). Six otherwise normal isolates, however, contained the active site mutation E152K. Similar variation was not observed at the other active site residues (D64 and D116) suggesting that D152 may be particularly prone either to sequencing error or to RNA editing as the observed mutation could result from unhindered APOBEC3F activity.

A flexible loop region encompassing F139 to G146 and an amphipathic alpha-helix (α4) extending from S147 to V165 are involved in both the direct binding and correct positioning of viral DNA to the integrase catalytic residues. The flexible loop, which is generally poorly resolved in crystallographic structures, is completely conserved in group M sequences with the exception of F139Y, which occurred in 12 subtype A infected persons. The conserved positively charged residues in the amphiphathic α4 helix including Q148, E152, N155, and K159 are positioned to contact negatively charged viral DNA molecules [17]. Site directed mutagenesis studies suggest that other conserved positively charged CCD residues including Q62 and N120 also participate in critical viral DNA binding [18].

Among the CCD mutations shown to directly reduce raltegravir or elvitegravir susceptibility – H51Y, T66I, E92Q, F121Y, G140S, Y143C/H/R, Q146P, S147G, Q148H/R/K, S153Y, N155H/S, and E157Q [19-21] – only positions 153 and 157 are polymorphic (prevalence ≥ 0.5%) with S153A and E157Q each present in 1% of sequences (Figures 1). In contrast, as summarized in the next paragraph, mutations at the remaining INI-resistance positions were rare.

The INI-resistance mutation H51Y was present in one subtype A isolate; H51Q (n = 3) and H51P (n = 2) were present in five isolates. T66A (n = 2) and T66S (n = 1) were present in three subtype C isolates. T66P was present as part of an electrophoretic mixture in one subtype B and one subtype F isolate. E92G (n = 2), E92D (n = 1), and E92A (n = 1) were present in four isolates. F121S (n = 2) and F121L (n = 1) were present in three isolates. G140E was present in one subtype G isolate. Y143H was present in three subtype C isolates and one subtype D isolate. The INI-resistance mutation S147G was present in one CRF01_AE isolate and in one subtype C isolate; S147R was present in one subtype B isolate. The INI-resistance mutations Q148H (subtype G) and Q148K (CRF02_AG) were each present in one isolate. The INI-resistance mutation, N155H was present in one subtype B isolate; N155D was present in one subtype D isolate.

Among mutations selected by raltegravir or elvitegravir that have not been shown to directly reduce susceptibility, L74R, Q95K, E138A/K, and H183P were conserved, whereas V54I, L68V, L74M, T97A, V151I, G163R, and I203M were present in approximately 1% to 2% of isolates from untreated persons (Figure 1).

In a crystallographic study containing a CCD dimer and the C-terminal LEDGF integrase-binding domain, 11 CCD residues were shown to participate in LEDGF binding: L102, T125, A128, A129, W131, W132, Q168, E170, H171, T174, and M178 [22]. All but T125 and H171 were nonpolymorphic in group M sequences. The side chains of A128, A129, W131, W132, E170, T174, and M178 participated in LEDGF binding; in contrast the main chains of the conserved position 168 and of the polymorphic positions 125 and 171 participated in LEDGF binding.

### N-terminal domain (NTD)

Of the 50 NTD residues, 25 (50%) were nonpolymorphic among group M sequences (Figure 1). The HHCC zinc-binding motif at positions 12, 16, 40, and 43 were nonpolymorphic. This motif interacts with residues 150–196 of an adjacent monomer. The interface between the NTD and the CCD within each monomer involves the connecting residues 47 to 55 (which are poorly resolved crystallographically) and hydrophilic contacts between the NTD side chains R20 and K34 and the CCD side chains T206, Q209, and E212 [6]. Of these interacting residues, R20K, K34R, and T206S occurred in 4%, 2%, and 16% of group M sequences, respectively, whereas Q209 and E212 were invariant among group M sequences. The polar NTD residues K14, N18, and Q44, and the polar CCD residues K160, Q168, and K186 contribute to the dimer-dimer interface in the tetrameric NTD-CCD crystal structure. group M variants at these positions include K14R in 31% of sequences and K160R/Q in 2% of sequences.

### C-terminal domain (CTD)

Of the 76 CTD residues, 32 (58%) were nonpolymorphic among group M sequences. A crystallographic structure containing the linked CCD and CTD domains demonstrated a Y-shaped dimer in which there are two symmetrically interfacing CCDs at the base and two symmetrically separated CTDs at the "Y" branches [5]. The residues linking the CCD to the CTD are part of an extended alpha helix encompass residues 195 to 225 [5]. Residues 270–288 were not delineated in the CCD-CTD crystal structure.

An electrostatic potential map identifies a strip of positively charged residues extending from the CCD active site through K159, K186, R187, and K188 in the CCD of one monomer towards the CTD of the other monomer [5]. Positively charged CTD residues include K215, K219, R228, R231, K236, K244, K258, R262, R263, K266, R269, K273, and R284. Whereas K215N/R, K219N/Q, R269K, and R284G are reported polymorphisms, the remaining positively charged residues were nonpolymorphic. Many of these positively charged residues have been implicated in DNA binding and been found to be essential to integrase function [23].

The nonpolymorphic mutation R263K has been shown to reduce elvitegravir susceptibility by five-fold. Its effect on raltegravir has not been reported. Y226C/D/F/H, S230N/R, and D232N have been selected *in vitro *or *in vivo *by raltegravir and/or elvitegravir [24,25]. Of these mutations, S230N has been reported in 2.0% of untreated isolates. The conservative substitution D232E has also been observed in 2.0% of untreated isolates. R263K (n = 2) and R263G (n = 1) were present in three isolates.

### Amino acid covariation

Ninety-eight pairs of amino acids were significantly associated with one another at a false discovery rate of 0.05. Fifty-seven pairs of amino acids were from the same subdomain (CCD – 40 pairs, NTD – 10 pairs, and CTD – 7 pairs); 41 were from different subdomains (CCD-NTD – 17 pairs, CCD-CTD – 12 pairs, and CTD-NTD – 12 pairs). Five pairs of CCD residues were associated in two or more subtypes. E157Q, which decreases raltegravir and elvitegravir susceptibility, was associated with K160Q/T in subtypes A, B, C, and CRF02 and with K156N in three unrelated subtype D isolates. In contrast, the other uncommon polymorphisms in the α4 helix including V151I, S153A, M154I/L, I162V, G163E/K/R, and V165I were not found to covary with each other or with other integrase mutations.

The remaining pairs of residues that were associated in two or more subtypes included S119R and A91T/E in subtypes B, C, and CRF02; S119G and T122I in subtypes B and D; K219N and N222K in subtypes C and CRF02, and T124A and S283G in subtypes A and C. 17 of the CCD pairs involved position 119; whereas the next most commonly involved position was position 124, which was involved in 13 pairs. Position 119, which has been associated with target site specificity [26,27], is one of the most polymorphic residues with S, P, T, G, and R occurring in 80%, 11%, 4%, 3%, and 2% of isolates, respectively.

## Discussion

The development of clinically active INIs is a remarkable therapeutic success story. Two decades of biochemical and biophysical studies established the fundamental mechanisms of HIV-1 integrase activity [1,3], facilitated the development of high-throughput inhibitor screening assays [28,29], and led to the identification of highly active, bioavailable, and safe INIs [30-33]. Several clinical trials have demonstrated the efficacy of these compounds for both initial and salvage ARV therapy [34-39].

The clinically active INIs are competitive inhibitors of target DNA and indeed there is much overlap between the sites associated with target DNA binding and INI binding [28,40]. Several aspects of HIV-1 integration and its inhibition, however, remain poorly understood. The relative positioning of the three separate integrase domains and the three-dimensional structure of the active multimeric form of the enzyme are not known. In addition, although there is a structure of HIV-1 integrase bound to the diketo acid structural homolog 5CITEP [7], there are no structures of integrase bound to a DNA substrate or to one of the recent classes of INIs.

Nonetheless, there is an increasing body of literature describing which integrase mutations are selected by INIs *in vitro *and *in vivo *and which integrase mutations reduce INI susceptibility. Some of these data are from studies of the early prototype INIs such as the diketo inhibitors S1360 and L-708,906 and the napthyridine carboxamide inhibitor L870,810 [4,9,30,31,41,42]. However, most are from studies of the licensed INI raltegravir or of elvitegravir, an INI in phase III clinical development including several clinical reports detailing the mutations developing in about 150 patients experiencing virological failure while receiving raltegravir or elvitegravir [19-21,24,33,43-50].

Several concepts of INI resistance have emerged from these studies. First, a large number of mutations have been selected by INIs either *in vitro *or *in vivo *(reviewed in [9]). Second, most of mutations that directly reduce INI susceptibility occur close to the active site residues D64, D116, and E152 in the vicinity of the pocket to which 5CITEP binds [7,15,16,51]. Third, many mutations appear to accessory in that they have little or no effect on susceptibility by themselves. Fourth, for both raltegravir and elvitegravir, virological failure has generally been accompanied by two or more INI-resistance mutations and decreases in susceptibility ranging from > 10-fold to > 100-fold [20,21,25,52]. Fifth, there is extensive overlap among the integrase mutations associated with raltegravir and elvitegravir resistance [19-21,33], as well as between these newer INIs and the earlier generation of INIs [9,42,53].

Our study characterized the distribution of integrase amino acid variants among more than 1,800 group M HIV-1 isolates from more than 1,500 INI-naïve individuals. Polymorphism rates equal or above 0.5% were found for 34% of the CCD positions, 42% of the CTD positions, and 49% of the NTD positions. Among 741 ARV-naïve HIV-1 group M isolates for which complete *pol *sequences were available, integrase displayed higher levels of amino acid conservation compared with RT and protease by several measures of diversity including mean inter- and intra-subtype diversity and Shannon's entropy.

Nearly all INI-resistance mutations known to directly reduce HIV-1 susceptibility were nonpolymorphic including H51Y, T66I, E92Q, F121Y, G140S, Y143C/H/R, Q146P, S147G, Q148H/R/K, S153Y, N155H/S, and R263K. Most accessory INI-resistance mutations including L74R, Q95K, E138A/K, H183P, Y226C/D/F/H, S230R, and D232N were also nonpolymorphic. The vast majority of integrase residues assigned specific roles such as the CCD active site residues, the NTD zinc binding residues, the residues involved in LEDGF/p75 binding, and the many positively charged CTD residues were also nonpolymorphic.

In contrast, E157Q – which has been reported to be selected by raltegravir [44] and to reduce elvitegravir susceptibility by about 3 to 6-fold [19,33] – occurred in about 1% of untreated persons almost always in combination with the uncommon mutations K156N or K160Q. In addition, several accessory INI-resistance mutations including V54I, L68V, L74M, T97A, V151I, G163R, I203M, and S230N [24,25,45,46,49,50,54] also displayed levels of polymorphism ranging from 1% to 2%. Recent independent surveys of isolates from smaller numbers of INI-naïve individuals confirmed these results frequently finding E157Q as well as L74M, T97A, V151I, and I203M in small proportions of untreated persons [55-59].

Mutations that have been selected *in vitro *or *in vivo *primarily by earlier INI compounds such as L-708,906, S-1360, and L-870,810 but which appear to be less essential for raltegravir or elvitegravir resistance include the highly polymorphic mutations V72I [31], V165I [41], and V201I [41]; the minimally polymorphic mutation M154I [30]; and the nonpolymorphic mutations T125K [31], A128T [41], and K160D [41]. The significance of these residues to the current generation of INIs is not yet known.

The high level of integrase sequence conservation results from a combination of functional and structural constraints. The functional constraints result from this enzyme's multiple functions including 3' processing, strand transfer which requires simultaneous interactions with both viral and host DNA, and binding to other components of the pre-integration complex including LEDGFp75. The structural constraints include the incompletely defined interactions among the different integrase subdomains and among the monomers that contribute to the multimeric form of the enzyme. HIV-1 integrase also contains a somewhat lower number of well-defined CTL epitopes (n = 11) relative to its size compared with protease (n = 7) and RT (n = 41), which could also contribute to its relatively higher level of sequence conservation compared with these two other enzymatic targets of ARV therapy [60].

## Supplementary Material

Additional File 1 Accession IDs Click here for file

Additional File 2 Variation by subtype Click here for file
