# Supplementary material for: Natural variation of HIV-1 group M integrase: Implications for a new class of antiretroviral inhibitors
Source: Retrovirology. 2008 Aug 7;5:74. doi: 10.1186/1742-4690-5-74 (PMC2546438; doi:10.1186/1742-4690-5-74)
Supplement: Additional File 1 — Accession IDs [file 1742-4690-5-74-S1.doc]

>M29975

TTCATTGGCAGGATAGAAGAAGCACAAGAAGAACATGATAGGTATCACAGTAACTGGAGAAATCTAGCAGACACATTTGGATTGCCACAAATAGTAGCTAAAGAAATTGTAGCAATGTGCCCAAAATGTCAAGTAAAAGGGGAACCAATACATGGACAAGTAGATGCTTCACCAGGAGTGTGGCAGATGGACTGCACACATATAGAAGGAAAAATAGTGATAGTAGCGGTCCATGTAGCCAGTGGGTTTATAGAAGCAGAGGTTATCCCTAGGGAAACAGGAAAAGAGACAGCAAAGTTCTTGTTAAAAATAATAGGAAGATGGCCCATCACTCACCTCCATACAGATAATGGACCAAATTTCACTTCTCAGGAAGTAGCTGCTATGTGCTGGTGGGGAAAGGTAGAACACACAACGGGGGTACCATATAATCCACAGTCCCAGGGATCTATAGAAAGTATGAACAAACAATTGAAAGAGATAATTGGAAAAATAAGAGATGACTGTCAATATACAGAAACAGCAGTACTTATGGCCTGCCACATTCACAATTTTAAAAGAAAGGGAGGAATAGGGGGGCTAACAGCTGCAGAGAGACTAATAAATATGATAACAACACAATTAGAAATCAACACTCTACAAACCAAAATCCAAAAAATTTTGAATTTTAGAGTCTACTACAGAGAAGGCAGAGATCCAGTGTGGAAGGGACCTGCTCGCCTGATCTGGAAAGGAGAAGGCGCGGTAGTTCTCAAGGAAGGTGAAGAACTGAAGGTAGTTCCGAGAAGGAAAGCAAAAATCATAAAAGACTAT

>M30931

TTTTTAGAAAAAATAGAAGAAGCCCAGGAGGAACATGAAAGGTACCATAATAATTGGAGGAACTTAGCAGACACTTATGGGCTACCACAAATTGTGGCAAAAGAAATAGTAGCCATGTGTCCAAAATGTCAGATAAAAGGGGAACCAGTCCATGGGCAAGTAGATGCCTCGCCAGGGGTATGGCAAATGGACTGTACACATTTAGAAGGCAAGGTAATCATAGTAGCAGTCCATGTAGCCAGTGGATTCATAGAAGCAGAAGTTATACCTAGAGAAACAGGGAAAGAAACAGCAAAATTTTTATTAAAGATACTAAGTAGATGGCCCATAACCCAACTGCATACAGACAATGGACCCAATTTTACGTCTCAAGAAGTAGCAGCAATGTGTTGGTGGGGAAAAATAGAACACACCACAGGTGTACCCTATAACCCTCAATCACAAGGCTCTATAGAGAGTATGAATAAACAGTTAAAAGAAATAATTGGGAAAATAAGAGATGACTGTCAATACACAGAAACAGCAGTACTTATGGCATGCCACATCCACAATTTTAAAAGAAAGGGAGGAATAGGGGGGTTAACACCGGCAGAGAGATTAATCAATATGATTACTACACAATTAGAATTACAACACCTACAAACCAAAATTCAAAAAATTTTAAATTTTAGAGTCTACTACAGAGAAGGGAGAGATCCTGTCTGGAAAGGACCAGGACAGTTAATTTGGAAAGGGGAAGGTGCAGTGGTCATCAAAGGAGGTGTGGAATTAAAAGAATACCCAAGAAGGAAAGCAAAAATTATAAAGGATTAT

>M66437

TTCTTAGATAGAATAGAAGAAGCACAAGATGACCATGCAAAGTACCATAACAATTGGAGAAGTATGGTACAGGAATTTGGATTACCTAATATAGTAGCAAAAGAGATAGTAGCGGCATGTCCCAAATGCCAAATAAGAGGAGAACCTAAGCATGGACAGGTAGACGCCTCCATTGAAACTTGGCAGATGGACTGCACCCATTTAGAAGGAAAAGTTATAATAGTAGCAGTACATGTAGCCAGTGGATTCATAGAAGCAGAGGTGATCCCAAGAGAAACTGGGAAGGAGACAGCACACTTTCTGCTGAAACTGTTAGCAAGATGGCCAGTGAAACATCTACACACTGATAATGGCCCAAACTTTACCTCTCAGAATGTGGCAGCGGTGTGCTGGTGGGGTAATATAGAGCACACCACTGGAATACCTTATAACCCACAGTCACAGGGTAGTGTAGAAAGCATGAACAGACAGCTCAAGGAAATCATCTCTCAAATAAGAGATGATTGTGAGAGATTGGAGACAGCAGTGCAAATGGCTACGCATATCCACAATTTTAAAAGAAAGGGAGGAATAGGGGGTATCTCTAGTGCAGAAAGATTGGTTAATATGCTAACAACACAACTAGAACTAAATACTCTACAAAACCAAATCCAAAAAATTTTGAATTTTAAGGTCTACTACAGAGAAGGTAGAGATCCAGTGTGGAAAGGACCAGCGCGACTCATCTGGAAAGGAGAAGGCGCGGTGGTAATTAAAGAGGGGGAAGACATCAAGGTAGTCCCCAGGAGAAAGGCTAAGATTATCAAAGATTATGGAGAGAGAATGGGTGGATCGGAGGCGGTACAGGGGCGG

>L40990

TTTATAGGAAGAATAGAAGAAGCTCAAGAAGAACATGACAGATATCATAGTAATTGGAAAAATTTAGCGGACACATTTGGATTACCACAGATAGTTGCCAAAGAGATAGTAGCAATGTGTCCAAATGCCAAAATAAAAGGGGAACCAATACATGGGCAAGTAGACGCCTCCCCAGGGGTATGGCAAATGGACTGCACGCATGTAGAAGGAAAAATAGTCATAGTAGCAGTTCATGTGGCCAGCGGATTCATAGAAGCAGAAGTCATACCTAGGGAAACAGGAAGAGAGACAGCCAAATTCCTTCTAAAAATATTGAGTAGATGGCCAATAGTCCAGTTACACACAGATAATGGACCAAATTTTACTTCCCAAGAGGTAGCGGCAATCTGTTGGTGGGGAAAGATAGAGCACACCACAGGAGTGCCATACAATCCACAGTCACAGGGATCAGTAGAAAGCATGAACAAACAACTCAAAGAAATAATTGGAAAAATAAGAGATGATTGTCAGTATACAGAAACAGCAGTACTTATGGCATGCCACATCCACAATTTTAAAAGAAAGGGAGGAATAGGGGGGTTAACACCAGCAGAAAGACTAATTAATATGATAACAACACAATTAGAACTACAAACATTACAAACCAAAATTCAAAAAATTTTAAATTTTAGAGTCTACTACAGAGAAGGGAGAGATCCAGTGTGGAAGGGACCAGCACAATTAATCTGGAAAGGGGAAGGTGCAGTGGTCATCAAAGAAGGAGAAGAGCTGAAAATTGTCCCAAGAAGAAAAGCAAAAATCATCAAAGATTATGGAAAAAGAATGGCAGGTGATCCCTGCATTAAATCT

>U04005

TTCCTGGACAGAATTGAAGAAGCACAAGAAGAGCATGATAAATACCATGCCAATTGGAGGAGTATGCAACAAGAATTTGGCTTACCTGCTATAGTAGCAAAAGAAATAGTAGCGGCATGTCCTAAATGCCAGATAAAAGGGGAGTCTGTACATGGGCAAGTAGATGCTAGTCCAGGAGTGTGGCAAATGGATTGTACACACCTAGAAGGAAAAATCATCATAGTGGCTGTTCATGTTGCTAGTGGATTTATAGAAGCAGAAGTAATCCCACAGGAAACAGGAAAAGCTACAGCACACTTCCTGCTGAAGTTAGCCAGCAGATGGCCCATCACACAGCTGCATACAGATAATGGTACCAACTTTACTAGCCAGCAAGTAGCAGCTATTTGCTGGTGGGGAAAAATAGAGCACACATTTGGGGTACCCTACAACCCCCAAAGTCAGGGAGTAGTAGAATCTATGAATAAGCAGTTAAAAGAAATCATAGGACAAATTAGAGATGATGCAGAAAGATTGGAAACAGCAGTCATAATGGCAGTGCACATTCACAATTTTAAAAGAAAAGGGGGGATTGGGGGGTACTCTGCAGCAGAAAGACTAATCAATATAATACATACAGAACTAGAAACCAAAACACTTCAACAAAAAATTTCAAAAATTCAAAATTTTCGGGTCTATTACAGGGAAGGCAGAGACCCTGTGTGGAAAGGTCCGGCTAAGCTCATCTGGAAAGGAGAAGGAGCAGTAGTTATACAAGAACAGGGTGAATTGAAAACAATCCCAAGAAGAAAAGCCAAAATTATAAAAGATTATGGAAAAGCATTG

>L20587

TTCCTAGAAGGAATAGACCAGGCACAAGAAGATCATGAAAAATATCATAGTAATTGGAAAGCACTAGCTAGTGAATTTGGACTACCACCAGTGGTGGCCAAGGAAATCATTGCTAGCTGTCCTAAATGTCATATAAAAGGGGAAGCAATTCATGGTCAGGTAGACTGCAGTCCAGAAGTATGGCAAATAGATTGCACACATATGGAAGGCAAAATCATAATAGTTGCTGTCCATGTGGCAAGTGGGTTCATAGAAGCAGAAGTGATACCAGCAGAAACAGGACAAGAAACTGCCTACTTCCTGTTAAAACTGGCTGCAAGATGGCCTGTTAAAGTAATACATACAGACAACGGGCCTAATTTTACAAGTACAACTATGAAGGCTGCATGTTGGTGGGCCAACATACAACATGAGTTTGGAATACCATATAATCCACAAAGTCAAGGAGTAGTAGAAGCCATGAATAAGGAATTAAAATCAATTATACAGCAGGTGAGGGACCAAGCAGAACACTTAAGAACAGCAGTACAAATGGCAGTATTTGTTCACAATTTTAAAAGAAAAGGGGGGATTGGGGGGTACACTGCAGGAGAAAGGATAATAGACATATTAGCATCACAAATACAAACAACAGAATTACAAAAACAAATTTTAAAANTTCACAAATTTCGGGTCTATTACAGAGACAGCAGAGACCCTATCTGGAAAGGACCGGCACAGCTCCTGTGGAAAGGTGAGGGAGCAGTAGTCATACAAGATAAGGGAGACATTAAGGTAGTACCAAGAAGGAAGGCAAAAATAATCAGAGAGTATGGAAAACAGATGGCAGGTACTGATAGTATGGCAAGTGGACAGACAGAA

>M30502

TTCCTGGAAAAGATAGAGCCCGCTCAAGAGGAACATGAAAAATATCATAGCATTATAAAAGAACTAACCCATAAATTTGGAATACCCCTTCTAGTAGCAAGACAGATAGTAAACTCATGTGCCCAATGCCAACAGAAAGGAGAAGCCATACATGGGCAAGTAAATGCAGAAATAGGCGTTTGGCAAATGGACTACACACACTTAGAAGGAAAAATCATTATAGTAGCAGTACATGTTGCAAGTGGATTCATAGAAGCAGAAGTCATCCCACAGGAATCAGGAAGGCAGACAGCACTCTTCCTATTAAAACTGGCCAGTAGGTGGCCAATAACGCACTTGCACACAGACAATGGCCCCAACTTCACTTCACAGGAAGTGAAGATGGTGGCATGGTGGGTAGGTATAGAACAATCCTTTGGAGTACCTTACAACCCACAAAGCCAGGGAGTAGTAGAAGCAATGAATCACCACCTAAAGAATCAGATAAGTAGAATTAGAGAACAGGCAAATACAATAGAAACAATAGTACTGATGGCAGTTCATTGCATGAATTTTAAAAGAAGGGGAGGAATAGGGGATATGACCCCAGCAGAAAGACTAATCAACATGATTACCACAGAACAAGAAATACAATTCCTCCAAAGAAAAAATTCAAATTTTAAAAATTTCCAGGTCTATTACAGAGAAGGCAGAGATCAGCTGTGGAAAGGACCTGGTGAACTACTGTGGAAGGGAGAAGGAGCAGTCATAGTCAAGGTAGGGACAGACATAAAAGTAGTACCAAGAAGGAAGGCCAAGATTATCAGGGACTATGGA

>M32741

TTCTTGGAAAAGATAGAGCCAGCACAAGAAGAACATGATAAATACCATAGTAATGTAAAAGAATTGGTATTCAAATTTGGATTACCCAGACTAGTGGCCAAACAGATAGTAGACACATGTGATAAATGTCATCAGAAAGGAGAAGCTATACATGGGCAGGTAAATTCAGATCTAGGGACTTGGCAAATGGATTGTACCCATCTAGAGGGAAAAATAATCATAGTTGCAGTACATGTAGCTAGTGGATTCATAGAAGCAGAAGTAATTCCACAAGAGACAGGAAGACAGACAGCACTATTTCTGTTAAAATTGGCAAGCAGATGGCCTATTACGCATCTACACACAGATAATGGTGCCAACTTTGCTTCGCAAGAAGTAAAGATGGTTGCATGGTGGGCAGGGATAGAGCACACCTTTGGGGTACCATACAATCCACAGAGTCAGGGAGTAGTGGAAGCAATGAATCACCATCTAAAAAATCAAATAGATAGAATCAGGGAACAAGCAAATTCAATGGAAACCATAGTATTAATGGCAGTTCATTGCATGAATTTTAAAAGAAGGGGAGGAATAGGGGATATGACTCCAGCAGAAAGATTACTTAACATGATCACTACAGAACAAGAAATACAATTCCAACAATCAAAAAACTCAAAATTTAAAAATTTTCGGGTCTATTACAGAGAAGGCAGAGATCAGCTGTGGAAAGGACCTGGTGAGCTATTGTGGAAAGGGGAAGGAGCAGTCGTCTTAAAGGTAGGGACAGACATTAAGGTAGTACCCAGAAGAAAGGCTAAGATTATCAAAGATTATGGA

>K02013

TTTTTAGATGGAATAGATAAGGCCCAAGATGAACATGAGAAATATCACAGTAATTGGAGAGCAATGGCTAGTGATTTTAACCTGCCACCTGTAGTAGCAAAAGAAATAGTAGCCAGCTGTGATAAATGTCAGCTAAAAGGAGAAGCCATGCATGGACAAGTAGACTGTAGTCCAGGAATATGGCAACTAGATTGTACACATTTAGAAGGAAAAGTTATCCTGGTAGCAGTTCATGTAGCCAGTGGATATATAGAAGCAGAAGTTATTCCAGCAGAAACAGGGCAGGAAACAGCATACTTTCTTTTAAAATTAGCAGGAAGATGGCCAGTAAAAACAATACATACAGACAATGGCAGCAATTTCACCAGTACTACGGTTAAGGCCGCCTGTTGGTGGGCGGGAATCAAGCAGGAATTTGGAATTCCCTACAATCCCCAAAGTCAAGGAGTAGTAGAATCTATGAATAAAGAATTAAAGAAAATTATAGGCCAGGTAAGAGATCAGGCTGAACATCTTAAGACAGCAGTACAAATGGCAGTATTCATCCACAATTTTAAAAGAAAAGGGGGGATTGGGGGGTACAGTGCAGGGGAAAGAATAGTAGACATAATAGCAACAGACATACAAACTAAAGAATTACAAAAACAAATTACAAAAATTCAAAATTTTCGGGTTTATTACAGGGACAGCAGAGATCCACTTTGGAAAGGACCAGCAAAGCTCCTCTGGAAAGGTGAAGGGGCAGTAGTAATACAAGATAATAGTGACATAAAAGTAGTGCCAAGAAGAAAAGCAAAGATCATTAGGGATTATGGAAAACAGATGGCAGGTGATGATTGTGTGGCAAGTAGACAGGATGAGGAT

>U46016

TTTCTAGATGGAATAGATAAGGCTCAAGAAGAGCATGAAAAATATCACAGCAATTGGAGAGCAATGGCTAATGAATTTAATATCCCACCCGTAGTACCCAAAGAAATAGTAGCTTGCTGTGATAAATGTCAGCTAAAAGGGGAAGCCATACATGGACAAGTAAATTGTAGTCCAGGGATATGGCAATTAGATTGTACACACTTAGAAGGGAAAATCATCCTGGTAGCAGTCCATGTAGCCAGTGGCTACATAGAGGCAGAGGTTATTCCAGCAGAAACAGGACAAGAAACAGCATACTTTCTACTAAAATTAGCAGGGAGATGGCCAGTCAGGGTAATACATACAGATAATGGCAGTAACTTCACCAGTAATGCAGTTAAAGCAGCCTGTTGGTGGGCAGGTATTCAACAGGAATTTGGAATTCCCTACAATCCCCAAAGTCAAGGAGTAGTAGAATCTATGAATAAAGAATTAAAGAAAATCATAGGGCAGGTAAGAGAACAAGCTGAGCACCTTAAGACAGCAGTACAAATGGCAGTATTCATTCACAATTTTAAAAGAAGAGGGGGGATTGGGGGGTACAGTGCAGGGGAAAGAATAATAGATATAATAGCATCAGACATACAGACTAAAGAACTCCAAAACCAAATTTTAAAAATTCAAAATTTTCGGGTTTATTACAGAGACAGCAGAGACCCTATTTGGAAAGGACCAGCCAAACTACTCTGGAAAGGTGAAGGGGCAGTAGTAATACAAGATAATAGTGACATAAAGGTAGTACCAAGGAGGAAAGCAAAAATCATTAGGGATTATGGAAAACAGATGGCAGGTGCTGATTGTGTGGCAGGTAGACAGGATGAAGAT

>D10112

TTTTTAGATGGAATAGATAAGGCCCAAGAAGAACATGAGAAATATCACAGTAATTGGAGAGCAATGGCTAGTGATTTTAACCTGCCACCTGTAGTAGCAAAAGAAATAGTAGCCAGCTGTGATAAATGTCAGCTAAAAGGAGAAGCCATGCATGGACAAGTAGACTGTAGTCCAGGAATATGGCAGCTAGATTGTACACATTTAGAAGGAAAAGTTATCCTGGTAGCAGTGCATGTAGCCAGTGGATATATAGAAGCAGAAGTTATTCCAGCAGAGACAGGGCAAGAAACAGCATACTTTCTCTTAAAATTAGCAGGAAGATGGCCAGTAAAAACAATACACACAGACAATGGTGGCAATTTCATCAGTACTACAGTTAAGGCCGCCTGTTGGTGGGCAGGGATCAAGCAGGAATTTGGCATTCCCTACAATCCCCAAAGTCAAGGAGTAGTAGAATCTATGAATAATGAATTAAAGAAAATTATAGGACAGGTAAGAGATCAAGCTGAACATCTTAAGACAGCAGTACAAATGGCAGTATTCATCCACAATTTTAAAAGAAAAGGGGGGATTGGGGGGTACAGTGCAGGGGAAAGAATAATAGACATAATAGCAACAGATATACAAACTAAAGAATTACAAAAACAAATTACAAAGATTCAAAATTTTCGGGTTTATTACAGGGACAGCAGAGATCCACTTTGGAAAGGACCAGCAAAGCTTCTCTGGAAAGGTGAAGGGGCAGTAGTAATACAAGATAATAGTGACATAAAAGTAGTGCCAAGAAGAAAAGCAAAGATCATTAGGGATTATGGAAAACAGATGGCAGGTGATGATTGTGTGGCAAGTAGACAGGATGAGGAT

>D00835

TTCCTAGAAAAGATAGAGCCTGCTCAGGAAGAACATGAAAAATATCATACCAATGTAAAAGAGCTATGCCATAAATTTGATATACCCCAACTAGTGGCAAGACAAATAGTAAACACATGTGCCCAATATCAACAGAAAGGGGAGGCTATACATGGGCAAGTAAATGCAGAAGTGGGCACCTGGCAAATGGACTGCACACATCTAGAGGGAAAAATCATCATAGTAGCAGTACATGTTGCAAGTGGATTTATAGAAGCAGAAGTCATCCCACAGGAATCAGGAAGGCAAACAGCACTCTTCCTATTAAAATTGGCTAGCAGGTGGCCAATAACACACTTACACACAGATAATGGTGCCAACTTCACTTCACAGGAAGTAAAGATGGTAGCATGGTGGGTAGGCATAGAACAAACCTTTGGAGTACCTTACAACCCACAAAGCCAAGGAGTAGTAGAAGCAATGAATCACCACCTAAAAAATCAGATAAGTAGAATTAGAGAACAGGCAAATACAGTAGAAACAATAGTACTAATGGCAGTTCATTGCATGAATTTTAAAAGAAGGGGAGGAATAGGGGATATGACTCCATCAGAAAGACTAATCAATATGATCACCACAGAACAAGAGATACAATTCCTCCAAGCCAAAAATTCAAAATTAAAAAATTTTCGGGTCTATTTCAGAGAAGGCAGAGATCAGTTGTGGAAAGGACCTGGGGAACTACTGTGGAAGGGAGACGGAGCAGTCATAGTCAAGGTAGGAACAGATATAAAAATAATACCAAGGAGGAAAGCCAAGATCATCAGAGACTATGGA

>U21135

TTTTTAGATGGGATAGATAAGGCCCAAGAAGAACATGAGAAATATCACAGTAATTGGAGAGCAATGGCTAGTGATTTTAACCTGCCACCTGTAGTAGCAAAAGAAATAGTAGCCAGCTGTGATAAATGTCAGCTAAAAGGAGAAGCCATGCATGGACAAGTAGACTGTAGTCCAGGAATATGGCAACTAGATTGTACACATTTAGAGGGAAAAATTATCCTGGTAGCAGTTCATGTAGCCAGTGGATATATAGAAGCAGAAGTTATTCCAGCAGAGACAGGGCAAGAAACAGCATACTTTATCTTAAAACTAGCAGGAAGATGGCCAGTAAAAACAATACATACAGACAATGGCAGCAATTTCACCAGTACTACGGTTAAGGCCGCCTGTTGGTGGGCGGGGATCAAGCAGGAATTTGGCATTCCCTACAATCCCCAAAGTCAAGGAGTGATAGAATCTATGAATAAAGAATTAAAGAAAATTATAGGACAGGTAAGAGATCAAGCTGAACATCTTAAGACAGCAGTACAAATGGCAGTATTCATCCACAATTTTAAAAGAAAAGGGGGGATTGGGGGGTACAGTGCAGGGGAAAGAATAATAGACATAATAGCAACAGACATACAGACTCAACAATTACAAAAACAAATTACAAAAATTCAAAATTTTCGGGTCTATTACAGGGACAGCAGAGATCCACTTTGGAAAGGACCAGCAAAGCTTCTCTGGAAAGGTGAAGGGGCAGTAGTAATACAAGATAATAGTGACATAAAAGTAGTGCCAAGAAGAAAAGCAAAGATCATTAGGGATTATGGAAAACAGATGGCAGGTGATGATTGTGTGGCAAGTAGACAGGATGAGGAT

>U54771

TTTTTAGATGGGATAGATAAGGCTCAAGAAGAACATGAAAGATATCACAGCAATTGGAGAACAATGGCTAGTGATTTTAATTTGCCACCTATAGTAGCAAAGGAAATAGTAACCAACTGTGATAAATGTCAACTAAAAGGGGAAGCTATGCATGGACAAGTAGACTGTAGTCCAGGGATATGGCAATTAGATTGCACACATCTAGAAGGAAAAGTCATCCTGGTAGCAGTCCACGTGGCCAGTGGATATATAGAAGCAGAAGTTATCCCAGCAGAAACAGGACAGGAGACAGCATACTTTCTGCTAAAACTAGCAGGAAGATGGCCAGTAAAAGTAATACACACAGACAACGGTAGCAATTTCACCAGCGCTGCAGTTAAAGCAGCCTGTTGGTGGGCCAATGTCCAACAGGAATTTGGGATCCCCTACAATCCCCAAAGTCAAGGAGTAGTAGAATCTATGAATAAGGAATTAAAGAAAATCATAGGGCAGGTAAGAGAGCAAGCTGAACACCTTAAAACAGCAGTACAAATGGCAGTATTCATTCACAATTTTAAAAGAAAAGGGGGGATTGGGGGGTACAGTGCAGGGGAAAGAATAATAGACATAATAGCAACAGACATACAAACTAAAGAATTACAAAAACAAATTACAAAAATTCAAAATTTTCGGGTTTATTACAGGGACAGCAGAGACCCAATTTGGAAAGGACCAGCAAAACTACTCTGGAAAGGTGAAGGGGCAGTAGTAATACAAGACAATAGTGATATAAAAGTAGTACCAAGAAGAAAAGCAAAGATCATTAGGGATTATGGAAAACAGATGGCAGGTGATGATTGTGTGGCAGGTAGACAGGATGAGGAT

>U42720

TTTCTGGAAGGCATAGATAAAGCTCAAGAGGACCATGATAAATATCATAGCAATTGGAGATCATTAGCAGATGAATACAATCTTCCCCCTATTGTGGCTAAAGAAATTATAGCACAGTGTGATAAATGTCACGTAAAGGGAGAAGCCAGGCATGGACAAGTAGACTGCAGTCCAGGAATATGGCAAGTAGATTGCACCCATTTAGAAGGTAAAGTAATCATAGTAGCAGTGCATGTATCTAGTGGCTTCATAGAAGCTGAAGTAATGGCAGATGAGACAGGGAAAAGTACAGCATACTTCCTGTTAAAATTAGCCAGCAGATGGCCAGTAAAAACAATACACACTGACAATGGAGCTAATTTCACAAGTGCAGCAGTAAAAGCGGCATGTTGGTGGGCTAATATCCAACAGGAATTTGGAATACCATACAATCCACAAAGTCAAGGAGTAGTGGAATCCATGAATAAACAATTGAAGCAAATTATAGGACAAATTAGAGACCAAGCAGAACAATTAAAGACAGCAGTAGTAATGGCAGTGCACATTCACAATTTTAAAAGAAAAGGGGGGATTGGGGGGTACACACCTGGACAGAGAATATTAGACATACTAGCAACAGACATACAGACAACTCAATTACAAAATCAAATTTTAAAAATTCAACAATTTCGGGTTCATTACAGGGATAGCAGAGACCCTGTGTGGAAAGGACCAGCACAACTTCTGTGGAAAGGTGAAGGGGCAGTAGTCATCAAAGACCAAGAGGAAATTAAGGTAGTCCCTCGAAGGAAAGCAAAGATTATAAAGGAGTATAGAAAAAAGATAGAAGATAGAGATGATTTGGCAGGTAGACAGAATGAGGAT

>X52154

TTCCTAGATGGAATAGACAGGGCCCAAGAAGAACATGAAAGGTATCATAGTAATTGGAAAGCTATGGCTAGTGATTTTAATTTACCACCCATAGTAGCAAAAGAAATAGTGGCCCATTGTGATAAGTGCCAGGTAAAAGGAGAAGCCATGCATGGGCAGGTAGACTGTAGCCCAGGGATTTGGCAAGTAGATTGTACCCACCTAGAAGGCAAAGTGATCATAGTGGCAGTTCACGTAGCCAGTGGCTATATAGAAGCAGAAGTTATCCCAGCTGAGACAGGACAAGAAACAGCTTATTTCCTGTTAAAATTAGCAGGTAGATGGCCAGTAAAAACTATTCACACAGATAATGGGCCAAATTTTACAAGTGCTGCAGTCAAGGCTGCCTGTTGGTGGGCAGACATCAAGCAGGAATTTGGAATACCCTATAATCCACAGAGTCAAGGAGTGGTAGAATCCTTAAATAAAGAGCTAAAGAAAATAATAGGACAGGTTAGGGATCAAGCAGAACATTTAAAAACAGCAGTACAAATGGCAGTGTTCATTCACAATTTTAAAAGAAAAGGGGGGATTGGGGGGTACACTGCAGGGGAAAGAATAATAGACATAATAGCAACAGACATACAAACAAGCGAATTACAAAAACAAATTTTAAAAGTTCAAAAATTTCGGGTTTATTACAGAGACAGCAGAGACCCAATTTGGAAAGGACCGGCAACCTTACTGTGGAAAGGTGAAGGGGCAGTAGTGATCCAGGATCAAGGGGAACTAAAGGTAGTACCAAGAAGGAAAGCAAAGATCATTAGAGATTATGGAAAACAGATGGCAGGTGATGATTGTGTGGCAAGTAGACAGAATGAGGAT

>J04542

TTCCTGGAAAAGATAGAGCCCGCTCAAGAAGAACACGAAAAATATCATAGCAATATAAAAGAACTAACCCATAAATTTGGAATACCCCAACTAGTGGCAAGACAGATAGTAAACACATGTGCCCAATGCCAACAGAAAGGAGAAGCCATACATGGGCAAGTAAATGCAGAAATAGGCGTTTGGCAAATGGACTGCACACACTTAGAAGGAAAAATCATTATAGTAGCAGTGCATGTTGCAAGTGGATTCATAGAAGCAGAAGTCATCCCACAGGAATCAGGAAGGCAGACAGCACTCTTCCTATTAAAACTGGCCAGTAGGTGGCCAATAACACACTTGCACACAGACAATGGCCCCAACTTCACTTCACAGGAAGTGAAGATGGTGGCATGGTGGATAGGTATAGAGCAATCCTTTGGAGTACCTTACAATCCACAAAGCCAGGGAGTAGTAGAAGCAATGAATCACCACCTAAAAAATCAGATAAGTAGAATTAGAGAACAGGCAAATACAATAGAAACAATAGTACTAATGGCAGTTCATTGCATGAATTTTAAAAGAAGGGGAGGAATAGGGGATATGACCCCAGCAGAAAGACTAATTAACATGATCACCACAGAACAAGAAATACAATTCCTCCAAAGAAAAAATTCAAATTTTAAAAAATTCCAGGTCTATTACAGAGAAGGCAGAGATCAGCTGTGGAAAGGACCTGGAGAGCTACTGTGGAAGGGAGACGGAGCAGTCATAGTCAAGGTAGGGGCGGACATAAAAGTAGTACCAAGAAGGAAGGCCAAGATTATCAGGGACTATGGA

>X61240

TTCCTAGAAAAAATAGAACCAGCCCAGGAAGAGCATGAAAAATATCATGGCAATGTAAAAGAACTGGTCCATAAATTCGGAATTCCACAATTAGTGGCAAAACAGATAGTAAATTCCTGTGATAAATGCCAACAAAAAGGGGAAGCTATTCATGGACAGGTAAATGCAGACCTAGGGACATGGCAGATGGACTGTACACATTTAGAAGGAAAAATTATAATAGTGGCAGTCCATGTAGCCAGTGGGTTTATAGAAGCAGAGGTAATACCCCAAGAGACAGGAAGACAGACAGCTCTCTTCCTACTAAAGTTGGCCAGCAGATGGCCTATCACACACCTACACACAGACAACGGTGCCAACTTCACCTCACCAAGTGTAAAGATGGTAGCCTGGTGGGTAGGAATAGAACAAACTTTTGGAGTACCCTATAACCCACAAAGTCAAGGAGTAGTGGAAGCAATGAACCATCACCTGAAAAATCAAATAGACAGACTCAGAGACCAAGCAGTATCAATAGAGACAGTTGTACTAATGGCAACTCACTGCATGAATTTTAAAAGAAGGGGAGGAATAGGGGATATGACCCCTGCAGAAAGACTAGTTAACATGATAACCACAGAGCAAGAAATACAGTTCTTCCAAGCAAAAAATTTAAAATTTCAAAATTTCCAGGTCTATTACAGAGAAGGCAGAGATCAACTCTGGAAGGGACCTGGTGAACTATTGTGGAAAGGGGAAGGAGCAGTCATCATAAAGGTAGGGACAGAAATCAAAGTAGTACCCAGGAGAAAAGCAAAAATTATAAGGCACTATGGAGGAGGAAAA~~~GGATTGGATTGTAGTGCCGACATGGAGGAT

>U43096

TTTTTGGATGGAATAGATAAGGCCCAAGATGAGCATGAGAAATATCACAGTAATTGGAGAGCAATGGCTAGTGATTTTAACCTGCCACCTGTAGTAGCAAAAGAAATAGTAGCCAGTTGTGATAAATGTCAGCTAAAAGGAGAAGCCATGCATGGACAAGTAGACTGTAGTCCAGGAATATGGCAACTAGATTGTACACATTTGGAAGGAAAAGTTATCCTGGTAGCAGTTCATGTAGCCAGTGGATATATAGAAGCAGAAGTTATTCCAGCAGAGACAGGGCAGGAAACAGCATACTTTATCTTAAAATTAGCAGGAAGATGGCCAGTAAAAACAATACATACAGACAATGGCAGCAACTTCACCAGTACAACGGTTAAGGCCGCCTGTTGGTGGGCAGGGGTCAAGCAGGAATTTGGCATTCCCTACAATCCCCAAAGTCAAGGAGTAGTAGAATCTATGAATAAAGAATTAAAGAAAATTATAGGACAGGTAAGAGATCAAGCTGAACATCTTAAGACAGCAGTACAAATGGCAGTATTCATCCACAATTTTAAAAGAAAAGGGGGGATTGGGGGGTACAGTGCAGGGGAAAGAATAGTAGACATAATAGCAACAGACATACAAACTAAAGAATTACAAAAACAAATTACAAAAATTCAAAATTTTCGGGTTTATTACAGGGACAGCAGAGATCCACTTTGGAAAGGACCAGCAAAGCTCCTCTGGAAAGGTGAAGGGGCAGTAGTAATACAAGATAATAGTGAAATAAAAGTAGTGCCAAGAAGAAAAGCAAAGATCATTAGGGATTATGGAAAACAGATGGCAGGTGATGATTGTGTGGCAGGTAGACAGGATGAGGAT

>AF042102

TTTTTAGATGGAATAGATAGGGCACAAGAAGACCATGAGAAATATCACAGTAATTGGAGAGCAATGGCTAGTGATTTTAACCTGCCACCTATAGTAGCAAAAGAAATAGTAGCCAGCTGTGATAAATGTCAGCTGAAAGGAGAGGCCATGCATGGACAAGTAGACTGTAGTCCAGGAATATGGCAACTAGATTGTACACATCTAGAAGGAAAAATTATCCTGGTAGCAGTTCATGTAGCCAGTGGATATATAGAAGCAGAAGTTATTCCAGCAGAGACAGGGCAGGAAACAGCATACTTTATCTTAAAATTAGCAGGAAGATGGCCAGTAACAACAATACATACAGACAATGGCAGCAATTTCATCAGTACTACAGTTAAGGCCGCCTGTTGGTGGGCAGGGATCAAGCAGGAATTTGGCACTCCCTACAATCCCCAAAGCCAAGGAGTAGTAGAATCTATGAATAAAGAATTAAAGAAAATTATAGGACAGGTAAGAGATCAGGCTGAACATCTTAAGACAGCAGTACAAATGGCAGTATTCATCCACAATTTTAAAAGAAAAGGGGGGATTGGGGGATACAGTGCAGGGGAAAGAATAGTAGACATAATAGCATCAGACATACAAACTAAGGAATTACAAAAACAAATTACAAAAATTCAAAATTTTCGGGTTTATTACAGGGACAACAGAGATCCACTTTGGAAAGGACCAGCAAAACTTCTCTGGAAAGGCGAAGGGGCAGTAGTAATACAAGATAATAGTGACATAAAAGTAGTGCCAAGAAGAAAAGTAAAGATCATTAGGGATTATGGAAAACAGATGGCAGGTGATGATTGTGTGGCAAGTAGACAGGATGAGGAT

>AF042106

TTTTTAGATGGAATAGATAAGGCACAAGAAGACCATGAGAAATATCACAGTAATTGGAGAGCAATGGCTAGTGATTTTAACCTGCCACCTATAGTAGCAAAAGAAATAGTAGCCAGCTGTGATAAATGTCAGCTAAAAGGAGAAGCCATGCATGGACAAGTAGACTGTAGTCCAGGAATATGGCAACTAGATTGTACACATCTAGAAGGAAAAATTATCCTGGTAGCAGTTCATGTAGCCAGTGGATATATAGAAGCAGAAGTTATTCCAGCAGAGACAGGGCAGGAAACAGCATACTTTATCTTAAAATTAGCAGGAAGGTGGCCAGTAAACACAATACATACAGACAATGGCAGCAATTTCATCAGTACCACGGTTAAGGCCGCCTGTTGGTGGGCAGGGATCAAGCAGGAATTTGGCATTCCCTACAATCCCCAAAGCCAAGGAGTAGTGGAATCTATGAATAGAGAATTAAAGAAAATTATAGGACAGGTAAGAGATCAGGCTGAACATCTTAAGACAGCAGTACAAATGGCAGTATTCATCCACAATTTTAAAAGAAAAGGGGGGATTGGGGGATACAGTGCAGGGGAAAGAATAGTAGACATAATAGCAACAGACATACAAACTAAAGAATTACAAAAGCAAATTACAAAAATTCAAAATTTTCGGGTTTATTACAGGGACAGCAGAGATCCACTTTGGAAAGGACCAGCAAAACTTCTCTGGAAAGGCGAAGGGGCAGTAGTAATACAAGATAATAGTGACATAAAAGTAGTGCCAAGAAGAAAAGTAAAGATCATTAGGGATTATGGAAAACAGATGGCAGGTGATGATTGTGTGGCAAGTAGACAGGATGAGGAT

>U37270

TTTTTAGATGGAATAGATAAGGCACAAGAAGGCCATGAGAAATATCACAGTAATTGGAGAGCAATGGCTAGTGGTTTTAACCTGCCACCTATAGTAGCAAAAGAAATAGTAGCCAGCTGTGATAAATGTCAGCTAAAAGGAGAAGCCATGCATGGACAAGTAGACTGTAGTCCAGGAATATGGCAACTAGATTGTACACATCTAGAAGGAAAAATTATCCTGGTAGCAGTTCATGTAGCCAGTGGATATATAGAAGCAGAAGTTATTCCAGCAGAGACAGGGCAGGAAACAGCATACTTTATCTTAAAATTAGCAGGAAGGTGGCCAGTAAACACAATACATACAGACAATGGCGGCAATTTCATCAGTACCACGGTTAAGGCCGCCTGTTGGTGGGCAGGGATCAAGCAGGAATTTGGCATTCCCTACAATCCCCAAAGCCAAGGAGTAGTGGAATCTATGAATAGAGAATTAAAGAAAATTATAGGACAGGTAAGAGATCAGGCTGAACATCTTAAGACAGCAGTACAAATGGCAGTATTCATCCACAATTTTAAAAGAAAAGGGGGGATTGGGGGATACAGTGCAGGGGAAAGAATAGTAGACATAATAGCAACAGACATACAAACTAAAGAATTACAAAAGCAAATTACAAAAATTCAAAATTTTCGGGTTTATTACAGGGACAGCAGAGATCCACTTTGGAAAGGACCAGCAAAACTTCTCTGGAAAGGCGAAGGGGCAGTAGTAATACAAGATAATAGTGACATAAAAGTAGTGCCAAGAAGAAAAGTAAAGATCATTAGGGATTATGGAAAACAGATGGCAGGTGATGATTGTGTGGCAAGTAGACAGGATGAGGAT

>U27200

TTTCTAGAAAAAATAGAACCAGCTCAAGAAGAACATGAAAAATATCATAATAATGTAAAAGAACTAGTCCATAAATTTGGGATTCCACAATTAGTGGCAAGACAAATAGTAAATTCCTGTGATAAATGCCAACAAAAAGGGGAAGCTATTCATGGACAGGTAAATTCAGAACTAGGGACATGGCAAATGGACTGTACACATTTAGAGGGAAAGGTTATAATAGTGGCAGTTCATGTAGCCAGTGGATTCATAGAAGCAGAAGTAATACCCCAAGAAACAGGAAGACAGACAGCTCTCTTCCTGTTAAAGCTGGCCAGCAGATGGCCTATCACACACCTGCACACAGACAACGGTGCCAACTTCACTTCACAAGATGTGAAAATGGCAGCCTGGTGGATAGGGATAGAACAAACATTCGGAGTGCCCTATAATCCAGAAAGTCAGGGAGTAGTAGAAGCAATGAACCATCATCTGAAAAATCAGATAGACAGAATTAGAGATCAGGCAGTATCAATAGAGACAGTTGTGTTAATGGCAACTCACTGCATGAATTTTAAAAGAAGGGGAGGAATAGGGGATATGACCCCTGCAGAAAGAATAGTCAACATGATAACTACAGAACAAGAAATACAATTCCTCCAAACAAAAAATTTAAAATTCCAAAATTTCCGGGTCTATTACAGAGAAGGCAGAGATCAACTCTGGAAGGGACCTGGTGATCTATTGTGGAAAGGGGAAGGAGCAGTCATCATAAAGGTAGGGACAGAAATCAAAGTAATACCCAGAAGAAAAGCAAAGATCATAAGGAACTATGGA

>K03454

TTTTTGGATGGAATAGATAAGGCTCAAGAAGAACATGAGAAATATCACAACAATTGGAGAGCAATGGCTAGTGATTTTAACCTACCACCCGTGGTAGCAAAAGAAATAGTAGCTAGCTGTGATAAATGTCAGCTAAAAGGAGAAGCCATGCATGGACAAGTAGACTGTAGTCCAGGAATATGGCAATTAGATTGTACACACTTAGAAGGAAAAGTTATCCTGGTAGCAGTTCATGTAGCCAGTGGCTATATAGAAGCAGAAGTTATTCCAGCAGAAACAGGGCAGGAAACAGCATATTTTCTTTTAAAATTAGCAGGAAGATGGCCAGTAAAAGTAGTACATACAGACAATGGCAGCAATTTCACCAGTGCTGCAGTTAAGGCCGCCTGTTGGTGGGCAGGTATCAAACAGGAATTTGGAATTCCCTACAATCCCCAAAGTCAAGGAGTAGTAGAATCTATGAATAAAGAATTAAAGAAAATTATAGGACAGGTAAGAGATCAAGCTGAACATCTTAAGACAGCAGTACAAATGGCAGTATTCATCCACAATTTTAAAAGAAGAAGGGGGATTGGGGGATACAGTGCAGGGGAAAGAATAATAGACATAATAGCAACAGACATACAAACTAAAGAATTACAAAAACAAATTATAAAAATTCAAAATTTTCGGGTTTATTACAGAGACAGCAGAGATCCAATTTGGAAAGGACCAGCAAAGCTCCTCTGGAAAGGTGAAGGGGCAGTAGTAATACAAGACAAGAGTGACATAAAGGTAGTACCAAGAAGAAAAGTAAAGATTATTAGGGATTATGGAAAACAGATGGCAGGTGATGATTGTGTGGCAAGTAGACAGGATGAGGAT

>U51188

TTTTTAGATGGGATAGATAAGGCTCAAGAAGACCATGAAAGATATCACAGCAATTGGAGAGCAATGGCTAGTGACTTTAATTTGCCACCTATAGTAGCAAAGGAAATAGTAGCCAGCTGTGATAAATGTCAGCTAAAAGGGGAAGCCATGCATGGACAAGTAGACTGTAGTCCAGGGATATGGCAACTAGATTGCACGCATCTAGAAGGAAAAGTCATCCTGGTAGCAGTCCACGTGGCCAGTGGATATATAGAAGCAGAAGTTATCCCAGCAGAAACAGGACAGGAGACAGCATACTTTCTGCTAAAATTAGCAGGAAGATGGCCAGTAAGGGTAATACACACAGACAATGGCAGCAATTTCACCAGCGCTGCAGTTAAAGCAGCCTGTTGGTGGGCCAATGTCCAACAGGAATTTGGAATTCCCTACAATCCCCAAAGCCAAGGAGTAGTGGAATCTATGAATAAGGAATTAAAGAAAATCATAGGGCAGGTAAGAGAGCAAGCTGAACACCTTAAGACAGCAGTACAAATGGCAGTATTCATTCACAATTTTAAAAGAAAAGGGGGGATTGGGGAGTACAGTGCAGGGGAAAGAATAATAGACATAATAGCAACAGACATACAAACTAAAGCATTACAAAAACAAATTACAAAAATTCAAAATTTTCGGGTTTATTACAGGGACAGCAGAGATCCAATTTGGAAAGGACCAGCAAAACTACTCTGGAAAGGTGAAGGGGCAGTAGTAATACAGGACAATAGTGATATAAAAGTAGTACCAAGAAGAAAAGCAAAGATCATTAGGGATTATGGAAAACAGATGGCAGGTGATGATTGTGTGGCAGGTAGACAGAATGAGGAT

>U51189

TTTTTAGATGGGATAAATAAGGCTCAAGAAGAACATGAAAGATATCACAGCAATTGGAGAACAATGGCTAGTGACTTTAATTTGCCACCTATAGTAGCAAAGGAAATAGTAGCCAACTGTGATAAATGTCAACTAAAAGGGGAAGCTATGCATGGACAAGTAGACTGTAGTCCAGGGATATGGCAATTAGATTGCACACATCTAGAAGGAAAAGTCATCCTGGTAGCAGTCCACGTGGCCAGTGGATATATAGAAGCAGAAGTTATCCCAGCAGAAACAGGACAGGAGACAGCATACTTTCTGCTAAAATTAGCAGGAAGGTGGCCAGTAAAAGTAATACACACAGACAACGGTAGCAATTTCACCAGCGCTGCAGTTAAAGCAGCCTGTTGGTGGGCCAATGTCCGACAGGAATTTGGGATCCCCTACAATCCCCAAAGTCAAGGAGTAGTAGAATCAATGAATAAGGAATTAAAGAAAATCATAGGGCAGGTAAGGGAGCAAGCTGAACACCTTAAGACAGCAGTACAAATGGCAGTATTCATTCACAATTTTAAAAGAAAAGGGGGGATTGGGGGGTACAGTGCAGGGGAAAGAATAATAGACATAATAGCAACAGACATACAAACTAAAGAATTACAAAAACAAATTACAAAAATTCAAAATTTTCGGGTTTATTACAGGGACAGCAGAGACCCAATTTGGAAAGGACCAGCAAAACTACTCTGGAAAGGTGAAGGGGCAGTAGTAATACAAGACAATAGTGATATAAAAGTAGTACCAAGAAGAAAAGCAAAGATCATTAGGGATTATGGAAAACAGATGGCAGGTGATGATTGTGTGGCAGGTAGACAGGATGAGGAT

>M30895

TTCCTAGAAAGGATAGAGCCCGCCCAAGAAGAACATGAAAAATATCATAGCAATATGAAAGAACTAACCCATAAATTTGGAATACCCCAACTAGTAGCAAGACAGATAGTAAACACATGTGCCCAATGCCAACAGAAAGGAGAGGCCATACATGGGCAGGTAAATGCAGAAATAGGTGTCTGGCAAATGGACTGCACACACTTAGAAGGAAAGATCATTATAGTAGCAGTACATGTTGCAAGTGGATTCATAGAAGCAGAAGTTATCCCACAGGAATCAGGAAGGCAGACAGCGCTCTTCCTATTAAAACTGGCCAGTAGGTGGCCAATAACACACTTACACACAGACAATGGCTCCAACTTCACTTCACAGGAAGTGAAGATGGTGGCATGGTGGATAGGTATAGAGCAATCCTTTGGAGTACCTTACAACCCACAAAGCCAGGGAGTAGTAGAAGCAATGAATCACCACTTAAAGAATCAGATAAGTAGAATTAGAGAACAGGCAAATACAATAGAAACCATAGTACTAATGGCAGTTCATTGCATGAATTTTAAAAGAAGGGGAGGAATAGGGGATATGACCCCAGCAGAAAGACTAATCAACATGATTACCACAGAACAAGAAATACAATTCCTCCAAAGAAAAAATTCAAATTTTAAAAACTTCCAGGTCTATTACAGAGAAGGCAGAGATCAGCTGTGGAAAGGACCTGGAGAACTACTGTGGAAGGGAGACGGAGCAGTCATAGTCAAGGTAGGGGCAGACATAAAAGTAATACCAAGAAGGAAGGCCAAGATTATCAGAGACTATGGAAGGCAAGAACTGGATAGTTCCCACCTGGAGGGTGCCAGGGAGGAGGAT

>U43141

TTTTTAGATGGAATAGATAAGGCCCAAGAAGACCATGAGAAATATCACAGTAATTGGAGAGCAATGGCTAATGATTTTAACCTGCCACCTGTAGTAGCAAAAGAAATAGTAGCCAGCTGTGATAAATGTCAGCTAAAAGGAGAAGCCATGCATGGACAAGTAGACTGTAGTCCAGGAATATGGCAACTAGATTGTACACATTTAGAAGGAAAAATTATCCTGGTAGCAGTTCATGTAGCTAGTGGATATATAGAAGCAGAAGTCATTCCAGTAGAGACAGGGCAGGAAACAGCATATTTTCTCTTAAAATTAGCAGGAAGATGGCCAGTAAAAACAGTACATACAGACAATGGCCCTAATTTCACCAGTACTACGGTTAAGGCCGCCTGTTGGTGGGCAGGGATCAAGCAGGAATTTGGCATTCCCTACAATCCCCAAAGTCAAGGGGTAGTAGAATCTATGAATAAAGAGTTAAAGAAAATTATAGGACAGGTAAGAGATCAGGCTGAACATCTTAAGACAGCAGTACAAATGGCAGTATTCATCCACAATTTTAAAAGAAAAGGGGGGATTGGGGGGTACAGTGCAGGGGAAAGAATAGTAGACATAATAGCAACAGACATACAAACTAAAGAATTACAAAAACAAATTACAAAAATTCAAAATTTTCGGGTTTATTACAGGGACAGCAGAGAACCATTTTGGAAAGGACCAGCAAAGCTTCTCTGGAAAGGTGAAGGGGCAGTAGTAATACAAGATAATAGTGACATAAAAGTAGTGCCAAGAAGAAAAGCAAAGATCATTAGGGATTATGGAAAACAGATGGGCAGTGATGATTGTGTGGCAAGTAGACAGGATGAGGAT

>L39106

TTTTTAGATGGCATAGATAAAGCCCAAGAAGAGCATGAAAGATATCACAGCAATTGGAAGGCAATGGCTAGTGATTTTAATCTGCCACCTATAGTAGCAAAAGAAATAGTGGCCAGCTGTGATAAATGTCAGATGAAAGGGGAAGCCATGCATGGACAAGTAGACTGTGGTCCAGGAATATGGCAATTAGATTGTACACATTTAGAAGGGAAAATTATCTTAGTAGCAGTCCATGTAGCCAGTGGCTATATAGAAGCAGAAGTTATCCCAGCAGAAACAGGACAGGAGACAGCATACTTTATATTAAAATTAGCAGGAAGATGGCCAGTGAAAGTAATACACACAGACAATGGCAGCAATTTCACCAGTGCTGCAGTAAAGGCGGCATGTTGGTGGGCAAATGTCACACAAGAATTTGGAATTCCCTACAATCCCCAAAGCCAAGGAGTAGTGGAATCTATGAATAAAGAATTAAAGAAAATTATAGGACAGGTCAGAGATCAAGCTGAACACCTTAAGACAGCAGTACAGATGGCAGTATTCATTCACAATTTTAAAAGAAAAGGGGGGATTGGGGGGTACAGTGCAGGGGAAAGAATAATAGATATAATAGCATCAGATATACAAACTAAAGAACTACAAAAACAGATTATAAAAATTCAAAATTTTCGGGTTTATTACAGGGACAGCAGAGACCCCATTTGGAAAGGACCAGCAAAACTACTCTGGAAAGGTGAAGGGGCAGTAGTAATACAGGACAATAGTGATATAAAGGTAGTACCAAGAAGAAAAGCAAAAATCATTAGGGATTATGGAAAACAGATGGCAGGTGATGATTGTGTGGCAGGTAGACAGGATGAGGAT

>J04498

TTCCTAGAGAGAATAGAGCCCGCTCAGGAAGAACATGGAAAATATCATAGCAATGTAAAAGAACTAGCCCATAAGTTTGGATTACCCAACCTGGTGGCAAGACAAATAGTAAACACATGTGCCCAGTGCCAACAAAAAGGGGAAGCTATACATGGGCAAGTAAATGCAGAACTAGGCACCTGGCAAATGGACTGCACACACTTAGAAGGAAAAATCATTATAGTAGCAGTACATGTTGCAAGTGGATTTATAGAAGCAGAAGTCATCCCACAGGAATCAGGAAGGCAAACAGCACTCTTCCTATTAAAACTGGCCAGTAGGTGGCCAATAACACACTTGCACACAGATAATGGTGCCAACTTCACTTCACAGGAGGTAAAGATGGTAGCATGGTGGGTAGGCATAGAACAATCCTTTGGAGTACCTTACAATCCACAAAGCCAGGGAGTAGTAGAAGCAATGAATCACCACCTGAAAAATCAGATAGAAAGAATTAGAGAGCAGGCAAATACAATGGAAACAATAGTACTAATGGCAGTTCATTGCATGAATTTTAAAAGAAGGGGAGGAATAGGGGATATGACCCCAGTAGAAAGACTAGTCAATATGATCACCACAGAACAAGAAATACAATTCCTCCAAGCAAAAAATTCAAAATTAAAAAATTTTCGGGTCTATTTCAGAGAAGGCAGAAATCAACTGTGGCAAGGACCTGGGGAGCTACTGTGGAAAGGGGACGGAGCAGTCATAGTCAAGGTAGGGACAGATATAAAAGTAATACCAAGAAGAAAGGCCAAGATCATCAGAGACTATGGACCAAGGCAAGAGATGGAT

>M38429

TTTTTAGATGGAATAGATAAGGCCCAAGAAGATCATGAAAAATATCACAGTAATTGGAGAGCAATGGCTAGTGATTTTAACCTGCCACCTATAGTAGCAAAAGAAATAGTAGCCAGCTGTGATAAATGTCAGCTAAAAGGAGAAGCCATGCATGGACAAGTAGACTGTAGTCCAGGAATATGGCAACTAGATTGTACACATTTAGAAGGAAAAATTATCCTGGTAGCAGTTCATGTAGCCAGTGGATATATAGAAGCAGAAGTTATTCCAGCAGAAACAGGGCAGGAAACAGCATACTTTCTCTTAAAATTAGCAGGCAGATGGCCAGTAACAACAATACATACAGACAATGGCAGCAATTTCACCAGTACTACAGTTAAGGCCGCCTGTTGGTGGGCTGGGATCAAGCAGGAATTTGGCATTCCCTACAATCCCCAAAGTCAAGGAGTAGTAGAATCTATGAATAAAGAATTAAAGAAAATTATAGGACAGGTAAGAGATCAGGCTGAACATCTTAAGACAGCAGTACAAATGGCAGTATTCATCCACAATTTTAAAAGAAAAGGGGGGATTGGGGGGTACAGTGCAGGGGAAAGAATAATAGACATAATAGCAACAGACATACAAACTAAAGAATTACAAAAACAAATTACAAAAATTCAAAATTTTCGGGTTTATTACAGGGACAACAGAGATCCAATTTGGAAAGGACCAGCAAAGCTTCTCTGGAAAGGTGAAGGGGCAGTAGTAATACAAGATAATAGTGACATAAAAGTAGTGCCAAGAAGAAAAGTAAAAATCATTAGGGATTATGGAAAACAGATGGCAGGTGATGATTGTGTGGCAAGTAGACAGGATGAGGAT

>U63632

TTTTTAGATGGAATAGATAAGGCCCAAGAAGATCATGAGAAATATCACAGTAATTGGAAAGCAATGGCTAGTGATTTTAACCTGCCACCTGTAGTAGCAAAAGAAATAGTAGCCAGCTGTGATAAATGTCAGCTAAAAGGAGAAGCCATGCATGGACAAGTAGATTGTAGTCCAGGAATATGGCAACTAGATTGTACACATTTAGAAGGAAAAATTATCCTGGTAGCAGTTCATGTAGCCAGTGGATATATAGAAGCAGAAGTTATTCCAGCAGAAACAGGGCAGGAAACAGCATACTTTCTCTTAAAATTAGCAGGAAGATGGCCAGTAAAAACAATACATACAGACAATGGCAGCAATTTCACCAGTACTACGGTTAAGGCCGCCTGTTGGTGGGCTGGGATCAAGCAGGAATTTGGCATTCCCTACAATCCCCAAAGTCAAGGAGTAGTAGAATCTATGAATAAAGAATTAAAGAAAATTATAGGACAGGTAAGAGATCAGGCTGAACATCTTAAGACAGCAGTACAAATGGCAGTATTCATCCACAATTTTAAAAGAAAAGGGGGGATTGGGGGGTACAGTGCAGGGGAAAGAATAGTAGACATAATAGCAACAGACATACAAACTAAAGAATTACAAAAACAAATTACAAAAATTCAAAATTTTCGGGTTTATTACAGGGACAGCAGAGAGCCACTTTGGAAAGGACCAGCAAAGCTTCTCTGGAAAGGTGAAGGGGCAGTAGTAATACAAGATAATAGTGATATAAAAGTAGTGCCAAGAAGAAAAGTAAAGATCATTAGGGATTATGGAAAACAGATGGCAGGTGATGATTGTGTGGCAAGTAGACAGGATGAGGAT

>U22047

TTCCTAGAAAAAATAGAGCCCGCTCAGGAAGAACATGAGAAATATCATAGCAATGTAAAAGAATTATCCCATAAATTTGGACTGCCCAAACTAGTGGCAAGACAAATAGTAAACACATGTGCCCAATGTCAACAGAAAGGGGAAGCTATACATGGGCAAGTAGATGCAGAACTGGGCACTTGGCAAATGGACTGCACACACTTAGAGGGAAAAATCATTATAGTAGCAGTACATGTTGCAAGCGGGTTTATAGAAGCAGAAGTTATCCCACAGGAAACGGGAAGGCAAACAGCACTCTTCCTATTAAAACTGGCCAGTAGGTGGCCAATAACACACCTGCACACAGATAATGGTGCCAACTTCACCTCACAGGAAGTAAAGATGGTAGCGTGGTGGACAGGTATAGAACAATCCTTTGGAGTACCTTACAATCCACAAAGCCAAGGAGTAGTAGAAGCAATGAATCACCACTTAAAAAACCAGATAAGCAGAATTAGAGAGCAGGCAAATACAATGGAAACAATAGTATTAATGGCAGTTCATTGCATGAATTTTAAAAGAAGGGGAGGAATAGGGGATATGACCCCAGCAGAAAGACTAATCAATATGATCACCACAGAACAAGAAATACAATTCCTCCACGCAAAAAATTCAAAATTAAAAAATTTCCGGGTCTATTTCAGAGAAGGCAGAGATCAGCTGTGGAAAGGACCTGGGGAACTACTGTGGAAGGGAGATGGAGCAGTCATAGTCAAGGTAGGGACAGACATAAAAATAGTGCCAAGAAGGAAAGCTAAGATCATCAGAGACTATGGA

>M27470

TTCCTACAAAATATAGAACCAGCACAGGAAGAACATGAGAAATATCATAGCAATGAAGCACAATTAAGAGAGAAATTCCACTTACCAGCTCTAGTAGCCAAACAGATTGTGCAAAGTTGCAGTAAGTGCTGTCATCATGGAGAGCCCATAAAGGGACAGACAGATGCTTCACTTGGAGTCTGGCAGATAGATTGCACACATCTGGAAAATCAAATTATTATAGTAGCAGTGCATGTAGCTTCAGGCTTCATGAAGGCAGAAGTTATAACAGCAGAAACTGGAAAAAAGACAGCAGAGTTTCTGTTAAAGTTAGCAGCACAATGGCCTATTAGTAAACTACACACAGATAATGGGCCTAACTTTACTAGTCAGGAAGTAGAAACCATGTGTTGGTGGTTAGGGATAGAACACACATTTGGAATACCCTATAACCCACAAAGTCAGGGGGTAGTGGAAAATAAAAATAAGTATCTAAAAGAATTGATTGAGAAAATAAGAGAAGATTGCAAAGAATTAAAAACAGCAGTAGCCATGGCCACATTCATTCATAATTTTAAACAAAGGGGAGGACTAGGGGGGATGACAGCAGGAGAGAGAATAGTAAATATGATCAATACAGAATTAGAATATCAATATCAACAAAATCAAATTTCAAAAAATTTAAATTTTAAGGTTTACTTCAGAGAAGGAAGAGATCAGCTGTGGAAAGGACCTGGTATCCTTTTGTGGAAAGGAGAAGGGGCAGTAGTTTTAAAATATCAAGAAGAGATAAAGATAGTACCTAGAAGAAAGTGTAAAATAATAAAAGATTATGGAGAG

>Z48731

TTCCTAGAAAAAATAGAGCCCGCTCAAGAAGAACATGAAAAATATCATAGCAATGTAAAAGAACTATCCCATAAATTTGGATTACCCCAACTAGTGGCAAGACAAATAGTAAACACATGTGCCCAATGTCAACAGAAAGGAGAGGCTATACATGGGCAAGTAAATGCAGACTTAGGCACTTGGCAAATGGACTGCACTCATTTAGAAGGAAAAGTCATTATAGTAGCAGTACATGTTGCAAGTGGATTTATAGAGGCAGAAGTCATCCCACAGGAAACAGGAAGGCAGACAGCACTCTTCCTGTTAAAACTGGCTAGTAGGTGGCCAATAACACACTTGCACACAGATAATGGTGCCAACTTCACTTCACAGGAAGTGAAGATGGTAGCATGGTGGATAGGTATAGAACAATCCTTTGGAGTACCTTACAATCCACAAAGCCAAGGAGTAGTAGAAGCCATGAATCACCACCTAAAAAATCAGATAAGTAGAATCAGAGATCAGGCAAATACAGTAGAAACAATAGTACTAATGGCAGTTCATTGCATGAATTTTAAAAGAAGGGGAGGAATAGGGGATATGACCCCATCAGAAAGACTAATCAATATGATCACCACAGAACAAGAAATACAATTCCTCCAAGCCAAAAATTCAAAATTAAAAAATTTTCGGGTCTATTTCAGAGAAGGCAGAGATCAGTTGTGGAAAGGACCTGGGGAGCTACTGTGGAAGGGAGACGGAGCAGTCATAGTCAAGGTAGGAACAGACATAAAAATAGTACCAAGAAGGAAGGCCAAGATCATCAGAGACTATGGA

>M33262

TTCTTGGAAAAGATAGAGCCAGCACAAGAAGAACATGATAAATACCATAGTAATGTAAAAGAATTGGTATTCAAATTTGGATTACCCAGAATAGTGGCCAGACAGATAGTAGACACCTGTGATAAATGTCATCAGAAAGGAGAGGCTATACATGGGCAGGCAAATTCAGATCTAGGGACTTGGCAAATGGATTGTACCCATCTAGAGGGAAAAATAATCATAGTTGCAGTACATGTAGCTAGTGGATTCATAGAAGCAGAGGTAATTCCACAAGAGACAGGAAGACAGACAGCACTATTTCTGTTAAAATTGGCAGGCAGATGGCCTATTACACATCTACACACAGATAATGGTGCTAACTTTGCTTCGCAAGAAGTAAAGATGGTTGCATGGTGGGCAGGGATAGAGCACACCTTTGGGGTACCATACAATCCACAGAGTCAGGGAGTAGTGGAAGCAATGAATCACCACCTGAAAAATCAAATAGATAGAATCAGGGAACAAGCAAATTCAGTAGAAACCATAGTATTAATGGCAGTTCATTGCATGAATTTTAAAAGAAGGGGAGGAATAGGGGATATGACTCCAGCAGAAAGATTAATTAACATGATCACTACAGAACAAGAGATACAATTTCAACAATCAAAAAACTCAAAATTTAAAAATTTTCGGGTCTATTACAGAGAAGGCAGAGATCAACTGTGGAAGGGACCCGGTGAGCTATTGTGGAAAGGGGAAGGAGCAGTCATCTTAAAGGTAGGGACAGACATTAAGGTAGTACCCAGAAGAAAGGCTAAAATTATCAAAGATTATGGA

>M19499

TTCTTGGAAAAGATAGAGCCAGCACAAGAAGAACATGATAAATACCATAGTAATGTAAAAGAATTGGTATTCAAATTTGGATTACCCAGAATAGTGGCCAGACAGATAGTAGACACCTGTGATAAATGTCATCAGAAAGGAGAAGCTATACATGGGCAGGTAAATTCAGATCTAGGGACTTGGCAAATGGACTGTACCCATCTAGAAGGAAAAATAGTCATAGTTGCAGTACATGTAGCTAGTGGATTCATAGAAGCAGAAGTAATTCCACAAGAGACAGGAAGACAGACAGCACTATTTCTGTTAAAATTGGCAGGCAGATGGCCTATTACACATCTACACACAGATAATGGTGCTAACTTTGCCTCGCAAGAAGTAAAGATGGTTGCATGGTGGGCAGGGATAGAGCACACCTTTGGGGTACCATACAATCCACAGAGTCAGGGAGTAGTGGAAGCAATGAATCACCACCTGAAAAATCAAATAGATAGAATCAGGGAACAAGCAAATTCAGTAGAAACCATAGTATTAATGGCAGTTCATTGCATGAATTTTAAAAGAAGGGGAGGAATAGGGGATATGACTCCAGCAGAAAGATTAATTAACATGATCACTACAGAACAAGAAATACAATTTCAACAATCAAAAAACTCAAAATTTAAAAATTTTCGGGTCTATTACAGAGAAGGCAGAGATCAACTGTGGAAGGGACCCGGTGAGCTATTGTGGAAAGGGGAAGGAGCAGTCATCTTAAAGGTAGGGACAGACATTAAGGTAGTACCCAGAAGAAAGGCTAAAATTATCAAAGATTATGGA

>Y00277

TTCTTGGAAAAGATAGAGCCAGCACAAGAAGAACATAGTAAATACCATAGTAACATAAAAGAATTGGTATTCAAATTTGGATTACCCAGACTAGTGGCCAAACAGATAGTAGACACATGTGATAAATGTCATCAAAAAGGAGAAGCTATACATGGGCAGGTAAATTCAGACCTAGGGACTTGGCAAATGGATTGTACCCATCTAGAGGGAAAAATAGTCATAGTTGCAGTACATGTAGCTAGTGGATTCATAGAAGCAGAAGTAATTCCACAAGAAACAGGAAGACAGACAGCACTATTTCTGTTAAAATTGGCAAGCAGATGGCCTATTACACATCTGCACACAGATAATGGTGCTAACTTTGCTTCGCAAGAAGTAAAGATGGTTGCATGGTGGGCAGGGATAGAGCACACCTTTGGGGTACCATACAATCCACAGAGTCAGGGAGTAGTGGAAGCAATGAATCACCACCTGAAAAATCAAATAGATAGAATCAGGGAACAAGCAAATTCAGTAGAAACCATAGTATTAATGGCAGTTCATTGCATGAATTTTAAAAGAAGGGGAGGAATAGGGGATATGACTCCAGCAGAAAGATTAATTAACATGATCACTACAGAACAAGAAATACAATTTCAACAATCAAAAAACTCAAAATTTAAAAATTTTCGGGTCTATTACAGAGAAGGCAGAGATCAGCTGTGGAAGGGACCCGGTGAGCTATTGTGGAAAGGGGAAGGAGCAGTCATCTTAAAGGTAGGAACAGACATTAAGGTAGTACCCAGGAGAAAGGCTAAAATTATCAAAGATTATGGA

>L20571

TTCCTGGAAGGAATAGATCAGGCACAAGAAGATCATGAAAAATATCATAGTAATTGGAGAGCATTAGCTAGTGACTTTGGATTACCACCAATAGTAGCCAAGGAAATCATTGCTAGTTGTCCTAAATGCCATATAAAAGGGGAAGCAACGCATGGTCAAGTAGACTACAGCCCAGAGATATGGCAAATGGATTGTACACATTTAGAAGGCAAAATCATAATAGTTGCTGTCCATGTAGCAAGTGACTTTATAGAAGCAGAGGTGATACCAGCAGAAACAGGACAGGAAACTGCCTATTTCCTGTTAAAATTAGCAGCAAGATGGCCTGTCAAAGTAATACATACAGACAATGGACCTAATTTTACAAGTGCAGCCATGAAAGCTGCATGTTGGTGGACAGGCATACAACATGAGTTTGGGATACCATATAATCCACAAAGTCAAGGAGTAGTAGAAGCCATGAATAAAGAATTAAAATCTATTATACAGCAGGTGAGGGACCAAGCAGAGCATTTAAAAACAGCAGTACAAATGGCAGTCTTTGTTCACAATTTTAAAAGAAAAGGGGGGATTGGGGGGTACACTGCAGGGGAGAGACTAATAGACATACTAGCATCACAAATACAAACAACAGAACTACAAAAACAAATTTTAAAAATCAACAATTTTCGGGTCTATTACAGAGATAGCAGAGACCCTATTTGGAAAGGACCGGCACAACTCCTGTGGAAAGGTGAGGGGGCAGTAGTCATACAAGATAAAGGAGACATTAAAGTGGTACCAAGAAGAAAGGCAAAAATAATCAGAGATTATGGAAAACAGATGGCAGGTACTGATAGTATGGCAAATAGACAGACAGAA

>M27323

TTTTTGGATGGAATAGATAAGGCTCAGGAAGAACATGAGAAATATCACAACAATTGGAGAGCAATGGCTAGTGATTTTAACCTACCACCTGTGGTAGCGAAAGAAATAGTAGCTAGCTGTGATAAATGTCAGCTAAAAGGAGAAGCCATGCATGGACAAGTAGACTGTAGTCCAGGAATATGGCAATTAGATTGTACACATCTGGAAGGAAAAGTTATCCTGGTAGCAGTTCATGTAGCCAGTGGCTATATAGAAGCAGAAGTTATTCCAGCAGAAACGGGGCAAGAAACAGCATACTTTCTCTTAAAATTAGCAGGAAGATGGCCAGTAAAAGTAGTACATACAGATAATGGCAGCAATTTCACCAGTGCTACAGTTAAGGCCGCCTGTTGGTGGGCAGGGATCAAACAGGAATTTGGAATTCCCTACAATCCCCAAAGTCAAGGAGTAGTAGAATCTATGAATAAAGAATTAAAGAAAATTATAGGACAGGTAAGAGATCAAGCTGAACATCTTAAGACAGCAGTACAAATGGCAGTATTTATCCACAATTTTAAAAGAAAAGGGGGGATTGGGGGATACAGTGCAGGGGAAAGAATAATAGACATAATAGCAACAGACATACAAACTAGAGAATTACAAAAACAAATCATAAAAATTCAAAATTTTCGGGTTTATTACAGGGACAGCAGAGATCCAATTTGGAAAGGACCAGCAAAGCTTCTCTGGAAAGGTGAAGGGGCAGTAGTAATACAAGACAATAGTGACATAAAGGTAGTACCAAGAAGAAAAGTAAAGATCATTAGGGATTATGGAAAACAGATGGCAGGTGATGATTGTGTGGCAAGTAGACAGGATGAGGAT

>J03654

TTCCTAGAGAAAATAGAACCCGCGCAGGAAGAACATGAAAAATATCATAGCAATATAAAAGAACTGTCCCATAAATTTGGAATACCCAAGCTAGTGGCAAGACAAATAGTAAACACATGTGCCCATGTGCAACAGAAAGGGGAGGCTATACATGGGCAAGTAAATGCAGAACTAGGCACTTGGCAAATGGACTGCACACATTTAGAAGGAAAAGTCATTATAGTAGCAGTACATGTTGCAAGTGGATTTATAGAGGCAGAAGTTATCCCACAGGAATCAGGAAGGCAAACAGCACTGTTCTTACTGAAACTGGCCAGTAGGTGGCCAATAACACACCTGCACACAGATAATGGTGCCAACTTCACTTCACAGGAAGTGAAAATGGTAGCATGGTGGGTAGGTATAGAACAAACCTTTGGAGTGCCTTACAATCCACAAAGCCAAGGAGTAGTAGAAGCAATGAATCATCATCTAAAAAATCAGATAGACAGAATTAGAGAGCAGGCAAATACAGTAGAAACAATAGTATTAATGGCAGTTCATTGCATGAATTTTAAAAGAAGGGGAGGAATAGGGGATATGACCCCAGCAGAAAGAATAATCAATATGATCACCACAGAACAAGAAATACAATTCCTCCAAGCAAAAAATTCAAAATTAAAAAATTTTCGGGTCTATTTCAGAGAAGGCAGAGATCAGCTGTGGAAAGGACCTGGGGAACTGCTGTGGAAGGGAGACGGAGCAGTCATAGTCAAGGTAGGGACAGAAATAAAAGTAGTACCAAGAAGGAAAGCCAAGATCATTAAAGACTATGGAGGAAGGCAAGAGATGGAT

>M19921

TTTTTAGATGGAATAGATAAGGCCCAAGAAGAACATGAGAAATATCACAGTAATTGGAGAGCAATGGCTAGTGATTTTAACCTACCACCTGTAGTAGCAAAAGAAATAGTAGCCAGCTGTGATAAATGTCAGCTAAAAGGGGAAGCCATGCATGGACAAGTAGACTGTAGCCCAGGAATATGGCAGCTAGATTGTACACATTTAGAAGGAAAAGTTATCTTGGTAGCAGTTCATGTAGCCAGTGGATATATAGAAGCAGAAGTAATTCCAGCAGAGACAGGGCAAGAAACAGCATACTTCCTCTTAAAATTAGCAGGAAGATGGCCAGTAAAAACAGTACATACAGACAATGGCAGCAATTTCACCAGTACTACAGTTAAGGCCGCCTGTTGGTGGGCGGGGATCAAGCAGGAATTTGGCATTCCCTACAATCCCCAAAGTCAAGGAGTAATAGAATCTATGAATAAAGAATTAAAGAAAATTATAGGACAGGTAAGAGATCAGGCTGAACATCTTAAGACAGCAGTACAAATGGCAGTATTCATCCACAATTTTAAAAGAAAAGGGGGGATTGGGGGGTACAGTGCAGGGGAAAGAATAGTAGACATAATAGCAACAGACATACAAACTAAAGAATTACAAAAACAAATTACAAAAATTCAAAATTTTCGGGTTTATTACAGGGACAGCAGAGATCCAGTTTGGAAAGGACCAGCAAAGCTCCTCTGGAAAGGTGAAGGGGCAGTAGTAATACAAGATAATAGTGACATAAAAGTAGTGCCAAGAAGAAAAGCAAAGATCATCAGGGATTATGGAAAACAGATGGCAGGTGATGATTGTGTGGCAAGTAGACAGGATGAGGAT

>M38431

TTTTTAGATGGAATAGATAAGGCCCAAGAAGAACATGAGAAATATCACAGTAATTGGAGAGCAATGGCTAGTGATTTTAACCTACCACCTGTAGTAGCAAAAGAAATAGTAGCCAGCTGTGATAAATGTCAGCTAAAAGGAGAAGCCATGCATGGACAAGTAGACTGTAGCCCCGGAATATGGCAGCTAGATTGTACACATTTAGAAGGAAAAGTTATCTTGGTAGCAGTTCATGTGGCCAGTGGATATATAGAAGCAGAAGTAATTCCAGCAGAGACAGGGCAAGAAACAGCATACTTCCTCTTAAAATTAGCAGGAAGATGGCCAGTAAAAACAGTACATACAGACAATGGCAGCAATTTCACCAGTACTACAGTTAAAGCCGCCTGTTGGTGGGCGGGGATCAAGCAGGAATTTGGCATTCCCTACAATCCCCAAAGTCAAGGAGTAATAGAATCTATGAATAAAGAATTAAAGAAAATTATAGGACAGGTAAGAGATCAGGCTGAACATCTTAAGACAGCAGTACAAATGGCAGTATTCATCCACAATTTTAAAAGAAAAGGGGGGATTGGGGGGTACAGTGCAGGGGAAAGAATAGTAGACATAATAGCAACAGACATACAAATTAAAGAATTACAAAAACAAATTACAAAAATTCAAAATTTTCGGGTTTATTACAGGGACAGCAGAGATCCAGTTTGGAAAGGACCAGCAAAGCTCCTCTGGAAAGGTGAAGGGGCAGTAGTAATACAAGATAATAGTGACATAAAAGTAGTGCCAAGAAGAAAAGCAAAGATCATCAGGGATTATGGAAAACAGATGGCAGGTGATGATTGTGTGGCAAGTAGACAGGATGAGGAT

>M26727

TTTTTGGATGGAATAGATAAGGCCCAAGAGGAACATGAGAAATATCACAGTAACTGGAGAGCAATGGCTAGTGATTTTAACCTGCCACCTGTAGTAGCAAAAGAAATAGTAGCCAGCTGTGATAAATGCCAGCTAAAAGGAGAAGCCATGCATGGACAAGTAGACTGTAGTCCAGGAATATGGCAACTAGATTGTACACATTTAGAAGGAAAAATTATCCTGGTAGCAGTTCATGTAGCCAGTGGATATATAGAAGCAGAAGTTATTCCAGCAGAGACAGGGCAGGAAACAGCATACTTTATCTTAAAATTAGCAGGAAGATGGCCAGTAAAAACAATACATACAGACAATGGCAGCAATTTCACCAGTACTACGGTTAAGGCCGCCTGTTGGTGGGCAGGGATCAAGCAGGAATTTGGCATTCCCTACAATCCCCAAAGTCAAGGAGTAGTAGAATCTATGAATAATGAATTAAAGAAAATTATAGGACAAGTAAGAGATCAGGCTGAACATCTTAAGACAGCAGTACAAATGGCAGTATTTATCCACAATTTTAAAAGAAAAGGGGGGATTGGGGGGTACAGTGCAGGGGAAAGAATAGTAGATATAATAGCTACAGACATACAAACTAAAGAACTACAAAAACAAATTACAAAAATTCAAAATTTTCGGGTTTATTACAGGGACAGCAGAGAACCACTTTGGAAAGGACCAGCAAAGCTTCTTTGGAAAGGTGAAGGGGCAGTAGTAATACAAGATAATAGTGACATAAAAGTAGTGCCAAGAAGGAAAGCAAAGATCATTAGGGATTATGGAAAACAGATGGCAGGTGATGATTGTGTGGCAAGTAGACAGGATGAGGAT

>M15390

TTCCTGGAAAAAATAGAGCCCGCTCAGGAAGAACATGAAAAATATCATAGCAATGTAAAAGAACTGTCTCATAAATTTGGAATACCCAATTTAGTGGCAAGGCAAATAGTAAACTCATGTGCCCAATGTCAACAGAAAGGGGAAGCTATACATGGGCAAGTAAATGCAGAACTAGGCACTTGGCAAATGGACTGCACACATTTAGAAGGAAAGATCATTATAGTAGCAGTACATGTTGCAAGTGGATTTATAGAAGCAGAAGTCATCCCACAGGAATCAGGAAGACAAACAGCACTCTTCCTATTGAAACTGGCAAGTAGGTGGCCAATAACACACTTGCATACAGATAATGGTGCCAACTTCACTTCACAGGAGGTGAAGATGGTAGCATGGTGGATAGGTATAGAACAATCCTTTGGAGTACCTTACAATCCACAGAGCCAAGGAGTAGTAGAAGCAATGAATCACCATCTAAAAAACCAAATAAGTAGAATCAGAGAACAGGCAAATACAATAGAAACAATAGTACTAATGGCAATTCATTGCATGAATTTTAAAAGAAGGGGGGGAATAGGGGATATGACTCCATCAGAAAGATTAATCAATATGATCACCACAGAACAAGAGATACAATTCCTCCAAGCCAAAAATTCAAAATTAAAAGATTTTCGGGTCTATTTCAGAGAAGGCAGAGATCAGTTGTGGAAAGGACCTGGGGAACTACTGTGGAAAGGAGAAGGAGCAGTCCTAGTCAAGGTAGGAACAGACATAAAAATAATACCAAGAAGGAAAGCCAAGATCATCAGAGACTATGGAGGAAGACAAGAGATGGATAGTGCAGTAGCAGGTAGA

>X05291

TTCCTGGAAAAAATAGAGCCCGCTCAGGAAGAACATGAAAAATATCATAGCAATGTAAAAGAACTGTCTCATAAATTTGGAATACCCAATTTAGTGGCAAGGCAAATAGTAAACTCATGTGCCCAATGTCAACAGAAAGGGGAAGCTATACATGGGCAAGTAAATGCAGAACTAGGCACTTGGCAAATGGACTGCACACATTTAGAAGGAAAGATCATTATAGTAGCAGTACATGTTGCAAGTGGATTTATAGAAGCAGAAGTCATCCCACAGGAATCAGGAAGACAAACAGCACTCTTCCTATTGAAACTGGCAAGTAGGTGGCCAATAACACACTTGCATACAGATAATGGTGCCAACTTCACTTCACAGGAGGTGAAGATGGTAGCATGGTGGATAGGTATAGAACAATCCTTTGGAGTACCTTACAATCCACAGAGCCAAGGAGTAGTAGAAGCAATGAATCACCATCTAAAAAACCAAATAAGTAGAATCAGAGAACAGGCAAATACAATAGAAACAATAGTACTAATGGCAATTCATTGCATGAATTTTAAAAGAAGGGGGGGAATAGGGGATATGACTCCATCAGAAAGATTAATCAATATGATCACCACAGAACAAGAGATACAATTCCTCCAAGCCAAAAATTCAAAATTAAAAGATTTTCGGGTCTATTTCAGAGAAGGCAGAGATCAGTTGTGGAAAGGACCTGGGGAACTACTGTGGAAAGGAGAAGGAGCAGTCCTAGTCAAGGTAGGAACAGACATAAAAATAATACCAAGAAGGAAAGCCAAGATCATCAGAGACTATGGAGGAAGACAAGAGATGGATAGTGCAGTAGCAGGTAGA

>L02317

TTTTTAGATGGAATAGATAAGGCCCAAGAAGAACATGAGAAATATCACAGTAATTGGAGAGCCATGGCTAGTGATTTTAACTTACCACCTGTAGTAGCAAAAGAAATAGTAGCCAGCTGTGATAAATGTCAGCTAAAAGGAGAAGCCATGCATGGACAAGTAGACTGTAGTCCAGGAATATGGCAACTAGATTGCACACATCTAGAAGGAAAAATTATCCTGGTGGCGGTTCATGTAGCCAGTGGATATATAGAAGCAGAAGTTATTCCAGCAGAGACAGGGCAGGAAACAGCATACTTTCTCTTAAAATTAGCAGGAAGATGGCCAGTAAAAACAATACATACAGACAATGGCAGCAATTTCACCAGTACCACGGTTAAGGCCGCCTGTTGGTGGGCAGGGATCAAGCAGGAATTTGGCATTCCCTACAATCCCCAAAGTCAAGGAGTAGTAGAATCTATGAATAAAGAATTAAAGAAAATTATAGGACAGGTAAGAGATCAGGCTGAACATCTTAAAACAGCAGTACAAATGGCAGTATTTATCCACAATTTTAAAAGAAAAGGGGGGATTGGGGGGTACAGTGCAGGGGAAAGAATAGTAGACATAATAGCAACAGACATACAAACTAAAGAACTACAAAAACAAATTACAAAAATTCAAAATTTTCGGGTTTATTACAGGGACAACAAAGATCCACTTTGGAAAGGACCAGCAAAGCTTCTCTGGAAAGGTGAAGGGGCAGTAGTAATACAAGATAATAGTGACATAAAAGTAGTGCCAAGAAGAAAAGCAAAGATCATTAGAGATTATGGAAAACAGATGGCAGGTGATGATTGTGTGGCAAGTAGACAGGATGAGGAT

>M80194

TTCTTAGAAAAAATAGAACCAGCGCAGGAAGAGCATGAAAAATACCATAGCAATGTAAAAAAATTGGTATTCAAATTTRGTTTACCTAGGCTAGTAGCAAAACAAATAGTAGACACATGTGATAAATGCCACCTGAAAGGAGAAGCYATACATGGGCAAGTAAATGCRGRACTAGGGACTTGGCAAATGGACTGTACACACCTAGAGGGCAAAATAATTATAGTTGCAGTACATGTGGGTAGTGGATTCATAGAGGCAGAAGTAATCCCGCRGGAAACAGGAAGACAAACAGCACTGTTTCTGCTAAAATTAGCCAGCAGATGGCCCATCACACATCTGCATACTGATAATGGTGCCAATTTCACATCACAAGAAGTGAAAATGGTTGCTTGGTGGGCAGGRATTGAACAGACCTTTRGGGTACCTTATAATCCACAGAGCCAARGGGTAGTGGAAGCGATGAACCATCATCTAAAAACCCAAATAGATAGAATTAGAGAACAGGCAAATTCAATAGAAACTATAGTACTAATGGCAATTCATTGCATCAATTTTAAAAGAAGGGGAGGAATAGGGGATATGACTCCAGCAGAAAGATTAGTCAATATGATTACCACAGAACAAGAAATACAATTCCAACAATCAAAAAATTCAAAATTTAAAAATTTTCGGGTCTATTACAGAGAAGGCAGAGACCAGCTGTGGAAAGGACCCGGTGAGCTATTGTGGAAAGGGGAAGGAGCAGTCATCCTAAAGGTAGGGACAGAGATCAAGGTAGTACCAAGGAGGAAAGCTAAAATTATCAAAGACTATGGA

>X14307

ATAGAACCAGCACAAGAAGAGCATGAAAAGTACCATAGCAATGTAAAAGAATTGGTATTCAAATTTGGTTTACCTAGGCTAGTAGCAAAACAGATAGTAGACACATGTGATAAATGCCACCAGAAAGGAGAAGCCATACATGGGCAAGTAAATGCAGAACTAGGGACTTGGCAAATGGACTGTACGCACCTAGAAGGCAAAATAATTATAGTTGCAGTACATGTGGCTAGTGGATTTATAGAGGCAGAAGTAATCCCGCAGGAAACAGGAAGACAAACAGCACTGTTTCTGTTAAAGTTAGCTGGCAGATGGCCCATCACACATCTGCATACTGATAATGGTGCCAATTTCACATCACAAGAAGTGAAAATGGTTGCCTGGTGGGCAGGGATTGAACAGACCTTTGGGGTGCCTTATAATCCACAGAGCCAAGGAGTAGTGGAAGCAATGAACCATCATTTAAAAACCCAGATAGATAGAATTAGAGAACAAGCAAACTCAATAGAGACTATAGTACTAATGGCAGTTCATTGTATGAATTTTAAAAGAAGGGGAGGAATAGGGGATATGACTCCAGCAGAAAGATTAGTCAATATGATCACCACAGAACAAGAAATACAATTCCAACAATCAAAAAATTCAAAATTTAAAAATTTTCGGGTCTATTACAGAGAAGGCAGAGACCAGCTGTGGAAAGGACCCGGTGAGCTATTGTGGAAAGGGGAAGGAGCAGTCATCCTAAAGGTAGGGACAGAGATCAAGGTAGTACCAAGGAGGAAAGCTAAAATTATCAAAGACTATGGA

>M31113

TTCCTAGAGAAAATAGAACCCGCTCAGGAGGAACATGAAAAATATCATAGCAATGTAAAAGAACTATCCCATAAATTTGGACTGCCCAAATTAGTGGCAAGACAAATAGTAAACACATGCACCCAATGTCAGCAGAAAGGGGAGGCTATACATGGGCAAGTAAATGCAGAATTAGGCACTTGGCAAATGGACTGCACACACTTAGAAGGAAAAATCATTATAGTAGCAGTACATGTTGCAAGTGGATTTATAGAAGCAGAAGTCATCCCACAGGAATCAGGAAGGCAAACGGCACTCTTCCTACTAAAACTGGCCAGTAGGTGGCCAATAACACATTTGCACACAGACAATGGTGCCAACTTCACTTCACAGGAAGTAAAGATGGTGGCATGGTGGATAGGTATAGAACAATCCTTCGGAGTACCTTACAATCCACAAAGCCAAGGAGTAGTGGAAGCAATGAATCACCACCTAAAAAATCAGATAAGCAGAATTAGAGAGCAGGCAAACACAGTAGAAACAATAGTACTAATGGCAGTTCATTGCATGAATTTTAAAAGGAGGGGAGGAATAGGGGATATGACCCCAGCAGAAAGACTAATCAATATGGTCACTGCAGAACAGGAAATACAATTCCTCCAAGCAAAAAATTCAAAATTACAAAATTTTCGGGTCTATTTCAGAGAAGGCAGAGATCAGCTGTGGAAAGGACCTGGGGAACTACTGTGGAAGGGGGACGGAGCAGTCATAGTCAAGGTAGGGGCTGACATAAAAATAATACCAAGAAGGAAAGCTAAGATCATCAAAGACTATGGAGGAAGGCAAGAGATGGAT

>M83293

TTCCTAGAAAAAATAGAACCAGCTCAAGAAGAGCATGAAAAATATCATAGCAATGTGAAAGAGCTAGTCTTCAAGTTTGGCATACCAAGGCTAGTAGCAAAGCAAATAGTGGATACATGTGACAAGTGCCACCAGAAAGGAGAAGCTATACATGGGCAAGTAAATGCAGAGTTAGGGACTTGGCAGATGGACTGTACACATCTAGAAGGCAAGATAATTATAGTAGCAGTACATGTAGCTAGTGGGTTTATAGAGGCAGAAGTAATCCCACAAGAAACAGGGAGACAGACAGCATTGTTTCTGTTGAAGTTAGCAAGCAGATGGCCTGTCACACACCTGCACACGGATAATGGCGCTAACTTCACCTCGCAGGAGGTAAAGATGGTAGCCTGGTGGGCGGGAATAGAACAAACCTTTGGAGTACCTTACAATCCACAGAGCCAAGGAGTAGTGGAAGCCATGAATCATCATTTAAAGACCCAGATAGACAGAATTAGAGATCAAGCAAACACAGTAGAAACTGTAGTACTCATGGCAGTTCACTGCATGAATTTTAAGAAAAGGGGAGGACTAGGGGATATGACCCCAGCAGAAAGATTAGTTAATATGATCACCACAGAACAAGAAATACAATTTCAACAATCAAAAAATTCAAAATTCAAAAATTTTCGGGTCTATTACAGGGAAGGCAGAGATCAACTATGGAAGGGACCCGGTGAGCTATTGTGGAAAGGGGAAGGAGCAGTCATCGTAAAGGTAGGGACAGACATTAAAGTAGTACCAAGGAGAAAGGCTAAAATCATCAAAGATTATGGA

>L06042

TTCCTAGAAAGAATTCCCCAAGCACAGGAAGATCATGAAAGGTACCACTCAAATATGGAGTATCTTAGGCAGGAGTTTCATCTACCCCGACAGGTAGCAAAAGCAATTATACAACAATGTCCAAAATGTCAAAATAGAGGAGAACCAAAACATGGGCAAGTAGATGTAGATATATATAATTGGCAGATGGACTGTACTCATGAGGAAGGCAAAGTAATCTGTGTAGCAGTAAACACAGCTAGTGGATATATAGAAACCAAGATCTTAAAAAGGGAAACGGGGGATGAAACAGCATTGTTTCTCATGCAAATAGCCAGTAGATGGCCAATAAAACAGATACACACTGATAATGGACCAAACTTTGTCAGTGACAAGTTCAAAGCAGCATGTTGGTGGTGTGGCATAGAACACACTACAGGTATTCCATACAACCCACAAAGCCAGGGAATAGTAGAGTCCAAAAATAGGTATTTAAAAGAAGCTATCTCACAAATTAGAGACGACGTCACACATTTGCAAACAGCAGTGGCAATGGCAACCTTCATCTTAAATTTTAAAAGAAAGGGAGGAATAGGGGGGATATCACCAGGAGAAAGGTACATTAATATGCTATATACAGAACTACAATTACAACAA~~~~AATACAACATCACCAAAATTTCGAATTTTAGGGTTTACTACCGCCAGGGGAAGAACGAG~~~TGGAAGGGACCAGCTCGTCTACTTTGGAAAGGTGAAGGTGCAGTAGTGGTGCAAGAGGAGGGAGACATTTTTGCAGTACCAAGAAGAAAGGCAAAAATAATTACAGACCATGGAGAAAGAATG

>X07805

TTCTTAGAAAAAATAGAAGAAGCTCAAGAAAAGCATGAAAGATATCATAATAATTGGAAAAACCTAGCAGATACATATGGGCTTCCACAAATAGTAGCAAAAGAGATAGTGGCCATGTGTCCAAAATGTCAAATAAAGGGAGAACCAGTGCATGGACAAGTGGATGCCTCACCTGGAACATGGCAGATGGATTGTACTCATCTAGAAAAAAAAGTAGTCATAGTTGCGGTCCATGTAGCCAGTGGATTCATAGAAGCAGAAGTCATACCTAGGGAAACAGGAAAAGAAACGGCAAAGTTTCTATTAAAAATACTGAGTAGATGGCCTATAACACAGTTACACACAGACAATGGGCCTAACTTTACCTCCCAAGAAGTGGCAGCAATATGTTGGTGGGGAAAAATTGAACATACAACAGGTATACCATATAACCCCCAATCTCAAGGATCAATAGAAAGCATGAACAAGCAATTAAAAGAGATAATTGGGAAAATAAGAGATGATTGCCAATATACAGAGGCAGCAGTACTGATGGCTTGCATACTTCACAATTTTAAAAGAAAGGGAGGAATAGGGGGACAGACTTCAGCAGAGAGACTAATTAATATAATAACAACACAATTAGAAATACAACATTTACAAACCAAAATTCAAAAAATTTTAAATTTTAGAGTCTACTACAGAGAAGGGAGAGACCCTGTGTGGAAAGGACCGGCACAATTAATCTGGAAAGGGGAAGGAGCAGTGGTCCTCAAGGACGGAAGTGACCTAAAGGTTGTACCAAGAAGGAAAGCTAAAATTATTAAGGATTATGCCAAACAAAGAGTGGGTAATGAG

>M62320

TTTTTAGATGGGATAGATAAGGCTCAAGAGGACCATGAAAAATATCACTGCAACTGGAGAGCAATGGCTAGTGATTTTAATCTGCCACCTGTGGTAGCGAAGGAAATAGTAGCCAGCTGTAATAAATGTCAACTAAAAGGGGAAGCCATGCATGGACAAGTAGACTGTAGTCCAGGGATATGGCAATTAGATTGCACACATCTAGAAGGAAAAGTAATCCTAGTAGCAGTCCATGTAGCCAGTGGCTACATAGAAGCAGAAGTTATCCCAGCAGAAACAGGACAGGAGACAGCATACTTTATACTAAAATTAGCAGGAAGATGGCCAGTAAAAGTAATACACACAGACAATGGCAGCAATTTCACCAGCGCTGCAGTTAAAGCAGTCTGTTGGTGGGCAAATATCCAACAGGAATTTGGGATCCCCTACAATCCCCAAAGTCAAGGAGTAGTGGAATCCATGAACAAGGAATTAAAGAAAATCATAGGGCAGGTAAGAGAGCAAGCTGAACACCTTAAGACAGCAGTACAAATGGCAGTATTCATTCACAATTTTAAAAGAAAAGGGGGGATTGGGGGATACAGTGCAGGGGAAAGAATAATAGACATAATAGCAACAGACATACAAACTAAAGAATTACAAAAACAAATTTCAAAAATTCAAAATTTTCGGGTTTATTACAGGGACAGCAGAGACCCCATTTGGAAAGGACCAGCAAAACTACTCTGGAAAGGTGAAGGGGCAGTAGTAATACAAGACAATAGTGATATAAAGGTAGTACCAAGAAGAAAAGCAAAGATCATTAGGGATTATGGAAAACAGATGGCAGGTGATGATTGTATGGCAGGTAGACAGGATGAAGAT

>L07625

TTCCTAGAAAAGATAGAACCAGCACAAGAAGAACATGAAAAGTACCATGGCAATGTAAAAGAATTAGTTCATAAATTTGGACTCCCACAGTTAGTGGCAAAACAAATAGTAAACTCCTGTGATAAATGCCAACAAAAAGGGGAAGCTGTTCATGGACAGGTAAATGCAGAACTAGGAACATGGCAGATGGACTGTACACACTTAGAAGGAAAGGTCATAATAGTGGCAGTCCATGTAGCCAGTGGATTTATAGAGGCAGAGGTAATACCCCAAGAAACAGGAAGACAAACAGCTCTCTTCCTGTTAAAATTGGCCAGCAGATGGCCTATCACACACCTGCACACAGACAACGGCGCCAACTTCACCTCACAAGACGTGAAGATGGCGGCCTGGTGGATAGGGATAGAACAAACCTTCGGGGTACCCTATAACCCACAAAGTCAGGGAGTAGTAGAAGCAATGAACCATCACCTAAAAAACCAAATAGATAGAATCAGGGACCAGGCAGTATCAATAGAGACAGTTGTACTAATGGCAGCTCACTGCATGAATTTTAAAAGAAGGGGAGGAATAGGGGATATGACCCCTGCAGAGAGACTAGTTAACATGATAACCACAGAACAAGAAATACAGTTCTTCCAAGCAAAAAATTTAAAATTTCAAAATTTCCAGGTCTATTACAGAGAAGGCAGAGATCAACTCTGGAAAGGACCTGGTGAGCTATTGTGGAAAGGGGAAGGAGCAGTCCTCATAAAGGTAGGGACAGAGATCAAAGTAATACCCAGAAGGAAAGCAAAGATCATAAGGCACTATGGA

>U38293

TTCCTGGAGAAAATAGAGCCTGCCCAAGAGGAACATGAAAAATATCATAGCAATATAAAAGAGCTGATCCATAAATTTGGAATACCCCAACTAGTAGCAAGACAGATAGTAAACACATGTGCCCAATGCCAACAGAAAGGAGAGGCCATACATGGGCAAGTAAATGCAGAAATAGGCGTTTGGCAAATGGACTGCACACACTTAGAAGGAAAAATCATTATAGTAGCAGTACATGTTGCTAGTGGATTCATAGAAGCAGAGGTCATCCCACAGGAATCAGGAAGGCAGACAGCACTCTTCCTATTAAAACTGGCCAGTAGGTGGCCAATAACACACTTGCACACAGACAATGGCCCCAACTTCACTTCACAGGAAGTAAAAATGGTGGCATGGTGGGTAGGTATAGAGCAATCCTTTGGAGTACCTTACAACCCACAAAGCCAGGGAGTAGTAGAAGCAATGAATCACCACCTAAAGAATCAGATAAGTAGAATTAGAGAACAGGCTAATACAGTGGAAACAATAGTACTAATGGCAGTTCATTGCATGAATTTTAAAAGAAGGGGAGGAATAGGGGATATGACCCCGGCAGAAAGACTAATCAACATGATTACCACAGAACAAGAAATACAATTCCTCCAAAGAAAAAATTCAAATTTCAAAAAATTCCAGGTCTATTACAGAGAAGGCAGAGATCAGCTGTGGAAAGGCCCTGGAGAACTACTGTGGAAGGGAGAAGGAGCGGTCATAGTCAAGGTAGGGGCAGACATAAAAGTAGTACCAAGAAGGAAGGCCAAGATTATCAGGGACTATGGAGGAAGGCAACTAGGAGATGATTGTGTAAATAAAAGAAGA

>M22639

TTTTTGGATGGAATAGATAAAGCTCAAGAAGAACATGAGAAATATCACAACAATTGGAGAGCAATGGCTAGTGATTTTAACCTACCACCTGTGGTAGCAAAAGAAATAGTAGCTAGCTGTGATAAATGTCAGCTAAAAGGAGAAGCCATGCATGGACAAGTAGACTGTAGTCCAGGAATATGGCAATTAGATTGTACACATTTAGAAGGAAAAGTTATCCTGGTAGCAGTTCATGTAGCCAGTGGCTATATAGAAGCAGAAGTTATTCCAGCAGAAACAGGGCAGGAAACAGCATATTTTATTTTAAAATTAGCAGGAAGATGGCCAGTAAAAATAGTACATACAGACAATGGCAGCAATTTCACCAGTGCTGCAGTTAAGGCTGCCTGTTGGTGGGCAGGTATTAAACAGGAATTTGGAATTCCCTACAATCCCCAAAGTCAAGGAGTAGTAGAATCTATGAATAAAGAATTGAAGAAAATTATAGGACAGGTAAGAGATCAAGCTGAGCATCTTAAGACAGCTGTACAAATGGCAGTATTCATCCACAATTTTAAAAGAAAAGGGGGGATTGGGGGATACAGTGCAGGGGAGAGAATAATAGACATAATAGCAACAGACATACAAACTAAAGAATTACAAAAACAAATCACAAAAATTCAAAATTTTCGGGTTTATTACAGGGACAGCAGAGATCCAATTTGGAAAGGACCAGCAAAGCTCCTCTGGAAAGGTGAAGGGGCAGTAGTAATACAAGACAATAGTGACATAAAGGTAGTACCAAGAAGAAAAGTAAAGATTATCAGGGATTATGGAAAACAGATGGCAGGTGATGATTGTGTGGCAAGTAGACAGGATGAGGAT

>U51190

TTTTTAGATGGGATAGATAAAGCTCAAGAAGAACATGAAAGATATCACAGCAATTGGAGAGCAATGGCTAGTGATTTTAATCTGCCACCTATAGTAGCAAAGGAAATAGTAGCCAGCTGTGATAAATGTCAGCTAAAAGGGGAAGCCATGCATGGACAAGTAGACTGTAGCCCAGGGATATGGCAATTAGATTGTACACATCTAGAAGGAAAAGTAATTCTGGTAGCAGTCCATGTGGCTAGTGGCTACGTAGAAGCAGAAGTTATTCCAGCAGAAACAGGACAGGAGACAGCATACTTTCTACTAAAGCTAGCAGGAAGATGGCCAGTAAAAGTAGTACACACAGACAATGGCAGCAATTTCACCAGCGCTGCGGTTAAAGCAGCCTGTTGGTGGGCAAATGTTAAACAGGAATTTGGTATTCCCTACAATCCCCAAAGTCAAGGAGTAGTAGAATCTATGAATAAGGAATTAAAGAAAATCATAGGGCAGGTAAGGGAGCAAGCTGAACATCTTAAGACAGCAGTACAAATGGCAGTGTTCATTCACAATTTTAAAAGAAAAGGGGGGATTGGGGGGTACAGTGCAGGGGAAAGAATAATAGACATAATAGCATCAGACTTACAAACTAAAGAATTACAAAAACAAATTACAAAAATTCAAAAATTTCGGGTTTGTTACAGGGACAGCAGAGATCCAATTTGGAAAGGACCAGCAAAACTACTCTGGAAAGGTGAAGGGGCAGTGGTAATACAGGACAATAGTGATATAAAAGTAGTACCAAGAAGAAAAGTAAAGATCATTAAGGATTATGGAAAACAGATGGCAGGTGATGATTGTGTGGCAGGTAGACAGGATGAGGAT

>U58991

TTCTTAGACAGAATGGAAGAAGCCCAGGAATCACATGATAAATATCATACTAATTGGCAATTTATAAGAGATGCATTCGGTATACCAGCACTGGTAGCAAAAGAAATAGTAGCTGCTTGCCCAAAATGTCAAATTCGAGGAGAACCGATACATGGGCAAGTAGATGCCAGTGTAGGGGTCTGGCAAATGGACTGTACACATTTGGAAGGAAAAATCATTATAGTAGCAGTCCATGTGGCCAGTGGCTTCTTAGAAGCAGAAGTCATAGCTAGGGAAACAGGAAAAGAAACAGCAAAATTCCTGTTAAAAATCATTAGTAGGTGGCCTATTACAAAATTACATACTGACAATGGGCCAAATTTTGTTAGTCAGGAGGTACAAACTATTTGTTGGTGGGGACAAGTAGAGCACACAACAGGGATCCCCTATAACCCACAATCACAAGGAAGTGTAGAAAGTATGAATAGACAATTAAAAGAAACTATAGAAAAAATTAGAGAAGACTGTGCATTTTTGGAAACGGCAGTCCTCATGGCCTGCCACATTCACAATTTTAAAAGAAAGGGAGGAATAGGGGGTATGACCCCAGCAGAGAGGCTAATAAACATGATAACCACACAACTAGAAATACAACACATACAAACACAACAACAAAAAATTTCAAATTTTAAGGTCTACTACAGAGAAGGAAGAGAGCCAGTGTGGAAAGGACCAGCCACTCTCCTCTGGAAGGGAGAAGGAGCGGTAGTCATAAAAGAAGGTGAGGAGTTGAAAGTAGTACCAAGGAGAAAGGCAAAAATTATCAAAGAATATGGAGAGAGAAAAACTGTGGGTGAC

>U52953

TTTCTAGATGGAATAAATAAGGCTCAAGAAGAGCATGAAAAATATCACAGCAATTGGAGAGCAATGGCTAGTGAGTTTAATCTGCCACCCATAGTAGCAAAAGAAATAGTAGCTAGCTGTGATAAATGTCAGCTAAAAGGGGAAGCCACACATGGACAAGTAGACTGTAGTCCAGGAATATGGCAATTAGATTGTACACATTTAGAAGGAAAAATCATCCTGGTAGCAGTCCATGTAGCCAGTGGATACATAGAAGCAGAGGTTATCCCAGCAGAAACAGGGCAAGAAACAGCATACTTTATACTAAAATTAGCAGGAAGATGGCCAGTCAAAGTAATACATACAGACAATGGCAGTAATTTCATCAGTAATACAGTTAAAGCAGCCTGTTGGTGGGCAGGTATCCAACAGGAATTTGGAATTCCCTACAATCCCCAAAGTCAGGGAGTAGTAGAATCAATGAATAAAGAATTAAAGAAAATCATAGGACAGGTAAGAGATCAAGCTGAGCACCTTAAGACAGCAGTACAAATGGCAGTATTCATTCACAATTTTAAAAGAAAGGG~GGGATTGGGGGGTACAGTGCAGGGGAAAGAATAATAGATATAATAGCAACAGACATACAAACTAAAGAACTACAAAAACAAATTATGAAAATTCAAAATTTTCGGGTTTATTACAGAGACAGCAGAGATCCTATTTGGAAAGGACCAGCCAAACTACTCTGGAAAGGTGAAGGGGCAGTAGTACTACAAGATAACAGTGACATAAAGGTAGTACCAAGGAGGAAAGTAAAAATCATTAAGGACTATGGAAAACAGATGGCAGGTGCTGATTGTATGGCAAGTAGACAGGATGAAGAT

>U88824

TTTTTGGATGGAATAGATAAGGCTCAAGAAGAACATGAGAAATACCACAACAATTGGAGGGCAATGGCTAGTGAGTTTAACCTGCCACCTGTAGTAGCAAAAGAAATAGTAGCCAGCTGTGATAAATGTCAGGTGAAAGGAGAAGCCTTGCATGGACAAGTAGACTGTAGTCCAGGAATATGGCAATTAGATTGTACACATTTAGAAGGAAAAGGTATCCTGGTAGCAGTCCATGTAGCCAGTGGTTATATAGAAGCAGAAGTTATTCCAGCAGAAACAGGGCAGGAAACAGCCTACTTTCTTTTGAAATTAGCAGGAAGATGGCCAGTAAAAGTAGTACATACAGATAATGGCAGCAATTTCACCAGCGCTGCAGTAAAGGCCGCCTGTTGGTGGGCAGGTATCAAGCAGGAATTTGGAATTCCCTACAATCCCCAAAGTCAAGGAGTAGTAGAATCTATGAATAAAGAATTAAAGAAAATTATCGGGCAGGTAAGAGAACAAGCTGAACATCTTAAGACAGCAGTACAAATGGCAGTATTCATCCACAATTTTAAAAGAAAAGGGGGGATTGGGGGGTACAGTGCAGGGGAAAGAATAATAGATATCATAGCAACAGACATACAAACTAAGGAATTACAAAAACAAATCATAAAAATTCAAAATTTTCGGGTTTATTACAGGGACAGCAGAGATCCAGTTTGGAAAGGACCAGCAAAGCTTCTCTGGAAAGGTGAAGGGGCAGTAGTAATACAAGACAATAGTGAAATAAAGGTAGTACCAAGAAGAAAAGCAAAGATTATTAGGGATTATGGAAAACAGATGGCAGGTGATGATTGTGTGGCAAGTAGACAGGATGAGGAT

>U88825

TTTTTAGATGGCATAGACAAAGCTCAAGAGGACCATGAAAGATATCACAGCAATTGGAGAGCAATGGCTAGTGATTTTAATCTGCCACCTATAGTAGCAAAAGAAATAGTGGCCAGCTGTGATAAATGTCAGCTAAAAGGGGAAGCCATGCATGGACAAGTAGACTGTAGTCCAGGAATATGGCAATTAGATTGCACACATCTAGAAGGAAAAGTCATTATAGTAGCAGTCCATGTAGCCAGTGGCTATATAGAAGCAGAAGTTATCCCAGCAGAAACAGGACAGGAGACAGCATACTTCCTGCTAAAATTAGCAGGAAGATGGCCAGTAAAAGTAATACACACAGACAATGGCAGCAATTTCACCAGTGCTGCAATGAAAGCAGCCTGTTGGTGGGCAAATATCCAACAGGAATTTGGAATTCCCTACAATCCCCAAAGCCAAGGAGTAGTGGAATCTATGAATAAAGAATTAAAGAAAATTATAGGGCAGGTCAGGGATCAAGCTGAACACCTCAAGACAGCAGTACAGATGGCAGTATTCATTCACAATTTTAAAAGAAAAGGGGGGATTGGGGGGTACAGTGCAGGGGAAAGAATAATAGACATAATAGCATCAGATATACAAACTAAAGAACTACAAAAACAGATTATAAAAATTCAAAATTTTCGGGTCTATTACAGGGACAGCAGAGACCCCATTTGGAAAGGACCAGCAAAACTACTCTGGAAAGGTGAAGGGGCAGTAGTAATACAGGACAATAGTGAGATAAAGGTAGTACCAAGAAGAAAAGTAAAAATCATTAAGGATTATGGAAAACAGATGGCAGGTGGTGATTGTGTGGCAGGTAGACAGGATGAGGAT

>U88826

TTTTTGGATGGCATAGATAAAGCCCAAGAAGAACATGAAAGATATCACAGCAATTGGAGAGCAATGGCTAGTGATTTTAATCTGCCACCTGTAGTAGCAAAAGAAATAGTGGCCAGCTGTGATAAATGTCAACTAAAAGGGGAAGCCATGCATGGACAAGTAGACTGTAGTCCAGGAATATGGCAATTAGATTGTACACATTTAGAAGGAAAAATTATCATAGTAGCAGTTCATGTAGCCAGTGGCTATATAGAAGCAGAAGTTATCCCAGCAGAAACAGGGCAGGAAACAGCATACTTTATATTAAAATTAGCAGGAAGGTGGCCAGTAAAAGTGATACATACAGACAATGGTCCCAATTTCATCAGTGCTGCAGTAAAGGCAGCATGTTGGTGGGCAAATATCACACAGGAATTTGGAATTCCCTACAATCCCCAAAGCCAAGGAGTAGTGGAATCTATGAATAAGGAATTAAAGAAAATCATCGGACAGGTTGGAGATCAAGCTGAACATCTTAAGACAGCAGTACAGATGGCAGTATTCATTCACAATTTTAAAAGAAAAGGGGGGATTGGGGGGTACAGTGCAGGGGAAAGAATAATAGACATAATAGCATCAGATATACAAACTAAAGAACTACAAAAACAAATTATAAAAATTCAAAATTTTCGGGTTTATTACAGGGACAGCAGAGACCCAATTTGGAAAGGACCAGCAAAGCTACTCTGGAAAGGTGAAGGGGCAGTAGTAATACAGGACAATAACGAAATAAAGGTAGTACCAAGAAGAAAAGCAAAGATCCTTAAGGATTATGGAAAACAGATGGCAGGTGGTGATTGTGTGGCAGGTAGACAGGATGAGGAT

>AF004394

TTTTTGGATGGAATAGATAAGGCCCAAGAAGATCATGAGAAATATCACAGTAATTGGAGAGCAATGGCTAGTGATTTTAACCTGCCACCTATAGTAGCAAAAGAGATAGTAGCCAGCTGTGATAAATGTCAGCTAAAAGGAGAAGCCATGCATGGACAAGTAGACTGTAGTCCAGGAATATGGCAACTAGATTGTACACATTTAGAAGGAAAAATTATCCTGGTAGCAGTTCATGTAGCCAGTGGATATATAGAAGCAGAGGTTATTCCAGCAGAGACAGGACAGGAAACAGCATACTTTATCTTAAAATTAGCAGGAAGATGGCCAGTAACAACAATACATACAGACAATGGCACCAATTTCACCAGCACTACGGTTAAGGCCGCCTGTTGGTGGGCAGGGATCAAGCAGGAATTTGGCATTCCCTACAATCCCCAAAGTCAAGGGGTAGTAGAATCTATGAATAAAGAATTAAAGAAAATTATAGGACAGGTAAGAGATCAGGCTGAACATCTTAAGACAGCAGTACAAATGGCAGTATTCATCCACAATTTTAAAAGAAAAGGGGGGATTGGGGGATACAGTGCAGGGGAAAGAATAGTAGACATGATAGCAACAGACCTACAAACTAAAGAATTACAAAAACAAATTACAAAAATTCAAAATTTTCGGGTTTATTACAGGGACAGCAGAGATCCACTTTGGAAAGGACCAGCAAAGCTTCTCTGGAAAGGTGAAGGGGCAGTAGTAATACAAGATAATAGTGACATAAAAGTAGTGCCAAGAAGAAAAGCAAAAATCATTAGGGATTATGGAAAACAGATGGCAGGTGATGATTGTGTGGCAAGTAGACAGGATGAGGAT

>AF005494

TTTCTAGATGGGATAGATAAGGCACAAGAGGAACATGAAAAATATCACAACAATTGGAGAGCAATGGCTAGTGATTTTAATATACCAGCTGTAGTAGCAAAAGAAATAGTAGCTAGCTGTGATAAATGTCAGCTAAAAGGGGAAGCCATGCATGGACAAGTAGATTGTAGCCCAGGGATATGGCAATTAGATTGCACACATTTAGAAGGAAAAATTATCCTGGTAGCAGTCCATGTAGCTAGTGGGTACCTAGAAGCAGAAGTTATCCCAGCAGAAACAGGACAAGAGACAGCCTACTTCCTACTAAAGTTAGCAGGAAGATGGCCAGTAAAAACAATACATACAGACAATGGCACCAATTTCACCAGTGCCACGGTTAAGGCAGCTTGTTGGTGGGCAGGTATCCAGCAGGAATTTGGAATTCCTTACAACCCCCAAAGTCAAGGAGTAGTAGAATCTATGAATAAAGAGCTAAAGAAAATCATAGGACAGATAAGAGATCAAGCTGAACATCTTAAGACAGCAGTCCAAATGGCAGTATTCATTCACAATTTTAAAAGAAAAGGGGGGATTGGGGGATACAGTGCAGGGGAAAGAACAATAGACATAATAGCAACAGACATACAAACTAGAGAATTACAAAAACAAATTATAAAAATTCAAAATTTCCGGGTTTATTACAGGGACAGCAGAGACCCAGTTTGGAAAGGACCAGCAAAGCTACTCTGGAAAGGTGAAGGGGCAGTAGTCATACAAGACAATAGTGAAATAAAGGTAGTTCCAAGAAGAAAAGCAAAGATCATTAGGGATTATGGAAAACAGATGGCAGGTGATGATTGTGTGGCAGGTAGACAGGATGAGGAT

>AF005496

TTTCTAGATGGGATAGATAAAGCTCAAGAAGAACATGAAAGGTATCATAACAATTGGAGAGCAGTGGCTAGTGATTTTAATCTACCACCTATAGTAGCAAAAGAAATAGTAGCTAGCTGTGATAAATGTCAGCTAAAAGGGGAAGCCATGCATGGACAAGTAGACTGTAGCCCAGGAATATGGCAATTAGATTGCACACATTTGGAAGGACAAGTTATTCTGGTAGCAGTCCATGTAGCCAGTGGCTATATAGAAGCAGAAGTCATCCCAGCAGAAACAGGAAAGGAAACAGCATACTTCCTGTTGAAACTAGCAAGCAGATGGCCAGTAAAAGTAATACATACAGACAATGGCAGCAATTTCACGAGTGCTGCGGTTAAGGCAGCCTGTTGGTGGGCAGATATCCAACAGGAATTTGGGATTCCCTACAATCCCCAAAGTCAGGGAGTAGTAGAATCTATGAATAAAGAATTAAAGAAGATCATAGGGCAGGTAAGAGACCAAGCAGAACACCTTAAGACAGCAGTACAAATGGCAGTATTCATTCACAATTTTAAAAGAAAAGGGGGGATTGGGGGGTACAGTGCAGGGGAAAGAATAATAGACATAATAGCAACAGACATACAAACTAAAGAATTACAAAAACAAATTTCAAACATTCAAAAATTTCGGGTTTATTACAGGGACAGCAGAGACCCAATTTGGAAAGGACCAGCAAAACTCCTCTGGAAAGGTGAAGGGGCAGTAGTAATACAAGACAATAGTGAAATAAAAGTAGTACCAAGAAGAGAGGCAAAAATCATTAGGGATTATGGAAAACAGATGGCAGGTGATGATTGTGTGGCAAGTAGACAGGATGAGGAT

>AF004885

TTTTTAGATGGGATAGACAAAGCTCAAGAAGAACATGAAAGATATCATAGCAATTGGAGAACAATGGCTAGTGATTTTAATCTGCCACCTATAGTAGCAAAAGAAATAGTAGCCAGCTGTGATAAATGTCAGCTAAAAGGGGAAGCCATGCATGGACAAGTAGACTGCAGTCCAGGAATATGGCAATTAGATTGCACACATTTAGAAGGGAAAGTAATTGTGGTAGCAGTTCATGTAGCCAGTGGCTATATAGAAGCAGAAGTTATCCCAGCAGAAACAGGACAAGAGACAGCATACTTTCTACTAAAATTAGCAGGAAGATGGCCAGTAAAAATAGTACACACAGACAATGGCAGCAATTTCACCAGCGCTGCAGTTAAAGCAGCCTGTTGGTGGGCAAATATCCAACAGGAATTTGGGATTCCCTACAATCCCCAAAGTCAAGGAGTAGTGGAATCTATGAATAAGGAATTAAAGAAAATCATAGGGCAAGTAAGAGAGCAAGCTGAACACCTTAAAACAGCAGTACAAATGGCAGTATTCATTCACAATTTTAAAAGAAAAGGGGGGATTGGGGGGTACAGTGCAGGGGAAAGAATAATAGACATAATAGCAACAGACATACAAACTAAAGAATTACAAAAACACATTACAAAAATTCAAAATTTTCGGGTTTATTACAGGGACAGCAGAGATCCACTTTGGAAAGGACCAGCAAAACTATTCTGGAAAGGTGAAGGGGCAGTAGTAATACAGGACAATAGTGACATAAAGGTAGTACCAAGAAGAAAAGCAAAGATCATTAGGGATTATGGAAAACAGATGGCAGGCGATGATTGTGTGGCAGGTAGACAGGATGAGGAT

>U71182

TTTTTAGATGGAATAGATAAGGCCCAAGAAGAACATGAGAAATATCACTGTAACTGGAGAGCAATGGCTAGTGATTTTAACCTACCACCTGTAGTAGCAAAAGAAATAGTAGCCAGCTGTGATAAATGTCAGCTAAAAGGAGAAGCCATGCATGGACAAGTAGACTGTAGCCCAGGAATATGGCAATTAGATTGTACACATTTAGAAGGAAAAATTATCCTAGTAGCAGTTCATGTAGCCAGTGGATATATAGAAGCAGAAGTTATTCCAGCAGAGACAGGGCAGGAAACAGCATACTTTCTCCTAAAATTAGCAGGAAGATGGCCAGTGAAAACAATACATACAGACAATGGCAGAAATTTCACCAGTAATTCGGTTAAGGCCGCCTGTTGGTGGGCGGGGATCAAGCAGGAATTTGGCATTCCCTACAATCCCCAAAGTCAAGGCGTAGTAGAATCTATGGATAAAGAATTAAAGAAAATTATAGGACAGGTAAGAGATCAAGCTGAACATCTTAAGACAGCAGTACAAATGGCAGTATTCATCCACAATTTTAAAAGAAAAGGGGGGATTGGGGGGTACAGTGCAGGGGAAAGAATAGTAGACATAATAGCAACAGACATACAAACTAGAGAATTACAAAAACAAATTACAAAAATTCAAAATTTTCGGGTTTATTACAGGGGCAGCAGAGATCCACTTTGGAAAGGACCAGCAAAGCTCCTTTGGAAAGGTGAGGGGGCAGTAGTAATACAAGATAATAGTGACATAAAAGTAGTGCCAAGAAGAAAGGTAAAGATCATTAGGGACTATGGAAAACAGATGGCAGGTGATGATTGTGTGGCAAGTAGACAGGATGAGGAT

>U92049

TTTTTAGATGGCATAGATAAAGCCCAAGAAGAGCATGAAAGATATCACAGCAATTGGAGAGCAATGGCTAGTGATTTTAATTTGCCACCTATAGTAGCAAAAGAAATAGTGGCCAGCTGTGATAAATGTCAAATAAATGGGGAGGCCATGCATGGACAAGTAGACTGTAGTCCAGGGATATGGCAATTAGATTGTACACATTTAGAAGGAAAAGTTATCCTGGTAGCAGTCCATGTAGCCAGTGGTTATATAGAAGCAGAAGTTATCCCAGCAGAAACAGGACAGGAGACAGCATACTCTATATTAAAATTAGCAGGAAGATGGCCAGTGAAAATAATACACACAGACAATGGCAGCAATTTCACCAGTGCTGCAGTAAAAGCAGCATGTTGGTGGGCAAATGTCACACAAGAATTTGGAATTCCCTACAATCCCCAAAGTCAAGGAGTAGTGGAATCTATAAATAAAGAATTAAAGAAAATTATAGGGCAGGTCAGGGATCAAGCTGAACACCTTAAGACAGCAGTACAGATGGCAGTATTCATTCACAATTTTAAAAGAAAAGGGGGGATTGGGGGGTACAGTGCAGGGGAAAGAATAATAGACATAATAGCATCAGATATACAAACTAAAGAACTACAAAAACATATTACAAAAATTCAAAATTTTCGGGTTTATTACAGGGACAGCAGAGACCCAATTTGGAAAGGACCAGCAAAACTACTCTGGAAAGGTGAAGGGGCAGTAGTAATACAGGACAATAGTGATATAAAGGTAGTACCAAGAAGAAAAGCAAAGATCCTTGGAGATTATGGAAAACAGATGGCAGGTGATGATTGTGTGGCAGGTAGACAGGATGAGGAT

>AF042100

TTTTTAGATGGGATAGATAAGGCCCAAGAGGAGCATGAGAAATATCACAGTAATTGGAGAGCAATGGCTAGTGATTTTAACCTGCCACCTGTAGTAGCAAAAGAAATAGTAGCCAGCTGTGATAAATGTCAGCTAAAAGGAGAAGCCATGCATGGACAAGTAGACTGTAGTCCAGGAATATGGCAACTAGATTGTACACATTTAGAAGGAAAAGTTATCCTGGTAGCAGTTCATGTAGCCAGTGGATATATAGAAGCAGAAGTTATTCCAGCAGAGACAGGGCAGGAAACAGCATACTTTCTCTTAAAATTAGCAGGAAGATGGCCAGTAAAAGCAATACATACAGACAATGGCAGCAATTTCACCAGTACTACGGTTAAGGCCGCCTGCTGGTGGGCGGGGATCAAGCAGGAATTTGGCATTCCCTACAATCCCCAAAGTCAAGGAGTAGTAGAATCTATGAATAAAGAATTAAAGAAAATTATAGGACAGGTAAGAGATCAGGCTGAACATCTTAAGACAGCAGTACAAATGGCAGTATTCATCCACAATTTTAAAAGAAAAGGGGGGATTGGGGATTACTGTGCAGGGGAAAGAATAGTAGACATAATAGCAACAGACATACAAACTAAAGAATTACAGAAACAAATTACAAAAATTCAAAATTTTCGGGTTTATTACAGGGACAGCAGAGATCCACTTTGGAAAGGACCAGCAAAGCTCCTCTGGAAAGGTGAAGGGGCAGTAGTAATACAAGATAATAGTGACATAAAAGTAGTGCCAAGAAGAAAAGCAAAGATCATTAGGGATTATGGAAAACAGATGGCAGGTGATGATTGTGTGGCAGGTAGACAGGATGAGGAT

>AF042101

TTTTTAGATGGAATAGATAAGGCCCAAGAAGAACATGAGAAATATCACAATAATTGGAGAGCAATGGCTAGTGATTTTAACATACCACCTGTAGTAGCAAAAGAAATAGTAGCCTGCTGTGATAAATGTCAGCTAAAAGGAGAAGCCATGCATGGACAAGTAGACTGTAGTCCAGGAATATGGCAACTAGATTGTACACATTTAGAAGGAAAAGTTATCCTGGTAGCAGTTCATGTAGCCAGTGGATATATAGAAGCAGAAGTTATTCCAGCAGAGACAGGGCAGGAAACAGCATACTTTCTCTTAAAATTAGCAGGAAGATGGCCAGTAAAAACAATACATACAGACAATGGCCCCAATTTCACCAGTAATACAGTTAAGGCCGCCTGTTGGTGGGCGGGGATCAAGCAGGAATTTGGCATTCCCTACAATCCCCAAAGTCAAGGAGTAGTAGAATCTATAAATAAAGAATTAAAGAAAATTATAGGACAGGTAAGAGATCAGGCTGAACATCTTAAGACAGCAGTACAAATGGCAGTATTCATCCACAATTTTAAAAGAAAAGGGGGGATTGGGGGGTACAGTGCAGGGGAAAGAATAATAGACATAATAGCAACAGACATACAAACTAAAGAATTACAAAAACAAATTACAAAAATTCAAAATTTTCGGGTTTATTACAGGGACAGCAGAGATCCACTTTGGAAAGGACCAGCAAAGCTTCTCTGGAAAGGTGAAGGGGCAGTAGTAATACAAGATAATAGTGACATAAAAGTAGTGCCAAGAAGAAAAGCAAAGATCATTAGGGATTATGGAAAACAGATGGCAGGTGATGATTGTGTGGCAAGTAGACAGGATGAGGAT

>AF042103

TTTTTAGATGGAATAGATAAGGCACAAGAAGAACATGAGAAATATCACAGTAATTGGAGAGCCATGGCTAGTGATTTTAACCTGCCACCGGTAGTAGCAAAAGAAATAGTAGCCAGCTGTGATAAATGTCAGCTAAAAGGAGAAGCCACGCATGGACAAGTAGACTGTAGTCCAGGAATATGGCAACTAGATTGTACACATCTAGAAGGAAAAGTTATCCTGGTAGCAGTTCATGTAGCCAGTGGATATATAGAGGCAGAAGTTATTCCAGCAGAGACAGGGCAAGATACAGCATACTTTCTCTTAAAATTAGCAGGAAGATGGCCAGTAAAAACAATACATACAGACAATGGCAGCAATTTCATCAGTACTACAGTTAAGGCCGCCTGTTGGTGGGCGGGGATCAAGCAGGAATTTGGCATTCCCTACAATCCCCAAAGCCAAGGAGTTGTAGAATCTATGAATAAAGAATTAAAGAAAATTATAGGACAGGTAAGAGATCAGGCTGAACATCTTAAGACAGCCGTACAAATGGCAGTATTCATCCACAATTATAAAAGAAAAGGGGGGATTGGGGGGTACAGTGCAGGGGAAAGAATAGTAGACATAATAGCAACAGACATGCAAACTAAAGAATTACAAAAACAAATTACAAGAATTCAACATTTTCGGGTTTATTACAGGGACAGCAGAGATCCACTTTGGAAAGGACCAGCAAAGCTTCTCTGGAAAGGTGAAGGGGCAGTAGTAATACAAGATAATAGTGACATAAAAGTAGTGCCAAGAAGAAAAGTAAAGATCATTAGGGATTATGGAAAACAGATGGCGGGTGATGATTGTGTGGCAAGTAGACAGGATGAGGAT

>AF042105

TTTTTAGATGGAATAGATAAGGCACAAGAAGAACATGAGAAATATCACAGTAATTGGAGAGCAATGGCTAGTGATTTTAATCTGCCACCAGTAGTAGCAAAAGAAATAGTAGCCAGCTGTGATAAATGTCAGCTAAAAGGAGAAGCCATGCATGGACAAGTAGACTGTAGTCCAGGAATATGGCAACTAGATTGTACACATCTAGAAGAAAAAGTTATCCTGGTAGCAGTTCATGTAGCCAGTGGATATATAGAGGCAGAAGTCATTCCAGCAGACACAGCACAGGATACAGCATACTTTCTCTTAAGATTAGCAGGGAGATGGCCAGTAAAAACAATACATACAGACAATGGCAGCAATTTCATCAGTGCTACGGTTAAGGCCGCCTGTTGGTGGGCGGGGATCAAGCAAGAATTTGGCATACCCTACAATCCCCAAAGCCAAGGAGTAGTGGAATCTATGAATAGAGAATTAAAGAAAATTATAGGACAGGTAAGAGATCAGGCTGAACATCTTAAGACAGCAGTACAAATGGCAGTATTCATCCACAATTTTAAAAGAAAAGGGGGGATTGGGGGATACAGTGCAGGGGAAAGAATAGTAGACATAATAGCAACAGACATACAAACTAAAGAATTACAAAAACAAATTACAAAAATTCAAAATTTTCGGGTTTATTACAGGGACAGCAGAGACCCACTTTGGAAAGGACCAGCAAAGCTTCTCTGGAAAGGTGAAGGGGCAGTAGTAATACAAGATAATAGTGACATAAAAGTAGTGCCAAGAAGAAAAGTAAAGATCATCAGGGATTATGGAAAACAGATGGCAGGTGATGATTGTGTGGCAAGTAGACAGGATGAGGAT

>AJ006287

TTTTTAGATGGAATAGATAAAGCCCAAGAAGAACATGAAAAATATCACAGTAATTGGAGGGCAATGGCTAGTGATTTTAACCTGCCACCTGTGGTAGCAAAAGAGATAGTAGCCAGCTGTGATAAATGTCAGCTAAAAGGAGAAGCCATGCATGGACAAGTAGACTGTAGTCCAGGAATATGGCAACTAGATTGTACACATTTAGAAGGAAAAATTATCCTGGTAGCAGTTCATGTAGCCAGTGGATATATAGAAGCAGAAGTTATTCCAGCAGAAACAGGGCAGGAAACAGCATACTTTCTCTTAAAATTAGCAGGAAGATGGCCAGTAAAAACAGTACATACAGACAATGGCAGCAATTTCACCAGTACTACAGTTAAGGCCGCCTGTTGGTGGGCAGGAATCAAGCAGGAATTTGGCATTCCCTACAATCCCCAAAGTCAAGGAGTAGTAGAATCTATAAATAAAGAATTAAAGAAAGTTATAGGACAGATAAGAGATCAGGCTGAACATCTTAAGACAGCAGTACAAATGGCAGTATTCATCCACAATTTTAAAAGAAAAGGGGGGATTGGGGGGTACAGTGCAGGGGAAAGAATAGTAGACATAATAGCAACAGACATACAAACTAAAGAACTACAAAAACAAATTACAAAAATTCAAAATTTTCGGGTTTATTACAGGGACAGCAGAGATCCACTTTGGAAAGGACCAGCAAAGCTTCTCTGGAAAGGTGAAGGGGCAGTAGTAATACAAGATAATAGTGACATAAAAGTAGTGCCAAGAAGAAAAGCAAAGATCATTAGGGATTATGGAAAACAGATGGCAGGTGATGATTGTGTGGCAAGTAGACAGGATGAGGAT

>AF005495

TTTTTAGATGGAATAGATAAGGCCCAAGAAGAACATGAGAAATATCACAATAATTGGAGAGCAATGGCTAGTGACTTTAACATACCACCTGTAGTAGCAAAAGAAATAGTAGCCAGCTGTGATAAATGTCAGCTAAAAGGAGAAGCCATGCATGGACAAGTAGACTGTAGTCCAGGAATATGGCAGCTAGATTGTACACACTTAGAAGGAAAAGTTATCCTGGTAGCAGTGCATGTAGCCGGTGGATATATAGAAGCAGAAGTTATTCCAGCAGAGACAGGGCAAGAAACAGCATACTTTCTCTTAAAATTAGCAGGAAGATGGCCAGTAAAAACAATACACACAGACAATGGCAGCAATTTCACCAGTACTACAGTCAAGGCCGCCTGTTGGTGGGCGGGGATCAAGCAGGAATTTGGCATTCCCTACAATCCCCAAAGTCAAGGAGTAATAGAATCTATGAATAAAGAATTAAAGAAAATTATAGGACAGGTAAGGGATCAGGCTGAACATCTTAAGACAGCAGTACAAACGGCAGTATTCATCCACAATTTTAAAAGAAAAGGGGGGATTGGGGGGTACAGTGCAGGGGAAAGAATAGTAGACATAATAGCAACAGACATACAGACTAAAGAATTACAAAAACAAATTACAAAAATTCAAAATTTTCGGGTTTATTACAGAGACAGCAGAGATCCACTTTGGAAAGGACCAGCAAAGCTTCTCTGGAAAGGTGAAGGGGCAGTAGTAATACAAGATAATAGTGACATAAAAGTAGTGCCAAGAAGAAAAGTAAAGATCATTAGGGATTATGGAAAACAGATGGCAGGTGGTGATTGTGTGGCAGGTAGACAGGATGAGGAT

>AF067154

TTTCTAGATGGAATAGATAAGGCTCAAGAAGAGCATGAAAAGTATCACAGCAATTGGAGAGCAATGGCTAGTGACTTTAATCTGCCACCCATAGTAGCAAAAGAAATAGTAGCTAGCTGTGGTCAATGTCAGCAAAAAGGGGAAGCCATGCATGGACAAGTAGACTGTAGTCCAGGGATATGGCAATTAGATTGTACACATCTAGAAGGAAAAATCATCCTGGTAGCAGTCCATGTAGCCAGTGGTTACATAGAAGCAGAGGTTATCCCAGCAGAAACAGGACAAGAAACAGCATACTATATACTAAAATTAGCAGGAAGATGGCCAGTCAAAGTAATACATACAGACAATGGTAGTAATTTCACCAGTGCTGCAGTTAAAGCAGCCTGTTGGTGGGCAGGTATCCAACAGGAATTTGGAATTCCCTACAATCCCCAAAGTCAGGGAGTAGTAGAATCCATGAATAAGGAATTAAAGAAAATTATAGGGCAGGTAAGAGATCAAGCTGAGCACCTTAAGACAGCAGTACAAGTGGCAGTATTCATTCACAATTTTAAAAGAAGAGGGGGGATTGGGGGGTACAGTGCAGGGGAAAGAATAATAGACATAATAGCAACAGACATACAAACTAAAGAATTACAAAAACAAATTATAAAAATTCACAATTTTCGGGTTTATTACAGAGACAGCAGAGACCCTATTTGGAAAGGACCAGCCAAACTACTCTGGAAAGGTGAAGGGGCAGTAGTAATACAAGATAATAGTGACATAAAGGTAGTACCAAGGAGGAAAGCAAAAATCATTAGGGACTATGGAAAACAGATGGCAGGTGCTGATTGTGTGGCAGGTAGACAGGATGAAGAT

>AF067155

TTTCTAGATGGAATAGATAAAGCTCAAGAAGAGCATGAAAGGTATCACAGCAATTGGAGAGCGATGGCTAGTGACTTTAATCTGCCACCCGTAGTAGCAAAAGAAATAGTAGCTAGCTGTGATCAATGTCAGTTAAAAGGGGAAGCCACGCATGGACAAGTAGACTGTAGTCCAGGGATATGGCAATTAGATTGCACACATTTAGAAGGAAAAATCATCCTGGTAGCAGTCCATGTAGCCAGTGGCTACATGGAAGCAGAGGTTATCCCAGCAGAAACAGGACAAGAAACAGCATACTTTATACTAAAATTAGCAGGAAGATGGCCAGTCAAAGTAATACATACAGACAATGGTAGTAATTTCACAAGTGCTGCAGTTAAGGCAGCCTGTTGGTGGGCAGGTATCCAACAGGAATTTGGAATTCCCTACAATCCCCAAAGTCAGGGAGTAGTAGAATCCATGAATAAAGAATTAAAGAAAATTATAGGGCAAGTAAGAGATCAAGCTGAGCACCTTAAGACAGCAGTACAAATGGCAGTATTCATTCACAATTTTAAAAGAAAAGGGGGGATTGGGGGGTACAGTGCAGGGGAAAGAATAATAGACATAATAGCAACAGACATACAAACTAAAGAATTACAAAAACAAATTACAAAAGTTCAAAATTTTCGGGTTTATTACAGAGACAGCAGAGACCCCATTTGGAAAGGACCAGCCAAACTACTCTGGAAAGGTGAAGGGGCAGTAGTAATACAAGATAATAGTGACATAAAGGTGGTACCGAGGAGGAAAGCAAAAATCATTAAGGACTATGGAAAACAGATGGCAGGTGCTGATTGTGTGGCAGGTAGACAGGATGAAGAT

>AF067156

TTTCTAGATGGAATAGATAAGGCTCAAGAAGAGCATGAAAGGTATCACAGCAATTGGAGAGCAATGGCTAGTGACTTTAATCTGCCACCCATAATAGCAAAAGAAATAGTAGCTAGCTGTAATCAATGTCAGCTAAAAGGGGAAGCCATGCATGGACAAGTAGACTGTAGTCCAGGGATATGGCAATTAGATTGTACACATTTAGAAGGAAAAATCATCCTGGTAGCAGTCCATGTAGCCAGTGGCTACATAGAAGCAGAGGTTATCCCAGCAGAAACAGGACAAGAAACAGCATACTATATACTAAAATTAGCAGGAAGATGGCCAGTCAAAGTAATACATACAGACAATGGTAGTAATTTCACCAGTGCTGCAGTTAAGGCAGCCTGTTGGTGGGCAGGTATCCAACAGGAATTTGGAATTCCCTACAATCCCCAAAGTCAGGGAGTAGTAGAATCCATGAATAAGGAATTAAAGAAAATTATAGGGCAGGTAAGAGATCAAGCTGAGCACCTTAAGACAGCAGTACAAATGGCAGTATTCATTCACAATTTTAAAAGAAAAGGGGGGATTGGGGGGTACAGTGCGGGGGAAAGAATAATAGACATAATAGCAACAGACATACAAACTAAAGAATTACAAAAACAAATTATAAAAATTCAAAATTTTCGGGTTTATTACAGAGACAGCAGAGACCCTATTTGGAAAGGACCAGCCAAACTACTCTGGAAAGGTGAAGGGGCAGTAGTAATACAAGATAATAGTGACATAAAGGTAGTACCAAGGAGGAAAGCAAAAATCATTAAGGACTATGGAAAACAGATGGCAGGCGCTGATTGTGTGGCAGGTAGACAGGATGAAGAT

>AF067157

TTTCTAGATGGAATAGATAAAGCTCAAGAAGAGCATGAAAAGTATCACAGTAATTGGAGAGCAATGGCTAGTGACTTTAATCTGCCACCCGTAGTAGCAAAAGAAATAGTAGCTAGCTGTGATCAATGTCAGTTAAAAGGGGAAGCCATGCATGGACAAGTAGACTGTAGTCCAGGAATATGGCAATTAGATTGTACACATTTAGAAGGAAAAATCATCCTGGTAGCAGTCCATGTAGCTAGTGGCTACATAGAAGCAGAGGTTATCCCAGCAGAAACAGGACAAGAAACAGCATACTTTATACTAAAATTAGCAGGAAGATGGCCAGTCAAAGTAATACATACAGACAATGGTAGTAATTTCACCAGTGCTGCAGTTAAGGCAGCCTGTTGGTGGGCAAGTATCCAACAGGAATTTGGAATTCCCTACAATCCCCAAAGTCAGGGAGTAGTAGAAGCCATGAATAAAGAATTAAAGAAAATTATAGGGCAGGTAAGAGATCAAGCTGAGCACCTTAAGACAGCAGTACAAATGGCAGTATTCATTCACAATTTTAAAAGAAAAGGGGGGATTGGGGGGTACAGTGCAGGGGAAAGAATAATAGACATAATAGCAACAGACATACAAACTAAAGAATTACAAAAACAAATTACAAAAATTCAAAATTTTCGGGTTTATTACAGAGACAGCAGAGACCCCATTTGGAAAGGACCAGCCAAACTACTCTGGAAAGGTGAAGGGGCAGTAGTACTACAAGATAATAGTGACATAAAGGTAGTACCAAGGAGGAAAGCAAAAATCATTAAGGACTATGGAAAACAGATGGCAGGTGCTGATTGTGTGGCAGGTAGACAGGATGAAGAT

>AF067158

TTTCTAGATGGAATAGATAAAGCTCAAGAAGAGCATGAAAAGTATCACAGCAATTGGAGAGCAATGGCTAGTGACTTTAATCTGCCACCCGTAGTAGCAAAAGAAATAGTAGCCAGCTGTGATCAATGTCAGCTAAAAGGGGAAGCCATGCATGGACAAGTAGACTGTAGTCCAGGGATATGGCAATTAGATTGTACACATTTAGAAGGAAAAATCATCCTGGTAGCAGTCCATGTAGCCAGTGGCTACATAGAAGCAGAGGTTATCCCAGCAGAAACAGGACAAGAAACAGCATACTTTATACTAAAATTAGCAGGAAGATGGCCAGTCAAAGTAATACATACAGACAATGGTAGTAATTTCACCAGTGCTGCAGTTAAGGCAGCCTGTTGGTGGGCAGGTATCCAACAGGAATTTGGAATTCCCTACAATCCCCAAAGTCAGGGAGTAGTAGAATCCATGAATAAAGAATTAAAGAAAATTATAAGGCAGGTAAGAGATCAAGCTGAGCACCTTAAGACAGCAGTACAAATGGCAGTATTTATTCACAATTTTAAAAGAAAAGGGGGGATTGGGGGGTACAGTGCAGGGGAAAGAATAATAGACATAATAGCAACAGACATACAAACTAAAGAATTACAAAAACAAATTACAAAAATTCAAAATTTTCGGGTTTATTACAGAGACAGCAGAGACCCCATTTGGAAAGGACCAGCCAAACTACTCTGGAAAGGTGAAGGGGCAGTAGTAATACAAGATAATAGTGACATAAAGGTAGTACCAAGGAGGAAAGCAAAAATCATTAAGGACTATGGAAAACAGATGGCAGGTGCTGATTGTGTGGCAGGTAGACAGGATGAAGAT

>AF067159

TTCCTAGATGGAATAGATAAAGCTCAAGAAGAGCATGAAAAGTATCACAGCAATTGGAGAGCAATGGCTAGTGACTTTAATCTGCCACCCGTAGTAGCAAAAGAAATAGTAGCTAGCTGTGATCAATGTCAGCTAAAAGGGGAAGCCATGCATGGACAAGTAGATTGTAGTCCAGGGATATGGCAATTAGATTGTACACATTTAGAAGGAAAAATCATCCTAGTAGCAGTCCATGTAGCCAGTGGCTACATGGAAGCAGAGGTTATCCCAGCAGAAACAGGACAAGAAACAGCATACTTTATACTAAAATTAGCAGGAAGATGGCCAGTCAAAGTAATACATACAGACAATGGTAGTAATTTCACCAGTGCTGCAGTTAAGGCAGCCTGTTGGTGGGCAGGTATCCAACAGGAATTTGGAATTCCCTACAATCCCCAGAGTCAGGGAGTAGTAGAAGCCATGAATAAAGAATTAAAGAAAATTATAGGGCAGGTAAGAGATCAAGCTGAGCACCTTAAGACAGCAGTACAAATGGCAGTATTCATTCACAATTTTAAAAGAAAAGGGGGGATTGGGGGGTACAGTGCAGGGGAAAGAATAATAGACATAATATCAACAGACATACAAACTAGAGAATTACAAAAACAAATTATAAAAATTCAAAATTTTCGGGTTTATTACAGAGACAGCAGAGACCCCATTTGGAAAGGACCAGCCAAACTACTCTGGAAAGGTGAAGGGGCAGTAGTAATACAAGATAATAGTGACATAAAGGTAGTACCAAGGAGGAAAGCAAAAATCATTAAGGACTATGGAAAACAGATGGCAGGTGCTGATTGTGTGGCAGGTAGACAGGATGAAGAT

>AJ006022

TTCCTAGATGGTATAGAAAAAGCCCAAGAAGATCATGACAGATATCACAGCAATTGGAAAGCAATGGCCAGTGATTTTAACTTACCCCCCATAGTGGCAAAAGAAATAGTAGCCAGCTGTGACAAATGCCAGCTAAAAGGGGAAGCCATGCATGGACAGGTCAATTGTAGTCCAGGAGTGTGGCAATTAGATTGTACACACTTAGAGGGAAAAATCATCCTTGTGGCGGTCCATGTGGCCAGTGGCTACTTAGAAGCAGAAGTTATTCCTGCAGAGACAGGACAGGAAACAGCATATTTTATTTTAAAGTTAGCTGGAAGATGGCCAGTAAAAGTTATACACACTGATAATGGATCCAATTTCACTAGTGCCACTGTAAAAGCAGCCTGTTGGTGGGCAAATATCAAACAGGAATTTGGGATACCCTACAATCCTCAAAGTCAGGGAGCAGTAGAGTCCATGAATAAAGAATTAAAGAAAATTATAGGACAAATCAGAGATCAAGCAGAACATCTAAAGACAGCAGTGCAAATGGCGGTTTTCATTCACAATTTTAAAAGAAAAGGGGGGATTGGGGGGTACACTGCAGGGGAAAGAATAATAGACATAATAGCAACAGACATACAGACAACAAATTTACAAACACAAATTTTAAAAGTTCAAAATTTTCGGGTTTATTACAGAGACAGCAGAGATCCCATTTGGAAAGGACCAGCCAAACTTCTGTGGAAAGGAGAAGGGGCAGTGGTAATTCAAGATAACGGGGATATAAAAGTAGTCCCACGTAGGAAAGCAAAAATAATTAGGGATTATGGAAAACAGATGGCAGGTGATGGTTGTGTGGCAAGTGGACAGGATGAAAAT

>AF061642

TTTCTAGATGGCATAGATAAAGCCCAAGAAGAGCATGAGAGATATCACAACAACTGGAGAGCAATGGCCAGTGATTTTAATTTGCCACCTATAGTAGCAAAAGAAATAGTGGCCAGCTGTGATAAATGTCAGCTAAAAGGGGAAGCCATGCATGGACAAGTAGACTGTAGTCCAGGAATATGGCAATTAGATTGTACACATTTAGAAGGAAAAATTATCATAGTAGCAGTTCATGTAGCCAGTGGCTATATAGAAGCAGAAGTTATTCCAGCAGAAACAGGACAGGAAACAGCATACTTTATATTAAAATTAGCAGGAAGGTGGCCAGTAACAGTAATACATACAGATAATGGCAGCAATTTCACCAGTGCTGCAGTAAAGGCAGCATGTTGGTGGGCAAATATCACACAGGAATTTGGAATTCCCTACAATCCCCAAAGCCAAGGAGTAGTAGAATCTATGAATAAGGAATTAAAGAAAATCATCGGGCAGGTCAGGGATCAAGCTGAACATCTTAAGACAGCAGTACAGATGGCAGTATTCATTCACAATTTTAAAAGAAAAGGGGGGATTGGGGGGTACAGTGCAGGAGAAAGAATAATAGACATAATAGCATCAGATATACAAACTAAAGAACTACAAAAACAAATTACAAAAATTCAAAATTTTCGGGTTTATTACAGGGACAGCAGAGACCCAGTTTGGAAAGGACCAGCCAAACTACTCTGGAAAGGTGAAGGGGCAGTAGTAATACAAGACAATAACGAAATAAAGGTAGTACCAAGAAGAAAAGCAAAGATCATTAGGGATTATGGAAAACAGATGGCAGGTGATGATTGTGTGGCAGGTAGACAGGATGAGGAT

>AF061640

TTTTTAGATGGCATAGATAAAGCCCAAGAAGATCATGAAAAATATCACAGCAATTGGAGAGCAATGGCTAGTGATTTTAATCTGCCACATATAGTAGCAAAAGAAATAGTGGCCAGCTGTCATAAATGTCAGCTAAAAGGGGAAGCCATGCATGGTCAAGTAGACTGTAGTCCAGGAATATGGCAATTAGATTGTACACATTTAGAAGGAAAAATTATCCTGGTAGCAGTACATGTAGCCAGTGGGTATATAGAAGCAGAAGTTATCCCAGCAGAAACAGGACAGGAAACAGCATACTTCATACTAAAATTAGCAGGAAGGTGGCCAGTGAAAGTAATACATACAGACAATGGCAGCAATTTCACCAGTGCTGCAGTAAAGGCAGCGTGTTGGTGGGCAGATATTACACAAGAATTTGGAATTCCCTACAATCCCCAAAGCCAAGGAGTAGTAGAATCTATGAATAAAGAATTAAAGAAAATCATCGGGCAGGTCAGGGATCAAGCTGAACACCTTAAGACAGCAGTACAAATGGCAGTATTCATTCACAATTTTAAAAGAAAAGGGGGGATTGGGGGGTACAGTGCAGGGGAAAGAATAATAGACATAATAGCATCAGATATACAAACTAAAGAACTACAAACCCAAATTACAAAACTTCAAAATTTTCGGGTTTATTTCAGGGACAGCAGAGACCCAGTTTGGAAAGGACCAGCAAAACTGCTCTGGAAAGGTGAAGGGGCAGTAGTAATACAAGACAATAACGAAATAAAGGTAGTACCAAGAAGAAAAGCAAAAATTATTAGGGATTATGGAAAACAGATGGCAGGTGATGATTGTGTGGCAGGTAGACAGGATGAGGAT

>AF061641

TTTTTAGATGGCATAGATAAAGCCCAAGAAGATCATGAAAAATATCACAGCAATTGGAGAGCAATGGCTAGTGATTTTAATCTGCCACATATAGTAGCAAAAGAAATAGTGGCCAGCTGTCATAAATGTCAGCTAAAAGGGGAAGCCATGCATGGTCAAGTAGACTGTAGTCCAGGAATATGGCAATTAGATTGTACACATTTAGAAGGAAAAATTATCCTGGTAGCAGTACATGTAGCCAGTGGGTATATAGAAGCAGAAGTTATCCCAGCAGAAACAGGACAGGAAACAGCATACTTCATACTAAAATTAGCAGGAAGGTGGCCAGTGAAAGTAATACATACAGACAATGGCAGCAATTTCACCAGTGCTGCAGTAAAGGCAGCGTGTCGGTGGGCAGATATTACACAAGAATTTGGAATTCCCTACAATCCCCAAAGCCAAGGAGTAGTGGAATCTATGAATAAAGAATTAAAGAAAATCATCGGGCAGGTCAGGGATCAAGCTGAACACCTTAAGACAGCAGTACAAATGGCAGTATTCATTCACAATTTTAAAAGAAAAGGGGGGATTGGGGGGTACAGTTCAGGGGAGAGGATAATAGACATAATAGCATCAGATATACAAACTAAAGAACTACAAAAACAAATTACMCAAATTCAAAATTTTCGGGTTTATTTCAGGGACAGCAGAGACCCAGTTTGGAAAGGACCAGCAAAACTGCTCTGGAAAGGTGAAGGGGCAGTAGTAATACAAGACAATAACGAAATAAAGGTAGTACCAAGGAGAAAAGCAAAAATTATTAGGGATTATGGAAAACAGATGGCAGGTGATGATTGTGTGGCAGGTAGACAGGATGAGGAT

>AF063223

TTTTTAGATGGCATAGATAAAGCCCAAGAAGAGCATGGAAGATATCACAGCAATTGGAGAGCAATGGCTAGTGATTTTAATCTGCCACCTATAATAGCAAAAGAAATAGTGGCCTGCTGTGATCAATGTCAGCTGAAAGGGGAAGCCATGCATGGACAAGTAGACTGTGGTCCAGGAATATGGCAATTAGATTGTACACATTTAGAAGGAAAAATTATCCTGGTAGCAGTCCATGTAGCCAGTGGTTATATAGAAGCAGAAGTTATCCCAGCAGAAACAGGACAGGAGACAGCATACTTTATACTAAAATTAGCAGGAAGATGGCCAGTGAAAGTAATACACACAGACAATGGCAGCAATTTTACCAGTGCTGCAGTAAAGGCAGCATGTTGGTGGGCAAATGTCACACAGGAATTTGGAATTCCCTACAATCCCCAAAGCCAAGGAGTAGTGGAAGCTATGAATAAAGAATTAAAGAAAATCATAGGGCAGGTCAGGGATCAAGCTGAACACCTTAAGACAGCAGTACAGATGGCAGTATTCATTCACAATTTTAAAAGAAAAGGGGGGATTGGGGGGTACAGTGCAGGGGAAAGAATAATAGACATAATAGCATCAGATATACAAACTAAAGAACTACAAAAACAGATTACAAAAATTCAAAATTTTCGGGTCTATTACAGGGACAGCAGAGACCCCATTTGGAAAGGACCAGCAAAACTACTCTGGAAAGGTGAAGGGGCAGTAGTAATACAGGACAAGAGTGATATAAAGGTAGTACCAAGAAGAAAAGCAAAAATCATTAAAGATTATGGAAAACAGATGGCAGGTGATGATTGTGTGGCAGGTAGACAGGATGAGGAT

>AF063224

TTTTTAGATGGCATAGATAAAGCTCAAGAAGAGCATGAAAGATATCACAGCAATTGGAGAGCAATGGCTAGTGATTTTAATCTGCCACCTATAGTAGCAAAAGAAATAGTGGCCTGCTGCGATAAATGTCAGCTAAAAGGGGAAGCCATGCATGGGCAAGTAGACTGTAGTCCAGGAATATGGCAATTAGATTGTACACATTTAGAAGGAAAAATTATCCTGGTAGCAGTCCATGTAGCCAGTGGCTATATAGAAGCAGAAGTTATCCCAGCAGAAACAGGACAGGAGACAGCATACTTTATACTAAAATTAGCAGGAAGATGGCCAGTGAAAGTAATACACACAGACAATGGCAGCAATTTCACCAGTGCTGCAGTAAAGGCAGCATGTTGGTGGGCAAATGTCACACAGGAATTTGGAATTCCCTACAATCCCCAAAGCCAAGGAGTAGTAGAATCTATGAATAAGGAATTAAAGAAAATCATAGGGCAGGTCAGGGATCAAGCTGAACACCTTAAGACAGCAGTACAGATGGCAGTATTCATTCACAATTTTAAAAGAAAAGGGGGGATTGGGGGGTACAGTGCAGGGGAAAGAATAATAGACATAATAGCATCAGATATACAAACTAAGGAACTACAAAAACAGATTATAAAAATTCAAAATTTTCGGGTCTATTACAGGGACAGCAGAGACCCCATTTGGAAAGGACCAGCAAAACTACTCTGGAAAGGTGAAGGGGCAGTAGTAATACAGGACAAGAGTGATATAAAGGTAGTACCAAGAAGAAAAGCAAAAATCATTAAAGATTATGGAAAACAGATGGCAGGTGATGATTGTGTGGCAGGTAGACAGGATGAGGAT

>AF049495

TTTCTAGATGGAATAGATAAGGCCCAAGAAGAACATGAGAAATATCATAATAATTGGAGAGCAATGGCTAGTGATTTTAACCTGCCACCTGTAGTAGCAAAGGAAATAGTAGCCAGCTGTGATAAATGTCAGCTAAAAGGAGAAGCCATGCATGGACAAGTAGACTGTAGTCCAGGAATATGGCAACTAGATTGTACACATTTAGAAGGAAAAGTTATCCTAGTAGCAGTTCATGTAGCCAGTGGATATATAGAAGCAGAAGTTATTCCAGCAGAAACAGGGCAGGAAACAGCATACTTTCTCTTAAAATTAGCAGGAAGATGGCCAGTAAAAACAATACACACAGATAATGGCAGCAATTTCACCAGTGCTGCGGTTAAGGCCGCCTGTTGGTGGGCGGGAGTCAAGCAGGAATTTGGAATTCCCTACAATCCCCAAAGTCAAGGAGTAGTAGAATCTATGAATAAAGAATTAAAGAAAATTATAGGACAGGTAAGAGATCAAGCTGAACATCTTAAGACAGCAGTACAAATGGCAGTATTTGTCCACAATTTTAAAAGAAAAGGGGGGATTGGGGGGTACAGTGCAGGGGAAAGAATAATAGACATAATAGCAACAGACATACAAACTAGAGAACTACAAAAACAAATTACAAAAATTCAAAATTTTCGGGTTTATTACAGGGACAGCAGAGACCCACTTTGGAAAGGACCAGCAAAGCTCCTCTGGAAAGGTGAAGGGGCAGTAGTAATACAAGATAATAGTGACATAAAAGTAGTGCCAAGAAGAAAAGCAAAGATCATTAGGGATTATGGAAAACAGATGGCAGGTGATGATTGTGTGGCAAGTAGACAGGATGAGGAT

>AF049494

TTTTTAGATGGAATAGATAAGGCCCAAGAAGAACATGAGAAATATCACAATAATTGGAGAGCAATGGCTAGTGATTTTAACCTGCCACCTGTAGTAGCAAAAGAAATAGTAGCCAGCTGTGATAAATGTCAGCTAAAAGGAGAAGCTATGCATGGACAAGTAGACTGTAGTCCAGGAATATGGCAACTAGATTGTACACATTTAGAAGGAAAAGTTATCCTGGTAGCAGTTCATGTAGCCAGTGGATATATAGAAGCAGAAGTTATTCCAGCAGAAACAGGGCAGGAAACAGCATACTTTCTCTTAAAATTAGCAGGAAGATGGCCAGTAAAAACAATACATACAGACAATGGCAGCAATTTCACCAGTGCTGCGGTTAAGGCCGCCTGTTGGTGGGCAGGAGTCAAACAAGAATTTGGAATTCCCTACAATCCCCAAAGTCAAGGAGTAGTAGAATCTATGAATAAAGAATTAAAGAAAATTATAGGACAGGTAAGAGATCAAGCTGAACATCTTAAGACAGCAGTACAAATGGCAGTATTTGTCCACAATTTTAAAAGAAAAGGGGGGATTGGGGGGTACAGTGCAGGGGAAAGAATAATAGACATAATAGCAACAGACATACAAACTAGAGAACTACAAAAACAAATTACAAAAATTCAAAATTTTCGGGTTTATTACAGGGACAGCAGAGATCCACTTTGGAAAGGACCAGCAAAGCTCCTCTGGAAAGGTGAAGGGGCAGTAGTAATACAAGATAATAGTGACATAAAAGTAGTGCCAAGAAGAAAAGCAAAGATCATTAGGGATTATGGAAAACAGATGGCAGGTGATGATTGTGTGGCAAGTAGACAGGATGAGGAT

>U88822

TTTTTGGATGGAATAGATAAGGCTCAAGAGGAACATGAGAAATATCACAGCAATTGGAGAGCAATGGCTAGTGATTTTAACCTGCCACCTGTGGTAGCAAAAGAAATAGTAGCTAGCTGTGATAAATGTCAGCTAAAAGGAGAAGCAATGCATGGACAAGTAGACTGTAGTCCAGGAATATGGCAATTAGACTGTACACATTTAGAAGGAAAAGTTATCCTGGTAGCAGTTCATGTAGCCAGTGGCTATATAGAAGCAGAAGTTATTCCAGCAGAAACAGGGCAGGAAGCAGCATACTTTCTCTTAAAATTAGCAGGAAGATGGCCAGTAAAAGTAGTGCATACAGACAATGGCAGCAATTTCACCAGTGCTACAGTTAAGGCCGCCTGCTGGTGGGCAGGTATCAAGCAGGAATTTGGAATTCCCTACAATCCCCAAAGTCAAGGAGTAGTAGAATCTATGAATAAAGAATTAAAGAAAATTATAGGACAGGTAAGAGATCAAGCTGAACATCTTAAGACAGCAGTACAAATGGCAGTATTCATTCACAATTTTAAGAGAAAAGGGGGGATTGGGGGGTACAGTGCAGGGGAAAGAATAATAGACATAATAGCATCAGATATACAAACTAGAGAATTACAAAAACAAATCACAAAAATTCAAAATTTTCGGGTTTATTACAGGGACAGCAGAGATCCAATTTGGAAAGGACCAGCAAAGCTTCTCTGGAAAGGTGAAGGGGCAGTAGTAATACAAGACAATAGTGACATAAAGGTAGTACCAAGAAGAAAAGCAAAGATCATTAGGGATTATGGAAAACAGATGGCAGGTGATGATTGTGTGGCAAGTAGACAGGATGAGGAT

>AF077017

TTCTTAGAAAAAATAGAACCAGCACAAGAAGAGCATGAAAAGTACCATAGCAATGTAAAAGAATTGGTATTCAAATTTGGTATACCTAGGCTAGTAGCAAAACAGATAGTAGACACATGTCATAAATGCCACCAGAAAGGAGAAGCCATACATGGGCAAGTAAATGCAGAACTAGGGACTTGGCAAATGGACTGTACACACCTAGAGGGCAAAATAATCATAGTAGCAGTACATGTGGCTAGTGGATTCATAGAGGCAGAAGTAATTCCGCAGGAAACAGGAAGGCAAACAGCACTGTTTCTGCTAAAATTAGCTAGCAGATGGCCCATCACACATCTGCATACTGATAATGGTGCCAATTTCACATCACAAGAAGTGAAAATGGTTGCCTGGTGGGCAGGAATTGAGCAGACCTTCGGGGTACCTTATAATCCACAGAGCCAAGGAGTAGTGGAAGCAATGAACCATCATCTAAAAACCCAGATAGATAGAATTAGAGAACAGGCAAATTCAATAGAGACTATAGTACTAATGGCAGTTCATTGCATGAATTTTAAAAGAAGGGGAGGAATAGGGGATATGACTCCAGCAGAAAGATTAGTCAATATGATCACCACAGAACAAGAAATACAATTCCAACAATCAAAAAATTCAAAATTTAAAAATTTTCGGGTCTATTACAGAGAAGGCAGAGACCAGCTGTGGAAAGGACCCGGTGAGCTATTGTGGAAAGGGGAAGGAGCAGTCATCTTAAAGGTAGGGACAGAGATCAAGGTAGTACCAAGAAGGAAAGCTAAAATTATCAAAGATTATGGA

>AF086817

TTTTTGGATGGAATAGATAGGGCCCAAGAAGAACATGAGAAATACCACAGTAATTGGAGAGCAATGGCTAGTGATTTTAACCTGCCACCTGTAGTAGCAAAAGAAATAGTAGCCAGCTGTGATAAATGTCAGCTAAAAGGAGAAGCCATGCATGGACAAGTAGACTGTAGTCCAGGAATATGGCAACTAGATTGTACACATTTAGAAGGAAAAATTATCCTAGTAGCAGTTCATGTAGCCAGTGGGTATATAGAAGCAGAAGTTATTCCAGCAGAGACAGGACAGGAAACAGCATACTTTCTCTTAAAATTAGCAGGAAGATGGCCAGTAAAAACAATCCATACAGACAATGGCAGCAATTTCACTAGTGCTGCGGTTAAGGCCGCCTGTTGGTGGGCAGGGATCAAGCAGAAATTTGGCATTCCCTACAATCCCCAAAGTCAAGGAGTAATAGAATCTATGAATAAAGAATTAAAGAAAATTATAGGACAGGTAAGAGATCAGGCTGAACATCTTAAGACAGCAGTACAAATGGCAGTATTCATCCACAATTTTAAAAGAAAAGGGGGGATTGGGGGATACAGTGCAGGGGAAAGAATAGTAGACATAATAGCAACAGACATACAAACTAAAGAACTACAAAAACAAATTACAAAAATTCAAAATTTTCGGGTTTATTACAGAGACAACAGAGATCCACTTTGGAAAGGACCAGCAAAGCTGCTCTGGAAAGGTGAAGGGGCAGTAGTAATACAAGATAATAGTGACATAAAAGTAGTGCCAAGAAGAAAAGCAAAAATCATTAGGGATTATGGAAAACAGATGGCAGGTGATGATTGTGTGGCAGGTAGACAGGATGAGGAT

>AF064699

TTTTTAGATGGCATAGATAAAGCCCAGGAAGATCATGAAAGATATCACAGCAATTGGAGAGCCATGGCTAATGATTTTAATCTGCCACCTATAGTAGCAAAAGAAATAGTGGCCAGCTGTGACAAATGTCAGCTAAAAGGGGAAGCCATACATGGACAAGTAGATTGTAGTCCAGGGATATGGCAATTAGATTGCACACACCTAGAAGGAAAAATAATCCTGGTAGCAGTCCATGTAGCCAGTGGCTATATAGAAGCAGAAGTTATCCCAGCAGAAACAGGACAGGAGACAGCATACTTTATATTAAAATTAGCAGGAAGATGGCCAGTAAAAGTGATACACACAGACAATGGTAGCAATTTCACCAGTGCTGCAGTTAAAGCAGCCTGTTGGTGGGCAAATATCACACAAGAATTTGGAATTCCTTACAATCCCCAAAGTCAAGGAGTAGTGGAATCTATGAATAAAGAATTAAAGAAAATCATAGGGCAGGTAAGAGAACAAGCTGAACACCTTAAGACAGCAGTACAAATGGCAGTATTCATTCACAATTTTAAAAGAAAAGGGGGGATTGGGGGGTACAGTGCAGGGGAAAGAATAATAGACATAATAGCATCAGATATACAAACTAAAGAACTACAAAAACAAATTATAAAAATTCAAAATTTTCGGGTTTATTACAGGGACAGCAGAGACCCAATTTGGAAAGGACCAGCAAAACTACTCTGGAAAGGTGAAGGGGCAGTAGTAATACAAGACAATAGTGAAATAAAGGTAGTACCAAGAAGAAAAGCAAAGATCATTAGAGATTATGGAAAACAGATGGCAGGTGATGATTGTGTGGCAGGTAGACAGGATGAGGAT

>AF049337

TTTTTAGATGGAATAGATAAGGCTCAAGAAGAACATGAGAAATATCACAATAACTGGAGAGCAATGGCTAGTGATTTTAATCTGCCATCAGTGGTAGCAAAAGAGATAGTAGCTAGCTGTAATAAATGTCAGCTAAAAGGGGAAGCCATGCATGGACAAGTGGACTGTAGTCCAGGGATATGGCAGTTAGATTGTACACATTTAGAAGGTAAAGTTATCATGGTAGCAGTTCATGTGGCTAGTGGATACATAGAAGCAGAAGTTATCCCAGCAGAAACAGGACAGGAAACAGCCTACTTCATACTAAAATTAGCAGGAAGATGGCCAGTGAAAATGATACATGCAGACAACGGCCCCAATTTCACCAGTGCTGCGGTTAAGGCAGCCTGTTGGTGGGCAGATATCAACCAGGAATTTGGAATTCCCTACAATCCCCAAAGCCAAGGAGTAGTGGAATCTATGAATAAAGAATTAAAGAAAATCATAGGGCAGGTCAGGGATCAAGCTGAACACCTTAAGACAGCAGTACAGATGGCAGTATTCATTCACAATTTTAAAAGAAAAGGGGGGATTGGGGGGTACAGTGCAGGGGAAAGAATAATAGACATAATAGCATCAGATATACAAACTAAAGAACTACAAAAACAAATTACAAAAATTCAAAATTTTCGGGTTTATTACAGGGACAGCAGAGAACCAATTTGGAAGGGACCAGCAAAACTACTCTGGAAAGGTGAAGGGGCAGTAGTAATACAGGACAACAGTGATATCAAAGTAGTACCAAGAAGAAAAGCAAAGATTATTAGGGACTATGGCAAACAGATGGCAGGTAATGATTGTGTGGCAGGTAGACAGGATGAAGAT

>AF069673

TTTTTAGATGGGATAGATAAGGCTCAAGAAGAACATGAAAGATATCACAGTAATTGGAGAACAATGGCTAGTGATTTTAATCTGCCCCCTATAGTAGCAAAGGAAATAGTAGCCAGCTGTGATAAATGTCAACTAAAAGGGGAAGCCATGCATGGACAAGTAGACTGTAGTCCAGGGATGTGGCAATTAGATTGCACACATCTAGAAGGAAAAGTAATTCTGGTAGCAGTCCATGTAGCCAGTGGCTATATAGAAGCAGAAGTGATCCCAGCAGAAACAGGACAGGAAACAGCATACTTTCTGCTAAAATTAGCAGGAAGATGGCCAGTAAAAGTAGTACACACAGACAATGGCAGCAATTTCACCAGCGCTGCATTTAAAGCAGCCTGTTGGTGGGCAAATGTCCAACAGGAATTTGGGATCCCCTACAATCCCCAAAGTCAAGGAGTAGTGGAATCTATGAATAAGGAATTAAAGAAAATCATAGGGCAGGTAAGAGAACAAGCTGAACACCTTAAGACAGCAGTACAAATGGCAGTATTCATTCACAATTTTAAAAGAAAAGGGGGGATTGGGGGGTACAGTGCAGGGGAAAGAATAATAGACATAATAGCAACAGACATACAAACTAAAGAACTACAAAAACAAATTACAAAAATTCAAAATTTTCGGGTTTATTACAGGGACAGCAGAGATCCACTTTGGAAAGGACCAGCAAAACTACTCTGGAAAGGTGAAGGGGCAGTACTAATACAGGACAATAGTGATATAAAGGTAGTGCCCAGAAGAAAAGCAAAGATCATTAGGGATTATGGAAAACAGATGGCAGGTGATGGTTGTGTGGCAGGTAGACAGGATGAGGAT

>AF069672

TTTTTAGATGGGATAGATAAAGCTCAAGAAGAACATGAAAGATATCACAGCAATTGGAGAACAATGGCTAGTGATTTTAATCTGCCACCTGTAATAGCAAAAGAAATAGTAGCCAGCTGTGATAAATGTCAGCTAAAAGGGGAAGCCATACATGGACAAGTAGACTGTAGTCCAGGGATATGGCAATTAGATTGCACACATTTAGAAGGAAAAGTAATTCTGGTAGCAGTCCATGTAGCCAGTGGCTATATAGAAGCAGAAGTTATCCCAGCAGAAACAGGACAAGAGACAGCATACTTTCTACTAAAATTAGCAGGAAGATGGCCAGTAAAAGTAGTACACACAGACAATGGCAGCAATTTCACCAGCGCTGCAGTTAAAGCAGCCTGTTGGTGGGCCAATATCCAACAGGAATTTGGGATTCCCTACAATCCCCAAAGTCAAGGAGTAGTGGAGTCTATGAATAAGGAATTAAAGAAAATCATAGGACAGGTAAGAGAGCAAGCTGAACACCTTAAAACAGCAGTACAAATGGCAGTATTCATCCACAATTTTAAAAGAAAAGGGGGGATTGGGGGGTACAGTGCAGGGGAAAGAATAATAGACATAATAGCAACAGACATACAAACCAGAGAATTACAAAAACAAATTATAAAAATTCAAAATTTTCGGGTTTATTACAGGGACAGCAGAGATCCAATTTGGAAAGGACCAGCAAAACTACTCTGGAAAGGTGAAGGGGCAGTAGTAATACAAGACAATAGTGATATAAAGGTAGTACCAAGAAGAAAAGCAAAAATCATCAGGGATTATGGAAAACAGATGGCAGGTGATGATTGTGTGGCAGGTAGACAGGATGAGGAT

>AF069671

TTTTTAGATGGAATAGATAAAGCTCAAGAAGAACATGAAAGATATCACAGTAATTGGAGAACAATGGCTAGTGATTTTAATCTGCCACCCATAGTAGCAAAGGAAATAGTAGCCAGCTGTGATAAATGTCAGCTAAAAGGGGAAGCCATGCATGGACAAGTAGACTGCAGTCCAGGGATATGGCAATTAGATTGCACGCATCTAGAAGGAAAAGTAATTCTGGTAGCAGTCCATGTAGCCAGTGGCTATATAGAAGCAGAAGTCATTCCAGCAGAAACAGGACAAGAGACAGCATATTTTATACTAAAATTAGCAGGAAGATGGCCAGTAAAAGTAATACACACAGACAATGGCAGCAATTTCACCAGCGCTGCAGTTAAAGCAGCCTGTTGGTGGGCAAACGTCAAACAGGAATTTGGGATTCCCTACAATCCCCAAAGTCAAGGAGTAGTGGAATCTATGAATAAGGAATTAAAGAAAATCATAGGACAAGTAAGAGAGCAAGCTGAACACCTTAAAACAGCAGTACAAATGGCAGTATTCATTCACAATTTTAAAAGAAGAGGGGGGATTGGGGGATACAGTGCAGGGGAAAGAATAATAGACATAATAGCAACAGACATACAAACTAAAGAATTACAAAAACAAATTACAAAAATTCAAAATTTTCGGGTTTATTACAGGGACAGCAGAGATCCAATTTGGAAAGGACCAGCAAAACTACTCTGGAAAGGTGAAGGGGCAGTAGTAATACAGGACAATAGTGATATAAAGGTAGTACCAAGAAGAAAAGTAAAAATCATCAGGGATTATGGAAAACAGATGGCAGGTGATGATTGTGTGGCAGGTAGACAGGATGAGGAT

>AF069670

TTTTTAGATGGGATAGATAAAGCTCAAGAAGAGCATGAAAGATATCACAGCAACTGGAGAGCAATGGCTAGTGATTTTAATCTGCCACCTGTAATAGCAAAGGAAATAGTAGCCAGCTGTGATAAATGCCAGCTAAAAGGGGAAGCCATGCATGGACAAGTAGACTGCAGTCCAGGGATATGGCAACTAGATTGCACACATCTAGAAGGAAAAGTAATTCTGGTAGCAGTCCATGTGGCCAGTGGCTATATAGAAGCAGAAGTTATTCCAGCAGAAACAGGACAGGAGACAGCATACTTTCTACTAAAATTAGCAGGAAGATGGCCAGTAAAAATAGTACACACAGATAATGGCAGCAATTTCACCAGCGCTGCATTTAAAGCAGCCTGTTGGTGGGCAAGTATCCAACAGGAATTTGGAATTCCCTACAATCCCCAAAGTCAAGGAGTAGTGGAATCTATGAATAAGGAATTAAAGAAAATCATAGGGCAGGTAAGAGAGCAAGCTGAACATCTTAAGACAGCAGTACAAATGGCAGTATTCATTCACAATTTTAAAAGAAAAGGGGGGATTGGGGGGTACAGTGCAGGGGAAAGAATAATAGACATAATAGCAACAGACATACAAACTAAAGAATTACAAAAACAAATTACAAAAATTCAAAAATTTCGGGTTTATTACAGGGACAGCAGAGATCCCATTTGGAAAGGACCAGCAAAACTACTCTGGAAAGGTGAAGGGGCAGTGGTAATACAGGACAACAATGATATAAAGGTAGTACCAAGAAGAAAAGCAAAGATCCTTAGGGATTATGGAAAACAGATGGCAGGTGATGATTGTGTGGCAGGTAGACAGGATGAGGAT

>AF069669

TTTTTGGATGGGATAGATAAAGCTCAAGAAGAACATGAGAGATATCACAGCAATTGGAGAGCAATGGCTAGTGATTTTAATCTGCCACCTGTAATAGCAAAGGAAATAGTAGCCAGCTGTAATAAATGTCAACTAAAAGGAGAAGCCATGCATGGACAAGTAGACTGTAGTCCAGGGATGTGGCAATTAGATTGCACACATCTAGAAGGAAAAGTAATTCTGGTAGCAGTCCATGTAGCCAGTGGCTATATAGAAGCAGAAGTTATCCCAGCAGAAACAGGACAGGAGACAGCATACTTTCTGCTAAAATTAGCAGGAAGATGGCCAGTAAAAGTAGTACACACAGACAATGGCAGCAATTTCACCAGCGCTGCATTTAAAGCAGCCTGTTGGTGGGCAAATGTCCAACAGGAATATGGAATTCCCTACAATCCCCAAAGTCAAGGAGTAGTGGAATCTATGAATAAGGAATTAAAGAAAATCATAGGACAGGTAAGAGAGCAAGCTGAACACCTTAAAACAGCAGTACAAATGGCAGTATTCATTCACAATTTTAAAAGAAAAGGGGGGATTGGGGGGTACAGTGCAGGGGAAAGGATAATAGACATAATAGCAACAGACATACAAACTAAAGAATTACAAAAACAAATTACAAAAATTCAAAATTTTCGGGTTTATTACAGGGACAGCAGAGATCCAATTTGGAAAGGACCAGCAAAACTACTCTGGAAAGGTGAAGGGGCAGTAGTAATACAGGACAATAGTGATATAAAGGTAGTACCAAGAAGAAAAGCAAAAATCATTAGGGATTATGGAAAACAGATGGCAGGTGATGATTGTGTGGCAGGTAGACAGGATGAGGAT

>AF082339

TTTCTAGAAAAAATAGAGCCAGCTCAGGAAGAACATGAAAAGTATCATAGCAATGTGAAAGAACTATCCCATAAATTTGGATTACCCAATCTGGTGGCAAGACAGATAGTAAACACATGTGCCCAATGTCAGCAGAAGGGAGAGGCTATACATGGGCAAGTGAATGCAGAACTAGGCACTTGGCAAATGGACTGCACACACTTAGAAGGAAAAGTCATCATAATAGCAGTGCATGTTGCCAGTGGATTCATAGAAGCAGAGGTCATCCCGCAGGAATCAGGAAGACAAACAGCACTCTTCCTATTAAAACTGGCTAGTAGATGGCCAATAACACACTTGCACACAGATAGTGGTGTCAACTTCACCTCACAGGAAGTAAAGATGGTAGCATGGTGGGTTGGTATAGAGCAATCCTTTGGAGTACCTTACAATCCACAAAGCCAAGGAGTAGTAGAAGCAATGAATCACCACCTAAAAAATCAGATAAGCAGAATTAGAGAGCAGGCAAATACAGTGGAAACAATAGTACTAATGGCAGTTCATTGCATGAATTTTAAAAGAAGGGGAGGAATAGGGGATATGACCCCAGCAGAAAGACTCATCAATATGATCTCCACAGAACAAGAAATACAATTCCTCCAAACAAAAAATTTGAAATTTAAAAATTTCCCGGTCTATTACAGGGAAGGCAGAGATCAGCTGTGGAAAGGACCTGGGGAGCTACTGTGGAAAGGGGACGGAGCAGTCATAGTTAAGGTAGGGACAGACATAAAAGTAGTACCAAGAAGGAAGGCCAAGATCATCAGAGACTATGGAGGAAGGCAAGAGGCAGACAACTGCCCCAGGGGACTAGGGGATGAAGAG

>AF082394

TTTCTAGATGGGATAGATAAAGCTCAAGAAGATCATGAAAAATATCATAGCAATTGGAGAGCAATGGCTAGTGATTTTAATCTGCCACCTGTAGTAGCAAAAGAGATAGTAGCTAGCTGTGATAAATGTCAGCTAAAGGGGGAAGCCATGCATGGACAAGTAGACTGTAGTCCAGGGATATGGCAATTAGATTGTACACATCTAGAAGGAAAAGTTATCCTGGTAGCAGTTCATGTAGCCAGTGGCTATATAGAAGCAGAAGTTATCCCAGCAGAAACAGGACAGGAAGCAGCATTTTTTATATTAAAATTAGCAGGCAGATGGCCAGTAAAAGTAATACATACAGACAATGGCAGCAACTTCACCAGTGGTGCTGTGAAGGCAGCCTGTTGGTGGGCAGATATCAAGCAGGAATTTGGAATTCCCTACAATCCCCAAAGTCAAGGAGTAGTAGAATCTATGAATAAAGAATTAAAGAAAATCATAGGACAGGTAAGAGAACAAGCTGAACACCTTAAGACAGCAGTACAAATGGCAGTATTCATACACAATTTTAAAAGAAAAGGGGGGATTGGGGGATACAGTGCAGGGGAAAGAATAATAGACATAATAGCAACAGACATACAAACTAGAGAATTACAAAAACAAATTACAAAAATTCAAAATTTTCGGGTTTATTACAGGGACAGCAGAGACCCAATTTGGAAAGGACCAGCAAAACTACCCTGGAAAGGTGAAGGGGCAGTAGTAATACAGGACAATAGTGAAATAAAGGTAGTACCAAGAAGAAAAGCAAAGATCATTAGAGATTATGGAAAACAGATGGCAGGTGATGATTGTGTGGCAGGTAGACAGGATGAGGAT

>AF082395

TTTCTAGATGGGATAGATAAAGCTCAAGAAGAACATGAAAAATATCATAGCAATTGGAGAGCAATGGCTAGTGATTTTAATCTACCACCTGTAGTAGCAAAAGAAATAGTAGCTAGCTGTGATAAATGTCAGCTAAAGGGGGAAGCCATGCATGGACAAGTAGACTGTAGTCCAGGGATATGGCAATTAGATTGTACACATTTAGAAGGAAAAGTTATCCTGGTAGCAGTTCATGTAGCCAGTGGCTATATAGAAGCAGAAGTTATCCCAGCAGAAACAGGACAGGAAGCAGCATTTTTTATATTAAAATTAGCAGGCGGATGGCCAGTAAAAGCAATACATACAGATAATGGCAGCAACTTCACCAGTGGTGCTGTGAAGGCAGCCTGTTGGTGGGCAGATATCAAACAGGAATTTGGAATTCCCTACAATCCCCAAAGTCAAGGAGTAGTAGAATCTATGAATAAAGAATTAAAGAAAATCATAGGACAGGTAAGAGAACAAGCTGAACACCTTAAGACAGCAGTACAGATGGCAGTATTCATACACAATTTTAAAAGAAAAGGGGGGATTGGGGGATACAGTGCAGGGGAAAGAATAATAGACATAATAGCAACAGACATACAAACTAAAGAATTACAAAAACAAATCACAAAAATTCAAAATTTTCGGGTTTATTACAGGGACAGCAGAGACCCAATTTGGAAAGGACCAGCAAAACTGCTCTGGAAAGGTGAAGGGGCAGTAGTAATACAAGACAATAGTGAAATAAAGGTAGTACCAAGAAGAAAAGCAAAGATCATTAGAGATTATGGAAAACAGATGGCAGGTGATGATTGTGTGGCAGGTAGACAGGATGAGGAT

>AF084936

TTTTTAGATGGCATAGATAAAGCCCAAGAAGAGCATGAAAGATATCACAGCAATTGGAAAGCAATGGCTAGTGATTTTAATCTGCCACCTATAGTAGCAAAAGAAATAGTGGCCAGCTGTGATAAATGCCAGTTAAAAGGGGAAGCCATGCATGGACAAGTAGACTGTAGTCCAGGAATATGGCAATTGGATTGTACACATTTAGAAGGAAAAATTATCCTGGTAGCAGTCCATGTAGCCAGTGGCTATATAGAAGCAGAAGTTATCCCAGCAGAAACAGGACAGGAAACAGCATACTTTATATTAAAATTAGCAGGAAGGTGGCCAGTAAAAATAATACATACAGACAATGGCAGCAATTTTACCAGTGCTGCAGTAAAGGCAGCATGTTGGTGGGCAAGCATCACACAGGAATTTGGAATTCCCTACAATCCCCAAAGCCAAGGAGTAGTGGAATCTATGAATAAGGAATTAAAGAAAATCATAGGACAGGTCAGGGATCAAGCTGAACATCTTAAGACAGCAGTACAGATGGCAGTATTCATTCACAATTTTAAAAGAAAAGGGGGGATTGGGGGGTACAGTGCAGGGGAAAGAATAATAGACATAATAGCATCAGATATACAAACTAAAGAACTACAAAAACAAATTACAAAAATTCAAAATTTTCGGGTTTATTACAGGGACAGCAGAGATCCAATTTGGAAAGGACCAGCAAAACTACTCTGGAAAGGTGAAGGGGCAGTAGTAATACAAGACAATAACGAAATAAAAGTAGTACCAAGAAGAAAAGCAAAGATCATTAGGGATTATGGAAAACAGATGGCAGGTGATGATTGTGTGGCAGGTAGACAGGATGAGGAT

>AF103818

TTCCTAGATGGCATAGATAAAGCACAGGAAGACCATGACAAATATCATAGTAATTGGACAGCTATGGCCAGTGATTTTAACCTGCCACCAGTGGTCGCTAAGGAGATAGTGGCCAGCTGTGATAAATGTCAGCCAAAAGGAGAGGCCATACATGGGCAGGTAGATTGCAGTCCAGGTATCTGGCAGCTAGATTGTACACATTTAGAAGGGAAAATCATTATAGTGGCAGTACATGTGGCCAGTGGATACCTAGAAGCAGAGGTCATTCCTGCAGAAACAGGACAAGAGACAGCCTATTTTATCTTAAAATTAGCAGGAAGATGGCCTGTAAAAGTGATTCATACTGATAATGGATCTAACTTTACAAGTAGTACAGTTAGAGCAGCTTGCTGGTGGGCAGGCATACAACAAGAGTTTGGAATTCCATACAATCCACAAAGTCAAGGAGTGGTAGAATCCATGAATAAAGAATTAAAGAAAATCATAGGACAAATCAGGGATCAAGCAGAGCATTTAAAGACAGCTGTACAGATGGCAGTATTCATTCACAATTTTAAAAGAAAAGGGGGGATTGGGGGGTATACTGCAGGAGAAAGAATCATAGACATCATAGCATCAGAACTACAAACAGACTTATTACAAAAACAAATTTTAAAAGTTCAAAATTTTCGGGTCTATTACAGGGACAGCAGAGATCCAATTTGGAAAGGACCAGCCAAACTTCTGTGGAAAGGTGAAGGGGCAGTAGTAATCAAGGAAAACGAGGAGGTTAAAGTAGTACCCAGAAGAAAAGCAAAAATTATAAAAGACTATGGAAAACAGATGGCAGGTGCTGATAGTATGGCAGGTAGACAGGATGAG

>AF110969

TTTCTAGATGGAATAGATAAGGCTCAAGAAGAGCATGAAAAATATCACAGCAATTGGAGAGCTATGGCTAGTGAGTTTAATCTGCCACCCATAGTAGCAAAAGAAATAGTAGCCAGCTGTGATAAATGTCAGCTAAAAGGGGAAGCCATACATGGACAAGTAGACTGTAGTCCAGGGATATGGCAATTAGATTGTACACATTTAGAAGGAAAAGTCATCCTGGTAGCAGTCCATGTAGCCAGTGGCTACATAGAAGCAGAGGTTATCCCAGCAGAAACAGGACAAGAAACAGCATATTACATACTAAAATTAGCAGGAAGATGGCCAGTCAAAATAATACATACAGATAATGGCAGTAATTTCACCAGTGCTGCAGTTAAGGCAGCCTGTTGGTGGGCAGGTATCCAACAGGAATTTGGAATTCCCTACAATCCCCAGAGTCAGGGAGTAGTAGAATCCATGAATAAAGAATTAAAGAAAATCATAGGGCAGGTAAGAGATCAAGCTGAGCACCTTAAGACAGCAGTACAAATGGCAGTATTCATTCACAATTTTAAAAGAAAAGGGGGGATTGGGGGGTACAGTGCAGGGGAAAGAATAATAGACATGATAGCAACAGACATACAAACTAAAGAATTACAAAAACAAATTATAAAAATTCAAAATTTTCGGGTTTATTACAGAGACAGCAGAGACCCTATTTGGAAAGGACCAGCCAAGCTACTCTGGAAAGGTGAAGGAGCAGTAGTAATACAAGATAATAGTGACATAAAGGTAGTACCAAGGAGGAAAGTAAAAATCATTAGGGACTATGGAAAACAGATGGCAGGTGCTGATTGTGTGGCAGGTAGACAGGATGAGGAT

>AF110970

TTTCTAGATGGAATAGATAAGGCTCAAGAAGAGCATGAAAAATATCACAGCAATTGGAGAGCTATGGCTAGTGAGTTTAATCTGCCACCCATAGTAGCAAAAGAAATAGTAGCCAGCTGTGATAAATGTCAGCTAAAAGGGGAAGCCATACATGGACAAGTAGACTGTAGTCCAGGGATATGGCAATTAGATTGTACACATTTAGAAGGAAAAGTCATCCTGGTAGCAGTCCATGTAGCCAGTGGCTACATAGAAGCAGAGGTTATCCCAGCAGAAACAGGACAAGAAACAGCATATTACATACTAAAATTAGCAGGAAGATGGCCAGTCAAAATAATACATACAGACAATGGCAGTAATTTCACCAGTGCTGCAGTTAAGGCAGCCTGCTGGTGGGCAGGTATCCAACAGGAATTTGGAATTCCCTACAATCCCCAGAGTCAGGGAGTAGTAGAATCCATGAATAAAGAATTAAAGAAAATCATAGGGCAGGTAAGAGATCAAGCTGAGTACCTTAAGACAGCAGTACAAATGGCAGTATTCATTCACAATTTTAAAAGAAAAGGGGGGATTGGGGGGTACAGTGCAGGGGAAAGGATAATAGACATGATAGCAACAGACATACAAACTAGAGAACTACAAAAACAAATTATAAAAATTCAAAATTTTCGGGTTTATTACAGAGACAGCAGAGACCCTATTTGGAAAGGACCAGCCAAGCTACTCTGGAAAGGTGAAGGAGCAGTAGTAATACAAGATAATAGTGACATAAAGGTAGTACCAAGGAGGAAAGTAAAAATCATTAGGGACTATGGAAAACAGATGGCAGGCGCTGATTGTGTGGCAGGTAGACAGGATGAGGAT

>AF110971

TTTCTAGATGGAATAGATAAGGCTCAAGAAGAGCATGAAAAATATCACAGCAATCGGAGAGCTATGGCTAGTGAGTTTAATCTGCCACCCATAGTAGCAAAAGAAATAGTAGCCAGCTGTGATAAATGTCAGCTAAAAGGGGAAGCCATACATGGACAAGTAGACTGTAGTCCAGGGATATGGCAATTAGATTGTACACATTTAGAAGGAAAAGTCATCCTGGTAGCAGTCCATGTAGCCAGTGGCTACATAGAAGCAGAGGTTATCCCAGCAGAAACAGGACAAGAAACAGCATATTACATACTAAAATTAGCAGGAAGATGGCCAGTCAAAATAATACATACAGATAATGGTAGTAATTTCACCAGCGCCGCAGTTAAGGCAGCCTGTTGGTGGGCAGGTATCCAACAGGAATTTGGAATTCCCTACAATCCCCAGAGTCAGGGAGTAGTAGAATCCATGAATAAAGAATTAAAGAAAATCATAGGGCAGGTAAGAGATCAAGCTGAGCACCTTAAGACAGCAGTACAAATGGCAGTATTCATTCACAATTTTAAAAGAAAAGGGGGGATTGGGGGGTACAGTGCACGGGAAAGAATAATAGACATGATAGCAACAGACATACAAACTAAAGAATTACAAAAACAAATTATAAAAATTCAAAATTTTCGGGTTTATTACAGAGACAGCAGAGACCCTATTTGGAAAGGACCAGCCAAGCTACTCTGGAAAGGTGAAGGAGCAGTAGTAATACAAGATAATAGTGACATAAAGGTAGTACCAAGAAGGAAAGTAAAAATCATTAGGGACTACGGAAAACAGATGGCAGGCGCTGATTGTGTGGCAGGTAGACAGGATGAGGAT

>AF110979

TTTTTAGATGGAATAGATAAAGCTCAAGAAGACCATGAGAAATATCACGGCAATTGGAGAGCAATGGCTAATGAGTTTAATTTGCCACCCATAGTAGCAAAAGAGATAGTAGCTAGCTGTGATAAATGTCAGTTAAAAGGAGAAGCCATGCATGGACAGGTAGACTGTAGTCCAGGAATGTGGCAATTAGATTGTACACACTTAGAAGGGAAAGTTATCCTGGTAGCAGTCCATGTAGCCAGTGGCTACATAGAAGCAGAAGTAATCCCAGCAGAAACAGGACAGGAAACAGCAGACTTCATATTAAAATTAGCAGGAAGATGGCCAGTACAAATAATACATACAGACAATGGCAGCAATTTCACCAGCACTGCAGTCAAGGCAGCCTGTTGGTGGGCAGGGATCCAGCAGGAATTTGGAATTCCCTACAATCCCCAAAGTCAGGGAGTAGTAGAATCCATGAATAAAGAATTAAAGAAAATAATAGGACAAGTAAGAGATCAAGCTGAGCACCTTAAGACAGCAGTACTAATGGCAGTATTCATTCACAATTTTAAAAGAAAAGGGGGGATTGGGGGGTACAGTGCAGGGGAAAGAATAGTAGATATAATAGCAACAGACATACAAACTAAAGAACTACAAAAACAAATTATAAAAATTCAAAATTTTCGGGTTTATTACAGAGACAGCAGAGAACCCGTTTGGAAAGGACCAGCCAAATTGCTCTGGAAAGGTGAAGGGGCAGTAGTAATACAAGACAATAGTGACATAAAGGTAGTACCAAGGAGGAAAGCAAAGATCATTAGGGATTATGGAAAACAGATGGCAGGTGCTGATTGTGTGGCAGGTAGACAGGATGAAGAT

>AF110980

TTTTTAGATGGAATAGATAAAGCTCAAGAAGACCATGAGAAATATCACGGCAATTGGAGAGCAATGGCTAATGAGTTTAATTTGCCACCCATAGTAGCAAAAGAGATAGTAGCTAGCTGTGATAAATGTCAGTTAAAAGGAGAAGCCATGCATGGACAGGTAGACTGTAGTCCAGGAATATGGCAATTAGATTGTACACACTTAGAAGGGAAAGTTATCCTGGTAGCAGTCCATGTAGCCAGTGGCTACATAGAAGCAGAAGTAATCCCAGCAGAAACAGGACAGGAAACAGCATACTTCATATTAAAATTAGCAGGAAGATGGCCAGTACAAATAATACATACAGACAATGGCAGCAATTTCACCAGCACTGCAGTCAAGGCAGCCTGTTGGTGGGCAGGGATCCAGCAGGAATTTGGAATTCCCTACAATCCCCAAAGTCAGGGAGTAGTAGAATCCATGAATAAAGAATTAAAGAAAATAATAGGACAAGTAAGAGATCAAGCTGAGCACCTTAAGACAGCAGTACTAATGGCAGTATTCATTCACAATTTTAAAAGAAAAGGGGGGATTGGGGGGTACAGTGCAGGGGAAAGAATAGTAGATATAATAGCAACAGACATACAAACTAAAGAACTACAAAAACAAATTATAAAAATTCAAAATTTTCGGGTTTATTACAGAGACAGCAGAGAACCCGTTTGGAAAGGACCAGCCAAATTGCTCTGGAAAGGTGAAGGGGCAGTAGTAATACAAGACAATAGTGACATAAAGGTAGTACCAAGGAGGAAAGCAAAGATCATTAGGGATTATGGAAAACAGATGGCAGGTGCTGATTGTGTGGCAGGTAGACAGGATGAAGAT

>AF110981

TTTTTAGATGGAATAGATAAAGCTCAAGAAGACCATGAGAAATATCACGGCAATTGGAGAGCAATGGCTAATGAGTTTAATTTGCCACCCATAGTAGCAAAAGAGATAGTAGCTAGCTGTGATAAATGTCAGTTAAAAGGAGAAGCCATGCATGGACTGGTAGACTGTAGTCCAGGAATATGGCAATTAGATTGTACACACTTAGAAGGGAAAGTTATCCTGGTAGCAGTCCATGTAGCCAGTGGCTACATAGAAGCAGAAGTAATCCCAGCAGAAACAGGACAGGAAACAGCATACTTCATATTAAAATTAGCAGGAAGATGGCCAGTACAAATAATACATACAGACAATGGCAGCAATTTCACCAGCACTGCAGTCAAGGCAGCCTGTTGGTGGGCAGGGATCTAGCAGGAATTTGGAATTCCCTACAATCCCCAAAGTCAGGGAGTGGTAGAATCCATGAATAAAGAATTAAAGAAAATAATAGGACAAGTAAGAGATCAAGCTGAGCACCTTAAGACAGCAGTACTAATGGCAGTATTCATTCACAATTTTAAAAGAAAAGGGGGGATTGGGGGGTACAGTGCAGGGGAAAGAATAGTAGATATAATAGCAACAGACATACAAACTAAAGAACTACAAAAACAAATTATAAAAATTCAAAATTTTCGGGTTTATTACAGAGACAGCAGAGAACCCGTTTGGAAAGGACCAGCCAAATTGCTCTGGAAAGGTGAAGGGGCAGTAGTAATACAAGACAATAGTGACACAAAGGTAGTACCAAGGAGGAAAGCAAAGATCATTAGGGATTATGGAAAACAGATGGCAGGTGCTGATTGTGTGGCAGGTAGACAGGATGAAGAT

>AF110967

TTTCTAGATGGGATAGATAAGGCTCAAGAAGAGCATGAAAAATATCACAACAATTGGAGAGCAATGGCTGATGAATTTAATCTGCCACCCATAGTAGCAAAGGAAATAGTAGCTAGCTGTGATAAATGTCAGCTAAAAGGGGAAGCCATACATGGACAAGTAGACTGTAGTCCAGGAATATGGCAATTAGATTGTACACACTTAGAAGGAAAAGTCATCCTGGTAGCAGTCCATGTAGCCAGTGGCTACATGGAAGCAGAGGTTATCCCAGCAGAAACAGGACAGGAAACAGCATACTTCATACTAAAATTAGCAGGAAGATGGCCAGTCAGAGTAATACATACAGACAATGGTACTAATTTCACTAGTGCTGCAGTTAAGGCAGCCTGTTGGTGGGCAGGTATCCAACAGGAATTTGGAATTCCCTACAATCCCCAAAGTCAGGGAGTAGTAGAATCCATGAATAAAGAATTAAAGAAAATTATAGGGCAGGTAAGAGATCAAGCTGAGCACCTTAAGACAGCAGTACAAATGGCAGTATTCATTCACAATTTTAAAAGAAAAGGGGGGATTGGGGGGTACAGTGCAGGGGAAAGAATAATAGACATTATAGCAACAGACATACAAACTAAAGAACTACAAAAACAAATTATAAAAATTCAAAATTTTCGGGTTTATTACAGAGACAGCAGAGACCCTATTTGGAAAGGACCAGCCAAACTACTCTGGAAAGGTGAAGGGGCGGTAGTAATACAAGATAACAGTGACATAAAGGTAGTACCAAGGAGGAAAGCAAAAATCATTAAGGATTATGGAAAACAGATGGCAGGTGCTGATTGTGTGGCAGGTGGACAGGATGAAAAT

>AF110968

TTTCTAGATGGAATAGATAAGGCTCAAGAAGAGCATGAAAAATATCACAACAATTGGAGAGCAATGGCTGATGAATTTAATCTGCCACCCATAGTAGCAAAGGAAATAGTAGCTAGCTGTGATAAATGTCAGCTAAAAGGGGAAGCCATACATGGACAAGTAGACTGTAGTCCAGGAATATGGCAATTAGATTGTACACACTTAGAAGGAAAAGTCATCCTGGTAGCAGTCCATGTAGCCAGTGGCTACATGGAAGCAGAGGTTATCCCAGCAGAAACAGGACAGGAAACAGCATACTTCATACTAAAATTAGCAGGAAGATGGCCAGTCAGAGTAATACATACAGACAATGGTACTAATTTCACTAGTGCTGCAGTTAAGGCAGCCTGTTGGTGGGCAGGTATCCAACAGGAATTTGGAATTCCCTACAATCCCCAAAGTCAGGGAGTAGTAGAATCCATGAATAAAGAATTAAAGAAAATTATAGGGCAGGTAAGAGATCAAGCTGAGCACCTTAAGACAGCAGTACAAATGGCAGTATTCATTCACAATTTTAAAAGAAAAGGGGGGATTGGGGGGTACAGTGCAGGGGAAAGAATAATAGACATTATAGCAACAGACATACAAACTAAAGAACTACAAAAACAAATTATAAAAATTCAAAATTTTCGGGTTTATTACAGAGACAGCAGAGACCCTATTTGGAAAGGACCAGCCAAACTACTCTGGAAAGGTGAAGGGGCGGTAGTAATACAAGATAACAGTGACATAAAGGTAGTACCAAGGAGGAAAGCAAAAATCATTAAGGATTATGGAAAACAGATGACAGGTGCTGATTGTGTGGCAGGTGGACAGGATGAAAAT

>AF110976

TTTCTAGATGGAATAGATAAGGCTCAAGAAGATCATGAAAAGTATCACAGCAATTGGAGAGCGATGGCTAGTGACTTTAATCTGCCACCCATAGTAGCAAAAGAAATAGTAGCTAGCTGTGACAAATGTCAGCTAAAAGGAGAAGCCATGCATGGACAAGTAGACTGTAGCCCAGGGATATGGCAATTAGATTGTACACATTTAGAAGGAAAAATCATCCTGGTAGCAGTCCATGTAGCCAGTGGCTACATAGAAGCAGAGGTTATCCCAGCAGAAACAGGACAAGAAACAGCATACTTTATACTAAAATTAGCAGGAAGATGGCCAGTCAAAGTAATACATACAGACAATGGTAGTAATTTCACCAGTGCTACAGTTAAGGCAGCCTGTTGGTGGGCAGGCATCCAACAGGAATTTGGAATTCCCTACAATCCCCAAAGTCAGGGAGTAGTAGGATCCATGAATAAAGAATTAAAGAAAATTATAGGGCAGGTAAGAGATCAAGCTGAGCACCTTAAGACAGCAGTGCAAATGGCAGTATTCATTCACAATTTTAAAAGAAAAGGGGGGATTGGGGGGTACACTGCAGGGGAAAGAATAATAGACATAATAGCAACAGACATACAAACTAAAGAATTACAAAACCAAATTATAAAAATTCAAAATTTTCGGGTTTATTACAGAGACAGCAGAGACCCTATTTGGAAAGGACCAGCCAAACTACTCTGGAAAGGTGAAGGGGCAGTAGTACTACAAGATAATAGTGACATAAAGGTAGTACCAAGGAGGAAAGTAAAAATCATTAAGGACTATGGAAAACAGATGGCAGGTGCTGATTGTGTGGCAGGTGGACAGGATGAAAAT

>AF110977

TTTCTAGATGGAATAGATAAGGCTCAAGAAGATCATGAAAAGTATCACAGCAATTGGAGAGCGATGGCTAGTGACTTTAATCTGCCACCCATAGTAGCAAAAGAAATAGTAGCTAGCTGTGACAAATGTCAGCTAAAAGGGGAAGCCATGCATGGACAAGTAGACTGTAGCCCAGGGATATGGCAATTAGATTGTACACATTTAGAAGGAAAAATCATCCTGGTAGCAGTCCATGTAGCCAGTGGCTACATAGAAGCAGAGGTTATCCCAGCAGAAATAGGACAAGAAACAGCATACTTTATACTAAAATTAGCAGGAAGATGGCCAGTCAAAGTAATACATACAGACAATGGTAGTAATTTCACCAGTACTGCAGTTAAGGCAGCCTGTTGGTGGGCAGGCATCCAACAGGAATTTGGAATTCCCTACAATCCCCAAAGTCAGGGAGCAGTAGAATCCATGAATAAAGAATTAAAGAAAATTATAGGGCAGGTAAGAAATCAAGCTGAGCACCTTAAGACAGCAGTACAAATGGCAGTATTCATTCACAATTTTAAAAGAAAAGGGGGGATTGGGGGGTACACTGCAGGGGAAAGAATAATAGACATAATAGCAACAGACATACAAACTAAAGAATTACAAAACCAAATTATAAAAATTCAAAATTTTCGGGTTTATTACAGAGACAGCAGAGACCCTATTTGGAAAGGACCAGCCAAACTACTCTGGAAAGGTGAAGGGGCAGTAGTACTACAAGATAATAGTGACATAAAGGTAGTACCAAGGAGGAAAGTAAAAATCATTAAGGACTATGGAAAACAGATGGCAGGTGCTGATTGTGTGGCAGGTGGACAGGATGAAAAT

>AF110978

TTTCTAGATGGAATAGATAAGGCTCAAGAAGATCATGAAAAGTATCACAGCAATTGGAGAGCGATGGCTAGTGACTTTAATCTGCCACCCATAGTAGCAAAAGAAATAGTAGCTAGCTGTGACAAATGTCAGCTAAAAGGGGAAGCCATGCATGGACAAGTAGACTGTAGCCCAGGGATATGGCAATTAGATTGTACACATTTAGAAGGAAAAATCATCCTGGTAGCAGTCCATGTAGCCAGTGGCTACATAGAAGCAGAGGTTATCCCAGCAGAAACAGGACAAGAAACAGCATACTTTATACTAAAATTAGCAGGAAGATGGCCAGTCAAAGTAATACATACAGACAATGGTAGTAATTTCACCAGTGCTACAGTTAAGGCAGCCTGTTGGTGGGCAGGCATCCAACAGGAATTTGGAATTCCCTACAATCCCCAAAGTCAGGGAGTAGTAGAATCCATGAATAAAGAATTAAAGAAAATTATAGGGCAGGTAAGAGATCAAGCTGAGCACCTTAAGACAGCAGTACAAATGGCAGTATTCATTCACAATTTTAAAAGAAAAGGGGGGATTGGGGGGTACACTGCAGGGGAAAGAATAATAGACATAATAGCAACAGACATACAAACTAAAGAATTACAAAAACAAATTATAAAAATTCAAAATTTTCGGGTTTATTACAGAGACAGCAGAGACCCTATTTGGAAAGGACCAGCCAAACTACTTTGGAAAGGTGAAGGGGCAGTAGTACTACAAGATAATAGTGACATAAAGGTAGTACCAAGGAGGAAAGTAAAAATCATTAAGGACTATGGAAAACAGATGGCAGGTGCTGATTGTGTGGCAGGTGGACAGGATGAAAAT

>AF110973

TTTCTAGATGGAATAGATAAGGCTCAAGAAGAGCATGAAAAATATCACAGCAATTGGAGAGCAATGGCTAGTGAGTTTAATCTACCACCCATAGTAGCAAAAGAAATAGTAGCTAGCTGTGATAAGTGTCAGCTAAAAGGAGAAGCCATACATGGACAAGTAGACTGTAGTCCAGGAATATGGCAATTAGATTGTACACATTTAGAAGGAAAAATCATCCTGGTAGCAGTCCATGTAGCCAGTGGCTACATAGAAGCAGAGGTTATCCCAGCAGAAACAGGACAAGAAACAGCATACTATATACTAAAGTTAGCAGGAAGATGGCCAGTCAAAGTAATACATACAGACAATGGCAGTAATTTCACCAGTGCTGCAGTTAAGGCAGCCTGTTGGTGGGCAGGTATCCAACAGGAATTTGGGATTCCCTACAATCCCCAAAGTCAGGGAGTAGTAGAATCCATGAATAAAGAATTAAAGAAAATCATAGGGCAGGTAAGAGATCAAGCTGAGCACCTTAAGACAGCAGTACAAATGGCAGTATTCATTCACAATTTTAAAAGAAAAGGGGGGATTGGGGGGTATAGTGCAGGGGAGAGAATAATAGACATAATAGCAACAGACATACAAACTAAAGAATTACAAAAACAAATTACAAAAATTCAAAATTTTCGGGTTTATTACAGAGACAGCAGAGACCCTATTTGGAAAGGACCAGCCAAACTAATCTGGAAAGGTGAAGGAGCAGTAGTAATACAAGATAATAGTGACATAAAGGTAGTACCAAGGAGGAAAGTAAAAATCATTAGGGACTATGGAAAACAGATGGCAGGCGCTGATTGTGTGGCAGGTAGACAGGATGAGGAT

>AF110974

TTTCTAGATGGAATAGATAAGGCTCAAGAAGAGCATGAAAAATATCACAGCAATTGGAGAGCAATGGCTAGTGAGTTTAATCTACCACCCATAGTAGCAAAAGAAATAGTAGCTAGCTGTGATAAGTGTCAGCTAAAAGGAGAAGCCATACATGGACAAGTAGACTGTAGTCCAGGAATATGGCAATTAGATTGTACACATTTAGAAGGAAAAATCATCCTGGTAGCAGTCCATGTAGCCAGTGGCTACATAGAAGCAGAAGTTATCCCAGCAGAAACAGGACAAGAAACAGCATACTATATACTAAAGTTAGCAGGAAGATGGCCAGTCAAAGTAATACATACAGACAATGGCAGTAATTTCACCAGTGCTGCAGTTAAGGCAGCCTGTTGGTGGGCAGGTATCCAACAGGAATTTGGGATTCCCTACAATCCCCAAAGTCAGGGAGTAGTAGAATCCATGAATAAAGAATTAAAGAAAATCATAGGGCAGGTAAGAGATCAAGCTGAGCACCTTAAGACAGCAGTACAAATGGCAGTATTCATTCACAATTTTAAAAGAAAAGGGGGGATTGGGGGGTATAGTGCAGGGGAAAGAATAATAGACATAATAGCAACAGACATACAAACTAAAGAATTACAAAAACAAATTACAAAAATTCAAAATTTTCGGGTTTATTACAGAGACAGCAGAGACCCTATTTGGAAAGGACCAGCCAAACTACTCTGGAAAGGTGAAGGAGCAGTAGTAATACAAGATAATAGTGACATAAAGGTAGTACCAAGGAGGAAAGTAAAAATCATTAGGGACTATGGAAAACAGATGGCAGGCGCTGATTGTGTGGCAGGTAGACAGGATGAGGAT

>AF110975

TTTCTAGATGGAATAGATAAGGCTCAGGAAGAGCATGAAAAATATCACAGCAATTGGAGAGCAATGGCTAGTGAGTTTAATCTACCACCCATAGTAGCAAAAGAAATAGTAGCTAGCTGTGATAAGTGTCAGCTAAAAGGAGAAGCCATACATGGACAAGTAGACTGTAGTCCAGGAATATGGCAATTAGATTGTACACATTTAGAAGGAAAAATCATCCTGGTAGCAGTCCATGTAGCCAGTGGCTACATAGAAGCAGAAGTTATCCCAGCAGAAACAGGACAAGAAACAGCATACTATATACTAAAGTTAGCAGGAAGATGGCCAGTCAAAGTAATACATACAGACAATGGCAGTAATTTCACCGGTGCTGCAGTTAAGGCAGCCTGTTGGTGGGCAGGTATCCAACAGGAATCTGGGATTCCCTACAATCCCCAAAGTCAGGGAGTAGTAGAATCCATGAATAAAGAATTAAAGAAAATCATAGGGCAGGTAAGAGATCAAGCTGAGCACCTTAAGACAGCAGTACAAATGGCAGTATTCATTCACAATTTTAAAAGAAAAGGGGGGATTGGGGGGTATAGTGCAGGGGAAAGAATAATAGACATAATAGCAACAGACATACAAAGTAAAGAATTACAAAAACAAATTACAAAAATTCAAAATTTTCGGGTTTATTACAGAGACAGCAGAGACCCTATTTGGAAAGGACCAGCCAAAGTACTCTGGAAAGGTGAAGGAGCAGTAGTAATACAAGATAATAGTGACATAAAGGTAGTACCAAGGAGGAAAGTAAAAATCATTAGGGACTATGGAAAACAGATGGCAGGCGCTGATTGTGTGGCAGGTAAACAGGATGAGGAT

>AF119820

TTTTTAGATGGGATAGATAAGGCTCAAGAAGACCATGAGAAATATCACAGTAACTGGAGAGCAATGGCTAGTGATTTTAATCTGCCACCAGTAGTAGCAAAAGAGATAGTAGCTAGCTGTAATAAATGTCAGCTAAAAGGGGAAGCCATGCATGGACAAGWGGACTGTAGTCCAGGGATATGGCAATTAGACTGTACACATTTAGAAGGTAAAATTATCCTGGTACCAGTTCATGTAGCTAGTGGATATATAGAAGCAGAAGTTATCCCAGCAGAAACAGGACAGGAAACAGCCTACTTCATACTAAAATTAGCAGGAAGATGGCCAGTAAAAATAATACATACAGACAATGGCCCCAATTTCACCAGTGCTGCGGTTAAGGCAGCCTGTTGGTGGGCAGATGTCCAACAGGAATTTGGAATTCCCTACAATCCCCAAAGCCAAGGAGTAGTGGAATCTATGAATAAAGAATTAAAGAAAATCATAAAGCAGGTCAGGGATCAAGCTGAACACCTTAAGACAGCAGTACAGATGGCAGTATTCATTCACAATTTTAAAAGAAAAGGGGGGATTGGGGGGTACAGTGCAGGGGAAAGAATAATAGACATAATAGCATCAGATATACAAACTAAAGAACTACAAAAACAAATTACAAAAATTCAAAATTTTCGGGTTTATTACAGGGACAGCAGAGACCCAATTTGGAAGGGACCAGCAAAACTACTCTGGAAAGGTGAAGGGGCAGTAGTAATACAGGACAACAGTGATATAAAGGTAGTACCAAGAAGAAAAGCAAAGATTATCAGGGACTATGGAAAACAGATGGCAGGTGATGATTGTGTGGCAGGTAGACAGGATGAAGAT

>AF119819

TTTTTAGATGGAATAGATAAGGCTCAAGAAGAACATGAGAAATATCACAATAATTGGAAAGCAATGGCTAGTGATTTTAATCTGCCACCAGTAGTAGCAAAAGAGATAGTAGCTAGCTGTAATAAATGTCAGCTAAAGGGGGAAGCCATGCATGGACAAGTGGACTGTAGTCCAGGGATATGGCAATTAGACTGTACACATTTAGAAGGTAAAATTATCCTGGTAGCAGTTCATGTAGCTAGTGGATATATAGAAGCAGAAGTTATCCCAGCAGAAACAGGACAGGAAACAGCTTACTTCATACTAAAATTAGCAGGAAGATGGCCAGTAAAAATAATACATACAGACAATGGCTCCAATTTCACCAGTGCTGCGGTTAAGGCAGCCTGTTGGTGGGCAAATATCCAACAGGAATTTGGAGTTCCCTACAATCCCCAAAGCCAAGGAGTAGTGGAATCTATGAATAAAGAATTGAAGAAAATCATAGGGCAGGTCAGAGATCAAGCTGAACACCTTAAGACAGCAGTACAAATGGCAGTATTCATTCACAATTTTAAAAGAAAAGGGGGGATTGAGGGGTACAGTGCAGGGGAAAGAATAATAGACATAATAGCATCAGATATACAAACTAAAGAACTACAGAAACAAATTATAAAAATTCAAAATTTTCGGGTTTATTACAGGGACAGCAAAGACCCAATTTGGAAGGGACCAGCAAAACTACTCTGGAAAGGTGAAGGGGCAGTAGTAATACAGGACAACAGTGATATAAAGGTAGTACCAAGAAAAAAAGCAAAAATCATTAGGGACTATGGAAAACAGATGGCAGGTGATGACTGTGTGGCAGGTAGACAGGATGAAGAT

>AF075701

TTCTTGGATGGGATAGATAAGGCTCAAGAAGAACATGAAAAATACCACAACAATTGGAGAGCAATGGCTAGTGATTTTAACCTGCCGCCTGTGGTAGCAAAAGAAATAGTAGCTAGCTGTGATAAATGTCAGCTAAAAGGAGAAGCCTTGCATGGACAAGTAGACTGTAGTCCAGGAATATGGCAATTAGATTGTACACATTTAGAAGGAAAAGTTATCCTGGTAGCAGTCCATGTAGCCAGTGGCTATATAGAAGCAGAAGTTATTCCAGCAGAAACAGGGCAGGAAACAGCCTACTTTCTCTTGAAATTAGCAGGAAGATGGCCAGTAAGAGTAGTACATACAGACAATGGCAGCAATTTCACCAGCACTGCAGTTAAGGCCGCCTGTTGGTGGGCAGGCATCAAGCAGGAGTTTGGGATTCCCTACAATCCCCAAAGTCAAGGAGTAGTAGAATCTATGAATAAGGAATTGAAGAAAATTATAGGGCAGGTAAGAGATCAAGCGGAACATCTTAGGACAGCAGTACAAATGGCAGTATTCATCCACAATTTTAAAAGAAAAGGGGGGATTGGGGGGTACAGTGCAGGGGAAAGAATAATAGACATAATAGCAACAGACATACAAACTAAAGAATTACAAAGACAAATCACAAAAATTCAAAATTTTCGGGTTTATTACAGGGACAGCAGAGATCCAATTTGGAAAGGACCAGCAAAACTTCTCTGGAAAGGTGAAGGGGCAGTAGTAATACAAGACAATAGTGAAATAAAGGTAGTACCAAGAAGAAAAGTAAAGATCATTAGGGATTATGGAAAACAGATGGCAGGTGATGATTGTGTGGCAAGTAGACAGGATGAGGAT

>AF077336

TTTTTAGATGGGATAGATAAGGCACAAGAAGAACATGAAAAATATCACAACAATTGGAGAGCGATGGCTAGTGATTTTAATCTGCCACCTATAGTAGCAAAAGAAATAGTAGCTAGCTGTGATAAGTGTCAGCTAAAAGGGGAAGCCATGCACGGACAAGTAGACTGTAGTCCAGGGATATGGCAATTAGATTGCACACATTTAGAAGGCAAGGTTATCCTGGTAGCAGTCCATGTAGCTAGTGAGTACATAGAAGCAGAAGTTATTCCAGCAGAAACAGGACAGGAAACAGCCTACTTCATATTAAAGTTAGCAGGAAGATGGCCAGTAAAAATAATACATACAGACAATGGCAGCAATTTCACCAGTGCCGCGGTTAAGGCATCCTGTTGGTGGGCAGGTATCCAACAGGAATTTGGAATCCCCTACAATCCCCAAAGTCAAGGAGTAGTAGAATCTATAAATAAAGAGTTAAAAAAGATCATAGGACAGGTAAGAGATCAAGCTGAACATCTTAAGACAGCAGTACAAATGGCAGTATTCATCCACAATTTTAAAAGAAAAGGGGGGATTGGGGGGTACAGTGCAGGGGAAAGAATAATAGACATAATATCAACAGACATACAAACTAGAGAATTACAAAAACAAATTACGAAAATTCAAAATTTCCGGGTTTATTACAGGGACAGCAGAAACCCAGTTTGGAAAGGACCAGCAAAGCTACTCTGGAAAGGTGAAGGGGCAGTAGTCATACAAGACAATAGTGAAATAAAGATAGTACCAAGGAGGAAAGCAAAGATCATTAGGGATTATGGAAAACAGATGGCAGTTGATGATTGTGTGGCAGGTAGACAGGATGAGGAT

>AF076475

TTTTTAGATGGAATAGATAAGGCTCAAGAAGAGCATGAAAAATATCACAATAATTGGAGAGCAATGGCTAGTGATTTTAATCTGCCACCAATAGTAGCAAAAGAGATAGTAGCTAGCTGTGATAAATGTCAACTAAAAGGGGAAGCCATGCATGGACAAGTAGACTGTAGCCCAGGAATATGGCAATTAGATTGTACACATTTAGAAGGAAAAATTATCCTGGTAGCAGTCCATGTAGCCAGTGGCTATATAGAAGCAGAAGTTATCCCAGCAGAAACAGGACAGGAGACAGCCTTCTTCATATTAAAGTTGGCAGGAAGATGGCCAGTAAAAATAATACATACAGACAATGGCAGCAATTTCATCAGTGCTACGGTTAAGGCAGCCTGTTGGTGGGCAGGTATCCAGCAGGAATTTGGAATTTCCTACAATCCCCAGAGTCAAGGAGTAGTAGAATCTATGAACAAAGAATTAAAGAAAATTATAGGACAAATAAGAGATCAGGCTGAACACCTTAAGACAGCAGTACAAATGGCAGTATTCATCCACAATTTTAAAAGAAAAGGGGGGATTGGGGGGTACAGTGCAGGGGAAAGAATAGTAGACATAATAGCATCAGACATACAAACTAGAGCATTACAAAAACAAATTACAAAAATTCAAAATTTTCGGGTTTATTACAGGGACAGCAGAGACCCAATTTGGAAAGGACCAGCAAAGCTACTCTGGAAAGGTGAAGGGGCAGTAGTAATACAAGACAATAGTGAAATAAAAGTAGTACCAAGAAGAAAAGCAAAGATCATTAGGGACTATGGAAAACAGATGGCAGGTGATGATTGTGTGGCAGGTAGACAGGATGAGGAT

>AF076474

TTTTTAGATGGAATAGATAAGGCTCAAGAAGACCATGAAAGATATCACAGCAATTGGAAAGCAATGGCTAGTGATTTTAATCTGCCACCCATAGTAGCAAAAGAAATAGTAGCTAGCTGTGATAAATGTCAGCTAAAAGGGGAAGCAATGCATGGACAAGTAGACTGTAGTCCAGGAATATGGCAATTAGATTGTACACACTTAGAAGGAAAAATTATTCTGGTAGCAGTCCATGTAGCCAGTGGCTATATAGAAGCAGAAGTTATCCCAGCAGAAACAGGACAGGAAACAGCATACTTTATATTAAAATTAGCAGGAAGATGGCCAGTAAAAGTAATACACACAGACAATGGCACCAATTTCACCAGTGCTGCAGTAAAGGCAGCATGTTGGTGGGCAAATGTTACACAAGAATTTGGAATTCCCTACAATCCCCAAAGTCAAGGAGTAGTAGAATCTATGAATAAGGAATTAAAGAAAATCATAGGGCAGGTCAGGGATCAAGCTGAACACCTTAAGACAGCAGTACAGATGGCAGTATTCATTCATAATTTTAAAAGAAAAGGGGGGATTGGGGGGTACAGTGCAGGGGAAAGAATAATAGACATAATAGCATCAGATATACAAACTAAAGAACTACAAAAACAAATTATAAAAATTCAAAATTTTCGGGTTTATTACAGGGACAGCAGAGACCCAATTTGGAAAGGACCAGCAAAACTTCTCTGGAAAGGTGAAGGGGCAGTAGTAATACAAGACAATAGTGAAATAAAGGTAGTACCAAGAAGAAAGGCAAAAATCATTAGGGATTATGGAAAACAGATGGCAGGTGATGATTGTGTGGCAGGTAGACAGGATGAGGAT

>AF076998

TTTTTGGATGGGATAGATAAGGCACAAGAAGAACATGAAAAATACCACAACAATTGGAGAGCAATGGCTAGTGATTTTAATCTGCCACCTGTAGTCGCAAAAGAAATAGTAGCTAGCTGTGATAAGTGTCAGCTAAAAGGGGAAGCCATGCATGGACAAGTAGACTGTAGTCCAGGGATATGGCAACTAGATTGTACACATTTAGAAGGAAAAGTTATCCTGGTAGCAGTCCATGTAGCTAGTGGCTATATAGAAGCAGAAGTCATCCCAGCAGAGACAGGACAGGAAACAGCCTACTTCATACTAAAGTTAGCAGGAAGATGGCCAGTAAAAATGGTACATACAGATAATGGCAGCAATTTCACCAGTGCTGCAGTTAAGGCTGCCTGTTGGTGGGCAGGTATCAAACAGGAATTTGGAATTCCCTACAATCCCCAAAGTCAAGGAGTAGTAGAGTCGATGAATAAAGAGTTAAAGAAAATTATAGGACAGGTAAGAGATCAAGCTGAACATCTTAAGACAGCAGTACAAATGGCAGTATTCATCCACAATTTTAAAAGAAAAGGGGGGATTGGGGGGTACAGTGCAGGGGAAGGAATAATAGACATAATATCAACAGACATACAAACTAAAGAATTACAAAAACAAATTACAAAAATTCAAAATTTCCGGGTTTATTACAGGGACAGCAGAGACCCAGTGTGGAAAGGACCAGCAAAACTACTCTGGAAAGGTGAAGGGGCAGTAGTCATACAAGACAATAGTGAAATAAAGGTAGTACCAAGAAGAAAAGCAAAAATCATTAGGGATTATGGAAAACAGATGGCAGGTGATGATTGTGTGGCAGGTAGACAGGATGAGGAT

>AF075702

TTTTTGGATGGGATAGATAAAGCTCAAGAAGAACATGAAAGGTATCACAGCAATTGGAGAGCAATGGCTAGTGACTTTAATCTGCCACCTGTAATAGCAAAAGAAATAGTAGCCAGCTGTGATAAATGTCAGATAAAAGGGGAAGCCATGCATGGACAAGTAGACTGCAGTCCAGGGATATGGCAATTAGATTGCACGCATTTAGAAGGAAAAGTAATTCTGGTAGCAGTCCATGTAGCCAGTGGCTATATAGAAGCAGAAGTTATCCCAGCAGAAACAGGACAGGAGACAGCATACTTTCTACTAAAATTAGCAGGAAGATGGCCAGTAAAAGTAGTACACACAGACAATGGCAGCAATTTCACCAGTGCTGCATTTAAAGCAGCCTGTTGGTGGGCAAGTGTCCAACAGGAATTTGGAATTCCCTACAATCCCCAAAGTCAAGGAGTAGTGGAATCTATGAATAAGGAATTAAAGAAAATCATAGGGCAGGTAAGAGAGCAAGCTGAACACCTTAAGACAGCAGTACAAATGGCAGTATTCATTCACAATTTTAAAAGAAAAGGGGGGATTGGGGGGTACAGTGTAGGGGAAAGAATAATAGACATAATAGCAACAGACATACAAACTAAAGAATTACAAAAACAAATTACAAAAATTCAAAAATTCCGGGTTTATTACAGGGACAGCAGAAATCCAATTTGGAAAGGACCAGCAAAACTACTCTGGAAAGGTGAAGGGGCAGTGGTAATACAGGACAATAGTGATATAAAGGTAGTACCAAGAAGAAAGGCAAAGATCATTAGGGATTATGGAAAACAGATGGCAGGTGATGATTGTGTGGCAGGTAGACAGGATGAGGAT

>AF193253

TTTTTGGATGGAATAGATAAAGCTCAGGATGAACATGAGAAATATCATAGCAATTGGAGAGCAATGGCTAGTGATTTTAATCTGCCACCTGTAGTAGCAAAAGAAATAGTAGCTAGCTGTGATAAGTGTCAGTTAAAAGGAGAAGCCATGCATGGACAAGTAGACTGTAGTCCAGGAATATGGCAATTAGATTGTACACATTTAGAAGGGAAAGTTATCCTAGTAGCAGTTCATGTAGCCAGTGGCTATATAGAAGCAGAAGTTATTCCAGCAGAGACAGGACAGGACACAGCCTACTTCATATTAAAGCTGGCAGGAAGATGGCCAGTAAAAATGATACATACAGATAATGGCCCCAATTTCACCAGTGGTGCGGTTAAGGCAGCCTGTTGGTGGGCAGGTATCCAGCAAGAATTTGGAATTCCCTACAATCCCCAAAGTCAAGGAGTAGTAGAGTCTATGAATAAAGAGTTAAAGAAAATCATAGGACAGGTAAGAGATCAAGCTGAACATCTTAAGACAGCAGTACAAATGACAGTATTCATCCACAATTTTAAAAGAAAAGGGGGGATTGGGGGGTACGGTGCAGGGGAAAGAATAATAGACATAATAACAACAGACATACAAACTAAAGAATTACAAAAACAAATCATAAAAATTCAAAATTTTCGGGTTTATTACAGGGACAGCAGAGACCCAGTTTGGAAAGGACCAGCAAAGCTACTCTGGAAAGGTGAGGGAGCAGTAGTCATACAAGACAATAGTGAAATAAAGGTAGTACCAAGAAGAAAAGCAAAGATCATTAGGGATTATGGAAAACAGATGGCAGGTGATGATTGTGTGGCAGGTAGACAGGATGAGGAT

>AF190128

TTTCTAGATGGGATAGACAAAGCTCAAGAAGCACATGAAAGGTATCACAACAATTGGAGAGCAATGGCTAGTGAGTTTAATCTGCCACCTATAGTAGCAAAAGAAATAGTAGCTAGCTGTGATAAATGTCAGCTAAAAGGAGAAGCCATGCATGGACAAGTGGACTGTAGCCCAGGAATATGGCAATTAGATTGTACACATTTAGAAGGAAAAGTTATTCTGGTAGCAGTCCACGTAGCCAGTGGCTATATASAACCAGAAGTCATCCCAGCAGAAACAGGACAGGAAACAGCATATTTTATATTGAAACTAGCAGGCAGATGGCCAGTAAAAATGATACATACAGACAATGGCACCAATTTCACAAGTACTGCGGTTAAGGCAGCCTGTTGGTGGGCAGATATCCAACAGGACTTTGGAATTCCCTACAATCCCCAAAGTCAAGGAGTAGTAGAATCTATGAATAAGGAATTAAAAAAGATCATAGGGCAGGTAAGAGACCAAGCTGAACACCTTAGGACAGCAGTACAAATGGCAGTATTCATTCACAATTTTAAAAGAAAAGGGGGGATTGGGGGGTACAGTGCAGGGGAAAGAATAATAGACATAATAGCAACAGACATACAAACTAAAGAATTACAAAAACAAATTTCAAATATTCAAAAATTTCGGGTTTATTACAGGGACAGCAGAGACCCAATTTGGAAAGGACCAGCAAAACTCCTCTGGAAAGGTGAAGGGGCAGTAGTAATACAAGACAATAGTGAAATAAAAGTAGTACCAAGAAGAAAGGCAAAAATCATTAGGGATTATGGAAAACAGATGGCAGGTGATGATTGTGTGGCAGGTAGACAGGATGAGGAT

>AF190127

TTTCTAGATGGGATAGATAAAGCTCAAGTACAGCATGAAAAATATCACAGTAATTGGAGAGCAATGGCCAGTGATTTTAATCTGCCACCTATAGTAGCAAAAGAAATAGTAGCTAGCTGTGATAAATGTCAGTTAAAAGGGGAAGCCATGCATGGACAAGTAGACTGTAGCCCAGGCATATGGCAATTAGATTGTACACACTTAGAAGGAAAAATTATTCTGGTAGCAGTCCATGTAGCCAGTGGCTATATAGAAGCAGAAGTTATCCCAGCAGAAACAGGACAGGAAACAGCATACTTTATATTGAAACTAGCAGGCAGATGGCCAGTAAAAATGATACATACAGACAATGGCAGCAACTTCACAAGTGCTGCGGTTAAGGCAGCCTGTTGGTGGGCAGATATCCACCAGGAATTTGGAATTCCCTACAATCCCCAAAGCCAGGGAGTAGTAGAATCTATGAATAAAGAACTAAAGAAGATCATAGGGCAGGTAAGAGACCAAGCTGAACACCTTAGGACAGCAGTACAAATGGCAGTATTCATTCACAATTTTAAAAGAAAAGGGGGGATTGGGGGGTACAGTGCACGGGAAAGAATAATAGACATAATAGCAACAGACATACCAACTAAAGAACTACAAAAACAAATTTCACAAATTCAAAAATTTCGGGTTTATTACAGGGACAGCAGAGACCCAATTTGGAAAGGACCAGCAAAACTTCTCTGGAAAGGTGAAGGGGCAGTAGTAATACAAGACAATAGTGAAATAAAAGTAGTACCAAGAAGAAAGGCAAAAATCATTAGGGATTATGGAAAACAGATGGCAGGTGATGATTGTGTGGCAGGTAGACAGGATGAGGAT

>AF193277

TTTTTAGATGGAATAGATAAGGCACAAGAAGCACATGAGAAATATCACAGTAATTGGAGAGCAATGGCTAGTGATTTTAACCTGCCACCTGTGGTAGCAAAAGAAATAGTAGCCAGCTGTGATAAATGTCAATTAAAAGGAGAAGCCATGCACGGACAAGTAGACTGTAGTCCAGGAATATGGCAACTAGATTGTACACATTTAGAAGGAAAAATTATCCTAGTAGCAGTTCATGTAGCCAGTGGATATATAGAAGCAGAAGTTATTCCAGCAGAAACAGGACAGGAAACAGCATACTTTGTCTTAAAATTAGCAGGAAGATGGCCAGTAAAAGTAATACATACAGACAATGGCAGCAATTTCATCAGTACTGCGGTTAAGGCTGCCTGTTGGTGGGCAGGGATCAAGCAGGAATTTGGCATTCCCTACAATCCCCAAAGTCAAGGAGTAGTAGAATCTATGAATAAACAATTAAAGCAAATTATAGGACAGGTAAGAGATCAAGCTGAACATCTTAAGACAGCAGTACAAATGGCAGTATTCATCCACAATTTTAAAAGAAAAGGGGGGATTGGGGGGTACAGTGCAGGGGAAAGAATAATAGACATAATAGCAACAGACATACAAACTAAAGAATTACAAAAACAAATTATAAAAATTCAAAATTTTCGGGTTTATTACAGAGACAGCAGAGATCCAATTTGGAAAGGACCAGCAAAACTACTCTGGAAAGGTGAAGGGGCGGTGGTAATACAGGACAATAACGATATAAAAGTAGTACCAAGAAGAAAAGCAAAGATCATTAGGGATTATGGAAAACAGATGGCAGGTGATGATTGTGTGGCAAGTAGACAGGATGAGGAT

>AF193276

TTTTTAGATGGAATAGATAAGGCACAAGAAGAACATGAGAAATATCACGGTAATTGGAGAGCAATGGCTAGTGATTTTAACCTGCCACCTGTGGTAGCAAAAGAAATAGTAGCCAGCTGTGATAAATGTCAATTAAAAGGAGAAGCCATGCACGGACAAGTAGACTGTAGTCCAGGAATATGGCAACTAGATTGTACACATTTAGAAGGAAAAATTATCCTAGTAGCAGTTCATGTAGCCAGTGGATATATAGAAGCAGAAGTTATTCCAGCAGAAACAGGACAGGAAACAGCATACTTTGTCTTAAAATTAGCAGGAAGATGGCCAGTAAAAATAATACATACAGACAATGGCAGCAATTTCACCAGTACTGCGGTTAAGGCTGCCTGTTGGTGGGCAGGGATCAAGCAGGAATTTGGCATTCCCTACAATCCCCAAAGTCAAGGAGTAGTAGAATCTATGAATAAACAATTAAAGCAAACTATAGGACAGGTAAGAGATCAAGCTGAACATCTTAAGACAGCAGTACAAATGGCAGTATTCATCCACAATTTTAAAAGAAAAGGGGGGATTGGGGGGTACAGTGCAGGGGAAAGAATAATAGACATAATAGCAACAGACATACAAACTAAAGAATTACAAAAACAAATTATAAAAATTCAAAATTTTCGGGTTTATTACAGAGACAGCAGAGATCCAATTTGGAAAGGACCAGCAAAACTACTCTGGAAAGGTGAAGGGGCAGTGGTAATACAGGACAATAACGATATAAAAGTAGTACCAAGAAGAAAAGCAAAGATCATTAGGGATTATGGAAAACAGATGGCAGGTGATGATTGTGTGGCAAGTAGACAGGATGAGGAT

>AF192135

TTTCTAGATGGGATAGATAAAGCTCAAGAAGAGCATGAAAAATATCACAGCAAATGGAGAGCAATGGCTAGTGATTTTAATCTACCACCTATAGTAGCAAAAGAAATAGTAGCTAGCTGTGATAAATGTCAGTTAAAAGGGGAAGCCATGCATGGGCAAGTAGACTGTAGTCCAGGGATATGGCAATTAGATTGTACACATTTAGAAGGAAAAGTTATCATAGTAGCAGTCCATGTAGCCAGTGGCTATATGGAAGCAGAGGTTATCCCAGCAGAAACAGGACAGGAAACAGCATACTTTCTGCTAAAATTAGCAGGAAGATGGCCAGTAACAGTAATACACACAGACAATGGTAGCAATTTCACCAGTGCTGCAGTTAAAGCAGCCTGTTGGTGGGCAGGTGTCAGGCAAGAATTTGGGATTCCATACAATCCCCAAAGTCAAGGAGTAGTAGAGTCTATGAATAAGGAATTAAAGAAAATCATAGGGCAGGTTAGGGAACAAGCTGAGCACCTTAAGACAGCAGTACAGATGGCAGTATTCATTCACAATTTTAAAAGAAAAGGGGGGATTGGGGGGTACAGTGCAGGGGAAAGAATAATAGACATGATAGCAACAGACATACAAACTAAAGAACTACAAAAACAAATCATAAAAATTCAAAATTTTCGGGTTTATTACAGGGACAGCAGAGACCCAATTTGGAAAGGACCAGCCAAACTGCTCTGGAAAGGTGAAGGGGCAGTAGTAATACAAGACAATAGTGAAATAAAGGTAGTGCCAAGAAGAAAAGCAAAGATCATTAGAGATTATGGAAAACAGATGGCAGGTGATGATTGTGTGGCAGGTAGACAGGATGAGGAT

>AF115393

TTTTTAGATGGTATAGATTAAGCACAAGAAGAACATGAGAAATATCATAACAATTGGAGAGCTATGGCAAGTGATTTCAATATACCACATATAGTGGCCAAAGAACTAGTAGCCCGTTGTGATAAATGCCAACTAAAAGGAGAGGCCATGCATGGGCAAGTAGACTGCAGCCCAGGCATATGGCAATTAGACTGCACACACCTAGAAGGAAAAGTTATCCTGGTGGCAGTACATGTAGCCAGTGGGTACCTAGAGGCAGAGGTCATTCCTGCAGAGACTGGACAGGAAACAGCTTATTTTATTCTAAAATTAGCAGGAAGATGGCCTGTAAAAGTAATCCACACTGATAATGGGCCTAACTTTACCAGTAATACAGTTAAGGCAGCCTGCTGGTGGGCAGGCATCCAACAGGAATTTGGAATTCCCTATAATCCACAAAGTCAAGGAGTGGTAGAATCAATGAATAAGGAATTAAAAAAGATCATAGGACAAATTAGAGAACAAGCAGAACACTTGAGGACAGCAGTCCAGATGGCAGTGTTCATTCACAATTTTAAAAGAAAAGGGGGGATTGGGGGGTACACTGCAGGAGAAAGAATTATAGACATCATAGCAACAGACATACAAACAACTAATCTACAAAAACAAATTTTAAAAGTTCAAAATTTTCGGGTCTATTACAGGGACAGCAGAGATCCCATTTGGAAAGGACCTGCCAGACTTCTGTGGAAAGGTGAAGGGGCAGTGGTAATTAAAGAGAGAGAGGAAGTTAAAGTAATACCCAGAAGGAAAGCAAAAATAATTAGGGATTATGGAAAACAGATGGCTGGTGATGATAGTATGGCAGGTGGACAGGATGAG

>AB032741

TTTTTAGATGGGATAGATAAGGCTCAAGAAGAACATGAAAGATATCACAGCAATTGGAGAACAATGGCTAGTGATTTTAATTTGCCACCTGTAGTAGCAAAGGAAATAGTAGCCAACTGTGACAAATGTCAACTAAAAGGGGAAGCTATGCATGGACAAGTAGACTGTAGTCCAGGGATATGGCAATTAGATTGCACACATCTAGAAGGAAAAGTCATCCTGGTAGCAGTCCACGTGGCCAGTGGATATATAGAAGCAGAAGTTATCCCAGCAGAAACAGGACAAGAGACAGCATACTTTCTGCTAAAATTAGCAGGAAGATGGCCAGTAAAAGTAATACACACAGACAACGGTAGCAATTTCACCAGCGCTGCAGTTAAAGCAGCCTGTTGGTGGGCCAATGTCCGACAGGAATTTGGGATCCCCTATAATCCCCAAAGTCAAGGAGTAGTAGAATCCATGAATAAGGAATTAAAGAAAATCATAGGGCAGGTAAGAGATCAAGCTGAGCACCTTAAGACAGCAGTACAAATGGCAGTATTCATTCACAATTTTAAAAGAAAAGGGGGGATTGGGGGGTACAGTGCAGGGGAAAGAATAATAGACATAATAGCAACAGACATACAAACTAAAGAATTACAAAAACAAATTACAAAAATTCAAAATTTTCGGGTTTATTACAGGGACAGCAGAGACCCAATTTGGAAAGGACCAGCCAAACTACTCTGGAAAGGTGAAGGGGCAGTAGTAATACAAGACAATAGTGATATAAAAGTAGTACCAAGAAGAAAAGCAAAGATCATTAGGGATTATGGAAAACAGATGGCAGGTGATGATTGTGTGGCAGGTAGACAGGATGAGGAT

>AB032740

TTTTTAGATGGGATAGATAAAGCTCAAGAAGAACATGAAAGATATCACAGCAATTGGAGAACAATGGCTAGTGATTTTAACTTGCCACCTATAGTAGCAAAGGAAATAGTAGCCAACTGTGATAAATGTCAGCTAAAAGGGGAAGCTATGCATGGACAAGTAGACTGCAGTCCAGGGATATGGCAATTAGATTGTACACATCTAGAAGGAAAAGTCATCCTGGTAGCAGTCCACGTGGCCAGTGGATATATAGAAGCAGAAGTTATCCCAGCAGAAACAGGACAGGAGACAGCATACTTTCTGCTAAAATTAGCAGGAAGATGGCCAGTAAAAGTAGTACACACAGACAATGGTAGTAATTTCACCAGCGCTGCAGTTAAAGCAGCCTGTTGGTGGGCCAATGTCCGGCAGGAATTTGGAATCCCCTACAATCCCCAAAGCCAAGGAGTAGTAGAATCTATGAATAAAGAATTAAAGAAAATCATAGGGCAGGTAAGAGAGCAAGCTGAACACCTTAAAACAGCAGTACAAATGGCAGTATTCATTCACAATTTTAAAAGAAAAGGGGGGATTGGGGGGTACAGTGCAGGGGAAAGAATAATAGACATAATAGCAACAGACATACAAACTAAAGAATTACAAAAACAAATTACAAAAATTCAAAATTTTCGGGTTTATTACAGGGACAGCAGAGACCCAATTTGGAAAGGACCAGCAAAACTACTCTGGAAAGGTGAAGGGGCAGTAGTAATACAAGACAATAGTGATATAAAAGTAGTACCAAGAAGAAAAGCAAAGATCATTAGGGATTATGGAAAACAGATGGCAGGTGATGATTGTGTGGCGGGTAGACAGGATGAGGAT

>AJ288982

TTTTTAGATGGCATAGATAAAGCCCAAGAAGATCATGAAAGATATCACAGCAATTGGAGAGCCATGGCTAGTGATTTTAATCTGCCACCTATAGTAGCAAAAGAAATAGTGGCCAGCTGTGACAAATGTCAACTAAAAGGGGAAGCCATGCATGGACAAGTAGACTGTAGTCCAGGGATATGGCAATTAGATTGCACACACCTAGAGGGAAAAATAATCCTGGTAGCAGTTCATGTAGCCAGTGGCTATATAGAAGCAGAAGTAATCCCAGCAGAAACAGGACAGGAGACAGCATACTTTATACTAAAATTAGCAGGAAGATGGCCAGTAAAAGTGATACACACAGACAATGGTAGCAATTTCACCAGTGCTGCAGTTAAAGCAGCCTGTTGGTGGGCAAATATCACACAAGAATTTGGAATTCCCTACAATCCCCAAAGTCAAGGAGTAGTGGAATCTATGAATAAGGAATTAAAGAAAATCATAGGGCAGGTAAGAGAACAAGCTGAGCACCTTAAGACAGCAGTACAAATGGCAGTATTCATTCACAATTTTAAAAGAAAAGGGGGGATTGGGGGGTACAGTGCAGGGGAAAGAATAATAGACATAATAGCATCAGATATACAAACTAAAGAACTACAAAAACAAATTACAAAAATTCAAAATTTTCGGGTTTATTACAGGGACAGCAGAGATCCAATTTGGAAAGGACCAGCAAAACTACTTTGGAAAGGTGAAGGGGCAGTAGTAATACAAGACAATAGTGAAATAAAGGTAGTACCAAGAAGAAAAGCAAAGATCATTAAAGATTATGGAAAACAGATGGCAGGTGATGATTGTGTGGCAGGTAGACAGGATGAGGAT

>AJ288981

TTTTTAGATGGCATAGATAAGGCCCAAGAAGAACATGAAAGATATCACAGCAATTGGAGAGCCATGGCTAATGATTTTAATCTGCCACCTATAGTAGCAAAAGAAATAGTGGCCAGCTGTGACAAATGTCAGCTAAAAGGGGAACCCATGCATGGACAAGTAGACTGTAGCCCAGGGATATGGCAATTAGATTGCACACACCTAGAGGGAAAAATAATCCTGGTAGCAGTCCATGTAGCCAGTGGCTATATAGAAGCAGAAGTTATCCCAGCAGAAACAGGACAGGAGACAGCATACTTTATATTAAAATTAGCAGGAAGATGGCCAGTAAAAGTGATCCACACAGACAATGGTAGCAATTTCACCAGTGCTGCAGTGAAAGCAGCCTGTTGGTGGGCAAATGTCACACAAGAATTTGGAATTCCCTACAATCCCCAAAGTCAAGGAGTAGTGAAATCTATGAATAAGGAGTTAAAGAAAATCATAGGGCAAATAAGAGACCAAGCTGAACACCTTAAGACAGCAGTACAAATGGCAGTATACATTCACAATTTTAAAAGAAAAGGGGGGATTGGGGGGTACAGTGCAGGGGAAAGAATAATAGACATAATAGCATCAGATATCCAAACTAAAGAACTACAAAAACAAATTACAAAAATTCGAAATTTTCGGGTTTATTACAGGGACAGCAGAGACCCAATTTGGAAAGGACCAGCAAAACTACTCTGGAAAGGTGAAGGGGCAGTAGTAATACAAGACAATAGTGAAATAAAAGTAGTACCAAGAAGAAAAGCAAAAATCATTAGAGATTATGGAAAACAGATGGCAGGTGATGATTGTGTGGCAGGTAGACAGGATGAGGAT

>AJ271369

TTCTTAGATGGTATCGATAAGGCACAGGAAGAACATGAAAAGTATCATAACAACTGGAAAGCTATGGCCAGTGATTTCAATCTACCACCTGTAGTGGCCAAAGAAATAGTAGCCAGTTGTGACAAGTGCCAGTTAAAAGGGGAAGCCATGCATGGACAAGTAGACTGTAGTCCAGGCATTTGGCAATTAGACTGTACACATCTAGAAGGAAAAATCATCCTAGTGGCAGTACATGTGGCCAGTGGATACCTAGAGGCAGAGGTCATTCCTGCAGAAACAGGACAAGAAACAGCTTATTTTATTTTAAAATTAGCAGGAAGATGGCCTGTAAAAGTAATACATACTGATAATGGGCCTAACTTTACTAGTAACACAGTCAAAGCAGCCTGTTGGTGGGCTGGCATCCAACAGGAGTTTGGAATCCCCTACAATCCACAAAGTCAAGGAGTAGTGGAATCCATGAATAAAGAATTAAAGAAAATCATAGGACAGATTAGAGACCAAGCAGAGCAGCTAAAGACAGCTGTCCAAATGGCAGTCTTCATTCACAATTTTAAAAGAAAAGGGGGGATTGGGGGGTATACTGCAGGAGAAAGGATTATAGACATCATAGCAACAGACATACAAACAACTAATTTACAAAAACAAATTTTAAAAGTTCAAAATTTTCGGGTTTATTACAGGGACAGCAGAGATCCAATTTGGAAAGGACCTGCCAGACTTCTGTGGAAAGGTGAAGGGGCAGTAGTGATTAAAGAGAACGAGGAAGTTAAAGTAGTACCCAGAAGAAAGGCTAAGATAATCAGAGATTATGGAAAACAGATGGCAGGTGATGGTAGTATGGCAGGTAGACAGAATGAGGAT

>AJ271370

TTCCTAGATGGTATAGAAAAAGCCCAAGAAGAACATGAAAGATATCACAGTAATTGGAAAGCAATGGCCAGTGATTTTAACTTACCCCCCATAGTAGCAAAAGAAATAGTAGCCAGCTGTGACAAATGCCAGCTAAAAGGGGAAGCCATGCATGGGCAGATCAATTGTAGTCCAGGAGTGTGGCAGTTAGATTGTACACACTTAGAAGGAAAAATCATTCTTGTAGCAGTCCATGTGGCCAGTGGCTACTTAGAAGCAGAAGTTATTCCTGCAGAAACAGGACAGGAAACAGCATATTTTATTTTAAAGTTAGCTGGAAGATGGCCAGTAAAAGTTATACACACTGATAATGGACCCAATTTTATTAGTGCCACTGTAAAAGCAGCCTGTTGGTGGGCAGGTATCAAACAGGAATTCGGGATACCCTACAATCCTCAAAGTCAGGGAGCAGTAGAGTCCATGAATAAAGAATTAAAGAAAATTATAGGACAAATCAGGGATCAAGCAGAACATCTAAAAACAGCAGTGCAAATGGCGGTTTTCATTCACAATTTTAAAAGAAAAGGGGGGATTGGGGGGTAMACGGCAGGGGAAAGAATAATAGACATAATAGCAACAGACATACAAACAACAAAATTACAAACACAAATTTTAAAAGTTCAAAATTTTCRGGTTTATTACAGAGACAGCAGAGATCCTATTTGGAAAGGACCAGCCAAACTTCTGTGGAAAGGAGAAGGGGCAGTGGTAATCCAAGATAACGGGGATATAAAGGTAGTCCCACGTAGGAAAGCAAAAATAATTAGAGATTATGGAAAACAGATGGCAGGTGATGGTTGTGTGGCAAGTGGACARGATGAAAAT

>AF208027

TTCTTGRAAAAAATAGAACCTGCTCAAGAAGAACATGAAAARTTTCATAGTAATGTAAAAGAGTTAACTCATAAGTTTGGCATTCCTCAATTAGTAGCAAAACAGATAGTAAATTCATACCACAGTTGCCAACAGAAAGGAGAAGCCATTCATGGACAGGTAAATGCAGAATTGGGTACTTGGCAAATGGACTGCACCCATTTAGAAGGAAAAGTAATCATAGTAGCAGTTCATGTGGCCAGTGGCTTTGTAGAAGCAGAAGTAATCCCACAAGAAACAGGAAGGCAAACAGCATTATTCCTGTTAAAATTAGCAGGGAGATGGCCTATCACACACCTACATACAGACAATGGTGCCAACTTCACCTCGCAAGAGGTGAAGATGGTAGCCTGGTGGGTAGGCATCGAGCAAGCATTTGGAGTACCATACAACCCCCAGAGTCAAGGGGTAGTAGAATCAATGAATCATCATCTAAAGAAACAAATAGATAAAATTAGAGATCAAGCAAATTCAATAGAAACCATAGTGTTAATGGCAGTACATTGCATGAATTTTAAAAGAAGGGGAGGAATAGGGGATATGACCCCTGCAGAAAGAATAATTAACATGATTACTACAGAACAAGAAATACAATTTCAACAAACAAAAAATTCAAAATTTAAAAATTTTCGGGTCTATTACAGAGAAGGCAGAGACCAGCTCTGGAAAGGACCTGGTGAGCTTTTGTGGAAAGGGGAAGGAGCTGTCATCATAAAGGTAGGGACAGAAATCAAAGTTGTACCCAGAAGAAAAGCAAAAATCATAAAAGATTATGG~AGGAGGAAAAGAGGTGAGGACTGCCTATAGAATAAGCAGAAAGAGGAC

>AJ237565

TTTTTAGATGGGATAGATAAGGCTCAAGAAGCACATGAAAAATATCACAGCAATTGGAGAGCAATGGCTAGTGATTTTAATCTACCACCTATAGTAGCAAAGGAAATAGTAGCCAGCTGTGATAAATGTCAACTAAAAGGAGAAGCCATACATGGACAAGTAGACTGCAGTCCAGGAATATGGCAATTAGATTGCACACATCTAGAAGGAAAAATAATCCTAGTAGCAGTCCATGTAGCTAGTGGCTATATAGAAGCAGAAGTTATCCCAGCAGAAACAGGACAGGAAACAGCATACTTTATACTAAAATTAGCAGGAAGATGGCCAGTAAAAGTAATACACACAGACAATGGTAGCAATTTCATCAGTGCTGCAGTCAAAGCAGCCTGTTGGTGGGCAGATATTAAACAGGAATTTGGAATTCCCTACAATCCCCAAAGTCAAGGAGTAGTGGAATCTATGAACAAGGAATTAAAGAAAATCATAGGGCAGGTAAGAGAGCAAGCTGAACACCTTAAGACAGCARTACAAATGGCAGTATTCATTCACAATTTTAAAAGAAAAGGGGGGATTGGGGGGTACAGTGCGGGGGAAAGAATAATAGACATRATAGCAACAGACATACAAACTAAAGAACTACAAAAACAAATTATAAAAATTCAAAATTTTCGGGTTTATTACAGGGACAGCAGAGACCCAATTTGGAAAGGACCAGCAAAACTACTCTGGAAAGGTGAAGGGGCAGTAGTAATACAGGATAATGGTGATATAAAGGTAGTACCAAGAAGAAAAGCAAAGATCATTAGGGATTATGGAAAACAGATGGCAGGTGATGATTGTGTGGCAGGTAGACAGGATGAGGAT

>AF110959

TTTCTAGATGGAATAGATAAGGCTCAAGAAGAGCATGAAAAGTATCACAACAATTGGAGAGCAATGGCTAGTGAATTTAATCTACCACCCATAGTAGCAAAAGAAATAGTAGCTAGCTGTGATAAATGTCAGCTAAAAGGGGAAGCCATGCATGGACAAGTAGACTGTGGTCCAGGGATATGGCAATTAGATTGTACACATCTAGAAGGAAAAGTCATCCTGGTAGCAGTCCATGTAGCCAGTGGCTACATGGAAGCAGAGGTTATCCCAGCAGAAACAGGGCAGGAAACAGCATACTATATACTAAAATTAGCAGGAAGATGGCCAGTCAAAGTAATACATACAGACAATGGTACTAATTTCACCAGTGCTGCAGTTAAGGCAGCCTGTTGGTGGGCAGGTATCCAACAGGAATTTGGAATTCCCTACAATCCCCAAAGTCAGGGAGTAGTAGAATCTATGAATAAAGAATTAAAGAAGATTATAGGGCAGGTAAGAGAGCAAGCTGAGCACCTTAAGACAGCAGTACAAATGGCAGTATTCATTCACAATTTTAAAAGAAGAGGGGGGATTGGGGGGTATAGTGCAGGGGAAAGAATAATAGACATAATAGCAACAGACATACAAACTAAAGAATTACAAAAACAAATTATGAAAATTCAAAATTTTCGGGTTTATTACAGAGACAGCAGAGACCCTATTTGGAAAGGACCAGCCAAACTACTCTGGAAAGGTGAAGGGGCAGTAGTAATACAAGATAATAGTGACATAAAGGTAGTACCGAGGAGGAAAGTAAAAATCATTAAGGACTATGGAAAACAGATGGCAGGTGCTGATTGTGTGGCAGGTAGACAGGATGAGGAT

>AF110960

TTTCTAGATGGAATAGATAAGGCTCAAGAAGAGCATGAAAAGTATCACAACAATTGGAGAGCAATGGCTAGTGAATTTAATCTACCACCCATAGTAGCAAAAGAAATAGTAGCTAGCTGTGATAAATGTCAGCTAAAAGGGGAAGCCATGCATGGACAAGTAGACTGTAGTCCAGGGATATGGCAATTAGATTGTACACATCTAGAAGGAAAAGTCATCCTGGTAGCAGTCCATGTAGCCAGTGGCTACATGGAAGCAGAGGTTATCCCAGCAGAAACAGGACAGGAAACAGCATACTATATACTAAAATTAGCAGGAAGATGGCCAGTCAAAGTAATACATACAGACAATGGTACTAATTTCACCAGTGCTGCAGTTAAGGCAGCCTGTTGGTGGGCAGGTATCCAACAGGAATTTGGAATTCCCTACAATCCCCAAAGTCAGGGAGTAGTAGAATCTATGAATAAAGAATTAAAGAAGATTATAGGGCAGGTAAGAGAGCAAGCTGAGCACCTTAAGACAGCAGTACAAATGGCAGTATTCATTCACAATTTTAAAAGAAGAGGGGGGATTGGGGGGTATAGTGCAGGGGAAAGAATAATAGACATAATAGCAACAGACATACGAACTAAAGAATTACAAAAACAAATTATGAAAATTCGAAATTTTCGGGTTTATTACAGAGACAGCAGAGACCCTATTTGGAGAGGACCAGCCAAACTACTCTGGAAAGGTGAAGGGGCAGTAGTAATACAAGATAATAGTGACATAAAGGTAGTACCGAGGAGGAAAGTAAAAATCATTAAGGACTATGGAAAACAGATGGCAGGTGCTGATTGTGTGGCAGGTAGACAGGATGAGGAT

>AF110961

TTTCTAGATGGAATAGATAAGGCTCAAGAAGAGCATGAAAAGTATCACAACAATTGGAGAGCAATGGCTAGTGAATTTAATCTACCACCCATAGTAGCAAAAGAAATAGTAGCTAGCTGTGATAAATGTCAGCTAAAAGGGGAAGCCATGCATGGACAAGTAGACTGTAGTCCAGGGATATGGCAATTAGATTGTACACATCTAGAAGGAAAAGTCATCCTGGCAGCAGTCCATGTAGCCAGTGGCTACATGGAAGCAGAGGTTATCCCAGCAGAAACAGGACAGGAAACAGCATACTATATACTAAAATTAGCAGGAAGATGGCCAGTCAAAGTAATACATACAGACAATGGTACTAATTTCACCAGTGCTGCAGTTAAGGCAGCCTGTTGGTGGGCAGGTATCCAACAGGAATTTGGAATTCCCTACAATCCCCAAAGTCAGGGAGTAGTAGAATCTATGAATAAAGAATTAAAGAAGATTATAGGGCAGGTAAGAGAGCAAGCTGAGCACCTTAAGACAGCAGTACAAATGGCAGTATTCATTCACAATTTTAAAAGAAGAGGGGGGATTGGGGGGTATAGTGCAGGGGAAAGAATAATAGACATAATAGCAACAGACATACAAACTAAAGAATTACAAAAACAAATTATGAAAATTCAAAATTTTCGGGTTTATTACAGAGACAGCAGAGACCCTATTTGGAAAGGACCAGCCAAACTACTCTGGAAAGGTGAAGGGGCAGTAGTAATACAAGATAATAGTGACATAAAGGTAGTACCGAGGAGGAAAGTAAAAATCATTAAGGACTATGGAAAACAGATGGCAGGTGCTGATTGTGTGGCAGGTAGACAGGATGAGGAT

>AF110963

TTTCTAGATGGAATAGATAAGGCTCAAGAAGAGCATGAAAAGTATCACTGCAATTGGAGAGCAATGGCTAGTGATTTTAATCTGCCACCAGTAGTAGCAAAAGAAATAGTAGCTAGCTGTGATAAATGTCAACTAAAAGGGGAAGCCATGCATGGACAAGTAGACTGCAGTCCAGGGATCTGGCAATTAGATTGTACACATTTAGAAGGCAAGATCATCCTGGTAGCAGTCCATGTAGCCAGTGGCTACATAGAAGCAGAGGTTATCCCAGCAGAAACAGGACAAGAAACAGCATACTATATACTAAAATTAGCAGGAAGATGGCCAGTCAAAGTAATACACACAGACAATGGCAGTAATTTCACCAGTACTGCAGTTAAGGCAGCCTGTTGGTGGGCAGGTATACAACAGGAATTTGGGATTCCCTACAATCCCCAAAGTCAGGGAGTAGTAGAATCCATGAATAAAGAATTAGAGAAAATCATAGGGCAGGTAAGAGAACAAGCTGAGCACCTTAAGACAGCAGTACAAATGGCAGTATTCATTCACAATTTTAAAAGAAAAGGGAGGATTGGGGGGTACAGTGCAGGGGAGAGAATAATAGACATAATAGCAACAGACATACAAACTAAAGAATTACAAAAACAAATTATAAAAATTCAAAACTTTCGGGTTTATTACAGAGACAGCCGAGACCCTGTTTGGAAGGGACCAGCCAAACTACTCTGGAAAGGTGAAGGGGCAGTAGTAATACAAGACAATAGTGACATAAAGGTAGTACCAAGGAGGAAAGTTAAAATCATTAGGGACTATGGAAAACAGATGGCAGGTGATGATTGTGTGGCAGGTAGACAGGATGAAGAT

>AF110964

TTTCTAGATGGAATAGATAAGGCTCAAGAAGAGCATGAAAAGTATCACTGCAATTGGAGAGCAATGGCTAGTGATTTTAATCTGCCACCCGTAGTAGCAAAAGAAATAGTGGCTAGCTGTGATAAATGTCAACTAAAAGGGGAAGCCATACATGGACAAGTAGACTGTAGTCCAGGGATCTGGCAATTAGATTGTACACATTTAGAAGGAAAGATCATCCTGGTAGCAGTCCATGTAGCCAGTGGTTACATAGAGGCAGAGGTTATCCCAGCAGAAACAGGACAAGAAACAGCATACTATATACTAAAATTAGCAGGAAGATGGCCAGTCAAAGTAATACACACAGACAATGGCAGTAATTTCACCAGTGCTGCAGTTAAGGCAGCCTGTTGGTGGGCAGGTATACAACAGGAATTTGGGATTCCCCACAATCCCCAAAGTCAGGGAGTAGTAGAATCCATGAATAAAGAATTAAAGAAAATCATAGGACAGGTAAGAGAACAAGCTGAGCACCTTAAGACAGCAGTACAAATGGCAGTATTCATTCACAATTTTAAAAGAAAAGGGGGGATTGGGGGGTACAGTGCAGGGGAGAGAATAATAGACATAATAGCAACAGACATACAAACTAAAGAATTACAAAAACAAATTATAAAAATTCAAAACTTTCGGGTTTATTACAGAGACAGCCGAGACCCTGTTTGGAAGGGACCAGCCAAACTACTCTGGAAAGGTGAAGGGGCAGTAGTAATACAAGATAATAGTGACATAAAGGTAGTACCAAGGAGGAAAGTAAAAATCATTAGGGACTATGGAAAACAGATGGCAGGTGATGATTGTGTGGCAGGTAGACAGGATGAAGAT

>AF110965

TTTCTAGATGGAATAGATAAGGCTCAAGAAGAGCATGAAAAGTATCACTGCAATTGGAGAGCAATGGCTAGTGATTTTAATCTGCCACCAGTAGTAGCAAAAGAAATAGTAGCTAGCTGTGATAAATGTCAACTAAAAGGGGAAGCCATGCATGGACAAGTAGACTGCAGTCCAGGGATCTGGCAATTAGATTGTACACATTTAGAAGGCAAGATCATCCTGGTAGCAGTCCATGTAGCCAGTGGCTACATAGAAGCAGAGGTTATCCCAGCAGAAACAGGACAAGAAACAGCATACTATATACTAAAATTAGCAGGAAGTTGGCCAGTCAAAGTAATACACACAGACAATGGCAGTAATTTCACCAGTACTGCAGTTAAGGCAGCCTGTTGGTGGGCAGGTATACAACAGGAATTTGGGATTCCCTACAATCCCCAAAGTCAGGGAGTAGTAGAATCCATGAATAAAGAATTAAAGAAAATCATAGGGCAGGTAAGAGAACAAGCTGAGCACCTTAAGACAGCAGTACAAATGGCAGTATTCATTCACAATTTTAAAAGAAAAGGGAGGATTGGGGGGTACAGTGCAGGGGAGAGAATAATAGACATAATAGCAACAGACATACAAACTAAAGAATTACAAAAACAAATTATAAAAATTCAAAACTTCCGGGTTTATTACAGAGACAGCCGAGACCCTGTTTGGAAGGGACCAGCCAAACTACTCTGGAAAGGTGAAGGGGCAGTAGTAATACAAGACAATAGTGACATAAAGGTAGTACCAAGGAGGAAAGTAAAAATCATTAGGGACTATGGAAAACAGATGGCAGGTGATGATTGTGTGGCAGGTAGACAGGATGAAGAT

>AF110962

TTTCTAGATGGAATAGATAAGGCTCAAGAAGAGCATGAAAAGTATCACTGCAATTGGAGAGCAATGGCTAGTGAGTTTAATCTGCCACCCATAGTAGCAAAAGAAATAGTAGCTAGCTGTGATAAATGTCAACTAAAAGGGGAAGCCATACATGGACAAGTAGACTGTAGTCCAGGGATCTGGCAATTAGATTGTACACATTTAGAAGGAAAGATCATCCTGGTAGCAGTCCATGTAGCCAGTGGCTACATAGAAGCAGAGGTTATCCCAGCAGAAACAGGACAAGAAACAGCATACTATATACTAAAATTAGCAGGAAGATGGCCAGTCAAAGTAATACACACAGACAATGGCAGTAATTTCACCAGTACTGCAGTTAAGGCAGCCTGTTGGTGGGCAGGTATACAACAGGAATTTGGAATTCCCTACAATCCCCAAAGTCAGGGAGTAGTAGAATCCATGAATAAAGAATTAAAGAAAATCATAGGGCAGGTAAGAGAACAAGCTGAGCACCTTAAGACAGCAGTACAAATGGCAGTATTCATTCACAATTTTAAAAGAAAAGGGGGGATTGGGGGGTACAGTGCAGGGGAGAGAATAATAGACATAATAGCAACAGACATACAAACTAAAGAATTACAAAAACAAATTATAAAAATTCAAAACTTTCGGGTTTATTACAGAGACAGCCGAGACCCTGTTTGGAAGGGACCAGCCAAACTACTCTGGAAAGGTGAAGGGGCAGTAGTAATACAAGATAATAGTGACATAAAGGTAGTACCAAGGAGGAAAGTAAAAATCATTAGGGACTATGGAAAACAGATGGCAGGTGCTGATTGTGTGGCAGGTAGACAGGATGAAGAT

>AF110966

TTTCTAGATGGAATAGATAAGGCTCAAGAAGAGCATGAAAAGTATCACTGCAATTGGAGAGCAATGGCTAGTGATTTTAATCTGCCACCCGTAGTAGCAAAAGAAATAGTAGCTAGCTGTGATAAATGTCAACTAAAAGGGGAAGCCATGCATGGACAAGTAGACTGTAGTCCAGGGATCTGGCAATTAGATTGTACACACTTAGAAGGGAAGATCATCCTGGTAGCAGTCCATGTAGCCAGTGGTTACATAGAGGCAGAGGTTATCCCAGCAGAAACAGGACAAGAAACAGCATACTATATACTAAAATTAGCAGGAAGATGGCCAGTCAAAGTAATACACACAGACAATGGCAGTAATTTCACCAGTACTGCAGTTAAGGCAGCCTGTTGGTGGGCAGGTATACAACAGGAATTTGGGATTCCCTACAATCCCCAAAGTCAGGGAGTAGTAGAATCCATGAATAAAGAATTAAAGAAAATCATAGGGCAGGTAAGAGAACAAGCTGAGCACCTTAAGACAGCAGTACAAATGGCAGTATTCATTCACAATTTTAAAAGAAAAGGGGGGATTGGGGGGTACAGTGCAGGGGAGAGAATAATAGACATAATAGCAACAGACATACAAACTAAAGAATTACAAAAACAAATTATAAAAATTCAAAACTTCCGGGTTTATTACAGAGACAGCCGAGACCCTGTTTGGAAGGGACCAGCCAAACTACTCTGGAAAGGTGAAGGGGCAGTAGTAATACAAGATAATAGTGACATAAAGGTAGTACCAAGGAGGAAAGTAAAAATCATTAGGGACTATGGAAAACAGATGGCAGGTGATGATTGTGTGGCAGGTAGACAGGATGAAGAT

>AF110972

TTTCTAGATGGAATAGATAAAGCTCAAGAAGAGCATGAAAAATATCACAACAATTGGAGAGCAATGGCTAGTGATTTTAATCTGCCACCCATAGTAGCAAAAGAAATAGTAGCTAGCTGTGATAAATGCCAGTTAAAAGGGGAAGCCATGCATGGACAAGTAGACTGTAGTCCAGGAATATGGCAATTAGATTGTACACATTTAGAAGGGAAAATCATCCTGGTAGCAGTCCGTGTAGCCAGTGGCTACATAGAAGCAGAGGTTATTCCAGCAGAAACAGGACAAGAAACAGCATACTTTATACTAAAATTAGCAGGAAGATGGCCAGTCAAAGTAATACACACAGACAATGGCAGTAATTTTACCAGCAATGCAGTTAAGGCAGCCTGTTGGTGGGCAGGTACCCAACAGGAATTTGGAATTCCCTACAATCCCCAAAGTCAGGGAGTAGTAGAATCCATGAATAAAGAATTAAAGAAAATCATAGGGCAAGTAAGAGATCAAGCTGAGCACCTTAAGACAGCAGTACAAATGGCAGTATTCATTCACAATTTTAAAAGAAAAGGGGGGATTGGGGGGTACAGTGCAGGGGAAAGAATAATAGACATAATAGCAACAGATATACAAACTACAGAACTACAAAAACAAATTATAAAAATTCAAAATTTTCGGGTTTATTACAGAGACAGCAGAGACCCTATTTGGAAAGGACCAGCCAAACTACTCTGGAAAGGTGAAGGGGCAGTAGTAATACAAGATAATAGTGACATAAAAGTAGTACCAAGGAGAAAGGTAAAAATCATTAAGGACTATGGAAAACAGATGGCAGGGGCTGATTGTGTGGCAGGTAGACAGGATGAGGAT

>AF071474

TTTTTAGATGGAATAGATAAGGCTCAAGAAGAACATGAAAGATATCACAGCAATTGGAGAGCAATGGCTAGTGATTTTAATCTGCCACCTATAGTAGCAAAGGAAATAGTAGCCAGCTGTGATAAATGTCAACTAAAAGGGGAAGCCATGCATGGACAAGTAGATTGTAGCCCAGGGATATGGCAACTAGATTGCACACATCTAGAAGGAAAAGTAATACTGGTAGCAGTCCATGTAGCCAGTGGCTATATAGAAGCAGAAGTTATCCCAGCAGAAACAGGACAGGAGACAGCATACTTTCTGCTAAAATTAGCAGGAAGATGGCCAGTAAGAAGAGTACACACAGACAATGGCAGCAATTTCACCAGCGCTGCAGTTAAAGCAGCCTGTTGGTGGGCAAATATCCAACAGGAATTTGGAATTCCCTACAATCCCCAAAGTCAGGGAGTAGTAGAATCCATGAATAAAGAATTAAAGAAAATCATAGGACAGGTAAGAGATCAAGCTGAGCACCTTAAGACAGCAGTACAAATGGCAGTATTCATTCACAATTTTAAAAGAAAAGGGGGGATTGGGGGGTACAGTCCAGGGGAAAGAATAATAGACATAATAGCAACAGATATACAAACTAAAGAATTACAAAAACAAATTACAAAAATTCAAAATTTTCGGGTTTATTACAGGGACAGCAGAGATCCAATTTGGAAAGGACCAGCAAAACTACTCTGGAAAGGTGAAGGGGCAGTAGTAATACAGGACAATAGTGATATAAAGGTAGTACCAAGAAGAAAAGTAAAGATCATTAGGGATTATGGAAAACAGATGGCAGGTGATGATTGTGTGGCAGGTAGACAGGATGAGGAT

>AF071473

TTTTTAGATGGGATAGATAAAGCTCAAGAAGAACATGAAAGATATCACAGCAATTGGAGAGCAATGGCTAGTGATTTTAATCTGCCACCTATAGTAGCAAAGGAAATAGTAGCCAGCTGTGATAAATGTCAGCTAAAAGGGGAAGCCATGCATGGACAAGTAGACTGCAGTCCAGGGATATGGCAATTAGATTGCACACATCTAGAAGGAAAAGTAATTCTGGTAGCAGTTCATGTAGCTAGTGGCTATATAGAAGCAGAAGTTATCCCAGCAGAAACAGGACAAGAGACAGCATACTTTATACTAAAATTAGCAGGAAGATGGCCAGTAAAAGTAATACACACAGACAATGGCAGCAATTTCACCAGCGCTGCAGTTAAAGCAGCCTGTTGGTGGGCAGGTATCCAACAGGAATTTGGGATTCCCTACAATCCCCAAAGTCAAGGAGTAGTGGAATCTATGAATAAAGAATTAAAGAAAATCATAGGACAAGTAAGAGAGCAAGCTGAACACCTTAAAACAGCAGTACAAATGGCAGTATTCATTCACAATTTTAAAAGAAAAGGGGGGATTGGGGGGTACAGTGCAGGGGAAAGAATAATAGACATAATAGCAACAGACATACAAACTAAAGAATTACAAAAACACATTACAAAAATTCAAAATTTTCGGGTTTATTACAGGGACAGCAGAGATCCACTTTGGAAAGGACCAGCAAAACTACTCTGGAAAGGTGAAGGGGCAGTAGTAATACAGGACAATAGTGATATAAAGGTAGTACCAAGAAGAAAAGCAAAGATCATTAGGGATTATGGAAAACAGATGGCAGGTGATGATTGTGTGGCAGGTAGACAGGATGAGGAT

>AJ286133

TTTTTAGATGGCATAGATAAAGCCCAAGAAGAGCATGAAAGATATCACAGCAATTGGAGAGCAATGGCTAGTGATTTTAATCTGCCACCTATAGTAGCAAAAGAAATAGTGGCCAGCTGTGATAAATGCCAGCTAAAAGGGGAAGCCATGCATGGACAAGTAGACTGTAGTCCAGGAATATGGCAATTAGATTGTACACACTTAGAAGGAAAAATTATCATGGTAGCAGTCCATGTAGCCAGTGGCTATATAGAAGCAGAAGTAATCCCAGCAGAAACAGGACAGGAGACAGCATACTTTATATTAAAATTAGCAGGAAGATGGCCAGTGAAAGTAATACACACAGACAATGGCAGTAATTTCACCAGTGCTGCAGTAAAGGCAGCATGTTGGTGGGCAAATGTCACACAAGAATTTGGAATTCCCTACAATCCCCAAAGCCAAGGAGTAGTGGAATCTATGAATAAAGAATTAAAGAAAATTATAGGGCAGGTCAGGGATCAAGCTGAACACCTTAAGACAGCAGTACAGATGGCAGTATTCATTCACAATTTTAAAAGAAAAGGGGGGATTGGGGGGTACAGTGCAGGGGAAAGAATAATAGACATAATAGCATCAGATATACAAACTAAAGAACTACAAAAACAAATTACAAAAATTCAAAATTTTCGGGTTTATTACAGGGACAGCAGAGATCCAATTTGGAAAGGACCAGCAAAACTACTCTGGAAAGGTGAAGGGGCAGTAGTAATACAGGACAATAGTGATATAAAGGTAGTACCAAGAAGAAAAGTAAAAATCATTAGAGATTATGGAAAACAGATGGCAGGTGATGATTGTGTGGCAAGTAGACAGGATGAGGAT

>AF197340

TTTTTAGATGGGATAGATAAAGCTCAAGAAGAACATGAAAGATATCACAGTAATTGGAGAACAATGGCTAGTGATTTTAATTTGCCACCTATAGTAGCGAAAGAAATAGTAGCCAACTGTGATAAATGTCAACTAAAAGGAGAAGCTATGCATGGACAAGTGGACTGTAGTCCAGGGATATGGCAATTAGATTGCACACATTTAGAAGGAAAAGTCATCCTGGTAGCAGTCCACGTGGCCAGTGGATATATAGAAGCAGAAGTTATCCCAGCAGAAACAGGACAGGAGACAGCATACTTTCTGCTAAAATTAGCAGGAAGATAGCCAGTAAAGGTCATACACACAGACAATGGTAGCAATTTCACCAGCGCTGCTATGAAAGCAGCCTGTTGGTGGGCCAATGTCCAACAGGAATTTGGGATTCCCTACAATCCCCAAAGCCAAGGAGTAGTAGAATCTATGAATAAGGAATTAAAGAAAGTCATAGGGCAGGTAAGAGAGCAAGCTGAACACCTTAAGACAGCAGTACAAATGGCAGTATTCATTCACAATTTTAAAAGAAAAGGGGGGATTGGGGGGTACAGTGCAGGGGAAAGAATAATAGACATAATAGCAACAGAAATACAAACTAAAGAAYTACAAAAACAAATTACAAAAATTCAAAATTTTCGGGTTTATTACAGGGACAGCAGAGACCCAATTTGGAAAGGACCAGCAAAACTACTCTGGAAAGGTGAAGGGGCAGTAGTAATACAAGACAATAGTGATATAAAAGTAGTACCAAGAAGAAAAGCAAAGATTATTAGGGATTATGGAAAACAGATGGCAGGTGATGATTGTGTGGCAGGTAGACAGGATGAGAAT

>AF197341

TTTTTAGATGGGATAGATAAGGCCCAAGAAGAACATGAAAGATATCACAGCAATTGGAGAACAATGGCTAGTGATTTTAATTTGCCACCTATAGTAGCAAAGGAAATAGTAGCCAACTGTGATAAATGTCAACTAAAAGGGGAAGCTATGCATGGACAAGTGGACTGTAGTCCAGGGATATGGCAACTAGATTGCACACATCTAGAAGGAAAAGTCATCCTGGTAGCAGTCCACGTGGCCAGTGGATATATAGAAGCAGAAGTTATCCCAGCAGAAACAGGACAGGAGACAGCATACTTTCTGCTAAAATTAGCAGGAAGATGGCCAGTAAAGGTAATACACACAGACAATGGTAGCAATTTCACCAGTGCTACAGTTAAAGCAGCCTGTTGGTGGGCCAATGTCCAACAGGAATTTGGGATTCCCTACAATCCCCAAAGTCAAGGAGTAGTGGAATCTATGAATAAGGAATTAAAGAAAATCATAGGGCAGGTAAGAGAACAAGCTGAACACCTCAAGACAGCAGTACAAATGGCAGTATTCATTCACAATTTTAAAAGAAAAGGGGGGATTGGGGGGTACAGTGCAGGGGAAAGAATAATAGACATAATAGCAACAGACATACAAACTAAAGAACTACAAAAACATATTACAAAAATTCAAAATTTTCGGGTTTATTACAGGGACAGCAGAGACCCAATTTGGAAAGGACCAGCAAAGCTACTCTGGAAAGGTGAAGGGGCAGTAGTAATACAAGACAATAGTGATATAAAAGTAGTACCAAGAAGAAAAGCAAAGATCATTAGGGATTATGGAAAACAGATGGCAGGTGATGATTGTGTGGCAGGTAGACAGGATGAGGAT

>AF197338

TTTTTAGATGGGATAGATAAGGCTCAAGAAGAACATGAAAGATATCACAGCAATTGGAGAACAATGGCTAGTGATTTTAATTTGCCACCTATAGTAGCAAAGGAAATAGTAGCCAACTGTGATAAATGTCAACTAAAAGGGGAAGCTATGCATGGACAAGTAGACTGCAGTCCAGGGATATGGCAATTAGATTGCACACATCTAGAAGGAAAAGTCATCCTGGTAGCAGTCCACGTGGCCAGTGGATATATAGAAGCAGAAGTTATCCCAGCAGAAACAGGACAGGAGACAGCATACTTTCTGCTAAAATTAGCAGGAAGATGGCCAGTAAAAGTAATACACACAGACAACGGTAGCAATTTCACCAGCGCTGCTGTTAAAGCAGCCTGTTGGTGGGCCAATGTCCGACAGGAATTTGGGATCCCCTACAATCCCCAAGGTCAAGGAGTAGTAGAATCTATGAATAAGGAATTAAAGGAAATCATAGGGCAGGTAAGAGAGCAAGCTGAACACCTTAAGACAGCAGTACAAATGGCAGTATTCATTCACAATTTTAAAAGAAAAGGGGGGATTGGGGGGTACAGTGCAGGAGAAAGAATAATAGACATAATAGCAACAGACATACAAACTAAAGAATTACAAAAACAAATTACAAAAATTCAAAATTTTCGGGTTTATTACAGGGACAGCAGAGACCCAATTTGGAAAGGACCAGCAAAACTACTCTGGAAAGGTGAAGGGGCAGTAGTAATACAAGACAATAGTGATATAAAAGTAGTCCCAAGAAGAAAAGCAAAGATCATTAGGGATTATGGAAAACAGATGGCAGGTGATGATTGTGTGGCAGGTAGACAGGATGAGGAT

>AF184155

TTTTTAGATGGCTTAGATAAAGCCCAAGAAGAGCATGAGAAATTTCACAGCAATTGGAGAGCAATGGCTAGTGATTTTAATCTGCCACCTATAGTAGCAAAAGAAATAGTAGCCAGCTGTGATAAATGTCAGCTAAAAGGGGAAGCCATGCATGGACAAGTAGATTGTAGTCCAGGAATATGGCAATTAGATTGTACACATTTAGAAGGAAAAATTATCCTGGTAGCAGTCCATGTAGCCAGTGGCTATATAGAGGCAGAAGTTATCCCAGCAGAAACAGGACAGGAGACAGCATACTTTATATTAAAATTAGCAGGAAGATGGCCAGTGAAAATAATACACACAGACAATGGCAGCAATTTCACCAGTGCTGCAGTAAGGGCAGCATGTTGGTGGGCAAATGTCACACAAGAATTTGGAATTCCCTACAATCCCCAAAGCCAAGGAGTAGTGGAATCTATGAATAAAGAATTAAAGAAAATTATAGGGCAGGTCAGGGATCAAGCTGAACACCTTAAGACAGCAGTACAGATGGCAGTATTCATTCACAATTTTAAAAGAAAAGGGGGGATTGGGGGGTACAGTGCAGGGGAAAGAATAATAGATATAATAGCATCAGATATACAAACTAAAGAACTACAAAAACAGATTACAAAAATTCAAAATTTTCGGGTCTATTACAGGGACAGCAGAGACCCCATTTGGAAAGGACCAGCAAAGCTACTCTGGAAAGGTGAAGGGGCAGTAGTAATACAGGACAATAGTGATATAAAGGTAGTACCAAGAAGAAAAGCAAAAATCCTTAGGGATTATGGAAAACAGATGGCAGGTGATGATTGTGTGGCAGGTAGACAGGATGAGGAT

>AF289550

TTTCTAGATGGAATAGATAAGGCTCAAGAAGAGCATGAAAAATATCACAACAATTGGAGAGCAATGGCTAGTGATTTTAACCTGCCACCTGTGGTAGCAAAAGAAATAGTAGCTAGCTGTGATAAATGTCAGCTAAAAGGAGAAGCATTGCATGGACAAGTAGACTGTAGTCCAGGAATATGGCAATTAGATTGTACACATTTAGAAGGAAAAGTTATCCTGGTAGCAGTCCATGTGGCCAGTGGCTATATAGAAGCAGAAGTTATTCCAGCAGAAACAGGACAGGAAACAGCCTACTTTCTTTTGAAACTAGCAGGAAGATGGCCAGTAAAAGTAGTACATACAGACAATGGCAGCAATTTCACCAGCGCTGCAGTTAAGGCTGCCTGTTGGTGGGCAGGCATCAAGCAGGAATTTGGAATTCCCTACAATCCCCAAAGTCAGGGAGTAGTAGAATCCATGAATAAGGAATTAAAGAAAATCATAGGACAGGTGAGAGATCAAGCTGAACATCTTAAGACAGCAGTACAAATGGCAGTATTCATCCACAATTTTAAAAGAAAAGGGGGGATTGGGGGGTACAGTGCAGGGGAAAGAATAATAGACATAATAGCAACAGATATACAAACTAAAGAACTACAAAAACAAATTATAAAAATTCAAAATTTTCGGGTTTATTACAGAGACAGCAGAGACCCTATTTGGAAAGGACCAGCCAAACTACTCTGGAAAGGTGAAGGGGCAGTAGTAATACAAGATAACAGTGAAATAAAGGTAGTACCAAGGAGGAAAGTAAAAATCATTAAGGACTATGGAAAACAGATGGCAGGTGCTGATTGTGTGGCAAGTAGACAGGATGAGGAT

>AF289549

TTTCTAGATGGAATAGATAAAGCTCAAGAAGAGCATGAGAAATACCACAGCAATTGGAGAGCAATGGCTAGTGATTTTAACCTGCCACCTGTGGTAGCAAAAGAGATAGTAGCTAGCTGTGATAAATGTCAGCTAAAGGGAGAAGCCTTGCATGGACAAGTAGACTGTAGTCCAGGAATATGGCAATTAGATTGTACACATTTAGAAGGAAAAGTTATCCTGGTAGCAGTCCATGTAGCCAGTGGCTATATAGAAGCAGAAGTAATTCCAGCAGAAACAGGGCAGGAAACAGCCTACTTTCTCTTGAAATTAGCAGGAAGATGGCCAGTAAAAGTAGTACATACAGACAATGGTAGCAATTTCACCAGCGCCGCAGTTAAGGCCGCCTGTTGGTGGGCAGGCATCAAGCAGGAATTTGGAATTCCCTACAATCCCCAAAGTCAGGGAGTAGTAGAATCCATGAATAAAGAATTAAAGAAAATCATAGGACAGGTAAGAGATCAAGCTGAACATCTTAAAACAGCAGTACAAATGGCAGTATTCATTCACAATTTTAAAAGAAAAGGGGGGATTGGGGGGTACAGTGCAGGGGAAAGAATAATAGACATAATAGCAACAGACATACAAAGTAAAGAACTACAAAAACAAATTATAAAAATTCAAAATTTTCGGGTTTATTACAGAGACAGCAGAGACCCGATTTGGAAAGGACCAGCAAAGCTTCTCTGGAAAGGTGAAGGGGCAGTAGTAATACAAGACAATAGTGACATAAAGGTAGTACCAAGGAGGAAAGTAAAAATCATTAAGGACTATGGAAAACAGATGGCAGGTGCTGATTGTGTGGCAAGTAGACAGGATGAGGAT

>AF289548

TTTCTAGATGGAATAGATAAAGCTCAAGAAGAGCATGAGAAATACCACAACAATTGGAGAGCAATGGCTAGTGATTTTAACCTGCCACCTGTGGTAGCAAAAGAAATAGTAGCTAGCTGTGATAAATGTCAGCTAAAGGGAGAAGCCTTGCATGGACAAGTAGACTGTAGCCCAGGAATATGGCAATTAGATTGTACACATTTAGAAGGGAAAGTTATCCTGGTAGCAGTCCATGTAGCCAGTGGCTATATAGAAGCAGAAGTTATTCCAGCAGAAACAGGGCAGGAAACAGCCTATTTCCTCTTGAAATTAGCAGGAAGATGGCCAGTAAAAGTAGTACATACAGACAATGGCAGCAATTTCACCAGCGCTGCAGTTAAGGCCGCCTGTTGGTGGGCAGGCATCAAGCAGGAATTTGGAATTCCCTACAATCCCCAAAGTCAGGGAGTAGTAGAATCCATGAATAAAGAACTAAAGAAAATCATAGGACAGGTAAGAGATCAAGCTGAACAGCTTAAGACAGCAGTACAAATGGCAGTATTCATTCACAATTTTAAAAGAAAAGGGGGGATTGGGGGGTACAGTGCAGGGGAAAGAATAATAGACATAATAGCAACAGACATACAAACTAAAGAACTACAAAAACAAATTATAAAAATTCAAAATTTTCGGGTTTATTACAGAGACAGCAGAGGCCCTATTTGGAAAGGACCAGCCAAACTACTCTGGAAAGGTGAAGGGGCAGTAGTAATACAAGAAAACAGTGACATAAAGGTAGTACCAAGGAGGAAAGTAAAAATCATTAAGGACTATGGAAAACAGATGGCAGGTGCTGATTGTGTGGCAAGTAGACAGGATGAGGAT

>AJ276595

TTTTTAGATGGCATAGATAAAGCCCAAGAAGAGCACGAAAGATACCACAGCAATTGGAGAGCTATGGCTAGTGATTTTAATCTGCCACCTGTAATAGCAAAAGAAATAGTGGCCAGCTGTGATAAGTGCCAGCTAAAAGGGGAAGCCATGCATGGACAGGTAGACTGTAGTCCAGGAATATGGCAAATAGATTGTACACATTTAGAAGGAAAAGTTATCATAGTAGCAGTCCATGTAGCCAGTGGCTATATGGAAGCAGAAGTTATCCCAGCAGAAACAGGACAGGAGACAGCGTACTTTCTATTAAAATTAGCAGGAAGATGGCCAGTGAGAGTAATACACACAGACAATGGCAGCAATTTCACCAGTGCTGCAGTAAAGGCAGCATGTTGGTGGGCAAATGTCACACAAGAATTTGGAATTCCCTACAATCCCCAAAGCCAAGGAGTAGTGGAATCTATGAATAAAGAATTAAAGAAAATTATAGGGCAGGTTAGGGATCAAGCTGAACACCTTAAGACAGCAGTACAGATGGCAGTATTCATTCACAATTTTAAAAGAAAAGGGGGGATTGGGGGGTACAGTGCACGGGAAAGAATAATAGACATAATAGCATCAGATATACAAACTAAAGAACTACAAAAACCAATTACAAAAATTCAAAATTTTCGGGTCTATTACAGGGACAGCAGAGACCCAATTTGGAAAGGACCGGCAAAACTACTCTGGAAAGGTGAAGGGGCAGTAGTAATACAGGACAATAGTGATATAAAAGTAGTACCAAGGAGAAAAGCAAAAATCATTAGGGATTATGGAAAACAGATGGCAGGTGATGGTTGTGTGGCAGGTAGACAGGATGAGGAT

>AY008718

TTTTTAGATGGGATAGATAAGGCTCAAGAAGAACATGAAAGATATCACAGCAATTGGAGAACAATGGCTAGTGATTTTAATTTGCCACCTATAGTAGCAAAGGAGATAGTAGCCAACTGTGATAAATGTCAACTAAAAGGGGAAGCTATGCATGGACAAGTGGACTGTAGTCCAGGAATATGGCAATTAGATTGTACACATCTAGAAGGAAAAGTCATCCTGGTAGCAGTCCACGTGGCCAGTGGATATATAGAAGCAGAAGTTATCCCAGCAGAAACAGGACAGGAGACAGCATACTTTCTGCTAAAATTAGCAGGAAGATGGCCAGTAAAAGTAATACACACAGACAACGGTAGCAATTTCACCAGCGCTGCAGTTAAAGCAGCCTGTTGGTGGGCCAATGTCCGACAGGAATTTGGGATCCCCTACAATCCCCAAAGTCAAGGAGTAGTAGAATCTATGAATAAAGAATTAAAGAAAATCATAGGGCAGGTAAGAGAGCAAGCTGAATACCTTAAGACAGCAGTACAAATGGCAGTATTCATTCACAATTTTAAAAGAAAAGGGGGGATTGGGGGGTACAGTGCAGGGGAAAGAATAATAGACATAATAGCAACAGACATACAAACTAAAGAATTACAAAAACAAATTACAAAAATTCAAAATTTTCGGGTTTATTACAGGGACAGCAGAGACCCAATTTGGAAAGGACCAGCAAAACTACTCTGGAAAGGTGAAGGGGCAGTAGTGATACAAGACAATAGTGATATAAAAGTAGTACCAAGAAGAAAAGCAAAGATCATTAGGGATTATGGAAAACAGATGGCAGGTGATGATTGTGTGGCAGGTAGACAGGATGAGGAT

>AY008717

TTTCTAGATGGAATAGATAAAGCTCAAGAAGAGCATGAAAAGTATCACAGCAATTGGAGAGCAATGGCTAGTGACTTTAATCTGCCACCCATAGTAGCAAAAGAAATAGTAGCTAGCTGTGATCAATGTCAGCTAAAAGGAGAAGCCATGCATGGACAAGTAGACTGTAGTCCAGGGATATGGCAATTAGATTGTACACATTTAGAAGGAAAAATCATTCTGGTAGCAGTCCATGTAGCCAGTGGCTACATAGAAGCAGAGGTTATCCCAGCAGAAACAGGACAAGAAACAGCATACTTTATACTAAAATTAGCAGGAAGATGGCCAGTCAAAGTAATACATACAGACAATGGTAGTAATTTCACCAGTGCTGCAGTTAAGGCAGCCTGTTGGTGGGCAGGTATCCAACAGGAATTTGGAATTCCCTACAATCCCCAAAGTCAGGGAGTAGTAGAATCCATGAATAAGGAATTAAAGAAACTTATAGGGCAGGTAAGAGATCAAGCTGAGCACCTTAAGACAGCAGTACAAATGGCAGTATTCATTCACAATTTTAAAAGAAAAGGGGGGATTGGGGGGTACAGTGCAGGGGAAAGAATAGTAGACATAATAGCAACAGACATACAAACTAGAGAATTACAAAAACAAATTATAAAAATTCAAAATTTTCGGGTTTATTACAGAGACAGCAGAGACCCCATTTGGAAAGGACCAGCCAAACTACTCTGGAAAGGTGAAGGGGCAGTAGTAATACAAGATAATAGTGACATAAAGGTAGTACCAAGGAGGAAAGCAAAAATCATTAAGGACTATGGAAAACAGATGGCAGGTGCTGATTGTGTGGCAGGTAGACAGGATGAAGAT

>AY008716

TTTCTAGATGGAATAGATAAAGCTCAAGAAGAGCATGAAAAGTATCACAGCAATTGGAGAGCAATGGCTAGTGACTTTAATCTGCCACCCATAGTAGCAAAAGAAATAGTAGCTAGCTGTGATCAATGTCAGCTAAAAGGAGAAGCCATGCATGGACAAGTAGACTGTAGTCCAGGGATATGGCAATTAGATTGTACACATTTAGAAGGAAAAATCATTCTGGTAGCAGTCCATGTAGCCAGTGGCTACATAGAAGCAGAGGTTATCCCAGCAGAAACAGGACAAGAAACAGCATACTTTATACTAAAATTAGCAGGAAGATGGCCAGTCAAAGTAATACATACAGACAATGGTAGTAATTTCACCAGTGCTGCAGTTAAGGCAGCCTGTTGGTGGGCAGGTATCCAACAGGAATTTGGAATTCCCTACAATCCCCAAAGTCAGGGAGTAGTAGAATCCATGAATAAGGAATTAAAGAAACTTATAGGGCAGGTAAGAGATCAAGCTGAGCACCTTAAGACAGCAGTACAAATGGCAGTATTCATTCACAATTTTAAAAGAAAAGGGGGGATTGGGGGGTACAGTGCAGGGGAAAGAATAGTAGACATAATAGCAACAGACATACAAACTAGAGAATTACAAAAACAAATTATAAAAATTCAAAATTTTCGGGTTTATTACAGAGACAGCAGAGACCCCATTTGGAAAGGACCAGCCAAACTACTCTGGAAAGGTGAAGGGGCAGTAGTAATACAAGATAATAGTGACATAAAGGTAGTACCAAGGAGGAAAGCAAAAATCATTAAGGACTATGGAAAACAGATGGCAGGTGCTGATTGTGTGGCAGGTAGACAGGATGAAGAT

>AY008715

TTTCTAGATGGAATAGATAAAGCTCAAGAAGAGCATGAAAAGTATCACAGCAATTGGAGAGCAATGGCTAGTGACTTTAATCTGCCACCCATAGTAGCAAAAGAAATAGTAGCTAGCTGTGATCAATGTCAGCTAAAAGGAGAAGCCATGCATGGACAAGTAGACTGTAGTCCAGGGATATGGCAATTAGATTGTACACATTTAGAAGGAAAAATCATTCTGGTAGCAGTCCATGTAGCCAGTGGCTACATAGAAGCAGAGGTTATCCCAGCAGAAACAGGACAAGAAACAGCATACTTTATACTAAAATTAGCAGGAAGATGGCCAGTCAAAGTAATACATACAGACAATGGTAGTAATTTCACCAGTGCTGCAGTTAAGGCAGCCTGTTGGTGGGCAGGTATCCAACAGGAATTTGGAATTCCCTACAATCCCCAAAGTCAGGGAGTAGTAGAATCCATGAATAAGGAATTAAAGAAACTTATAGGGCAGGTAAGAGATCAAGCTGAGCACCTTAAGACAGCAGTACAAATGGCAGTATTCATTCACAATTTTAAAAGAAAAGGGGGGATTGGGGGGTACAGTGCAGGGGAAAGAATAGTAGACATAATAGCAACAGACATACAAACTAGAGAATTACAAAAACAAATTATAAAAATTCAAAATTTTCGGGTTTATTACAGAGACAGCAGAGACCCCATTTGGAAAGGACCAGCCAAACTACTCTGGAAAGGTGAAGGGGCAGTAGTAATACAAGATAATAGTGACATAAAGGTAGTACCAAGGAGGAAAGCAAAAATCATTAAGGACTATGGAAAACAGATGGCAGGTGCTGATTGTGTGGCAGGTAGACAGGATGAAGAT

>AY008714

TTTTTAGATGGGATAGATAAGGCTCAAGAAGAACATGAAAGATATCACAGCAATTGGAGAACAATGGCTAGTGATTTTAATTTGCCACCTATAGTAGCAAAGGAAATAGTAGCCAACTGTGATAAATGTCAACTAAAAGGGGAAGCTATGCATGGACAAGTGGACTGTAGTCCAGGAATATGGCAATTAGATTGTACACATCTAGAAGGAAAAGTCATCCTGGTAGCAGTCCACGTGGCCAGTGGGTATATAGAAGCAGAAGTTATCCCAGCAGAAACAGGACAGGAGACAGCATACTTTCTGCTAAAATTAGCAGGAAGATGGCCAGTAAAAGTAATACACACAGACAACGGTAGCAATTTCACCAGCGCTGCAGTTAAAGCAGCCTGTTGGTGGGCCAATGTCCGACAGGAGTTTGGGATCCCCTACAATCCCCAAAGTCAAGGAGTAGTAGAATCTATGAATAAAGAATTAAAGAAAATCATAGGGCAGGTAAGAGAGCAAGCTGAACACCTTAAGACAGCAGTACAAATGGCAGTATTCATTCACAATTTTAAAAGAAAAGGGGGGATTGGGGGGTACAGTGCAGGGGAAAGAATAATAGACATAATAGCAACAGACATACAAACTAAAGAATTACAAAAACAAATTACAAAAATTCAAAATTTTCGGGTTTATTACAGGGACAGCAGAGACCCAATTTGGAAAGGACCAGCAAAACTACTCTGGAAAGGTGAAGGGGCAGTAGTGATACAAGACAATAGTGATATAAAAGTAGTACCAAGAAGAAAAGCAAAGATCATTAGGGATTATGGAAAACAGATGGCAGGTGATGATTGTGTGGCAGGTAGACAGGATGAGGAT

>AF286229

TTTCTAGATGGAATAGATAAAGCTCAAGAAGAGCATGAAAAGTATCACAGCAATTGGAGAGCAATGGCTAGTGACTTTAATCTGCCACCCATAGTAGCAAAAGAAATAGTAGCTAGCTGTGATCAATGTCAGCTAAAAGGGGAAGCCATGCATGGACAAGTAGACTGTAGTCCAGGGATATGGCAATTAGATTGTACACATTTAGAAGGAAAAATCATTCTGGTAGCAGTCCATGTAGCCAGTGGCTACATGGAAGCAGAGGTTATCCCAGCAGAGACAGGACAAGAAACAGCATACTTTATACTAAAATTAGCAGGAAGATGGCCAGTCAAAGTAATACATACAGACAATGGTAGTAATTTCACCAGTACTGCAGTTAAGGCAGCCTGTTGGTGGGCAGGTATCCAACAGGAATTTGGAATTCCCTACAATCCCCAAAGTCAGGGAGTAGTAGAATCCATGAATAAAGAATTAAAGAAACTTATCGGGCAGGTAAGAGATCAAGCTGAACACCTTAAGACAGCAGTACAAATGGCAGTATTCATTCACAATTTTAAAAGAAAAGGGGGGATTGGGGGGTACAGTGCAGGGGAAAGAATAGTAGACATAATAGCAACAGACATACAAACTAGAGAATTACAAAAACAAATTATAAAAATTCAAAATTTTCGGGTTTATTACAGAGACAGCAGAGACCCCATTTGGAAAGGACCAGCCAAACTACTCTGGAAAGGTGAAGGGGCAGTAGTAATACAAGATAATAGTGACATAAAGGTAGTACCAAGGAGGAAAGCAAAAATCATTAAGGACTATGGAAAACAGATGGCAGGTGCTGATTGTGTGGCAGGTAGACAGGATGAAGAT

>AF286226

TTTCTAGATGGAATAGATAAAGCTCAAGAAGAGCATGAAAAGTATCACAGCAATTGGAGAGCAATGGCTAGTGACTTTAATCTGCCACCCATAGTAGCAAAAGAAATAGTGGCTAGCTGTGATCAATGTCAGCTAAAAGGAGAAGCCATGCATGGACAAGTAGACTGTAGTCCAGGGATATGGCAATTAGATTGTACACATTTAGAAGGAAAAATCATCCTGGTAGCAGTCCATGTAGCCAGTGGCTACATGGAAGCAGAGGTTATCCCAGCAGAAACAGGACAAGAGACAGCATACTTTATACTAAAATTAGCAGGTAGATGGCCAGTCAAAGTAATACATACAGACAATGGTAGTAATTTCACCAGTACTGCAGTTAAGGCAGCCTGTTGGTGGGCAGGTATCCAACAGGAATTTGGAATTCCCTACAATCCCCAAAGTCAGGGAGTAGTAGAAGCCATGAATAAAGAATTAAAGAAAATTATAGGGCAGGTAAGAGATCAAGCTGAGCACCTTAAGACAGCAGTACAAATGGCAGTATTCATTCACAATTTTAAAAGAAAAGGGGGGATTGGGGGGTACAGTGCAGGGGAAAGAATAATAGATATAATAGCAACAGACATACAAACTAAAGAATTACAAAAACAGATTACAAAAATTCAAAATTTTCGGGTTTATTACAGAGACAGCAGAGACCCCAGTTGGAAAGGACCAGCCAAACTACTCTGGAAAGGTGAAGGGGCAGTAGTAATACAAGATAATAGTGACATAAAGGTAGTACCAAGGAGGAAAGCAAAAATCATTAAGGACTATGGAAAACAGATGGCAGGTGCTGATTGTGTGGCAGGTAGACAGGATGAAGAT

>AF286230

TTTCTAGATGGAATAGATAAAGCTCAAGAAGAGCATGAAAGGTATCACAGCAATTGGAGAGCAATGGCTAGTGACTTTAATCTGCCACCCGTAGTAGCAAAAGAAATAGTGGCTAGCTGTGATCAATGTCAGCTAAAAGGAGAAGCCATGCATGGACAAGTAGACTGTAGTCCAGGGATATGGCAATTAGATTGTACACATTTAGAAGGAAAAATCATCCTGGTAGCAGTCCATGTAGCCAGTGGCTACATGGAAGCAGAGGTTATCCCAGCAGAAACAGGACAAGAGACAGCATACTTTATACTAAAATTAGCAGGAAGATGGCCAGTCAAAGTAATACATACAGACAATGGTAGTAATTTCACCAGTACTGCAGTTAAGGCAGCCTGTTGGTGGGCAGGTATCCAACAGGAATTTGGAATTCCCTACAATCCCCAAAGTCAGGGAGTAGTAGAATCCATGAATAAAGAATTAAAGAAAATTATAGGGCAGGTAAGAGATCAAGCTGAGCACCTTAAGACAGCAGTACAAATGGCAGTATTCATTCACAATTTTAAAAGAAAAGGGGGGATTGGGGGGTACAGTGCAGGGGAAAGAATAATAGATATAATAGCAACAGACATACAAACTAAAGAATTACAAAAACAGATTACAAAAATTCAAAATTTTCGGGTTTATTACAGAGACAGCAGAGACCCCAGCTGGAAAGGACCAGCCAAACTACTCTGGAAAGGTGAAGGGGCAGTAGTAATACAAGATAATAGTGACATAAAGGTAGTACCAAGGAGGAAAGCAAAAATCATTAAGGACTATGGAAAACAGATGGCAGGTGCTGATTGTGTGGCAGGTAGACAGGATGAAGAT

>AF286231

TTTTTAGATGGAATAGATAAGGCTCAAGAAGAGCATGAAAAGTATCACAGCAATTGGAGAGCAATGGCTAGTGACTTTAATCTGCCACCCGTAGTGGCAAAAGAAATAGTAGCTAGCTGTAATCAATGTCAGCAAAAAGGGGAAGCTATGCATGGACAAGTAGACTGTAGTCCAGGGATATGGCAATTAGATTGTACACATCTAGAAGGAAAAATCATCCTGGTAGCAGTCCATGTAGCCAGTGGCTACATAGAAGCAGAGGTTATCCCAGCAGAAACAGGACAAGAAACAGCATACTATACACTAAGGTTAGCAGGAAGATGGCCAGTTAAAGTAATACATACAGACAATGGTAGTAATTTCATCAGTAATGCAGTTAAGGCAGCCTGTTGGTGGGCAGGTATCCAACAGGAATTTGGAATTCCCTACAATCCCCAAAGTCAGGGAGTAGTAGAATCCATGAATAAAGAATTAAAGAAAATTATAGGGCAGGTAAGAGATCAAGCTGAGCACCTTAAGACAGCAGTACAAATGGCAGTATTCATTCACAATTTTAAAAGAAGAGGGGGGATTGGGGGGTACAGTGCAGGGGAAAGAATAATGGACATAATAGCAACAGACATACAAACTAAAGAATTACAAAAACAAATTTTAAAAGTTCAAAATTTTCGGGTTTATTACAGAGACAGCAGAGACCCTATTTGGAAAGGACCAGCCAAACTACTCTGGAAAGGTGAAGGGGCAGTAGTAATACAAGATAATAGTGACATAAAGGTAGTACCAAGAAGGAAAGCAAAAATCATTAAGGATTATGGAAAACAGATGGCAGGTGCTGATTGTGTGGCAGGTAGACAGGATGAAGAT

>AF286232

TTTCTAGATGGAATAGATAAAGCCCAAGAAGAGCATGAAAGGTATCATAGCAATTGGAGAGCAATGGCTAGTGACTTTAATCTGCCACCCATAGTAGCAAAAGAAATAGTAGCTAGCTGTGACCAATGTCAGCTAAAAGGGGAAGCCATGCATGGACAAGTGGACTGTAGTCCAGGAATATGGCAATTAGATTGTACACACTTAGAAGGAAAAATCATCCTGGTAGCAGTACATGTAGCCAGTGGCTACATAGAAGCAGAGGTTATCCCAGCAGAAACAGGACAAGAAACAGCATACTTTATACTAAAATTAGCAGGAAGATGGCCAGTCAAAGTAATACATACAGACAATGGAAGTAATTTCACCAGTGCTGCAGTTAAAGCAGCCTGTTGGTGGGCAGGGGTCCAACAGGAATTTGGAATTCCCTACAATCCCCAAAGTCAGGGAGTAGTAGAATCCATGAATAAAGAATTAAAGAAAATTATAGGGCAGGTAAGAGATCAAGCTGAGCACCTTAAGACAGCAGTACAAATGGCAGTATTCATTCACAATTTTAAAAGAAAAGGGGGGATTGGGGGGTACAGTGCAGGGGAAAGAATAATAGACATAATAGCAACAGACATACAAACTAAAGAATTACAAAAACAAATTATAAAAATTCAAAATTTTCGGGTTTATTACAGAGACAGCAGAGACCCCATTTGGAAAGGCCCAGCCAAACTACTCTGGAAAGGTGAAGGGGCAGTAGTAATCCAAGATAATAGTGACATAAAGGTAATACCAAGGAGGAAAGCAAAAATCATTAAGGACTATGGAAAACAGATGGCAGGTGCTGATTGTGTGGCAGGTAGACAGGATGAAGAT

>AF286223

TTTCTAGATGGGATAGATAAGGCTCAAGAAGATCATGAAAAGTATCACAGCAATTGGAGAGCAATGGCTAATGAGTTTAATCTGCCACCCATAGTAGCAAAAGAAATAGTAGCTAGCTGTGATAAATGCCAGCTAAAAGGGGAAGCCATGCATGGACAAGTAGACCGTAGCCCAGGGATATGGCAATTAGATTGTACACATCTAGAAGGAAAAATCATCCTGGTAGCAGTCCATGTAGCCAGTGGCTACATAGAAGCAGAGGTTATCCCAGCAGAAACAGGACAAGAAACAGCATACTATATACTAAAATTAGCAGGAAGATGGCCAGTCAAAGTAATACATACAGACAATGGTAGTAATTTCACCAGTGCTGCAGTTAAGGCAGCCTGTTGGTGGGCAGGTATCCAACAGGAATTTGGAATTCCCTACAATCCCCAAAGCCAGGGAGTAGTAGAATCCATGAATAAAGAATTAAAGAAAATTATAGGGCAGGTAAGAGAACAAGCTGAGCACCTTAAGACAGCAGTACAAATGGCAGTATTCATTCACAATTTTAAAAGAAAAGGGGGGATTGGGGGGTACAGTGCAGGGGAAAGAACAATAGACATAATAGCAACAGACATACAAACTAAAGAATTACAAAACCAAATTACAAAAATTCAAAATTTTCGGGTTTATTACAGAGACAGCAGAGACCCCATTTGGAAAGGACCAGCCAAACTGCTCTGGAAAGGTGAAGGGGCAGTAGTAATACAAGATAATAGTGACATAAAGGTAGTGCCAAGGAGGAAAGCAAAAATTATTAGGGATTATGGAAAACAGATGGCAGGTGCTGATTGTGTGGCAGGTAGACAGGATGAGGAT

>AF286233

TTTCTAGATGGAATAGATAAGGCTCAAGAAGATCATGAAAAATATCACAGCAATTGGAGAGCAATGGCTAATGAGTTTAATCTGCCACCCATAGTAGCAAAAGAAATAGTAGCTAGCTGTGATAAATGTCAGCTAAAAGGGGAAGCCATGCATGGACAAGTAGACTGTAGTCCAGGGATATGGCAATTAGATTGTACACATTTAGAAGGGAAGGTCATCCTGGTAGCAGTCCATGTAGCCAGTGGCTACATAGAAGCAGAGGTTATCCCAGCAGAAACAGGACAGGAAACAGCATACTATATACTAAAATTAGCAGGAAGATGGCCAGTCAAAGTAATACATACAGATAATGGCCCTAATTTCACCAGTGCTGCAGTTAAAGCAGCCTGTTGGTGGGCAGGTATCCAACAGGAATTTGGAATTCCCTACAATCCCCAAAGTCAGGGAGTAGTAGAATCCATGAATAAGGAATTAAAGAAAATCATAGGGCAGGTAAGAGAACAAGCTGAGCACCTTAAGACAGCAGTACAAATGGCAGTATTCATTCACAATTTTAAAAGAAAAGGGGGGATTGGGGGGTACAGTGCAGGGGAAAGAATAATAGATATAATAGCAACAAATATACAAACTACAGAATTACAAAAACAAATTACAAAAATTCAAAATTTTCGGGTTTATTACAGAGACAGCAGAGACCCTATTTGGAAAGGACCAGCCAAACTACTCTGGAAAGGTGAAGGGGCAGTAGTAATACAAGATAACAGTGACATTAAGGTAGTACCAAGGAGAAAAGCAAAGATCATTAGGGATTATGGAAAACAGATGGCAGGTGATGATTGTGTGGCAAGTAGACAGGATGAAGAT

>AF286234

TTTCTAGATGGAATAGACAAGGCTCAAGAAGAGCATGAAAAGTATCACAGCAATTGGAGAGCAATGGCTAGTGAGTTTAATCTGCCACCCATAGTAGCAAAAGAAATAGTAGCAAGCTGTGATAAATGTCAGCTAAAAGGGGAAGCCATACATGGACAAGTAGACTGTAGTCCAGGGATATGGCAATTAGATTGTACACATTTAGAAGGAAAAATCATCCTGGTAGCAGTCCATGTAGCCAGTGGCTACATAGAAGCAGAGGTTATCCCAGCAGAAACAGGACAAGAAACAGCATAATACATATTAAAATTAGCAGGAAGATGGCCAGTCAAAGTAATACATACAGACAATGGTAGTAATTTTACCAGCAGTGCAGTCAAAGCAGCCTGTTGGTGGGCAGGTATCCAACAGGAATTTGGAATTCCCTACAATCCCCAAAGTCAGGGAGTAGTAGAATCCATGAATAAAGAATTAAAGAAAATCATAGGGCAGGTAAGAGATCAAGCTGAGCACCTTAAGACAGCAGTACAAATGGCAGTACTCATTCACAATTTTAAAAGAAAAGGGGGGATTGGGGGGTACAGTGCAGGGGAAGGAATAATAGACATAATAGCAACAGACATACAAACTAAAGAATTACAAAAACAAATTATAAAAATTCAAAATTTTCGGGTTTATTACAGAGACAGCAGAGACCCTATTTGGAAAGGACCAGCCAAACTACTCTGGAAAGGTGAAGGGGCAGTAGTAATACAAGATAACAGTGACATAAAGGTAGTGCCAAGGAGAAAAGCAAAAATCATCAGGGACTATGGAAAACAGATGGCAGGTGCTGATTGTGTGGCAAGTAGACAGGATGAAGAT

>AF286235

TTTCTAGATGGAATAGATAAGGCTCAAGAAGAACATGAAAAATATCACAACAATTGGAGAGCAATGGCTAGTGATTTTAATATACCACCCGTAGTAGCAAAAGAAATAGTAGCCAGCTGTGATAAATGTCAACTAAAAGGGGAAGCCATACATGGGCAAGTAGACTGTAGTCCAGGGATATGGCAATTAGACTGTACACATTTAGAAGGAAAAATCATCCTGGTAACAGTCCATGTAGCCAGTGGCTACATGGAAGCAGAAGTTATCCCAGCAGAAACAGGACAGGAAACAGCATACTTTATATTAAAATTAGCAGGAAGATGGCCAGTCAAAGTAATACACACAGATAATGGTAGTAATTTCACTAGTGCTGCAGTTAAGGCAGCCTGTTGGTGGGCAGGTATCCAACAGGAATTTGGAATTCCCTACAATCCCCAAAGTCAGGGAGTAGTAGAATCCATGAATAAAGAATTAAAAAAGATCATAGGGCAGGTAAGAGATCAAGCTGAGCACCTTAAGACAGCAGTACAAATGGCAGTACTCATTCACAATTTTAAAAGAAAAGGGGGGATTGGGGGGTACAGTGCAGGGGAAAGAATAATAGACATAATAGCAACAGACATACAAACTAAAGAATTACAAAAACAAATTATAAAAATTCAAAATTTTCGGGTTTATTACAGAGACAGCAGAGACCCTATTTGGAAAGGACCAGCCAAGCTACTCTGGAAAGGTGAAGGGGCGGTAGTAATACAAGATAATAGTGACATAAAGATAGTACCAAGGCGGAAAGCAAAAATCATTAAGGACTATGGAAAACAGATGGCAGGTGCTGATTGTGTGGCAGGTAGACAGGATGAAGAT

>AF286227

TTTCTAGACGGAATAGATAAGGCTCAAGAAGATCATGAAAAGTATCACAGCAATTGGAGAGCAATGGCTAGTGAATTTAATCTACCACCCATAGTAGCAAAAGAAATAATAGCCAGCTGTGATAAATGTCAGCTGAAAGGGGAAGCCATACATGGACAAGTGGACTGTAGTCCAGGGGTATGGCAATTAGATTGTACACATTTAGAAGGAAAAGTCATCCTGGTAGCAGTCCATGTAGCCAGTGGCTACATGGAAGCAGAGGTTATCCCAGGAGAAACAGGACAAGAAACAGCATACTATATACTAAAATTAGCAGGAAGATGGCCAGTCAAAGTAATACATACAGACAATGGCAGTAATTTCACTAGTGCTGCAGTTAAGGCAGCCTGTTGGTGGGCTGGTATCCAACAGGAATTTGGGATTCCCTACAATCCCCAAAGTCAGGGAGTAGTAGAATCCATGAATAAAGAATTAAAGAAAATCGTAGGGCAGGTAAGAGATCAAGCTGAGCACCTTAAGACAGCAGTACAAATGGCAGTATTCATTCACAATTTTAAAAGAAAAGGGGGGATTGGGGGGTACAGTGCAGGGGAAAGAATGATAGACATAATAGCAACAGACATACAAACTAAAGAATTACAAAAACAAATTATAAAAATTCAAAATTTTCGGGTTTATTACAGAGACAGCAGAGACCCTATTTGGAAAGGACCAGCCAAGCTACTCTGGAAAGGTGAAGGGGCAGTAGTAATACAAGATAACAGTGACATAAAGGTAGTACCAAGGAGGAAAGCAAAAATCATTAAGGATTATGGAAAACAGATGGCAGGTGCTGATTGTGTGGCAGGTAGACAGGATGAGGAT

>AF286224

TTTCTAGATGGAATAGACAAGGCTCAAGAAGAGCATGAAAAATATCACAACAATTGGAGAGCAATGGCTAGTGAATTTAATCTACCACCAGTAGTAGCAAAAGAAATAGTAGCTAGTTGTGATAAATGTCAGCAAAAAGGGGAAGCCACACATGGACAAGTAGACTGTAGTCCAGGGATATGGCAATTAGACTGTACACATTTAGAAGGAAAAATCATCCTGGTAGCAGTCCATGTAGCCAGTGGCTACATAGAAGCAGAGGTTATCCCAGCAGAAACAGGACAAGAAACAGCATACTATATATTAAAATTAGCAGGAAGATGGCCAGTCAAAGTAATACATACAGACAATGGTAGCAATTTTACCAGTGCTGCAGTTAAGGCAGCCTGTTGGTGGGCAGGTATCAAACAAGAATTTGGAATTCCCTACAATCCACAAAGTCAGGGAGTAGTAGAATCCATGAATAAAGAATTAAAGAAAATCATAGGGCAGGTAAGAGATCAGGCTGAGCATCTTAAAACAGCAGTACAAATGGCAGTATTCATTCACAATTTTAAAAGAAAAAGGGGGATTGGGGGGTACAGTGCAGGGGAAAGAATAATAGACATAATAGCAACAGACATACAAACCAAAGAACTACAAAAACAAATTATAAACATTCAAAAATTTCGGGTTTATTACAGAGACAGCAGAGACCCCATTTGGAAAGGACCAGCCAAACTACTCTGGAAAGGTGAAGGGGCAGTAGTAATACAAGATAATAGTGACATAAAAGTGGTACCAAGGAGGAAAGCAAAAATCATTAGGGACTATGGAAAACAGATGGCAGGCGCTGATTGTGTGGCAGGTAGACAGGATGAGGAT

>AF286225

TTTCTAGATGGAATAGATAAGGCTCAAGAAGAGCATGAAAAATATCACAGCAATTGGAGAGCAATGGCTAGTGAGTTTAATCTGCCACCCATAGTAGCAAAAGAAATAGTAGCCAGCTGTGATAAATGTCAGCTAAAAGGGGAAGCCATACATGGACAAGTAGACTGTAGTCCAGGAATATGGCAATTAGATTGTACCCATTTAGAAGGAAAAGTCATCTTGGTAGCAGTCCATGTAGCCAGTGGTTACATAGAAGCAGAGGTCACCCCAGCGGAAACAGGACAAGAAACAGCACTTTTCATACTAAAATTAGCAGGAAGATGGCCAGTCAAAGTAGTACATACAGACAATGGCAGTAATTTCACCAGTGCTGCAGTCAAGGCAGCCTGTTGGTGGGCAGGTATCCACCAGGAATTTGGAATTCCCTACAATCCCCAAAGTCAAGGAGTAGTAGAATCCATGAATAAAGAATTAAAGAAAATTATAGGGCAGGTAAGAGATCAAGCTGAGCACCTTAAGACAGCAGTACAAATGGCAGTATTCATTCACAATTTTAAAAGAAAAGGGGGGATTGGGGGGTACAGTGCAGGGGAAAGAATAATAGACATAATAGCAACAGACATACAAACTAGAGAATTACAAAAACAAATTATAAAAATTCAAAATTTTCGGGTTTATTACAGAGACAGCAGAGACCCTATTTGGAAAGGACCAGCCAAACTACTCTGGAAAGGTGAAGGGGCAGTAGTAATACAAGATAATAGTGACATAAAGGTAATACCAAGGAGGAAAGCAAAAATCATTAGGGACTATGGAAAACAGATGGCAGGTACTGATAGTGTGGCAGGTAGACAGGATGAAGAT

>AY043176

TTTCTAGATGGAATAGATAAGGCTCAAGAAGATCATGAAAGGTATCACAGCAATTGGAGAGCAATGGCTAATGAGTTTAATCTGCCACCCATAGTAGCAAAAGAAATAGTAGCTAGCTGTGATAAATGTCAGCTAAAAGGGGAAGCCATACATGGACAAGTAGACTGCAACCCAGGGATATGGCAATTAGATTGTACACATTTAGAAGGGAAAATCATCCTGGTAGCAGTCCATGTAGCCAGTGGCTACATGGAAGCAGAGGTTATCCCAGAAGAAACAGGACAAGAAACAGCATACTATATACTAAAACTAGCAGGAAGATGGCCAGTCAAAGTAATACATACAGACAATGGCAGGAATTTCACCAGTAATGCAGTTAAGGCAGCCTGTTGGTGGGCAGGTATCCAACAGGAATTTGGAATTCCCTATAATCCCCAAAGTCAGGGAGTAGTAGAATCCATGAATAAAGAATTAAAGAAAATCATAGGGCAAGTAAGAGATCAAGCTGAACACCTTAAGACAGCAGTACAAATGGCAGTATTCATACACAATTTTAAAAGAAAAGGGGGGATTGGGGGGTACAGTGCAGGGGAAAGGATAATAGACATAATAGCAACAGACATACAAACTACAGAATTACAAAAACAAATTACAAAAATTCAAAATTTTCGGGTTTATTACAGAGACAGCAGAGACCCTATTTGGAAAGGACCAGCCAAACTCCTCTGGAAAGGTGAAGGGGCAGTAGTAATACAAGATAATAGTGACATAAAGGTAGTACCAAGGAGGAAGGCAAAAATCATTAGGGACTATGGAAAACAGATGGCAGGTGCTGATTGTGTGGCAGGTAGACAGGATGAAGAT

>AY043175

TTTCTAGATGGAATAGATAAGGCTCAAGATGAGCATGAAAAGTATCACAGCAATTGGAGAGCAATGGCTAGTGAGTTTAATCTGCCACCCGTAGTAGCTAAAGAAATAGTAGCTAGCTGTGATAAATGTCAGCTAAAAGGGGAAGCCATACATGGACAAGTAGATTGTAGTCCGGGGATATGGCAATTAGACTGTACACATTTAGAAGGAAAAATCATCCTGGTAGCAGTCCATGTAGCCAGTGGCTACATAGAAGCAGAGGTTATCCCAGCAGAAACAGGACAAGAAACAGCATACTATATACTAAAATTAGCAGGAAGATGGCCAGTCAAAGTAATACATACAGACAATGGCAGTAATTTCACCAGTGCTGCAGTTAAGGCAGCCTGTTGGTGGGCAGGTATCAAACAGGAATTTGGGATTCCCTACAATCCCCAAAGTCAGGGAGTAGTAGAATCCATGAATAAAGAATTAAAGAAAATCATAGGGCAGGTAAGAGATCAAGCTGAGCACCTTAAGACAGCAGTACAAATGGCAGTATTCATTCACAATTTTAAAAGAAAAGGGGGGATTGGGGAGTATAGTGCAGGGGAAAGAATAATAGACATAATAGCAACAGACATACAAACTAAAGAATTACAAAAACAAATTTTAAAAATTCAAAATTTTCGGGTTTATTACAGAGACAGCAGAGATCCTATTTGGAAAGGACCAGCCAAGCTACTCTGGAAAGGTGAAGGGGCAGTAGTAATACAAGATAACAGTGACATAAAGGTAGTACCAAGGAGGAAAGTAAAAATCATTAGGGACTATGGAAAACAGATGGCAGGTGCTGATTGTGTGGCAGGTAGACAGGATGAAGAT

>AY043174

TTTCTAGATGGAATAGATAAGGCTCAAGAAGAGCATGAAAAATATCACAGCAATTGGAGAGCAATGGCTAGTGAGTTTAATCTGCCACCCATAGTAGCAAAAGAAATAGTAGCTAGCTGTGATAAATGTCAGCAAAAAGGGGAAGCCATGCATGGACAAGTAGACTGTAGTCCAGGGATATGGCAATTAGATTGTACACATTTAGAAGGAAAAGTCATCCTAGTAGCAGTCCATGTAGCTAGTGGCTACATGGAAGCAGAGGTTATCCCAGCAGAAACAGGACAAGAAACAGCATACTATATACTAAAATTAGCAGGAAGATGGCCAGTTAAAGTAATACACACAGACAATGGTAGTAATTTCACCAGTAGTGCAGTTAAGGCAGCCTGTTGGTGGGCAGGTATCCAGCAGGAATTTGGAATTCCCTACAATCCCCAAAGTCAGGGAGTAGTAGAATCCATGAATAAAGAATTAAAGAAAATCATAGGACAGGTAAGAGATCAAGCTGAGCACCTTAAGACAGCAGTACAAATGGCAGTATTCATTCACAATTTTAAAAGAAAAGGGGGGATTGGGGGGTACAGTGCAGGGGAAAGGATAATAGACATAATAGCAACAGACATACAAACTAAAGAATTACAAAAACAAATTATAAAAATTCAAAATTTCCGGGTTTATTACAGAGACAACAGAGACCCTATTTGGAAAGGACCAGCCAAACTACTCTGGAAAGGTGAAGGGGCAGTAGTAATACAAGATAACAGTGACATAAAGGTAGTACCAAGGAGGAAAGTAAAAATCATTAGGGACTATGGAAAACAGATGGCAGGTGCTGATTGTGTGGCAGGTAGACAGGATGAAGAT

>AY043173

CTAGATGGAATAGATAAGGCTCATGAAGAGCATGAAAAGTATCACAGCAATTGGAGAGCAATGGCTAGTGAGTTTAATCTGCCACCCGTAGTAGCAAGAGAAATAGTAGCCAGCTGTGATAAATGTCAGCTAAAAGGGGAAGCCATACATGGACAAGTAGATTGTAGTCCGGGGATATGGCAATTAGATTGTACACATTTAGAAGGAAAAATCATCCTGGTAGCAGTCCATGTAGCCAGTGGCTACATAGAAGCAGAGGTTATCCCAGCAGAAACAGGACAAGAAACAGCATACTATATACTAAAATTAGCAGGAAGATGGCCAGTCAAAGTAATACATACAGACAATGGCAGTAATTTCACCAGTGCTGCAGTTAAGGCAGCCTGTTGGTGGGCAGGTATCCAACAGGAATTTGGGATTCCCTACAATCCCCAAAGTCAGGGAGTAGTAGAATCCATGAATAAAGAATTAAAGAAAATCATAGGGCAGGTAAGAGATCAAGCTGAGCACCTTAAGACAGCAGTACAAATGGCAGTATTCATTCACAATTTTAAAAGAAAAGGGGGGATTGGGGGGTACAGTGCAGGGGAAAGAATAATAGACATAATAGCAACAGACATACAAACTAAAGAATTACAAAAACAAATTATAAAAATTCAAAATTTTCGGGTTTATTACAGAGACAGCAGAGATCCTATTTGGAAAGGACCAGCCAAGCTACTCTGGAAAGGTGAAGGGGCAGTAGTAATACAAGACAACAGTGACATAAAGGTAGTACCAAGGAGGAAAGTAAAAATCATTAGGGACTATGGAAAACAGATGGCAGGTGCTGATTGTGTGGCAGGTAGACAGGATGAAGAT

>AF355337

TTTCTAGATGGGATAGACAAGGCTCAAGAAGAACATGAAAAATATCACAGTAACTGGAGAGCAATGGCTAGTGATTTTAATCTGCCACCTATAGTAGCAAAGGAAATAGTAGCCAGCTGTGATAAATGTCAACTAAAAGGGGAAGCCATGCATGGACAAGTAGACTGTAGTCCAGGAATATGGCAATTAGATTGCACACATCTAGAGGGAAAAATAATCCTGGTAGCAGTCCATGTAGCCAGTGGCTACATAGAAGCAGAAGTTATCCCAGCAGAAACAGGACAAGAAACAGCATACTTTATATTAAAATTAGCAGGAAGATGGCCAGTAAAAGTAGTACATACAGACAATGGCAGCAATTTCACCAGCGCTGCAGTTAAAGCAGCCTGTTGGTGGGCAGATATCCAACAGGAATTTGGGATTCCCTACAATCCCCAAAGTCAAGGAGTGGTGGAATCTATGAATAAGGAATTAAAGAAAATCATAGGGCAGGTAAGAGAGCAAGCTGAACATCTTAAGACAGCAGTACAAATGGCAGTATTCATCCACAATTTTAAAAGAAAAGGGGGGATTGGGGGGTACAGTGCAGGGGAAAGAATAGTAGACATAATAGCAACAGACTTACAAACTAAAGAACTACGAAAACAAATTACAAAAATTCAAAATTTTCGGGTTTATTTCAGGGACAGCAGAGACCCAATTTGGAAAGGACCAGCAAAACTACTCTGGAAAGGTGAAGGGGCAGTAGTAATACAGGACAATAATGATATAAAGGTAGTACCAAGAAGAAAGGCAAAAATCATTAGGGATTATGGAAAACAGATGGCAGGTGATGATTGTGTGGCAAGTACACAGGATGAGAAT

>AF457090

TTCTTGGATGGAATAGATAAGGCTCAGGAAGAACATGAGAGATATCATAACAACTGGAGAGCAATGGCTAGTGATTTTAACCTGCCCCCTGTGGTAGCAAAAGAAATAGTAGCTAGCTGTGATAAATGTCAGCTAAAAGGAGAAGCCTTGCATGGACAAGTAGACTGTAGTCCAGGAATATGGCAATTAGATTGTACACACTTAGAAGGAAAAGTTATCCTGGTAGCAGTCCATGTAGCCAGTGGTTATATAGAAGCAGAAGTTATTCCAGCAGAAACAGGGCAGGAAACAGCCTACTTCCTCTTGAAATTGGCAGGGAGATGGCCAGTAAAAGTAGTACATACAGACAATGGCAGCAACTTCACCAGCGCTGCAGTTAAGGCCGCCTGTTGGTGGGCAGGCATTAAGCAGGAATTTGGAATTCCCTACAATCCCCAAAGTCAAGGAGTAGTAGAATCTATGAATAAAGAATTAAAGAAAATTATAGGACAAGTAAGAGATCAAGCTGAACATCTTAAGACAGCAGTACAAATGGCAGTATTCATCCACAATTTTAAAAGAAAAGGGGGGATTGGGGGGTACAGTGCAGGGGAAAGAATAATAGACATAATAGCAACAGACATACAAACTAAAGAATTACAAAAACAAATTATAAAAATTCAAAATTTTCGGGTTTATTACAGGGACAGCAGAGATCCAATTTGGAAAGGACCAGCAAAGCTTCTCTGGAAAGGTGAAGGGGCAGTAGTAATACAAGATAATAGTGAAATAAAGGTAGTACCAAGAAGAAAAGCAAAGATCATTAGGGATTATGGAAAACAGATGGCAGGTGATGATTGTGTGGCAAGTAGACAGGATGAGGAT

>AF457089

TTTTTAGATGGGATAGATAAAGCTCAAGAAGAACATGAAAGATATCACAGCAATTGGAGAACAATGGCTAGTGATTTTAATCTGCCACCTATAGTAGCAAAGGAAATAGTAGCCAGCTGTGATAAATGTCAGCTAAAAGGGGAAGCCATGCATGGACAAGTAGACTGCAGTCCAGGGATATGGCAATTAGATTGCACACATCTAGAAGGAAAAGTAATTCTGGTAGCAGTCCATGTAGCCAGTGGCTATATAGAAGCAGAAGTTATCCCAGCAGAAACAGGACAAGAAGCAGCATACTTTCTACTAAAGTTAGCAGGAAGATGGCCAGTAAAAGTAGTACACACGGACAATGGCAGCAATTTCACCAGTGCTGCATTTAAAGCAGCCTGTTGGTGGGCAAATGTCAAACAGGAATTTGGAATTCCCTACAATCCCCAAAGTCAAGGAGTAGTAGAATCTATGAATAAGGAATTAAAGAAAATCATAGGGCAGGTAAGAGAGCAAGCTGAACACCTTAAGACAGCGGTACAAATGGCAGTGTTCATTCACAATTTTAAAAGAAAAGGGGGGATTGGGGGGTACAGTGCAGGGGAAAGAATAATAGATATAATAGCAACAGACATACAAACCAGAGAATTACAAAAACAAATTACAAAAATTCAAAAATTTCGGGTTTATTACAGGGACAGCAGAGATCCAGTTTGGAAAGGACCAGCAAAACTACTCTGGAAAGGTGAAGGGGCAGTAGTAATACAGGACAATAGTGATATAAAGGTAGTACCAAGAAGAAAAGCAAAGATCCTTAGGGATTATGGAAAACAGATGGCAGGTGATGATTGTGTGGCAGGTAGACAGGATGAGGAT

>AF457088

TTTTTAGATGGGATAGATAAGGCTCAAGAAGAGCATGAAAGATATCACAGCAATTGGAGAGCAATGGCTAGTGATTTTAATCTACCACCTGTAGTAGCAAAGGAAATAGTAGCCAGCTGTGATAAATGTCAACAAAAAGGGGAAGCCATGCATGGACAAGTAGACTGTAGTCCAGGGATGTGGCAATTAGATTGCACACATCTAGAAGGAAAAGTAATTCTGGTAGCAGTCCATGTAGCCAGTGGCTATATAGAAGCAGAAGTTATCTCAGCAGAAACAGGACAGGAGACAGCATATTTTCTGCTAAAATTAGCAGGAAGATGGCCAGTAAAAATAGTACACACAGACAATGGCAGCAACTTCACCAGTGCTGCATTTAAAGCAGCTTGTTGGTGGGCAAGTGTCCAACAGGAATTTGGGATTCCCTACAATCCCCAAAGTCAAGGAGTAGTGGAATCTATGAATAAGGAATTAAAGAAAATCATAGGGCAGGTAAGAGACCAAGCTGAACACCTTAAGACAGCAGTACAAATGGCAGTATTCATTCACAATTTTAAAAGAAAAGGGGGGATTGGGGGGTACAGTGCAGGGGAAAGAATAATAGACATAATAGCAACAGACATACAAACTAGAGAACTACAAAAACAAATTACAAAAATTCAAAATTTTCGGGTTTATTACAGGGACAGCAGAGATCCACATTGGAAAGGACCAGCAAAACTACTCTGGAAAGGTGAAGGGGCAGTAGTAATACAGGACAATAGCGATATAAAGGTAGTGCCCAGAAGAAAAGCAAAGATCATTAGGGACTATGGAAAACAGATGGCAGGTGATGATTGTGTGGCAGGTAGACAGGATGAGGAT

>AF457087

TTTTTAGATGGGATAGATAAAGCCCAAGAAGAACATGAAAGATATCACAGCAATTGGAGAGCAATGGCTAGTGATTTTAATCTGCCACCTATAGTAGCAAAGGAAATAGTAGCCAGCTGTGATAAATGTCAACTAAAAGGGGAAGCCATACATGGACAAGTAGACTGTAGTCCAGGGATGTGGCAATTAGATTGCACACATCTAGAAGGAAAAGTAATTTTGGTAGCAGTCCATGTAGCCAGTGGCTATATAGAAGCAGAAGTTATCCCAGCAGAAACAGGACAGGAGACAGCATACTTTCTGTTAAAATTAGCAGGAAGATGGCCAGTAAAAGTAGTACACACAGATAATGGCAGCAATTTCACCAGTGCTGCAGTTAAAGCAGCCTGTTGGTGGGCAGGTATCCAACAAGAATTTGGAATTCCCTACAATCCCCAAAGTCAAGGAGTAGTGGAATCTATGAATAAGGAATTAAAGAAAATCATAGGGCAGGTAAGAGAGCAAGCTGAACACCTTAAGACAGCAGTACAAATGGCAGTATTCATTCACAATTTTAAAAGAAAAGGGGGGATTGGGGGGTACAGTGCAGGGGAAAGAATAATAGACATAATAGCAACAGACATACAAACTAAAGAACTACAAAAACAAATTACAAAAATTCAAAATTTTCGGGTTTATTACAGGGACAGCAGAGATCCACTTTGGAAAGGACCAGCAAAACTACTCTGGAAAGGTGAAGGAGCAGTAGTAATACAGGACAATAGTGATATAAAGGTAGTCCCCAGAAGAAAAGTAAAGATCCTTAGGGATTATGGAAAACAGATGGCAGGTGATGATTGTGTGGCAGGTAGACAGGATGAGGAT

>AF457086

TTTTTAGATGGGATAGATAAAGCTCAAGAAGACCATGAAAGATATCACAGCAATTGGAGAGCAATGGCTAGTGATTTTAATCTGCCACCTATAGTAGCAAAAGAAATAGTAGCCAGCTGTGATAAATGTCAACTAAAAGGGGAAGCCATGCATGGACAAGTAGACTGTAGTCCAGGGATGTGGCAAATAGATTGCACACATCTAGAAGGAAAAGTAATTCTAGTAGCAGTCCATGTAGCCAGTGGCTATATAGAAGCAGAAGTTATCCCAGCAGAAACAGGACAGGAGTCAGCATACTTTCTGCTAAAATTAGCAGGGAGATGGCCAGTAAAAGTAGTACACACAGACAATGGCAGCAATTTCACCAGCGCTGCATTTAAAGCAGCCTGTTGGTGGGCAAATGTCCAACAGGAATTTGGAATTCCCTACAATCCCCAAAGTCAAGGAGTAGTGGAATCTATGAATAAGGAATTAAAGAAAATCATAGGACAGGTAAGAGAGCAAGCTGAACACCTTAAAACAGCAGTACAAATGGCAGTATTCATTCACAATTTTAAAAGAAAAGGGGGGATTGGGGGGTACAGTGCAGGGGAAAGAATAATAGACATAATAGCAACAGACATACAAACTAAAGAACTACAAAAACAAATTACAAAAATTCAAAATTTTCGGGTTTATTACAGGGACAGCAGAGATCCACTTTGGAAAGGACCAGCAAAACTACTTTGGAAAGGTGAAGGGGCAGTAGTAATACAGGACAATAGTGACATAAAGGTAGTACCAAGAAGAAAAGCAAAGATCATCAGGGACTATGGAAAACAGATGGCAGGTGATGATTGTGTGGCAGGTAGACAGGATGAGGAT

>AF457085

TTTCTAGATGGAATAGATAAGGCCCAAGAAGAGCATGAAAAATATCACAACAATTGGAGGGCAATGGCTAGTGACTTTAATCTGCCACCCATAGTAGCAAAAGAAATAGTAGCTAGCTGTGATAAATGTCAGCTAAAAGGGGAAGCCATACATGGACAAGTAGACTGTAGTCCAGGAATATGGCAATTAGATTGTACACATCTAGAAGGAAAAGTCATCCTGGTAGCAGTCCATGTAGCCAGTGGCTACATGGAAGCAGAGGTTATCCCAGCCGAAACAGGACAAGAAACAGCATACTATATACTAAAATTAGCAGGAAGATGGCCAGTCAAAGTAATACATACAGACAATGGCAGCAATTTCACCAGTCAGGCAGTTAAGGCAGCCTGTTGGTGGGCAGGTATCCAACAGGAATTTGGAATTCCCTACAATCCCCAAAGTCAGGGAGTAGTAGAATCCATGAATAAAGAATTAAAGAAAATCATAGGGCAGGTGCGAGATCAAGCTGAGCACCTTAAGACAGCAGTACAAATGGCAGTATTCATTCACAATTTTAAAAGAAAAGGGGGGATTGGGGGGTACAGTGCAGGGGAAAGAATGATAGACATAATAGCAACAGACTTACAAACTAAAGAACTACAAAAACAAATTATAAAAATTCAAAATTTTCGGGTTTATTACAGAGACAGCAGAGACCCTATTTGGAAAGGACCAGCCAAACTACTCTGGAAAGGTGAAGGGGCAGTAGTAATACAAGATAACAGTGACATAAAGGTAGTACCAAGGAGGAAAGCAAAAATCATTAAGGACTATGGAAAACAGATGGCAGGGGCTGATTGTGTGGCAAGTAGACAGGATGAAGAT

>AF457084

TTTTTAGATGGGATAGATAAGGCTCAAGAAGATCATGAAAGATATCATAGCAATTGGAGAACAATGGCTAGTGATTTTAACCTGCCACCTGTAGTAGCAAAGGAGATAGTAGCCAGCTGTGATAAATGTCAGCTAAAAGGGGAAGCCATGCATGGACAGGTAGACTGTAGCCCAGGGATATGGCAATTAGATTGCACACATCTAGAAGGAAAAGTAATTCTGGTAGCAGTTCATGTAGCCAGTGGCTATATAGAAGCAGAAGTTATCCCAGCAGAAACAGGGCAGGAGACAGCATACTTTCTGCTAAAATTAGCAGGAAGATGGCCAGTAAAAGTAATTCACACAGACAATGGCAGCAATTTCACCAGTGCTGCAGTTAAAGCAGCCTGTTGGTGGGCAGGTATCCAACAGGAATTTGGGATTCCCTACAATCCCCAAAGTCAAGGAGTAGTGGAATCTATGAATAAGGAATTAAAAAAAATCATAGGGCAGGTAAGAGAGCAAGCTGAACATCTTAAAACAGCAGTACAAATGGCAGTATTCATTCACAATTTTAAAAGAAAAGGGGGGATTGGGGGGTACAGTGCAGGGGAAAGAATAATAGACATAATAGCAACAGACATCCAAACTAAAGAACTACAAAAACAAATTACAAAAATTCAAAATTTTCGGGTTTATTACAGGGACAGCAGAGATCCACTTTGGAAAGGACCAGCAAAACTACTCTGGAAAGGTGAAGGGGCAGTAGTAATACAGGACAATAGTGATATAAAAGTAGTGCCCAGAAGAAAAGCAAAAATCATTAGGGATTATGGAAAACAGATGGCAGGTGATGATTGTGTGGCAGGTAGACAGGATGAGGAT

>AF457083

TTTTTAGATGGAATAGATAAAGCTCAAGAAGAACATGAAAGATATCACAGCAATTGGAGAGCAATGGCTAGTGATTTTAATCTGCCACCTATAGTAGCAAAGGAAATAGTAGCCAGCTGTGATAAATGTCAACTAAAAGGGGAAGCCATGCATGGACAAGTAGACTGTAGTCCAGGGATGTGGCAATTAGATTGCACACATCTAGAAGGAAAAATAATTCTAGTAGCAGTTCATGTAGCCAGTGGCTATATAGAAGCGGAAGTTATCCCAGCAGAAACAGGACAGGAGACAGCATACTTTCTACTAAAATTAGCAGGAAGATGGCCAGTAAAAGTAGTACACACAGACAATGGCAGCAATTTCACCAGCACTGCATTTAAAGCAGCCTGTTGGTGGGCAGGTGTCCAACAAGAATTTGGGATTCCCTACAATCCCCAAAGTCAAGGAGTAGTGGAGTCTATGAATAAGGAATTAAAGAAAATCATAGGGCAGGTAAGAGAACAAGCTGAACACCTTAAGACAGCAGTACAAATGGCAGTATTCATTCACAATTTTAAAAGAAAAGGGGGGATTGGGGGATACAGTGCAGGGGAAAGAATAATAGACATAATAGCAACAGACATACAAACTAAAGAACTACAAAAACAAATTACAAAAATTCAAAATTTTCGGGTTTATTACAGGGACAGCAGAGATCCACTTTGGAAAGGACCAGCAAAACTACTCTGGAAAGGTGAAGGGGCAGTAGTAATACAGGATAATAGTGATATAAAGGTAGTGCCCAGAAGAAAAGCAAAGATCATTAGGGATTATGGAAAACAGATGGCAGGTGATGATTGTGTGGCAGGTAGACAGGATGAGGAT

>AF457082

TTTTTAGATGGGATAGATAAAGCTCAAGAAGAACATGAAAGATATCACAGCAATTGGAGAGCAATGGCTAGTGATTTTAATCTGCCACCTATAGTAGCAAAGGAAATAGTAGCCAGCTGTGATAAATGTCAACTAAAAGGGGAAGCTATACATGGACAAGTAGACTGTAGTCCAGGGATGTGGCAATTAGACTGCACACATCTAGAAGGAAAAGTAATTGTGGTAGCAGTCCATGTAGCCAGTGGCTATATAGAGGCAGAAGTTATCCCCGCAGAAACAGGACAGGATACAGCATACTTTCTGCTAAAATTGGCAGGAAGATGGCCAGTACAAGTAGTACACACAGACAATGGCAGCAATTTCACTAGCGCTGCATTTAAAGCAGCCTGTTGGTGGGCCGGTATCCAACAGGAATTTGGGATTCCCTACAATCCCCAAAGTCAAGGAGTAGTAGAATCTATGAATAAAGAATTAAAGAAAATTATAGGACAGGTAAGAGATCAAGCTGAACATCTTAAGACAGCAGTACAGATGGCAGTATTCATTCACAATTTTAAAAGAAAAGGGGGGATTGGGGGGTACAGTGCAGGGGAAAGAATAATAGACATAATAGCAACAGACATACAAACTAGAGAATTACAAAAACAAATCTCAAAAATTCAAAACTTTCGGGTTTATTTCAGGGACAGCAGAGATCCAATTTGGAAAGGACCAGCAAAACTACTCTGGAAAGGTGAAGGGGCAGTAGTAATACAGGACAATAATGATATAAAGGTAGTACCAAGAAGAAAAGCAAAGATTATTAGGGATTATGGAAAACAGATGGCAGGTAATGATTGTGTGGCAGGTAGACAGGATGAGGAT

>AF457081

TTTTTAGATGGGATAGATAAGGCTCAAGAAGACCATGAAAGATATCACAGCAATTGGAGAACAATGGCTAGTGATTTTAATCTGCCACCTATAGTAGCAAAAGAAATAGTAGCCAGCTGTGATAAATGTCAACTAAAAGGGGAAGCCATGCATGGACAAGTAGACTGTAGCCCAGGGATATGGCAATTAGATTGCACACATCTAGAAGGGAAAGTAATCCTGGTAGCAGTCCATGTAGCCAGTGGCTATATAGAAGCAGAAGTTATCCCAGCAGAAACAGGGCAAGAGACAGCATACTTTCTGCTAAAATTAGCAGGAAGATGGCCAGTAAAAGTAGTACACACTGACAATGGAAGCAATTTCACCAGTGCTGCAGTTAAAGCAGCCTGTTGGTGGGCAAATATCCAACAGGAATTTGGGATTCCCTACAATCCCCAAAGTCAAGGAGTAGTGGAATCTATGAATAAAGAATTAAAGAAAATCATAGGGCAAGTAAGAGATCAAGCTGAACATCTTAAAACAGCAGTACAAATGGCAGTATTTATTCACAATTTTAAAAGAAAAGGGGGGATTGGGGGGTACAGTGCAGGGGAAAGAATAATAGACATAATAGCAACAGACATACAAACTAAAGAATTACAAAAACAAATTACAAAAATTCAAAATTTTCGGGTTTATTACAGGGACAGCAGAGATCCCATTTGGAAAGGACCAGCAAAACTACTTTGGAAAGGTGAAGGGGCAGTAGTAATACAGGACAACAGTGATATAAAGGTAGTACCAAGAAGAAAAGCAAAGATCCTTAGGGATTATGGAAAACAGATGGCAGGTGATGATTGTGTGGCAGGTAGACAGGATGAGGAT

>AF457080

TTTTTAGATGGGATAGATAAAGCTCAAGAAGAACATGAAAGATATCACAGCAATTGGAGAACAATGGCTAGTGACTTTAATCTGCCACCTGTAATAGCAAAGGAAATAGTAGCCTGCTGTGATAAATGTCAGCTAAAAGGGGAAGCCATACATGGACAAGTAGACTGTAGTCCAGGGATATGGCAACTAGATTGCACACATCTAGAAGGAAAAGTAATTCTGGTAGCAGTTCATGTAGCCAGTGGCTACATAGAAGCAGAAGTAATCCCAGCAGAAACAGGACAAGAGACAGCATACTTTATATTAAAATTAGCAGGAAGATGGCCAGTAAAAGTAGTACACACAGACAATGGCAGCAACTTCACCAGCGCTGCAGTTAAAGCAGCCTGTTGGTGGGCAAATATCCAACAGGAATTTGGGATTCCCTACAATCCCCAAAGTCAAGGAGTAGTGGAGTCTATGAATAAGGAATTAAAGAAAATCATAGGACAGGTAAGAGATCAAGCAGAACACCTTAAAACAGCAGTACAAATGGCAGTCTTCATTCACAATTTTAAAAGAAAAGGGGGGATTGGGGGGTACAGTGCAGGGGAAAGAATAATAGACATAATAGCAACAGACATACAAACTAAAGAATTACAAAAACAAATTACAAAAATTCAAAATTTTCGGGTTTATTACAGGGACAGCAGAGATCCACTTTGGAAAGGACCAGCAAAACTACTCTGGAAAGGTGAAGGGGCAGTAGTAATACAGGACAATAGTGATATAAAGGTAGTACCAAGAAGAAAAGCAAAGATCATTAGGGAATATGGAAAACAGATGGCAGGTGATGATTGTGTGGCAGGTAGACAGGATGAGGAT

>AF457079

TTTTTAGATGGGATAGATAAGGCTCAAGAAGAACATGAAAGATATCACAGCAATTGGAGAGCAATGGCTAGTGATTTTAACCTACCACCTATAGTAGCAAAGGAAATAGTAGCCAGCTGTGATAAATGTCAGCTAAAAGGGGAAGCCATGCATGGACAAGTAGACTGCAGTCCAGGAATATGGCAGTTAGATTGCACACATCTAGAAGGAAAAGTAATTCTGGTAGCAGTTCATGTAGCCAGTGGCTATATAGAAGCAGAAGTTATCCCAGCAGAAACAGGACAAGAGACAGCATACTTTATACTAAAATTAGCAGGAAGATGGCCAGTAAAAGTAGTACACACAGACAATGGCAGCAATTTCACCAGCGGTGCATTCAAAGCAGCCTGTTGGTGGGCAGGTATCCAACAGGAATTTGGAATTCCCTACAATCCCCAGAGTCAAGGAGTAGTAGAATCTATGAATAAGGAATTAAAGAAAATCATAGGACAGGTAAGAGAGCAAGCTGAACACCTTAAAACAGCAGTACAAATGGCAGTATTCATTCACAATTTTAAAAGAAAAGGGGGGATTGGGGGGTACAGTGCAGGGGAAAGAATAATAGACATAATAGCAACAGACATACAAACTAAAGAATTACAAAAACAAATTACAAAAATTCAAAATTTTCGGGTTTATTACAGGGACAGCAGAGATCCACTTTGGAAAGGACCAGCAAAACTGCTCTGGAAAGGTGAAGGGGCAGTAGTAATACAGGATAATAGTGATATAAAGGTAGTACCAAGAAGAAAAGCAAAGATCATCAGGGACTATGGAAAACAGATGGCAGGTGATGATTGTGTGGCAGGTAGACAGGATGAGGAT

>AF457078

TTTTTAGATGGGATAGATAAAGCTCAAGAAGAGCATGAAAGGTATCACAGCAATTGGAGAACAATGGCTAGTGACTTTAATCTACCACCTATAGTAGCAAAGGAAATAGTAGCCAGCTGTGATAAATGTCAGCTAAAAGGGGAAGCCATGCATGGACAGGTAGACTGCAGTCCAGGGATATGGCAATTAGATTGCACACATCTAGAAGGAAAAGTAATTCTGGTAGCAGTTCATGTAGCCAGTGGCTACATAGAAGCAGAAGTTATCCCAGCAGAAACAGGACAAGAGACAGCATATTTTCTACTAAAATTAGCAGGAAGATGGCCAGTAAAAGTAGTACACACAGATAATGGCAGCAATTTCACCAGCGCTGCATTCAAAGCAGCCTGTTGGTGGGCAAATGTCAAACAGGAGTTTGGGATTCCCTACAATCCCCAAAGTCAAGGAGTAGTGGAATCTATGAATAAGGAATTAAAGAAAATCATAGGGCAGATAAGAGACCAAGCTGAACACCTTAAGACAGCAGTACAAATGGCAGTATTCATCCACAATTTTAAAAGAAAAGGGGGGATTGGGGGGTACAGTGCAGGGGAAAGAATAATAGACATAATAGCAACAGACATACAAACTAAAGAATTACAAAAACAAATTACAAAAATTCAAAAATTTCGGGTTTATTACAGGGACAGCAGAGATCCAGTTTGGAAAGGACCAGCAAAACTACTCTGGAAAGGTGAAGGGGCAGTGGTAATACAGGACAATAGTGATATAAAGGTAGTACCAAGAAGAAAAGCTAAGATCATTAGGGATTATGGAAAACAGATGGCAGGTGATGATTGTGTGGCAGGTGGACAGGATGAGGAT

>AF457077

TTTTTAGATGGGATAGATAAAGCTCAAGAAGAACATGAAAGATATCACAGCAATTGGAGAACAATGGCTAGTGATTTTAATCTGCCACCTATAGTAGCAAAGGAAATAGTAGCCAGCTGTAATAAATGTCAGCTAAAAGGGGAAGCCATGCATGGACAAGTAGACTGCAGTCCAGGGATATGGCAATTAGATTGCACACATCTAGAAGGAAAAATAATCATAGTAGCAGTTCATGTAGCTAGTGGCTATATAGAAGCAGAAGTTATCCCAGCAGAAACAGGACAAGAGGCAGCATACTTTCTACTAAAATTAGCAGGAAGATGGCCAGTAAAAGTAGTGCATACAGATAATGGCAGCAATTTCACCAGTGCTGCATTTAAAGCAGCCTGTTGGTGGGCAAATATCCAACAGGAATTTGGGATTCCCTACAATCCCCAAAGTCAAGGAGTAGTGGAGTCTATGAATAAGCAATTAAAGCAAATCATAGGACAAGTAAGAGAGCAAGCTGAACACCTTAAAACAGCAGTACAAATGGCAGTATTCATTCACAATTTTAAAAGAAAAGGGGGGATTGGGGGGTACAGTGCAGGGGAAAGAATAATAGACATAATAGCAACAGACATACAAACTAAAGAATTACAAAAACATATTACAAAAATTCAAAATTTTCGGGTTTATTTCAGGGACAGCAGAGATCCAGTTTGGAAAGGACCAGCAAAACTACTCTGGAAAGGTGAAGGGGCAGTAGTAATACAGGACAATAGTGACATAAAGGTAGTACCAAGAAGGAAAGCAAAAATCATCAGGGATTATGGAAAACAGATGGCAGGTGATGATTGTGTGGCAGGTAGACAGGATGAGGAT

>AF457075

TTTTTAGATGGGATAGATAAAGCTCAAGAAGAACATGAAAGATATCACAGCAATTGGAAAGCAATGGCTAGTGATTTTAACCTGCCACCTGTAGTAGCAAAGGAAATAGTAGCCAGCTGTGATAAATGTCAACTAAAAGGGGAAGCCATACATGGACAAGTAGACTGCAGTCCAGGGATATGGCAATTAGATTGCACACATCTAGAAGGAAAAGTAATTCTGGTAGCAGTTCATGTAGCCAGTGGTTATATAGAAGCAGAAGTTATCCCAGCAGAAACAGGACAGGAGACAGCATACTTTCTACTAAAATTAGCAGGAAGATGGCCAGTAAAAGTAGTACACACAGACAATGGCAGCAATTTCACCAGTGCTGCAGTTAAAGCAGCCTGTTGGTGGGCAGGTATCCAACAAGAATTTGGGATTCCCTACAATCCCCAAAGTCAAGGAGTAGTGGAATCTATGAATAAGGAATTAAAGAAAATCATAGGGCAGGTAAGAGAGCAAGCTGAACACCTTAGAACAGCAGTACAAATGGCAGTATTCATTCACAATTTTAAAAGAAAAGGGGGGATTGGAGGGTACAGTGCAGGGGAAAGAATAATAGACATAATAGCAACAGACATACAAACTAAAGAATTACAAAAACAAATTACAAAAATTCAAAATTTTCGGGTTTATTACAGGGACAGCAGAGATCCAGTTTGGAAAGGACCAGCAAAACTACTCTGGAAAGGTGAAGGGGCAGTAGTAATACAGGATAATAGTGATATAAAGGTAGTACCAAGAAGGAAAGCAAAGATCATAAGGGACTATGGAAAACAGATGGCAGGTGATGATTGTGTGGCAGGTAGACAGGATGAGGAT

>AF457073

TTTTTGGATGGAATAGATAAGGCTCAAGAAGAACATGAGAAATACCACAGCAATTGGAGAGCAATGGCTAGTGATTTTAATCTGCCACCTGTGGTAGCAAAAGAAATAGTAGCTAGCTGTGATAAATGTCAGCTAAAAGGAGAAGCCTTGCATGGACAAGTAGATTGTAGTCCAGGAATATGGCAATTAGATTGTACACACTTAGAAGGAAAAGTTATCCTGGTAGCAGTCCATGTAGCCAGTGGCTATATAGAAGCAGAAGTCATTCCAGCAGAAACAGGGCAGGAAACAGCCTACTTTCTCTTAAAATTAGCAGGAAGATGGCCAGTGAAAGTAGTACACACAGACAATGGCAGCAATTTCACCAGCGCGGTGGTTAAGGCCGCCTGTTGGTGGGCAGGCATCAAGCAGGAATTTGGAATTCCCTACAATCCCCAAAGTCAAGGAGTAGTAGAATCTATGAATAAAGAATTAAAGAAAATTATAGGACAGGTAAGAGATCAAGCTGAACATCTTAAGACAGCAGTACAAATGGCAGTATTCATCCACAATTTTAAAAGAAAAGGGGGGATTGGGGGGTACAGTGCAGGGGAAAGAATAATAGACATAATAGCAACAGACATACAAACTAAAGAATTACAAAAACACATTACAAATATTCAAAATTTTCGGGTTTATTACAGGGACAGCAGAGATCCAATTTGGAAAGGACCAGCAAAACTTCTCTGGAAAGGTGAAGGGGCAGTAGTAATACAGGACAATAGTGATATAAAGGTAGTACCAAGAAGAAAAGCAAAGATCATTAGGGACTATGGAAAACAGATGGCAGGTGATGATTGTGTGGCAGGTAGACAGGATGAGGAT

>AF457072

TTTTTAGATGGAATAGATAAGGCTCAAGAGGAGCATGAAAGATATCACAGTAATTGGAGAGCAATGGCTCATGACTTTAATATACCACCTGTAGTAGCAAAAGAAATAGTAGCTAGCTGTGATAAATGTCAAATAAAAGGGGAAGCCATGCATGGACAAGTAGACTGTAGTCCAGGCATATGGCAACTAGATTGTACACATCTAGAAGGAAGAGTTATCCTGGTAGCAGTCCATGTAGCCAGTGGCTATATAGAAGCAGAAGTCATCCCAGCAGAAACAGGACAGGAGACAGCATACTTTATATTAAAACTAGCAGGAAGGTGGCCAGTAAAAGTAATACATACGGACAATGGGCCCAATTTTATCAGTGCACCAGTCAAGGCCGCCTGTTGGTGGGCAGGTATCAAACAGGAATTTGGGATTCCTTACAATCCCCAAAGTCAAGGAGTAGTGGAATCTATGAATAAAGAATTAAAGAAAATCATAGGGCAGGTAAGAGATCAAGCTGAACACCTTAAGACAGCAGTACAAATGGCAGTATTCATCCACAATTTTAAAAGAAAAGGGGGGATTGGGGACTATAGTGCAGGGGAAAGAATAATAGACATAATAGCAACAGATATACAAACTAAAGAATTACAAAGACAAATTACAAAAATTCAAAATTTTCGGGTTTATTACAGAGACAGCAGAGACCCAATTTGGAAAGGACCAGCAAAACTCCTCTGGAAAGGTGAAGGGGCAGTAGTAATACAAGACAATAGTGATATAAAGGTAGTACCAAGAAGAAAAGTAAAAATCATTAGGGATTATGGAAAACAGATGGCAGGTGATGATTGTGTGGCAGGTAGACAGGATGAGGAT

>AF457070

TTTCTAGATGGGGTAGATAAAGCTCAAGAAGAACATGAAAGATATCACAGCAATTGGAGAGCAATGGCTAGTGATTTTAATCTGCCACCTGTAGTAGCAAAGGAAATAGTAGCCAGCTGTGATAAATGTCAGCTAAAAGGGGAAGCCATGCATGGACAAGTAGATTGTAGTCCAGGGATATGGCAATTAGATTGCACACATCTAGAAGGAAAAGTAATTCTGGTAGCAGTTCATGTAGCCAGTGGCTATATAGAAGCAGAAGTTATCCCAGCAGAAACAGGACAAGAGACAGCATACTTTCTACTAAAATTGGCAGGAAGATGGCCAGTAAAAGTAGTCCACACAGACAATGGCCCCAACTTTATCAGCGCTGCAGTTAAAGCAGCCTGTTGGTGGGCAGGTATCCAACAGGAATTTGGAATTCCCTACAATCCCCAAAGTCAAGGAGTAGTGGAATCTATGAATAAGGAATTAAAGAAAATCATAGGACAGGTAAGAGAGCAAGCTGAACACCTTAAAACAGCAGTACAAATGGCAGTATTCATTCACAATTTTAAAAGAAAAGGGGGGATTGGGGGGTACAGTGCAGGGGAAAGAATAATAGACATAATAGCAACAGACATACAAACTAAAGAATTACAAAAACAAATTATAAAAATTCAAAATTTTCGGGTTTATTACAGGGACAGCAGAGATCCAATTTGGAAAGGACCAGCAAAACTACTCTGGAAAGGTGAAGGGGCAGTAGTAATACAGGACAATAGTGATATAAAGGTAGTACCAAGAAGAAAAGCAAAAATCATCAGGGACTATGGAAAACAGATGGCAGGTGATGATTGTGTGGCAGGTAGACAGGATGAGGAT

>AF457069

TTTATAGATGGGATAGATAAAGCTCAAGAGGATCATGAAAGATATCACAGCAATTGGAGAACAATGGCTAGTGATTTTAATCTGCCACCTATAGTAGCAAAAGAAATAGTAGCCAGCTGTGATAAATGTCAGCTAAAAGGAGAAGCCATGCATGGACAAGTAGACTGCAGCCCAGGGATATGGCAATTAGATTGCACACATCTAGAAGGAAAAGTAATTTTGGTAGCAGTTCATGTAGCCAGTGGCTATATAGAAGCAGAAGTTATCCCAGCAGAAACAGGACAAGAGACAGCATACTTTCTACTAAAATTAGCAGGAAGATGGCCAGTAAAAGTAGTACACACAGACAATGGCAGCAATTTTACCAGCGCTGCAGTCAAAGCAGCCTGTTGGTGGGCAGGTATCCAACAGGAATTTGGGATCCCCTACAATCCCCAAAGTCAAGGAGTAGTGGAATCTATGAATAAGGAATTAAAGAAAATCATAGGACAGGTAAGAGAGCAAGCTGAACACCTTAAAACAGCTGTACAAATGGCAGTATTCATTCACAATTTTAAAAGAAAAGGGGGGATTGGGGGGTACAGTGCAGGGGAAAGAATAATAGACATAATAGCAACAGACATACAAACTAAAGAATTACAAAAACAAATTACAAAAATTCAAAATTTTCGGGTTTATTACAGGGACAGCAGAGATCCAGTTTGGAAAGGACCAGCAAAACTGCTCTGGAAAGGTGAAGGGGCAGTAGTAATACAGGACAATAGTGATATAAAGGTAGTACCAAGAAGAAAAGCAAAAATCATCAGGGACTATGGAAAACAGATGGCAGGTGATGATTGTGTGGCAGGTAGACAGGATGAGGAT

>AF457068

TTTTTAGATGGGATAGATAAAGCTCAAGAAGAACATGAAAGATATCATAGTAATTGGAGAACAATGGCTAGTGATTTTAATCTGCCACCTATAGTAGCAAAGGAAATAGTAGCCAGCTGTGACAAATGTCAACTAAAAGGGGAAGCCATGCATGGACAAGTAGATTGTAGTCCAGGGATATGGCAATTAGATTGCACACATCTAGAAGGAAAAGTAATCCTGGTAGCAGTCCATGTAGCCAGTGGCTATATAGAAGCAGAAGTTATCCCAGCAGAAACAGGACAGGAAACAGCATACTTTCTGTTAAAATTAGCAGGAAGATGGCCAGTAAAAGTAGTACACACAGACAATGGCAGCAATTTCACCAGCGCTGCATTTAAAGCAGCCTGTTGGTGGGCAAATATCCAACAGGAATTTGGAATTCCCTACAATCCCCAAAGTCAAGGAGTAGTGGAATCTATGAATAAAGAATTAAAGAAAATCATAGGGCAGGTAAGAGAGCAAGCTGAACACCTTAGGACAGCAGTACAAATGGCAGTATTCATTCACAATTTTAAAAGAAAAGGGGGGATTGGGGGGTACAGTGCAGGGGAAAGAATAATAGACATAATAGCAMCAGATATACAAACTAAAGAATTACAAAAACACATTTCAAAAATTCAAAATTTTCGGGTCTATTACAGGGACAGCAGAGATCCCATTTGGAAAGGACCAGCAAAACTACTCTGGAAAGGTGAAGGGGCAGTAGTAATACAGGACAATAGTGATATAAAGGTAGTACCAAGAAGAAAAGCAAAGATCATTAGGGATTATGGAAAACAGATGGCAGGTGATGATTGTATGGCAAGTAGACAGGATGAGGAT

>AF457067

TTTTTAGATGGGATAGATAAAGCTCAAGAAGAACATGAAAGATATCACAGCAATTGGAGAGCAATGGCTAGTGATTTTAATTTGCCACCTATAGTAGCAAAGGAGATAGTAGCCAGCTGTGATAAATGTCAGCTAAAAGGGGAAGCCATGCATGGACAAGTAGACTGCAGTCCAGGGATATGGCAATTAGATTGCACACATCTAGAAGGAAAAGTAATACTGGTAGCAGTTCATGTAGCCAGTGGCTATATGGAAGCAGAAGTTATCCCAGCAGAAACAGGACAAGAGACAGCATACTTTATACTAAAACTAGCAGGAAGATGGCCAGTAAAAGTAGTACACACAGACAATGGCAGCAATTTCACCAGTGCTGCAGTTAAAGCAGCCTGTTGGTGGGCAAATATCAAACAGGAATTTGGGATTCCCTACAATCCCCAAAGTCAAGGAGTAGTGGAATCTCTAAATAAGGAATTAAAGAAAATTATAGGACAGGTAAGAGAGCAAGCTGAACATCTTAAAACAGCAGTACAAATGGCAGTATTCATTCACAATTTTAAAAGAAAAAGGGGGATTGGGGGGTACAGTGCAGGGGAAAGAATAATAGACATAATAGCAACAGACATACAAACTACAGAATTACAAAAACAAATTACAAAAATTCAAAATTTTCGGGTTTATTACAGGGACAGCAGAGATCCAATTTGGAAAGGACCAGCAAAACTACTCTGGAAAGGTGAAGGGGCAGTAGTAATACAGGACAATGGTGATATAAAGGTAGTACCAAGAAGAAAAGTAAAAATCATTAGGGACTATGGAAAACAGATGGCAGGTGATGATTGTGTGGCAGGTGGACAGAATGAGGAT

>AF457066

TTTTTAGATGGGATAGATAAAGCTCAAGAAGAACATGAAAGATATCACAGCAATTGGAGAGCAATGGCTAGTGATTTTAATCTACCACCTATAGTAGCAAAGGAAATAGTAGCCTGCTGTGATAAATGTCAGCTAAAAGGAGAAGCCATACATGGACAAGTAGACTGCAGTCCAGGGATATGGCAATTAGATTGTACACATCTAGAAGGAAAAATAATTTTGGTAGCAGTCCATGTAGCCAGTGGCTATATAGAAGCAGAAGTTATCCCAGCAGAAACAGGACAAGAGACAGCATACTTTATACTAAAATTAGCAGGAAGATGGCCAGTAAAAGTAATACACACAGACAATGGCAGCAATTTCACCAGCGCTGCAGTTAAAGCAGCCTGTTGGTGGGCAGATATCCAGCAGGAATTTGGAATTCCCTACAACCCCCAAAGTCAAGGAGTAGTAGAATCTATGAATAAGGAATTAAAGAAAATCATAGGACAGGTAAGAGAGCAAGCTGAACACCTTAAAACAGCAGTACAAATGGCAGTATTCATTCACAATTTTAAAAGAAAAGGGGGGATTGGGGGGTACAGTGCAGGGGAAAGAATAATAGATATAATAGCAACAGACATACAAACTAAAGAATTACAAAAACACATTACAAAAATTCAAAATTTTCGGGTTTATTACAGGGACAGCAGAGATCCAGTTTGGAAAGGACCAGCAAAACTACTTTGGAAAGGTGAAGGGGCAGTAGTAATACAGGACAATAGTGATATAAAGGTAGTACCAAGAAGAAAAGCAAAAATCATCAGGGATTATGGAAAACAAATGGCAGGTGATGATTGTGTGGCAGGTAGACAGGATGAGGAT

>AF457065

TTTTTAGATGGGATAGATAAAGCTCAAGAAGAACATGAAAGATATCACAGCAATTGGAGAACAATGGCTAGTGACTTTAATCTGCCACCTATAGTAGCAAAGGAAATAGTTGCCAGCTGTGATAAATGTCAACTAAAAGGGGAAGCCATGCATGGACAAGTAGACTGTAGTCCAGGGATATGGCAGTTAGATTGCACACATCTGGAAGGAAAAGTAATTCTGGTAGCAGTCCATGTAGCCAGTGGCTACATAGAAGCAGAAGTTATCCCAGCAGAGACAGGACAGGAGACAGCATACTTTCTGCTAAAATTAGCAGGAAGGTGGCCAGTAAAAGTGGTACACACAGACAATGGCAGCAATTTCACCAGCGCTGCAGTTAAAGCAGCCTGTTGGTGGGCAGGTATCCAACAGGAATTTGGAATTCCCTACAATCCCCAGAGTCAAGGAGTAGTAGAATCTATGAATAAGGAATTAAAGAAAATCATAGGACAAGTAAGAGAGCAAGCTGAACACCTTAAAACAGCAGTACAAATGGCAGTATTCATTCACAATTTTAAAAGAAAAGGGGGGATTGGGGGGTACAGTGCAGGGGAAAGAATAATAGACATAATAGCAACAGACATACAAACTAAAGAATTACAAAAACAAATTACAAAAATTCAAAATTTTCGGGTTTATTACAGGGACAGCAGAGATCCAATTTGGAAAGGACCAGCAAAACTACTCTGGAAAGGTGAAGGGGCAGTAGTAATACAGGACAATAGTGATATAAAAGTAGTACCAAGAAGAAAAGCAAAAATCATTAGAGATTATGGAAAACAGATGGCAGGTGATGATTGTGTGGCAGGTAGACAGGATGAGGAT

>AF457064

TTTTTAGATGGGATAGATAAGGCTCAAGAAGAACATGAAAGATATCACAACAATTGGAGAGCAATGGCTAGTGATTTTAATCTGCCACCTATAATAGCAAAGGAAATAGTAGCCAGCTGTGATAAATGTCAACTAAAAGGGGAAGCCATGCATGGACAAGTAGACTGTAGTCCAGGGATGTGGCAGTTAGATTGCACACATCTAGAAGGAAAGGTAATTCTGGTAGCAGTCCATGTAGCCAGTGGCTATATAGAAGCAGAAGTGATCCCAGCAGAAACAGGACAGGAGACAGCATACTTTCTGCTAAAATTAGCAGGAAGATGGCCAATAAAAGTAGTACACACAGACAATGGCAGCAATTTCACCAGTGCTGCATTTAAAGCAGCCTGTTGGTGGGCAAATATCCAACAGGAATTTGGGATTCCCTACAATCCCCAAAGTCAAGGAGTAGTAGAATCTATGAATAAGGAATTAAAGAAAATCATAGGGCAGGTAAGAGAGCAAGCTGAACACCTTAGGACAGCAGTACAGATGGCAGTATTCATTCACAATTTTAAAAGAAAAGGGGGGATTGGGGGGTACAGTGCAGGGGAAAGAATAATAGACATAATAGCAACAGACATACAAACTAAAGAATTACAAAAACAAATTACAAACATTCAAAAATTTCGGGTTTATTACAGGGACAGCAGAGATCCACTTTGGAAAGGACCAGCAAAACTACTCTGGAAAGGTGAAGGGGCAGTGGTAATACAGGACAATAGTGATATAAAGGTAGTGCCCAGAAGAAAAGCAAAAATCATTAGGGATTATGGAAAACAGATGGCAGGTGATGATTGTGTGGCAGGTAGACAGGATGAGGAT

>AF457063

TTCTTAGATGGGATAGATAAAGCTCAAGAAGAACATGAAAGATATCACAGCAATTGGAAAGCAATGGCTAGTGATTTTAATTTGCCACCTATAGTAGCAAAGGAAATAGTAGCCAGCTGTGATAAATGCCAGCTAAAAGGGGAAGCCATACATGGACAGGTAGACTGCAGCCCAGGGATGTGGCAATTAGATTGCACACATCTAGAAGGAAAAGTAATTCTGGTAGCAGTCCATGTAGCCAGTGGCTATATAGAAGCAGAAGTTATCCCAGCAGAAACAGGACAGGAGACAGCATATTTTCTACTAAAATTAGCAGGAAGATGGCCAGTAAAAGTAGTACACACAGACAATGGCAGCAATTTCACCAGCACTGCATTTAAGGCAGCCTGTTGGTGGGCAGGTGTCCAACAGGAATTTGGGATCCCCTACAATCCCCAAAGTCAAGGAGTAGTGGAATCTATGAATAAAGAATTAAAGAAAATCATAGGACAAGTAAGAGAGCAAGCTGAACACCTTAAGACAGCAGTACAAATGGCAGTATTCATTCACAATTTTAAAAGAAAAGGGGGGATTGGGGGGTACAGTGCAGGGGAAAGAATAATAGACATAATAGCAACAGACATACAAACTAAAGAATTACAAAAACAAATTACAAAAATTCAAAAATTTCGGGTTTATTACAGGGACAGCAGAGATCCAATTTGGAAAGGACCAGCAAAACTACTCTGGAAAGGTGAAGGGGCAGTGGTAATACAGGACAATAGTGATATAAAGGTAGTACCAAGAAGAAAAGTAAAGATCATTAGGGATTATGGAAAACAGATGGCAGGTGATGATTGTGTGGCAGGTAGACAGGATGAGGAT

>AF457062

TTTTTAGATGGTATAGATAAAGCCCAAGAAGAGCATGAAAGATATCACAGCAATTGGAGAGCAATGGCTAGTGACTTTAATCTGCCACCTGTAGTAGCAAAAGAAATAGTGGCCAGCTGTGATAAGTGTCAGTTAAAAGGGGAGGCCATGCATGGACAAGTAGACTGTAGTCCAGGAATATGGCAGTTAGATTGTACACATGTAGAAGGAAAAATTATCCTGGTAGCAGTCCATGTAGCCAGTGGCTATATAGAAGCAGAAGTTATCCCAGCAGAAACAGGACAAGAGACAGCATACTTTCTACTAAAATTAGCAGGAAGATGGCCAGTAAAAGTAGTACACACAGACAATGGCAGCAATTTCACCAGCGCTGCAGTTAAAGCAGCCTGTTGGTGGGCAGGTATCCAACAAGAATTTGGGATTCCCTACAATCCCCAAAGCCAAGGAGTAGTGGAATCTATGAATAAAGAATTAAAGAAAATCATAGGGCAGGTCAGGGAGCAAGCTGAACACCTTAAGACAGCAGTACAGATGGCAGTATTCATTCACAATTTTAAAAGAAAAGGGGGGATTGGGGGGTACAGTGCAGGGGAAAGAATAATAGACATAATAGCATCAGATATACAAACTAAAGAACTACAAAAACAAATTACAAAAATTCAAAATTTTCGGGTTTATTACAGGGACAGCAGAGATCCACTTTGGAAAGGACCAGCAAAACTACTTTGGAAAGGTGAAGGGGCAGTAGTAATACAGGACAATAGTGATATAAAGGTAGTACCAAGAAGAAAAGCAAAGATCATCAGGGATTATGGAAAACAGATGGCAGGTGATGATTGTGTGGCAGGTAGACAGGATGAGGAT

>AF457061

TTTCTAGATGGGATAGATAAGGCTCAAGAAGAGCATGACAAATATCACAACAATTGGAGAGCAATGGCTAGCGACTTTAATCTGCCACCCATAGTAGCAAAAGAAATAGTAGCTAGCTGTGATAAATGTCAGCTAAAAGGGGAAGCCATACATGGACAAGTAGACTGTAGTCCAGGAATATGGCAATTAGATTGTACACATTTAGAAGGGAAAATCATCCTGGTAGCAGTCCACGTAGCCAGTGGCTACATGGAAGCAGAGGTTATCCCAGCAGAAACAGGGCAAGAAACAGCATACTATATACTAAAATTAGCAGGAAGATGGCCAGTCAGAGTAATACATACAGACAATGGCAGTAATTTTACTAGTAGTGCAGTTAAGGCAGCCTGTTGGTGGGCAGGTATCCAACAGGAATTTGGAATTCCCTACAATCCCCAAAGTCAGGGGGTAGTAGAATCCATGAATAAAGAATTAAAGAAAATCATAGGACAGGTAAGAGATCAAGCTGAGCACCTTAAGACAGCAGTACAAATGGCAGTATTCATTCACAATTTTAAAAGAAAAGGGGGGATTGGGGGGTACAGTGCAGGGGAAAGAATAATAGACATAATAGCAACAGACATACAAACTAAAGAATTACAAAAACAAATTACAAACATTCAAAAATTTCGGGTTTATTACAGGGACAGCAGAGACCCTATCTGGAAAGGACCAGCCAAACTACTCTGGAAAGGTGAAGGGGCAGTAGTAATACAAGATAACAGTGAGATAAAGGTAGTACCAAGGAGGAAAGCAAAAATCATTAGGGACTATGGAAAACAGATGGCAGGTGATGATTGTATGGCAGGTAGACAGGATGAGGAT

>AF457059

TTCTTGGAGGGGATAGATAAGGCTCAAGAAGAACATGAGAGATACCACAACAATTGGAGAGCAATGGCTAGTGATTTTAACCTGCCACCTGTGGTAGCAAAAGAAATAGTAGCTAGCTGTGATAAATGTCAGCTAAAGGGAGAAGCCTTGCATGGACAAGTAGACTGTAGTCCAGGAATATGGCAATTAGATTGTACACATTTAGAAGGAAAAGTTATCCTGGTAGCAGTCCATGTAGCCAGTGGCTATATAGAAGCAGAGGTTATTCCAGCAGAAACAGGGCAGGAAACAGCTTACTTTCTCTTGAAATTAGCAGGAAGATGGCCAGTAAAAGTAGTACACACAGACAATGGCCCCAATTTCACCAGCGCTGCAGTTAAGGCCGCCTGTTGGTGGGCAGGCATCAAGCAGGAATTTGGAATTCCCTACAATCCCCAAAGTCAAGGAGTAGTAGAATCTATGAATAAAGAATTAAAGAAAATTATAGGACAGGTAAGAGATCAAGCTGAACATCTTAAGACAGCAGTACAAATGGCAGTATTCATCCACAATTTTAAAAGAAAAGGGGGGATTGGGGGGTACAGTGCAGGGGAAAGAATAATAGACATAATAGCATCAGACTTACAAACTAAAGAATTACAAAAACAAATTACAAATATTCAAAAATTTCGGGTTTATTACAGGGACAGCAGAGATCCAATTTGGAAAGGACCAGCAAAACTTCTCTGGAAAGGTGAAGGGGCAGTAGTAATACAGGACAATAGTGAGATAAAGGTAGTACCAAGAAGAAAAGCAAAGATCATCAGGGATTATGGAAAACAGATGGCAGGTGATGATTGTGTGGCAGGTAGACAGGATGAGGAT

>AF457058

TTTTTAGATGGGATAGATAAAGCTCAAGAAGATCATGAAAAATATCACAGTAATTGGAGAGCAATGGCGAGTGATTTTAATCTGCCACCTGTAATAGCAAAGGAAATAGTAGCCAGCTGTGATAAATGTCAGCTAAAAGGGGAAGCCATACATGGACAAGTAGACTGCAGTCCAGGGATATGGCAATTAGATTGTACACATCTAGAAGGAAAAGTAATTCTGGTAGCAGTTCATGTAGCCAGTGGTTATATAGAAGCAGAAGTTATCCCAGCAGAAACAGGACAAGAGACAGCATACTTTATATTAAAATTAGCAGGAAGATGGCCAGTAAAAATAGTACACACAGACAATGGCAGCAATTTCACCAGCGCTGCAGTTAAAGCAGCCTGTTGGTGGGCAGATATCCAACAGGAATTTGGGATTCCCTACAATCCCCAAAGTCAAGGAGTAGTGGAATCTATGAATAAGGAATTAAAGAAAATCATAGGACAAGTAAGAGAGCAAGCTGAACACCTTAAAACAGCAGTACAAATGGCAGTATTCATTCACAATTTTAAAAGAAAAGGGGGGATTGGGGGGTACAGTGCAGGGGAAAGAATAATAGACATAATAGCAACAGACATACAAACTAAAGAATTACAAAAACAAATTACAAAAATTCAAAATTTTCGGGTTTATTACAGGGACAGCAGAGATCCAATTTGGAAAGGACCAGCAAAACTACTCTGGAAAGGTGAAGGGGCAGTAGTAATACAGGACAAGAGTGATATAAAGGTAGTACCAAGAAGAAAGGCAAAGATCATTAGAGATTATGGAAAACAGATGGCAGGTGATGATTGTGTGGCAGATAGACAGAATGAGGAT

>AF457056

TTTTTGGATGGAATAGATAAGGCACAAGAAGACCATGAGAAATATCATAGCAACTGGAGAGCAATGGCTAGTGATTTTGGCCTACCACCTGTGGTAGCAAAAGAAATAGTAGCTAGCTGTGATAAATGCCAGTTAAAAGGAGAAGCTATACATGGACAAGTAGACTGTAGTCCAGGAATATGGCAATTAGATTGTACACATTTAGAAGGAAAAGTTATCCTGGTGGCAGTTCATGTAGCCAGTGGCTATATAGAAGCAGAAGTTATTCCAGCAGAAACAGGGCAGGAAGCAGCCTACTTTATCTTAAAATTAGCAGGAAGATGGCCAGTAAAAATAGTACACACAGACAATGGCAGTAATTTCACCAGCGCTGCAGTTAAAGCAGCCTGTTGGTGGGCAGGTATCCAACAGGAATTTGGGATTCCCTACAATCCCCAAAGTCAAGGAGTAGTGGAATCTATGAATAAAGAATTAAAGAAAATCATAGGACAAGTAAGAGAGCAAGCTGAACACCTTAAAACAGCAGTACAAATGGCAGTATTCATACACAATTTTAAAAGAAAAGGGGGGATTGGGGGGTACAGTGCAGGGGAAAGAATAATAGACATAATAGCAACAGACATACAAACTAAAGAATTACAAAAACAAATTACAAAAATTCAAAATTTTCGGGTTTATTACAGGGACAGCAGAGATCCAATTTGGAAAGGACCAGCAAAACTACTCTGGAAAGGTGAAGGGGCAGTAGTAATACAGGACAATAGTGATATAAAGGTAGTACCAAGAAGAAAAGCAAAAATCATCAGAGACTATGGAAAACAGATGGCAGGTGATGATTGTGTGGCAGGTAGACAGGATGAGGAT

>AF457055

TTTTTAGATGGAATAGATAAAGCTCAAGAAGAACATGAAAGATATCACAGTAATTGGAGAGCAATGGCTAGTGATTTTAATCTGCCACCTATAGTAGCAAAGGAAATAGTAGCCAGCTGTGATAAATGCCAGCTAAAAGGGGAAGCCATACATGGACAAGTAGACTGCAGTCCAGGGATGTGGCAATTAGATTGCACACATCTAGAAGGAAAAGTAATTCTGGTAGCAGTCCATGTAGCCAGTGGCTATATAGAAGCAGAAGTAATCCCAGCAGAAACAGGACAGGAGACAGCCTACTTTCTACTAAAATTAGCAGGAAGATGGCCAGTAAAAGTAGTACACACAGATAATGGCAGCAATTTTACCAGTGCTGCATTTAAAGCAGCCTGTTGGTGGGCAGGTGTCCAACAGGAATTTGGGATTCCCTACAATCCCCAAAGTCAAGGAGTAGTGGAATCTATGAATAGAGAATTAAAGAAAATCATAGGACAAGTAAGGGAGCAGGCTGAACACCTTAAGACAGCAGTACAAATGGCAGTATTCATTCACAATTTTAAAAGAAAAGGGGGGATTGGGGGGTACAGTGCAGGGGAAAGAATAATAGACATAATAGCAACAGACATACAAACTAAAGAATTACAAAAACAAATTACAAAAATTCAAAAATTTCGGGTTTATTACAGGGACAGCAGAGATCCAATTTGGAAAGGACCAGCAAAACTACTCTGGAAAGGTGAAGGGGCAGTGGTGATACAGGACAATAGTGATATAAAGGTAGTACCAAGAAGAAAAGCAAAGATCATTAGGGATTATGGAAAACAGGTGGCAGGTGATGATTGTGTGGCAGGTAGACAGGATGAGGAT

>AF457054

TTCTTAGATGGAATAGATAAAGCTCAAGAAGAGCATGAGAAGTATCACAGCAATTGGAGAGCAATGGCTAGTGAGTTTAATCTGCCACCCATAGTAGCAAAAGAAATAGTAGCTAGCTGTGATAAATGTCAGCTAAAAGGGGAAGCCACACATGGACAAGTAGACTGTAGTCCGGGGATATGGCAATTGGATTGTACACATTTAGAAGGAAAAATCATCCTGGTAGCAGTCCATGTAGCCAGTGGATACATAGAAGCAGAGGTTATCCCAGCAGAAACAGGACAAGAAACAGCATACTACATACTAAAATTAGCAGGAAGATGGCCAGTTAAAGTAATACATACAGACAATGGCAGTAATTTCACCAGTGCTGCAGTTAAGGCAGCCTGTTGGTGGGCAGGTATCCAACAGGAATTTGGAATTCCCTACAATCCCCAAAGTCAGGGAGTAGTAGAATCCATGAATAAGGAATTAAAGAAAATCATAGGACAGGTAAGAGATCAAGCAGAGCACCTTAAGACAGCAGTACAAATGGCAGTATTCATTCACAATTTTAAAAGAAAAGGGGGGATTGGGGGGTACAGTGCAGGGGAAAGAATAATAGACATAATAGCAACAGACATACAAACTAGAGAATTACAAAAGCAAATTATACAAATTCAAAATTTTCGGGTTTATTACAGAGACAGCAGAGACCCTATTTGGAAAGGACCAGCCAAGCTACTCTGGAAAGGTGAAGGGGCAGTAGTAATACAAGATAACAGTGACATAAAGGTAGTACCAAGGAGGAAGGCAAAAATCATTAAGGACTATGGAAAACAGATGGCAGGTGCTGATTGTGTGGCAGGTAGACAGGATGAGGAT

>AF457053

TTCTTAGATGGGATAGATAAAGCTCAAGAGGAACATGAAAGATATCACAGCAATTGGAGAACAATGGCTAGTGATTTTAATCTGCCACCTGTAGTAGCAAAGGAAATAGTAGCCAGCTGTGATAAATGTCAGCTAAAAGGGGAAGCCATGCATGGACAAGTAGACTGCAGTCCAGGGATATGGCAATTAGATTGCACACATCTAGAAGGAAAAATAATTCTGGTAGCAGTTCATGTAGCCAGTGGCTATATAGAAGCAGAAGTTATCCCAGCAGAAACAGGACAAGAGACAGCATACTTTTTACTGAAATTAGCAGGAAGATGGCCAGTAAAAGTAGTACACACAGACAATGGCAGCAATTTCACCAGCGCTGCAGTTAAAGCAGCCTGTTGGTGGGCAGGTATCCAACAGGAATTTGGAATTCCCTACAATCCCCAAAGTCAAGGAGTAGTGGAATCTATGAATAAGGAATTAAAGAAAATCATAGGACAGGTAAGAGAACAAGCTGAACACCTTAAAACAGCGGTACAAATGGCAGTATTCATTCACAATTTTAAAAGAAAAGGGGGGATTGGGGGGTACAGTGCAGGGGAAAGAATAATAGACATAATAGCAACAGACATACAAACTAAAGAACTACAAAAACAAATTACAAAAGTTCAAAATTTTCGGGTTTATTTCAGGGACAGCAGAGATCCACTTTGGAAAGGACCAGCAAAACTACTCTGGAAAGGTGAAGGGGCAGTAGTAATACAGGATAATAGTGAGATAAAAGTAGTACCAAGGAGAAAAGCAAAGATCATCAGGGACTATGGAAAACAGATGGCAGGTGATGATTGTGTGGCAGGTAGACAGGATGAGGAT

>AF457052

TTTCTAGATGGGATAGATAAGGCTCAAGAAGAACATGAAAGATATCACAACAATTGGAGAGCAATGGCTAGTGATTTTAATATCCCACCTATAATAGCAAAGGAAATAGTAGCCAGCTGTGATAAATGTCAACTAAAAGGGGAAGCCATGCATGGACAAGTAGACTGTAGTCCAGGGATGTGGCAATTAGATTGCACACATCTAGAAGGAAAAGTAATTCTGGTAGCAGTCCATGTAGCCAGTGGCTATATAGAAGCAGAAGTTATCCCAGCAGAAACAGGACAGGAGACAGCATACTTTCTGCTAAAATTAGCAGGAAGATGGCCAGTAAAAGTAGTACACACAGACAATGGCAGCAACTTCACCAGCGCTGCATTTAAAGCAGCCTGTTGGTGGGCAAATGTCCAACAAGAATATGGAATTCCCTACAATCCCCAAAGTCAAGGAGTAGTGGAATCTATGAATAAGGAATTAAAGAAAATCATAGGGCAGGTAAGGGACCAAGCTGAACACCTCAAGACAGCAGTACAAATGGCAGTATTCATTCACAATTTTAAAAGAAAAGGGGGGATTGGGGGGTACAGTGCAGGGGAAAGAATAATAGACATAATAGCATCAGACATACAAACCAAAGAACTACAAAAACAAATTACAAAAATTCAAAATTTTCGGGTTTATTACAGGGACAGCAGAGATCCACTTTGGAAAGGACCAGCAAAACTACTCTGGAAAGGTGAAGGGGCAGTAGTAATACAGGACAATAGTGATATAAAGGTAGTACCAAGGAGAAAAGCAAAGATTATCAGGGACTATGGAAAACAGATGGCAGGTGATGATTGTGTGGCAGGTAGACAGGATGAGGAT

>AF457051

TTTTTAGATGGAATAGATAAGGCTCAAGAAGAACATGAAAAATATCACAGTAATTGGAGAGCAATGGCTCATGACTTTAATCTACCACCTATAGTAGCAAAAGAAATAGTAGCTAGCTGTGATAAATGTCAGCTAAAAGGGGAAGCCATGCATGGACAAGTAGACTGTAGTCCAGGAATATGGCAACTAGATTGCACACATTTAGAAGGGAAAGTTATCCTGGTAGCAGTCCATGTAGCCAGTGGCTATATAGAAGCAGAAGTCATCCCAGCAGAAACAGGACAGGAAACAGCATACTTTTTATTAAAGCTAGCAGGAAGATGGCCAGTAAAAGTAATACATACAGACAATGGGCCCAATTTCATCAGTGCAGCAGTTAAGGCCGCCTGCTGGTGGGCAGGTATCAAACAGGAATTTGGGATTCCTTACAATCCCCAAAGTCAAGGAGTAGTGGAATCTATGAATAAAGAATTAAAGAAAATCATAGGGCAGGTAAGAGATCAAGCTGAACACCTTAAGACAGCAGTACAAATGGCAGTATTCATTCACAACTTTAAAAGAAAAGGGGGGATTGGGGACTACAGTGCAGGGGAAAGGATAATAGACATAATAGCAACAGATATACAAACTAAAGAATTACAAAGACAAATTACAAAAATTCAAAATTTTCGGGTTTATTTCAGAGACAGCAGAGATCCAATTTGGAAAGGACCAGCAAAACTCCTCTGGAAAGGTGAAGGGGCAGTAGTAATACAAGACAATAGTGATATAAAGGTAGTACCAAGAAGAAAAGTAAAAATCATTAGGGATTATGGAAAACAGATGGCAGGTGATGATTGTGTGGCAAGTAGACAGGATGAGGAT

>AF361879

TTTCTAGATGGAATAGATAGAGCTCAAGAAGAGCATGAAAAGTATCACAGCAATTGGAGAGCAATGGCTAGTGAGTTTAATCTGCCACCCATAGTAGCAAAAGAAATAGTAGCTAACTGTGATAAATGTCAGTTAAAAGGGGAAGCCATGCATGGACAAGTAGACTGTAGTCCAGGAATATGGCAATTGGATTGTACACATTTAGAAGGAAAAATCATCCTGGTAGCAGTTCATGTCGCCAGTGGCTACATAGAAGCAGAGGTTATCCCAGCAGAAACAGGACAAGAAACAGCATACTACATATTAAAATTAGCAGGAAGATGGCCAGTCAAAGTGATACATACAGACAATGGCAGTAACTTCACCAGTAATGCAGTTAAAGCAGCCTGTTGGTGGGCAGGTATCCAACAGGAATTTGGAATTCCCTACAATCCCCAAAGTCAGGGAGTAGTAGAATCCATGAATAAAGAATTAAAGAAAATCATAGGGCAGGTAAGAGAGCAAGCTGAGCATCTTAAGACAGCAGTACAAATGGCAGTATTCATTCACAATTTTAAAAGAAGAGGGGGGATTGGGGGGTACAGTGCAGGGGAAAGAATAATAGACATAATAGCAACAGACATACAAACTAAAGAATTACAAAAACAAATCTTAAAAATTCAAAATTTTCGGGTTTATTACAGAGACAGCAGAGACCCTATTTGGAAAGGACCAGCAAAACTACTCTGGAAAGGTGAAGGAGCAGTAGTGATACAAGATAATAGTGACATAAAGGTAGTACCAAGGAGGAAAGTAAAAATCATTAAGGACTATGGAAAACAGATGGCAGGTGCTGATTGTGTGGCAGGTAGACAGGATGAGGAT

>AF361878

TTTCTAGATGGGATAGATAAGGCTCAAGAAGAGCATGAAAGATATCACAGCAATTGGAGAACAATGGCTAGTGATTTTAATCTGCCACCTGTAGTAGCAAAGGAAATAGTAGCCAGCTGTGATAAATGTCAGCTAAAAGGGGAAGCCATGCATGGACAAGTAGACTGCAGTCCAGGGATATGGCAATTAGATTGCACACATCTAGAAGGAAAAGTAATTCTGGTAGCAGTTCATGTAGCCAGTGGCTATATAGAAGCAGAAGTTATCCCAGCAGAAACAGGACATGAGACAGCATACTTTCTACTAAAGTTAGCAGCAAGATGGCCAGTAAAAGTAGTACACACAGACAATGGCAGCAATTTCACTAGCGCTGCAGTTAAAGCAGCCTGTTGGTGGGCAAATATCCAACAGGAATTTGGAATTCCCTACAATCCCCAAAGTCAGGGAGTAGTAGAATCCATGAATAAAGAATTAAAGAAAATCATAGGACAGGTAAGAGATCAAGCTGAGCACCTTAAGACAGCAGTACAAATGGCAGTATTCATTCACAATTTTAAAAGAAAAGGGGGGATTGGGGGGTACAGTGCAGGGGAAAGAATAATAGACATAATAGCAACAGACATACAAACTAAAGAATTACAAAAACAAATTACAAAAATTCAAAATTTTCGGGTTTATTACAGAGACAGCAGAGACCCTATGTGGAAAGGACCAGCCAAACTACTCTGGAAAGGTGAAGGGGCAGTAGTCATACAAGATAATAGTGACATCAAGGTAGTACCAAGGAGGAAAGCAAAAATCATTAGGGACTATGGAAAACAGATGGCAGGTGCTGATTGTGTGGCAGGTAGACAGGATGAAGAT

>AF361877

TTTCTAGATGGAATAGATAAGGCTCAAGAAGAGCATGAAAGATATCACAGCAATTGGAGAGCAATGGCTAGTGAGTTTAATCTACCCCCCATAGTAGCAAAAGAAATAGTAGCCAGCTGTGATAAATGTCAGCTAAAGGGGGAAGCCATACATGGACAGGTAGACTGTAGCCCAGGAATATGGCAATTAGATTGTACACATTTAGAAGGAAAAATTATCCTGGTAGCAGTCCATGTAGCCAGTGGCTACATGGAAGCAGAGGTAATCCCAGCAGAAACAGGACAAGAAACAGCATACTTTATATTAAAATTAGCAGGAAGATGGCCAGTAAAAGTAATACATACAGATAATGGCAGTAATTTCACCAGTGCTACAGTTAAGGCAGCCTGTTGGTGGGCAGGTATCCAACAGGAATTTGGAATTCCCTACAATCCCCAAAGTCAGGGAGTAGTAGAATCCATGAATAAAGAATTAAAGAAAATCATAGGGCAGGTAAGAGATCAAGCTGAGCACCTTAAAACAGCAGTACAAATGGCAGTATTCATTCACAATTTTAAAAGAAAAGGGGGGATTGGAGGGTACAGTGCAGGGGAAAGAATAATAGACATAATAGCATCAGACATACAAACTAAAGAATTACAAAAACAAATTATAAAAATTCAAAATTTTCGGGTTTATTACAGAGACAGCAGAGACCCTATTTGGAAAGGACCAGCCAAACTACTCTGGAAAGGTGAAGGGGCAGTAGTAATACAAGACAATAGTGAAATAAAAGTAGTGCCAAGAAGAAAAGCAAAAATCATTAGGGATTATGGAAAACAGATGGCAGGTGATGATTGTGTGGCAAGTAGACAGGATGAGGAT

>AF361876

TTTCTAGATGGAATAGATAAGGCCCAAGACGAGCATGAAAAATATCACAGTAATTGGAGAGCAATGGCTAGTGATTTTAATCTGCCACCTATAGTAGCAAAAGAAATAGTAGCTAGCTGTGATAAATGTCAGCTAAAAGGGGAAGCCATGCATGGACAAGTAGACTGTAGTCCAGGGATATGGCAATTAGATTGTACACATTTAGAAGGAAAAATCATCCTGGTAGCAGTCCATGTAGCCAGTGGCTACATAGAAGCAGAGGTTATTCCAGCAGAAACAGGACAAGAAACAGCATACTTCATCTTAAAATTAGCAGGAAGATGGCCAGTCAAAGTAATACATACAGACAATGGCAGTAATTTCACTAGTGCTGCAGTCAAGGCAGCCTGTTGGTGGGCAGGTATCCAACAGGAATTTGGAATTCCCTACAATCCCCAAAGTCAGGGAGTAGTAGAATCCATGAATAAAGAATTAAAGAAAATCATAGGGCAAGTAAGAGATCAAGCTGAGCACCTTAAGACAGCAGTACAAATGGCAGTATTCATTCACAATTTTAAAAGAAAAGGGGGGATTGGGGGGTACAGTGCAGGGGAAAGAATAATAGACATAATAGCAACAGACATACAAACTAAAGAACTACAAAAACAAATTATAAAAATTCAAAATTTTCGGGTTTATTACAGAGACAGCAGAGACCCTATTTGGAAAGGACCAGCCAAACTACTCTGGAAAGGTGAAGGGGCAGTAGTCATACAAGATAATAGTGACATAAAGGTAGTACCAAGGAGGAAAGTAAAAATCATTAAAGACTATGGAAAACAGATGGCAGGTGCTGATTGTGTGGCAGGTAGACAGGATGAAGAT

>AF361875

TTTCTAGATGGAATAGATAAGGCTCAAGAAGAGCATGAAAAATATCACAGCAATTGGAGAGCAATGGCTAGTGAGTTTAATCTGCCACCCATAGTAGCAAAAGAAATAGTAGCTAGCTGTGACAAATGTCAGCTAAAAGGGGAAGCCATACATGGACAAGTAGACTGTAGTCCAGGGATATGGCAACTAGATTGCACACATTTAGAAGGAAAGATCATCCTGGTAGCAGTCCATGTAGCCAGTGGCTACATGGAAGCAGAGGTTATCCCGGCAGAGACAGGACAAGAAACAGCATACTACATACTAAAATTAGCAGGAAGATGGCCAGTCCAAGTAATACATACAGACAATGGCAGTAATTTCACCAGTGCTGCAGTTAAGGCAGCCTGTTGGTGGGCAGGAATCCAACAGGAATTTGGGATTCCCTACAATCCCCAAAGTCAGGGAGTAGTAGAATCCATGAATAAAGAATTAAAGAAAATCATAGGGCAGGTAAGAGATCAAGCTGAGCACCTTAAGACAGCAGTACAAATGGCAGTATTCATTCACAATTTTAAAAGAAAAGGGGGGATTGGGGGGTACAGTGCAGGGGAAAGAATAATAGATATAATAGCAACAGACATACAAACTAAAGAATTACAAAAGCAAATTACAAAAATTCAAAATTTTCGGGTTTATTACAGAGACAGCAGAGACCCTATTTGGAAAGGACCAGCCAAGCTACTCTGGAAAGGTGAAGGGGCAGTAGTAATACAAGATAACAGTGACATAAAGGTAGTACCAAGGAGGAAAGCAAAGATCATTAAGGACTATGGAAAACAGATGGCAGGTGCTGATTGTGTGGCAGGTAGACAGGATGAGGAT

>AF361874

TTCCTAGATGGAATAGATAAGGCTCAAGAGGAACATGAAAAATATCACAACAATTGGAGAGCAATGGCTAGTGATTTTAATATACCACCCGTAGTAGCAAAAGAAATAGTAGCCAGCTGTGATAAATGTCAACTAAAAGGGGAAGCCATGCATGGACAAGTAGACTGTAGTCCAGGGATATGGCAATTAGACTGTACACATTTAGAAGGAAAAGTCATCCTGGTAGCAGTCCATGTAGCCAGTGGCTACATGGAAGCAGAGGTTATCCCAGCAGAAACAGGACAAGAAACAGCATACTTTCTACTAAAACTAGCAGGAAGATGGCCAGTCAAAGTGGTACATACAGACAATGGCAGTAATTTCACCAGTAATGCAGTCAAGGCAGCCTGTTGGTGGGCAGGTATCCAACAGGAATTTGGAATTCCCTACAATCCCCAAAGTCAGGGAGTAGTAGAATCCATGAATAAAGAATTAAAGAAGATCATAGGGCAGGTAAGAGATCAAGCTGAACACCTTAAGACAGCAGTACAAATGGCAGTATTCATTCACAATTTTAAAAGAAAAGGGGGGATTGGGGGGTATAGTGCAGGGGAAAGAATAATAGACATAATAGCAACAGACATACAAACTAAGGAATTACAAAAACAAATTATAAAAATTCAAAATTTTCGGGTTTATTACAGAGACAGCAGAGACCCTATTTGGAAAGGACCAGCCAAACTACTCTGGAAAGGTGAAGGAGCAGTAGTGATACAAGATAATAGTGACATAAAGGTAGTACCAAGGAGGAAAGTAAAAATCATTAAGGACTATGGAAAACAGATGGCAGGTGCTGATTGTGTGGCAGGTAGACAGGATGAGGAT

>AF361873

TTTTTAGATGGGATAGATAAAGCTCAAGAAGACCATGAAAAATATCACAGCAATTGGAGAGCAATGGCTAGTGATTTTAATCTGCCACCTATAGTAGCAAAGGAAATAGTAGCCAGCTGTGATAAATGTCAACTAAAAGGGGAAGCCATGCATGGACAAGTAGACTGTAGTCCAGGAATGTGGCAATTAGATTGCACACATCTAGAAGGAAAAGTAATCCTGGTAGCAGTTCATGTAGCCAGTGGCTATATAGAAGCAGAAGTTATCCCAGCAGAAACAGGACAGGAGACAGCATACTTTCTGCTAAAATTAGCAGGAAGATGGCCAGTAAAAGTAGTACACACAGACAATGGCAGCAATTTCACCAGCGCTGCATTTAAAGCAGCCTGTTGGTGGGCAAATGTCCAACAGGAATATGGGATCCCCTACAATCCCCAAAGTCAAGGAGTAGTGGAATCTATGAATAAAGAATTAAAGAAAATCATAGGACAAGTAAGAGAGCAAGCTGAACACCTTAAAACAGCAGTACAAATGGCAGTATTCATTCACAATTTTAAAAGAAAAGGGGGGATTGGGGGGTACAGTGCAGGAGAAAGAATAATAGACATAATAGCAACAGACATACAAACTAAAGAATTACAAAAACAAATTACAAAAATTCAAAATTTTCGGGTTTATCACAGGGACAGCAGAGATCCAATTTGGAAAGGACCAGCAAAACTACTCTGGAAAGGTGAAGGGGCGGTAGTAATACAGGACAATAGTGATATAAGGATAGTACCAAGAAGGAAAGCAAAGATCATTAGGGATTATGGAAAACAGATGGCAGGTGATGATTGTGTGGCAGATAGACAGAATGAGGAT

>AF361872

TTTTTAGATGGGATAGATAAAGCTCAAGAAGAGCATGAAAGATATCACAGCAATTGGAGAGCAATGGCTAGTGATTTTAATCTGCCACCTATAGTAGCAAAGGAAATAGTAGCCAGCTGTGATAAATGTCAGCTAAAAGGGGAAGCCATGCATGGACAAGTAGACTGCAGTCCAGGGATATGGCAATTAGATTGCACACATCTAGAAGGAAAAGTAATTCTGGTAGCAGTTCATGTAGCCAGTGGCTATATAGAAGCAGAAGTTATCCCAGCAGAAACAGGACAAGAGACAGCATACTTTATACTAAAATTAGCAGGAAGATGGCCAGTAAAAGTAGTACACACAGACAATGGTAGCAATTTCACCAGCGCTGCAGTTAAAGCTGCCTGTTGGTGGGCAGGTGTCCAACAGGAATTTGGGATTCCCTACAATCCCCAAAGTCAAGGAGTAGTGGAATCTATGAATAAGGAATTAAAGAAAATCATAGGACAAGTAAGAGATCAAGCTGAACACCTTAAAACAGCAGTACAAATGGCAGTATTCATTCACAATTTTAAAAGAAAAGGGGGGATTGGGGGGTACAGTGCAGGGGAAAGAATAATAGACATAATATCAACAGACATACAAACTAAAGAATTACAAAAACAAATTATAAAAATTCAAAATTTTCGGGTTTATTACAGGGACAGCAGAGATCCAATTTGGAAAGGACCAGCAAAACTACTCTGGAAAGGTGAAGGGGCAGTAGTCATACAGGATAATAGTGATATAAAGGTAGTACCCAGAAGAAAAGTAAAGATCATAAGGGACTATGGAAAACAGATGGCAGGTGATGATTGTGTGGCAGGTAGACAGGATGAGGAT

>AF361871

TTTCTAGATGGGATAGATAAAGCTCAAGAAGAACATGAGAAGTATCACAGCAATTGGAGAGCAATGGCTAGTGAGTTTAATCTGCCACCCATAGTAGCAAAAGAAATAGTAGCTAGCTGTGATAAATGTCAGCTAAAAGGGGAAGCCATACATGGACAAGTAGACTGTAGTCCAGGGATATGGCAATTAGATTGTACACATTTAGAGGGAAAAATCATCCTGGTAGCAGTCCATGTAGCCAGTGGCTACATAGAAGCAGAGGTTATCCCAGAAGAAACAGGACAGGAAACAGCATACTATATACTAAAATTAGCAGGAAGATGGCCAGTCAAAGTGATACATACAGACAATGGTAGAAATTTTACCAGTAATGCAGTTAAGGCAGCCTGTTGGTGGGCAGGTATCCAACAGGAATTTGGGATTCCCTACAATCCCCAAAGTCAGGGAGTAGTAGAATCCATGAATAAAGAATTAAAGAAAATCATAGGGCAGGTAAGAGATCAAGCTGAGCACCTTAAGACAGCAGTACAAATGGCAGTATTCATTCACAATTTTAAAAGAAGAGGGGGGATTGGGGGATACAGTGCAGGGGAAAGAATAATAGACATAATAGCAACAGATATACAAACTAAAGAATTACAAAAGAATATTACAAAAATTCAAAATTTTCGGGTTTATTACAGAGACAGCAGAGACCCTATTTGGAAAGGACCAGCCAAACTACTCTGGAAAGGTGAAGGGGCAGTAGTAATACAAGATAACAGTGACATAAAGGTAGTACCAAGGAGGAAAGTAAAAATCATTAGGGACTATGGAAAACAGATGGCAGGTGATGATTGTGTGGCAGGTAGACAGAATGAGGAT

>AF443115

TTTCTAGATGGAATAGATAAGGCTCAAGAAGAACATGAAAAATATCACAGCAATTGGAGGTCAATGGCTAGTGATTTCAATCTGCCACCCATAGTAGCAAAAGAAATAGTGGCCAGCTGCGATAAATGTCAGCTAAAAGGGGAAGCCATGCATGGACAAGTAGACTGTAGCCCAGGGGTATGGCAATTAGATTGTACACATTTAGAAGGAAAAATCATACTAGTGGCAGTCCATGTAGCCAGTGGCTACATAGAGGCAGAAGTCATTCCAGCAGAAACAGGACAAGAAACAGTATATTTTATACTAAAATTAGCAGGGAGATGGCCAGTCAAAGCAATACATACAGACAATGGCAGCAATTTCACCAGTGCTGCGGTTAAGGCAGCCTGTTGGTGGGCAGGTATTAATCAGGAATTTGGAATTCCCTACAATCCCCAAAGTCAGGGAGTAGTAGAATCCATGAATAAAGAATTAAAGAAAATCATAGGGCAGGTAAGAGAACAAGCTGAGCACCTTAAGACAGCAGTACAAATGGCAGTATTCATTCACAATTTTAAAAGAAAAGGGGAGATTGGGGGGTACAGTGCAGGGGAAAGAATAATAGACATAATAGCAACAGATATACAAACTAAAGAACTACAAAAACAAATTACAAAAATTCAAAATTTTCGGGTTTATTACAGAGACAGCAGAGACCCTATTTGGAAAGGACCAGCCAAACTACTCTGGAAAGGTGAAGGGGCAGTAGTGATACAAGATAATAGTGACATAAAAGCAGTACCAAGGAGGAAAGCAAAAATCATTAAGGACTATGGACAACAGATGGCAGGGGCTGATTGTGTGGCAGGTAGACAGGATGAGAAT

>AF443114

TTTCTAGATGGAATAGATAAGGCTCAAGAAGAGCATGAAAAGTATCACAGCAATTGGAGAGCAATGGCTAGTGAATTTAATCTGCCACCCATAGTAGCAAAAGAAATAGTAGCTAGCTGTGATAAATGTCAACTAAAAGGGGAAGCCATGCATGGACAAGTAGACTGTAGTCCCGGGATATGGCAATTAGATTGTACACATTTAGAAGGAAAAATCATCCTGGTAGCAGTCCATGTAGCCAGTGGCTACATAGAAGCAGAGGTTATCCCAGCAGAAACAGGACAAGAAACAGCATACTATATACTAAAATTAGCAGGAAGATGGCCAGTCAAAGTAATACACACAGACAATGGCAGTAATTTCACCAGTACTGCAGTTAAGGCTGCCTGTTGGTGGGCAGGTATCAAACAGGAATTTGGAATTCCCTACAATCCCCAAAGTCAGGGAGTAGTAGAATCCATGAATAAAGAATTAAAGAAAATCATAGGGCAGGTAAGAGATCAAGCTGAGCACCTTAAGACAGCAGTACAAATGGCAGTATTCATTCACAATTTTAAAAGAAGAGGGGGGATTGGGGGGTACAGTGCAGGGGAAAGAATAATAGACATAATAGCAACAGACATACAAACTAAAGAACTACAGAAACAAATTATAAAAATTCAAAATTTTCGGGTTTATTACAGAGACAGCAGAGACCCTATCTGGAAAGGACCAGCCAAACTTCTCTGGAAAGGTGAAGGGGCAGTAGTGATACAAGATAATAGTGACATAAAGGTAGTACCAAGAAGGAGAGCAAAAATCATTAGGGACTATGGAAAACAGATGGCAGGTGCTGATTGTGTGGCAGATAGACAGGATGAGGAT

>AF443113

TTTCTAGATGGAATAGATAAGGCCCAAGAAGAGCATGAAAAATATCACAGCAATTGGAGAGCAATGGCTAGTGAGTTTAATCTGCCACCCGTAGTAGCAAAAGAAATAGTAGCCAGCTGTGATAAATGTCAGCTAAAAGGGGAAGCCATACATGGACAAGTAGACTGTAGTCCAGGGATATGGCAATTAGATTGTACACATTTAGAAGGAAAAGTTATACTGGTAGCAGTCCACGTAGCCAGTGGCTACATAGAAGCAGAGGTTATCCCAGCAGAAACAGGACAAGAAACAGCATACTATATATTAAAGTTAGCAGGACGATGGCCTGTCAAAGTAATCCATACAGACAATGGCAGTAATTTCACCAGTAATGCAGTTAAGGCAGCCTGTTGGTGGGCAGGTATCCAACAGGAATTTGGAATTCCCTACAATCCCCAAAGTCAGGGAGTAGTAGAATCCATGAATAAAGAACTAAAGAAAATCATAGGGCAGGTAAGGGATCAAGCTGAGCACCTTAAGACAGCAGTACAAATGGCAGTATTCATTCACAATTTTAAAAGAAAAGGGGGGATTGGGGGGTACAGTGCAGGGGGAAGAATAATAGACATAATAGCAACAGACATACAAACTAAAGAATTACAAAAACAAATTATAAAAATTCAAAATTTTCGGGTTTATTACAGAGACAGCAGAGACCCTATTTGGAAAGGACCAGCCAAACTACTCTGGAAAGGCGAAGGGGCAGTAGTAATACAAGATAACAGTGACATAAAGGTAGTACCAAGGAGGAAAGTAAAAATCATTAGGGACTATGGAAAACAGATGGCAGGTGATGATTGTGTGGCAGGTAGACAGGATGAAGAT

>AF443112

TTTCTAGACGGAATAGATAAGGCCCAAGAAGAACATGAAAAATATCACAGCAATTGGAGAGCAATGGCTAGTGAGTTTAATCTACCACCCATAGTAGCAAAAGAAATAGTAGCTAGCTGTGATAAATGTCAGCTAAAAGGAGAAGCCATACATGGACAAGTAGACTGTAGTCCAGGGATATGGCAATTAGATTGTACACATTTAGAAGGAAAAACCATCCTGGTAGCAGTCCATGTAGCTAGTGGCTACATAGAAGCAGAAGTTATCCCAGCAGAAACAGGACAAGAAACAGCATACTATATACTAAAATTAGCAGGAAGATGGCCAGTCAAAGTAATACATACAGACAATGGCAGTAATTTCACCAGTGCTGCGGTAAAAGCAGCCTGTTGGTGGGCAGGTATCCAACAGGAATTTGGAATTCCCTACAATCCCCAAAGTCAGGGAGTAGTAGGATCCATGAATAAAGAATTAAAGAAAATCATAGGGCAGGTAAGAGATCAGGCTGAGCACCTTAAGACAGCAGTACAAATGGCAGTATTCATTCACAATTTTAAAAGAAAAGGGGGGATTGGGGGGTACAGTGCAGGGGAAAGAATAATAGACATAATAGCAACAGACATACAAACCAAAGAACTACAAAACCAAATTATAAAAATTCAAAATTTTCGGGTTTATTACAGAGACAGCAGAGACCCTATTTGGAAAGGACCAGCCAAACTACTCTGGAAAGGTGAAGGGGCGGTAGTAATACAAGATAAAGGAGACATAAAGGTAGTACCAAGGAGGAAAGCAAAAATCATTAAGGACTATGGAAAACAGATGGCAGGTGCTGATTGTGTGGCAGGTAGACAGGATGAAGAT

>AF443111

TTTCTAGATGGAATAGATAAGGCTCAAGAAGAGCATGAAAAGTATCACAGCAATTGGAGAGCAATGGCTAGTGAGTTTAATCTGCCACCCATAGTAGCAAAAGAAATAGTAGCTAGCTGTGATAAATGTCAGCAAAAAGGGGAAGCCATACATGGACAAGTAGACTGTAGTCCAGGGATATGGCAATTAGATTGTACACATTTAGAAGGGAAAATCATCCTGGTAGCAGTTCATGTAGCCAGTGGCTACATAGAAGCAGAGGTTATCCCAGCAGAAACAGGACAAGAAACAGCATATTATATACTAAAATTAGCAGGAAGATGGCCGGTCAAAGTGATACATACAGACAATGGCAGTAATTTTACCAGTAGTGCAGTCAAAGCAGCCTGTTGGTGGGCAGGTACCCAACAGGGATTTGGAATTCCCTACAATCCCCAAAGCCAGGGAGTAGTAGAATCCATGAATAAAGAATTAAAGAAAATCATAGGGCAGGTAAGAGATCAAGCTGAGCACCTTAAGACAGCAGTACAAATGGCAGTATTCATTCACAATTTTAAAAGAAAAGGGGGGATTGGGGGGTACAGTGCAGGGGAAAGAATAATAGACATAATAGCAACAGACATACAAACTAAAGAATTACAAAACCAAATTACAAAAATTCAAAATTTTCGGGTTTATTACAGAGACAGCAGAGACCCCATTTGGAAAGGACCAGCCAAACTACTCTGGAAAGGTGAAGGGGCAGTAGTAATACAAGATAATAGTGACATAAAGGTAGTACCAAGGAGGAAAGCAAAAATCATTAAGAACTATGGAAAACAGATGGCAGGTGCTGATTGTGTGGCAGGTAGACAGGATGAGGAT

>AF443110

TTTCTAGATGGAATAGATAAGGCTCAAGAAGAACATGAAAAGTATCACAGCAACTGGAGAGCAATGGCTAGTGAGTTTAATCTGCCACCTATAGTAGCAAAAGAAATAGTAGCTAGCTGTGATAAGTGTCAGCTAAAAGGAGAAGCCACACATAGACAAGTAGATTGTAGTCCAGGGATATGGCAATTAGATTGTACACATTTAGAAGGAAAAGTCATCCTGGTAGCAGTCCATGTAGCCAGTGGCTATATAGAAGCAGAGGTTATCCCAGCAGAAACAGGACAAGAAACAGCATACTATATACTAAAACTAGCAGGAAGGTGGCCAGTCAAAGTAATACATACAGACAATGGCAGTAATTTCACCAGTACGGTAGTTAAGGCAGCCTGCTGGTGGGCAGGTATCCAACAGGAATTTGGAATTCCCTACAATCCCCAAAGTCAGGGAGTAGTAGAATCCATGAATAAAGAATTAAAGAAAATCATAGGGCAGGTAAGAGATCAAGCTGAGCACCTTAAGACAGCAGTACAAATGGCAGTATTCATCCACAATTTTAAAAGAAAAGGGGGGATTGGGGGGTACAGTGCAGGGGAAAGAATAATAGATATAATAGCAACAGACATACAAACTAAAGAATTACAAAAACAAATTACGAAAATTCAAAATTTTCGGGCTTATTACAGAGACAGCAGAGACCCTATTTGGAAAGGACCAGCCAAACTACTCTGGAAAGGTGAAGGAGCAGTAGTAATACAAGATAATAGTGACATAAAGGTAGTACCAAGGAGGAAAGCAAAAATCATTAAGGACTATGGAAAACAGATGGCAGGTGCTGATTGTGTGGCAGGTAGACAGGATGAGGAT

>AF443109

TTTCTAGATGGAATAGATAAGGCTCAAGAAGAACATGAGAAATATCACAGCAATTGGAGAGCAATGGCTAGTGAGTTTAATCTGCCACCCATAGTAGCAAAAGAAATAGTAGCTAGCTGTGATAAGTGTCAGCTAAAAGGAGAAGCCATGCATGGACAAGTAGACTGTAGTCCAGGAATATGGCAATTAGATTGTACACATTTAGAAGGAAAAATCATCCTGGTAGCAGTCCATGTAGCCAGTGGCTACATAGAAGCAGAAGTTATCCCAGCAGAAACAGGACAAGAAACAGCATACTACATACTAAAGTTAGCAGGAAGATGGCCAGTCAAAGTAATACATACAGACAATGGCAGTAATTTCACCAGTGCTGCAGTTAAGGCAGCCTGTTGGTGGGCAGGTATCCAACAGGAATTTGGAATTCCCTACAATCCCCAAAGTCAGGGAGTAGTAGAATCCATGAATAAAGAATTAAAGAAAATTATAGGGCAGATAAGAGATCAAGCTGAACACCTTAAGACAGCAGTACAAATGGCAGTATTCATTCACAATTTTAAAAGAAAAGGGGGGATTGGGGGGTATAGTGCAGGGGAAAGAATAATAGACATAATAGCAACAGACATACAAACTAAAGAATTACAAAACCAAATTACAAAAATTCAAAATTTTCGGGTTTATTACAGAGACAGCAGAGATCCTATTTGGAAAGGACCAGCCAAACTACTCTGGAAAGGTGAAGGAGCAGTAGTAATACAAGATAACAGTGACATAAAGGTAGTACCAAGGAGGAAAGTAAAAATCATTAAGGACTATGGAAAACAGATGGCAGGCGCTGATTGTGTGGCAGGTAGACAGGATGAGGAT

>AF443108

TTTCTAGATGGAATAGATAAGGCTCAAGAAGACCATGAAAAATATCACAATAATTGGAGAGCAATGGTTAGTGAGTTTAATCTGCCACCCATAGTAGCAAAAGAAATAGTAGCTAGCTGTGATAAATGTCAGCTAAAAGGAGAAGCTACACATGGACAAGTAGACTGTAGTCCAGGGATATGGCAATTAGATTGTACACATTTAGAAGGAAAAATCATCCTGGTAGCAATCCATGTAGCCAGTGGCTACATGGAAGCAGAGGTTACTCCAGCAGAAACAGGTCAAGAAACAGCATACTTTATACTAAAATTAGCAGGAAGATGGCCAGTCAAAGTAATACATACAGACAATGGCAGTAATTTCACCAGCAACGCAGTTAAGGCCGCCTGTTGGTGGGCAGGTATCCAACAGGAATTTGGAATTCCCTACAATCCCCAAAGTCAGGGAGTAGTAGAATCCATGAATAAAGAATTAAAGAAAATCATAGGACAAGTAAGAGATCAAGCTGAGCACCTTAAGACAGCAGTACAAATGGCAGTATTCATTCACAATTTTAAAAGAAAAGGGGGGATTGGGGGGTACAGTGCAGGGGAAAGAATAATAGACATAATAGCAACAGACATACAAACTAGAGAATTACAAAAACAAATTATACAAATTCAAAATTTTCGGGTTTATTACAGAGACAGCAGAGACCCTATTTGGAAAGGACCAGCCAAGCTACTCTGGAAAGGTGAAGGGGCAGTAGTAATACAAGATAACGGTGACATAAAGGTAGTACCAAGGAGGAAAGCAAAAATCATTAAGGACTATGGAAAACAGATGGCAGGTGCTGATTGTGTGGCAAGTAGACAGGATGAAAAT

>AF443107

TTTTTAGATGGTATAGATAAGGCTCAAGAAGAGCATGAAAAATATCACAACAATTGGAGAGCAATGGCTAGTGATTTTAATCTGCCACCCATAGTAGCAAAAGAAATAGTAGCTAGCTGTGATAAATGTCAGCTAAAAGGGGAGGCCATACACGGACAAGTAGACTGTAGTCCAGGGATATGGCAATTAGATTGCACACATTTAGAAGGAAAAGTCATCCTGGTAGCAGTCCACGTAGCCAGTGGCTACATGGAAGCAGAAGTTATCCCAGCAGAAACAGGACAAGAAACAGCATACTTTATACTAAAATTAGCAGGAAGATGGCCTGTCAAAGTAATACATACAGACAATGGCAGTAATTTCACCAGTACTACAGTTAAGGCAGCCTGTTGGTGGGCAGGTATCCAACAGGAATTTGGAATTCCCTACAATCCCCAAAGTCAGGGAGTAGTAGAATCCATGAATAAAGAATTAAAGAAAATAATAGGACAAGTAAGAGATCAAGCTGAGCACCTTAAGACAGCAGTACAAATGGCAGTATTCATTCACAATTTTAAAAGAAGAGGGGGGATTGGGGGGTACAGTGCAGGGGAAAGAATAATAGACATAATAGCAACAGACATACAAACTAAAGAATTACAAAAACAAATTACAAAAATTCAAAATTTTCGGGTTTATTACAGAGACAGCAGAGACCCTATTTGGAAAGGACCAGCCAAACTACTCTGGAAAGGTGAAGGGGCAGTAGTGATACAAGATAACAGTGACATAAAGGTAGTACCAAGGAGGAAAGTAAAAATCATTAAGGACTATGGAAAACAGATGGCAGGTGCTGATTGTGTGGCAGGTAGACAGGATGAAGAT

>AF443106

TTTCTAGATGGAATAGATAAGGCCCAAGAAGAGCATGAAAAGTATCACAGCAATTGGAGAGCAATGGCTAGTGAGTTTAATCTGCCACCCATAGTAGCAAAAGAAATAGTAGCTAGCTGTGATAAATGTCAGCTAAAAGGGGAAGCCATACATGGACAAGTAGACTGTAGTCCAGGGATATGGCAATTAGATTGTACACATTTAGAAGGAAAAATCATCCTGGTAGCAGTCCATGTAGCCAGTGGCTACATAGAAGCAGAGGTTATCCCAGCAGAAACAGGACAAGAAACAGCATACTATATACTAAAATTAGCAGGAAGGTGGCCAGTCAAAGTAATACACACAGACAATGGTAGTAATTTCACCAGTGCTGCAGTTAAGGCAGCCTGTTGGTGGGCGGGCATTCAACAGGAATTTGGAATTCCCTACAGTCCCCAAAGTCAGGGAGTAGTAGAATCCATGAATAAAGAATTAAAGAAAATTATAGGGCAGGTAAGAGATCAAGCGGAGCACCTTAAGACAGCAGTACAAATGGCAGTATTCATTCACAATTTTAAAAGAAAAGGGGGGATTGGGGGGTACAGTGCAGGGGAAAGAATAATAGACATCATAGCAACAGATATACAAACTAAAGAACTACAAAAACAAATTATAAAAATTCAAAATTTTCGGGTTTATTACAGAGACAGCAGAGACCCTATTTGGAAAGGACCAGCCAAACTACTCTGGAAAGGTGAAGGGGCGGTAGTACTACAAGATAATAGTGACATAAAGGTAGTACCAAGGAGGAAAGTAAAAATCATTAAGGACTATGGAAAACAGATGGCAGGTGCTGATTGTGTGGCAGGTGGACAGGATGAGAAT

>AF443105

TTTCTAAATGGAATAGATAAGGCTCAAGAAGAGCATGAAAAATATCACAGCAATTGGAGAGCAATGGCTAATGAGTTTAATTTACCACCCGTAGTAGCAAAAGAAATAGTAGCTAGCTGTGATAAATGTCAGCTAAAAGGGGAAGCTACACATGGACAAGTAGACTGTAGTCCAGGGATATGGCAATTAGATTGTACACATTTAGAAGGAAAAATCATCCTGGTAGCAGTCCATGTAGCCAGTGGCTACATAGAAGCAGAGGTTATCCCAGCAGAAACAGGACAAGAAACAGCATACTTTATACTAAAATTAGCAGGAAGATGGCCAGTCAAAGTAATACATACAGACAATGGCAGTAATTTCACCAGCGCTGCAGTTAAGGCAGCCTGTTGGTGGGCAGGTATCCAACAGGAATTTGGAATTCCCTACAATCCCCAAAGTCAGGGAGTAGTAGAATCCATGAATAAAGAATTAAAGAAAATCATAGGACAGGTAAGAGATCAAGCTGAGCACCTTAAAACAGCAGTACAAATGGCAGTATTCATTCACAATTTTAAAAGAAAAGGGGGGATTGGGGGGTACAGTGCAGGGGAAAGAATAATAGACATAATAGCAACAGACATACAAACTAAAGAATTACAAAAACGAATTATACAAATTCAAAATTTTCGGGTTTATTACAGAGACAGCAGAGACCCTATTTGGAAAGGACCAGCCAAACTACTCTGGAAAGGGGAAGGAGCAGTAGTAATACAAGATAACAGTGACATAAAGGTAGTACCAAGGAGGAAAGCAAAAATCATTAAGGACTATGGAAAACAGATGGCAGGTGCTGATTGTGTGGCAAGTAGACAGGATGAAGAT

>AF443104

TTTCTAGATGGAATAGATAAGGCTCAAGAAGACCATGAAAAATATCACAGCAATTGGAGAGCAATGGCTAATGAATTTAATCTGCCACCCATAGTAGCAAAAGAAATAGTAGCTAGCTGTGATAAATGTCAGCTAAAAGGAGAAGCCATACATGGACAAGTAGACTGTAGTCCAGGGATATGGCAATTAGACTGTACACATTTAGAAGGAAAAATCATCCTGGTAGCAGTCCATGTAGCCAGTGGCTACATAGAAGCAGAAGTTATCCCAGCAGAAACAGGACAAGAGACAGCATACTATATACTAAAATTAGCAGGAAGATGGCCTGTCAAAGTAATACACACAGACAATGGCAGTAATTTCACCAGTGCTGCAGTTAAGGCAGCCTGTTGGTGGGCAGGTATCCAACAGGAATTTGGAATTCCCTACAATCCCCAAAGTCAGGGAGTAGTAGAATCCATGAATAAAGAATTAAAGAAAATAATAGGACAAGTAAGAGATCAAGCTGAGCACCTTAAGACAGCAGTACAAATGGCAGTATTCATTCACAATTTTAAAAGAAAAGGGGGGATTGGGGGGTACAGTGCAGGGGAGAGAATAATAGATATAATAGCAACAGACATACAAACTAAAGAGTTACAAAAACAAATTATAAAAATTCAAAATTTTCGGGTTTATTACAGAGACAGCAGAGACCCTATTTGGAAAGGACCAGCCAAACTACTCTGGAAAGGTGAAGGGGCAGTAGTAATACAAGATAACAGTGACATAAAGGTAGTACCAAGGAGGAAAGTAAAAATCATTAAGGACTATGGAAAACAGATGGCAGGTGCTGATTGTGTGGCAGGTAGACAGGATGAAGAT

>AF443103

TTTCTAGATGGAATAGATAAGGCTCAAGAAGACCATGAAAGATATCACAGCAATTGGAGAACAATGGCTAGTGAGTTTAACCTGCCACCCATAGTAGCAAAAGAAATAGTAGCCAGCTGTGATAAATGTCAGTTAAAAGGGGAAGCCATACATGGACAAGTAGACTGTAGTCCAGGGATATGGCAATTAGATTGTACACATTTAGAAGGAAAAATCATCCTGGTAGCAGTCCACGTAGCCAGTGGCTACGTGGAAGCAGAGGTTATACCAGCAGAAACAGGACAAGAAACAGCATATTTTATACTAAAATTAGCAGGAAGATGGCCAGTCAAAATAATACATACAGACAATGGAAGTAATTTCACCAGTGCTGCAGTTAAGGCAGCCTGTTGGTGGGCAGGCATCCAACAGGAATTTGGGATTCCCTACAATCCCCAAAGTCAGGGAGTAGTAGAATCCATGAATAAAGAATTAAAGAAAATCATAGGGCAGGTAAGAGATCAAGCTGAGCACCTTAAGACAGCAGTACAAATGGCAGTATTCATTCACAATTTTAAAAGAAAAGGGGGGATTGGGGGGTACAGTGCAGGGGAAAGAATAATAGACATAATAGCAACAGACATACAAACCAGAGAATTACAAAAACAAATCATAAAAATTCAAAATTTTCGGGTTTATTACAGAGACAGCAGAGACCCTATTTGGAAAGGACCAGCCAAACTCCTCTGGAAAGGTGAAGGGGCAGTAGTAATACAAGATAATAGTGACATAAAGGTAGTACCAAGGAGGAAAGTAAAAATCATTAAGGACTATGGAAAACAGATGGCAGGGGCTGATTGTGTGGCAGGTAGACAGGATGAGGAT

>AF443102

TTTCTAGATGGAATAGATAAGGCTCAAGAAGAGCATGAAAAATATCACAGCAATTGGAGAGCAATGGCTAGTGAGTTTAATCTGCCACCCATAGTAGCAAAAGAAATAGTAGCTAGCTGTGACAAGTGTCAGCTAAAAGGGGAAGCCATACATGGACAAGTAGACTGTAGTCCAGGAATATGGCAATTAGATTGTACACATTTAGAAGGAAAAATCATCATAGTAGCAGCCCATGTAGCCAGTGGCTACATAGAAGCAGAAGTTATCCCAGCAGAAACAGGACAAGAAACAGCATACTTTATACTAAAGTTAGCAGGAAGATGGCCAGTCAAAGTAATACATACAGACAATGGCAGTAATTTCACCAGTGCTGCAGTTAAGGCAGCCTGCTGGTGGGCAGGTATCCAACAGGAATTTGGAATTCCCTACAATCCCCAAAGTCAGGGAGTAGTAGAATCCATGAATAAAGAATTAAAGAAAATCATAGGGCAGGTAAGAGATCAAGCTGAGCACCTTAAGACAGCAGTACAAATGGCAGTATTCATTCACAATTTTAAAAGAAAAGGGGGGATTGGGGGGTACAGTGCAGGGGAAAGAATAATAGACATAATAGCAACAGACATACAAACTAAAGAATTACAAAAACAAATTACAAAAATTCAAAATTTTCGGGTTTATTACAGAGACAGCAGAGACCCTATTTGGAAAGGACCAGCCAAGCTACTCTGGAAAGGTGAAGGAGCAGTAGTAATACAAGATAATAGTGACATAAAGGTAGTACCAAGGAGGAAAGTAAAAATCATTAAGGACTATGGAAAACAGATGGCAGGCGCTGATTGTGTGGCAGGTAGACAGGATGAGGAT

>AF443101

TTTCTAGATGGAATAGATAAGGCTCAAGAAGAACATGAAAAGTATCACAGCAATTGGAGAGCAATGGCTAGTGAATTTAACCTGCCACCTGTAGTAGCAAAAGAAATAGTAGCTAGCTGTGATAAATGTCAGCTAAAAGGGGAAGCCATGCATGGACAAGTAGACTGTAGTCCAGGGATATGGCAATTAGATTGTACACATTTAGAAGGAAAAATCATCCTGGTAGCCGTCCATGTAGCCAGTGGCTATATAGAAGCAGAAGTTATCCCAGCAGAAACAGGACAAGAAACAGCATACTATATACTAAAATTAGCAGGAAGATGGCCAGTCAAAGTAATACATACAGACAATGGCAGTAATTTCACCAGTACTGCAGTTAAGGCAGCCTGCTGGTGGGCAGGTATCCAACAGGAATTTGGAATTCCCTACAATCCCCAAAGTCAGGGAGTGGTAGAATCCATGAATAAAGAATTAAAGAAAATTATAGGGCAGGTAAGAGATCAAGCTGAGCACCTTAAGACAGCAGTACAAATGGCAGTATTCATTCACAATTTTAAAAGAAAAGGGGGGATTGGGGGGTACAGTGCAGGGGAAAGAATAATAGATATAATAGCAACAGATATACAGACTAAAGAATTACAAAAACAAATTACAAACATTCAAAAATTTCGGGTTTATTACAGAGACAGCAGAGACCCTATTTGGAAAGGACCAGCCAAACTACTCTGGAAAGGTGAAGGGGCAGTAGTAATACAAGATAACAGTGACGTAAAGGTAGTACCAAGGAGGAAAGCAAAGATCATTAGGGACTATGGAAAACAGATGGCAGGTGCTGATTGTGTGGCAGATAGACAGGATGAAGAT

>AF443100

TTTCTAGATGGGATAGATAAGGCTCAAGAAGAGCATGAAAAATATCACAGCAATTGGAGAGCTATGGCTAGTGAGTTTAATCTGCCACCCGTAGTAGCAAAAGAAATAGTAGCCAGCTGTGATAAATGTCAGCTAAAAGGGGAAGCCATACATGGACAAGTAGACTGTAGTCCAGGGATATGGCAATTAGACTGTACACATTTAGAAGGAAAAATCATCCTGGTAGCAGTCCATGTAGCCAGCGGCTACATAGAAGCAGAGGTTACTCCAGAAGAAACAGGACAAGAAACAGCATATTATATACTAAAATTAGCAGGAAGATGGCCAGTCAAAATAATACATACAGACAATGGCAGAAATTTCACCAGTGCTGCAGTTAAGGCAGCCTGTTGGTGGGCAGGTATCCAACAGGAATTTGGAATTCCCTACAATCCTCAAAGTCAGGGAGTAGTAGAATCCATGAATAAAGAATTAAAGAAAATCATAGGGCAAGTAAGAGATCAAGCTGAGCACCTTAAAACAGCAGTACAAATGGCAGTATTCATTCACAATTTTAAAAGAAAAGGGGGGATTGGGGGGTACAGTGCAGGGGAAAGAATAATAGACATAATAGCAACAGACATACAAACTAGAGAATTACAAAAACAAATTATAAAAATTCAAAATTTTCGGGTTTATTACAGAGACAGCAGAGACCCTATTTGGAAAGGACCAGCCAAACTACTCTGGAAAGGTGAAGGAGCAGTAGTAATACAAGATAAGAGTGACATAAAGGTAGTACCAAGGAGGAAAGTAAAAATCATTAGGGATTATGGAAAACAGATGGCAGGTGCTGATTGTGTGGCAGATAGACAGGATGAAGAT

>AF443099

TTTCTAGATGGAATAGATAAGGCTCAAGAGGAGCATGAAAAGTATCACAGCAATTGGAGAGCAATGGCTAATGAGTTTAATTTGCCACCTATAGTAGCAAAAGAGATAGTAGCTAGCTGTGATAAATGTCAGCTAAAAGGGGAAGCCATACATGGGCAAGTAGACTGTAGCCCAGGGATATGGCAATTAGATTGTACACATTTAGAAGGAAAAATCATCTTGGTAGCAGTCCATGTAGCCAGTGGCTACATGGAAGCAGAAGTTATCCCAGCAGAAACAGGACAAGAAACAGCATACTATATACTAAAATTAGCAGGAAGATGGCCAGTCAAAGTAATACATACAGACAATGGCAGTAATTTCACCAGTGCTGCAGTTAAGGCAGCCTGCTGGTGGGCAGGTATCCAACAGGAATTTGGAATTCCCTACAATCCCCAAAGTCAGGGAGTAGTAGAATCCATGAATAAAGAATTAAAGAAAATCATAGGACAGGTAAGAGATCAAGCTGAGCACCTTAAGACAGCAGTACAAATGGCAGTATTCATTCACAATTTTAAAAGAAAAGGGGGGATTGGGGGGTACAGTGCAGGGGAAAGAATAATAGACATAATAGCAACAGACATACAAACTAGAGAATTACAAAAACAAATTATAAAAATTCAAAATTTTCGGGTTTATTACAGAGACAGCAGAGACCCTATTTGGAAAGGACCAGCCAAACTACTCTGGAAAGGTGAAGGGGCAGTAGTAATACAAGATAATAGTGACATAAAGGTAGTACCAAGGAGGAAAGCAAAAATCATTAGGGACTATGGAAAACAGATGGCAGGTGCTGATTGTGTGGCAAGTAGACAGGATGAAGAT

>AF443098

TTTTTAGATGGAATAGATAAGGCTCAAGAAGAGCATGAAAAGTATCACAGCAATTGGAGAGCAATGGCCAGTGAGTTTAATCTGCCACCCGTAGTAGCAAAAGAAATAGTGGCTAGCTGTGATGAATGTCAGCTAAAAGGGGAAGCCATGCATGGACAAGTAGACTGTAGTCCAGGAATATGGCAATTAGATTGTACACATTTAGAAGGAAAAATCATCCTGGTAGCAGTCCATGTAGCCAGTGGCTACATAGAAGCAGAGGTTATCCCGGCAGAAACAGGACAAGAAACAGCATACTTTCTACTAAAATTAGCAGGAAGATGGCCAGTCAAAGTAATACATACAGACAATGGTAGTAATTTTACCAGTAATGCAATGAAGGCAGCCTGTTGGTGGGCAGGAATCCAACAGGAATTTGGAATTCCCTACAATCCCCAAAGTCAGGGAGTAGTAGAATCCATGAATAAAGAATTAAAGAAAATCATAGGGCAAGTAAGAGATCAAGCTGAGCACCTTAAGACAGCAGTGCAAATGGCAGTATTCATTCACAATTTTAAAAGAAGAGGGGGGATTGGGGGGTACAGTGCAGGGGAAAGAATAATAGACATAATAGCAACAGACATACAAACTAAAGAATTACAAAAACAAATTATAAAAATTCAAAATTTCCGGGTTTATTACAGGGACAGCAGAGACCCTATTTGGAAAGGACCAGCCAAACTACTCTGGAAAGGTGAAGGGGCAGTAGTAATACAAGATAATAGTGACATAAAGGTAGTACCAAGAAGGAAAGTAAAAATCATTAGGGATTATGGAAAACAGATGGCAGGTGCTGATTGTGTGGCAGGTGGACAGGATGAAAAT

>AF443097

TTTCTAGATGGAATAGATAAGGCTCAAGAAGAGCATGAAAAATATCACAGCAATTGGAGAGCAATGGCTAATGAGTTTAATCTGCCACCCATAGTAGCAAAAGAGATAGTAGCCAGCTGTGATAAATGTCAGCTAAAAGGAGAAGCCATACATGGACAAGTAGACTGTAGTCCAGGGATATGGCAATTAGATTGTACTCATTTAGAAGGAAAAATCGTCCTGGTAGCAGTCCATGTAGCCAGTGGCTACCTGGAAGCAGAGGTCATACCAACAGAAACAGGACAAGAAACAGCATATTACATACTAAAATTAGCAGGAAGATGGCCAGTCAAAGTAATTCATACAGACAATGGCAGTAATTTCACCAGTGCTGCAGTTAAGGCCGCCTGTTGGTGGGCAGGTATCCAACAGGAATTTGGAATTCCCTACAATCCCCAAAGTCAGGGAGTAGTAGAATCCATGAATAAAGAATTAAAGAAGATTATAGGGCAGGTAAGAGATCAAGCTGAGCACCTTAAGACAGCAGTACAAATGGCAGTATTCATTCACAATTTTAAAAGAAAAGGGGGGATTGGGGGGTACAGTGCAGGGGAAAGAATAATAGACATAATAGCAACAGACATACAAACCAGAGAATTACAAAAACAAATTATCAAAATTCAAAATTTTCGGGTTTATTACAGAGACAACAGAGACCCTATTTGGAAAGGACCAGCTAAACTACTCTGGAAAGGTGAAGGGGCAGTAGTACTACAAGATAACAGTGAAATAAAGGTGGTACCAAGGAGGAAAGTAAAAATCATTAGGGACTATGGAAAACAGATGGCAGGTGCTGATTGTGTGGCAGGTAGACAGGATGAAGAT

>AF443096

TTTCTAGATGGAATAGATAAGGCTCAAGAAGATCATGAAAAATATCACAGCAATTGGAGAGCAATGGCTAATGAGTTTAATCTGCCACCCATAGTAGCAAAAGAAATAGTAGCCAGCTGTGATAAATGTCAGCTAAAAGGGGAAGCCATACATGGGCAAGTAGACTGTAGTCCAGGGATATGGCAATTAGATTGTACACATTTAGAAGGAAAAATCATCCTGGTAGCAGTCCATGTAGCCAGTGGCTACATAGAAGCAGAGGTTATCCCAGCAGAAACAGGACAAGAGACAGCATATTTTATACTAAAATTAGCAGGAAGATGGCCAGTCAAAATAATACATACAGACAATGGCAGTAATTTCACCAGTGCTACAGTTAAGGCAGCCTGTTGGTGGGCAGGTATCCAACAGGAATTTGGAATTCCCTACAATCCCCAAAGTCAGGGAGTAGTAGAATCCATGAATAAAGAATTAAAGAAAATTATAGGGCAGGTAAGAGAGCAAGCTGAGCACCTTAAGACAGCAGTACAAATGGCAGTATTCATTCACAATTTTAAAAGAAAAGGGGGGATTGGGGGGTATAGTGCAGGGGAAAGAATAATAGACATAATAGCAACAGACATACAAACTAGAGAATTACAAAAACAAATTATAAAAATTCAAAATTTTCGGGTTTATTACGGAGACAGCAGAGACCCTATTTGGAAAGGACCAGCCAAACTACTCTGGAAAGGTGAAGGGGCGGTAGTAATACAAGATAACAGTGACATAAAAGTAGTACCAAGGAGGAAAGTAAAAATCATTAAGGACTATGGAAAACAGATGGCAGGTGCTGATTGTATGGCAGGTAGACAGGATGAGGAT

>AF443095

TTTCTAGATGGAATAGATAAGGCTCAAGAAGAGCATGAAAAGTATCACAACAATTGGAGAGCAATGGCTAGTGATTTTAATCTGCCACCCGTAGTAGCAAAGGAAATAGTAGCTAGCTGTGATAAATGTCAGCTAAAAGGGGAAGCCATACATGGACAAGTAGACTGTAGTCCAGGGATATGGCAATTAGATTGTACACATTTAGAAGGAAAAGTCATCATAGTAGCAGTCCATGTAGCCAGTGGCTACATAGAAGCAGAGGTTATCCCAGCAGAAACAGGACAAGAAACAGCATACTATATCCTAAAATTAGCAGGAAGATGGCCAGTCAAAGTAATACATACAGACAATGGTAGCAATTTCACCAGTGCTGCAGTTAAGGCAGCCTGTTGGTGGGCAGGTGTCCAACAGGAATTTGGAATTCCCTACAATCCCCAAAGTCAGGGAGTAGTAGAATCCATGAATAAAGAATTAAAGAAAATTATAGGGCAGGTAAGAGATCAAGCTGAGCACCTTAAGACAGCAGTACAAATGGCAGTATTTATTCACAATTTTAAAAGAAAAGGGGGGATTGGGGGGTACAGTGCAGGGGAAAGAATAATAGACATAATAGCAACAGACATACAAACTAAAGAATTACAAAAACAAATTATAAAAATTCAAAATTTTCGGGTTTATTACAGAGACAGCAGAGACCCTATTTGGAAAGGACCAGCCAAACTACTCTGGAAAGGTGAAGGGGCGGTAGTAATACAAGATAATAATGACATAAAGGTAGTACCAAGGAGGAAAGTAAAAATCATTAAGGGCTATGGAAAACAGATGGCAGGTGCTGATTGTGTGGCAGGTGGACAGGATGAAAAT

>AF443094

TTTCTAGATGGAATAGATAAGGCTCAAGGAGGATCTGAAAAGTATCACAGCAATTGGAGAGCAATGGCTAGTGAGTTTAATCTGCCACCCATAGTAGCAAAAGAAATAGTAGCCAGCTGTGATAAATGTCAGCTAAAAGGGGAAGCCATACATGGACAAGTAGACTGTAGTCCAGGGATATGGCAATTAGATTGTACACACTTAGAAGGAAAGATCATCCTGGTAGCAGTCCATGTAGCCAGTGGCTACATAGAAGCAGAGGTTATCCCAACAGAAACAGGACAGGAAACAGCATATCTCATACTGAAATTAGCAGGAAGATGGCCAGTCAAAATAATACATACAGACAATGGCAGTAATTTCACCAGTACTACAGTCAAGGCAGCCTGTTGGTGGGCAGGTATCCAACAGGAATTTGGAATTCCCTACAATCCCCAAAGTCAGGGAGTAGTAGAATCCATGAATAAAGAATTAAAGAAAATCATAGGGCAGGTAAGAGATCAAGCTGAGCACCTTAAGACAGCAGTACAAATGGCAGTATTCATTCACAATTTTAAAAGAAAAGGGGGGATTGGGGGGTACAGTGCAGGGGAAAGAATAATAGACATAATAGCAACAGACATACAAACTAAAGAATTACAAAAACAAATTACAAAAATTCAAAATTTTCGGGTTTATTACAGAGACAGCAGAGACCCTATTTGGAAAGGACCAGCCAAGCTACTCTGGAAAGGTGAAGGAGCAGTAGTAATACAAGATAATAGTGACATAAAGGTAGTACCAAGGAGGAAAGTAAAAATCATTAAGGACTATGGAAAACAGATGGCAGGTGCTGATTGTGTGGCAGGTAGACAGGATGAAGAT

>AF443093

TTCCTGGATGGAATAGATAAGGCTCAGGAAGAGCATGAAAAGTATCACAGCAATTGGAGAGCAATGGCTAGTGAGTTTAATTTGCCACCCGTAGTAGCAAAAGAAATAGTAGCTAGCTGTGATAAGTGTCAGCTAAAAGGGGAAGCCATCCATGGACAAGTAGATTGTAGTCCAGGGATATGGCAATTAGATTGTACACATTTAGAAGGAAAAGTTATCCTGGTAGCAGTCCATGTAGCCAGTGGTTATATAGAAGCAGAGGTTATCCCAGCAGAAACAGGACAAGAAACAGCATACTATATACTAAAATTAGCAGGAAGATGGCCAGTCAAAATAATACATACAGACAATGGCAGTAATTTCACCAGTGCTGCAGTTAAGGCAGCCTGCTGGTGGGCAGGTATCCAACAGGAATTTGGAATTCCCTACAATCCCCAAAGTCAGGGAGTAGTAGAATCCATGAATAAAGAATTAAAGAAAATCATAGGGCAGGTAAGAGATCAAGCTGAGCACCATAAGACAGCAGTACAAATGGCAGTATTTGTTCACAATTTTAAAAGGAAAGGGGGGATTGGGGGGTACAGTGCAGGGGAAAGAATAATAGATATAATAGCAACAGACATACAAACTAAAGAATTACAAAAACAAATTACAAAAATCCAAAATTTTCGGGTTTATTACAGAGACAGCAGAGACCCTATTTGGAAAGGACCAGCCAAACTACTCTGGAAAGGTGAAGGGGCAGTAGTAATACAAGATAATAGTGACATAAAAGTAGTACCAAGGAGGAAAGCAAAAATCATTAGGGACTATGGAAAACAGATGGCAGGTGCTGATTGTGTGGCAGGTAGACAGGATGAAGAT

>AF443092

TTTCTAGATGGAATAGATAAGGCTCAAGAAGAGCATGAAAAATATCACAGCAGTTGGAGAGCAATGGCTAATGAGTTTAATCTGCCACCCATAGTAGCAAAAGAAATAGTAGCTAGCTGTGATAAATGTCAGCTAAAAGGGGAAGCTACACATGGGCAAGTAGACTGTAGTCCAGGAATATGGCAATTAGACTGTACACATCTAGAAGGAAAAATCATCCTGGTAGCAGTCCATGTAGCCAGTGGCTACATAGAAGCAGAGGTTATCCCAGCAGAAACAGGACAAGAAACGGCATACTACATATTAAAATTAGCAGGAAGATGGCCAGTCAAAATAATACATACAGATAATGGTAGTAATTTCACCAGTGCTGCAGTTAAGGCAGCCTGTTGGTGGGCAGGTATCCAACAGGAATTTGGAATTCCCTACAATCCCCAAAGTCAGGGAGTAGTAGAATCCATGAATAAAGAATTAAAGAAAATCATAGGGCAAATAAGAGATCAAGCTGAGCATCTTAAGACAGCAGTACAGATGGCAGTATTCATTCACAATTTTAAAAGAAAAGGGGGGATTGGGGGGTACAGTGCAGGGGAAAGAATAGTAGACATAATAGCAACAGACATACAAACTAGAGAATTACAAAAACAAATTATAAAAATTCAAAATTTTCGGGTTTATTACAGAGACAGCAGAGACCCTATTTGGAAAGGACCAGCCAAACTACTCTGGAAAGGTGAAGGGGCAGTAGTAATACAAGATAACAGTGACATAAAGGTAGTACCAAGGAGGAAAGTAAAAATCATTAGGGACTATGGAAAACAGATGGCAGGTGCTGATTGTGTGGCAGGTAGACAGGATGAGGAT

>AF443091

TTTTTAGATGGAATAGATAAAGCTCAAGAAGAACATGAAAGATATCACAGCAATTGGAGAGCAATGGCTAATGAGTTTAATTTGCCACCCATAGTAGCAAAAGAGATAGTAGCCAGCTGTGATAAATGTCAGTTAAAAGGAGAAGCCATGCATGGACAGGTAGACTGTAGTCCAGGGATATGGCAATTAGATTGCACACACTTAGAAGGAAAAGTTATCCTGGTAGCAGTCCATGTAGCCAGTGGCTACATAGAAGCAGAAGTAATCCCAGCAGAAACAGGACAGGAAGCAGCATACTTCATATTAAAATTAGCAGGAAGATGGCCAGTACAAATAATACATACAGACAATGGTAGCAATTTCACCAGCGCTGCAGTCAAGGCAGCCTGTTGGTGGGCAGGGATCCAGCAGGAATTTGGAATTCCCTACAATCCCCAAAGTCAGGGAGTAGTAAAATCCATGAACAAAGAATTAAAGAAAATAATAGGACAAGTAAGAGATCAAGCTGAACACCTTAAGACAGCAGTACTAATGGCAGTATTCATTCACAATTTTAAAAGAAAAGGGGGGATTGGGGGGTACAGTGCAGGAGAAAGAATAGTAGATATAATAGCATCAGACATACAAACTAAAGAATTACAAAAACAAATTACAAAAATTCAAAATTTTCGGGTTTATTACAGAGACAGCAGAGAACCCGTTTGGAAAGGACCAGCCAAATTGCTCTGGAAAGGTGAAGGGGCAGTAGTAATACAAGACAATAGTGACATAAAGGTGGTACCAAGGAGAAAAGCAAAGATCATTAGGGATTATGGAAAACAGATGGCAGGTGCTGATTGTGTGGCAGGTAGACAGGATGAAGAT

>AF443090

TTTCTAGATGGAATAGATAAGGCTCAAGAAGAGCATGAAAAGTATCACAGCAATTGGAGAGCAATGGCCAGTGAGTTTAATCTACCACCCATAGTAGCAAAGGAAATAGTGGCTAGCTGTGATAAATGTCAGCTAAAAGGGGAAGCCATGCATGGACAAGTAGACTGTAGTCCAGGGATATGGCAATTAGATTGTACACATTTAGAAGGAAAAGTCATCCTGGTAGCAGTCCATGTAGCCAGTGGCTACATAGAGGCAGAGGTTATCCCAGCAGAAACAGGACAAGACACAGCATACTATATACTAAAATTAGCAGGAAGATGGCCAGTCAAAGTAATACATACAGACAATGGCAGTAACTTCACCAGTACAGCAGTTAAGGCAGCCTGTTGGTGGGCAGATATCCAACAGGAATTTGGAATTCCCTACAATCCCCAAAGTCAGGGAGTAGTAGAATCCATGAATAAAGAATTAAAGAAAATAATAGGGCAAGTGAGAGATCAAGCTGAGCACCTGAAGACAGCAGTACAAATGGCAGTATTCATTCACAATTTTAAAAGAAGAGGGGGGATTGGGGGGTACAGTGCAGGGGAAAGAATAATAGACATAATAGCAACAGACATACAAACTAAAGAATTACAAAAACAAATTATAAAAATTCAAAATTTTCGGGTTTATTACAGGGACAGCAGAGACCCTATTTGGAAAGGACCAGCCAAACTGCTCTGGAAAGGTGAAGGGGCAGTAGTAATACAAGATAACGGTGACATAAAGGTAGTTCCAAGGAGGAAAGTAAAAATCATTAAGGACTATGGAAAACAGATGGCAGGTGCTGATTGTGTGGCAGGTAGACAGGATGAAGAT

>AF443089

TTTTTAGATGGAATAGATAAGGCTCAAGAAGAGCATGAAAAGTATCACAGCAATTGGAGAGCAATGGCTAGTGAATTTAATCTGCCACCCATAGTAGCAAAAGAAATAGTAGCTAGCTGTGATAAGTGTCAGCTAAAAGGGGAAGCCATACATGGACAAGTAGACTGTAGTCCAGGGATATGGCAATTAGATTGTACACATTTAGAAGGAAAAATTATCCTGGTAGCAGTCCATGTAGCCAGTGGCTACATGGAAGCAGAGGTTATCCCAGCAGAAACAGGACAGGAAACAGCATACTACATATTAAAATTAGCAGGAAGATGGCCAGTCAAAGTAATACATACAGACAATGGCAGTAATTTCACCAGTGCTGCGGTTAAAGCAGCCTGTTGGTGGGCAGGTATCCAACAGGAATTTGGAATTCCCTACAATCCCCAAAGTCAGGGAGTAGTAGAATCCATGAATAAAGAATTGAAGAAAATCATAGGGCAGATAAGAGATCAAGCTGAGCACCTTAAGACAGCAGTACAGATGGCAGTATTCATTCACAATTTTAAAAGAAAAGGGGGGATTGGGGGGTACAGTGCAGGGGAAAGAATAATAGACATAATAGCAACAGACATACAAACTAAAGAATTACAAAAACAAATTATAAAAATTCAAAATTTTCGGGTTTATTACAGAGACAGCAGAGACCCTATTTGGAAAGGACCAGCCAAACTACTCTGGAAAGGTGAAGGGGCAGTAGTGATACAAGATAATAGTGACATAAAGGTAGTACCAAGGAGGAAAGTAAAAATCATTAAGGACTATGGAAAACAGATGGCAGGGGCTGATTGTGTGGCAGGTAGACAGGATGAAGAT

>AF443088

TTTCTAGATGGAATAGATAAGGCTCAAGACGAGCATGAAAAATATCACAGCAATTGGAGGGCTATGGCCAATGAGTTTAATCTGCCACCCATAGTAGCAAAAGAAATAGTAGCCAGCTGTGATAAGTGTCAGCTAAAAGGGGAAGCCATACATGGACAAGTAGACTGTAGTCCAGGGATATGGCAATTAGATTGTACACATTTAGAAGGAAAGACCATCCTGGTAGCAGTCCATGTAGCCAGTGGCTACATGGAAGCAGAGGTTATCCCAGCAGAAACAGGACAAGAAACAGCATATTACATACTAAAATTAGCAGGAAGATGGCCAGTCAAAATAATACATACAGACAATGGCAGTAATTTCACCAGTGCTGCAGTTAAGGCAGCCTGTTGGTGGGCAGGTATCCAGCAGGAATTTGGAATTCCCTACAATCCCCAAAGTCAGGGAGTAGTAGAATCCATGAACAAAGAATTAAAGAAAATCATAGGACAGGTAAGAGATCAAGCTGAGCACCTTAAGACGGCAGTACAAATGGCAGTATTCATTCACAATTTTAAAAGAAGAGGGGGGATTGGGGGGTACAGTGCAGGGGAAAGAATAATAGACATAATAGCAACAGACATACAAACTAGAGAATTACAAAAACGAATTATACAAATTCAAAATTTTCGGGTTTATTACAGAGACAGCAGAGACCCTATTTGGAAAGGACCAGCCAAACTACTCTGGAAAGGTGAAGGGGCAGTAGTAATACAAGATAATAGTGACATAAAGGTAATACCAAGGAGGAAAGCAAAAATCATTAAGGACTATGGAAAACAGATGGCAGGTGCTGATTGTGTGGCAGGTAGACAGGATGAAGAT

>AF443087

TTTTTAGATGGAATAGATAAAGCTCAAGAAGAGCATGAAAAATATCACAGCAATTGGAGAGCAATGGCTAGTGAGTTTAACCTGCCACCCATAGTAGCAAAAGAAATAGTAGCCAGCTGTGATAAGTGTCAGCTAAAGGGAGAAGCCATACATGGGCAAGTAGACTGTAGTCCAGGAGTATGGCAATTAGATTGTACACATTTAGAAGGAAAAGTCATCCTGGTAGCAGTCCATGTAGCCAGTGGCTACATAGAAGCAGAGGTTATCCCAGCAGAAACAGGACAAGACACAGCATACTATATGCTAAAGTTAGCAGGAAGATGGCCAGTCAAAGTAATACATACAGACAATGGCAGTAATTTCACCAGTGCTGCAGTTAAGGCAGCCTGCTGGTGGGCAGGTGTCCAACAGGAATTTGGAATTCCCTACAATCCCCAAAGTCAGGGAGTAGTAGAATCCACGAATAAAGAGTTAAAGAAAATCATAGGGCAGGTAAGAGAGCAAGCTGAACACCTTAAGACAGCAGTACAAATGGCAGTATTCATTCACAATTTTAAAAGAAGAGGGGGGATTGGGGGGTACAGTGCAGGGGAAAGAATAATAGACATAATAGCAACAGACATACAGACTAAAGAATTACAAAAACAAATTACAAAAATTCAAAATTTTCGGGTTTATTACAGAGACAGCAGAGACCCTATTTGGAAAGGACCAGCCAAACTACTCTGGAAAGGTGAAGGGGCAGTAGTAATACAAGATAATAGTGACATAAAAGTAGTACCAAGGAGGAAAGCAAAAATCATTAAGGACTATGGAAAACAGATGGCAGGGGCTGATTGTGTGGCAGGTAGACAGGATGAGGAT

>AF443086

TTTCTAGATGAAATAGATAAGGCTCAAGACGAGCATGAAAAATATCACAGCAATTGGAGAGCAATGGCTAGTGAGTTTAATCTGCCACCCGTAGTAGCAAAAGAAATAGTAGCCTGCTGTGATAAATGTCAGCAAAAAGGGGAAGCCATACATGGTCAAGTAGACTGTAGTCCAGGGATATGGCAATTAGATTGTACACATTTAGAAGGAAAAATCATCCTGGTAGCAGTCCATGTAGCCAGTGGCTACATAGAAGCAGAGGTTATCCCAGCAGAAACAGGACAAGAAACAGCATATTATATACTAAAATTAGCAGGAAGATGGCCAGTCAAAACAATACATACAGACAATGGCAGTAATTTCACCAGTACTGCAGTTAAGGCAGCCTGTTGGTGGGCAGGTATCCAACAGGAATTTGGAATTCCCTACAATCCCCAAAGTCAGGGAGTAGTAGAATCCATGAATAAAGAATTAAAGAAAATCATAGGGCAAGTAAGAGATCAAGCTGAGCACCTTAAGACAGCAGTACAAATGGCAGTATTCATTCACAATTTTAAAAGAAAAGGGGGGATTGGGGGGTACAGTGCAGGGGAAAGAATAATAGACATAATAGCATCAGACATACAAACTAGAGAATTACAAAAACAAATAATAAAAATTCAAAATTTTCGGGTTTATTACAGAGACAGCAGAGACCCTATTTGGAAAGGACCAGCCAAACTACTCTGGAAAGGTGAAGGGGCAGTAGTGATACAAGATAAGAGTGACATAAAAGTAGTACCAAGGAGGAAAGCAAAAATCATTAAGGATTATGGAAAACAGATGGCAGGTGATGATTGTGTGGCAGGTAGACAGGATGAAGAT

>AF443085

TTTCTAGATGGGATAGATAAAGCCCAAGAAGAGCATGAAAAATATCACAGCAATTGGAGAGCAATGGCTAGTGAGTTTAATCTGCCACCCGTAGTAGCAAAAGAAATAGTAGCCAGCTGTGATAAATGTCAGCTAAAAGGGGAAGCCATACATGGACAAGTAGACTGTAGTCCAGGAATATGGCAATTAGATTGTACACATCTAGAAGGAAAAGTCATACTGGTAGCAGTCCACGTAGCCAGTGGCTACATAGAGGCAGAAGTTATCCCAGCAGAAACAGGACAAGAAACAGCATACTATATATTAAAGTTAGCAGGACGATGGCCTGTCAAAGTAATACATACAGACAATGGTAGTAATTTCACCAGTACTGCAGTAAAGGCAGCCTGTTGGTGGGCAGGTATCCAACAGGAATTTGGAATTCCCTACAATCCCCAAAGTCAAGGAGTAGTAGAACCCATGAATAAAGAATTAAAGAAAATCATAGGGCAGGTAAGAGATCAAGCTGAGCACCTTAAGACAGCAGTACAAATGGCAGTATTCATTCACAATTTTAAAAGAAAAGGGGGGATTGGGGGGTACAGTGCAGGGGAAAGAATAATAGACATAATAGCAACAGACATACAAACTAAAGAATTACAAAAACAAATTATAAAAATTCAAAATTTTCGGGTTTATTACAGAGACAGCAGAGACCCTATTTGGAAAGGACCAGCCAAACTACTCTGGAAAGGTGAAGGGGCAGTAGTAATACAAGATAACAGTGACATAAAGGTAGTACCAAGGAGGAAAGTAAAAATCATTAAGGACTATGGAAAACAGATGGCAGGTGCTGATTGTGTGGCAGGTAGACAGGATGAGGAT

>AF443084

TTTCTAGATGGAATAGATAAGGCTCAAGATGAGCATGAAAAATATCACAGCAATTGGAGAGCAATGGCTAGTGAGTTTAATTTGCCACCCATAGTAGCAAAAGAGATAGTAGCTAGCTGTGATAAATGTCAGCTAAAAGGGGAAGCCATACATGGACAAGTAGACTGTAGTCCAGGGATATGGCAATTAGATTGTACACATTTAGAAGGAAAAATCATCCTGGTAGCAGTCCATGTAGCCAGTGGCTACATAGAAGCAGAGGTCATCCCAGCAGAAACAGGACAAGAAACAGCATACTACATACTAAAATTAGCAGGAAGATGGCCAGTCAAAGTAATACATACAGACAATGGCAGTAATTTCACCAGTGCTGCAGTTAAGGCAGCCTGCTGGTGGGCAGGTATCCAACAGGAGTTTGGAATTCCCTACAATCCCCAAAGTCAGGGAGTAGTAGAATCCATGAATAAAGAATTAAAGAAAATCATAGGACAGGTAAGAGACCAAGCTGAGCACCTTAAGACAGCGGTACAAATGGCAGTATTCATTCACAATTTTAAAAGAAAAGGGGGGATTGGGGGGTACAGTGCAGGGGAAAGAATAATAGACATAATAGCAACAGACATACAAACTAGAGAATTACAAAAGCAAATTATAAAAATTCAAAATTTTCGGGTTTATTACAGAGACAGCAGAGACCCTATTTGGAAAGGACCAGCCAAACTACTCTGGAAAGGTGAAGGGGCAGTAGTAATCCAAGATAATAGTGACATAAAGGTAGTACCAAGGAGGAAAGCAAAAATCATTAAGGACTATGGAAAACAGATGGCAGGTGCTGATTGTGTGGCAGATAGACAGGATGAAGAT

>AF443083

TTTCTAGATGGAATAGATAAGGCTCGGGAAGAGCATGAAAAATATCACAGCAATTGGAGAGCAATGGCCAGTGAGTTTAATCTGCCACCCATAGTAGCAAAAGAAATAGTAGCTAGCTGTGATAAATGTCAGTTAAAAGGGGAAGCTACACATGGACAAGTAGACTGTAGTCCAGGAATATGGCAATTAGATTGTACACATCTAGAAGGAAAAGTCATCCTGGTAGCAGTCCATGTAGCCAGTGGCTACATAGAAGCAGAGGTTATCCCAGCAGAAACAGGACAAGAAACAGCATACTTTATACTAAAGTTAGCAGGAAGATGGCCAGTCAAAGTGATACATACAGACAATGGCAGTAATTTTACCAGTGCTGCAGTTAAGGCAGCCTGTTGGTGGGCAGGTATCCAACAGGAATTTGGAATTCCCTACAATCCCCAAAGTCAGGGAGTAGTAGAATCCATGAATAAAGAATTAAAGAAAATCATAGGACAGGTAAGAGATCAAGCTGAGCACCTTAAGACAGCAGTACAAATGGCAGTATTCATTCACAATTTTAAAAGAAAAGGGGGGATTGGGGGGTACAGTGCAGGGGAAAGAATAATAGATATAATAGCAACAGACATACAAACTAGAGAATTACAAAAACAAATTATACAAATTCAAAATTTTCGGGTTTATTACAGAGACAGCAGAGACCCTATTTGGAAAGGACCAGCCAAACTACTCTGGAAAGGGGAAGGAGCAGTAGTAATACAAGATAACAGTGACATAAAGGTAGTACCAAGGAGAAAAGCAAAAATCATTAAGGACTATGGAAAACAGATGGCAGGTGCTGATTGTGTGGCAAGTAGACAGGATGAGGAT

>AF443082

TTTTTAGATGGAATAGATAAGGCTCAAGAAGATCATGAAAAGTATCACAGCAATTGGAGAGCAATGGCTAATGAATTTAATCTGCCACCCATAGTAGCAAAAGAAATAGTAGCTAGCTGTGATAAATGTCAGTTAAAAGGAGAAGCCATACATGGGCAAGTAGACTGTAGTCCAGGGATATGGCAATTAGATTGTACACATTTAGAAGGAAAAATCATCATAGTAGCAGTCCATGTAGCCAGTGGCTATGTGGAAGCAGAAGTTATCCCAGCAGAAACAGGACAGGAAACAGCATACTATATACTAAAACTAGCAGGAAGATGGCCAGTCAAAGTAATACATACAGACAATGGCAGTAATTTCACCAGTACTGCAGTCAAGGCAGCCTGTTGGTGGGCAGGTATCCAACAGGAATTTGGAATTCCCTACAATCCCCAAAGTCAGGGGGTAGTAGAATCCATGAATAAAGAATTAAAGAAAATCATAGGGCAGGTAAGAGATCGAGCTGAGCACCTTAAGACAGCAGTACAAATGGCAGTATTCATTCACAATTTTAAAAGAAAAGGGGGGATTGGGGGGTACAGTGCAGGGGAAAGAATAATAGACATAATAGCAACAGACATACAAACTAAAGAACTACAAAAACAAATTACAAAAATTCAAAATTTTCGGGTTTATTACAGAGACAGCGGAGACCCTATTTGGAAAGGACCAGCCAAACTACTCTGGAAAGGTGAAGGAGCAGTAGTGATACAAGATAATAGTGACATAAAGGTAGTACCAAGAAGGAAAGCAAAAATTATTAGAGACTATGGAAAACAGATGGCAGGTGCTGATTGTGTGGCAGGTAGACAGGATGAAGAT

>AF443081

TTTCTAGATGGAATAGATAAGGCTCAAGAGGAGCATGAAAAATATCACAACAATTGGAGAGCAATGGCTAGTGAGTTTAATCTGCCACCCATAGTAGCAAAAGAAATAGTAGCTAGCTGTGATAAATGTCAGCTAAAAGGGGAAGCCATGCATGGACAAGTAGACTGTAGCCCAGGGATATGGCAATTAGACTGTACACATTTAGAAGGAAAAATCATCCTGGTAGCAGTCCATGTAGCCAGTGGTTACATAGAAGCAGAGGTTATCCCAGCAGAAACAGGACAAGAAACAGCATACTATATACTAAAATTAGCAGGAAGATGGCCAGTCAAAGTAATACATACAGACAATGGCAGTAATTTCACCAGTGCTGCAGTTAAGGCAGCCTGTTGGTGGGCAGGTATCCAACAGGAATTTGGAATTCCCTACAATCCCCAAAGTCAGGGAGTAGTAGAATCCATGAATAAAGAATTAAAGAAAATTATAGGGCAAGTAAGAGACCAAGCTGAACACCCTAAGACAGCAGTACAAATGGCAGTATTCATTCACAATTTTAAAAGAAAAGGGGGGATTGGGGGGTACAGTGCAGGGGAAAGAATAATAGACATAATAGCAACAGACATACAAACTAAAGAATTACAAAGACAAATTTTAAAAATTCAAAATTTTCGGGTTTATTACAGAGACAGCAGAGACCCTATTTGGAAAGGACCAGCCAAACTACTCTGGAAAGGTGAAGGGGCAGTAGTAATACAAGATAATAGTGACATAAAAGTAGTACCAAGGAGGAAAGCAAAAATCATTAAGGACTATGGAAAACAGATGGCAGGTGCTGATTGTGTGGCAGGTGGACAGGATGAGGAT

>AF443080

TTTCTAGATGGAATAGATAAGGCTCAAGAAGAGCATGAAAAATATCACAGCAATTGGAGAGCAATGGCTAGTGAGTTTAATCTACCACCCATAGTAGCAAAAGAGATAGTAGCTAGCTGTGATAAATGTCAGCTAAAAGGGGAAGCCATGCATGGACAGGTAGACTGTAGTCCAGGGATATGGCAATTAGATTGTACACATTTAGAAGGAAAAGTCATCCTGGTAGCAGTCCATGTAGCCAGTGGCTACATAGAAGCAGAGGTTATCCCAGCAGAAACAGGACAAGAAACAGCATATTATATACTAAAATTAGCAGGAAGATGGTCAGTCAAAACAATTCATACAGATAATGGCAGTAATTTCACCAGTGCTGCAGTTAAGGCAGCCTGTTGGTGGGCAGGTATCCAACAGGAATTTGGAATTCCCTACAATCCCCAAAGTCAGGGAGTAGTAGAATCCATGAATAAGGAATTAAAGAAAATCATAGGGCAAGTTAGAGACCAAGCTGAGCACCATAAGACAGCAGTACAAATGGCAGTATTCATTCACAATTTTAAAAGAAAAGGGGGGATTGGGGGGTACAGTGCAGGGGAGAGAATAATAGACATAATAGCAACAGACATACAAACTAGAGAATTACAAAGACAAATTATAAAAATTCAAATTTTTCGGGTTTATTACAGAGACAGCAGAGACCCTATTTGGAAAGGACCAGCCAAACTACTCTGGAAAGGTGAAGGGGCGGTAGTAATACAAGACAATAGTGACATAAAGGTAGTGCCAAGGAGGAAAGTAAAAATCATCAAGGATTATGGAAAACAGATGGCAGGTGCTGATTGTGTGGCAGGTAGACAGGATGAGGAT

>AF443079

TTTCTAGATGGAATAGATAAGGCTCAGGAAGAACATGAAAAATATCATAGCAATTGGAGAGCAAGGGCTAGTGAGTTCAATCTGCCACCCATAGTAGCAAAAGAAATAGTAGCTAGCTGTGATAAATGTCAGCTAAAAGGAGAAGCCATGCATGGACAAGTAGACTGTAGTCCAGGGATATGGCAATTGGACTGTACACATTTAGAAGGAAAAATCATCCTGGTAGCAGTCCATGTAGCCAGTGGCTACATAGAAGCAGAAGTTATCCCAGCAGAAACAGGACAAGAGACAGCATACTTTATACTAAAATTAGCAGGAAGATGGCCTGTCAAAGTAATACATACAGACAATGGCAGTAATTTCACCAGTGCTGCAGTTAAGGCAGCCTGTTGGTGGGCAGGTATCCAACAGGAATTTGGAATTCCCTACAATCCCCAAAGTCAGGGAGTAGTAGAATCCATGAATAAAGAATTAAAGAAAATCATAGGGCAGGTAAGAGACCAAGCTGAGCACCTTAAGACAGCAGTACAAATGGCAGTATTCATTCACAATTTTAAAAGAAAAGGGGGGATTGGGGGGTACAGTGCAGGGGAGAGAATAATAGATATAATAGCAACAGACATACAAACTAAAGAATTACAAAAACAAATTTTAAACATTCAAAAATTTCGGGTTTATTACAGAGACAGCAGAGACCCTATTTGGAAAGGACCAGCCAAACTACTCTGGAAAGGTGAAGGAGCAGTAGTAATACAAGATAACAGTGACATAAAGGTAGTACCAAGAAGGAAAGCAAAAATCATTAAGGACTATGGAAAACAGATGGCAGGTGCTGATTGTGTGGCAGGTAGACAGGATGAAGAT

>AF443078

TTTCTAGATGGAATAGATAAGGCTCAAGAAGAACATGAGAAGTATCATAGCAATTGGAGAGCAATGGCTAGTGAGTTTAATCTGCCACCCATAGTAGCAAAAGAAATAGTAGCTAGCTGTGATAAGTGTCAGCTAAAAGGGGAAGCCATACATGGACAAGTAGATTGTAGCCCAGGGATATGGCAATTAGATTGTACACATTTAGAAGGAAAAGTCATCCTGGTAGCAGTCCATGTAGCCAGTGGCTATATAGAAGCAGAGGTTATTTCAGCAGAAACTGGACAGGAAACAGCATACTATATACTAAAATTAGCAGCAAGATGGCCAGTCAAAGTAATACATACAGACAATGGCAGTAATTTCACCAGTGCTGCAGTTAAGGCAGCCTGTTGGTGGGCAGGTATCCAACAGGAATTTGGAATTCCCTACAATCCCCAAAGTCAGGGAGCAGTAGAATCCATGAATAAAGAATTAAAGAAAATCATAGGGCAGGTAAGAGATCAAGCTGAGCACCTTAAGACAGCAGTACAAATGGCAGTATTCATTCACAATTTTAAAAGAAAAGGGGGGATTGGGGGGTACAGTGCAGGGGAAAGAATAATAGATATAATAGCAACAGACATACAAACTCAAGAATTACAAAAACAAATTATCAAAATTCAAAATTTTCGGGTTTATTACAGAGACAGCAGAGACCCTATTTGGAAAGGACCAGCCAAACTACTCTGGAGAGGTGAAGGGGCAGTAGTAATACAAGATAGTAGTGACATAAAGGTAGTACCAAGGAGGAAAGCAAAGATCATTAAGGACTATGGAAAACAGATGGCAGGGGCTGATTGTGTGGCAGGTAGACAGGATGAAGAT

>AF443077

TTTCTAGATGGAATAGATAAGGCTCAGGACGACCATGAAAGATATCACAGCAATTGGAGAGCAATGGCTAATGAGTTTAACCTGCCACCCATAGTAGCAAAAGAAATAGTAGCCAGCTGTGATAAATGTCAGCTAAAAGGGGAAGCCATACATGGGCAAGTAGACTGTAGTCCAGGGATATGGCAATTAGATTGTACACATTTAGAAGGAAAAATCATCCTGGTAGCAGTCCATGTAGCCAGTGGCTACATAGAAGCAGAAGTTATACCAGCAGAAACAGGACAAGAAACAGCATATTATATACTAAAATTGGCAGGAAGATGGCCAGTCAAAATAATACATACAGACAATGGCAGTAATTTCACCAGTGCTGCAGTTAAAGCAGCCTGTTGGTGGGCAGGTATCCAACAGGAATTTGGAATTCCCTACAATCCCCAAAGTCAGGGAGTAGTAGAATCCATGAATAAAGAATTAAAGAAAATCATAGGGCAGGTAAGAGATCAAGCTGAGCACCTTAAGACAGCAGTACAAATGGCAGTATTCATTCATAATTTTAAAAGAAAAGGGGGGATTGGGGGGTACAGTGCAGGGGAAAGAATAATAGACATAATAGCAACAGACATACAAACCAGAGAATTACAAAAACAAATTATAAAAATTCAAAATTTTCGGGTTTATTACAGAGACAACAGAGACCCTATTTGGAAAGGACCAGCTAAACTACTCTGGAAAGGTGAAGGAGCAGTAGTAATACAAGATAACAGTGAAATAAAGGTAGTACCAAGGAGGAAAGTAAAAATCATTAGGGACTATGGAAAACAGATGGCAGGTGCTGATTGTGTGGCAGGTAGACAGGATGAAGAT

>AF443076

TTTCTAGATGGAATAGATAAGGCTCAAGACGAGCATGAAAAGTATCACAGCAATTGGAGAGCAATGGCCAGTGAGTTTAATCTGCCACCCATAGTAGCAAAAGAAATAGTGGCTAGCTGTGATAAATGTCAGCTAAAAGGGGAAGCCATACATGGACAAGTAGACTGTAGTCCAGGGATATGGCAATTAGATTGTACACATCTAGAAGGAAAAATCATCCTGGTAGCAGTCCATGTAGCCAGTGGTTACATGGAGGCAGAGGTTATCACAGCAGAAACAGGACAAGAAACAGCATACTTTATACTAAAATTAGCAGGAAGATGGCCTGTCAAAGTAATACACACAGACAATGGCAGTAACTTCACCAGTGGTGCAGTTAAGGCAGCCTGTTGGTGGGCAGGTATCCAACAGGAATTTGGAATTCCCTACAATCCCCAAAGTCAGGGAGTAGTAGAATCCATGAATAAAGAATTAAAGAAAATAATAGGGCAAATAAGAGATCAAGCAGAGCACCTTAAGACAGCAGTACAAATGGCAGTATTCATTCACAATTTTAAAAGAAAAGGGGGGATTGGGGGGTACAGTGCAGGGGAAAGAATAATAGACATAATAGCAACAGACATACAAACTAAAGAATTACAAAAACAAATTACAAAAATTCAAAATTTTCGGGTTTATTACAGGGATAGCAGAGACCCTGTTTGGAAAGGACCAGCCAAGCTACTCTGGAAAGGTGACGGGGCAGTAGTCATACAAGATAACAGTGACATAAAGGTAGTACCAAGGAGGAAAGCAAAAATCATTAGGGACTATGGAAAACAGATGGCAGGTGCTGATTGTGTGGCAGGTAGACAGGATGAGGAT

>AF443075

TTTTTAGATGGAATAGATAAGGCTCAAGAAGAGCATGAAAAGTATCACAACAATTGGAGAGCAATGGCTAGTGAGTTTAATTTGCCACCCATAGTAGCAAAAGAAATAGTAGCTAGCTGTGATAAATGTCAGCTAAAAGGGGAAGCCATGCATGGACAAGTAGACTGTAGTCCAGGAATATGGCAATTAGATTGTACCCATTTAGAAGGAAAAGTCATTCTGGTAGCAGTACATGTAGCCAGTGGCTACATGGAAGCAGAGGTTATCCCAGCAGAAACAGGACAAGATACAGCATACTTTCTACTAAAATTAGCAGGAAGGTGGCCAGTCAAAGTAATACATACAGACAATGGCAGTAATTTCACCAGTGCTGCAATGAAGGCAGCCTGTTGGTGGGCAGGTATCCAACAGGAATTTGGAATTCCCTATAATCCCCAAAGTCAGGGAGTAGTAGAATCCATGAATAAAGAATTAAAGAAAATCATAGGGCAGGTAAGAGATCAAGCTGAGCACCTTAAGACAGCAGTACAAATGGCAGTATTCATTCACAATTTTAAAAGAAAAGGGGGGATTGGGGGGTACAGTGCAGGGGAAAGAATAATAGATATAATAGCAACAGATATACAAACTAAAGAATTACAGAAACAAATTACAAAAATTCAAAATTTTCGGGTTTATTACAGAGACAGCAGAGACCCTATTTGGAAAGGACCAGCCAAACTACTCTGGAAAGGTGAAGGGGCAGTAGTAATACAAGATAACAGTGACATAAAGGTGGTACCCAGGAGGAAAGCAAAAATCATTAGGGACTATGGAAAACAGATGGCAGGTGCTGATTGTGTGGCAGGTAGACAGGATGAGGAT

>AF443074

TTTCTAGATGGAATAGATAAGGCTCAAGAAGAGCATGAAAGATACCACAGCAATTGGAGAGCAATGGCTAGTGAGTTTAACCTGCCACCCATAGTAGCAAAAGAAATAGTAGCCAGCTGTGATAAATGTCAGCTAAAAGGGGAAGCCATACATGGACAAGTAGATTGTAGCCCAGGGATATGGCAATTAGATTGTACACATTTAGAAGGAAAAATCATCCTGGTAGCAGTCCATGTAGCCAGTGGTTACATGGAAGCAGAGGTTATACCAGCGGAAACAGGACAAGAAACAGCATATTTTCTACTAAAACTAGCAGGAAGATGGCCAGTCAAAATAATACATACAGACAATGGCAGTAATTTCACCAGTGCTGCAGTTAAGGCGGCCTGTTGGTGGGCAGGTATCCAACAGGAATTTGGAATTCCCTACAATCCCCAAAGTCAGGGAGTAGTAGAATCCATGAATAAAGAATTAAAGAAAATCATAGGGCAGGTAAGAGATCAAGCTGAGCACCTTAAGACAGCAGTACAAATGGCAGTATTCATTCACAATTTTAAAAGAAAAGGGGGGATTGGGGGGTACAGTGCAGGGGAAAGAATAATAGACATAATAGCAACAGACATACAAACTAGAGAATTACAAAAACAAATTATAAAAATTCAAAATTTTCGGGTTTATTACAGAGACAACAGAGACCCTATTTGGAAAGGACCAGCTAAACTACTCTGGAAAGGTGAAGGGGCAGTAGTAATACAAGATAACAGTGAAATAAAGGTAGTACCAAGGAGGAAAGCAAAAATCATTAGGGATTATGGAAAACAGATGGCAGGGGCTGATTGTGTGGCAGGTAGACAGGATGAAGAT

>AF411967

TTTCTAGATGGGATAGATAAGGCTCAAGAAGAGCATGAAAAATATCACAGCAATTGGAGAGCAATGGCTAGTGAGTTTAATCTGCCACCCATAGTAGCAAAAGAAATAGTAGCTAGCTGTGATAAATGTCAACTAAAAGGGGAAGCCATGCATGGACAAGTAGACTGTAGTCCAGGGATATGGCAATTAGACTGTACACATTTAGAAGGAAAAATCATCCTGGTAGCAGTCCATGTAGCCAGTGGCTACGTAGAAGCAGAGGTTATCCCAGCAGAAACAGGACAAGAAACAGCATACTATATACTAAAGTTAGCAGGAAGATGGCCAGTCAAAGTAATACATACAGACAATGGCAGCAATTTCACCAGTACTGCAGTTAAGGCAGCCTGTTGGTGGGCAGGTATCCAACAGGAATTTGGAATTCCCTACAATCCTCAAAGCCAGGGAGTAGTAGAATCCATGAATAAAGAATTAAAGAAAATCATAGGGCAAGTAAGAGATCAAGCTGAGCACCTTAAGACAGCAGTACAAATGGCAGTATTCATTCACAATTTTAAAAGAAAAGGGGGGATTGGGGGATACAGTGCAGGGGAGAGAATAATAGACATAATAGCAACAGACATACAAACTAAAGAATTACAAAAACAAATTATAAAAATTCAAAATTTTCGGGTTTATTACAGAGACAGCAGAGACCCTATTTGGAAAGGACCAGCCAAACTACTCTGGAAAGGTGAAGGGGCAGTAGTAATACAAGATAATAGTGACATAAAGGTAGTACCAAGGAGGAAAGTAAAAATTATTAGGGACTATGGAAAACAGATGGCAGGTGATGATTGTGTGGCAGGTAGACAGGATGAGGAT

>AF411966

TTTCTAGATGGAATAGAGAAGGCTCAAGATGAGCATGAAAGGTATCACAGCAATTGGAGGGCAATGGCTAGTGATTTTAACCTGCCACCTATAGTAGCAAAAGAAATAGTAGCTAGCTGTGATAAATGTCAGCTAAAAGGAGAAGCCATACATGGACAAGTGGACTGTAGTCCGGGGATATGGCAATTAGATTGTACACATTTAGAAGGAAAAATCATCCTGGTAGCAGTCCATGTAGCCAGTGGCTACATAGAAGCAGAGGTTATCCCAGCAGAAACAGGACAAGAAACAGCATACTTTATATTAAAATTAGCAGGAAGATGGCCAGTCAAGGTAATACATACAGACAATGGTCCTAATTTCACCAGTGCTGCAGTTAAGGCAGCCTGTTGGTGGGCAGGTATCCAACAGGAATTTGGAATTCCCTACAATCCCCAAAGTCAGGGAGTAGTAGAATCCATGAATAAAGAATTAAAGAAAATTATAGGGCAGGTAAGAGATCAAGCTGAGCACCTTAAGACAGCAGTACAAATGGCAGTATTCATTCACAATTTTAAAAGAAAAGGGGGGATTGGGGGATACAGTGCAGGGGAAAGAATAATAGACATAATAGCAACAGACATACAAACTAAAGAATTACAAAAACAAATTATAAAAATTCAAAATTTTCGGGTTTATTACAGAGACAGCAGAGACCCTACTTGGAAAGGACCAGCCAAACTACTCTGGAAAGGTGAAGGGGCAGTAGTAATACAAGATAACAGTGACATAAAGGTAGTACCAAGGAGGAAAGTAAAAATCATTAAGGACTATGGAAAACAGATGGCAGGTGCTGATTGTGTGGCAGGTAGACAGGATGAAGAT

>AF411965

TTTCTAGATGGGATAGATAAGGCTCAAGAAGATCATGAAAGATATCACAGCAATTGGAGAGCAATGGCTAGTGAGTTTAATCTGCCACCCATAGTAGCAAAAGAAATAGTAGCTAGCTGTGATAAATGTCAGCTAAAAGGGGAAGCCATACATGGACAAGTAGACTGTGGTCCGGGGATATGGCAATTAGACTGTACACATTTAGAAGGAAAAATCATCCTGGTAGCAGTCCATGTAGCCAGTGGCTACATAGAAGCAGAGGTTATCCCAGCAGAAACAGGACAAGAAACAGCATACTTTATACTAAAATTAGCAGGAAGATGGCCAGTCAAAGTAATACATACAGACAATGGCAGTAATTTCACCAGTGCTGCAGTTAAGGCAGCCTGTTGGTGGGCAGGTATCCAACAGGAATTTGGGATTCCCTACAATCCCCAAAGTCAGGGAGTGGTAGAATCCATGAATAAAGAATTAAAGAAAATCATAGGGCAGGTAAGAGATCAAGCTGAGCACCTTAAGACAGCAGTACAAATGGCAGTATTCATTCACAATTTTAAAAGAAAAGGGGGGATTGGGGGGTACAGTGCAGGGGAAAGAATAATAGACATAATAGCAACAGACATACAAACTAAAGAATTACAAAAACAAATTATAAAAATTCAAAATTTTCGGGTTTATTACAGAGACAGCAGAGACCCTATTTGGAAAGGACCAGCCAAACTACTCTGGAAAGGTGAAGGAGCAGTAGTAATACAAGATAACAGTGACATAAAGGTAGTACCAAGGAGGAAAGCAAAAATCATTAAGGACTATGGAAAACAGATGGCAGGTGCTGATTGTGTGGCAGGTAGACAGGATGAAGAT

>AF411964

TTTTTAGATGGCATAGATAAAGCCCAAGAAGATCATGAAAAATATCACTGCAATTGGAGAGCAATGGCTAGTGACTTTAATCTGCCACCTATAGTAGCAAAAGAAATAGTGGCCAGCTGTGATAAATGTCAGCTAAAAGGGGAAGCCATGCATGGACAAGTAGACTGTAGTCCAGGAATATGGCAACTAGATTGTACACATTTAGAAGGAAAAATTATCCTGGTAGCAGTCCATGTAGCCAGTGGCTATATAGAAGCAGAAGTTATCCCAGCAGAAACAGGACAAGAAACAGCATACTTTATATTGAAATTAGCAGGAAGATGGCCAGTAAAAGTAGTACATACAGATAATGGCAGCAATTTCACCAGTGCTGCAGTAAAGGCAGCATGTTGGTGGGCGAATATCAAACAAGAATTTGGAATTCCCTACAATCCCCAAAGTCAAGGAGTAGTAGAATCTATGAATAAAGAATTAAAGAAAATTATAGGGCAGGTCAGAGATCAAGCTGAACACCTTAAGACAGCAGTACAGATGGCAGTATTCATTCACAATTTTAAAAGAAAAGGGGGGATTGGGGGGTACAGTGCAGGGGAAAGAATAGTAGACATAATAGCATCAGATATACAAACTAAAGAACTACAAAAACAAATTACAAAAATTCAAAAATTTCGGGTTTATTACAGGGACAGCAGAGACCCGATTTGGAAAGGACCAGCAAAACTACTCTGGAAAGGTGAAGGGGCGGTAGTAATACAGGACAATAGTGATATAAAGGTAGTACCAAGAAGAAAAGCAAAGATTATTAGGGATTATGGAAAACAGATGGCAGGTGATGATTGTGTGGCAGGTAGACAGGATGAGGAT

>AF484522

TTCTTAGATGGAATAGATAAGGCTCAAGAAGAACATGAGAAATACCACAACAATTGGAGAGCTATGGCTAGTGATTTTAATCTGCCACCTGTGGTAGCAAAAGAAATAGTAGCTAGCTGTGATAAATGTCAGCTAAAAGGAGAAGCCATGCATGGACAAGTAGACTGTAGTCCAGGAATATGGCAATTAGATTGTACACATTTAGAAGGAAAAGTTATCCTGGTAGCAGTCCATGTAGCCAGTGGCTATATGGAAGCAGAAGTTATTCCAGCAGAAACAGGGCAGGAAACAGCCTACTTTATCTTAAAATTAGCAGGAAGATGGCCAGTAAAAGTAGTACATACAGACAATGGCAGCAATTTCACCAGTGCTGCAGTTAAGGCCGCCTGTTGGTGGGCTGGCATCAAGCAGGAATTTGGAATTCCCTACAATCCCCAAAGTCAAGGAGTAGTAGAATCTATGAATAAAGAATTAAAGAAAATTATAGGACAGGTAAGAGATCAAGCTGAACATCTTAAGACAGCAGTACAAATGGCAGTATTCATTCACAATTTTAAAAGAAAAGGGGGGATTGGGGGGTACAGTGCAGGGGAAAGAATAATAGACATGATAGCAACAGACATACAAACTAAAGAACTACAAAAACAAATTACAAAAATTCAAAATTTTCGGGTTTATTACAGGGACAGCAGAGATCCAATTTGGAAAGGACCAGCAAAGCTTCTCTGGAAAGGTGAAGGGGCAGTAGTAATACAGGACAACAGTGATATAAAGGTAGTACCAAGAAGAAAAGCAAAGATCATTAGGCATTATGGAAAACAGATGGCAGGTGATGATTGTATGGCAGGTAGACAGGATGAGGAT

>AF484521

TTTTTAGATGGAATAGATAAAGCTCAAGAAGAACATGAGAAATACCATAACAATTGGAGAGCAATGGCTAGTGATTTTAATCTGCCACCTGTGGTAGCAAAAGAAATAGTAGCTAGCTGTGATAAATGTCAGCTGAAAGGAGAAGCCTTGCATGGACAAGTAGACTGTAGTCCAGGAATATGGCAATTAGATTGTACACATTTAGAAGGAAAAGTTATCCTGGTAGCAGTCCATGTAGCCAGTGGCTATATAGAAGCAGAAGTCATTCCAGCAGAAACAGGGCAGGAAACAGCCTACTTTCTCTTAAAATTAGCAGGAAGATGGCCAGTAAAAGTAGTACACACAGACAATGGCAGCAATTTCATCAGCGCTGCAGTTAAGGCCGCCTGTTGGTGGGCAGGCATCAAACAGGAATTTGGAATTCCCTACAATCCCCAAAGTCAAGGAGTAGTAGAATCTATGAATAAGGAATTAAAGAAAATTATAGGACAGGTAAGAGATCAAGCTGAACATCTTAAGACAGCAGTACAAATGGCAGTATTTATCCACAATTTTAAAAGAAAAGGGGGGATTGGGGGGTACAGTGCAGGGGAAAGAATAATAGACATAATAGCAACAGACATACAAACCAAAGAATTACAAAAACAAATCACAAAAATTCAAAATTTTCGGGTTTATTACAGGGACAGCAGAGATCCAGTTTGGAAAGGACCAGCAAAGCTCCTCTGGAAAGGTGAAGGGGCAGTAGTAATACAAGACAATAGTGAAATAAAAGTAGTACCAAGAAGAAAAGCAAAGATCATTAGGGATTATGGAAAACAGATGGCAGGTGATGATTGTGTGGCAAGTAGACAGGATGAGGAT

>AF484520

TTCTTGGATGGAATAGATAAGGCTCAAGAGGATCATGAGAAATACCACAGCAATTGGAGAGCAATGGCTAGTGATTTCAACCTGCCACCTGTGGTAGCAAAAGAAATAGTAGCTAGCTGTGATAAATGTCAGCTAAAAGGAGAAGCCTTGCATGGGCAAGTAGACTGTAGTCCAGGAATATGGCAGTTAGATTGTACACATTTAGAAGGAAAAGTTATCCTGGTAGCAGTCCATGTAGCCAGCGGCTATATAGAAGCAGAAGTTATTCCAGCAGAAACAGGGCAGGAMACAGCCTACTTCATCTTGAAATTAGCAGGAAGATGGCCAGTAAAAGTAGTACATACAGACAATGGCAGCAATTTCACCAGCACTGCAGTTAAGGCCGCCTGTTGGTGGGCAGGCATCAAGCAGGAATTTGGAATTCCCTACAATCCCCAAAGTCAAGGAGTAGTAGAATCYATGAATAAAGAACTAAAGAAAATTATAGGACAGGTAAGAGATCAAGCTGAACATCTTAAGACAGCAGTACAAATGGCAGTATTCATCCACAATTTTAAAAGAAAAGGGGGGATTGGGGAGTACAGTGCAGGGGAAAGAATAATAGATATAATAGCAACAGACATACAAACTAAAGAACTACAAAAGCAAATCATAAAAATTCAAAATTTTCGGGTTTATTACAGGGACAGCAGAGATCCAGTTTGGAAAGGACCAGCAAAGCTTCTCTGGAAAGGTGAAGGGGCAGTAGTAATACAAGACAATAGTGAAATAAAGGTAGTACCAAGAAGAAAAGTAAAGATCATTAGGGATTATGGAAAACAGATGGCAGGTGATGATTGTGTGGCAGGTAGACAGGATGAGAAT

>AF484519

TTCTTGGATGGAATAGATAAGGCTCAAGAAGAACATGAAAAATACCACAACAATTGGAGAGCAATGGCTAGTGATTTTAACCTGCCACCTGTGGTAGCAAAAGAAATAGTAGCTAGCTGTGATAAATGCCAGGTAAAAGGAGAAGCCTTGCATGGACAAGTAGACTGTAGTCCAGGAATATGGCAATTAGATTGTACACATTTAGAAGGAAAAGTTATCCTGGTAGCAGTCCATGTAGCCAGTGGCTACATGGAAGCAGAAATTATTCCAGCAGAAACAGGGCAGGAAACAGCCTACTTTCTCTTGAAATTAGCAGGAAGATGGCCAGTAAAAGTAGTACATACAGACAATGGCAGCAATTTCACCAGCGCTGCAGTTAAGGCTGCCTGTTGGTGGGCAGGCATCAAACAGGAATTTGGGATTCCCTACAATCCCCAAAGTCAAGGAGTAGTAGAATCTATGAATAAAGAATTAAAGAAAATTATAGGACAGGTAAGAGACCAAGCTGAACATCTTAAGACAGCAGTACAGATGGCAGTATTCATTCACAATTTTAAAAGAAAAGGGGGGATTGGGGGGTACAGTGCAGGGGAAAGAATAATAGACATAATAGCAACAGACATACAAACTAAAGAATTACAAAAACAAATCATAAAAATTCAAAATTTTCGGGTTTATTACAGGGACAGCAGAGATCCAATTTGGAAAGGACCAGCAAAGCTTCTCTGGAAAGGTGAAGGGGCAGTAGTAATACAGGACAATAGTGAAATAAAGGTAGTACCAAGAAGAAAAGCAAAGATCATTAGGGATTATGGAAAACAGATGGCAGGTGATGATTGTGTGGCAAGTAGACAGGATGAGGAT

>AF484518

TTCTTGGATGGGATAGATAAGGCACAAGAAGAACATGAGAAATACCACAACAATTGGAGAGCAATGGCTAGTGATTTTAACCTGCCACCTGTGGTAGCAAAAGAAATAGTAGCTAGCTGTGATAAATGTCAGCTAAAAGGAGAAGCCTTGCATGGGCAAGTAGACTGTAGTCCAGGAATATGGCAATTAGATTGCACACATTTAGAAGGAAAAGTTATCCTGGTAGCAGTCCATGTAGCCAGTGGCTATATAGAAGCAGAAGTTATTCCAGCAGAAACAGGGCAGGAAACAGCCTACTTTCTCTTGAAATTAGCAGGAAGATGGCCAGTAAAAGTAGTACATACAGACAATGGCAGCAATTTCACCAGCGCTGCACTTAAGGCCGCCTGCTGGTGGGCAGGTATCAAGCAGGAATTTGGAATTCCCTACAATCCCCAAAGTCAAGGAGTAGTAGAATCTATGAATAAAGAATTAAAGAAAATTATAGGACAGGTAAGAGATCAAGCTGAACATCTTAAGACAGCAGTACAAATGGCAGTATTCATCCACAATTTTAAAAGAAAAGGGGGGATTGGGGGGTACAGTGCAGGGGAAAGAATAATAGATATAATAGCAACAGACATACAAACTAAAGAATTACAAAAACAAATCACAAAAATTCAAAATTTTCGGGTTTATTACAGGGACAACAGAGATCCAATTTGGAAAGGACCAGCAAAGCTTCTCTGGAAAGGTGAAGGGGCAGTAGTACTACAAGACAATAGTGAAATAAAGGTAGTGCCTAGGAGAAAAGTAAAGATCATTAGGGATTATGGAAAACAGATGGCAGGTGATGATTGTGTGGCAAGTAGACAGGATGAGGAT

>AF484517

TTTTTAGATGGGATAGATAAGGCTCAAGAAGAACATGAGAAATACCACAACAATTGGAAAGCAATGGCTAGTGATTTTAACCTGCCACCTGTGGTAGCAAAAGAAATAGTAGCTAGCTGTGATAAATGTCAGCTAAAAGGAGAAGCCTTGCATGGACAAGTAGACTGTAGTCCAGGAATATGGCAATTAGATTGCACACATTTAGAAGGAAAAATTATCATGGTAGCAGTCCATGTAGCCAGTGGCTATATAGAAGCAGAAGTTATTCCAGCAGAAAATGGACAGGAAACAGGCTATTTTATCTTGAAATTAGCAGGAAGGTGGCCAGTAAAAGTAGTACATACAGACAATGGCAGCAATTTCACCAGCGCTGCAGTGAAGGCCGCCTGTTGGTGGGCGGGCATTAAGCAGGAATTTGGAATTCCCTACAATCCCCAAAGTCAAGGAGTAGTAGAATCTATGAATAAAGAATTAAAGAAAATTATAGGACAGGTGAGAGATCAAGCTGAACACCTTAAGACAGCAGTACAAATGGCAGTATTCATTCACAATTTTAAAAGAAAAGGGGGGATTGGGGGGTACAGTGCAGGGGAAAGAATAATAGACATAATAGCAACAGATATACAAACTAGAGAATTACAAAAACAAATCATAAAAATTCAAAATTTTCGGGTTTATTACAGGGACAGCAGAGATCCAATTTGGAAAGGACCAGCAAAACTTCTCTGGAAAGGTGAAGGGGCAGTAGTAATACAAGACAATAGTGAAATAAAGGTAGTACCAAGAAGAAAAGCAAAGATCATTAGGGATTATGGAAAACAGATGGCAGGTGATGATTGTGTGGCAAGTAGACAGGATGAGGAT

>AF484516

TTCTTGGACGGAATAGATAAGGCTCAAGAGGAACATGAGAAATACCACAACAATTGGAGAGCAATGGCTAGTGATTTTAACCTGCCACCTGTGGTAGCAAAAGAAATAGTAGCTAGCTGTGATAAATGTCAGCTAAAAGGAGAAGCTTTACATGGACAAGTAGACTGTAGTCCAGGAATATGGCAATTAGATTGTACACATTTAGAAGGGAAAGTTATCCTGGTAGCAGTCCATGTAGCCAGTGGCTATATAGAAGCAGAAGTTATTCCAGCAGAAACAGGGCAGGAGACAGCCTACTTTCTCTTGAAATTAGCAGGAAGATGGCCAGTAAAAGTAGTACATACAGACAATGGCAGCAATTTCACCAGCGCTGCAGTTAAGGCCGCCTGTTGGTGGGCAGGCATCAAGCAGGAATTTGGAATTCCCTACAATCCCCAAAGTCAAGGAGTAGTAGAATCTATGAATAAAGAATTAAAAAAGATTATAGGACAGGTAAGAGATCAAGCTGAACATCTTAAGACAGCAGTACAAATGGCAGTATTCATTCACAATTTTAAAAGAAAAGGGGGGATTGGGGGGTACAGTGCAGGGGAAAGAATAATAGACATAATAGCAACAGACATACAAACTAAAGAATTACAAAAACAAATCATAAAAATTCAAAATTTTCGGGTTTATTACAGGGACAGCAGAGATCCAATTTGGAAAGGACCAGCAAAGCTTCTCTGGAAAGGTGAAGGGGCAGTAGTAATACAAGACAATAGTGAAATAAAGGTAGTACCAAGAAGAAAAGTAAAGATCATTAGGGATTATGGAAAACAGATGGCAGGTGATGATTGTGTGGCAAGTAGACAGGATGAGGAT

>AF484515

TTCTTGGATGGAATAGATAAGGCCCAAGAAGAACATGAGAAATACCACAACAATTGGAGAGCAATGGCTAGTGATTTTAACCTGCCACCTGTGGTAGCAAAAGAAATAGTAGCTAGCTGTGATAAATGTCAGCTAAAAGGAGAAGCCTTGCATGGACAAGTAGACTGTAGTCCAGGAATATGGCAATTAGATTGTACACATTTAGAAGGAAAAGTTATCCTGGTAGCAGTCCATGTAGCCAGTGGCTATATAGAAGCAGAAGTTATTCCAGCAGAAACAGGGCAGGAAACGGCCTACTTTCTCTTGAAATTAGCAGGAAGATGGCCAGTAAAAGTAGTACATACAGACAATGGCAGTAATTTCACCAGCACTGTAGTTAAGGCCGCCTGTTGGTGGGCAGGCATCAAGCAGGAATTTGGAATTCCCTACAATCCTCAAAGTCAAGGAGTAGTAGAATCTATGAATAAAGAATTAAAAAGAATTATAGGACAAGTAAGAGATCAAGCTGAACATCTTAAGACAGCAGTACAAATGGCAGTATTCATCCACAATTTTAAAAGAAAAGGGGGGATTGGGGGATACAGTGCAGGGGAAAGAATAATAGACATAATAGCAACAGACATACAAACTAAAGAATTACAAAAACAAATCACAAAAATTCAAAATTTTCGGGTTTATTACAGGGACAGCAGAGATCCAATTTGGAAAGGACCAGCAAAGCTTCTCTGGAAAGGTGAAGGGGCAGTAGTACTACAAGACAATAGTGAAATAAAGGTAGTACCAAGAAGAAAAGTAAAGATCATTAGGGATTATGGAAAACAGATGGCAGGCGATGATTGTGTGGCAAGTAGACAGGATGAGGAT

>AF484514

TTTTTGGATGGAATAGATAAGGCTCAAGAAGAACATGAGAAATACCACAACAATTGGAGAGCAATGGCTAGTGATTTTAACCTACCACCTGTGGTAGCGAAAGAAATAGTAGCTAGCTGTGATAAATGTCAGCTAAAAGGAGAAGCCATGCATGGACAAGTAGACTGTAGCCCAGGAATATGGCAATTAGATTGTACACATTTAGAAGGAAAAGTTATCCTGGTAGCAGTTCATGTAGCCAGTGGTTATATAGAAGCAGAAGTGATTCCAACAGAAACAGGGCAGGAAACAGCCTACTTTATCTTAAAACTAGCAGGAAGATGGCCAGTAAAAGTAGTACATACAGACAATGGCGGCAATTTCATCAGTGCTGCAGTTAAGGCCGCCTGCTGGTGGGCAGGCATTAAGCAGGAATTTGGAATTCCCTACAATCCCCAAAGTCAAGGAGTAGTGGAATCTATGAATAAAGAATTAAAGAAAATTATAGGACAGGTAAGAGATCAAGCTGAACATCTTAAGACAGCAGTACAAATGGCAGTATTCATCCACAATTTTAAAAGAAAAGGGGGGATTGGGGGGTACAGTGCAGGGGAAAGAATAATAGACATAATAGCAACAGACATACAAACTAAAGAACTACAAAAACAAATCATAAAAATTCAAAATTTTCGGGTTTATTACAGGGACAGCAGAGATCCAATTTGGAAAGGACCAGCAAAGCTTCTCTGGAAAGGTGAAGGGGCAGTAGTAATACAGGACAATAACGACATAAAGGTAGTACCAAGAAGAAAAGCAAAGATCATTAGGGATTATGGAAAACAGATGGCAGGTGATGATTGTGTGGCAAGTAGACAGGATGAGGAT

>AF484513

TTTTTAGATGGAATAGATAAGGCTCAAGAAGAACATGAGAAATACCACAACAATTGGAGAGCAATGGCTAGTGATTTTAACCTGCCACCTGTGGTAGCAAAAGAAATAGTAAGAAGCTGTGATAAATGTCAGCTAAAAGGAGAAGCCTTGCATGGACAAGTAGACTGTAGTCCAGGAATATGGCAATTAGATTGTACACATTTAGAAGGAAAAGTTATCCTGGTAGCAGTCCACGTAGCCAGTGGCTATATAGAGGCAGAAGTCATTCCAGCAGAGACAGGGCAGGAAACAGCCTACTTTCTCTTAAAATTAGCAGGAAGATGGCCAGTAAAAGTAGTACACACAGACAATGGCAGCAATTTCACCAGCACTGTAGTTAAGGCCGCCTGTTGGTGGGCAGGCATCAAGCAGGAATTTGGAATTCCCTACAATCCCCAAAGTCAAGGAGTAGTAGAATCTATGAATAAAGAATTAAAGAAAATTATAGGACAGGTAAGAGATCAAGCTGAACATCTTAAGACAGCAGTACAAATGGCAGTATTCATTCACAATTTTAAAAGAAAAGGGGGGATTGGGGGGTACAGTGCAGGGGAAAGAATAATAGACATAATAGCAACAGACTTACAAACCAGAGAATTACAAAAACAAATCATAAAAATTCAAAATTTTCGGGTTTATTACAGGGACAGCAGAGATCCAATTTGGAAAGGACCAGCAAAGCTTCTCTGGAAAGGTGAAGGGGCAGTAGTAATACAGGACAAGAGTGAAATAAAGGTAGTACCAAGAAGAAAAGCAAAGATCATTAGGGACTATGGAAAACAGATGGCAGGTGATGATTGTGTGGCAAGTAGACAGGATGAGGAT

>AF484512

TTAGATGGGATAGATAAAGCTCAAGAAGAACATGAAAAATATCACAGCAATTGGAGAACAATGGTTAGTGATTTTAATCTGCCACCTATAGTAGCAAAGGAAATAGTAGCCAGCTGTGATAAATGTCAGCTAAAAGGGGAAGCCATGCATGGACAAGTAGACTGCAGTCCAGGGATATGGCAATTAGATTGCACACATCTAGAAGGAAAAGTAATTATGGTAGCAGTTCATGTAGCCAGTGGCTATATAGAAGCAGAAGTTATCCCAGCAGAAACAGGACAAGAAACAGCATATTTTCTACTAAAATTAGCAGGAAGATGGCCAGTAAAAGTAGTACACACAGACAATGGCAGCAATTTCACCAGCGCTGCAGTAAAAGCAGCCTGTTGGTGGGCAAATATCCAACAGGAATTTGGGATTCCCTACAATCCCCAAAGTCAAGGAGTAGTAGAATCTATGAATAAGGAATTAAAGAAAATCATAGGGCAGGTAAGAGAGCAAGCTGAACACCTTAAAACAGCAGTACAAATGGCAGTATTCATTCACAATTTTAAAAGAAAAGGGGGGATTGGGGGGTACAGTGCAGGGGAAAGAATAATAGACATAATAGCAACAGACATACAAACTAAAGAACTACAAAAACAAATTACAAAAATTCAAAATTTTCGGGTTTATTACAGGGACAGCAGAGATCCAATTTGGAAAGGACCAGCAAAACTACTCTGGAAAGGTGAAGGGGCAGTGGTAATACAGGACAATAGTGATATAAAAGTAGTACCAAGAAGAAAAGCAAAGATCATCAGGGATTATGGAAAACAGATGGCAGGTGATGATTGTGTGGCAGGTAGACAGGATGAGGAT

>AF484511

TTTTTGGATGGAATAGATAAGGCTCAAGAAGAACATGAGAAATACCACAACAATTGGAGAGCAATGGCTAGTGATTTTAACCTGCCACCAGTGGTAGCAAAAGAAATAGTGGCTAGCTGTGATAAATGTCAACTAAAGGGAGAAGCCTTGCATGGACAAGTAGACTGTAGTCCAGGAATATGGCAATTAGATTGTACACATTTAGAAGGAAAAGTTATTCTGGTAGCAGTCCATGTAGCCAGTGGCTATATAGAAGCAGAAGTCATTCCAGCAGAAACAGGGCAGGAAACAGCCTATTTTCTCTTAAAATTAGCAGGAAGATGGCCAGTAAAAGTAGTACATACAGACAATGGCAGCAATTTCACCAGCACTGTAGTTAAGGCCGCCTGTTGGTGGGCAGGCATCAAGCAGGAATTTGGAATTCCCTACAATCCCCAAAGTCAGGGAGTAGTAGAATCCATGAATAATGAACTAAAGAAAATTATAGGACAGGTAAGAGATCAAGCTGAACATCTTAAGACAGCAGTACAAATGGCAGTATTCATCCACAATTTTAAAAGAAAAGGGGGGATTGGGGGGTACAGTGCAGGGGAAAGAATAATAGACATAATAGCAACAGACATACAAACTAAAGAATTACAAAAACAAATTACAAAAATTCGAAATTTTCGGGTTTATTACAGGGACAGCAGAGATCCAATTTGGAAAGGACCAGCAAAGCTGCTCTGGAAAGGTGAAGGGGCAGTAGTAATACAAGACAATAGTGAAATAAAAGTAGTACCAAGAAGAAAAGCAAAAATCATTAGGGATTATGGAAAACAGATGGCAGGTGATGATTGTGTGGCAGGTAGACAGGATGAGGAT

>AF484510

TTCTTGGATGGAATAGATAAGGCTCAAGAAGAACATGAGAAATACCACAACAATTGGAGAGCAATGGCTAGTGATTTTAACCTGCCACCTGTGGTAGCAAAAGAAATAGTAGCTAGCTGTGATAAATGTCAGCTAAAAGGAGAAGCCTTGCATGGACAAGTAGACTGTAGTCCAGGAATATGGCAATTAGATTGTACCCATTTAGAAGGAAAAATTATCCTGGTAGCAGTACATGTAGCCAGTGGCTATATAGAAGCAGAAGTTATTCCAGCAGAAACAGGGCAGGAAACAGCCTACTTTATCTTGAAATTAGCAGGAAGATGGCCAGTAAAAGTAGTACATACAGACAATGGCAGCAATTTCACCAGCGCTGCAGTTAAGGCCGCCTGTTGGTGGGCAGGCATCAAGCAAGAATTTGGAATTCCCTACAATCCCCAAAGTCAAGGAGTAGTAGAATCTATGAATAAAGAATTAAAGAAAATTATAGGACAGGTAAGAGAGCAAGCTGAACATCTTAAGACAGCAGTACAAATGGCAGTATTCATCCACAATTTTAAAAGAAGAGGGGGGATTGGGGGGTACAGTGCAGGGGAGAGAATAATAGACATAATAGCAACAGACATACAAACTAAAGAATTACAAAAACAAATCACAAAAATTCAAAATTTTCGGGTTTATTACAGGGACAGCAGAGATCCAATTTGGAAAGGACCAGCAAAACTTCTCTGGAAAGGTGAAGGGGCAGTAGTAATACAAGACAATAGTGACATAAAGGTAGTTCCAAGAAGAAAAGTGAAGATTATTAGGGATTATGGAAAACAGATGGCAGGTGATGATTGTGTGGCAGGTAGACAGGATGAGGAT

>AF484509

TTTTTAGATGGGATAGATAAGGCTCAAGAAGAACATGAAAGATATCACAGCAATTGGAGAGCAATGGCTAGTGATTTTAATCTGCCACCTATAGTAGCAAAGGAAATAGTAGCCAGCTGTGATAAATGTCAACTAAAAGGGGAAGCCATGCATGGACAGGTAGACTGTAGTCCAGGGATGTGGCAATTAGATTGCACACATTTAGAAGGGAAAGTAATTCTGGTAGCAGTCCATGTAGCCAGTGGCTATATAGAAGCAGAAGTTATCCCAGCAGAAACAGGGCAGGAGGCAGCATACTTTTTGCTAAAATTAGCAGGAGGATGGCCAGTAAAAGTAGTACACACAGACAATGGCAGCAATTTTACCAGCGCTGCATTTAAAGCAGCCTGTTGGTGGGCAAACATCCAACAGGAATTTGGGATTCCCTACAATCCCCAAAGTCAAGGAGTGGTAGAATCTATGAATAAGGAATTAAAGAAAATCATAGGGCAGATAAGAGAGCAAGCTGAACACCTTAAAACAGCAGTACAAATGGCAGTATTCATTCACAATTTTAAAAGAAAAGGGGGGATTGGGGGGTACAGTGCAGGGGAAAGAATAATAGACATAATAGCAACAGACATACAAACTAAAGAATTACAAAAACAAATTACAAAAATTCAAAATTTTCGGGTTTATTACAGGGACAGCAGAGATCCAGTTTGGAAAGGACCAGCAAAACTACTCTGGAAAGGTGAAGGGGCAATAGTAATACAGGACAATAGTGATATAAAGGTAGTGCCCAGAAGAAAAGCAAAGATCATTAGGGATTATGGAAAACAGATGGCAGGTGATGATTGTGTGGCAGGTAGACAGGATGAGGAT

>AF484508

TTTTTAGATGGGATAGATAAGGCTCAAGAAGAACATGAAAGATATCACAGCAATTGGAGAGCAATGGCTAGTGATTTTAATCTGCCACCTATAGTAGCAAAGGAAATAGTAGCCAGCTGTGATAAATGTCAACTAAAAGGGGAAGCCATACATGGACAAGTAGACTGCAGTCCAGGGATGTGGCAATTAGATTGCACACATCTAGAAGGAAAAGTAATTCTGGTAGCAGTCCATGTAGCCAGTGGATATATAGAGGCAGAACTTATCCCAGCAGAAACAGGACAGGAGGCAGCATACTTTCTGCTAAAATTAGCAGGAAGGTGGCCAGTGAAAACAGTACACACAGACAATGGCAGCAATTTCACCAGCGCTGCATTTAAAGCAGCCTGTTGGTGGGCAAATGTCCAACAGGAATATGGGATTCCCTACAATCCCCAAAGTCAAGGAGTAGTGGAATCTATGAATAAAGAATTAAAGAAAATCATAGGGCAAGTAAGAGAGCAAGCTGAACACCTTAAGACAGCAGTACAAATGGCAGTATTCATTCGCAATTTTAAAAGAAAAGGGGGGATTGGGGGGTACAGTGCAGGGGAAAGAATAATAGACATAATAGCAACAGACATACAAACTAAAGAACTACAAAAACAAATTATAAAAATTCAAAATTTTCGGGTTTATTACAGGGACAGCAGAGATCCACTTTGGAAAGGACCAGCAAAACTACTCTGGAAAGGTGAAGGGGCAGTAGTAATACAGGACAATAGTGAGATAAAGGTAGTGCCCAGAAGAAAAGCAAAGATCATTAGGGATTATGGAAAACAGATGGCAGGTGATGATTGTGTGGTAGGTAGACAGGATGAGGAT

>AF484507

TTCTTAGATGGGATAGATAAAGCTCAAGAAGAACATGAAAGGTATCACAGCAATTGGAGAACAATGGCTAGTGATTTTAATCTGCCACCTATAGTAGCAAAGGAAATAGTAGCCAGCTGTGATAAATGTCAGCTAAAAGGGGAAGCCATGCATGGACAAGTAGACTGCAGTCCTGGGATATGGCAATTAGATTGCACACATCTAGAAGGAAAAATAATTCTGGTAGCAGTCCATGTAGCCAGTGGCTATATAGAAGCAGAAGTTATCCCAGCAGAAACAGGACAGGAGGCAGCATACTTTATACTAAAATTAGCAGGAAGATGGCCAGTAAAAGTAGTACACACAGACAATGGCAGCAATTTCACCAGCGCTGCATTCAAAGCAGCCTGTTGGTGAGCAAATGTCCAACAGGAATTTGGAATTCCCTACAATCCCCAAAGTCAAGGAGTAGTAGAATCTATGAATAAGGAATTAAAGAAAATCATAGGGCAGGTAAGAGAGCAAGCTGAGCACCTTAAGACAGCAGTACAAATGGCAGTATTCATTCACAATTTTAAAAGAAAAGGGGGGATTGGGGGGTACAGTGCAGGGGAAAGAATAATAGACATAATAGCATCAGATATACAAACTAGAGAATTACAAAAACAAATTACAAATATTCAAAAATTTCGGGTTTATTACAGGGACAGCAGAGATCCAATTTGGAAAGGACCAGCAAAACTACTCTGGAAAGGTGAAGGGGCAGTGGTAATACAAGACAATAGCGATATAAAAGTAGTACCAAGAAGAAAAGCAAAGATCATTAGGGATTATGGAAAACAGATGGCAGGTGATGATTGTGTGGCAGGTAGACAGGATGAAGAT

>AF484506

TTTTTGGATGGAATAGATAAGGCTCAAGAAGAACATGAGAAATACCACAACAATTGGAGAGCAATGGCTAGTGATTTTAATCTGCCACCTGTGGTAGCAAAAGAGATAGTAGCCAGCTGTGATAAATGTCAGCTAAAAGGAGAAGCTTTGCATGGACAAGTAGACTGTAGTCCAGGAATATGGCAATTAGATTGTACACATTTAGAAGGAAAAGTTATCCTGGTAGCAGTCCATGTAGCCAGTGGCTATATAGAAGCAGAAGTCATTCCAGCAGAAACAGGGCAGGAAACAGCCTACTTTCTCTTAAAATTAGCAGGAAGATGGCCAGTAAAAGTAGTACACACAGACAATGGCAGCAATTTCACCAGCGCTGCAGTTAAGGCAGCCTGCTGGTGGGCAGGCATCAAGCAGGAATTTGGGATTCCCTACAATCCCCAAAGTCAAGGAGTAGTAGAATCTATGAATAAAGAATTAAAGAAAATCATAGGACAGGTAAGAGAGCAAGCTGAACATCTTAAGACAGCAGTACAAATGGCAGTATTCATTCACAATTTTAAAAGAAAAGGGGGGATTGGGGGGTACAGTGCAGGGGAAAGAATAATAGACATAATAGCAACAGACATACAAACTAAAGAACTACAAAAACAAATTATAAAAATTCAAAATTTTCGGGTTTATTACAGGGACAGCAGAGATCCAATTTGGAAAGGACCAGCAAAGCTTCTCTGGAAAGGTGAAGGAGCAGTAGTAATACAAGACAATAGTGAAATAAAGGTAGTACCTAGAAGGAAAGTAAAGATCATTAGGGATTATGGAAAACAGATGGCAGGTGATGATTGTGTGGCAAGTAGACAGGATGAGGAT

>AF484505

TTCTTGGATGGAATAGATAAGGCTCAAGAGGACCATGAGAAATACCACAGCAATTGGAGAGCAATGGCTAGTGATTTCAACCTGCCACCTGTGGTAGCAAAAGAAATAATAGCTAGCTGTGATAAATGTCAGCTAAAAGGAGAAGCCTTGCATGGACAAGTAGACTGTAGTCCAGGAATATGGCAATTAGATTGTACACACTTAGAAGGAAAAGTTATCCTGGTAGCAGTCCATGTAGCTAGTGGCTATATAGAAGCAGAAGTTATTCCAGCAGAAACAGGGCAGGAAACAGCCTACTTCATCTTGAAATTAGCAGGAAGATGGCCAGTAAAAGTAATACATACAGACAATGGCAGCAATTTCACCAGCACCACAGTTAAGGCCGCCTGTTGGTGGGCAGGCATCAAGCAGGAATTTGGAATTCCCTACAATCCCCAAAGTCAGGGAGTAGTAGAATCTATGAATAAGGAATTAAAGAAAATTATAGGACAGGTAAGGGATCAAGCTGAACATCTTAAGACAGCAGTACAAATGGCAGTATTCATCCACAATTTTAAAAGAAAAGGGGGGATTGGGGGGTACAGTGCAGGGGAAAGAATAATAGACATAATAGCAACAGACATACAAACTAAAGAATTACAAAAACAAATCACAAAAATTCAAAATTTTCGGGTTTATTACAGGGACAGCAGAGATCCAATTTGGAAAGGACCAGCAAAGCTTCTCTGGAAAGGTGGAGGGGCAGTAGTAATACAAGACAATAGTGAAATAAAGGTGGTACCAAGAAGAAAAGCAAAGATCATTAGGGATTATGGAAAACAGATGGCAGGTGATGATTGTGTGGCAAGTAGACAGGATGAGGAT

>AF484504

TTTTTGGATGGAATAGATAAGGCTCAAGAAGAACATGAAAAATACCACAACAATTGGAGAGCAATGGCTAGTGATTTTAACCTGCCACCTGTGGTAGCGAAAGAAATAGTAGCTAGCTGTGATAAATGTCAGCTAAAAGGAGAAGCCATGCATGGACAAGTAGACTGTAGTCCAGGAATATGGCAACTAGATTGTACACATCTAGAAGGAAAAGTTATCCTGGTAGCAGTTCATGTAGCCAGTGGCTATATAGAAGCAGAAGTGATCCCAGCAGAAACAGGGCAGGAAACAGCCTACTTTATCTTAAAATTAGCAGGAAGATGGCCAGTAAAAGTAGTACATACAGACAATGGCAGCAATTTCACCAGTTCTGCAGTTAAGGCCGCCTGTTGGTGGGCAGGCGTTAAGCAGGAATTTGGAATTCCCTACAATCCCCAAAGTCAAGGAGTAGTGGAATCTATGAATAAAGAATTAAAGAAAATTATAGGACAGGTAAGAGATCAAGCTGAACATCTTAAGACAGCAGTACAAATGGCAGTATTCATTCACAATTTTAAAAGAAAAGGGGGGATTGGGGGGTACAGTGCAGGGGAAAGAATAATAGACATAATAGCAACAGACTTACAAACTAAAGAATTACAAAAACAAATCATAAAAATTCAAAATTTTCGGGTTTATTACAGGGACAGCAGAGATCCAATTTGGAAAGGACCAGCAAAGCTTCTCTGGAAAGGTGAAGGGGCAGTAGTAATACAAGATAATAGTGACATAAAGGTAGTACCAAGAAGAAAAGTAAAGATCATTAGGGATTATGGAAAACAGATGGCAGGTGATGATTGTGTGGCAAGTAGACAGGATGAGGAT

>AF484503

TTTTTAGATGGGATAGATAAGGCTCAAGAAGAACATGAAAGATATCACAGCAATTGGAGGGCAATGGCTAGTGATTTTAATCTGCCACCTATAGTAGCAAAGGAAATAGTAGCCAGCTGTGATAAATGTCAACTAAAAGGGGAAGCCATGCATGGACAAGTAGACTGTAGTCCAGGAATGTGGCAATTAGATTGCACACATCTAGAAGGAAAAGTAATTCTGGTAGCAGTCCATGTAGCCAGTGGTTATATAGAAGCAGAAGTTATCCCAGCAGAAACAGGACAGGAGACAGCATACTTTCTGCTAAAATTAGCTGGAAGATGGCCAGTAAAAGTAGTACACACAGATAATGGCAGCAATTTCACCAGCGCTGCCTTTAAAGCAGCCTGTTGGTGGGCAAATGTCCAACAGGAGTATGGGATCCCCTACAATCCCCAAAGTCAAGGAGTAGTGGAATCTATGAACAAGGAATTAAAGAAAATCATAGGGCAGGTAAGGGAGCAGGCTGAACACCTCAAGACAGCAGTACAAATGGCAGTATTCATTCACAATTTTAAAAGAAAAGGGGGGATTGGGGACTATAGTGCAGGGGAAAGAATAATAGACATAATAGCAACAGACATACAAACTAGAGAACTACAAAAACAAATTACAAAAATTCAAAATTTTCGGGTTTATTACAGGGACAGCAGAGATCCACTTTGGAAAGGACCAGCAAAACTACTCTGGAAAGGTGAAGGGGCAGTAGTAATACAGGACAATAGTGATATAAAGGTAGTGCCCAGAAGAAAAGCAAAGATCCTTAGGGATTATGGAAAACAGATGGCAGGTAATGATTGTGTGGCAGGTAGACAGAATGAGGAT

>AF484502

TTCTTGGATGGAGTAGATAAGGCTCAAGAAGAACATGAGAAATACCACAACAATTGGAGAGCTATGGCTAGTGATTTTAACCTGCCACCTGTGGTAGCAAAAGAAATAGTAGCTAGCTGTGATAAATGTCAGCTGAAAGGAGAAGCCTTGCATGGACAAGTAGACTGTAGTCCAGGAATATGGCAATTAGATTGTACACATTTAGGAGGAAAAGTTATCCTGGTAGCAGTCCATGTAGCCAGTGGCTATATAGAAGCAGAAGTCATTCCAGCAGAAACAGGACAGGAAACAGCCTACTTTCTTTTAAAATTAGCAGGAAGGTGGCCAGTAAAAATAGTGCATACAGACAATGGCAGCAATTTCACCAGCGCTGCAGTGAAGGCCGCCTGTTGGTGGGCAGGCATCAAGCAGGAATTTGGAATTCCCTACAATCCCCAAAGTCAAGGGGTAGTAGAATCTATGAATAAAGAATTAAAAAAGATTATAGGGCAGGTAAGAGATCAAGCTGAACATCTTAAGGCAGCAGTACAAATGGCAGTATTCATCCACAATTTTAAAAGAAAAGGGGGGATTGGGGGGTACAGTGCAGGGGAAAGAATAATAGACATAATAGCAACAGACATACAAACTAAAGAATTACAGAAACAAATCATAAAAATTCAAAATTTTCGGGTTTATTACAGGGACAGCAGAGATCCAATTTGGAAAGGACCAGCAAAACTTCTCTGGAAAGGTGAAGGGGCAGTAGTAATACAAGACAATAGTGAAATAAAGGTAGTACCAAGAAGAAAAGCAAAGATCATTAGGGATTATGGAAAACAGATGGCAGGTGATGATTGTGTGGCAAGTAGACAGGATGAGGAT

>AF484501

TTTTTAGATGGGATAGATAAGGCTCAAGAAGACCATGAAAGATATCACAGCAATTGGAGAACAATGGCTAGTGATTTTAATCTGCCACCTATAGTAGCAAAGGAAATAGTAGCCAGCTGTGATAAATGTCAACTAAAAGGGGAGGCCATGCATGGACAAGTAGACTGTAGTCCAGGGATGTGGCAATTAGATTGCACACATCTAGAAGGAAAAGTAATTCTGGTAGCAGTCCATGTAGCCAGTGGCTATATAGAGGCAGAAGTTATTCCAGCAGAAACAGGACAGGAGACAGCATACTTTCTGCTAAAATTAGCAGGAAGATGGCCAGTAAAAGTAGTACACACAGACAATGGCAGCAATTTCACCAGTGCTGCATTTAAAGCAGCCTGTTGGTGGGCAAATGTCCAACAGGAATTTGGAATTCCCTACAATCCCCAAAGTCAAGGAGTAGTGGAATCTATGAATAAGGAATTAAAGAAAATCATAGGGCAGGTACGAGAGCAAGCTGAACATCTTAAGACAGCAGTACAAATGGCAGTATTCATTCACAATTTTAAAAGAAAAGGGGGGATTGGGGGGTACAGTGCAGGGGAAAGAATAATAGACATAATAGCAACAGACATACAAACTAAGGAACTACAAAAACAAATTATAAAAATTCAAAATTTTCGGGTTTATTACAGGGACAGCAGAGATCCAATTTGGAAAGGACCAGCAAAACTACTCTGGAAAGGTGAAGGAGCAGTAGTAATACAGGACAATAGTGATATAAAGGTAGTGCCCAGAAGAAAAGCAAAGATCATTAGGGATTATGGAAAACAGATGGCAGGTGATGATTGTGTGGCAGGTAGACAGGATGAAGAT

>AF484500

TTCTTGGATGGGATAGATAAGGCTCAAGAAGAACATGAAAAATACCATAACAATTGGAGGGCAATGGCTAGTGATTTTAACCTGCCACCAGTGGTAGCAAAAGAAATAGTAGCTAGCTGTGATAAATGTCATCTGAAAGGAGAAGCCTTGCATGGACAAGTAGACTGTAGTCCAGGAATATGGCAATTAGATTGTACACATTTAGAAGGAAAAGTTATCCTGGTAGCAGTCCATGTAGCCAGTGGCTATATAGAAGCAGAAGTTATTCCAGCAGAAACAGGACAGGAAACAGCCTACTTTCTCTTGAAATTAGCTGGAAGATGGCCAGTAAAAGTAGTACATACAGACAATGGCAGTAATTTCACCAGCGCTGCAGTTAAGGCCGCCTGTTGGTGGGCAGGCATCAAGCAGGAATTTGGAATTCCCTACAATCCCCAAAGTCAAGGAGTAGTAGAATCTATGAATAAAGAATTAAAGAAAATCATAGGACAGGTAAGAGAGCAAGCAGAACATCTTAAGACAGCAGTACAAATGGCAGTATTCATCCACAATTTTAAAAGAAAAGGGGGGATTGGGGGGTACAGTGCAGGGGAAAGAATAATAGACATAATAGCAACAGACATACAAACTAAAGAATTACAAAAACAAGTCATAAAAATTCAAAATTTTCGGGTTTATTACAGGGACAGCAGAGATCCAATTTGGAAAGGACCAGCAAAGCTTCTCTGGAAAGGTGAAGGGGCAGTAGTAATACAAGACAATAGTGAAATAAAGGTAGTACCAAGAAGAAAAGCAAAGATCATTAGGGATTATGGAAAACAGATGGCAGGTGATGATTGTGTGGCAAGTAGACAGGATGAGGAT

>AF484499

TTCTTGGATGGAATAGATAAGGCCCAAGAAGAACATGAGAAATACCACAACAATTGGAGAGCAATGGCTAGTGATTTTAACCTGCCACCTGTAGTGGCAAAAGAAATAGTAGCTAGCTGTGATAAATGTCAGCTAAAAGGAGAAGCCTTGCATGGACAAGTAGACTGTAGTCCAGGAATATGGCAATTAGATTGTACACATTTAGAAGGCAAAGTTATCCTGGTAGCAGTCCATGTGGCCAGTGGCTATATAGAAGCAGAAGTTATTCCAGCAGAAACAGGGCAGGAAACAGCCTACTTTCTCTTGAAGTTAGCAGGAAGATGGCCAGTAAAAGTAGTACATACAGACAATGGCAGTAATTTCACCAGCGCTGCAGTTAAGGCCGCCTGTTGGTGGGCAGGCATCAAGCAGGAATTTGGAATTCCCTACAATCCCCAAAGTCAAGGAGTAGTAGAATCTATGAATAATCAATTAAAGAAAATTATTGGACAGGTAAGAGAGCAAGCTGAACATCTTAAGACAGCAGTACAAATGGCAGTATTCATCCACAATTTTAAAAGAAAAGGGGGGATTGGGGGGTACAGTGCAGGGGAAAGAATAATAGACATAATAGCAACAGACATACAAACTAAAGAATTACAAAAACAAATCATAAAAATTCAAAATTTTCGGGTTTATTACAGGGACAGCAGAGATCCAATTTGGAAAGGACCAGCAAAGCTTCTCTGGAAAGGTGAAGGGGCAGTAGTGATACAAGACAATAGTGAAATAAAGGTAGTACCAAGAAGAAAAGTAAAGATCATTAGGGATTATGGAAAACAAATGGCAGGTGATGATTGTGTGGCAAGTAGACAGGATGAGGAT

>AF484498

TTCTTGGATGGAATAGATAAGGCTCAAGAAGAACATGAGAAATACCACAATAATTGGAGAGCAATGGCTAGTGACTTTAACATACCACCTGTGGTAGCAAAAGAAATAGTAGCTAGCTGTGACAAATGCCAGCTAAAAGGAGAAGCCTTGCATGGACAAGTAGACTGTAGTCCAGGAATATGGCAATTAGATTGTACACATTTAGAAGGAAAAATTATCCTGGTAGCAGTCCATGTAGCCAGTGGCTATATAGAAGCAGAAGTTATTCCAGCAGAAACAGGGCAGGAGGCAGCATACTTTCTCTTGAAATTAGCCGGAAGATGGCCAGTAAGAGTAATACATACAGACAATGGCAGCAATTTCACCAGCAATGCACTTAAGGCCGCTTGTTGGTGGGCAGGCATCAAGCAGGAATTTGGAATTCCCTATAATCCCCAAAGTCAAGGTGTGGTAGAATCTATGAATAAAGAATTAAAGAAAATTATAAAACAGGTAAGAGATCAAGCTGAACATCTTAAGACAGCAGTACAAATGGCAGTATTCATCCACAATTTTAAAAGAAAAGGGGGGATTGGGGGATACAGTGCAGGGGAAAGAATAATAGACATAATAGCAACAGACATACAAACTAAAGAATTACAAAAACAAATCACAAAAATTCAAAATTTTCGGGTTTATTACAGGGACAGCAGAGATCCAATTTGGAAAGGACCAGCAAAACTTCTCTGGAAAGGTGAAGGGGCAGTAGTAATACAAGACAATAGTGACATAAAGGTAGTACCAAGAAGAAAAGCAAAAATCATTAGGGATTATGGAAAACAGATGGCAGGTGATGATTGTGTGGCAAGTAGACAGGATGAGGAT

>AF484497

TTCTTGGATGGAATAGATAAGGCTCAAGAAGAACATGAAAAATACCACAACAATTGGAGAGCAATGGCTAGTGATTTTAACCTGCCACCTGTGGTAGCAAAAGAAATAGTAGCTAGCTGTGATAAATGTCAGCTGAAAGGAGAAGCCTTGCATGGACAAGTAGACTGTAGCCCAGGAATATGGCAATTAGATTGTACACATTTAGAAGGAAAAGTTATCCTGGTAGCAGTCCATGTAGCCAGTGGCTATATAGAAGCAGAAGTTATTCCAGCAGAAACAGGGCAGGAAACAGCCTACTTTCTCTTAAAATTAGCAGGAAGATGGCCAGTAAAAGTAGTACATACAGACAATGGCAGCAATTTCACCAGCGCTGCAGTTAAGGCTGCCTGTTGGTGGGCAGGCATCAAGCAGGAATTTGGAATTCCCTACAATCCCCAAAGTCAAGGAGTAGTAGAATCTATGAATAAAGAATTAAAGAAAATTATAGGACAGGTAAGAGAGCAAGCTGAACATCTTAAGACAGCAGTACAAATGGCAGTATTCATCCACAATTTTAAAAGAAAAGGGGGGATTGGGGGGTACAGTGCAGGGGAAAGAATAATAGATATAATAGCAACAGACATACAAACTAAAGAATTACAAAAACAAATCATAAAAATTCAAAATTTTCGGGTTTATTACAGGGACAGCAGAGATCCAATTTGGAAAGGACCAGCAAAGCTTCTCTGGAAAGGTGAAGGGGCAGTAGTAATACAAGACAATAGTGAAATAAAAGTAGTACCAAGAAGAAAAGTAAAGATCATTAGGGATTATGGAAAACAGATGGCAGGTGATGATTGTGTGGCAAGTAGACAGGATGAGGAT

>AF484496

TTCCTGGATGGAATAGATAAGGCTCAGGAAGAACATGAGAAATATCACAATAATTGGAGAGCAATGGCTAGTGATTTTAACCTGCCACCTGTGGTAGCAAAAGAAATAGTAGCTAGCTGTGATAAATGTCAGCTAAAAGGAGAAGCCTTGCATGGACAAGTAGACTGTAGTCCAGGAATATGGCAATTAGATTGCACACATCTAGAAGGAAAAGTAATCCTGGTAGCAGTCCATGTAGCCAGTGGTTATATAGAAGCAGAAGTTATCCCAGCAGAAACAGGGCAGGAAACAGCCTACTTCCTCTTAAAATTAGCAGGAAGATGGCCAGTGAAAGTAGTACATACAGACAATGGCAGCAATTTCACCAGCGCTACAGTTAAGGCCGCCTGTTGGTGGGCAGGCATCAAGCAGGAATTTGGAATTCCCTACAATCCCCAAAGTCAAGGAGTAGTAGAATCTATGAATAAAGAATTAAAAAAGATTATAGGACAGGTAAGAGATCAGGCTGAACATCTTAAGACAGCAGTACAAATGGCAGTATTCATCCACAATTTTAAAAGAAAAGGGGGGATTGGGGGGTACAGTGCAGGGGAAAGAATAATAGACATAATAGCAACAGACATACAAACTAAAGAATTACAAAAACAAATTTCAAATATTCAAAAATTTCGGGTTTATTACAGGGACAGCAGAGATCCAATTTGGAAAGGACCAGCAAAACTACTCTGGAAAGGTGAAGGGGCGGTAGTAATACAGGACAACAGTGATATAAAGGTAGTACCAAGAAGGAAAGCAAAGATCATTAGGGATTATGGAAAACAAATGGCAGGTGATGATTGTGTGGCAGGTAGACAGGATGAGGAT

>AF484495

TTCTTGGATGGAATAGATAAGGCTCAAGAAGAACATGAGAAATACCACAACAATTGGAGAGCAATGGCTAGTGATTTTAATCTGCCACCTGTGGTAGCAAAAGAAATAGTAGCTAGCTGTGATAAATGTCAGATAAAAGGAGAAGCCTTGCATGGACAAGTAGACTGCAGTCCAGGAATATGGCAATTGGATTGTACACATTTAGAAGGAAAAGTTATCCTGGTAGCAGTCCATGTAGCCAGTGGCTATATAGAAGCAGAAGTTATTTCAGCAGAAACAGGGCAGGAAACAGCATACTTCCTCTTAAAACTAGCAGGAAGATGGCCAGTAAAAGTAGTACATACAGACAATGGCAGTAATTTCACCAGCGCTGCAGTTAAGGCCGCCTGTTGGTGGGCAGGCATCAAGCAGGAATTTGGAATTCCCTACAATCCCCAAAGTCAAGGAGTAGTAGAATCTATGAATAAAGAATTAAAGAAAATTATAGGACAGGTAAGAGAGCAAGCTGAACATCTTAAAACAGCAGTACAAATGGCAGTATTCATTCACAATTTTAAAAGAAAAGGGGGGATTGGGGGGTACAGTGCAGGGGAAAGAATAATAGACATAATAGCTACAGACATACAAACTAAAGAATTACAAAAACAAATCACAAAAATTCAAAATTTTCGGGTTTATTACAGGGACAGCAGAGATCCAATTTGGAAAGGACCAGCAAAGCTTCTCTGGAAAGGTGAAGGGGCAGTAGTAATACAAGACAATAGTGACATAAAGGTAGTACCAAGAAGAAAGGTAAAAATCATTAGGGATTATGGAAAACAGATGGCAGGTGATGATTGTGTGGCAAGTAGACAGGATGAGGAT

>AF484494

TTCTTGGATGGTATAGATAAGGCTCAAGAAGAACATGAGAAATACCACAACAATTGGAGAGCAATGGCTAGTGATTTTAACCTGCCACCTGTGGTAGCAAAAGAAATAGTAGCTAGCTGTGATAAATGTCAGCTAAAAGGAGAAGCCTTGCATGGACAAGTAGACTGTAGTCCAGGAATATGGCAATTAGATTGTACACATTTAGAAGGAAAAGTTATCCTGGTAGCAGTCCATGTAGCTAGTGGCTATATAGAAGCAGAAGTTATTCCAGCAGAAACAGGGCAGGAAACAGCCTACTTTCTCTTGAAGTTAGCAGGAAGATGGCCAGTAAAAGTAGTACATACAGACAATGGCAGCAATTTCACCAGCTCTGCAGTTAAGGCCGCCTGTTGGTGGGCAGGCATCAAGCAGGAATTTGGAATTCCCTACAATCCCCAAAGTCAAGGAGTAGTAGAATCTATGAATAAAGAATTAAAGAAAATTATAGGACAGGTAAGAGATCAAGCTGAACATCTTAAGACAGCAGTACAAATGGCAGTATTTATCCACAATTTTAAAAGAAAAGGGGGGATTGGGGGATACAGTGCAGGGGAAAGAATAATAGACATAATAGCAACAGACATACAAACTAAAGAATTACAAAAACAAATCACAAAAATTCAAAATTTTCGGGTTTATTACAGGGACAGCAGAGATCCAATTTGGAAAGGACCAGCAAAGCTTCTCTGGAAAGGTGAAGGGGCAGTAGTAATACAAGACAATAGTGAAATAAAGGTAGTACCAAGAAGAAAAGTAAAGATCATTAGGGATTATGGAAAACAGATGGCAGGTGATGATTGTGTGGCAAGTAGACAGGATGAGGAT

>AF484493

TTTTTAGATGGGATAGATAAAGCTCAAGAAGAACATGAAAGATATCACAGCAATTGGAGAACAATGGCTAGTGATTTTAATTTGCCACCTATAGTAGCAAAGGAAATAGTAGCCAGCTGTGATAAATGTCAGCTAAAAGGGGAAGCTATACATGGACAAGTAGACTGCAGTCCAGGGATGTGGCAATTAGATTGCACACATCTAGAAGGAAAAATAATTCTGGTAGCAGTCCATGTAGCTAGTGGCTATATAGAAGCAGAAGTTATCCCAGCAGAAACAGGACAGGAGACAGCATACTTTCTGCTAAAGTTAGCAGGAAGATGGCCAGTAAAAGTAGTACACACAGACAATGGCAGCAATTTCACCAGCGCTGCATTTAAAGCAGCCTGTTGGTGGGCAAATGTCCAACAAGAATATGGGATTCCCTACAATCCCCAAAGTCAAGGAGTAGTGGAATCTATGAATAAGGAATTAAAGAAAATCATAGGACAGGTAAGAGAGCAAGCTGAACACCTTAAGACAGCAGTACAAATGGCAGTGTTCATTCACAATTTTAAAAGAAAAGGGGGGATTGGGGGGTACAGTGCAGGAGAAAGAATAATAGACATAATAGCAACAGACATACAAACTAGAGAATTACAAAAACAAATTACAAATATTCAAAAATTTCGGGTTTATTACAGGGACAGCAGAGATCCAATTTGGAAAGGACCAGCAAAACTACTCTGGAAAGGTGAAGGGGCAGTGGTAATACAGGACAATAGTGATATAAAGGTAATACCAAGAAGAAAAGCAAAGATCATTAGGGATTATGGAAAACAGATGGCAGGTGATGATTGTGTGGCAGGTAGACAGGATGAGGAT

>AF484492

TTCTTGGATGGAATAGATAAGGCACAAGAAGAACATGAGAAATATCACAGCAATTGGAGAGCAATGGCTAGTGATTTTAACCTGCCACCTGTGGTAGCAAAAGAAATAGTAGCTAGCTGTGATAAATGTCAGCTAAAAGGAGAAGCCTTGCATGGACAAGTAGACTGTAGTCCAGGAATATGGCAATTAGATTGTACACATTTAGAAGGAAAAGTTATCCTGGTAGCAGTCCATGTAGCCAGTGGCTATATAGAAGCAGAAGTTATTCCAGCAGAAACAGGGCAGGAAACAGCATACTTCATCTTAAAATTAGCAGGAAGATGGCCAGTAAAAGTAGTGCATACAGACAATGGCAGCAATTTCACCAGTAATGTAGTTAAGGCCGCCTGTTGGTGGGCAGGAATCAAGCAGGAATTTGGAATTCCCTACAATCCCCAAAGTCAAGGAGTAGTAGAATCTATGAATAAAGAATTAAAGAAAATTATAGGACAGGTAAGAGATCAAGCTGAACATCTTAAGACAGCAGTACAAATGGCAGTATTCATCCACAATTTTAAAAGAAAAGGGGGGATTGGGGGGTACAGTGCAGGGGAAAGAATAATAGACATAATAGCAACAGACATACAAACTAGAGAACTACAAAAACAAATTACAAAAATTCAAAATTTTCGGGTTTATTACAGGGACAGCAGAGATCCAATTTGGAAAGGACCAGCAAAGCTTCTCTGGAAAGGTGAAGGGGCAGTAGTACTACAAGACAATAGTGAAATAAAGGTAGTACCAAGAAGGAAAGTAAAGATCATTAGGGATTATGGAAAACAGATGGCAGGTGATGATTGTGTGGCAAGTAGACAGGATGAGGAT

>AF484491

TTTCTAGATGGAATAGATAAGGCTCAAGAAGAGCATGACAAATATCACAGCAATTGGAGAGCAATGGCTAGTGATTTTAATCTGCCACCCATAGTAGCAAAAGAAATAGTAGCCAGCTGTGATAAATGTCAGCTAAAAGGGGAAGCCATGCATGGACAAGTAGACTGCAGTCCAGGGATGTGGCAATTAGATTGCACACATCTAGAAGGAAAAGTAATTATGGTAGCAGTACATGTAGCCAGTGGCTATATAGAAGCAGAAGTTATCCCAGCAGAAACAGGACAGGAGACAGCATATTTTCTTCTAAAATTAGCAGGAAGATGGCCAGTAAAAGTAGTACACACAGACAATGGCAGCAATTTCACCAGCGCTGCATTTAAAGCAGCCTGTTGGTGGGCAAATGTCCAACAGGAATTTGGGATTCCCTACAATCCCCAAAGTCAAGGAGTAGTGGAGTCTATGAATAAAGAATTAAAAAAGATCATAGGGCAGGTAAGAGAGCAAGCTGAACACCTTAAGACAGCAGTACAAATGGCAGTATTCATTCACAATTTTAAAAGAAAAGGGGGGATTGGGGGGTACAGTGCAGGGGAAAGAATAATAGACATAATAGCAACAGACATACAAACTAAAGAATTACAAAAACAAATCACAAAAATTCAAAAATTTCGGGTTTATTACAGGGACAGCAGAGATCCAATTTGGAAAGGACCAGCAAAACTACTCTGGAAAGGTGAAGGGGCAGTGGTGATACAGGACAATAGTGATATAAAGGTAGTACCAAGAAGAAAAGCAAAGATCATTAAAGATTATGGAAAACAGATGGCAGGTGATGATTGTGTGGCAGGTAGACAGGATGAGGAT

>AF484490

TTCTTGGATGGAATAGATAAGGCTCAAGAAGAACATGAGAGATACCACAACAATTGGAGAGCAATGGCTAGTGATTTTAGCCTACCACCTGTGGTGGCAAAAGAAATAGTAGCTAGCTGTGATAAGTGTCAGCTAAAAGGAGAAGCCTTGCATGGACAAGTAGACTGTAGTCCAGGAATATGGCAATTAGATTGTACACATATAGAAGGAAAAGTTATCCTGGTAGCAGTCCATGTGGCCAGTGGCTATATAGAAGCAGAAGTTATTCCAGCAGAAACAGGGCAGGAAACAGCCTACTTTCTCTTAAAATTAGCAGGAAGATGGCCAGTAAAAGTAGTACATACAGACAATGGCAGCAATTTCACCAGCACTGCAGTTAAGGCCGCCTGTTGGTGGGCAGGCATCAAGCAGGAATTTGGAATTCCCTACAATCCCCAAAGTCAAGGAGTAGTAGAATCTATGAATAAAGAATTAAAGAAAATTATAGGACAGGTAAGAGATCAAGCTGAACATCTTAGGACAGCAGTACAAATGGCAGTATTCATCCACAATTTTAAAAGAAAAGGGGGGATTGGGGGGTACAGTGCAGGGGAAAGAATAATAGACATAATAGCAACAGATATACAAACTAAAGAATTACAAAAACAAATCACAAAAATTCAAAATTTTCGGGTTTATTACAGGGACAGCAGAGATCCAATTTGGAAAGGACCAGCAAAACTTCTCTGGAAAGGTGAAGGGGCAGTAGTAATACAAGACAATAGTGAAATAAAAGTAGTACCAAGAAGAAAAGTAAAGATCATTAGGGATTATGGAAAACAGATGGCAGGTGATGATTGTGTGGCAAGTAGACAGGATGAGGAT

>AF484489

TTTTTGGATGGAATAGATAAGGCTCAAGAAGAACATGAGAAATACCACAACAATTGGAGAGCAATGGCTAGTGATTTTAACCTGCCACCTGTGGTAGCAAAAGAAATAGTAGCTAGTTGTGATAAATGTCAGATAAAAGGAGAAGCCTTGCATGGACAAGTAGACTGTAGTCCAGGAATATGGCAATTAGATTGTACACATTTAGAAGGAAAAGTCATCCTGGTAGCAGTCCATGTAGCCAGTGGCTATATAGAAGCAGAAGTTATTCCAGCAGAAACAGGGCAGGAAACAGCCTACTTTCTCTTGAAATTAGCAGCAAGATGGCCAGTAAAAGTAGTACATACAGACAATGGCAGCAATTTCATCAGCGCTGCAGTTAAGGCCGCCTGTTGGTGGGCAGGCATCAAGCAGGAATTTGGAATTCCCTACAATCCCCAAAGTCAAGGAGTAGTAGAATCTATGAATAAAGAATTAAAGAAAATTATTGGACAGGTAAGAGAGCAAGCTGAACATCTTAGGACAGCAGTACAAATGGCAGTATTCATTCACAATTTTAAAAGAAAAGGGGGGATTGGGGAGTACAGTGCAGGGGAAAGAATAATAGACATAATAGCAACAGACATACAAACTAAAGAATTACAAAAACAAATTATAAAAATTCAAAATTTTCGGGTTTATTACAGGGACAGCAGAGATCCAATTTGGAAAGGACCAGCAAAGCTTCTTTGGAAAGGTGAAGGGGCAGTAGTAATACAAGATAATAGTGAAATAAAGGTAGTACCAAGAAGAAAAGCAAAAATCATTAGGGATTATGGAAAACAGATGGCAGGTGATGATTGTGTGGCAAGTAGACAGGATGAGGAT

>AF484488

TTTTTAGATGGAATAGATAAGGCTCAGGAAGAACATGAAAAATACCACAATAATTGGAGAGCAATGGCTAGTGATTTTAACCTGCCACCTGTAGTAGCAAAAGAAATAGTAGCTAGCTGTGATAAATGTCAGCTAAAAGGAGAAGCCTTGCATGGACAAGTAGACTGCAGTCCAGGAATATGGCAATTAGATTGTACACATTTAGAAGGAAAAGTTATCCTGGTAGCAGTCCATGTAGCCAGTGGCTATATAGAAGCAGAAGTGATTCCAGCAGAAACAGGGCAGGAAACAGCCTACTTTATCTTGAAATTAGCAGGAAGATGGCCAGTAAAAATAGTACATACAGACAATGGCAGCAATTTCACCAGCGCTGCAGTTAAGGCCGCCTGCTGGTGGGCAGGCATCAAGCAGGAATTTGGAATTCCCTACAATCCCCAAAGTCAAGGAGTAGTAGAATCTATGAATAAAGAATTAAAAAAGATTATAGGACAGGTAAGAGATCAAGCTGAACATCTTAAGACAGCAGTACAAATGGCAGTATTCATCCACAATTTTAAAAGAAAAGGGGGGATTGGGGGGTACAGTGCAGGGGAAAGAATAATAGACATAATATCAACAGACATACAAACTAAAGAACTACAAAAACAAATTACAAAAATTCACAATTTTCGGGTTTATTACAGGGACAGCAGAGATCCAATTTGGAAAGGACCAGCAAAGCTCCTCTGGAAAGGTGAAGGGGCAGTAGTGATACAAGACAATAGTGAAATAAAAGTAGTACCAAGAAGAAAAGCAAAGATCATTAGGGATTATGGAAAACAGATGGCAGGTGATGATTGTGTGGCAGGTAGACAGGATGAGGAT

>AF484487

TTCTTGGATGGAATAGATAAGGCTCAAGAAGAACATGAGAAATACCACACCAATTGGAGAGCCATGGCTAGTGACTTTAACCTGCCACCTGTGGTAGCAAAGGAAATAGTAGCTAGCTGTGATAAATGTCAGCTAAAAGGAGAAGCCTTGCATGGACAAGTAGACTGTAGTCCAGGAATATGGCAATTAGATTGTACACATTTAGAAGGAAAAGTTATCCTGGTAGCAGTCCATGTGGCCAGTGGTTATATAGAAGCAGAAGTTATCCCAGCAGAAACAGGGCAGGAAACAGCCTACTTTATCTTGAAATTAGCAGGAAGATGGCCAGTAAAAGTAGTACATACAGACAATGGCAGTAATTTCACCAGCGCTGCAGTTAAGGCCGCCTGTTGGTGGGCAGGCATCAAGCAGGAATTTGGAATTCCCTACAATCCCCAAAGTCAAGGAGTAGTAGAGTCTATGAATAAAGAATTAAAGAAAATTATAGGACAGGTAAGAGAGCAAGCTGAACATCTTAAGACAGCAGTACAAATGGCAGTATTCATCCACAATTTTAAAAGAAAAGGGGGGATTGGGGGGTACAGTGCAGGGGAAAGAATAATAGACATAATAGCAACAGACATACAAACTAAAGAATTACAAAAACAAATCATAAAAATTCAAAATTTTCGGGTTTATTACAGGGACAGCAGAGATCCAATTTGGAAAGGACCAGCAAAGCTTCTCTGGAAAGGTGAAGGGGCAGTAGTAATACAAGACAATAGTGAAATAAAGGTAGTACCAAGAAGGAAAGTAAAGATCATTAGAGATTATGGAAAACAGATGGCAGGTGATGATTGTGTGGCAAGTAGACAGGATGAGGAT

>AF484486

TTTTTGGATGGAATAGATAAGGCGCAAGAAGAACATGAGAAATACCACAACAATTGGAGAGCAATGGCTAGTGATTTTAACCTACCACCTGTGGTAGCAAAAGAGATAGTAGCTAGCTGTGATAAATGTCAGCTAAAAGGAGAAGCCTTGCATGGACAAGTAGACTGTAGTCCAGGAATATGGCAGTTAGATTGTACACATTTAGAAGGAAAAGTTATCTTGGTAGCAGTCCATGTAGCCAGTGGCTATATAGAAGCAGAAGTTATTCCAGCAGAAACAGGGCAGGAAACAGCCTACTTTCTCTTAAAATTAGCAGGAAGATGGCCAGTAAAAGTAGTACATACAGACAATGGCAGCAATTTCACCAGCGCTGTAGTTAAGGCCGCCTGTTGGTGGGCAGGCATCAAACAGGAATTTGGAATTCCCTACAATCCCCAAAGTCAAGGAGTAGTAGAATCAATGAATAAAGAATTGAAGAAAATTATCGGACAGGTAAGAGATCAAGCTGAACATCTTAAAACAGCAGTACAAATGGCAGTATTCATCCACAATTTTAAAAGAAAAGGGGGGATTGGGGGGTACAGTGCAGGGGAAAGAATAATAGACATAATAGCAACAGACATACAAACTAAAGAATTACAAAAACAAATCATAAAAATTCAAAATTTTCGGGTTTATTACAGGGACAGCAGAGATCCAATTTGGAAAGGACCAGCAAAGCTTCTCTGGAAAGGTGAAGGGGCAGTAGTAATACAAGACAATAGTGAAATAAAGGTAGTCCCAAGAAGAAAAGTAAAGATCATTAGGGATTATGGAAAACAAATGGCAGGTGATGATTGTGTGGCAAGTAGACAGGATGAGGAT

>AF484485

TTTCTGGATGGAATAGATAAGGCTCAAGAAAAACATGAGAAATACCACAGCAACTGGAGAGCAATGGCTAGTGATTTCAACCTACCACCTGTGGTAGCAAAAGAAATAATAGCTAGCTGTGATAAATGTCAGCTAAAAGGAGAAGCCTTGCATGGACAAGTAGATTGTAGTCCAGGAATATGGCAATTAGACTGTACCCATTTAGAAGGAAAAGTTATCCTAGTAGCAGTCCATGTAGCCAGTGGCTACATAGAAGCAGAAGTTATTCCAGCAGAAACAGGGCAGGAAACAGCCTACTTCCTCTTAAAATTAGCAGGAAGATGGCCAGTAAAAGTAGTACATACAGACAATGGCAGCAACTTCACCAGCGCTGCAGTTAAGGCCGCCTGTTGGTGGGCAGGCATCAAGCAGGAATTTGGAATTCCCTATAATCCCCAAAGTCAAGGAGTAGTAGAATCTATGAATAAAGAATTAAAGAAAATTATAGGACAGGTAAGAGATCAAGCTGAACATCTTAAGACAGCAGTACAAATGGCAGTATTCATCCACAATTTTAAAAGAAAAGGGGGGATTGGGAGGTACAGTGCAGGGGAAAGAATAATAGACATAATAGCAACAGACATACAAACTAAAGAATTACAAAAACAAATCACAAAAATTCAAAATTTTCGGGTTTATTACAGGGACAGCAGAGATCCACTTTGGAAAGGACCAGCAAAGCTTCTCTGGAAAGGTGAAGGGGCAGTAGTAATACAAGACAATAGTGAAATAAAAGTAGTACCAAGAAGAAAAGCAAAGATTATTAGGGATTATGGAAAACAGATGGCAGGTGATGATTGTGTGGCAAGTAGACAGGATGAGGAT

>AF484483

TTCCTGGATGGAATAGATAAGGCTCAAGAAGAACATGAGAAATACCACAACAATTGGAGAGCAATGGCTAGTGATTTTAACCTGCCACCTGTGGTAGCAAAAGAAATAGTAGCTAGCTGTGATAAATGTCAGCTAAAAGGGGAAGCCTTGCATGGACAAGTAGACTGTAGTCCAGGAATATGGCAATTAGATTGTACACATTTAGAAGGAAAAGTTATCCTGGTAGCAGTCCATGTCGCCAGTGGCTACATAGAAGCAGAAGTTATTCCAGCGGAAACAGGACAGGAAACAGCCTACTTTCTTTTGAAACTGGCAGGAAGATGGCCAGTAAAAGTAGTACATACAGACAATGGCAGCAATTTCACTAGCGCTGCAGTTAAGGCTGCCTGTTGGTGGGCAGGCATCAAGCAGGAATTTGGAATTCCCTACAATCCCCAAAGTCAAGGAGTAGTAGAATCTATGAATAAAGAATTAAAAAAGATTATAGGACAGGTAAGAGATCAAGCTGAACATCTTAAGACAGCAGTACAAATGGCAGTCTTCATTCACAATTTTAAAAGAAGAGGGGGGATTGGGGGGTACAGTGCAGGGGAAAGAATAATAGACATAATAGCAACAGACATACAAACTAAAGAATTACAAAAACAAATCATAAAAATTCAAAATTTTCGGGTTTATTACAGGGACAACAGAGATCCAATTTGGAAAGGACCAGCAAAGCTTCTCTGGAAAGGTGAAGGGGCAGTAGTAATACAAGACAATAGTGACATAAAGGTAGTACCAAGAAGAAAAGTAAAAATTATTAGGGATTATGGAAAACAGATGGCAGGTGATGATTGTGTGGCAAGTAGACAGGATGAGGAT

>AF484482

TTTCTAGATGGGATAGATAAGGCTCAAGAAGAACATGAAAAATATCACAGCAATTGGAGAGCAATGGCTAGTGATTTTAACCTGCCACCTATAGTAGCAAAGGAAATAGTAGCCAGCTGTGATAAATGTCAACTAAAAGGGGAAGCTATGCATGGACAAGTAGACTGTAGTCCAGGGATATGGCAATTAGATTGCACACATCTAGAAGGAAAAGTAATCCTGGTAGCAGTCCATGTAGCCAGTGGCTATATAGAAGCAGAAGTTATTCCAGCAGAAACAGGACAGGAGACAGCATATTTCCTGTTAAAATTAGCAGGAAGATGGCCAGTAAAAGTAGTACACACAGACAATGGCAGCAATTTCACCAGCGCTGCATTTAAAGCAGCCTGTTGGTGGGCAAGTATCCAGCAGGAATTTGGAATTCCCTACAATCCCCAAAGTCAAGGAGTAGTGGAATCTATGAATAAGGAATTAAAGAAAATCATAGGGCAGGTAAGAGAGCAAGCTGAACATTTGAGAACAGCAGTACAAATGGCAGTATTCATTCACAATTTTAAAAGAAAAGGGGGGATTGGGGGGTACAGTGCAGGGGAAAGAATAATAGACATAATAGCAACAGACATACAAACTAGAGAATTACAAAAACAAATTATAAAAATCCAAAATTTTCGGGTTTATTACAGGGACAGCAGAGATCCAATTTGGAAAGGACCAGCAAAACTACTCTGGAAAGGTGAAGGAGCAGTAGTAATACAGGACAATAGTGATATAAAGGTAGTGCCAAGAAGAAAAGTAAAAATCATTAGGGATTATGGAAAACAGATGGCAGGTGATGATTGTGTGGCAGGTAGACAGGATGAG

>AF484481

TTCTTGGATGGAATAGATAAGGCTCAAGAAGAACATGAGAAATACCACAGCAATTGGAGAGCAATGGCTAGTGATTTTAACCTGCCACCTGTGGTAGCAAAAGAAATAGTAGCTAGCTGTGATAAATGTCAGCTAAAAGGAGAAGCCTTGCATGGACAAGTAGACTGCAGTCCAGGAATATGGCAATTAGATTGTACACATTTAGAAGGAAAAGTTATCCTGGTCGCAGTCCATGTAGCCAGTGGCTATATAGAAGCAGAAGTTATTCCAGCAGAAACAGGGCAGGAAACAGCCTACTTTCTCTTGAAATTAGCAGGAAGATGGCCAGTAAAAGCAGTACATACAGACAATGGCAGCAATTTCACTAGCGCTGCAGTTAAGGCCGCCTGTTGGTGGGCAGGCATCAAGCAGGAATTTGGAATTCCCTACAATCCCCAAAGTCAAGGAGTAGTAGAATCTATGAATAAAGAATTAAAGAAAATCATAGGACAGGTAAGAGATCAAGCTGAATATCTTAAGACAGCAGTACAAATGGCAGTATTCATCCACAATTTTAAAAGAAAAGGGGGGATTGGGGGATACAGTGCAGGGGAAAGAATAATAGACATAATAGCAACAGACATACAAACTAAAGAATTACAAAAACAAATCACAAAAATTCAAAATTTTCGGGTTTATTACAGGGACAGCAGAGATCCAATTTGGAAAGGACCAGCAAAACTTCTCTGGAAAGGTGAAGGGGCAGTAGTGATACAAGACAATAGTGAAATAAAGGTAGTACCAAGAAGAAAAGCAAAGATCATTAGGGATTATGGAAAACAGATGGCAGGTGATGATTGTGTGGCAAGTAGACAGGATGAGGAT

>AF484480

TTCTTGGATGGAATAGATAAGGCTCAAGAAGAACATGAGAAATACCACAACAATTGGAGAGCAATGGCTAGTGATTTTAACCTGCCACCTGTGGTGGCAAAAGAAATAGTAGCTAGCTGTGATAAATGTCAGCTAAAAGGAGAAGCCTTGCATGGACAAGTAGACTGTAGTCCAGGAATATGGCAATTAGATTGTACACATTTAGAAGGAAAAGTTATCCTGGTAGCAGTCCATGTAGCCAGTGGCTATATAGAAGCAGAAGTTATCCCAGCAGAAACAGGGCAGGAAACAGCCTACTTTCTCTTGAAATTGGCAGGAAGATGGCCAGTAAAAACAGTACATACAGACAATGGCAGCAATTTCACCAGCGCTGCGGTTAAGGCCGCCTGTTGGTGGGCAGGCATCAAACAGGAATTTGGAATTCCCTACAATCCCCAAAGTCAAGGAGTAGTAGAATCTATGAATAAAGAATTAAAGAAAATTATAGGACAAGTAAGAGATCAAGCTGAACATCTTAAGACAGCAGTACAAATGGCAGTATTCATTCACAATTTTAAAAGAAAAGGGGGGATTGGGGGGTACAGTGCAGGGGAAAGAATAATAGACATAATAGCAACAGACATACAAACTAAAGAGTTACAAAAACAAATCATAAAAATTCAAAATTTTCGGGTTTATTACAGGGACAGCAGAGATCCAATTTGGAAAGGACCAGCAAAGCTTCTCTGGAAAGGTGAAGGGGCAGTAGTAATACAAGACAATAGTGAAATAAAGGTAGTACCAAGAAGAAAAGTAAAGATCATTAGGGATTATGGAAAACAGATGGCAGGTGATGATTGTGTGGCAAGTAGACAGGATGAGGAT

>AF484479

TTTTTAGATGGAATAGATAAAGCCCAAGAAGAACATGAAAGATATCACAGCAATTGGAGAACAATGGCTAGTGATTTTAATCTGCCACCTATAGTAGCAAAAGAAATAGTAGCCAGTTGTGATAAATGTCAGCTAAAAGGGGAAGCCATGCATGGACAAGTAGACTGCAGTCCAGGGATATGGCAATTAGATTGCACACATCTAGAAGGAAAAGTAATTCTGGTAGCAGTCCATGTAGCCAGTGGCTACTTAGAAGCAGAAGTTATCCCAGCAGAAACAGGACAGGAGACAGCATACTTTCTACTAAAATTAGCAGGAAGATGGCCAGTAAAAGTAGTACACACAGACAATGGCAGCAATTTCACCAGCGCTGCATTTAAAGCAGCCTGTTGGTGGGCAAATGTCCAACAAGAATTTGGGATTCCCTACAATCCCCAGAGTCAAGGAGTAGTAGAATCTATGAATAAAGAATTGAAGAAAATTATAGGACAGGTAAGAGATCAAGCTGAACATCTTAAGACAGCAGTACAAATGGCAGTATTCATCCACAATTTTAAAAGAAAAGGGGGGATTGGGGGGTACAGTGCAGGGGAAAGAATAATAGACATAATAGCAACAGACATACAAACTAAAGAATTACAAAAACAAATCATAAAAATTCAAAATTTTCGGGTTTATTACAGGGACAGCAGAGATCCAGTTTGGAAAGGACCAGCAAAGCTTCTCTGGAAAGGTGAAGGGGCAGTAGTAATACAAGACAATAGTGAAATAAAGGTAGTACCAAGAAGAAAAGCAAAGATCATTAGGGATTATGGAAAACAGATGGCAGGTGATGATTGTGTGGCAAGTAGACAGGATGAGGAT

>AF484478

TTTTTAGATGGGATAGATAAGGCTCAAGAAGAACATGAAAGATATCACAGCAATTGGAGAACAATGGCTAGTGATTTTAATCTGCCACCTATAGTAGCAAAGGAAATAGTAGCCAGCTGTGATAAATGTCAACTAAAAGGGGAAGCCATGCATGGACAAGTAGACTGTAGCCCAGGGATGTGGCAATTAGATTGCACACATCTAGAAGGAAAAGTGATTCTGGTAGCAGTCCATGTAGCCAGTGGCTATATAGAAGCAGAAGTTATCCCAGCAGAAACAGGACAGGAGACAGCATACTTTCTACTAAAATTAGCAGGAAGGTGGCCAGTAAAAGTAGTACACACAGACAATGGCCCCAATTTCACCAGCGCTGCATTTAAAGCAGCCTGTTGGTGGGCAAATGTCCAACAGGAATTTGGGATTCCCTACAATCCCCAAAGTCAAGGAGTAGTGGAATCTATGAATAAGGAATTAAAGAAAATCATAGGGCAGGTAAGAGAGCAAGCTGAACACCTTAAGACAGCAGTACAAATGGCAGTATTCATTCACAATTTTAAAAGAAAAGGGGGGATTGGGGGATACAGTGCAGGGGAAAGAATAATAGACATAATAGCAACAGACATACAAACTAGAGAACTACAAAAACACATTACAAAAATGCAAAATTTTCGGGTTTATTTCAGGGACAGCAGAGATCCACTTTGGAAAGGACCAGCAAAACTACTCTGGAAAGGTGAAGGGGCAGTAGTAATACAGGATAATGGTGATATAAAGGTAGTGCCCAGAAGAAAAGCAAAGATCATTAGGGATTATGGAAAACAGATGGCAGGTGATGATTGTGTGGCAGGTAGACAGGATGAGGAT

>AF484477

TTCTTGGATGGAATAGATAAGGCTCAAGAGGAACATGAAAAATACCACAACAATTGGAGAGCAATGGCTAGTGATTTTAACCTGCCACCGGTGGTAGCAAAAGAAATAGTAGCTAGCTGTGATAAATGCCAGCTAAAAGGAGAAGCCTTGCATGGACAAGTAGACTGTAGTCCAGGGATATGGCAATTAGATTGTACACATTTAGAAGGAAAAGTTATCCTGGTAGCAGTCCATGTAGCCAGTGGCTATATAGAAGCAGAGGTTATTCCAGCAGAAACAGGACAKGAAACAGCCTACTTTCTCTTGAAACTAGCAGGAAGGTGGCCGGTAAAAGTAGTACATACCGACAATGGCAGCAACTTCACCAGCGCTGCAGTTAAGGCCGCCTGCTGGTGGGCAGACATCAAGCAGGAATTTGGAATTCCCTACAATCCCCAAAGTCAAGGAGTAGTAGAATCTATGAATAAAGAATTAAAGAAGATTATAGGACAGGTAAGAGATCAAGCTGAACATCTGAAGACAGCAGTACAAATGGCAGTATTCATTCACAATTTTAAAAGAAAAGGGGGGATTGGGGGGTACAGTGCAGGGGAAAGAATAATAGACATAATAGCAACAGATATACAAACTAAAGAATTACAAAAACAAATCACAAAAATTMRAAATTTTCGGGTTTATTACAGGGACAGCAGAGATCCAATTTGGAAAGGACCAGCAAAGCTTCTCTGGAAAGGTGAAGGGGCAGTAGTAATACAAGACAATAGTGAAATAAAAGTAGTACCAAGAAGAAAAGCAAAGATCATTAGGGAYTATGGAAAACAGATGGCAGGTGATGATTGTGTGGCAAGTAGACAGGATGAGGAT

>AJ293865

TTTTTAGATGGTATAGATAAGGCCCAAGAAGATCATGAAAGATATCACAGCAATTGGAGAGCAATGGCTAGTGATTTTAATCTGCCACCTATAGTAGCAAAAGAAATAGTGGCCAGCTGTGATAAATGTCAACTAAAAGGGGAAGCCATGCATGGACAAGTAGACTGTAGTCCAGGAATATGGCAATTAGATTGTACCCATTTAGAAGGAAAAGTTATCCTGGTAGCAGTCCATGTAGCCAGTGGCTATATAGAAGCAGAGGTTATCCCAGCAGGAACTGGACAAGAGACAGCATACTTTCTGCTAGGATTCGCGGGAAGATGGCCAGTGAAAGTATTACACACAGACAATGGCAGCAGTTTCACTAGTGCTGCTATGAAGGCAGCATGTTGGTGGGCAAATGTCACACAAGAATTTGGAATTCCCTACAATCCCCAAAGCCAAGGAGTAGTGGAATCTATGAATAAAGAATTAAAGAAAATTATAGGGCAGGTTAGGGATCAAGCTGAACACCTTAAGACAGCAGTACAGATGGCAGTATTCATTCACAATTTTAAAAGAAAAGGGGGGATTGGGGGGTACAGTGCAGGGGAAAGAATAATAGACATAATAGCATCAGAACTACAAACTAAAGAACTACAAAAACAAATTACAAAAATTCAAAATTTTCGGGTTTATTACAGGGACAGCAGAGACCCCATTTGGAAAGGACCAGCAAAACTACTCTGGAAAGGTGAAGGGGCAGTAGTAATACAGGACAATAGTGATATAAAGGTAGTACCAAGAAGAAAAGCAAAGATTATTAGGGATTATGGAAAACAGATGGCAGGTGATGATTGTGTGGCAGGTAGACAGGATGAGGAT

>AF377954

TTTTTAGATGGCATAGATAAAGCCCAAGAAGATCATGAAAGATATCACAGCAATTGGAGAGCAATGGCTAGTGATTTTAATCTGCCACCTATAGTAGCAAAAGAAATAGTGGCCAGCTGTGATAAATGTCAGTTAAAAGGGGAAGCCATGCATGGACAAGTAGACTGTGGTCCAGGAATATGGCAATTAGATTGTACACATTTAGAAGGAAAAATTATCCTGGTAGCAGTCCATGTAGCCAGTGGCTATATAGAAGCAGAAGTTATCCCAGCAGAATCAGGACAGGAAACAGCATACTTCATATTAAAATTAGCAGGAAGATGGCCAGTGAAAGTAATACACACAGACAATGGCAGCAATTTCACCAGTGCTGCAGTAAAGGCAGCATGTTGGTGGGCAAATGTCACACAAGAATTTGGAATTCCCTACAATCCCCAAAGCCAAGGAGTAGTAGAATCTATGAATAAAGAATTAAAGAAAATTATAGGGCAGGTCAGGGATCAAGCTGAACACCTTAAGACAGCAGTACAGATGGCAGTATTCATTCACAATTTTAAAAGAAAAGGGGGGATTGGGGGGTACAGTGCAGGGGAAAGAATAATAGACATAATAGCATCAGATATACAAACTAAAGAACTACAAAAACAGATTACAAAAATTCAAAATTTTCGGGTTTATTACAGGGACAGCAGAGACCCCATTTGGAAAGGACCAGCAAAACTACTTTGGAAAGGTGAAGGGGCAGTAGTAATACAGGACAATAGTGATATAAAGATAGTACCAAGGAGAAAAGCAAAAATCATTAAGGATTATGGAAAACAGATGGCAGGTGATGATTGTGTGGCAGGTAGACAGGATGAGGAT

>AF377955

TTTTTAGATGGCATAGATAAAGCCCAAGAAGAGCATGAAAGATATCACAGCAATTGGAGAGCAATGGCTAGTGATTTTAATCTGCCACCTATAGTAGCAAAAGAAATAGTGGCCAGCTGTGATAAATGTCAGTTAAAAGGGGAAGCCATGCATGGACAAGTAGACTGTGGTCCAGGAATATGGCAATTAGATTGTACACACTTAGAAGGAAAAATTATCCTGGTAGCAGTCCATGTAGCCAGTGGCTATATAGAAGCAGAAGTTATCCCAGCAGAAACAGGACAGGAGACAGCATACTTTATATTAAAATTAGCAGGAAGGTGGCCAGTAAAAGTAATACACACAGACAATGGCAGCAATTTCACCAGTGCTGCAGTAAAGGCAGCATGTTGGTGGGCAAATGTCACACAAGAATTTGGAATTCCCTACAATCCCCAAAGCCAAGGAGTAGTGGAATCTATGAATAAAGAATTAAAGAAAATTATAGGGCAGGTCAGGGATCAAGCTGAACACCTTAAGACAGCAGTACAGATGGCAGTATTCATTCACAATTTTAAAAGAAAAGGGGGGATTGGGGGGTACAGTGCAGGGGAAAGAATAATAGACATAATAGCATCAGATATACAAACTAAAGAACTACAAAAACAGATTATAAAAATTCAAAATTTTCGGGTTTATTACAGGGACAGCAGAGACCCCATTTGGAAAGGACCAGCAAAACTACTTTGGAAAGGTGAAGGAGCAGTAGTAATACAGGACAATAGTGATATAAAAGTAGTACCAAGAAGAAAAGTAAAAATCATTAAGGATTATGGAAAACAGATGGCAGGTGATGATTGTGTGGCAAGTAGACAGGATGAGAAT

>AF377956

TTTTTGGATGGGATAGATAAGGCTCAAGAAGAACATGAAAAATATCACAGCAATTGGAGAGCAATGGCTAGTGATTTTAATTTGCCACCTGTAGTAGCCAAAGAAATAGTAGCCAGCTGTGATAAATGTCAGCTAAAAGGGGAAGCCATGCATGGACAAGTAGACTGCAGTCCAGGGATATGGCAACTAGATTGTACACATTTAGAAGGAAAAATTATCTTGGTAGCAGTCCATGTGGCTAGTGGTTATATAGAAGCAGAAGTTATCCCAGCAGAAACGGGACAGGAAACAGCCTACTTCCTACTAAAGTTAGCAGGAAGATGGCCAGTAAAAATAATACATACAGATAATGGCAGCAATTTCACCAGTACTGTGGTTAAGGCAGCCTGTTGGTGGGCAGGTATCCAGCAGGAATTTGGAATTCCCTACAATCCTCAAAGTCAAGGAGTAGTAGAATCTATGAATAAAGAATTAAAGAAAATTATAGGACAGGTAAGAGATCAAGCTGAACATCTTAAGACAGCAGTACAAATGGCAGTATTCATTCACAATTTTAAAAGAAAAGGGGGGATTGGGGGGTACAGTGCAGGGGAAAGAATAATAGACATAATAGCAACAGACATACAAACTAAAGAATTACAAAAACAAATTACAAAAATTCAAAACTTTCGGGTTTATTTCAGGGACAGCAGAGACCCAATTTGGAAAGGACCAGCAAAGCTACTCTGGAAAGGTGAAGGGGCAGTAGTCATACAAGACAATAATGAAATAAAAGTAGTACCAAGAAGAAAAGCAAAAATCATTAGGGATTATGGAAAACAGATGGCAGGTGATGATTGTGTGGCAGGTAGACAGGATGAGGAT

>AF377957

TTTTTAGATGGGATAGATAAGGCTCAAGAAGATCATGAAAGATATCACACCAATTGGAGAGCAATGGTTAATGATTTTAATCTGCCACCTATAGTAGCAAAAGAAATAGTGGCCAGCTGTGATAAATGTCAGCTAAAAGGGGAGGCTATGCATGGACAAGTAGACTGTAGTCCAGGAATATGGCAATTAGATTGCACACATCTAGAAGGAAAAGTAATCCTGGTAGCAGTCCATGTGGCCAGTGGCTATATAGAAGCAGAAGTTATCCCAGCAGAAACAGGACAAGAGACAGCATACTTTCTGCTAAAATTAGCAGGAAGATGGCCAGTAAAGGTAGTACACACAGACAATGGTAGCAATTTCACCAGCGCTGCAGTTAAAGCAGCCTGTTGGTGGGCAAATGTCAAGCAGGAATTTGGAATTCCCTACAATCCCCAAAGTCAAGGAGTAGTGGAATCTATGAATAAAGAATTAAAGAAAATCATAGGACAGGTCAGGGAGCAAGCTGAACACCTTAAGACAGCAGTACAAATGGCAGTATTCATTCACAATTTTAAAAGAAAAGGGGGGATTGGGGGGTACAGTGCAGGGGAAAGAATAATAGATATAATAGCATCAGATATACAAACCAAAGAACTACAGAAACAAATTACAAAAATTCAAAATTTTCGGGTTTATTACAGGGACAGCAGAGACCCCATTTGGAAAGGACCAGCAAAACTACTCTGGAAAGGTGAAGGGGCAGTAGTAATACAAGACAATAGTGACATAAAAGTAGTACCAAGAAGGAAAGCAAAGATCATCAGGGATTATGGAAAACAGATGGCAGGTGATGATTGTGTGGCAGGTAGACAGGATGAGGAT

>AF377958

TTTTTGGATGGGATAGATAAGGCCCAAGAAGAACATGAGAGATATCACAGCAATTGGAGAGCAATGGCTAGTGAATTTAATTTGCCACCTATAGTAGCAAAAGAAATAGTGGCCAGCTGTGATAAATGTCAGCTAAAAGGGGAAGCCATGCATGGACAAGTAGACTGTAGTCCAGGAATATGGCAATTAGATTGTACACATTTAGAAGGAAAAATTATCCTGGTAGCAGTCCATGTAGCCAGTGGCTATATAGAAGCAGAAGTTATCCCAGAAGAAACAGGACAGGAGACAGCATACTTTATATTAAAATTAGCAGGAAGATGGCCAGTGAAAGTAATACACACAGACAATGGCAGAAATTTCACCAGTAATGCAGTAAAGGCAGCATGTTGGTGGGCAAATGTTACACAAGAATTTGGAATTCCCTACAATCCCCAAAGCCAAGGAGTAGTGGAATCTATGAATAAAGAATTAAAGAAAATTATAGGGCAGGTCAGGGATCAAGCTGAACACCTTAAGACAGCAGTACAGATGGCAGTATTCATTCACAATTTTAAAAGAAAAGGGGGGATTGGGGGGTACAGTGCAGGGGAAAGAATAATAGACATAATAGCATCAGATATACAAACCAAAGAACTACAAAAACAAATTACAAAAATTCAAAATTTTCGGGTTTATTACAGGGACAGCAGAGACCCCATTTGGAAAGGACCAGCAAAACTACTTTGGAAAGGTGAAGGGGCAGTAGTAATACAGGACAATAGTGATATAAAGGTAGTACCAAGAAGAAAAGCAAAAATCATTAAGGATTATGGAAAACAGATGGCAGGTGATGATTGTGTGGCAGGTAGACAGGATGAGGAT

>AF377959

TTTTTAGATGGAATAGATAAGGCTCAAGAAGAACATGAAAAATATCACAACAATTGGAGAGCAATGGCTAGTGATTTTAATCTGCCACCAGTAGTAGCAAAAGAAATAGTAGCTAACTGTGATAAATGTCAGCTAAAAGGGGAAGCCATGCATGGACAAGTAGACTGTAGTCCAGGGATATGGCAATTAGACTGTACACATTTAGAAGGTAAAATTATCCTGGTAGCAGTCCATGTAGCTAGTGGCTATATAGAAGCAGAAGTTATCCCAGCAGAAACAGGACAGGAAACAGCCTACTTCATATTAAAATTAGCAGGAAGATGGCCAGTAAAAATAATACACACAGACAATGGCAGCAATTTCACCAGTGCTGCAGTAAAGGCAGCATGTTGGTGGGCAAATGTTACACAAGAATTTGGAATTCCCTACAATCCCCAAAGCCAAGGAGTGGTGGAATCCATGAATAAAGAATTAAAGAAGATCATAGGACAGATAAGAGATCAAGCTGAACATCTTAAGACAGCAGTACAAATGGCAGTATTCATTCACAATTTTAAAAGAAAAGGGGGGATTGGGGGGTACAGTGCAGGGGAACGAATAGTAGACATAATATCAACAGACATACAAACTAAAGAATTACAAAAACAGATTTTAAAAATTCAAAATTTTCGGGTTTATTACAGAGACAGCAGAGACCCAATTTGGAAAGGACCAGCAAAACTACTCTGGAAAGGTGAAGGGGCAGTAGTAATACAGGACAATAGTGAAATAAAGGTAGTACCAAGAAGAAAAGCAAAGATTATTAGGGATTATGGAAAACAGATGGCAGGTGGTGATTGTATGGCAGGTAGACAGGATGAGGAT

>AY037266

TTTTTAGATGGAATAGATAAGGCCCAAGATGAACATGAGAAATATCACAGTAATTGGAGATCAATGGCTAGTGATTTTAACCTGCCACCTATAATAGCAAAAAAAATAGTAGCCAGCTGTGATAAATGTCAGCTAAAAGGAGAAGCCATGCATGGACAAGTAGACTGTAGTCCAGGAATATGGCAGCTAGATTGTACACACTTAGAAGGAAAAATCATCCTGGTAGCCGTTCATGTAGCCAGTGGATATATAGAAGCAGAAGTTATTCCAGCAGAAACAGGGCAAGAAACAGCATACTTTCTCTTGAAATTAGCAGGAAGATGGCCAGTAAAAACAATACATACAGACAATGGCAGCAATTTCACCAGTAATACGGTTAAAGCCGCCTGTTGGTGGGCAGGGATCAAACAGGAATTCGGCATTCCCTACAATCCCCAAAGTCAAGGAGTAATAGAATCTATGAATAAGGAATTAAAGAAAATTATAGGACAGGTAAGAGATCAGGCTGAACATCTTAAAACAGCAGTACAAATGGCAGTATTTATCCACAATTTTAAAAGAAAAGGGGGGATTGGGGGGTACAGTGCAGGGGAAAGAATAGTAGACATAATAGCAACAGACATACAAACTAAAGAATTACAAAAACAAATTACAAAAATTCAAAATTTTCGGGTTTATTACAGGGACAGCAGAGATCCACTTTGGAAAGGACCAGCAAAGCTGCTCTGGAAAGGTGAAGGGGCAGTAGTAATACAAGATAATAGTGACATAAAGGTAGTGCCAAGAAGAAAAGCAAAGATCATTAGGGATTATGGAAAACAGATGGCAGGTGATGATTGTGTGGCAGGTAGACAGGATGAGGAT

>AY037267

TTTTTAGATGGAATAGATAAGGCACAGGAGGACCATGAAAAATATCACAACAATTGGAGAGCAATGGCTAGTGATTTTAATCTGCCACCTGTAGTAGCAAAAGAAATAGTAGCTAGCTGTGATAAGTGCCAGCTAAAAGGGGAAGCCATGCATGGACAAGTAGACTGTAGTCCAGGGATATGGCAATTAGATTGTACACATTTAGAAGGAAAAGTTATCCTGGTAGCAGTCCATGTAGCTAGTGGGTACCTAGAAGCAGAAGTTATCCCAGCAGAAACAGGACAAGAAACAGCTTACTTCATACTAAAATTAGCAGGAAGGTGGCCAGTAAAAACAATACATACTGACAATGGCAGCAATTTCACCAGTACCGCGGTTAAGGCCGCCTGTTGGTGGGCAGGGATCCAGCAGGAATTTGGCATTCCCTACAACCCCCAAAGTCAAGGAGTAATAGAATCTATGAATAAAGAATTAAAGAAAATTATAGGACAAATAAGAGATCAGGCTGAACATCTTAAGACAGCAGTACAAATGGCAGTATTCATCCACAATTTTAAAAGAAAAGGGGGGATTGGGGGATACAGTGCAGGGGAAAGAATAATAGACATAATATCAACAGACATACAAACTAGAGAATTACAAAACCAAATTATAAAAATTCAAAATTTTCGGGTTTATTACAGGGACAGCAGAGACCCAGTTTGGAAAGGACCAGCAAAGCTACTCTGGAAAGGTGAAGGGGCAGTAGTCATACAAGACAATAGTGAAATAAAGGTAGTACCAAGAAGAAAAGCAAAAATCATTAGGGATTATGGAAAACAGATGGCAGGTGATGATTGTGTGGCAGGTAGACAGGATGAGGAT

>AY037268

TTCTTAGATGGAATAGATAAGGCCCAAGACGAACATGAAAAATATCACAGTAATTGGAGAGCAATGGCTAGTGATTTTAACCTGCCACCGGTAGTAGCAAAAGAAATAGTAGCCAGCTGTGATAAATGTCAGCTAAAAGGGGAAGCCATGCATGGACAAGTAGACTGTAGTCCAGGAATATGGCAGCTAGATTGTACACATTTAGAAGGAAAAGTTATCTTGGTAGCAGTTCATGTAGCCAGTGGATATATAGAAGCAGAAGTTATTCCAGCAGAAACAGGGCAGGAAACAGCATACTTCCTCTTAAAATTAGCAGGAAGATGGCCAGTAAAAACAATACATACAGACAATGGCAGCAATTTCACCAGTGCTACAGTTAAAGCTGCCTGCTGGTGGGCGGGGATCAAGCAGGAATTTGGTATTCCCTACAATCCCCAAAGTCAAGGGGTAGTAGAATCTATGAATAAAGAATTAAAGAAAATTATAGGACAGGTAAGAGATCAGGCTGAACATCTTAAAACAGCAGTACAAATGGCAGTATTCATTCACAATTTTAAGAGAAAAGGGGGGATTGGGGGATACAGTGCAGGGGAAAGAATAGTAGACATAATAGCAACAGACATACAAACTAAAGAATTACAAAAACAAATTACAAAAATTCAAAATTTTCGGGTTTATTACAGGGACAGCAGAGATCCACTTTGGAAAGGACCAGCAAAGCTTCTCTGGAAAGGTGAAGGGGCAGTAGTAATACAAGATAATAGTGACATAAAAGTAGTGCCAAGAAGAAAAGCAAAAATCATTAGGGACTATGGAAAACAGATGGCAGGTGATGATTGTGTGGCAAGTAGACAGGATGAGGAT

>AY037269

TTTTTAGATGGAATAGACAAGGCCCAAGAAGAACATGAGAAATATCACAGTAATTGGAGAGCAATGGCTAGTGATTTTAACCTGCCACCTGTGGTGGCAAAAGAAATAGTAGCCAGCTGTGATAAATGTCAGCTAAAAGGGGAAGCCATGCATGGACAAGTAGACTGTAGCCCAGGAATATGGCAGCTAGATTGTACACACTTAGAAGGAAAAATTATCCTGGTAGCAGTTCATGTAGCCAGTGGATATATAGAAGCAGAAGTTATTCCAGCAGAGACAGGGCAAGAAACAGCATACTTCATCTTAAAATTAGCAGGAAGATGGCCAGTGAAAACAATACATACAGACAATGGTCCCAATTTCACCAGTACTACTGTTAAGGCCGCCTGTTGGTGGGCGGGGATCAAGCAGGAATTTGGCATTCCCTACAATCCCCAAAGTCAAGGAGTAATAGAGTCTATGAATAAAGAATTAAAGAAAATCATAGGACAGGTAAGAGATCAGGCTGAACATCTTAAGACAGCAGTACAAATGGCAGTATTCATTCACAATTTTAAAAGAAAAGGGGGGATTGGGGGGTACAGTGCAGGGGAAAGAATAATAGACATAATAGCAACAGACATACAAACTAAAGAACTACAAAACCAAATTACAAAAATTCAAAATTTTCGGGTTTATTACAGGGACAGCAGAGATCCACTTTGGAAAGGACCAGCAAAGCTTCTCTGGAAAGGTGAAGGGGCAGTAGTAATACAGGATAATAGTGAAATAAAAGTAGTGCCAAGAAGAAAAGCAAAGATCATTAGGGATTATGGAAAACAGATGGCAGGTGATGATTGTGTGGCAAGTAGACAGGATGAGGAT

>AY037270

TTTCTGGATGGAATAGATAAGGCCCAAGATGAGCATGAGAAATATCACAGTAATTGGAGAGCAATTGCTAGTGATTTTAACCTGCCACCTGTAGTAGCAAAACAAATAGTAGCTAACTGTGATAAATGTCAGGTAAAGGGAGAAGCCATGCATGGACAAGTAGACTGTAGTCCAGGAATATGGCAACTAGATTGTACACATTTAGAAGGAAAAATTATCCTGGTAGCAGTTCATGTAGCCAGTGGATATATAGAAGCAGAAGTTATCCCAGCAGAAACAGGGCAGGAAACAGCATACTTTATCTTAAAATTAGCAGGAAGGTGGCCAGTAAAAACAATACATACAGACAATGGCAGCAATTTCACCAGTAATGCGGTCAAGGCCGCCTGCTGGTGGGCAGGGGTCAAGCAGGAATTTGGCATTCCCTACAATCCCCAAAGTCAAGGAGTAGTAGAATCTATGAATAATGAATTAAAGAAAATTATAGGACAAGTAAGAGATCAGGCTGAACATCTTAAAACAGCAGTACAAATGGCAGTATTCATCCACAATTTTAAAAGAAAAGGGGGGATTGGGGGGTACAGTGCAGGGGAAAGAATAGTAGACATAATAGCAACAGACATACAAACTAAAGAACTACAAAAACAAATTACAAAAATTCAAAATTTTCGGGTTTATTACAGGGACAGCAGAGATCCACTTTGGAAAGGACCAGCAAAGCTCCTCTGGAAAGGTGAAGGGGCAGTAGTAATACAAGATAATAGTGACATAAAAGTAGTGCCAAGGAGAAAAGCAAAGATCATTAGGGATTATGGAAAACAGATGGCAGGTGATGATTGTGTGGCAAGTAGACAGGATGAGGAT

>AY037271

TTTTTAGATGGGATAGATAAGGCACAGGAGGACCATGAAAAATATCACAGCAATTGGAGAGCAATGGCTAGTGATTTTAATCTGCCACCTGTGGTAGCAAAAGAAATAATAGCTAGCTGTGATAAGTGTCAGCTAAAAGGGGAAGCCATGCATGGACAAATAGATTGTAGTCCAGGGATATGGCAATTAGATTGTACACATTTAGAAGGGAAAGTTATCCTGGTAGCAGTCCATGTAGCTAGTGGGTACCTAGAAGCAGAAGTTATCCCAGCAGAAACAGGACAGGAAACAGCCTACTTCATACTAAAGTTAGCAGGAAGATGGCCAGTAAAAACAATACATACAGACAATGGCACCAATTTCACCAGTGCCGCGGTTAAGGCAGCCTGTTGGTGGGCAGGTATCCAGCAGGAATTTGGAATTCCCTACAACCCCCAAAGTCAAGGAGTAGTAGAATCTATGAATAAAGAGCTAAAAAAGATCATAAGACAGGTAAGAGATCAAGCTGAACATCTTAAGACAGCAGTACAAATGGCAGTATTCATCCACAATTTTAAAAGAAAAGGGGGGATTGGGGGATACAGTGCAGGGGAAAGAATAATAGACATAATATCAACAGACATACAAACTAGAGAATTACAAAAACAAATTATAAAAATTCAAAATTTCCGGGTTTATTACAGGGACAGCAGAGACCCAGTGTGGAAAGGACCAGCAAAGCTACTCTGGAAAGGTGAAGGGGCAGTAGTCATACAAGACAATAGTGAAATAAAGGTAGTACCAAGAAGGAAAGCAAAAATCATTAGGGATTATGGAAAACAGATGGCAGGTGATGATTGTGTGGCAGGTAGACAGGATGAGGAT

>AY037272

TTTTTAGATGGAATAGATAAGGCACAGGAGGAACATGAAAAATATCACAACAATTGGAGAGCAATGGCTAGTGATTTTAATCTGCCACCTGTAGTAGCAAAAGAAATAGTAGCTAGCTGTGATAAGTGTCAGCTAAAAGGGGAAGCCATGCATGGACAAGTAGATTGTAGTCCAGGGATATGGCAATTAGATTGTACACATTTAGAAGGAAAAGTTATCCTGGTAGCAGTCCATGTAGCTAGTGGGTACTTAGAAGCAGAAGTTATCCCAGCAGAAACAGGACAAGAAACAGCATACTTCATACTAAAGTTAGCAGGAAGATGGCCAGTAAAAACAATACATACAGACAATGGCACCAATTTCACCAGTGCAGCGGTTAAGGCAGCCTGTTGGTGGGCGGGTATCCAACAGGAATTTGGAATTCCCTACAACCCCCAAAGTCAAGGAGTAGTAGAATATATGAATAAAGAGCTAAAAAAGATCATAGGACAGGTAAGAGATCAAGCTGAACATCTTAAGACAGCAGTACAAATGGCGGTATTCATCCACAATTTTAAAAGAAAAGGGGGGATTGGGGGATACAGTGCAGGGGAAAGAATAATAGACATAATATCAACAGACATACAAACTAGAGAATTACAAAATCAAATTATAAAAATTCAAAATTTCCGGGTTTATTACAGGGACAGCAGAGACCCAGTTTGGAAAGGACCAGCAAAACTACTCTGGAAAGGTGAAGGGGCAGTAGTCATACAAGACAATAGTGAAATAAAGGTAGTACCAAGAAGAAAAGCAAAGATCATTAGGGATTATGGAAAACAGATGGCAGGTGATGATTGTGTGGCAGGTAGACAGGATGAGGAT

>AY037275

TTTTTAGATGGGATAGATAAGGCACAGGAAGAACATGAAAGATATCACAACAATTGGAGAGCAATGGTTAGTGATTTTAACATACCACCTGTAGTAGCAAAAGAAATAGTAGCTAGCTGTGATAAATGTCAGCTAAAAGGGGAAGCCATGCATGGACAAGTAGATTGTAGTCCAGGGATATGGCAATTAGATTGTACACATTTAGAAGGAAAAATTATCCTGGTAGCAGTCCATGTAGCTAGTGGGTACCTAGAAGCAGAAGTCATCCCAGCAGAAACAGGACAAGAAACAGCCTACTTCATACTAAAGTTAGCAGGAAGATGGCCAGTAAAAACAATACATACAGACAATGGCACCAATTTCACCAGTGCCACGGTTAAGGCAGCCTGTTGGTGGGCAGGTATCCAGCAGGAATTTGGGATCCCCTACAACCCCCAAAGTCAGGGAGTAGTAGAATCTATGAATAAAGAGTTAAAGAAGATCATAACACAGGTAAGAGATCAAGCTGAGCATCTTAAGACAGCAGTACAAATGGCAGTATTCATCCACAATTTTAAAAGAAAAGGGGGGATTGGGGGATACAGTGCAGGGGAAAGAATAATAGACATAATATCAACAGACATACAAACTAAAGAATTACAAAAACAAATTACAAAAATTCAAAATTTCCGGGTTTATTACAGGGACAGCAGAGATCCAGTTTGGAAAGGACCAGCAAAGCTACTCTGGAAAGGTGAAGGGGCAGTAGTCATACAAGACAATAGTGAAATAAAGGTAGTACCAAGAAGAAAAGCAAAAATCATTAGGGATTATGGAAAACAGATGGCAGGTGATGATTGTGTGGCAGGTAGACAGGATGAGGAT

>AY037277

TTTTTAGATGGGATAGATAAGGCACAGGAAGAACATGAAAAATATCACAACAATTGGAGAGCAATGGCTAGTGATTTTAATATACCACCTGTAGTAGCAAAAGAAATAGTAGCTAGCTGTGATAAATGTCAGCTAAAAGGGGAAGCCATGCATGGACAAGTAGATTGTAGTCCAGGGATATGGCAATTAGATTGTACACATTTAGAAGGAAAAATTATCCTGGTAGCAGTCCATGTAGCTAGTGGGTACCTAGAAGCAGAAGTTATCCCAGCAGAAACAGGACAAGAAACAGCCTACTTCATACTAAAGTTAGCAGGAAGATGGCCAGTAAAAACAATACATACAGACAATGGCACCAATTTCACCAGTGCCGCGGTTAAGGCAGCCTGTTGGTGGGCAGGTATCCAGCAGGAATTTGGGATCCCCTACAACCCCCAAAGTCAGGGAGTAGTAGAATCTATGAATAAAGAACTAAAGAAGATCATAAAACAGGTGAGAGATCAAGCTGAGCATCTTAAGACAGCAGTACAAATGGCAGTATTCATCCACAATTTTAAAAGAAAAGGGGGGATTGGGGGATACAGTGCAGGGGAAAGAATAATAGACATAATATCATCAGACATACAAACTAAAGAATTACAAAAACAAATTATAAAAATTCAAAATTTCCGGGTTTATTACAGGGACAGCAGAGACCCAGTTTGGAAAGGACCAGCAAAGCTACTCTGGAAAGGTGAAGGGGCAGTAGTCATACAAGACAATAGTGAAATAAAAGTAGTACCAAGAAGAAAAGCAAAAATCATTAGGGATTATGGAAAACAGATGGCAGGTGATGATTGTGTGGCAGGTAGACAGGATGAGGAT

>AY037278

TTTTTAGATGGGATAGATAAGGCACAGGAGGACCATGAAAGATATCACAGTAATTGGAGGGCAATGGCTAATGATTTTAACCTGCCACCTGTAGTAGCAAAAGAAATAGTAGCTAGCTGTGATAAATGTCAGCTAAAAGGAGAAGCCATGCATGGACAAGTAGACTGTAGTCCAGGAATATGGCAACTAGATTGTACACATTTAGAAGGGAAAGTTATCCTGGTAGCAGTTCATGTAGCCAGTGGATATATAGAAGCAGAAGTTATTCCAGCAGAGACAGGGCAGGAAACAGCATACTTTCTCTTAAAATTAGCAGGAAGATGGCCAGTAAAAACAATACATACAGACAATGGCAGCAATTTCACCAGTGCTACAGTTAAGGCCGCCTGTTGGTGGGCGGGGATCAAGCAGGAATTTGGCATTCCCTACAATCCCCAAAGTCAAGGAGTAGTAGAATCTATGAATAAAGAATTAAAGAAAATAATAGGACAAATAAGAGATCAGGCTGAACATCTTAAAACAGCAGTACAAATGGCAGTGTTCATTCACAATTTTAAAAGAAAAGGGGGGATTGGGGGATACAGTGCAGGGGAAAGAATAATAGACATAATAGCAACAGACATACAAACTAAAGAATTACAAAAACAAATTACAAAAATTCAAAATTTTCGGGTTTATTACAGGGACAACAGAGACCCAATTTGGAAAGGACCAGCAAAGCTACTCTGGAAAGGAGAAGGGGCAGTAGTCATACAAGACAATAGTGAAATAAAGGTAGTACCAAGAAGAAAGGCAAAAATCATTAGGGATTATGGAAAACAGATGGCAGGTGATGATTGTGTGGCAGGTAGACAGGATGAGGAT

>AY037279

TTTTTAGATAGGATAGATAAGGCACAAGAGGACCATGAAAAATATCACACCAATTGGAGAGCAATGGTTAGTGATTTTAATCTGCCACCTGTAGTAGCAAAAGAAATAGTAGCTAGCTGTGATAAGTGTCAGCTGAAAGAGGAAGCCATACATGGACAAGTAGATTGTAGTCCAGGGATATGGCAATTAGATTGTACACATTTAGAAGGAAAAATTATCATAGTAGCAGTCCATGTAGCTAGTAGGTACATAGAAGCAGAAGTTATCCCAGCAGAAACAGGACAAGAAACAGCCTACTTCATACTAAAGTTAGCAGGAAGATGGCCAGTAAAAACAATACATACAGACAATGGCCCCAATTTCAGCAGTGCCGCGGTTAAGGCAGCCTGTTGGTGGGCAGGTATCCAACAGGAATTTGGAATTCCCTACAACCCCCAAAGTCAAGGAGTAGTAGAATCTATGAATAAAGAGCTAAAGAAGATCATAGGACAGGTAAGAGATCAAGCTGAATATCTTAAGACAGCAGTACAAATGGCAGTATTCATCCACAATTTTAAAAGAAAAGGGGGGATTGGGGGATACAGTGCAGGGGAAAGAATAATAGACATAATAGCAACAGACATACAAACTAGAGAATTACAAAAACAAATTATAAAAATTCAAAATTTCCGGGTTTATTACAGGGACAGCAGAGACCCAGTTTGGAAAGGACCAGCAAAGCTACTCTGGAAAGGTGAAGGGGCAGTAGTCATACAAGACAATAGTGAAATAAAGGTAGTACCAAGAAGAAAAGCAAAGATCATTAAGGATTATGGAAAACAGATGGCAGGTGATGATTGTGTGGCAGGTAGACAGGATGAGGAT

>AY037280

TTTTTAGATGGGATAGATAAGGCACAGGAGGACCATGAAAAATATCACAGCAATTGGAGAGCAATGGCTAATGATTTTAATCTGCCACCTGTAGTAGCAAAAGAAATAGTAGCTAGCTGTGATAAGTGTCAGCTTAAAGGGGAAGCCATGCATGGACAAGTAGATTGTAGTCCAGGGATATGGCAATTAGATTGTACACATTTAGAAGGAAAAGTTATCCTGGTAGCAGTCCATGTAGCTAGTGGGTACCTAGAAGCAGAAGTTATCCCAGCAGAAACAGGACAAGAAACAGCCTACTTCATACTAAAGTTAGCAGGAAGATGGCCAGTAAAAACAATACATACAGACAATGGCACCAATTTCACCAGTACCGCGGTTAAGGCAGCCTGTTGGTGGGCAGGTATCCAGCAGGAATTTGGGATTCCCTACAACCCCCAAAGTCAAGGAGTAGTAGAGTCTATGAATAAAGAGCTAAAGAAGATCATAAGACAAGTAAGAGATCAAGCTGAACATCTTAAGACAGCAGTACAAATGGCAGTATTCATCCACAATTTTAAAAGAAAAGGGGGGATTGGGGGATACAGTGCAGGGGAAAGAATAATAGACATAGTATCAACAGACATACAAACTAGAGAATTACAAAAACAAATTATAAAAATTCAAAATTTCCGGGTTTATTACAGGGACAGCAGAGACCCAGTTTGGAAAGGACCAGCAAAACTACTCTGGAAAGGTGAAGGGGCAGTAGTCATACAAGACAATAGTGAAATAAAGGTAGTACCAAGAAGAAAAGCAAAGATCATTAGGGATTATGGAAAACAGATGGCAGGTGATGATTGTGTGGCAGGTAGACAGGATGAGGAT

>AY037281

TTTTTAGATGGGATAGATAAGGCACAGGAGGAACATGAAAAGTATCACAACAATTGGAGAGCAATGGCTAGTGATTTTAATCTGCCACCTGTAGTAGCAAAAGAAATAGTAGCTAGCTGTGATAAGTGTCAGCTGAAAGGGGAAGCCATGCATGGACAAATAGATTGTAGTCCAGGGATATGGCAATTAGATTGCACACATTTAGAAGGAAAAATTATCCTGGTAGCAGTCCATGTAGCTAGTGGGTACCTAGAAGCAGAGGTTATCCCAGCAGAAACAGGACAAGAAACAGCCTACTTCATACTAAAGTTAGCAGGAAGATGGCCAGTAAAAACAATACATACAGACAATGGCCCCAATTTCAGCAGTAACGCGGTTAAGGCAGCCTGTTGGTGGGCAGGTATCCAGCAGGAATTTGGAATTCCCTACAACCCCCAAAGTCAAGGAGTAGTAGAATCTATGAATAAAGAGTTAAAGAAGATCATAAGGCAGATAAGAGATCAAGCTGAACATCTTAAGACAGCAGTACAAATGGCAGTATTCATTCACAATTTTAAAAGAAAAGGGGGGATTGGGGGATACAGTGCAGGGGAAAGAATAATAGACATAATATCAACAGACATACAAACTAGAGAATTACAAAAACAAATTATAAAAATTCAAAATTTCCGGGTCTATTACAGGGACAGCAGAGACCCAGTTTGGAAAGGACCAGCAAAGCTACTCTGGAAAGGTGAAGGGGCAGTAGTCATACAAGACAATAGTGAAATAAAGGTAGTACCAAGAAGAAAAGCAAAGATCATTAGGGATTATGGAAAACAGATGGCAGGTGATGATTGTGTGGCAGGTAGACAGGATGAGGAT

>AY037282

TTTTTAGATGGAATAGATAAGGCCCAAGAAGACCATGAGAAATATCACAGTAATTGGAGAGCAATGGCTGATGATTTTAACCTGCCACCTATAGTAGCAAAAGAGATAATAGCCTGCTGTGATAAATGTCAGCTAAAAGGAGAAGCCATACATGGGCAAGTAGACTGTAGTCCAGGAATATGGCAGCTAGATTGTACACACTTAGAAGGAAAAGTTATCCTGGTAGCAGTCCATGTAGCCAGTGGATATTTAGAAGCAGAAGTTATTCCAGCAGAAACAGGGCAAGAAACAGCATACTTTCTCTTAAAATTAGCAGGAAGATGGCCAGTAAAAACAATACATACAGACAATGGCCCCAATTTCGTCAGTACTGCGGTTAAAGCCGCCTGTTGGTGGGCGGGGATCAAGCAGGAATTTGGCATTCCCTACAATCCCCAAAGTCAAGGAGTAGTAGAATCTATGAATAAAGAATTAAAGAAAATTATAGGACAGGTAAGAGATCAGGCTGAACATCTCAAGACAGCAGTACAAATGGCAGTATTCATCCACAATTTTAAGAGAAAAGGGGGGATTGGGGGGTACAGTGCAGGGGAAAGAATAATAGACATAATAGCAACAGACATACAAACTAAAGAATTACAAAAACAAATTACAAAAATTCAAAATTTTCGGGTTTATTACAGGGACAGCAGAGATCCAATTTGGAAAGGACCAGCAAAACTTCTCTGGAAAGGTGAAGGGGCAGTAGTGATACAAGAGAATAGTGACATAAAAGTAGTGCCAAGAAGAAAAGCAAAGATCATTAGGGATTATGGAAAACAGATGGCAGGTGATGATTGTGTGGCAGGTAGACAGGATGAGGAT

>AY037283

TTTTTAGATGGGATAGATAAGGCACAGGAGGAACATGAAAAATATCACAACAATTGGAGAGCAATGGCTAGTGATTTTAATTTGCCACCTGTAGTAGCAAAAGAAATAGTAGCTAGCTGTGATAGGTGTCAGCTAAAAGGGGAAGCCATACATGGACAAGTAGATTGTAGTCCAGGGATATGGCAATTAGATTGTACACATCTAGAAGGGAAAGTTATCCTGGTAGCAGTCCATGTAGCTAGTGGGTACCTAGAAGCAGAAGTTATCCCAGCAGAAACAGGACAAGAAACAGCCTACTTCATACTAAAGTTAGCAGGAAGATGGCCAGTAAAAACAATACATACAGACAACGGCCCCAATTTCATCAGTGCCTCAGTTAAGGCAGCCTGTTGGTGGGCAGGTATCCAGCAGGAATTTGGAATTCCCTACAACCCCCAAAGTCAAGGAGTAGTAGAATCTATGAATAAAGAGTTAAAGAAGATCATAGGACAGATAAGAGATCAAGCTGAACATCTTAAGACAGCAGTACAAATGGCAGTATTCATTCACAATTTTAAAAGAAAAGGGGGGATTGGGGGATACAGTGCAGGGGAAAGGATAATAGACATAATATCAACAGACATACAAACTAGAGAATTACAAAAACAAATTATAAAAATTCAAAATTTCCGGGTTTATTACAGGGACAGCAGAGACCCAGTTTGGAAAGGACCAGCAAAACTACTCTGGAAAGGTGAAGGGGCAGTAGTCATACAAGACAATAGTGAAATAAAGGTAGTACCAAGAAGAAAAGCAAAGATCATTAGGGATTATGGAAAACAGATGGCAGGTGATGATTGTGTGGCAGGTAGACAGGATGAGGAT

>AF385934

TTTTTAGATGGGATAGATAAGGCACAGGAGGAACATGAAAAATATCACAACAATTGGAGAGCAATGGCTAGTGATTTTAATCTGCCACCTGTAGTAGCAAAAGAAATAGTAGCTAGCTGTGATAAGTGTCAGGTAAAAGGGGAAGCCATGCATGGACAAGTAGATTGTAGTCCAGGGATATGGCAATTAGATTGTACACATTTAGAAGGAAAAATTATCCTGGTAGCAGTCCATGTAGCTAGTGGGTACCTAGAAGCAGAAGTTATCCCAGCAGAAACAGGACAGGAAACAGCCTACTTCCTACTAAAGTTAGCAGGAAGATGGCCAGTAAAAACAATACATACAGATAATGGCACCAATTTCAGCAGTGCCGCGGTTAAGGCAGCCTGTTGGTGGGCAGGTATCCAGCAGGAGTTTGGAATTCCCTACAACCCCCAAAGTCAAGGAGTAGTAGAATCTATGAATAAAGAGCTAAAGAAAATCATAAAACAGGTGAGAGATCAAGCTGAACATCTTAAGACAGCAGTACAAATGGCAGTATTCATCCACAATTTTAAAAGAAAAGGGGGGATTGGGGGATACAGTGCAGGGGAAAGAATAATAGACATAATATCAACAGACATACAAACTAAAGAATTACAAAAACAAATTATAAAAATTCAAAATTTCCGGGTTTATTACAGGGACAGCAGAGACCCAGTTTGGAAAGGACCAGCAAAGCTACTCTGGAAAGGTGAAGGGGCAGTAGTCATACAAGACAATAGTGAAATAAAGGTAGTACCAAGAAGAAAAGCAAAGATCATTAGGGATTATGGAAAACAGATGGCAGGTGATGATTGTGTGGCAGGTAGACAGGATGAGGAT

>AF385936

TTTTTAGATGGGATAGATAAGGCACAGGAGGAACATGAAAAATATCACAACAATTGGAGAGCAATGGCTAGTGATTTTAATCTGCCACCTGTAGTAGCAAAAGAAATAGTAGCTAGCTGTGATAAGTGTCAGCTAAAAGGGGAAGCCATGCATGGACAAGTAGATTGTAGTCCAGGGATATGGCAATTAGACTGTACACATTTAGAAGGAAAAACTATCCTGGTAGCAGTCCATGTAGCCAGTGGGTACCTAGAAGCAGAAGTTATCCCAGCAGAAACAGGACAAGAAACAGCCTACTTCATACTAAAGTTAGCAGGAAGATGGCCAGTAAAAACAATACATACAGACAATGGCCCCAATTTCATCAGTGCCATGGTTAAGGCAGCCTGTTGGTGGGCAGGTATCCAACAGGAATTTGGAATTCCCTACAACCCCCAAAGTCAGGGAGTAGTAGAATCTATGAATAAAGAGCTAAAAAAGATCATAAGCCAGGTAAGAGATCAAGCTGAACATCTTAAGACAGCAGTGCAAATGGCAGTATTCATCCACAATTTTAAAAGAAAAGGGGGGATTGGGGGATACAGTGCAGGGGAAAGAATAATAGACATAATATCAACAGACATACAAACTAGAGAATTACAAAAACAAATTATAAAAATTCAAAATTTCCGGGTTTATTACAGGGACAGCAGAGACCCAGTTTGGAAAGGACCAGCAAAGCTACTCTGGAAAGGTGAAGGGGCAGTAGTCATACAAGACGATAGTGAAATAAAGGTAGTACCCAGAAGAAAAGCAAAGATCATTAGGGACTATGGAAAACAGATGGCAGGTGATGATTGTGTGGCAGGTAGACAGGATGAGGAT

>AF385935

TTTTTAGATGGGATAGATAAGGCACAAGAGGACCATGAAAAATATCACAACAATTGGAGAGCAATGGCTAGTGACTTTAATCTGCCACCTGTAGTAGCAAAAGAAATAGTAGCTAGCTGTGATAAGTGTCAGCTAAAAGGGGAAGCCATGCATGGACAAGTAGATTGTAGTCCAGGGATATGGCAATTAGATTGTACACATTTAGAAGGAAAAGTTATCCTGGTAGCAGTCCATGTAGCTAGTGGGTACCTAGAAGCAGAAGTTATCCCAGCAGAAACAGGACAGGAAACAGCCTACTTTATACTAAAGTTAGCAGGAAGATGGCCAGTAAGAACAATACATACAGACAATGGCCCCAATTTCAGCAGTGCTGCAGTTAAGGCAGCCTGTTGGTGGGCAGGTGTCCAGCAGGAATTTGGAATTCCCTACAATCCCCAAAGTCAAGGAGTAGTAGAATCTATGAATAAAGAGCTAAAGAAGATCATACACCAAGTGAGAGATCAAGCTGAACATCTTAAGACAGCAGTACAAATGGCAGTATTCATTCACAATTTTAAAAGAAAAGGGGGGATTGGGGGATACAGTGCAGGGGAAAGAATAATAGACATAATAGCAACAGACATACAAACTAAAGAATTACAAAAACAAATTATAAAAATTCAAAATTTCCGGGTTTATTACAGGGACAACAGAGACCCAGTTTGGAAAGGACCAGCAAAACTGCTCTGGAAAGGTGAAGGGGCAGTAGTCATACAAGATAATAGTGAAATAAAGGTAGTACCAAGAAGAAAAGCAAAGATCATTAGGGATTATGGAAAACAGATGGCAGGTGATGATTGTGTGGCAGGTAGACAGGATGAGGAT

>AJ300450

TTCCTAGAAGGAATAGACCAGGCACAAGAAGATCATGAAAAGTATCATAGCAATTGGAGAGCATTAGCTAGTGATTTTGGATTACCACCAGTGGTGGCCAAAGAAATCATTGCTAATTGTCCTAAATGTCATATAAAAGGGGAAGCAATTCATGGTCAGGTAGACTGCAGTCCAGAAGTATGGCAAATAGATTGCACACATCTAGAAGGCAAAATCATAATAGTTGCTGTTCATGTGGCAAGTGGATTCATAGAAGCAGAGGTAATACCAGCAGAAACAGGACAAGAAACTGCCTACTTCCTGTTAAAATTAGCTGCAAGATGGCCTGTTAAAAGAATACATACAGACAATGGGCCTAATTTTACAAGTGCAACCATGAAGGCTGCATGCTGGTGGACCAACATACAACATGAGTTTGGAATACCATATAATCCACAAAGTCAAGGAGTAGTAGAGGCCATGAACAAGGAATTAAAATCAGTTATACAGCAGGTGAGGGACCAAGCAGAACACTTAAAAACAGCAGTACAAATGGCAGTATTTGTTCACAATTATAAAAGAAAAGGGGGGATTGGGGGGTACACTGCAGGAGAAAGGATAATAGACATATTAGCATCCCAAATACAAACAACAGAATTACAAAAACAAATTTTTAAAATTCAAAAATTTCAGGTCTATTACAGAGACAGCAGAGATCCTATTTGGAAAGGACCGGCACAGCTCCTGTGGAAAGGTGAGGGAGCAGTAGTCATACAAGATAAAGGAGATATTAAGGTAGTACCAAGAAGAAAGGCAAAAATAATCAGACATTATGGAAAACAGATGGCAGGTACTGATAGTATGGCAAGTGGACAGACAGAG

>AJ302646

TTCCTAGAAGGAATAGACCAGGCACAAGAAGATCATGAAAAGTATCATAGCAATTGGAGAGCATTAGCTAGTGATTTTGGATTACCACCAGTGGTGGCCAAAGAAATCATTGCTAATTGTCCTCAATGTCATATAAAAGGGGAAGCAATTCATGGTCAGGTAGACTGCAGTCCAGAAGTATGGCAAATGGATTGCACACATCTAGAAGGCAAAATCATAATAGTTGCTGTCCATGTGGCAAGTGGATTCATAGAAGCAGAAGTAATACCAGCAGAAACAGGACAGGAAACTGCCTACTTCCTGTTAAAATTAGCTGCAAGATGGCCTGTTAAGGTAATACATACAGACAATGGGCCTAATTTTACAAGTGCAGCCATGAAGGCTGCATGTTGGTGGGCCAACATAAAACATGAGTTTGGAATACCATATAATCCACAAAGTCAAGGAGTAGTAGAGGCCATGAACAAGGAATTAAAATCAATTATACAGCAGGTGAGGGACCAAGCAGAACACTTAAAAACAGCAGTACAAATGGCAGTATTTGTGCACAATTATAAAAGAAAAGGGGGGATTGGGGGGTACACTGCAGGAGAAAGGATAATAGACATATTAGCATCACAAATACAAACAACAGAATTACAAAAACAAATTTTTAAAATTCAAAAATTTCAGGTCTATTACAGAGACAGCAGAGATCCTATTTGGAAAGGACCGGCACAGCTCCTGTGGAAAGGTGAGGGAGCAGTAGTCATACAAGATAAAGGAGACATTAAGGTAGTACCAAGAAGAAAGGCAAAAATAATCAGACATTATGGAAAACAGATGGCAGGTACTGATAGTATGGCAAGTGGACAGACAGAG

>AJ302647

TTCCTAGAAGGAATAGACCAGGCACAAGAAGATCATGAAAAGTATCATAGCAATTGGAGAGCATTAGCTAGTGATTTTGGATTGCCACCAGTGGTGGCCAAAGAAATCATTGCTAATTGTCCTAAATGTCATATAAAAGGGGAAGCAATTCATGGTCAGGTAGACTACAGTCCAGAAGTATGGCAAATAGATTGCACACATCTAGAAGGCAAAATCATAATAGTTGCTGTTCATGTGGCAAGTGGATTCATAGAAGCAGAAGTAATACCAGCAGAAACAGGACAAGAAACTGCCTACTTCCTGTTAAAATTAGCTGCAAGATGGCCTGTTAAAATAATACATACAGACAATGGGCCTAATTTCACAAGTGCAACCATGAAGGCTGCATGTTGGTGGACAGGCATAAAACATGAGTTTGGAATACCATATAATCCACAAAGTCAAGGAGTAGTAGAGGCCATGAACAAGGAATTAAAATCAATTATACAGCAGGTGAGGGACCAAGCAGAACACTTAAAAACAGCAGTACAAATGGCAGTATTTGTTCACAATTATAAAAGAAAAGGGGGGATTGGGGGGTACACTGCAGGAGAAAGGATAATAGACATATTAGCATCACAAATACAAACAACAGAATTACAAAAACAAATTTTTAAAATTCAAAAATTTCAGGTCTATTACAGAGACAGCAGAGATCCTATTTGGAAAGGACCGGCACAGCTCCTGTGGAAAGGTGAGGGAGCAGTAGTCATACAAGATAAAGGAGACATTAAGGTAGTACCAAGAAGAAAGGCAAAAATAATCAGACATTATGGAAAACAGATGGCAGGTACTGATAGTATGGCAAGTGGACAGACAGAG

>AF349680

TTCCTAGACGGGATAGGAAAAGCACAGGAAGAACATGAAAAATATCATAGCAATTGGAGAGCAATGGCAGAAGAATTTCAAATACCTCAAATAGTAGCAAAAGAAATAGTAGCCCAATGCCCCAAGTGTCAGGTAAAAGGAGAAGCAGTACATGGACAGGTAGATGCTAGTCCAGGTACTTGGCAAATGGATTGCACACATCTAGAAGGAAAAATCATCATAGTAGCAGTCCATGTAGCCAGTGGATACATAGAAGCAGAAGTAATACCAGCAGAGACAGGAAAAGAGACAGCTCATTTCCTGTTAAAATTAGCAGCCAGATGGCCAGTGAAACATTTACACACAGACAATGGAACAAACTTTACAAGTGCAGCAGTGCAAGCAGACTGTTGGTGGGCTCAAATAGAGCACACCTTTGGAGTACCCTACAATCCTCAAAGCCAAGGAGTGGTAGAATCAATGAATCATCAATTGAAGATAATAATAGGGCAAATTAGAGATCAGGCAGAAAAGTTAGAGACAGCAGTTCAAATGGCAGTGCTCATTCACAATTTTAAAAGAAAAGGGGGGATTGGGGGGTACAGTGCAGGAGAAAGGATAATAGACATAATAGCATCAGACTTACAAACAACCAAATTACAAAACCAAATTTCAAAAATTCAAAATTTTCGGGTTTATTTCAGAGAAGGAAGGGATCAGCAGTGGAAGGGACCAGCTAAACTCATCTGGGAAGGAGAAGGGGCTGTCGTGATCCAGGATCAAGAGGATCTAAAGGTAGTCCCTAGAAGAAAGTGTAAGATCATAAAAGATTATGGCAGAAAAGAGGTGGGAGAGACCAATATGGAAGGTAGACAGGAGGAA

>AF349681

TTTTTAGATGGTATAGAAAAAGCTCAAGAAGAGCATGAAAAGCATCATAGCAATTGGAGAGCTATGGCAGAAGAATTTAAGATACCACAAATAGTGGCTAAAGAAATAGTAGCTCAATGTCCTAAGTGCCAGGTGAAAGGAGAAGCAGTTCATGGACAGGTAGATGCCAGTCCAGGAACATGGCAAATGGACTGTACACATTTAGAAGGAAAAATCATCATAGTGGCAGTCCATGTAGCCAGTGGATACATAGAAGCAGAAGTCATACCAGCAGAAACAGGAAAAGAGACAGCTTACTTCCTGTTAAAACTAGCAGCAAGATGGCCAGTGAAACATTTGCACACAGACAATGGAGCAAATTTTACAAGTGCAGCAGTACAAGCAGTCTGTTGGTGGGCTCAAATAGAGCACACCTTTGGGGTACCCTACAATCCTCAAAGCCGAGGAGTGGTAGAATCAATGAATCACCAATTGAAAACAATTATAGGCCAGATTAGAGACCAAGCAGAAAAATTAGAGACAGCAGTACAAATGGCAGTGCTTGTCCACAATTTTAAAAGAAAAGGGGGGATTGGGGGGTACAGTGCAGGAGAAAGAATAATAGACATAATAGCATCAGACCTACAAACAAATAAATTACAAAACCAAATTTCAAAAATTCAAAATTTTCGGGTCTATTACAGAGAAGGAAGGGATCAGCAGTGGAAGGGACCGGCTAAACTCATCTGGAAAGGAGAAGGGGCTGTGGTGATCGAGGATCAGCAGGATCTAAAGGTAGTTCCTAGAAGAAAGTGTAAAATCATAAAAGATTATGGCAGAAAAGATGTGGGTGAGACCAGTATGGAGGGTAGACAGGAGGAAAAT

>AF328295

TTCCTGGAAGGAATAGATAAAGCTCAGGAGGAACATGACAAATACCATAATAATTGGAGATCATTAAGTCAGGAATTTAGCATACCCCCTATAGTGGCAAAAGAAATAGTTGCACAGTGCCCAAAGTGTCAGGTAAAAGGAGAACCTATCCATGGACAGGTGGATGCGGCCCCAGGAACATGGCAAATGGATTGCACCCATCTAGAAGGAAAGGTTATCATAGTAGCAGTCCATGTGGCCAGTGGATATCTAGAAGCAGAAGTGATACCAGCAGAAACAGGAAAAGAAACAGCACATTTCCTGCTAAAACTAGCAGGAAGATGGCCAGTGAAACATTTACATACTGACAATGGTCCAAACTTTGTCAGTGAGAAGGTAGCCACAGTGTGTTGGTGGGCTCAGATAGAGCACACCACAGGAATACCCTATAACCCCCAAAGTCAAGGAGTTGTGGAAGCAAAAAATCATCATCTTAAGGTAATTATAGAGCAAGTTAGGGATCAAGCAGAGAGACTGGAGACAGCAGTACAAATGGCAGTACTCATTCACAATTTTAAAAGAAAAGGGGGGATAGGGGAGTACAGTCCAGGAGAAAGAATAGTGGATATAATAACAACAGATATTCTAACTACCAAATTACAACAAAATATTTCAAAAATTCAAAATTTTCGGGTTTATTACAGAGAAGGAAGGGAGCAGCTCTGGAAAGGACCAGCAGAACTCATTTGGAAAGGAGAAGGAGCAGTGGTAATCAAAGAAGGAACAGACTTAAAAGTTGTGCCCAGAAGAAAAGCTAAAATAATCAGGGATTATGGAAAAACAGTGGATAGTAATCCCCACATGGAGGATAGACAGGAG

>AJ404325

TTTTTAGATGGTATAGATAAAGCCCAAGAAGATCATGAAAGATATCACACCAATTGGAGAGCAATGGCTAGTGATTTTAATTTGCCACCTATAGTAGCAAAAGAAATAGTGGCCAGCTGTGATAAATGTCAGCTAAAAGGGGAAGCCATGCATGGGCAGGTAGACTGTAGTCCAGGAATATGGCAATTAGATTGTACACATTTAGAAGGAAAAATTATCCTGGTGGCAGTCCATGTAGCCAGTGGCTATATAGAAGCAGAAGTTATCCCAACAGAAACAGGACAGGAAACAGCATACTTTATATTAAAACTAGCAGGAAGATGGCCAGTAAAAGTAATACACACAGACAATGGCAGAAATTTCACCAGTACTGCAGTAAAGGCAGCATGCTGGTGGGCAAATGTTACACAAGAATTTGGAATTCCCTACAATCCCCAAAGCCAAGGAGTAGTGGAATCTATGAATAAAGAATTAAAGAAAATCATAGGGCAGGTCAGGGATCAAGCAGAACACCTTAAGACAGCAGTACAGATGGCAGTATTCATTCACAATTTTAAAAGAAAAGGGGGGATTGGGGGGTACAGTGCAGGGGAAAGAATAATAGACATAATAGCATCAGATATACAAACTAAAGAACTACAAAAACAAATTACAAAAATTCAAAATTTTCGGGTTTATTACAGGGACAGCAGAGACCCAATTTGGAAAGGACCAGCTAAACTACTCTGGAAAGGTGAAGGGGCAGTAGTAATACAAGACAATAGTGAAATAAAGGTAGTCCCAAGAAGAAAAGCAAAGATCATTAAAGATTATGGAAAACAGATGGCAGGTGATGATTGTGTGGCAGGTAGACAGGATGAGGAT

>U79412

TTCTTGGAAAAGATAGAGCCAGCACAAGAAGAACATGATAAATACCATAGTAATGTAAAAGAATTGGTATTCAAATTTGGATTACCCAGACTAGTGGCCAAACAGATAGTAGACACATGTGATAAATGTCATCAGAAAGGAGAAGCTATACATGGGCAGGTAAATTCAGATCTAGGGACTTGGCAAATGGATTGTACCCATCTAGAGGGAAAAATAATCATAGTTGCAGTACATGTAGCTAGTGGATTCATAGAAGCAGAAGTAATTCCACAAGAGACAGGAAGACAGACAGCACTATTTCTGTTAAAATTGGCAAGCAGATGGCCTATTACGCATCTACACACAGATAATGGTGCCAACTTTACTTCGCAAGAAGTAAAGATGGTTGCATGGTGGGCAGGGATAGAGCACACCTTTGGGGTACCATACAATCCACAGAGTCAGGGAGTAGTGGAAGCAATGAATCACCATCTAAAAAATCAAATAGATAGAATCAGGGAACAAGCAAATTCAATGGAAACCATAGTATTAATGGCAGTTCATTGCATGAATTTTAAAAGAAGGGGAGGAATAGGGGATATGACTCCAGCAGAAAGGTTACTTAACATGATCACTACAGAACAAGAAATACAATTCCAACAATCAAAAAACTCAAAATTTAAAAATTTTCGGGTCTATTACAGAGAAGGCAGAGATCAACTGTGGAAGGGACCCGGTGAGCTATTGTGGAAAGGGGAAGGAGCAGTCATCTTAAAGGTAGGGACAGACATTAAGGTAGTACCCAGAAGAAAGGCTAAGATTATCAAAGATTATGGA

>AF286236

TTTTTGGATGGAATAGATAAGGCTCAAGAGGAGCATGAAAGATATCATAGCAATTGGAGAGCAATGGCTAGTGATTTTAATCTGCCACCTATAGTAGCAAAAGAAATAGTAGCCAGCTGTGATAAATGTCAGCTAAAAGGGGAAGCCATGCATGGACAAGTAGACTGCAGTCCAGGGATATGGCAATTAGATTGTACACACCTAGAGGGAAAAGTTATTCTGGTAGCAGTCCATGTAGCCAGTGGCTATCTAGAAGCAGAAGTTATCCCGGCAGAAACAGGACAGGAAACAGCATACTTTGTACTAAAACTAGCAGGAAGATGGCCAGTAACAGTAATACATACAGACAATGGCAGCAATTTCACCAGTGCTGCAGTCAAAGCAGCCTGCTGGTGGGCAGGGATCCAACAGGAATTTGGAATTCCCTACAATCCCCAAAGCCAAGGAGTAGTAGAATCCATGAATAAAGAATTAAAGAAAATCATAGGGCAGGTAAGAGATCAAGCTGAACACCTTAAGACAGCAGTACAAATGGCAGTATTCATTCACAATTTTAAAAGAAAAGGGGGGATTGGGGGGTACAGTGCAGGGGAAAGAATAATAGACATAATAGCAACAGACATACAAACTAAAGAATTACAAAAACAAATTACAAAAATTCAAAATTTTCGGGTTTATTACAGAGACAGCAGAGACCCGATTTGGAAAGGACCAGCAAAACTACTCTGGAAAGGTGAAGGAGCAGTAGTCATACAAGACAATAGTGAAATAAAGGTAGTGCCAAGAAGGAAAGCAAAGATCATTAGGGACTATGGAAAACAGATGGCAGGTGATGATTGTGTGGCAAGTAGACAGGATGAGAAT

>AF457101

TTTTTGGATGGAATAGATAAGGCTCAAGAAGAACATGAAAAATATCATAGCAATTGGAGAGCAATGGCTAGTGATTTTAATCTGCCACCTATAGTAGCAAAAGAAATAGTAGCTAGCTGTGATAAATGTCAGCTAAAAGGAGAAGCCATACATGGACAAGTAGACTGCAGTCCAGGGATATGGCAACTAGATTGTACACACCTAGAGGGAAAAATTATCCTGGTAGCAGTCCATGTAGCCAGTGGCTATATAGAAGCAGAAGTTATCCCAGCAGAAACAGGACAGGACACAGCATACTTTGTACTAAAACTAGCAGGAAGATGGCCAGTAACAACTATACATACAGATAATGGCAGCAATTTCACCAGTGCTGCAGTCAAAGCAGCCTGCTGGTGGGCAGGGATCCAGCAGGAATTTGGAATTCCCTACAATCCTCAAAGTCAAGGAGTAGTAGAATCCATGAATAAAGAATTAAAGAAAATCATAGGGCAGGTAAGAGAGCAAGCTGAACACCTCAAGACAGCAGTGCAAATGGCAGTATTCATTCACAATTTTAAAAGAAAAGGGGGGATTGGGGGGTACAGTGCAGGGGAAAGAATAATAGACATAATAGCAACAGACATACAAACTAAAGAATTACAAAAACAAATTATACAAATTCAAAATTTTCGGGTTTATTACAGAGACAGCAGAGACCCCATTTGGAAAGGACCAGCAAAACTACTCTGGAAAGGTGAAGGAGCAGTAGTCATACAAGACAATAGTGAAATAAAGGTAGTGCCAAGAAGGAAAGCAAAGATCATTAGGGACTATGGAAAACAGATGGCAGGTGATGATTGTGTGGCAAGTAGACAGGATGAGAAT

>AF468659

TTCCTGGAAAACATAGAGCCTGCAGTAGAAGACCATGAAAAGTATCATAGTAATTGGAAGTACCTAAGAGATCAATACAAAATCCCCGCACTATTGGCTAAAGAAATAGTGAACAAATGTTCCAAGTGTCAGGTACATGGGGAACCAAAACATGGGCAGGTAAATGCAGAATTAGGCATGTGGCAGATGGACTGCACGCATTTGGAAGGAAAGGTCATCTTAGTGGCGGTCCACGTAGCAAGTGGGTATGTCTGGGCTAGGATAATACCCCAGGAAACAGGCAGACAAACTGCATTGAAGCTCTTAGAATTAGCAGCAACATGGCCTGTAACCCACCTACATACAGATAATGGCCCCAATTTCGTCTCCAAGGAATTGGAAGCAGCCTGCTGGTGGGCAGACATACAACACACCACAGGGGTGCCATACAACCCGCAAAGTCAAGGGGTAGTAGAAAACATGAACAAACAACTCAAGGAAACAATCACGAAAATCAGAGAAGAGGTCACATACTTAGAAACAGCAGTAGCACAGGCATGCTACATTCATAATTTTAAAAGAAAGGGAGGAATAGGGGATATGTGCCCTACAGAAAGAATAGTAAATATGATTCACACAGAATTAGAAACACAACACTTAAACACACAAAATTCCAAATTTCAAAAATTTCGGGTTTATTACAGGCAAGGAGCTAACCCTCTTTGGCAAGGACCAGCAGTACTCCTCTGGAAAGGTGAAGGAGCAGTAGTAGTCCAAACTCAAGCAGAGATTATTACAGTCCCTAGAAGGAAAGCAAAGATCATCAAGCCATATGGAGAGGCAAAGACAGAAGAT

>AF468658

TTCATGGAGAACATAGAGCCCGCAGTAGAGGACCATGAAAAATATCATAGTAATTGGAAGTATTTAAGGGATCAATACAAAATCCCGGCACTATTGGCTAAAGAAATAGTAAACAAGTGCTCCAAATGTCAGATACATGGGGAACCAAAACATGGGCAAGTAAATGCAGAATTAGGCATATGGCAAATGGACTGCACACATCTGGAAGGCAAAGTTATCTTAGTGGCAGTCCACGTAGCCAGTGGGTATGTTTGGGCTAGGATAATACCCCAAGAAACAGGCAGACAAACTGCATTAAAGCTATTAGAATTAGCAGCAACATGGCCAGTAACTCACCTACATACAGATAATGGCCCCAATTTCATCTCTAAGGAACTGGAGGCAGCTTGTTGGTGGGCAAACATACAACACAGTACAGGGGTGCCATACAACCCGCAAAGTCAAGGGGTAGTAGAAAACATGAACAAACAACTCAAGGAAACAATCCAAAAGATTAGAGATGAGGTCACATACTTAGAAACAGCAGTAGCACAAGCATGCTACATTCATAATTTTAAAAGAAAGGGAGGAATAGGGGATATGTGCCCTACAGAGAGGTTAGTGAACATGATCCACACAGAATTAGAAACACAACACTTAAACACACAAAGTTCCAAATTTCAAAAATTTCGGGTTTATTACAGGCAAGGAGCTAATCCTCATTGGCAAGGACCAGCAGTACTTCTCTGGAAGGGTGAAGGAGCAGTAGTGGTCCAAACCCAAGCAGAGATCATCACAGTCCCTAGAAGGAAAGCAAAGATCATCAAGCCATATGGAGAGGCAAAGGCAGAAGATGTGGGTAGTGCAGCCCATACAAGTAATGAC

>AJ488926

TTTTTGGATGGAATAAGTCAGGCTCAAGATGAACATGACAAATATCACAGCAATTGGAGAGCAATGGCTAGTGATTTTAACCTACCACCTGTGGTAGCAAAAGAAATAGTAGCTAGCTGTGATAAATGTCAAATAAAAGGAGAAGCCATGCATGGACAAGTAGACTGTAGTCCAGGAATATGGCAATTAGATTGTACACATCTAGAAGGACAAATTATCCTGGTAGCAGTTCATGTAGCCAGTGGCTATATAGAAGCAGAAGTTATTCCAGCAGAAACAGGGCAGGAAACAGCATACTTTCTTTTAAAATTAGCAGGAAGATGGCCAGTAAAAACGGTGCATACAGACAATGGCAGAAATTTTACCAGTGCTACTGTTAAGGCCGCCTGTTGGTGGGCAGGTATTAAGCAGGAATTTGGAATTCCCTACAATCCCCAAAGTCAAGGAGTAGTAGAATCTATGAATAAAGAATTAAAGAAAATTATAGAACAGGTAAGAGATCAAGCTGAACATCTTAAGACAGCAGTACAAATGGCAGTATTCATTCACAATTTTAAAAGAAAAGGGGGGATTGGGGGGTACAGTGCAGGGGAAAGAATAATAGACATAATAGCAACAGACATACAAACTAGAGAATTACAAAAACAAATCACAAAAATTCAAAATTTTCGGGTTTATTACAGGGACAGCAGAGATCCAATTTGGAAAGGACCAGCAAAGCTTCTCTGGAAAGGTGAAGGGGCAGTGGTAATACAAGAAAATAGTGACATAAAGGTAGTACCAAGAAGAAAAGCAAAGATCATTAGGGATTATGGAAAACAGATGGCAGGTGATGGTTGTGTGGCAAGTAGACAGGATGAGGAT

>AJ488927

TTTTTGGATGGGATAAGTCAGGCTCAAGAGGAACATGACAAATATCACAGCAATTGGAGAGCAATGGCTAGTGATTTTAACCTACCACCTGTGGTAGCAAAAGAAATAGTAGCTAGCTGTGATAAATGTCAACTAAAAGGAGAAGCCATGCATGGACAAGTAGACTGTAGTCCAGGAATATGGCAATTAGACTGTACACATCTAGAAGGAAAAATTATCCTGGTAGCAGTCCATGTAGCCAGTGGCTATATAGAAGCAGAAGTTATTCCAGCAGAGACAGGGCAGGAAACAGCATACTTTCTTTTAAAATTAGCAGGAAGATGGCCAGTAAAAGTAGTGCATACAGACAATGGCAGCAATTTTACTAGTGCTGCCGTTAAGGCCGCCTGTTGGTGGGCAGGTATTAAGCAGGAATTCGGAATTCCCTACAATCCCCAAAGTCAAGGAGTAGTGGAATCTATGAATAAAGAATTAAAGAAAATTATAGGACAAGTAAGAGATCAAGCTGAACATCTTAAGACAGCAGTACAAATGGCAGTATTCATTCACAATTTTAAAAGAAAAGGGGGGATTGGGGGGTACAGTGCAGGGGAAAGAATAATAGACATAATAGCAACAGACATACAAACTAGAGAATTACAAAAACAAATCATAAAAATTCAAAATTTTCGGGTTTATTACAGGGACAGCAGAGATCCAATTTGGAAAGGACCAGCAAAGCTTCTCTGGAAAGGTGAAGGGGCAGTAGTAATACAAGAAAATAGTGACATAAAAGTAGTACCAAGAAGAAAGGTAAAGATCATTAAGGATTATGGAAAACAGATGGCAGGTGATGGTTGTGTGGCAAGTAGACAGGATGAGGAT

>AF447763

TTCCTGGATGGAATTAATGAAGCACAGGAAGACCATGATAAATATCACAGTAATTGGAAAGCTTTAGCTGATGAATATAATCTGCCCCCAGTTGTGGCTAAAGAAATTATTGCTCAGTGTCCAAAATGCCATATAAAAGGAGAGGCTATACATGGACAGGTGGACTACAGTCCAGAAATCTGGCAAATAGACTGTACCCACCTAGAAGGAAAGGTCATCATAGTAGCAGTGCATGTAGCTAGTGGTTTCATAGAAGCAGAAGTCATACCAGAAGAAACAGGAAGAGAAACCGCTTACTTCATCCTAAAATTGGCAGGAAGATGGCCTGTAAAGAAAATACATACAGATAATGGACCAAATTTTACTAGTACAGCAGTGAAGGCAGCCTGCTGGTGGGCACAAATTCAACATGAATTTGGGATTCCATATAATCCTCAAAGTCAAGGAGTAGTAGAATCTATGAATAAACAATTAAAGCAAATTATAGAGCAAGTCAGGGACCAAGCAGAGCAACTGAGGACAGCAGTAATCATGGCAGTGTATATCCACAATTTTAAAAGAAAAGGGGGGATTGGGGAGTACACTGCAGGGGAAAGACTATTAGACATACTAACTACAAATATACAGACAAAACAATTACAAAAACAAATTTTAAAAGTTCAAAATTTTCGGGTTTATTATAGGGACGCCAGAGATCCAATTTGGAAGGGACCAGCGCGACTACTGTGGAAAGGTGAAGGGGCAGTAGTAATAAAAGAAGGAGAAGACATTAAAGTAGTACCCAGGAGAAAAGCAAAAATCATAAAAGAGTATGGAAAACAGATGGCAGGTGCAGGTGGTATGGATGATAGACAGAATGAG

>AY159321

TTCCTAGATAACATGGAAAAAGCACAGGAAGAACATGATTTATACCATAATAATTGGAGATCCTTAGCACAAGAATTTGGTTTACCAGGGATAGTGGCAAAAGAAATAGTGGCACAGTGTCCTAAATGTCAAATACATGGAGAGCCAATACATGGCCAAGTAGATGCAAGCCCAGGAACTTGGCAAATGGACTGCACACATTTAGAAGGAAAGATAATCATAGTGGCAGTCCACGTAGCAAGTGGACACATAGAAGCAGAAGTAATACCAGCAGAAACAGGAGAGAAAACAGCTTATTTTCTGCTAAAATTAGCTGGCAGATGGCCAGTATCACACTTGCACACAGATAATGGACCAAATTTCACCAGTGAAAAGGTAGCAACAGTATGCTGGTGGGCTAAGATAGAGCATACCACAGGAGTTCCCTATAACCCCCAGAGTCAAGGAGTGGTAGAATCAATGAACAATCAGCTAAAGAAAATTATAGGACAAATTAGAGATCAGGCAGAAAAACTAGAGACAGCAGTTCAAATGGCAGTACTGATTCACAATTTTAAAAGAAAAGGGGGGATAGGGGAGTACAGTGCAGCAGAAAGAATAGTAGACATTATAGCCTCTGACCTCTTAACAAGCAAACTACAACAAAATATTCTCAAAATTCAAAATTTTCGGGTTTATTACAGAGAGGGAAGAGATCAGTTGTGGAAAGGACCAGCAGAACTTGTCTGGAAAGGGGAAGGAGCGGTGGTCATCAAGGAAGGTACTGACTTGAAAGTAATACCTAGAAGAAAAGCAAAGATCATTAAAGATTATGGAAAAAATGTG

>AY159322

TTTTTAGAGGGAATAGATAAAGCCCAGGAAGAACATGACAAATATCACAATAATTGGAGAGCGTTGTCTCAGGACTTTAACCTACCCCCCATAGTGGCAAAAGAGATAGTTGCACAATGTCCAAAATGTCAGACAAAGGGAGAACCTGTTCATGGGCAGGTAAATGCAGATCCAGGGACATGGCAGATGGACTGCACCCATTTAGAAGGAAAAATCATCATAGTAGCAGTCCATGTAGCCAGTGGATATTTAGAAGCAGAGGTAATACCAGCAGAGACAGGAAAAGAAACAGCGCACTTCCTGTTAAAGCTAGCAGGTAGGTGGCCAGTAAAACATCTACACACTGACAATGGTCCTAACTTTGTCAGTGAAAAGGTGGCCACAGTCTGCTGGTGGGCTCAAATAGAGCACACCACAGGAGTACCTTACAACCCACAGAGTCAAGGAGTAGTAGAAGCAAAAAATCATCATCTTAAGAAGATCATAGAACAAGTTAGGGATCAAGCTGAAAGGCTAGAAACAGCAGTGCAAATGGCAGTGCTAATTCACAATTTTAAAAGAAAAGGGGGGATAGGGGAGTACAGTCCTGGAGAAAGAATAGTAGACATAATAACCACAGACATCCTAACAACCAAATTACGACAAAATATTTCAAAAATTCAAAATTTTCAGGTTTATTACAGAGAAGGAAGGGACCAACAGTGGAAAGGACCAGCAGAGCTCATTTGGAAAGGAGAAGGCGCTGTGGTAATCAAAGAAGGAGTTGATTTAAAGGTGGTTCCTAGAAGAAAAGCAAAAATAATTAAGGACTATGGAAAA

>AJ549283

TTCATGGAGAACATTGAACCRGCCCGAGAGGATCATGAAAAATATCACAGCAATTGGAAATATCTCAGGGATACTTATGGCATTCCAACACTCCTAGCCAAAGAGATAGTCAACCATTGCCCCAAGTGTCACACACAAGGAGAACCCAAACATGGACAGGTAGAYTCAGATATAGGGATATGGCAAATGGATTGCACTCATTATGAAGGAAGTATCATCCTAGTAGCCGTTCATGTAGCTAGTGGCTACGTTTGGGCCAAAATTCTTCCCAGAGAAACAGGGAAAGAAACAGGCATTGCCCTGTTGGAACTTGCAGCGACATGGCCAATACATCATGTACACACTGACAATGGACCAAATTTCACCAGTCAGGAATTTGCAGCYGCTGCATGGTGGGCAAACATAGAACACAGTACTGGAGTGCCCTACAACCCACAAAGTCAAGGTGTAGTAGAAAACATGAATAAGCAAATCAAAACCACATTAGGGAAAATCAGGGATGAAGTTCAGTTCCTCTCTACAGCACTGGCACAAACGCTCTACATTCTAAATTTTAAAAGAARGGGAGGAATAGGGGATATCAGTCCAATAGAGAGGTTAGTCAACATGATTCATACAGATTTAGAACTACAACACTCAAACAACCAAATTACAAAATTTTCGAAATTTCGGGTTTATTTCAGAACAGGCGCTGATCCTTCGTGGAAGGGACCAGCACATCTTCTCTGGAAGGGGGAAGGAGCTTTGGTGATTAAGGATCAAGGTGAGGTAATAACAGTACCACGCAGGAAGGCAAAGATCATTCGCCCTTATGGCTCCAAG

>AY162223

TTTCTAGATGGAATAGATAAAGCTCAAGAAGAGCATGAAAGGTACCACAGCAATTGGAGAGCAATGGCTAATGAGTTTAATCTGCCACCCATAGTAGCAAAAGAAATAGTAGCTAGCTGTGATAAATGTCAGCTAAAAGGGGAAGCCATACATGGACAAGTCGACTGTAGTCCAGGGATATGGCAATTAGATTGTACCCATTTAGAGGGAAAAATCATCCTGGTAGCAGTCCATGTAGCTAGTGGCTACATGGAAGCAGAGGTTATCCCAGCAGAAACAGGACAAGAAACAGCATATTTTATATTAAAATTAGCAGGAAGATGGCCAGTCAAAGTAATACATACAGACAATGGCAGTAATTTTACCAGTACTGCAGTTAAGGCAGCCTGTTGGTGGGCAGGTATCCAACAGGAATTTGGAATTCCCTACAATCCCCAAAGTCAGGGAGTGGTAGAATCCATGAATAAAGAATTAAAGAAAATAATAGGACAAGTAAGAGATCAAGCTGAGCACCTTAAGACAGCAGTACAAATGGCAGTATTCATTCACAATTTTAAAAGAAAAGGGGGAATTGGGGGGTACAGTGCAGGGGAAAGAATAATAGACATAATAGCAACAGACATACAAACTAAAGAATTACAAAAACAAATTATAAGAATTCAAAATTTTCGGGTTTATTACAGAGACAGCAGAGACCCTATTTGGAAAGGACCAGCCGAACTACTCTGGAAAGGTGAAGGGGTAGTAGTAATAGAAGATAAAGGTGACATAAAGGTAGTACCAAGGAGGAAAGCAAAAATCATTAGAGATTATGGAAAACAGATGGCAGGTGCTGATTGTGTGGCAGGTGGACAGGATGAAGAT

>AY162224

TTTCTAGATGGAATAGATAAAGCTCAAGAAGAGCATGAAAAATATCACAGCAATTGGAGAGCAATGGCTAGTGAGTTTAATCTGCCACCCATAGTAGCAAAGGAAATAGTAGCCAGCTGTGATAAATGTCAGCTAAAAGGGGAAGCCATGCATGGACAAGTCGACTGTAGTCCAGGAATATGGCAATTAGACTGTACACATTTAGAAGGAAAAATCATCCTAGTAGCAGTCCATGTAGCCAGTGGCTACATGGAAGCAGAGGTTATCCCAGCAGAAACAGGACAAGAAACAGCATACTTTATACTAAAATTAGCAGGAAGATGGCCAGTCAAAGTAATACATACAGATAATGGCAGTAATTTCACCAGTACCGCAGTTAAGGCAGCCTGTTGGTGGGCAGATATCCAACGGGAATTTGGAATTCCCTACAATCCCCAAAGTCAAGGAGTAGTAGAATCCATGAATAAAGAATTAAAGAAAATCATAGGGCAAGTAAGAGATCAAGCTGAGCACCTTAAGACAGCAGTACAAATGGCAGTATTCATTCACAATTTTAAAAGAAAAGGGGGGATTGGGGGGTACAGTGCAGGGGAGAGAATAATAGACATAATAGCATCAGACATACAAACTAAAGAATTACAAAAACAAATTATAAAAATTCAAAATTTTCGGGTTTATTACAGAGACAGCAGAGACCCTATTTGGAAAGGACCAGCCAAACTACTCTGGAAAGGTGAAGGGGCAGTAGTAATACAAGATAATAGTGATATAAAGGTAGTACCAAGAAGGAAAGCAAAAATCATTAAGGACTATGGAAAACAGATGGCAGGTGCTGATTGTGTGGCAGGTAGACAGGATGAAGAT

>AY162225

TTTCTAGATGGGATAGATAAGGCTCAAGAAGAGCATGAAAAATATCACAGCAATTGGAGAGCAATGGCTAGTGAGTTTAATCTGCCACCCATAGTAGCAAAAGAAATAGTAGCCAGCTGTGATAAATGTCAGCTAAAAGGGGAAGCCATACATGGACAAGTCGACTGTAGTCCAGGAATATGGCAATTAGATTGTACACATTTAGAAGGAAAAATCATCCTGGTAGCAGTCCATGTAGCCAGTGGCTACATAGAAGCAGAGGTTATCCCAGCAGAAACAGGACAAGAAACAGCATATTATATACTAAAATTAGCAGGAAGATGGCCAGTTAAAATAATACATACAGATAATGGCAGTAATTTCACCAGTGCTGCAGTTAAAGCAGCCTGTTGGTGGGCAGGAATCCAACAGGAATTTGGAATTCCCTACAATCCCCAAAGTCAGGGAGTAGTAGAATCCATGAATAAAGAATTAAAGAAAATCATAGGGCAGGTAAGAGATCAAGCTGAGCACCTCAAGACAGCAGTACAAATGGCAGTATTCATTCACAATTTTAAAAGAAAAGGGGGGATTGGGGGGTACAGTGCAGGGGAAAGGATAATAGACATAATAGCAACAGACATACAAACTAGAGAATTACAAAAACAAATTATAAAAATTCAAAATTTTCGGGTTTATTACAGGGACAGCAGAGACCCTATTTGGAAAGGACCAGCCAAACTACTCTGGAAAGGTGAAGGGGCAGTAGTAATACAAGATAATAGTGACATAAAGGTAGTACCAAGGAGGAAAGTAAAAATCATTAAGGACTATGGAAAACAGATGGCAGGTGCTGATTGTGTGGCAGGTAGACAGGATGAAGAT

>AF301156

TGGATAGATAAAATAGAAGCAGCAGAAGAAGACCATCAAAAGTTTCATAGTAATGTGCAGTATCTAAAAGAACAATTTGGGTTACCCACTGTAGTAGCAAAAGAAATTTGGGAAAGATGTTCAGAGTGTCAGAACAAAGGACAAGCTGTTCATGGCCAGCTAGATTATAGCTATGGCTTATGGCAATTAGATTGTACTCATGAGGAAGGAAAGGTTATCTTAGTAGCAGTACACGTCTGTACTCTGTTCTGCTGGGCAACCATATTAAAGAGAGAAACAGGGGAAGAAACAGGTAGGGCCCTCATAAAATTGGCTAGTCAGTGGGAGGTTAGACAAGTTCACACAGATAATGGGCCAAATTTTGTAAGTCAGCACTTTAAGGCAGCAGTGTGGTGGCTAGGAATAGCACACACCACAGGACACCCCTATAATCCTCAATCACAAGGAGTTGTAGAGCAGAGAAACAAGGATGTTAAAAGAAAGATTAAGAAAATGAAAGATCAAGCAGAAACATTAGAAAGCAAAGTAGCAATGGCAGTCTATGCGCTCAATTTTAAAAGAAAGGGAGGATTAGGGGGGAAGAGTCCATGGGAAAGACAAGTAGAAAGAGCAATAATAGAATTAGATACACAAAACCTAACAAAACAAAATCAAAAATTTAAAAATTTTAAGGCCTACTGGAAAGAGCACACAGGAGAG~~~TGGCAAGGACCAGGAGAACTGGTGTGGAAAGGTGAAGGAGCGGTAGTCATCAGAAATTCTCAAACCTTGTTTGTGAAACCTAGAAGAAAAGTAAAGATCACCAGACAATATGGAGAAGATGTGGGCAGTGAGAATTTGTTATCCAATGGGCAAAAGGAA

>AY173951

TTTCTAGATGGAATAGATAAGGCCCAAGAAGAGCATGAAAAATATCACAGTAATTGGAGAGCAATGGCTAGTGATTTTAACCTGCCACCTGTAGTAGCAAAAGAAATAGTAGCCAGCTGTGATAAATGTCAGCTAAAAGGAGAAGCCATGCATGGACAGGTAGACTGTAGTCCAGGAATATGGCAACTAGATTGTACACATTTAGAAGGAAAAGTTATCCTGGTAGCAGTTCATGTAGCCAGTGGATATATAGAAGCAGAAGTTATTCCAGCAGAGACAGGGCAGGAAACAGCATACTTTCTCTTAAAATTAGCAGGAAGATGGCCAGTAAAAACAATACATACAGACAATGGCAGCAATTTCACCAGTACTACGGTTAAGGCTGCCTGTTGGTGGGCGGGGATCAAGCAGGAATTTGGCATCCCCTACAATCCCCAAAGTCAAGGAGTAGTAGAATCTATAAATAAAGAATTAAAGAAAATTATAGGACAGGTAAGAGATCAGGCTGAACATCTTAAAACAGCAGTACAAATGGCAGTATTTATCCACAATTTTAAAAGAAAAGGGGGGATTGGGGGGTACAGTGCAGGGGAAAGAATAGTAGACATAATAGCAACAGACATACAGACTAAAGAATTACAAAAACAAATTACAAAAATTCAAAATTTTCGGGTTTATTACAGGGACAGCAGAGATCCACTTTGGAAAGGACCAGCAAAGCTTCTGTGGAAAGGTGAAGGGGCAGTAGTAATACAAGATAATAGTGACATAAAAGTAGTGCCAAGAAGAAAAGCAAAGATCATTAGGGATTATGGAAAACAGATGGCAGGTGATGATTGTGTGGCAAGTAGACAGGATGAGGAT

>AY173952

TTTTTAGATGGAATAGATAAGGCCCAAGAAGAACATGAGAAATATCACAATAATTGGAGAGCAATGGCTAGTGATTTTAACCTGCCACCTGTAGTAGCAAAAGAAATAGTAGCCAGCTGTGATAAATGCCAGCTAAAAGGAGAAGCCATGCATGGACAAGTAGACTGTAGTCCAGGCATATGGCAACTAGATTGTACACATTTAGAAGGAAAAGTTATCCTGGTAGCAGTTCATGTAGCCAGTGGATATGTAGAAGCAGAAGTTATTCCAGCAGAGACAGGGCAGGAAACAGCATACTTTCTCTTAAAATTAGCAGGAAGATGGCCAGTAAAAACAATACATACAGACAATGGCAGCAATTTCACCAGTGCTACGGTTAAGGCCGCCTGTTGGTGGGCGGGGATCAAGCAGGAATTTGGCATTCCCTACAATCCCCAAAGTCAAGGAGTAGTAGAATCTATAAATAAAGAATTAAAGAAAATTATAGGACAAGTAAGAGATCAGGCTGAACATCTTAGGACAGCAGTACAAATGGCAGTATTCATCCACAATTTTAAAAGAAAAGGGGGGATTGGGGGGTACAGTGCAGGGGAAAGAATAGTAGACATAATAGCAACAGACATACAAACTAAAGAATTACAAAAACAAATTACAAAAATTCAAAATTTTCGGGTTTATTACAGGGACAGCAGAGATCCGCTTTGGAAGGGACCAGCAAAGCTTCTCTGGAAAGGTGAAGGGGCAGTAGTAATACAAGATAATAGTGACATAAAAGTAGTGCCAAGAAGAAAAGCAAAGATCATTAGGGATTATGGAAAACAGATGGCAGGTGATGATTGTGTGGCAAGTAGACAGGATGAGGAT

>AY173953

TTTTTAGATGGAATAGATAAGGCCCAAGACGAACATGAGAAATATCACAGTAATTGGAGAGCAATGGCTAGTGATTTTAACCTACCACCTGTAGTAGCAAAAGAAATAGTAGCCAGCTGTGATAAATGTCAGCTAAAAGGAGAAGCCATGCATGGACAGGTAGACTGTAGTCCAGGAATATGGCAACTAGATTGTACACATTTAGAAGGAAAAGTTATCCTGGTAGCAGTTCATGTAGCCAGTGGATATATAGAAGCAGAAGTCATTCCAGCAGAGACAGGACAGGAAACAGCATACTTTCTCTTAAAATTAGCAGGAAGATGGCCAGTAAAAACAATACACACAGACAATGGCAGCAATTTCACTAGTACTACGGTTAAGGCCGCCTGCTGGTGGGCAGGGATCAAGCAGGAATTTGGCATTCCCTACAATCCCCAAAGTCAAGGAGTAGTAGAATCCATGAATAAAGAATTAAAGAAAATTATAGGACAGGTAAGAGATCAGGCTGAACATCTTAAGACAGCAGTACAAATGGCAGTATTCATCCACAATTTTAAAAGAAAAGGGGGGATTGGGGGGTACAGTGCAGGGGAAAGAATAGTAGACATAATAGCAACAGACATACAAACTAAAGAATTACAAAAACAAATTACAAAAATTCAAAATTTTCGGGTTTATTACAGGGACAGCAGAGATCCACTTTGGAAAGGACCAGCAAAGCTTCTCTGGAAAGGTGAAGGGGCAGTAGTAATACAAGATAATAGTGACATAAAAGTTGTGCCAAGAAGAAAAGTAAAGATCATTAGGGATTATGGAAAACAGATGGCAGGTGATGATTGTGTGGCAAGTAGACAGGATGAGGAT

>AY173954

TTTTTAGATGGAATAGATAAGGCCCAAGAAGAACATGAAAAATATCACAGTAATTGGAGAGCTATGGCTAGTGACTTTAACCTGCCACCTATAGTAGCAAAAGAAATAGTAGCCAGCTGTGATAAATGTCAGCTGAAAGGAGAAGCCATGCATGGACAAGTAGACTGTAGTCCAGGAATATGGCAGCTAGATTGTACACATTTAGAAGGAAAAGTTATCCTGGTAGCAGTTCATGTAGCCAGTGGATATATAGAAGCAGAAGTTATTCCAGCAGAGACAGGGCAAGAAACAGCATACTTTATCTTAAAATTAGCAGGAAGGTGGCCAGTAAAAACAATACATACAGACAATGGTGGCAATTTCATCAGTAATACAGTTAAGGCCGCCTGTTGGTGGGCGGGGATCAAGCAAGAATTTGGCATTCCCTACAATCCCCAAAGTCAAGGAGTAGTAGAATCTATGAATAAAGAATTAAAGAAAATTATAGGACAGGTAAGAGATCAGGCTGAACATCTTAAGACAGCAGTACAAATGGCAGTATTCATCCACAATTTTAAAAGAAAAGGGGGGATTGGGGGGTACAGTGCAGGGGAAAGAATAGTAGACATAATAGCAACAGACATACAAACTAAAGAATTACAAAAACAAATTACAAAGATTCAAAATTTTCGGGTTTATTACAGGGACAGCAGAGATCCACTTTGGAAAGGACCAGCAAAGCTTCTCTGGAAAGGTGAAGGGGCAGTAGTAATACAAGATAATAGTGACATAAAAGTAGTGCCAAGAAGAAAAGTAAAGATCATTAGGGATTATGGAAAACAGATGGCAGGTGATGATTGTGTGGCAAGTAGACAGGATGAGGAT

>AY173955

TTTTTGGATGGAATAGATAAGGCTCAAGAAGAACATGAAAAATATCACAGTAATTGGAGAGCAATGGCTAGTGATTTTAACCTGCCACCTGTAGTAGCAAAAGAAATAGTAGCCAGCTGTGATAAATGTCAGCTAAAAGGAGAAGCCATGCATGGACAAGTAGATTGTAGTCCAGGAATATGGCAACTAGATTGTACACATCTAGAAGGAAAGATTATCCTGGTAGCAGTTCATGTAGCCAGTGGCTATATAGAAGCAGAAGTTATTCCAGCAGAGACAGGGCAGGAAACAGCATACTTTATCTTAAAATTAGCAGGAAGATGGCCAGTAAAAACAATACATACAGACAATGGCAGCAATTTCATCAGTAACACAGTTAAGGCCGCCTGCTGGTGGGCAGGAATCAAGCAGGAATTTGGCATTCCCTACAATCCCCAAAGTCAAGGAGTAGTAGAATCTATGAATAAAGAATTAAAGAAAATTATAGGACAGGTAAGGGATCAGGCTGAACATCTTAAGACAGCAGTACAAATGGCAGTATTCATTCACAATTTTAAAAGAAAAGGGGGGATTGGGGGATATAGTGCAGGGGAAAGAATAGTAGACATAATAGCAACAGACATACAAACTAAAGAATTACAAAAACAAATTACAAAAATTCAAAATTTTCGGGTTTATTACAGGGACAGCAGAGATCCACTTTGGAAAGGACCAGCAAAGCTTCTCTGGAAAGGTGAAGGGGCAGTAGTAATACAAGATAATAGTGACATAAAAGTAGTGCCAAGAAGAAAAGTAAAAATCATTAGGGATTATGGAAAACAGATGGCAGGTGATGATTGTGTGGCAAGTAGACAGGATGAGGAT

>AY173956

TTTTTAGATGGAATAGATAAGGCCCAAGAGGAACATGAGAAATATCACAGTAATTGGAGAGCAATGGCTAGTGATTTTAACCTGCCACCTGTAGTAGCAAAAGAAATAGTAGCCAGCTGTGACAAATGTCAGCTAAAAGGAGAAGCTATGCATGGACAAGTAGACTGTAGTCCAGGAATATGGCARCTAGATTGTACACATTTAGAAGGAAAAATTATCCTGGTAGCAGTTCATGTAGCCAGTGGATATATAGAAGCAGAAGTTATTCCAGCAGAGACAGGGCAGGAAACAGCATACTTTATCTTAAAATTAGCAGGAAGATGGCCAGTAAAAACAATACATACAGACAATGGCAGCAATTTCACCAGTACTGCGGTTAAGGCCGCCTGTTGGTGGGCGGGGGTCAAGCAGGAATTTGGCATTCCCTACAATCCCCAAAGTCAAGGAGTAGTAGAATCTATGAATAAGGAATTAAAGAAAATTATAGGACAGGTAAGAGASCAGGCTGAACATCTTAAGACAGCAGTACAAATGGCAGTATTCATCCACAATTTTAAAAGAAAAGGGGGGATTGGGAGGTACAGTGCAGGGGAAAGAATAATAGACATAATAGCAACAGACTTACAAACTACAGAATTACAAAAACAAATTACAAAAATTCAAAATTTTCGGGTTTATTACAGAGACAGCAGAGACCCACTTTGGAAAGGACCAGCAAAGCTTCTCTGGAAAGGTGAAGGGGCAGTAGTAATACAAGATAATAGTGACATAAAAGTAGTGCCAAGAAGAAAAGTAAAGATCATTAGGGATTATGGAAAACAGATGGCAGGTGATGATTGTGTGGCAGGTAGACAGGATGAGGAT

>AY173957

TTTTTAGATGGGATAGATAAAGCACAGGAGGAACATGAAAAATATCACAACAATTGGAGAGCAATGGCTAGTGATTTTAATCTGCCACCTGTAGTAGCAAAAGAAATAGTAGCTAGCTGTGATAAGTGTCAGCTAAAAGGGGAAGCCATGCATGGACAAGTAGATTGTAGTCCAGGGATATGGCAATTAGATTGTACACATTTAGAAGGAAAAGTTATTCTAGTAGCAGTCCATGTAGCTAGTGGGTACCTAGAAGCAGAAGTKATCCCAACAGAAACAGGGCAAGAAACAGCCTACTTCATACTAAAGTTAGCAGGAAGATGGCCAGTAAAAACAATACATACAGACAATGGCACTAATTTCACCAGTGCCGCGGTTAAGGCAGCCTGTTGGTGGGCAGGTATCCAGCAGGAGTTTGGAATTCCCTACAACCCCCAAAGTCAAGGAGTAGTAGAATCTATGAATAAAGAGCTAAAGAAGATCATAAGACAGGTAAGAGATCAAGCTGAACATCTTAAGACAGCAGTACAAATGGCAGTATTCATCCACAATTTTAAAAGAAAAGGGGGGATTGGGGGATACAGTGCAGGGGAAAGAATAATAGACATAATATCATCAGACATACAAACTAAAGAATTACAAAAACAAATTATAAAAATTCGAAATTTCCGGGTTTATTACAGGGACAGCAGAGACCCAGTTTGGAAAGGACCAGCAAAGCTACTCTGGAAAGGTGAAGGGGCAGTAGTCATACAAGACAATAGTGAAATAAAGGTAGTACCAAGAAGAAAAGCAAAGATCATTAGGGATTATGGAAAACAGATGGCAGGTGATGATTGTGTGGCAGGTAGACAGGATGAGGAT

>AY173958

TTTTTAGATGGGATAGATAAAGCACAGGAGGAACATGAAAAATATCACAACAATTGGAGAGCAATGGCTAGTGATTTTAATCTGCCACCTGTAGTAGCAAAAGAAATAGTAGCTAGCTGTGATAAGTGTCAGCTAAAAGGGGAAGCCATGCATGGACAAGTAGATTG~AGTCCAGGGATATGGCAATTAGATTGTACACATTTAGAAGGAAAAGTTATTCTGGTAGCAGTCCATGTAGCTAGTGGGTACCTAGAAGCAGAAGTTATCCCAACAGAAACAGGACAAGAAACAGCCTACTTCATACTAAAGTTAGCAGGAAGATGGCCAGTAAAAACAATACATACAGACAATGGCACCAATTTCACCAGTGCCGCGGTTAAGGCAGCCTGTTGGTGGGCAGGTATCCAACAGGAATTTGGAATTCCCTACAACCCCCAAAGTCAAGGAGTAGTAGAATCTATGAATAAAGAGCTAAAGAAGATTATAAAACAGGTAAGAGATCAAGCTGAACATCTTAAGACAGCAGTACAAATGGCAGTATTCATCCACAATTTTAAAAGAAAAGGGGGGATTGGGGGATACAGTGCAGGGGAAAGAATAATAGACATAATATCATCAGACATACAAACTAAAGAATTACAAAAACAAATTATAAAAATTCGAAATTTCCGGGTTTATTACAGGGACAGCAGAGACCCAGTTTGGAAAGGACCAGCAAAGCTACTCTGGAAAGGTGAAGGGGCAGTAGTCATACAAGACAATAGTGAAATAAAGGTAGTACCAAGAAGAAAAGCAAAGATCATTAGGGATTATGGAAAACAGATGGCAGGTGATGATTGTGTGGCAGGTAGACAGGATGAGGAT

>AY173959

TTTTTAGATGGAATAGACAAGGCACAAGAAGAGCATGAGAAATATCATAATAATTGGAGAGCAATGGCTAGTGATTTTAACCTGCCACCTGTAGTAGCAAAAGAAATAGTAGCCAGCTGTGATAAATGTCAGCTAAAAGGAGAAGCCATACATGGACAAGTAGACTGTAGTCCAGGAATATGGCAACTAGATTGTACACATTTAGAAGGAAAAATTATTCTGGTAGCAGCTCATGTAGCCAGTGGTTATATAGAAGCAGAAGTCATCCCAGCAGAGACAGGGCAGGAAACAGCATACTTTATCTTAAAATTAGCAGGAAGATGGCCAGTAAAAACAATACATACAGACAATGGCAGCAATTTCACCAGTGCTGCGGTTAAGGCCGCCTGTTGGTGGGCAGGAATCAAGCAGGATTTTGGCATTCCCTACAATCCCCAAAGTCAAGGAGTAGTAGAATCTATGAATAAAGAATTAAAGAAAATTATAGGACAAGTAAGAGATCAGGCTGAACATCTTAAGACAGCAGTACAAATGGCAGTATTCATCCACAATTTTAAAAGAAAAGGGGGGATTGGGGGGTACAGTGCAGGAGAAAGAATAATAGACATAATAGCAACAGACATACAAACTAAAGAATTACAAAATCAAATTACAAAACTTCAAAATTTTCGGGTTTATTACAGGGACAGCAGAGATCCACTTTGGAAAGGACCAGCAAAGCTTCTCTGGAAAGGTGAAGGGGCAGTAGTAATACAAGATAACAGTGACATAAAAATAGTGCCAAGAAGAAAAGCAAAGATCATTAGGGATTATGGAAAACAAATGGCAGGTGATGATTGTGTGGCAAGTAGACAGGATGAGGAT

>AY173960

TTTTTAGATGGAATAGATAAGGCCCAAGAAGAACATGAAAAATATCACAATAATTGGAGAGCAATGGCTAGTGATTTTAACATACCAGCTGTGGTAGCAAAAGAAATAGTAGCCAGCTGTGATAAATGTCAGCTAAAAGGAGAGGCCATGCATGGACAAGTAGACTGTAGTCCAGGAATATGGCAGCTAGATTGTACACACTTAGAAGGAAAAATTATCCTGGTAGCAGTTCATGTAGCCAGTGGATATATAGAAGCAGAAGTTATTCCAGCAGAGACAGGGCAAGAAACAGCATACTTTCTCTTAAAATTAGCAGCAAGATGGCCAGTAAAAACAATACATACAGACAATGGCAGCAACTTCACCAGTACCACAGTTAAGGCCGCCTGTTGGTGGGCGGGGATCAAGCAGGAATTTGGCATTCCCTACAATCCCCAAAGTCAAGGGGTAGTAGAATCTATGAACAAAGAATTAAAGAAAATTATAGGACAGGTAAGAGATCAGGCTGAACATCTTAAGACAGCAGTACAAATGGCAGTATTCATCCACAATTTTAAAAGAAAAGGGGGGATTGGGGGGTACAGTGCAGGGGAAAGAATAATAGACATAATAGCAACAGACATACAAACTAAAGAACTACAAAAACAAATTACAAAAATTCAAAATTTTCGGGTTTATTACAGGGACAGCAGAGACCCAATTTGGAAAGGACCAGCAAAGCTTCTCTGGAAAGGTGAAGGGGCAGTAGTAATACAAGAAAATAGTGAAATAAAAGTAGTGCCAAGAAGAAAAGCAAAAATCATCAGGGATTATGGAAAACAGATGGCAGGTGATGATTGTGTGGCAAGTAGACAGGATGAGGAT

>AF544009

TTTCTAGGTGGAATAGATAAGGCTCAAGAAGAGCATGAAAAGTATCACAGCAATTGGAGAGCAATGGCTAGTGAGTTTGATCTGCCACCCGTAGTAGCAAGAGAAATAGTAGCCAGCTGTGATAAATGTCAGCTAAAAGGGGAAGCCATACATGGACAAGTAGATTGTAGTCCGGGGATATGGCAATTAGATTGTACGCATTTAGAAGGAAAAATCATCCTGGTAGCAGTCCATGTAGCCAGTGGCTACATAGAAGCAGAGGTTATCCCAGCAGAAACAGGACAAGAAACAGCATACTATATACTAAAATTAGCAGGAAGATGGCCAGTCAAAGTAATACATACAGACAATGGCAGTAATTTCACCAGTGCTGCAGTTAAGGCAGCCTGTTGGTGGGCAGGTATCCAACAGGAATTTGGGATTCCCTACAATCCCCAAAGTCAGGGAGTAGTAGAATCCATGAATAAAGAATTAAAGAAAATCATAGGGCAGGTAAGAGATCAAGCTGAGCACCTTAAGACAGCAGTACAAATGGCAGTATTCATTCACAATTTTAAAAGAAAAGGGGGGATTGGGGGGTACAGTGCAGGGGAAAGAATAATAGACATAATAGCAACAGACATACAAACTAAAGAATTACAAAAACAAATTATAAAAATTCAAAATTTTCGGGTTTATTACAGAGACAGCAGAGATCCTATTTGGAAAGGACCAGCCAAGCTACTCTGGAAAGGTGAAGGGGCAGTAGTAATACAAGACAACAGTGACATAAAGGTAGTACCAAGGAGGAAAGTAAAAATCATTAGGGACTATGGAAAACAGATGGCAGGTGCTGATTGTGTGGCAGGTAGACAGGATGAAGAT

>AB097873

TTTTTAGATGGAATAGATAAGGCCCAGGAAGAACATGAGAAATATCATTGTAACTGGAGAGCAATGGCTAGTGATTTTAACCTACCACCTGTAGTAGCAAAAGAAATAGTAGCCAGCTGTGATAAATGTCAGCTAAAAGGAGAAGCCATGCATGGACAAGTAGACTGTAGCCCAGGAATATGGCAACTAGATTGTACACATTTAGAAGGAAAAATTATCCTGGTAGCAGTTCATGTAGCCAGTGGATATATAGAAGCAGAAGTTATTCCAACAGAGACAGGGCAGGAAACAGCATACTTTATCCTAAAATTAGCAGGAAGATGGCCAGTGAGAACAATACATACAGACAATGGCAGAAATTTCACCAGTAATGCGGTTAAGGCCGCCTGTTGGTGGGCAGGGATCAAGCAGGAATTTGGCATTCCCTACAATCCCCAAAGTCAAGGAGTAGTAGAATCTATGAATAAAGAATTAAAGAAAATAATAGAACAGGTAAGAGATCAGGCTGAACATCTTAAGACAGCAGTACAAATGGCAGTATTCATCCACAATTTTAAAAGAAAAGGGGGGATTGGGGGGTACAGTGCAGGGGAAAGGATAGTAGACATAATAGCAACAGACATACAAACTAAAGAATTACAAAAACAAATTACAAAAATTCAAAATTTTCGGGTTTATTACAGGGACAGCAGAGATCCACTTTGGAAAGGACCAGCAAAGCTCCTTTGGAAAGGTGAAGGGGCAGTAGTAATACAAGATAATAGTGACATAAAAGTAGTGCCAAGAAGAAAAGCAAAGATCATTAGGGACTATGGAAAACAGATGGCAGGTGATGATTGTGTGGCAAGTAGACAGGATGAGGAT

>AB097872

TTTTTAGATGGGATAGATAAGGCTCAAGAAGACCATGAAAAATATCACAGCAATTGGAGAACAATGGCTAGTGAGTTTAATTTGCCACCTATAGTAGCAAAGGAAATAGTAGCTAACTGTGATAAATGTCAGCTAAAAGGGGAAGCTATGCATGGACAAGTAGACTGTAGTCCAGGGATATGGCAATTAGATTGCACACATCTAGAAGGAAAAGTCATCCTGGTAGCAGTCCATGTGGCCAGTGGATATATAGAAGCAGAAGTTATCCCAGCAGAAACAGGACAGGAGACAGCATACTTTCTGCTAAAATTAGCAGGAAGATGGCCAGTAAAGGTAATACACACAGACAACGGTAGCAATTTCACCAGCGCTGCAGTTAAAGCAGCCTGTTGGTGGGCCAATGTCCAACAGGAATTTGGGATCCCCTACAATCCCCAAAGTCAAGGAGTAGTAGAATCTATGAATAAGGAATTAAAGAAAATCATAGGGCAGGTAAGAGAGCAAGCTGAACACCTTAAGACAGCAGTACAAATGGCAGTATTCATTCACAATTTTAAAAGAAAAGGGGGGATTGGGGGGTACAGTGCAGGGGAAAGAATAATAGACATAATAGCAACAGACATACAAACTAAAGAATTACAAAAACAAATTACAAAAATTCAAAATTTTCGGGTTTATTACAGGGACAGCAGAGATCCAATTTGGAAAGGACCAGCAAAACTACTCTGGAAAGGTGAAGGGGCAGTAGTAATACAAGACAATAGTGATATAAAAGTAGTACCAAGAAGAAAAGCAAAGATCATTAGGGATTATGGAAAACAGATGGCAGGTGATGATTGTGTGGCAGGTAGACAGGATGAGGAT

>AB097871

TTCCTAGATGGAATAGATAAAGCTCAAGAAGAGCATGAAAAGTATCACAGCAATTGGAGAGCAATGGCTAGTGACTTTAATCTGCCACCCATAGTAGCAAAAGAAATAGTAGCTAGCTGTGATCAATGTCAGCTAAAAGGGGAAGCCATGCATGGACAAGTAGACTGTAGTCCAGGGATATGGCAATTAGATTGTACACATTTAGAAGGAAAAATCATCCTGGTAGCAGTCCATGTAGCCAGTGGCTACATGGAAGCAGAGGTTATCCCAGCAGAAACAGGACAAGAGACAGCATACTTTATACTAAAATTAGCAGGAAGATGGCCAGTCAAAGTAATACATACAGACAATGGTAGTAATTTCACCAGTGCTGCAGCTAAGGCAGCCTGTTGGTGGGCAGGTATCCACCAGGAATTTGGAATTCCCTACAATCCCCAAAGTCAGGGAGTAGTAGAATCCATGAATAAAGAATTAAAGAGAATCATAGGGCAGGTAAGAGATCAAGCTGAGCACCTTAAGACAGCAGTACAAATGGCAGTATTCATTCACAATTTTAAAAGAAAAGGGGGGATTGGGGGGTACAGTGCAGGGGAAAGAATAATAGACATAATAGCAACAGACATACAAACTAAAGAATTACAAAAACAAATTATAAAAATTCAAAATTTTCGGGTTTATTACAGAGACAGCAGAGACCCCATTTGGAAAGGACCAGCCAAACTACTCTGGAAAGGTGAAGGGGCAGTAGTAATACAAGATAATAGTGACATAAAGGTAGTACCAAGGAGGAAAGCAAAAATCATTAAGGACTATGGAAAACAGATGGCAGGTGCTGATTGTGTGGCAGGTAGACAGGATGAAGAT

>AB097870

TTTTTAGATGGAATAGAAAAGGCCCAAGAAGATCATGAGAAATATCACTGTAATTGGAGAGCAATGGCTAGTGATTTTAACCTACCACCTATAGTAGCAAAAGAAATAGTAGCCAGCTGTGATAAATGTCAGTTAAAAGGAGAAGCCATGCATGGACAAGTAGACTGTAGCCCAGGAATATGGCAACTAGACTGTACACATTTAGAAGGAAAAATTATCCTGGTAGCAGTTCATGTAGCCAGTGGATATATAGAAGCAGAAGTTATTCCAACAGAGACAGGGCAGGAAACAGCATACTTTATCCTAAAGTTAGCAGGAAGATGGCCAGTAAAAACAATACATACAGACAATGGCAGAAATTTCACCAGTAATTCAGTTAAGGCTGCCTGTTGGTGGGCAGGGATCAAGCAGGAATTTGGCATTCCCTACAATCCCCAAAGTCAAGGAGTAGTAGAATCTATGAATAAAGAATTAAAGAAAATTGTAGAACAGGTAAGAGATCAAGCTGAACATCTTGAGACAGCAGTACAAATGGCAGTATTCATCCACAATTTTAAAAGAGAAGGGGGGATTGGAGGGTACAGTGCAGGGGAAAGAATAGTAGACATAATAGCAACAGACATACAAACTAGAGAACTACAAAAACAAATTACAAAAATTCAAAATTTTCGGGTTTATTACAGGGACAACAGAGATCCACTTTGGAAAGGACCAGCAAAGCTCCTTTGGAAAGGTGAAGGGGCAGTAGTAATACAAGATAATAGTGACATAAAAGTAGTGCCAAGAAGAAAAGCAAAGATCATTAGGGACTATGGAAAACAGATGGCAGGTGATGATTGTGTGGCAAGTAGACAGGATGAGGAT

>AB097869

TTTTTAGATGGAATAGATAAGGCCCAAGATGAGCATGAAAAATATCACAGTAACTGGAGAGCAATGGCTAGTGATTTTAACCTACCACCTGTAGTAGCAAAAGAAATAGTAGCCAGCTGTGATAAATGTCAACTAAAAGGAGAAGCCATGCAGGGGCAAGTAGACTGTAGCCCAGGAATATGGCAACTAGATTGTACACACTTAGAAGGAAAAATTATCCTGGTAGCAGTTCATGTAGCCAGTGGCTACATAGAAGCAGAGATAATCCCAGCAGAAACAGGACAAGAAACAGCATACTTTATACTAAAATTAGCAGCAAGATGGCCAGTCAGAGTAATACATACAGACAATGGTAGTAATTTCACCAGTACTGCAGTTAAGGCAGCCTGTTGGTGGGCAGGTATCAAGCAGGAATTTGGCATTCCCTACAATCCCCAAAGTCAAGGAGTAGTAGAATCTATGAATAAACAATTAAAACAAATTATAGGACAGGTAAGAGATCAAGCTGAACATCTTAAGACAGCAGTACAAATGGCAGTATTCATCCACAATTTTAAGAGAAAAGGGGGGATTGGGGGGTACAGTGCAGGGGAAAGAATAGTAGACATAATAGCAACAGACATACAAACTAGAGAATTACAAAAACAAATTACAAAAATTCAAAATTTTCGGGTTTATTACAGGGACAACAAAGATCCACTTTGGAAAGGACCAGCAAAGCTCCTTTGGAAAGGTGAAGGGGCAGTAGTAATACAAGATAATAGTGACATAAAAGTAGTGCCAAGAAGAAAAGCAAAGATCATTAGGGACTATGGAAAACAGATGGCAGGTGATGATTGTGTGGCAAGTAGACAGGATGAGGAT

>AB097868

TTTCTAGATGGAATTGATAAAGCTCAAGAAGAGCATGAAAGGTATCACAGCAATTGGAGAGCAATGGCTAGTGACTTTAATCTGCCACCCATAGTAGCAAAAGAAATAGTGGCTAGCTGTGATCAATGTCAACTAAAAGGAGAAGCCATGCATGGACAAGTAGACTGTAGTCCAGGGATATGGCAATTAGATTGTACACATTTAGAAGGAAAAATCATCCTGGTAGCAATCCATGTAGCCAGCGGCTACATGGAAGCAGAGGTTATCCCAGCAGAAACAGGACAAGAGACAGCATACTTTATATTAAAATTAGCAGGAAGATGGCCAGTCAAAGTAATACATACAGATAATGGTAGTAATTTCACCAGTGCTGCAGTTAAGGCAGCCTGTTGGTGGGCAGGTATCCAACAGGAGTTTGGAATTCCCTACAATCCCCAAAGTCAGGGAGTAGTAGAATCCATGAATAAAGAATTAAAGAAAATTATAGGGCAGGTAAGAGATCAAGCTGAGCACCTTAAGACAGCAGTACAAATGGCAGTATTCATTCACAATTTTAAAAGAAAAGGGGGGATTGGGGGGTACAGTGCAGGGGAAAGAATAATAGACATAATAGCAACAGACATACAAACTAAAGAATTACAAAAACAAATTACAAAAATTCAAAATTTTCGGGTTTATTACAGAGACAGCAGAGACCCCATTTGGAAAGGACCAGCCAAACTACTCTGGAAAGGTGAAGGGGCAGTAGTAATACAAGATAATAGTGACATAAAGGTAGTACCAAGGAGAAAAGCAAAAATCATTAAGGACTATGGAAAACAGGTGGCAGGTGCTGATTGTGTGGCAGGTAGACAGGATGAAGAT

>AB097867

TTTTTAGATGGGATAGATAAGGCTCAAGAAGAACATGAAAGATATCACAGCAATTGGAGAACAATGGCTAGTGATTTTAATTTGCCACCTATAGTAGCAAAGGAAATAGTAGCCAATTGTGATAAATGCCAACTAAAAGGGGAAGCTATGCATGGACAAGTAGACTGTAGTCCAGGGATATGGCAATTAGATTGCACACATCTAGAAGGAAAAGTCATCCTGGTAGCAGTCCACGTGGCCAGTGGATATATAGAAGCAGAAGTTATCCCAGCAGAAACAGGACAGGAGACAGCATACTTTCTGCTAAAATTAGCAGGAAGATGGCCAGTAAAAGTAATACACACAGACAACGGTAGCAATTTCACCAGCGCTGCAGTTAAAGCAGCCTGTTGGTGGGCCAATGTCCGACAGGAATTTGGGATTCCCTACAATCCCCAAAGTCAAGGAGTAGTAGAATCTATGAATAAGGAATTAAAGAAAATCATAGGGCAGGTAAGAGAGCAAGCTGAACACCTTAAGACAGCAGTACAAATGGCAGTATTCATTCACAATTTTAAAAGAAAAGGGGGGATTGGGGGGTACAGTGCAGGGGAAAGAATAATAGACATAATAGCAACAGACATACAAACTAAAGAATTACAAAAACAAATTACAAAAATTCAAAATTTTCGGGTTTATTACAGGGACAGCAGAGACCCAATTTGGAAAGGACCAGCAAAACTACTCTGGAAAGGTGAAGGGGCAGTAGTAATACAAGACAATAGTGATATAAAAGTAGTGCCAAGAAGAAAAGCAAAGATCATTAGGGATTATGGAAAACAGATGGCAGGTGATGATTGTGTGGCAGGTAGACAGGATGAGGAT

>AB097866

TTTTTGGATGGAATAGATAAGGCTCAAGAAGACCATGAAAGATATCACAGCAATTGGAGAACAATGGCTAGTGATTTTAACCTACCACCTGTAGTAGCAAAAGAAATAGTAGCCAGCTGTGATAAATGTCAGCTAAAAGGAGAAGCCATGCATGGACAAGTAGACTGTAGTCCAGGAATATGGCAATTAGATTGTACACATCTAGAAGGAAAAGTCATCCTGGTAGCAGTCCACGTGGCCAGTGGCTACATAGAAGCAGAGGTTATCCCAGCAGAAACAGGACAAGAGACAGCATACTTTATACTAAAATTAGCAGGAAGATGGCCAGTCAAAGTAATACATACAGACAATGGTAGTAATTTCATCAGTGCTGCAGTTAGGGCAGCCTGTTGGTGGGCAGGTATCCAACAGGAATTTGGAATTCCCTACAATCCCCAAAGTCAGGGAGTAGTAGAGTCCATGAATAAAGAATTAAAGAAAATCATAGGACAGGTAAGAGAGCAAGCTGAGCACCTTAAGACAGCAGTACAAATGGCAGTATTCATTCACAATTTTAAAAGAAAAGGGGGGATTGGGGGGTACAGTGCAGGGGAAAGAATAATAGACATCATAGCAACAGACATGCAAACTAAAGAATTACAAAAACAAATTATAAAAATTCAAAATTTTCGGGTTTATTACAGGGACAGCAGAGATCCAATTTGGAAAGGACCAGCAAAACTACTCTGGAAAGGTGAAGGGGCAGTAGTAATACAAGACAATAGTGATATAAAAGTAGTACCAAGAAGAAAAGCAAAGATCATTAGGGATTATGGAAAACAGATGGCAGGTGATGATTGTGTGGCAGGTAGACAGGATGAGGAT

>AB097865

TTTTTAGATGGAATA~ATAAGGCCCAAGAAGAACATGAGAAATATCACAATAATTGGAGAGCAATGGCTAGTGATTTTAACCTACCACCTGTAGTAGCAAAAGGAATAGTAGCCAGCTGTGATAAATGTCAGCTAAAAGGAGAAGCCATGCATGGACAAGTAGACTGTAGCCCAGGAATATGGCAACTAGATTGTACACATCTAGAAGGAAAAATTATCCTGGTAGCAGTTCATGTAGCCAGTGGATATATAGAAGCAGAAGTTATTCCAGCAGAGACAGGGCAGGAAACAGCATACTTTCTCCTAAAACTAGCAGGAAGATGGCCAGTGAAAATAATACATACAGACAATGGCAGAAATTTCACCAGTAATTCGGTTAAGGCCGCCTGTTGGTGGGCGGGGATCAAGCAGGAATTTGGCATTCCCTACAATCCCCAAAGTCAAGGAGTAGTAGAATCCATGAATAAAGAATTAAAGAAAATTATAGAACAGGTAAGAGATCAAGCTGAACATCTTAAGACAGCAGTACAAATGGCAGTTTTCATCCACAATTTTAAGAGAAAAGGGGGGATTGGGGGGTACAGTGCAGGGGAAAGAATAGTAGACATAATAGCAACAGACATACAAACTAGAGAATTACAAAAACAAATTACAAAAATTCAAAATTTTCGGGTTTATTACAGGGACAGCAGAGATCCACTTTGGAAAGGACCAGCAAAGCTCCTTTGGAAAGGTGAAGGGGCAGTAGTAATACAAGATAATAGTGACATAAAAGTAGTGCCAAGAAGAAAAGCAAAGATCATTAGGGACTATGGAAAACAGATGGCAGGTGATGATTGTGTGGCAAGTAGGCAGGATGAGGAT

>AF382822

TTCCTGGATGGAATTAATGAAGCACAGGAAGACCATGATAAATATCACAGTAATTGGAAAGCTTTAGCTGATGAATATAATCTGCCCCCAGTTGTGGCTAAAGAAATTATTGCTCAGTGTCCAAAATGCCATATAAAAGGAGAGGCTATACATGGACAGGTGGACTACAGTCCAGAAATCTGGCAAATAGACTGTACCCACCTAGAAGGAAAGGTCATCATAGTAGCAGTGCATGTAGCTAGTGGTTTCATAGAAGCAGAAGTCATACCAGAAGAAACAGGAAGAGAAACCGCTTACTTCATCCTAAAATTGGCAGGAAGATGGCCTGTAAAGAAAATACATACAGATAATGGACCAAATTTTACTAGTACAGCAGTGAAGGCAGCCTGCTGGTGGGCACAAATTCAACATGAATTTGGGATTCCATATAATCCTCAAAGTCAAGGAGTAGTAGAATCTATGAATAAACAATTAAAGCAAATTATAGAGCAAGTCAGGGACCAAGCAGAGCAACTGAGGACAGCAGTAATCATGGCAGTGTATATCCACAATTTTAAAAGAAAAGGGGGGATTGGGGAGTACACTGCAGGGGAAAGACTATTAGACATACTAACTACAAATATACAGACAAAACAATTACAAAAACAAATTTTAAAAGTTCAAAATTTTCGGGTTTATTATAGGGACGCCAGAGATCCAATTTGGAAGGGACCAGCGCGACTACTGTGGAAAGGTGAAGGGGCAGTAGTAATAAAAGAAGGAGAAGACATTAAAGTAGTACCCAGGAGAAAAGCAAAAATCATAAAAGAGTATGGAAAACAGATGGCAGGTGCAGGTGGTATGGATGATAGACAGAATGAG

>AF334679

TTCCTAGAAAGTATAGAGCCAGCACAAGAAGACCATGACAAATATCATAGCAATGTAAAGGAGTTAGCTCAGAAATATAACATCCCACAATTAGTAGCTAAACAGATAGTAAATGCTTGTAACAAATGCCAACAGAAGGGAGAAGCGATACATGGTCAGACAAATGCAGAGGTAGGCACATGGCAGATGGACTGCACCCACTTAGAAGGAAAGGTTATCATAGTAGCAGTACATGTGGCTAGTGGCTTTATAGAAGCAGAGGTAATACCTAGGGAAACAGGAAGACAGACAGCATTGTTCCTCTTAAAAATAGCATCAAGATGGCCTATAAAGCATCTGCATACAGACAACGGTGCCAATTTCACCTCCCAGGAGGTGAAGATGGTAGCATGGTGGTTAGGAGTGGAACAGTCCTTTGGAGTACCGTACAACCCACAAAGTCAGGGAGTAGTAGAAGCCATGGACTTACATCTTAAGAAAAACATAGATAAAATAAGGGAACAAGCAGAGTCAGTAGAGACCCTAGTACTAATGGCAGCACATTGCATGAATTTTAAAAGAAGGGGAGGAATAGGGGATATGACTCCTGCAGAAAGAATAGTAAATATGATCACCACAGAACTAGAAACCCAATACTTAAATTCACAAAATTCAAAATTTCAAAATTTTCGGGTTTATTACAGAGAAGGAAGAGACCAACTGTGGAAGGGACCAGCAGAGCTCCTCTGGAAAGGAGAAGGGGCTGTTGTAATTAAGGTAGGGACAGAAATAAAAGTGGTACCAAGAAGAAAAGCAAAAATCATCAAAGATTATGGAAAGGCAAGAGATGGGTAGCAGTGCCAGTATGGAGGATCAGTAGAAGAA

>AY358036

TTTTTAGATGGAATAGATAAGGCTCAAGAAGAACATGAAAGATATCACAGCAATTGGAGAACAATGGCTAGTGATTTTAATTTGCCACCTGTGGTAGCAAAAGAAATAGTAGCCAACTGTGATAAATGTCAATTAAAAGGGGAAGCTATGCATGGACAAGTAGACTGTAGTCCAGGGATATGGCAATTAGATTGCACACATCTAGAAGGAAAAGTCATCCTGGTAGCAGTCCACGTGGCCAGTGGATATATAGAAGCAGAAGTTATCCCAGCAGAAACAGGACAAGAGACAGCATACTTTCTGCTAAAATTAGCAGGAAGATGGCCAGTAAAAGTAATACACACAGACAATGGTAGCAATTTCACCAGTGCTGCAGTTAAGGCAGCCTGTTGGTGGGCCAATGTCCGACAGGAATTTGGGATCCCCTACAATCCTCAAAGTCAAGGAGTAGTAGAATCTATGAATAAGGAATTAAAGAAAATCATAGGGCAGATAAGAGAGCAAGCTGAACACCTTAAGACAGCAGTACAAATGGCAGTATTCATTCACAATTTTAAAAGAAAAGGGGGGATTGGGGGGTACAGTGCAGGGGAAAGAATAATAGACATAATAGCAACAGACATACAAACTAAAGAATTACAAAAACAAATTACAAAAATTCAAAATTTTCGGGTTTATTACAGGGACAGCAGAGACCCAATTTGGAAAGGACCAGCAAAACTACTCTGGAAAGGTGAAGGGGCAGTAGTAATACAAGACAATAGTGATATAAAAGTAATACCAAGAAGAAAAGCAAAAATCATTAGGGATTATGGAAAACAGATGGCAGGTGATGATTGTGTGGCAGGTAGACAGGATGAGGAT

>AY358037

TTTTTAGATGGGATAGATAAGGCTCAAGAAGAACATGAAAGATATCACAGCAATTGGAGAACAATGGCTAGTGATTTTAATTTACCACCTATAGTAGCAAAGGAAATAGTAGCCAACTGTGATAAATGTCAGCTAAAAGGGGAAGCTATGCATGGACAAGTGGACTGTAGTCCAGGGATATGGCAATTAGATTGCACACATCTAGAAGGAAAAGTCATCCTGGTAGCAGTCCACGTGGCTAGTGGATATATAGAAGCAGAAGTTATCCCAGCAGAAACAGGACAGGAGACAGCATACTTTCTGCTAAAATTAGCAGGAAGATGGCCAGTAAAAGTAATACACACAGACAATGGTAGCAATTTCACCAGCGCTGCAGTTAAAGCAGCCTGTTGGTGGGCCAATGTCAAACAGGAATTTGGGATCCCCTACAATCCCCAAAGTCAAGGAGTAGTAGAATCTATGAATAAGGAATTAAAGAAAATCATAGGGCAGGTAAGAGAGCAAGCTGAACACCTTAAGACAGCAGTACAAATGGCAGTATTCATTCACAATTTTAAAAGAAAAGGGGGGATTGGGGGGTACAGTGCAGGGGAAAGAATAATAGACATAATAGCAACAGACATACAAACTAAAGAATTACAAAAACAAATTACAAAAATTCAAAATTTTCGGGTTTATTACAGGGACAGCAGAGACCCAATTTGGAAAGGACCAGCAAAACTACTCTGGAAAGGTGAAGGGGCAGTAGTAATACAAGACAATAGTGATATCAAAGTAGTACCAAGAAGAAAAGCAAAGATCATCAGGGATTATGGAAAACAGATGGCAGGTGATGATTGTGTGGCAGGTAGACAGGATGAGGAT

>AY358038

TTTTTAGATGGGATAGATAAGGCTCAAGAAGAACATGAAAGATATCACAGCAATTGGAGAACAATGGCTAGTGATTTTAATTTGCCACCTGTGGTAGCAAAAGAAATAGTAGCCAACTGTGATAAATGTCAATTAAAAGGGGAAGCTATGCATGGACAAGTAGACTGTAGTCCAGGGATATGGCAATTAGATTGCACACATCTAGAAGGAAAAGTCATCCTGGTAGCAGTCCACGTGGCCAGTGGATATATAGAAGCAGAAGTTATCCCAGCAGAAACAGGACAGGAGACAGCATACTTTCTGCTAAAATTAGCAGGAAGATGGCCAGTAAAAGTAATACACACAGACAATGGTAGCAATTTCACCAGTGCTGCAGTTAAGGCAGCCTGTTGGTGGGCCAATGTCCGACAGGAGTTTGGGATCCCCTACAATCCTCAAAGTCAAGGAGTAGTAGAATCTATGAATAAGGAATTAAAGAAAATCATAGGGCAGATAAGAGAGCAAGCTGAACACCTTAAGACAGCAGTACAAATGGCAGTATTCATTCACAATTTTAAAAGAAAAGGGGGGATTGGGGGGTACAGTGCAGGGGAAAGAATAATAGACATAATAGCAACAGACATACAAACTAAAGAATTACAAAAACAAATTACAAAAATTCAAAATTTTCGGGTTTATTACAGGGACAGCAGAGACCCAATTTGGAAAGGACCAGCAAAACTACTCTGGAAAGGTGAAGGGGCAGTAGTAATACAAGACAATAGTGATATAAAAGTAGTACCAAGAAGAAAAGCAAAAATCATTAGGGATTATGGAAAACAGATGGCAGGTGATGATTGTGTGGCAGGTAGACAGGATGAGGAT

>AY358039

TTTTTAGATGGGATAGATAAGGCCCAAGAAGAGCATGAAAGATATCACAGCAATTGGAGAACAATGGCTAGTGATTTTAATTTGCCACCTATAGTAGCAAAGGAAATTGTAGCCAACTGTGATAAATGTCAACTAAAAGGGGAAGCTATGCATGGACAAGTGGACTGTAGTCCAGGGATATGGCAATTAGATTGCACACATCTAGAAGGAAAAGTAATCCTGGTAGCAGTCCACGTGGCCAGTGGATATATAGAAGCAGAAGTTATCCCAGCAGAAACAGGATCGGAGACAGCATACTTTCTGCTAAAACTAGCAGGAAGATGGCCAGTAAAAGTAATACACACAGACAACGGTAGCAATTTCACCAGCGCTGCAGTTAAAGCAGCCTGTTGGTGGGCCAATGTCCGACAGGAATTTGGGATCCCCTACAATCCCCAAAGTCAAGGAGTAGTAGAATCCATGAATAAGGAATTAAAGAAAATCATAGGGCAGGTAAGAGAGCAAGCTGAACACCTTAAGACAGCAGTACAAATGGCAGTATTCATTCACAATTTTAAAAGAAAAGGGGGGATTGGGGGGTACAGTGCAGGGGAAAGAATAATAGACATAATAGCAACAGACATACAAACTAAAGAATTACAAAAACAAATTACAAAAATTCAAAATTTTCGGGTTTATTACAGGGACAGCAGAGACCCAATTTGGAAAGGACCAGCAAAACTACTCTGGAAAGGTGAAGGGGCAGTAGTAATACAAGACAATAGTGATATAAAAGTAGTACCAAGAAGAAAAGCAAAGATCATTAGGGATTATGGAAAACAGATGGCAGGTGATGATTGTGTGGCAGGTAGACAGGATGAGGAT

>AY358040

TTTTTAGATGGGATAGATAAGGCTCAAGAAGAACATGAAAGATATCACAGCAATTGGAGAACAATGGCTAGTGATTTTAATTTGCCACCTATAGTAGCAAAGGAAATAGTAGCCAACTGTGATAAGTGTCAACTAAAAGGGGAAGCTATGCATGGACAAGTAGACTGTAGTCCAGGAATATGGCAATTAGATTGCACACATCTAGAAGGAAAAGTCATCCTGGTAGCAGTCCACGTGGCCAGTGGATATATAGAAGCAGAAGTTATCCCAGCAGAAACAGGACACGAGACAGCATACTTTCTGCTAAAATTAGCAGGAAGATGGCCAGTAAAAGTAATACACACAGACAACGGTAGCAATTTCACCAGCGCTGCAGTTAAAGCAGCCTGTTGGTGGGCCAATATCCAACAAGAATTTGGGATCCCCTACAATCCCCAAAGTCAAGGAGTAGTAGAATCCATGAATAAGGAATTAAAGAAAATCATAGGGCAGGTAAGAGAGCAAGCTGAACACCTTAAGACAGCAGTACAAATGGCAGTATTCATTCACAATTTTAAAAGAAAAGGGGGGATTGGGGGGTACAGTGCAGGGGAAAGAATAATAGACATAATAGCAACAGACATACAAACCAAAGAATTACAAAAACAAATTACAAAAATTCAAAATTTTCGGGTTTATTACAGGGACAGCAGAGACCCAATTTGGAAAGGACCAGCAAAACTACTCTGGAAAGGTGAAGGGGCAGTAGTAATACAAGACAATAGTGATATAAAAGTAGTACCAAGAAGAAAAGCAAAGATCATTAGGGATTATGGAAAACAGATGGCAGGTGATGATTGTGTGGCAGGTAGACAGGATGAGGAT

>AY358041

TTTTTAGATGGGATAGATAAGGCACAAGAAGACCATGAAAGATATCACAGCAATTGGAGAACAATGGCTAGTGATTTTAATTTGCCACCTATAGTAGCAAAGGAAATAGTAGCCAACTGTGATAAATGTCAACTAAAAGGGGAAGCTATGCATGGACAAGTAGATTGTAGTCCAGGGATATGGCAATTAGATTGCACACATCTAGAAGGAAAAGTCATCATTGTAGCAGTCCACGTGGCCAGTGGATATATAGAAGCAGAAGTTATCCCAGCAGAAACAGGACAGGACACAGCATACTTTCTGCTAAAATTAGCAGGAAGATGGCCAGTAAAAGTAATACACACAGACAACGGTAGCAATTTCACCAGTGCTGCAGTTAAAGCAGCCTGTTGGTGGGCCAATGTCCGACAGGAATTTGGGATCCCCTACAATCCCCAAAGTCAAGGAGTAGTAGAATCTATGAATAAGGAATTAAAGAAAATCATAGGGCAGGTAAGAGAGCAAGCTGAACACCTTAAGACAGCAGTACAAATGGCAGTATTCATTCACAATTTTAAAAGAAAAGGGGGGATTGGGGGGTACAGTGCAGGGGAAAGAATAATAGACATAATAGCAACAGACATACAAACCAAAGAATTACAAAAACAAATTACAAAAATTCAAAATTTTCGGGTTTATTACAGGGACAGCAGAGACCCAATTTGGAAAGGACCAGCAAAACTACTCTGGAAAGGTGAAGGGGCAGTAGTAATACAAGACAATAGTGATATAAAAGTAGTACCAAGAAGAAAAGCAAAGATCATTAGGGATTATGGAAAACAGATGGCAGGTGATGATTGTGTGGCAGGTAGACAGGATGAGGAT

>AY358042

TTTTTAGATGGGATAGATAAGGCTCAAGAAGAACATGAAAGATATCACAGCAATTGGAGAACAATGGCTAGTGACTTTAATTTGCCACCTATAGTAGCAAAGGAAATAGTAGCCAACTGTGATAAATGTCAACTAAAAGGGGAAGCTATGCATGGACAGGTAGACTGTAGTCCAGGAATATGGCAATTAGATTGCACACATCTAGAAGGAAAAGTCATCCTGGTAGCAGTCCACGTGGCCAGTGGATATATAGAAGCAGAAGTTATCCCAGCAGAAACAGGACAGGAGACAGCATACTTTCTGCTAAAACTAGCAGGAAGATGGCCAGTAAAAGTAATACACACAGACAATGGTAGCAATTTCACCAGCGCTGCAGTTAAAGCAGCCTGTTGGTGGGCCAATGTCCGACAGGAATTTGGGATCCCCTACAATCCCCAAAGTCAAGGAGTAGTAGAATCTATGAATAAGGAATTAAAGAAAATCATAGGGCAGGTAAGAGAGCAAGCTGAACACCTTAAGACAGCAGTACAAATGGCAGTATTCATTCACAATTTTAAAAGAAAAGGGGGGATTGGGGGGTACAGTGCAGGGGAAAGAATAATAGACATAATAGCAACAGACATACAAACTAAAGAATTACAAAAACAAATTACAAAAATTCAAAATTTTCGGGTTTATTACAGGGACAGCAGAGACCCAATTTGGAAAGGACCAGCAAAACTACTCTGGAAAGGTGAAGGGGCAGTAGTAATACAAGACAATAGTGATATAAAAGTAGTACCAAGAAGAAAAGCAAAGATCATTAGGGATTATGGAAAACAGATGGCAGGTGATGATTGTGTGGCAGGTAGACAGGATGAGGAT

>AY358043

TTTCTAGATGGGATAGATAAGGCTCAAGAAGAACATGAAAGATATCACAGCAATTGGAGAACAATGGCTAGTGATTTTAATTTACCACCTATAGTAGCAAAGGAAATAGTAGCCAACTGTGATAAATGTCAGCTAAAAGGGGAAGCTATGCATGGACAAGTAGACTGTAGTCCAGGGATATGGCAATTAGATTGCACACATCTAGAAGGAAAAATCATCCTGGTAGCAGTCCACGTGGCCAGTGGATATATAGAAGCAGAAGTTATCCCAGCAGAAACAGGACAGGAGACAGCATACTTTATACTAAAATTAGCAGGAAGATGGCCAGTAAAAGTAATACATACAGACAATGGTAGCAATTTCACCAGCGCTACAGTTAAAGCAGCCTGTTGGTGGGCCAATGTCCGACAGGAATTTGGGATCCCCTACAATCCTCAAAGTCAAGGAGTAGTAGAATCTATGAATAAGGAATTAAAGAAAATCATAGGGCAAGTAAGAGAGCAAGCTGAACACCTTAAGACAGCAGTACAAATGGCAGTATTCATTCACAATTTTAAAAGAAAAGGGGGGATTGGGGGGTACAGTGCAGGGGAAAGAATAATAGATATAATAGCAACAGACATACAAACTAAAGAATTACAAAAACAAATTACAAAAATTCAAAATTTTCGGGTTTATTACAGGGACAGCAGAGACCCAATTTGGAAAGGACCAGCAAAACTACTTTGGAAAGGTGAAGGGGCAGTAGTAATACAAGACAATAGTGATATAAAAGTAGTACCAAGAAGAAAAGCAAAGATCATTAGGGATTATGGAAAACAGATGGCAGGTGATGATTGTGTGGCAGGTAGACAGGATGAGGAT

>AY358044

TTTTTAGATGGGATAGATAAGGCTCAAGAAGAACATGAAAGATATCACAGCAATTGGAGAACAATGGCTAGTGATTTTAATTTGCCACCTATAGTAGCAAAGGAAATAGTAGCCAACTGTGATAAATGTCAAGTAAAAGGGGAAGCTATGCATGGACAAGTAGACTGTAGTCCAGGGCTATGGCAATTAGATTGCACACATCTAGAAGGAAAAGTTATCCTGGTAGCCGTCCACGTGGCCAGTGGATATATAGAAGCAGAAGTTATCCCAGCAGAAACAGGACAGGAGACAGCATACTTTCTGCTAAAATTAGCAGGAAGATGGCCAGTAAAAGTAATACACACAGACAACGGTAGCAATTTCACCAGCGCTGCAGTTAAAGCAGCCTGTTGGTGGGCCAATGTCCGACAGGAATTTGGGATCCCCTACAATCCCCAAAGTCAGGGAGTAGTAGAATCTATGAATAAGGAATTAAAGAAAATCATAGGGCAGATAAGAGAGCAAGCTGAACACCTTAAGACAGCAGTACAAATGGCAGTATTCATTCACAATTTTAAAAAGAAAGGGGGGATTGGGGGGTACAGTGCAGGGGAAAGAATAATAGACATAATAGCAACAGACATACAAACTAAAGAATTACAAAAACAAATTACAAAAATTCAAAATTTTCGGGTTTATTACAGGGACAGCAGAGACCCAATTTGGAAAGGACCAGCAAAACTACTCTGGAAAGGTGAAGGGGCAGTAGTAATACAAGACAATAGTGATATAAAAGTAGTACCAAGAAGAAAAGCAAAGATTATCAGGGATTATGGAAAACAGATGGCAGGTGATGATTGTGTGGCAGGTAGACAGGATGAGGAT

>AY358045

TTTTTAGATGGGATAGATAAGGCTCAAGAAGAACATGAAAGATATCACAGCAATTGGAGAACAATGGCTAGTGATTTTAATTTGCCACCTATAGTAGCAAAGGAAATAGTAGCCAACTGTGATAAATGTCAAGTAAAAGGGGAAGCTATGCATGGACAAGTAGACTGTAGTCCAGGGCTATGGCAATTAGATTGCACACATCTAGAAGGAAAAGTTATCCTGGTAGCAGTCCACGTGGCCAGTGGATATATAGAAGCAGAAGTTATCCCAGCAGAAACAGGACAGGAGACAGCATACTTTCTGCTAAAATTAGCAGGAAGATGGCCAGTAAAAGTAATACACACAGACAACGGTAGCAATTTCACCAGCGCTGCAGTTAAAGCAGCCTGTTGGTGGGCCAATGTCCGACAGGAATTTGGGATTCCCTACAATCCCCAAAGTCAGGGAGTAGTAGAATCTATGAATAAGGAATTAAAGAAAATCATAGGGCAGATAAGAGAGCAAGCTGAACACCTTAAGACAGCAGTACAAATGGCAGTATTCATTCACAATTTTAAAAAGAAAGGGGGGATTGGGGGGTACAGTGCAGGAGAAAGAATAATAGACATAATAGCAACAGACATACAAACTAAAGAATTACAAAAACAAATTACAAAAATTCAAAATTTTCGGGTTTATTACAGGGACAGCAGAGACCCAATTTGGAAAGGACCAGCAAAACTACTCTGGAAAGGTGAAGGGGCAGTAGTAATACAAGACAATAGTGATATAAAAGTAGTACCAAGAAGAAAAGCAAAGATTATCAGGGATTATGGAAAACAGATGGCAGGTGATGATTGTGTGGCAGGTAGACAGGATGAGGAT

>AY358046

TTTTTAGATGGGATAGACAAGGCCCAAGAAGACCATGAAAGATATCACAGCAATTGGAGAACAATGGCTAGTGATTTTAATTTGCCACCTATAGTAGCAAAGGAAATTGTAGCCAACTGTGATAAATGTCAACTAAAAGAAAAAGCTATGCATGGACAAGTGGACTGTAGTCCAGGGATATGGCAATTAGATTGCACACATCTAGAAGGAAAAGTAATCCTGGTAGCAGTCCACGTGGCCAGTGGATATATAGAAGCAGAAGTTATCCCAGCAGAAACAGGATCAGAGACAGCATACTTTCTGCTAAAACTAGCAGGAAGATGGCCAGTAAAAGTAATACACACAGACAACGGTAGCAATTTCACCAGCGCTGCAGTTAAAGCAGCCTGTTGGTGGGCCAATGTCCGACAGGAATTTGGGATCCCCTACAATCCCCAAAGTCAAGGAGTAGTAGAATCCATGAATAAGGAATTAAAGAAAATCATAGGGCAGGTAAGAGAGCAAGCTGAACACCTTAAGACAGCAGTACAAATGGCAGTATTCATTCACAATTTTAAAAGAAAAGGGGGGATTGGGGGGTACAGTGCAGGGGAAAGAATAATAGACATAATAGCAACAGACATACAAACTAAAGAATTACAAAAACAAATTACAAAAATTCAAAATTTTCGGGTTTATTACAGGGACAGCAGAGACCCAATTTGGAAAGGACCAGCAAAACTACTCTGGAAAGGTGAAGGGGCAGTAGTAATACAAGACAATAGTGATATAAAAGTAGTACCAAGAAGAAAAGCAAAGATCATTAGGGATTATGGAAAACAGATGGCAGGTGATGATTGTGTGGCAGGTAGACAGGATGAGGAT

>AY358047

TTTTTAGATGGGATAGATAAGGCCCAAGAAGAGCATGAAAGATATCACAGCAATTGGAGAACAATGGCTAGTGATTTTAATTTGCCACCTATAGTAGCAAAGGAAATTGTAGCCAACTGTGATAAATGTCAACTAAAAGGGGAAGCTATGCATGGACAAGTGGACTGTAGTCCAGGGATATGGCAATTAGATTGCACACATCTAGAAGGAAAAGTAATCCTGGTAGCAGTCCACGTGGCCAGTGGATATATAGAAGCAGAAGTTATCCCAGCAGAAACAGGATCGGAGACAGCATACTTTCTGCTAAAACTAGCAGGAAGATGGCCAGTAAAAGTAATACACACAGACAACGGTAGCAATTTCACCAGCGCTGCAGTTAAAGCAGCCTGTTGGTGGGCCAATGTCCGACAGGAATTTGGGATCCCCTACAATCCCCAAAGTCAAGGAGTAGTAGAATCCATGAATAAGGAATTAAAGAAAATCATAGGGCAGGTAAGAGAGCAAGCTGAACACCTTAAGACAGCAGTACAAATGGCAGTATTCATTCACAATTTTAAAAGAAAAGGGGGGATTGGGGGGTACAGTGCAGGGGAAAGAATAATAGACATAATAGCAACAGACATACAAACTAAAGAATTACAAAAACAAATTACAAAAATTCAAAATTTTCGGGTTTATTACAGGGACAGCAGAGACCCAATTTGGAAAGGACCAGCAAAACTACTCTGGAAAGGTGAAGGGGCAGTAGTAATACAAGACAATAGTGATATAAAAGTAGTACCAAGAAGAAAAGCAAAGATCATTAGGGATTATGGAAAACAGATGGCAGGTGATGATTGTGTGGCAGGTAGACAGGATGAGGAT

>AY358048

TTTTTAGATGGGATAGATAAGGCCCAAGAAGAGCATGAAAGATATCACAGTAATTGGAGAACAATGGCTAGTGATTTTAATTTGCCACCTATAGTAGCAAAGGAAATTGTAGCCAACTGTGATAAATGTCAACTAAAAGGGGAAGCTATGCATGGACAAGTGGACTGTAGTCCAGGGATATGGCAATTAGATTGCACACATCTAGAAGGAAAAGTAATCCTGGTAGCAGTCCACGTGGCCAGTGGATATATAGAAGCAGAAGTTATCCCAGCAGAAACAGGATCGGAGACAGCATACTTTCTGCTAAAACTAGCAGGAAGATGGCCAGTAAAAGTAATACACACAGACAACGGTAGCAATTTCACCAGCGCTGCAGTTAAAGCAGCCTGTTGGTGGGCCAATGTCCGACAGGAATTTGGGATCCCCTACAATCCCCAAAGTCAAGGAGTAGTAGAATCCATGAATAAGGAATTAAAGAAAATCATAGGGCAGGTAAGAGAGCAAGCTGAACACCTTAAGACAGCAGTACAAATGGCAGTATTCATTCACAATTTTAAAAGAAAAGGGGGGATTGGGGGGTACAGTGCAGGGGAAAGAATAATAGACATAATAGCAACAGACATACAAACTAAAGAATTACAAAAACAAATTACAAAAATTCAAAATTTTCGGGTTTATTACAGGGACAGCAGAGACCCAATTTGGAAAGGACCAGCAAAACTACTCTGGAAAGGTGAAGGGGCAGTAGTAATACAAGACAATAGTGATATAAAAGTAGTACCAAGAAGAAAAGCAAAGATCATTAGGGATTATGGAAAACAGATGGCAGGTGATGATTGTGTGGCAGGTAGACAGGATGAGGAT

>AY358049

TTTTTAGATGGAATAGATAAGGCTCAAGAAGAACATGAAAGATATCACAGCAATTGGAGAACAATGGTTAGTGATTTTAATTTGCCACCTGTAGTAGCAAAGGAAATAGTAGCCAACTGTGATAAATGTCAACTAAAAGGGGAAGCTATGCATGGACAAGTAGACTGTAGTCCAGGGATATGGCAATTAGATTGCACACATCTAGAAGGTAAAGTCATCCTGGTAGCAGTCCACGTAGCCAGTGGGTATATAGAAGCAGAAGTTATTCCAGCAGAAACAGGACAGGAGACAGCATACTTTCTGCTAAAATTAGCAGGAAGATGGCCAGTAAAAGTAATACACACAGACAACGGTAGTAATTTCACCAGCGCTGCAGTTAAAGCAGCCTGTTGGTGGGCCAATGTCCGACAGGAATTTGGGATCCCCTACAATCCCCAAAGTCAAGGAGTAGTAGAATCTATGAATAAGGAATTAAAGAAAATCATAGGGCAGGTAAGAGAGCAAGCTGAACACCTTAAGACAGCAGTACAAATGGCAGTATTCATTCACAATTTTAAAAGAAAAGGGGGGATTGGGGGGTACAGTGCAGGGGAAAGAATAATAGATATAATAGCAACAGACATACAAACTAAAGAATTACAAAAACAAATTACAAAAATTCAAAATTTTCGGGTTTATTACAGGGACAGCAGAGACCCAATTTGGAAAGGACCAGCAAAACTACTCTGGAAAGGTGAAGGGGCAGTAGTAATACAAGACAATAGTGATATAAAAGTAGTACCAAGAAGAAAAGCAAAGATCATCAGGGATTATGGAAAACAGATGGCAGGTGATGATTGTGTGGCAGGTAGACAGGATGAGGAT

>AY358050

TTTTTAGATGGGATAGATAAGGCTCAAGAAGAACATGAAAGATATCACAGCAATTGGAGAACAATGGCTAGTGATTTTAATTTGCCACCTATAGTAGCAAAGGAAATAGTAGCCAACTGTAATAAGTGTCAGCTAAAAGGGGAAGCTATGCATGGACAAGTAGACTGTAGTCCAGGGATATGGCAATTAGATTGCACTCATCTAGAAGGAAAAGTCATCCTGGTAGCAGTCCACGTGGCCAGTGGATATATAGAAGCAGAAGTTATCCCAGCAGAAACAGGACAGGAGACAGCATACTTTCTGCTAAAATTAGCAGGAAGATGGCCAGTAAAAGTAATACACACAGACAATGGTAGCAACTTCACCAGCGCTGCAATGAAAGCAGCCTGTTGGTGGGCCGATGTCCACCAGGAATTTGGAATCCCCTACAATCCCCAGAGTCAGGGAGTAGTAGAATCAATGAATAAGGAATTAAAGAAAATCATAGGGCAGGTAAGAGAGCAAGCTGAACACCTTAAGACAGCAGTACAAATGGCAGTATTTATTCACAATTTTAAAAGAAAAGGGGGGATTGGGGGGTACAGTGCAGGGGAAAGAATAATAGACATGATAGCAACAGACATACAAACTAAAGAATTACAAAAACAAATTACAAAAATTCAAAATTTTCGGGTTTATTACAGGGACAGCAGAGACCCAATTTGGAAAGGACCAGCAAAACTACTCTGGAAAGGTGAAGGGGCAGTAGTAATACAAGACAATAGTGATATAAAAGTAGTACCAAGAAGAAAAGCAAAGATCATTAGGGATTATGGAAAACAGATGGCAGGTGATGATTGTGTGGCAGGTAGACAGGATGAGAAT

>AY358051

TTTTTAGATGGGATAGATAAAGCTCAAGAAGATCATGACAGATATCACAGCAATTGGAGAACAATGGCTAGTGATTTTAATTTGCCACCTGTAGTAGCAAAGGAAATAGTAGCCAACTGTGATAAATGTCAGCTAAAAGGGGAAGCTATGCATGGACAAGTAGACTGTAGTCCAGGGATATGGCAATTAGATTGCACACATCTAGAAGGTAAAGTCATCCTGGTAGCAGTCCACGTAGCCAGTGGGTATATAGAAGCAGAAGTTATTCCAGCAGAAACAGGACAGGAGACAGCATACTTTCTGCTAAAATTAGCAGGAAGATGGCCAGTAAAAGTAATACACACAGACAACGGTAGCAATTTCACCAGCGCTGCAGTTAAAGCAGCCTGTTGGTGGGCCAATGTCCGACAGGAATTTGGGATCCCCTACAATCCCCAAAGTCAAGGAGTAGTAGAATCTATGAATAAGGAATTAAAGAAAATCATAGGGCAGGTAAGAGAGCAAGCTGAACACCTTAAAACAGCAGTACAAATGGCAGTATTCATTCACAATTTTAAAAGAAAAGGGGGGATTGGGGGGTACAGTGCAGGGGAAAGAATAATAGACATAATAGCAACAGACATACAAACTAAAGAATTACAAAAACAAATTACAAAAATTCAAAATTTTCGGGTTTATTACAGGGACAGCAGAGACCCAATTTGGAAAGGACCAGCAAAACTACTCTGGAAAGGTGAAGGGGCAGTAGTAATACAAGACAATAGTGATATAAAAGTAGTACCAAGAAGAAAAGCAAAGATCATCAGGGATTATGGAAAACAGATGGCAGGTGATGATTGTGTGGCAGGTAGACAGGATGAGGAT

>AY358052

TTTTTAGATGGGATAGATAAGGCTCAAGAAGATCATGACAGATATCACAGCAATTGGAGAACAATGGCTAGTGATTTTAATTTGCCACCTGTAGTAGCAAAGGAAATAGTAGCCAACTGTGATAAATGTCAGCTAAAAGGGGAAGCTATGCATGGACAAGTAGACTGTAGTCCAGGGATATGGCAATTAGATTGCACACATCTAGAAGGAAAAGTCATCCTGGTAGCAGTCCATGTGGCCAGTGGATATATAGAAGCAGAAGTTATCCCAGCAGAAACAGGACAGGAGACAGCACACTTTCTGCTAAAATTAGCAGGAAGATGGCCAGTAAAAGTAATACACACAGACAACGGTAGCAATTTCACCAGCGCTGCAGTTAAAGCAGCCTGTTGGTGGGCCAATGTCCAACAGGAATTTGGGATCCCCTACAATCCCCAAAGTCAAGGAGTAGTACAATCTATGAATAAGGAATTAAAGAAAATCATAGGGCAGGTGAGAGAGCAAGCTGAACACCTTAAGACAGCAGTACAAATGGCAGTATTCATTCACAATTTTAAAAGAAAAGGGGGGATTGGGGGGTACAGTGCAGGGGAAAGAATAATAGACATAATAGCAACAGACATACAAACTAAAGAATTACAAAAACAAATTACAAAAATTCAAAATTTTCGGGTTTATTACAGGGACAGCAGAGACCCAATTTGGAAAGGACCAGCAAAACTACTCTGGAAAGGTGAAGGGGCAGTAGTAATACAAGACAATAGTGATATAAAAGTAGTACCAAGAAGAAAAGCAAAGATCATTAGGGATTATGGAAAACAGATGGCAGGTGATGATTGTGTGGCAGGTAGACAGGATGAGGAT

>AY358053

TTTTTAGATAGGATAGATAAGGCTCAAGAAGAACATGAAAGATATCACAGCAATTGGAGAACAATGGCTAGTGATTTTAATTTGCCACCTATAGTAGCAAAGGAAATAGTAGCCAACTGTGAAAAATGTCAACTAAAAGGGGAAGCTATGCATGGACAAGTAGACTGTAGTCCAGGGATATGGCAATTAGATTGCACACATCTAGAAGGAAAAGTCATCCTAGTAGCAGTCCACGTGGCCAGTGGATATATAGAAGCAGAAGTTATCCCAGCAGAAACAGGACAGGAGACAGCATACTTTCTGCTAAAATTAGCAGGGAGATGGCCAGTAAAAGTAATACACACAGACAATGGTAGCAATTTCACCAGCGCTGCAGTTAAAGCAGCCTGTTGGTAGGCCAATGTCCGGCAGGAATTTAGGATTCCCTACAATCCCCAAAGTCAAGGAGTAGTAGAATCCATGAATAAGGAATTAAAGAAAATCATAGGGCAGGTAAGAGAGCAAGCTGAACACCTTAAAACAGCAGTACAAATGGCAGTATTCATTCACAATTTTAAAAGAAAAGGGGGGATTGGGGGGTACAGTGCAGGAGAAAGAATAATAGACATAATAGCAACAGACATACAAACTAAAGAATTACAAAAACAAATTACAAAAATTCAAAATTTTCGGGTTTATTACAGGGACAGCAGAGATCCAATTTGGAAAGGACCAGCAAAACTACTCTGGAAAGGTGAAGGGGCAGTAGTAATACAAGACAATAGTGATATAAAAGTAGTGCCAAGAAGAAAAGCAAAGATCATTAGGGATTATGGAAAACAGATGGCAGGTGATGATTGTGTGGCAGGTAGACAGGATGAGGAT

>AY358056

TTTTTAGATGGGATAGATAAGGCTCAAGAAGAACATGAAAGATATCATAGCAATTGGAGAACAATGGCTAGTGAATTTAATCTGCCACCTATAGTAGCAAAGGAAATAGTAGCCAACTGTGATAAATGTCAACTAAAAGGGGAAGCTATGCATGGACAAGTGGACTGTAGTCCAGGGATATGGCAATTAGATTGTACACATCTAGAAGGAAAAGTCATCCTGGTAGCAGTCCACGTGGCCAGTGGATATATAGAAGCAGAAGTTATCCCAGCAGAAACAGGACAGGAGACAGCATACTTTCTGCTAAAATTAGCAGGAAGATGGCCAGTAAAAGTAATACACACAGACAACGGTAGCAATTTCACCAGCGCTGCAGTTAAAGCAGCCTGTTGGTGGGCCAATGTCCGACAGGAATTTGGGATCCCCTACAATCCTCAAAGTCAAGGAGTAGTAGAATCTATGAATAAGGAATTAAAGAAAATCATAGGGCAGGTAAGAGAGCAAGCTGAACACCTTAAGACAGCAGTACAAATGGCAGTATTCATTCACAATTTTAAAAGAAAAGGGGGGATTGGGGGGTACAGTGCAGGGGAAAGAATAATAGACATAATAGCAACAGACATACAAACTAAAGAATTACAAAAACAAATTACAAAAATTCAAAATTTTCGGGTTTATTACAGGGACAACAGAGACCCAATTTGGAAAGGACCAGCAAAACTACTTTGGAAAGGTGAAGGGGCAGTAGTAATACAAGACAATAGTGATATAAAAGTTGTACCAAGAAGAAAAGCAAAGATCATTAGAGATTATGGAAAACAGATGGCAGGTGATGATTGTGTGGCAGGTAGACAGGATGAGGAT

>AY358057

TTTTTAGATGGGATAGATAAAGCTCAAGAAGAGCATGAAAGATATCACAGCAATTGGAGAACAATGGCTAGTGACTTTAATTTGCCACCTATAGTAGCAAAGGAAATAGTAGCCAACTGTGATAAATGTCAACTAAAAGGGGAAGCTATGCATGGACAAGTAGACTGTAGTCCAGGGATATGGCAACTAGATTGCACACATCTAGAAGGAAAAGTCATCCTGGTGGCAGTCCACGTGGCCAGTGGATATATAGAAGCAGAAGTTATCCCAGCAGAAACAGGACAGGAGACAGCATACTTTCTGCTAAAATTAGCAGGAAGATGGCCAGTAAAAGTAATACACACAGACAACGGTAGCAATTTCACCAGCGCTGCAGTTAAAGCAGCCTGTTGGTGGGCCAATGTCCGACAGGAATTTGGGATCCCCTACAATCCCCAAAGTCAAGGAGTAGTAGAATCTATGAATAAGGAATTAAAGAAAATCATAGGGCAGGTAAGGGAGCAAGCTGAACACCTTAAGACAGCAGTACAAATGGCAGTATTCATTCACAATTTTAAAAGAAAAGGGGGGATTGGGGGGTACAGTGCAGGGGAAAGAATAATAGACATAATAGCAACAGACATACAAACTAAAGAATTACAAAAACAAATTACAAAAATTCAAAATTTTCGGGTTTATTACAGGGACAGCAGAGACCCAATTTGGAAAGGACCAGCAAAACTACTCTGGAAAGGTGAAGGGGCAGTAGTAATACAAGACAATAGTGACATAAAAGTAGTACCAAGAAGAAAAGCAAAGATCATTAGGGATTATGGAAAACAGATGGCAGGTGATGATTGTGTGGCAGGTAGACAGGATGAGGAT

>AY358059

TTTTTAGATGGGATAGATAAGGCTCAAGAAGAACATGAAAGATATCACAGCAATTGGAGAACAATGGCTAGTGATTTTAATTTGCCACCTATAGTAGCAAAGGAAATAGTAGCCAACTGTGATAAATGTCAACTAAAAGGGGAAGCTATGCATGGACAAGTGGACTGTAGTCCAGGGATATGGCAATTAGATTGCACACATCTAGAAGGAAAAGTCATCCTGGTAGCAGTCCACGTGGCCAGTGGATATATAGAAGCAGAAGTTATCCCAGCAGAAACAGGACAGGAGACAGCATACTTTCTACTAAAATTAGCAGGAAGATGGCCAGTAAAAGTAATACACACAGACAACGGTAGCAATTTCACCAGCGCTGCAGTTAAAGCAGCCTGTTGGTGGGCCAATATCCGACAGGAATTTGGGATCCCCTACAATCCCCAAAGTCAAGGAGTAGTAGAATCTATGAATAAAGAATTAAAGAGAATAATAGGGCAGGTAAGAGAGCAAGCTGAACACCTTAAGACAGCAGTACAGATGGCAGTATTCATTCACAATTTTAAAAGAAAAGGGGGGATTGGGGGGTACAGTGCAGGGGAAAGAATAATAGACATAATAGCAACAGACATACAAACTAAAGAATTACAAAACCAAATTACAAAAATTCAAAATTTTCGGGTTTATTACAGGGACAGCAGAGACCCAATTTGGAAAGGACCAGCAAAACTACTCTGGAAAGGTGAAGGGGCAGTAGTAATACAAGACAATAGTGATATAAAAGTAGTACCAAGAAGAAAAGCAAAGATCATTAGGGATTATGGAAAACAGATGGCAGGTAATGATTGTGTGGCAGGTAGACAGGATGAGGAT

>AY358060

TTTTTAGATGGGATAGATAAGGCCCAAGAAGAGCATGAAAGATATCACAGCAATTGGAGAACAATGGCTAGTGATTTTAATTTGCCACCTATAGTAGCAAAGGAAATAGTAGCCAACTGTGATAAATGTCAACTAAAAGGGGAAGCTATGCATGGACAAGTAGACTGTAGTCCAGGGATATGGCAATTAGATTGCACACATCTAGAAGGAAAAGTCATCCTGGTAGCAGTTCACGTGGCCAGTGGATATATAGAAGCAGAAGTTATCCCAGCAGAAACAGGACAGGAAACAGCATACTTTCTGCTAAAGTTAGCAGGAAGATGGCCAGTAAAAGTAATACATACAGACAACGGTAGCAATTTCACCAGCGCTGCAGTTAAAGCAGCCTGTTGGTGGGCCAATGTCCAACAGGAATTTGGGATCCCCTACAATCCCCAAAGTCAAGGAGTAGTAGAATCTATGAATAAGGAATTAAAGAAAATCATAGGGCAGGTAAGAGAGCAAGCTGAACACCTTAAGACAGCAGTACAAATGGCAGTATTCATTCACAATTTTAAAAGAAAAGGGGGGATTGGGGGGTACAGTGCAGGGGAAAGAATAATAGACATAATAGCAACAGACATACAAACTAAAGAATTACAAAAACAAATTACAAAAATTCAAAATTTTCGGGTTTATTACAGGGACAGCAGAGACCCAATTTGGAAAGGACCAGCAAAACTACTCTGGAAAGGTGAAGGGGCAGTAGTAATACAAGACAATAGTGATATAAAAGTAGTACCAAGAAGAAAAGCAAAGATCATTAGGGATTATGGAAAACAGATGGCAGGTGATGATTGTGTGGCAGGTAGACAGGATGAGGAT

>AY358061

TTTTTAGATGGGATAGATAAGGCTCAAGAAGAACATGAAAGATATCACAGCAATTGGAGAACAATGGTTAGTGATTTTAATTTGCCACCTGTAGTAGCAAAGGAAATAGTAGCCAACTGTGATAAATGTCAACTAAAAGGGGAAGCTATGCATGGACAAGTGGACTGTAGTCCAGGGATATGGCAATTAGATTGCACACATCTAGAAGGTAAAGTCATCCTGGTAGCAGTTCACGTAGCCAGTGGGTATATAGAAGCAGAAGTTATTCCAGCAGAAACAGGACAAGAGACAGCATACTTTCTACTAAAATTAGCAGGAAGATGGCCAGTAAAAGTAATACACACAGACAACGGTAGCAATTTCACCAGCGCTGCAGTTAGAGCAGCCTGTTGGTGGGCCAATGTCCGACAGGAATTTGGGATCCCCTACAATCCCCAAAGTCAAGGAGTGGTAGAATCTATGAATAAGGAATTAAAGAAAATCATAGGGCAGGTAAGAGAGCAAGCTGAACACCTTAAGACAGCAGTACAAATGGCAGTATTCATTCACAATTTTAAAAGAAAAGGGGGGATTGGGGGGTACAGTGCAGGGGAAAGAATAATAGATATAATAGCAACAGACATACAAACTAAAGAATTACAAAAACAAATTACAAAAATTCAAAATTTTCGGGTTTATTACAGGGACAGCAGAGACCCAATTTGGAAAGGACCAGCAAAACTACTCTGGAAAGGTGAAGGGGCAGTAGTAATACAAGACAATAGTGATATAAAAGTAGTACCAAGAAGAAAAGCAAAGATCATCAGGGATTATGGAAAACAGATGGCAGGTGATGATTGTGTGGCAGGTAGACAGGATGAGGAT

>AY358062

TTTTTAGATGGGATAGATAAGGCTCAAGAAGAACATGAAAGATATCACAGCAATTGGAGAACAATGGCTAGTGATTTTAATTTACCACCTATAGTAGCAAAGGAAATAGTAGCCAACTGTGATAAATGTCAGCTAAAAGGGGAAGCTATGCATGGACAAGTGGACTGTAGTCCAGGGATATGGCAATTAGATTGCACACATCTAGAAGGAAAAGTCATCCTGGTAGCAGTCCACGTGGCTAGTGGATATATAGAAGCAGAAGTTATCCCAGCAGAAACAGGACAGGAGACAGCATACTTTCTGCTAAAATTAGCAGGAAGATGGCCAGTAAAAGTAATACACACAGACAATGGTAGCAATTTCACCAGCGCTGCAGTTAAAGCAGCCTGTTGGTGGGCCAATGTCAAACAGGAATTTGGGATCCCTTACAATCCCCAAAGTCAAGGAGTAGTAGAATCTATGAATAAGGAATTAAAGAAAATCATAGGGCAGGTAAGAGAGCAAGCTGAACACCTTAAGACAGCAGTACAAATGGCAGTATTCATTCACAATTTTAAAAGAAAAGGGGGGATTGGGGGGTACAGTGCAGGGGAAAGAATAATAGACATAATAGCAACAGACATACAAACTAAAGAATTACAAAAACAAATTACAAAAATTCAAAATTTTCGGGTTTATTACAGGGACAGCAGAGACCCAATTTGGAAAGGACCAGCAAAACTACTCTGGAAAGGTGAAGGGGCAGTAGTAATACAAGACAATAGTGATATCAAAGTAGTACCAAGAAGAAAAGCAAAGATCATCAGGGATTATGGAAAACAGATGGCAGGTGATGATTGTGTGGCAGGTAGACAGGATGAGGAT

>AY358063

TTTTTAGATGGGATAGATAAGGCTCAAGAAGAACATGAAAGATATCACAGCAATTGGAGAACAATGGCTAGTGATTTTAATTTACCACCTATAGTAGCAAAGGAAATAGTAGCCAGCTGTGATAAATGTCAACTAAAAGGGGAAGCTATGCATGGACAAGTAGACTGTAGTCCAGGGATATGGCAATTAGATTGCACACATCTAGAAGGAAAAGTCATCCTGGTAGCAGTCCACGTGGCCAGTGGATATATAGAAGCAGAAGTTATCCCAGCAGAAACAGGACAGGAAACAGCATACTTTCTGCTAAAATTAGCAGGAAGATGGCCAGTGAAAGTAATACACACAGACAACGGTAGCAATTTCACCAGTGCTGCAGTTAAAGCAGCCTGTTGGTGGGCCAATGTCCGACAGGAATTTGGGATCCCCTACAATCCCCAAAGTCAAGGAGTAGTAGAATCTATGAATAAGGAATTAAAGACAATCATAGGGCAGGTAAGAGAGCAAGCTGAACACCTTAAGACAGCAGTACAAATGGCAGTATTCATTCACAATTTTAAAAGAAAAGGGGGGATTGGGGGGTACAGTGCAGGGGAAAGAATAATAGACATAATAGCAACAGACATACAAACTAAAGAATTACAAAAGCAAATTACAAAAATTCAAAATTTTCGGGTTTATTACAGGGACAGCAGAGATCCAATTTGGAAAGGACCAGCAAAACTACTCTGGAAAGGTGAAGGGGCAGTAGTAATACAAGACAATAGTGATATAAAAGTAGTACCAAGAAGAAAAGCAAAGATCATTAGGGATTATGGAAAACAGATGGCAGGTGATGATTGTATGGCAGGTAGACAGGATGAGGAT

>AY358064

TTTTTAGATGGGATAGATAAGGCTCAAGAAGAACATGAAAGATATCACAACAATTGGAGAACAATGGCTAGTGACTTTAATTTGCCACCGATAGTAGCAAAGGAAATAGTAGCCAACTGTGATAAATGCCAACTAAAAGGGGAAGCTATGCATGGACAAGTAGACTGTAGTCCAGGGATATGGCAATTAGATTGCACACATCTAGAAGGAAAAGTCATCCTGGTAGCAGTCCACGTGGCCAGTGGATATATAGAAGCAGAAGTTATCCCAGCAGAAACAGGACAGGAGACAGCATACTTTCTGCTAAAATTAGCAGGAAGATGGCCAGTAAAAGTCATACACACAGACAACGGTAGCAATTTCACCAGCGCTGCAGTTAAAGCAGCATGTTGGTGGGCCAATGTCCGGCAGGAATTTGGGATCCCCTACAACCCCCAAAGTCAAGGAGTAGTAGAATCTATGAATAAGGAATTAAAGAAAATCATAGGACAGGTAAGAGAGCAAGCTGAACACCTTAAGACAGCAGTACAAATGGCAGTATTCATTCACAATTTTAAAAGAAAAGGGGGGATTGGGGGGTACAGTGCAGGGGAAAGAATAATAGACATAATAGCAACAGACATACAAACTAAAGAATTACAAAAACAAATTACAAAAATTCAAAATTTTCGGGTTTATTACAGGGACAGCAGAGACCCAATTTGGAAAGGACCAGCAAAACTACTCTGGAAAGGTGAAGGGGCAGTAGTAATACAAGACAATAGTGACATAAAAGTAGTACCAAGAAGAAAAGCAAAGATCATTAGGGATTATGGAAAACAGATGGCAGGTGATGATTGTGTGGCAGGTAGACAGGATGAGGAT

>AY358065

TTTCTAGATGGGATAGATAGGGCTCAAGAAGAACATGAAAGATATCACAGCAATTGGAGAACAATGGCTAGTGATTTTAATTTACCACCTATAGTAGCAAAGGAAATAGTAGCCAACTGTGATAAATGTCAGCTAAAAGGGGAAGCTATGCATGGACAAGTAGACTGTAGTCCAGGGATATGGCAATTAGATTGCACACATCTAGAAGGAAAAATCATCCTGGTAGCAGTCCACGTGGCCAGTGGATATATAGAAGCAGAAGTTATCCCAGCAGAAACAGGACAGGAGACAGCATACTTTATACTAAAATTAGCAGGAAGATGGCCAGTAAAAGTAATACATACAGACAATGGTAGCAATTTCACCAGCGCTGCAGTTAAAGCAGCCTGTTGGTGGGCCAATGTCCGACAGGAATTTGGGATCCCCTACAATCCTCAAAGTCAAGGAGTAGTAGAATCTATGAATAAGGAATTAAAGAAAATCATAGGGCAAGTAAGAGAGCAAGCTGAACACCTTAAGACAGCAGTACAAATGGCAGTATTCATTCACAATTTTAAAAGAAAAGGGGGGATTGGGGGGTACAGTGCAGGGGAAAGAATAATAGACATAATAGCAACAGACATACAAACTAAAGAATTACAAAAACAAATTACAAAAATTCAAAATTTTCGGGTTTATTACAGGGACAGCAGAGACCCAATTTGGAAAGGACCAGCAAAACTACTTTGGAAAGGTGAAGGGGCAGTAGTAATACAAGACAATAGTGATATAAAAGTAGTACCAAGAAGAAAAGCAAAGATCATTAGGGATTATGGAAAACAGATGGCAGGTGATGATTGTGTGGCAGGTAGACAGGATGAGGAT

>AY358066

TTTTTAGATGGGATAGATAAGGCCCAAGAAGAGCATGAAAGATATCACAGCAATTGGAGAACAATGGCTAGTGATTTTAATTTGCCACCTATAGTAGCAAAGGAAATTGTAGCCAACTGTGATAAATGTCAACTAAAAGGGGAAGCTATGCATGGACAAGTGGACTGTAGTCCAGGGATATGGCAATTAGATTGCACACATCTAGAAGGAAAAGTAATCCTGGTAGCAGTCCACGTGGCCAGTGGATATATAGAAGCAGAAGTTATCCCAGCAGAAACAGGATCGGAGACAGCATACTTTCTGCTAAAACTAGCAGGAAGATGGCCAGTAAAAGTAATACACACAGACAACGGTAGCAATTTCACCAGCGCTGCAGTTAAAGCAGCCTGTTGGTGGGCCAATGTCCGACAGGAATTTGGGATCCCCTACAATCCCCAAAGTCAAGGAGTAGTAGAATCCATGAATAAGGAATTAAAGAAAATCATAGGGCAGGTAAGAGAGCAAGCTGAACACCTTAAGACAGCAGTACAAATGGCAGTATTCATTCACAATTTTAAAAGAAAAGGGGGGATTGGGGGGTACAGTGCAGGGGAAAGAATAATAGACATAATAGCAACAGACATACAAACTAAAGAATTACAAAAACAAATTACAAAAATTCAAAATTTTCGGGTTTATTACAGGGACAGCAGAGACCCAATTTGGAAAGGACCAGCAAAACTACTCTGGAAAGGTGAAGGGGCAGTAGTAATACAAGACAATAGTGATATAAAAGTAGTACCAAGAAGAAAAGCAAAGATCATTAGGGATTATGGAAAACAGATGGCAGGTGATGATTGTGTGGCAGGTAGACAGGATGAGGAT

>AY358067

TTTTTAGATGGGATAGATAAGGCTCAAGAAGAACATGAAAGATATCACAGCAATTGGAGAACAATGGTTAGTGATTTTAATTTGCCACCAATAGTAGCAAAGGAAATAGTAGCCAGCTGTGATAAATGTCAGCTAAAAGGGGAAGCTATGCATGGACAAGTAGACTGTAGTCCAGGGATATGGCAATTAGATTGCACACATCTAGAAGGAAAAGTTATCCTAGTAGCAGTCCACGTGGCCAGTGGATATATAGAAGCAGAAGTTATCCCAGCAGAAACAGGACAGGAGACAGCATACTTTCTACTAAAATTAGCAGGAAGATGGCCAGTAAAAGTAATACACACAGACAACGGTAGCAATTTCACCAGCGCTGCAGTTAAAGCAGCCTGTTGGTGGGCCAATGTCCAACAGGAATTTGGGATCCCCTACAATCCCCAAAGTCAAGGAGTAGTAGAATCTATGAATAAGGAATTAAAGAAAATCATAGGACAGATAAGAGAACAAGCTGAACATCTTAAGACAGCAGTACAAATGGCAGTATTCATTCACAATTTTAAAAGAAAAGGGGGGATTGGGGGGTACAGTGCAGGGGAAAGAATAATAGACATAATAGCAACAGACATACAAACTAAAGAATTACAAAAACAAATTACAAAAATTCAAAATTTTCGGGTTTATTACAGGGACAGCAGAGACCCAATTTGGAAAGGACCAGCAAAACTACTCTGGAAAGGTGAAGGGGCAGTAGTAATACAAGACAATAGTGATATAAAAGTAGTACCAAGAAGAAAAGCAAAGATTATCAGGGATTATGGAAAACAGATGGCAGGTGATGATTGTGTGGCAGGTAGACAGGATGAGGAT

>AY358068

TTTTTAGATGGGATAGATAAGGCTCAAGAAGAACATGAAAGATATCACAGCAATTGGAGAACAATGGCTGGTGATTTTAATCTGCCACCTATAGTAGCAAAAGAAATAGTAGCCAACTGTGATAAATGTCAACTAAAAGGGGAAGCTATGCATGGACAAGTGGACTGTAGTCCAGGAATATGGCAATTAGATTGCACACATCTAGAAGGAAAAGTCATCCTGGTAGCAGTCCACGTGGCCAGTGGATATATAGAAGCAGAAGTTATCCCAGCAGAAACAGGACAGGAGACAGCATACTTTCTGCTAAAATTAGCAGGAAGATGGCCAGTAAAAGTAATACACACAGACAACGGTAGCAATTTCACCAGCGCTGCAGTTAAAGCAGCCTGTTGGTGGGCCAATGTCCAACAGGAATTTGGGATCCCCTACAATCCCCAAAGTCAAGGAGTAGTAGAATCCATGAATAAAGAATTAAAGAAAATCATAGGGCAGGTAAGAGAGCAAGCTGAACACCTTAAGACAGCAGTACAAATGGCAGTATTCATTCACAATTTTAAAAGAAAAGGGGGGATTGGGGGGTACAGTGCAGGGGAAAGAATAATAGACATGATAGCAACAGACATACAAACTAAAGAATTACAAAAACAAATTACAAAAATTCAAAATTTTCGGGTTTATTACAGGGACAGCAGAGACCCAATTTGGAAAGGACCAGCAAAACTACTCTGGAAAGGTGAAGGGGCAGTAGTAATACAAGACAATAGTGATATAAAAGTAGTACCAAGAAGAAAAGCAAAGATCATTAGGGATTATGGAAAACAGATGGCAGGTGATGATTGTGTGGCAGGTAGACAGGATGAGGAT

>AY358069

TTTTTAGATGGGATAGATAAAGCTCAAGAAGAACATGAAAGGTATCACAGCAATTGGAGAACAATGGCTAGTGATTTTAATTTGCCACCTATAGTAGCAAAGGAAATAGTAGCCAACTGTGATAAATGTCAACTAAAAGGGGAAGCTATGCATGGACAAGTAGACTGTAGTCCAGGGATATGGCAATTAGATTGTACACATCTGGAAGGAAAAGTCATCCTGGTAGCAGTCCACGTGGCCAGTGGATATATAGAAGCAGAAGTTATCCCAGCAGAAACAGGACAGGAGACAGCATACTTTCTGCTAAAATTAGCAGGAAGATGGCCAGTAAAAGTAATACACACAGACAACGGTAGCAATTTCACCAGTGCTGCAGTTAAAGCAGCCTGTTGGTGGGCCAATGTCCAACAGGAATTTGGGATCCCCTACAATCCCCAAAGTCAAGGAGTAGTAGAATCTATGAATAAGGAATTAAAGAAAATCATAGGGCAGGTAAGAGAGCAAGCTGAACACCTTAAGACAGCAGTACAAATGGCAGTATTCATTCACAATTTTAAGAGAAAAGGGGGGATTGGGGGGTACAGTGCAGGGGAAAGAATAATAGACATGATAGCAACAGACATACAAACTAAAGAATTACAAAGACAAATTACAAAAATTCAAAATTTCCGGGTTTATTACAGGGACAGCAGAGATCCAATTTGGAAAGGACCAGCAAAACTACTCTGGAAAGGTGAAGGGGCCGTAGTAATACAAGACAATAGTGATATAAAAGTAGTACCAAGAAGAAAAGCAAAAATCATTAGGGATTATGGAAAACAGATGGCAGGTGATGATTGTGTGGCAGGTAGACAGGATGAGGAT

>AY358070

TTTTTAGATGGGATAGATAAGGCTCAAGAAGAACATGAAAGATATCACAGTAATTGGAGAACAATGGCTAGTGATTTTAATTTACCACCTATAGTAGCAAAGGAAATAGTAGCCAGCTGTGATAAATGTCAACTAAAAGGGGAAGCTATGCATGGACAAGTAGACTGTAGTCCAGGAATATGGCAATTAGATTGCACACATCTAGAAGGAAAAGTCATCCTGGTAGCAGTCCATGTGGCCAGTGGATATATAGAAGCAGAAGTTATCCCAGCAGAAACAGGACAGGAGACAGCATACTTTCTGCTAAAATTAGCAGGAAGATGGCCAGTAAAAGTGATACACACAGACAATGGTAGCAATTTCACCAGCGCTGCAGTTAAAGCAGCTTGTTGGTGGGCCAATGTCCGACAGGAATTTGGGATCCCCTACAATCCCCAAAGTCAAGGAGTAGTAGAATCTATAAATAAGGAATTGAAGAAAATCATAGGGCAGGTAAGAGAGCAAGCTGAACACCTTAAGACAGCAGTACAAATGGCAGTATTCATTCACAATTTTAAAAGAAAAGGGGGGATTGGGGGGTACAGTGCAGGGGAAAGAATAATAGACATAATAGCAACAGATATACAAACTAAAGAATTACAAAAACAAATTACAAAAATTCAAAATTTTCGGGTTTATTACAGGGACAGCAGAGACCCAATTTGGAAAGGACCAGCAAAACTACTCTGGAAAGGTGAAGGGGCAGTAGTAATACAAGACAATAGTGATATAAAAGTAGTACCAAGAAGAAAAGCAAAGATCATTAGGGATTATGGAAAACAGATGGCAGGTGATGATTGTGTGGCAGGTAGACAGGATGAGGAT

>AY358071

TTTTTAGATGGAATAGATAAAGCTCAAGAAGAACATGAAAGATATCACAGCAATTGGAGAACAATGGCTAGTGATTTTAATTTGCCACCTGTAGTAGCAAAGGAAATAGTAGCCAACTGTGATAAATGTCAACTAAAAGGGGAAGCTATGCATGGACAAGTAGATTGTAGTCCAGGGATATGGCAATTAGATTGCACACATCTAGAAGGAAAAGTCATCCTGGTAGCAGTCCACGTGGCCAGTGGATATATAGAAGCAGAAGTTATCCCAGCAGAAACAGGACAAGAAACAGCATACTTTCTGCTAAAATTAGCAGGAAGATGGCCAGTAAAAGTAATACACACAGACAATGGTAGCAATTTCACCAGCGCTGCTGTTAAAGCAGCCTGTTGGTGGGCCAATGTCCGACAGGAATTTGGGATCCCCTACAATCCCCAAAGTCAAGGAGTAGTAGAATCTATGAATAAGGAATTAAAGAAAATCATAGGGCAGGTAAGAGAGCAAGCTGAACACCTTAAGACAGCAGTACAAATGGCAGTATTCATTCACAATTTTAAAAGAAAAGGGGGGATTGGGGGGTACAGTGCAGGGGAAAGAATAATAGATATAATAGCAACAGACATACAAACTAAAGAATTACAAAAACAAATTACAAAAATTCAAAATTTTCGGGTTTATTACAGGGACAGCAGAGACCCAATTTGGAAAGGACCAGCAAAACTACTCTGGAAAGGTGAAGGGGCAGTAGTAATACAAGACAATAGTGATATAAAAGTAGTACCAAGAAGAAAAGCAAAGATCATTAGGGATTATGGAAAACAGATGGCAGGTGATGATTGTGTGGCAGGTAGACAGGATGAGGAT

>AY358072

TTTTTAGATGGAATAGATAAAGCTCAAGAAGAACATGAAAGATATCACAGCAATTGGAGAACAATGGCTAGTGATTTTAATTTGCCACCTATAGTAGCAAAGGAAATAGTAGCCAACTGTGATAAATGTCAACTAAAAGGGGAAGCTATGCATGGACAAGTAGATTGTAGTCCAGGGATATGGCAATTAGATTGCACACATCTAGAAGGAAAAGTCATCCTGGTAGCAGTCCACGTGGCCAGTGGATATATAGAAGCAGAAGTTATCCCAGCAGAAACAGGACAGGAAACAGCATACTTTCTGCTAAAACTAGCAGGAAGATGGCCAGTAAAAGTAATACACACAGACAACGGTAGCAATTTCACCAGCGCTGCAGTTAAAGCAGCTTGTTGGTGGGCCAATGTCCGACAGGAATTTGGAATCCCCTACAATCCCCAAAGTCAAGGAGTAGTAGAATCTATGAATAAGGAATTAAAGAAAATTATAGGGCAGGTAAGAGAGCAAGCTGAACACCTCAAGACAGCAGTACAAATGGCAGTATTCATTCACAATTTTAAAAGAAAAGGGGGGATTGGGGGGTACAGTGCAGGGGAAAGAATAATAGATATAATAGCAACAGACATACAAACTAAAGAATTACAAAAACAAATTACAAAAATTCAAAATTTTCGGGTTTATTACAGGGACAGCAGAGACCCAATTTGGAAAGGACCAGCAAAACTACTCTGGAAAGGTGAAGGGGCAGTAGTAATACAAGACAATAGTGATATAAAAGTAGTACCAAGAAGAAAAGCAAAGATCATTAGGGATTATGGAAAACAGATGGCAGGTGATGATTGTGTGGCAGGTAGACAGGATGAGGAT

>AY358073

TTTTTAGATGGGATAGATAGGGCTCAAGAAGAACATGAAAGATATCACAGCAATTGGAGAGCAATGGCTAGTGATTTTAATTTGCCACCTATAGTAGCAAAGGAAATAGTAGCCAGCTGTGATAAATGTCAGCTAAAAGGGGAAGCTATGCATGGACAAGTGGACTGTAGTCCAGGGATATGGCAATTAGATTGCACACATCTAGAAGGAAAAGTCATCCTGGTGGCAGTCCACGTGGCCAGTGGATATATAGAAGCAGAAGTTATACCAGCAGAAACAGGACAAGAAACAGCATACTTTCTGCTAAAATTAGCAGGAAGATGGCCAGTAAAAGTAATACACACAGACAATGGTAGCAATTTCACCAGTGCTGCAGTTAAAGCAGCCTGTTGGTGGGCCAATGTCCGACAGGAATTTGGGATCCCCTACAATCCCCAAAGTCAAGGAGTAGTAGAATCTATGAATAAGGAATTAAAGAAAATCATAGGGCAGGTAAGAGAGCAAGCTGAACACCTTAAGACAGCAGTACAAATGGCAGTATTCATTCACAATTTTAAAAGAAAAGGGGGGATTGGGGGGTACAGTGCAGGGGAAAGAATAATAGACATAATAGCAACAGACATACAAACTAAAGAATTACAAAACCAAATTACAAAACTTCAAAATTTTCGGGTTTATTACAGGGACAGCAGAGATCCACTTTGGAAAGGACCAGCAAAGCTCCTTTGGAAAGGTGAAGGGGCAGTAGTAATACAAGACAATAGTGATATAAAAGTAGTACCAAGAAGAAAAGCAAAGATCATCAGGGATTATGGAAAACAGATGGCAGGTGATGATTGTGTGGCAGGTAGACAGAATGAGGAT

>AY530889

TTTCTAGAAAAAATAGAACCAGCTCAGGAAGAACATGAGAAATACCATAGCAATGTTAAAGAGCTACAGCATAAATTTGGTATACCACAATTAGTAGCCAAACAAATAGTAAATACTTGTCATGTTTGCCAGCAGAAAGGAGAAGCCATGCATGGACAGGTAAATACAGAAGTAGGGACATGGCAAATGGACTGTACCCACTTAGAGGGAAAAGTTGTCATCGTGGCAGTACATGTGGCCAGTGGCTTTATAGAAGCAGAAGTGATCCCGCAGGAAACTGGTAGGCAAACAGCCCTGTTCCTACTAAAGCTAGCAGGAAGGTGGCCTATCACTCATTTGCACACAGATAATGGTGCCAACTTCACTTCACAGGAAGTAAAGATGGTAGCATGGTGGGTAGGTATAGAGCATACCTTTGGAGTACCCTATAACCCGCAAAGCCAAGGAGTGGTAGAAGCCATGAATCACCATCTAAAGAACCAAATAGAGAGAATCAGAGAACAGGCAAATACAATGGAGACCATAGTCCTAATGGCAGCTCACTGCATGAATTTTAAAAGAAGGGGAGGAATAGGGGATATGACTCCTGCAGAAAGAATCATTAACATGATATCCACAGAACAAGAAATACAATTCCTCCAATCAAAAAATTCAAAATTTAAAAATTTTCGGGTCTATTACAGAGAAGGCAGAGACCAATTGTGGAAAGGCCCAGCAGACTTACTGTGGAAGGGAGAAGGAGCGGTCCTTATTAAGGTAGGGACAGACATTAAGGTAATACCCAGGAGAAAAGCAAAGATCATCAGAGATTATGGA

>AJ320484

TTTTTGGATGGAATAGATAAGGCTCAAGAAGAACATGAGAAATACCACAACAATTGGAGAGCAATGGCTAGTGATTTTAACCTGCCACCTGTGGTAGCGAAAGAAATAGTAGCTAGCTGTGATAAATGTCAGCTAAAAGGAGAAGCCATGCATGGACAAGTAGACTGTAGTCCAGGAATATGGCAATTAGATTGTACACATCTAGAAGGAAAAGTTATCCTGGTAGCAGTTCATGTAGCCAGTGGCTATATAGAAGCAGAAGTGATCCCAGCAGAAACAGGGCAGGAAACAGCCTACTTTATCTTAAAATTAGCAGGAAGATGGCCAGTAAAAGTAGTACATACAGACAATGGCAGCAATTTCACCAGTTCTGCAGTTAAGGCTGCCTGTTGGTGGGCAGGCATTAAGCAGGAATTTGGAATTCCCCACAATCCCCAAAGTCAAGGAGTAGTGGAATCTATGAATAAAGAATTAAAGAAAATTATAGGACAGGTAAGAGATCAAGCTGAACATCTTAAGACAGCAGTACAAATGGCAGTATTCATTCACAATTTTAAAAGAAAAGGGGGGATTGGGGAGTACAGTGCAGGGGAAAGAATAATAGACATAATAGCAACAGACATACAAACTACAAAATTACAAAAACAAATCATAAAAATTCAAAATTTTCGGGTTTATTACAGGGACAGCAGAGATCCAATTTGGAAAGGACCAGCAAAGCTTCTCTGGAAAGGTGAAGGGGCAGTAGTACTACAAGATAATAGTGACATAAAGGTAGTACCAAGAAGAAAAGTAAAGATCATTAGGGATTATGGAAAACAGATGGCAGGTGATGATTGTGTGGCAAGTAGACAGGATGAGGAT

>AF259954

TTTTTAGATGGGATAGATAAGGCTCAAGAAGAACATGAAAGATATCACAGCAATTGGAGAACAATGGTTAGTGATTTTAATTTGCCACCTATAGTAGCAAAGGAAATAGTAGCCAACTGTGATAAATGTCAACTAAAAGGGGAAGCTATGCATGGACAAGTAGACTGTAGTCCAGGGATATGGCAATTAGATTGCACACATCTAGAAGGAAAAGTCATCCTGGTAGCAGTCCACGTGGCCAGTGGATATATAGAAGCAGAAGTTATCCCAGCAGAAACAGGACAGGAGACAGCATACTTTCTGCTAAAATTAGCAGGAAGATGGCCAGTAAAAGTAATACACACAGACAACGGTAGCAATTTCACCAGCGCTGCAGTTAAAGCAGCCTGTTGGTGGGCCAATGTCCGACAGGAATTTGGGATCCCCTACAATCCCCAAAGTCAAGGAGTAGTAGAATCTATGAACAAGGAATTAAAGAAAATCATAGGGCAGGTAAGAGAGCAAGCTGAACACCTTAAGACAGCAGTACAAATGGCAGTATTCATTCACAATTTTAAAAGAAAAGGGGGGATTGGGGGGTACAGTGCAGGGGAAAGAATAATAGACATAATAGCAACAGACATACAAACTAAAGAATTACAAAAACAAATTACAAAAATTCAAAATTTTCGGGTTTATTACAGGGACAGCAGAGACCCAATTTGGAAAGGACCAGCAAAACTACTCTGGAAAGGTGAAGGGGCAGTAGTAATACAAGACAATAGTGATATAAAAGTAGTACCAAGAAGAAAAGCAAAGATCATTAGGGATTATGGAAAACAGATGGCAGGTGATGATTGTGTGGCAGGTAGACAGGATGAGGAT

>AF259955

TTTTTAGATGGGATAGATAAGGCTCAAGAAGAACATGAAAGATATCACAGCAATTGGAGAACAATGGTTAGTGATTTTAATTTGCCACCTATAGTAGCAAAGGAAATAGTAGCCAACTGTGATAAATGTCAACTAAAAGGGGAAGCTATGCATGGACAAGTAGACTGTAGTCCAGGGATATGGCAATTAGATTGCACACATCTAGAAGGAAAAGTCATCCTGGTAGCAGTCCACGTGGCCAGTGGATATATAGAAGCAGAAGTTATCCCAGCAGAAACAGGACAGGAGACAGCATACTTTCTGCTAAAATTAGCAGGAAGATGGCCAGTAAAAGTAATACACACAGACAACGGTAGCAATTTCACCAGCGCTGCAGTTAAAGCAGCCTGTTGGTGGGCCAATGTCCGACAGGAATTTGGGATCCCCTACAATCCCCAAAGTCAAGGAGTAGTAGAATCTATGAACAAGGAATTAAAGAAAATCATAGGGCAGGTAAGAGAGCAAGCTGAACACCTTAAGACAGCAGTACAAATGGCAGTATTCATTCACAATTTTAAAAGAAAAGGGGGGATTGGGGGGTACAGTGCAGGGGAAAGAATAATAGACATAATAGCAACAGACATACAAACTAAAGAATTACAAAAACAAATTACAAAAATTCAAAATTTTCGGGTTTATTACAGGGACAGCAGAGACCCAATTTGGAAAGGACCAGCAAAACTACTCTGGAAAGGTGAAGGGGCAGTAGTAATACAAGACAATAGTGATATAAAAGTAGTACCAAGAAGAAAAGCAAAGATCATTAGGGATTATGGAAAACAGATGGCAGGTGATGATTGTGTGGCAGGTAGACAGGATGAGGAT

>AY271690

TTTTTAGATGGCATAGATAAAGCCCAAGAAGAGCATGAAAAATATCACAGCAATTGGAGAGCAATGGCTAGTGATTTTAACCTGCCACCTATAGTAGCAAAAGAAATAGTGGCCAGCTGTGATAAATGTCAACTAAAAGGGGAAGCCATGCATGGACAAGTAGACTGTAGTCCAGGAATATGGCAATTAGATTGTACACATTTAGAAGGAAAAATTATCATAGTAGCAGTCCATGTAGCCAGTGGCTATATAGAAGCAGAAGTTATCCCAGCAGAAACAGGACAGGAGACAGCATACTTTATATTAAAATTAGCAGGAAGATGGCCAGTGAAAGTAATACACACAGATAATGGCAGTAATTTTACCAGTGCTGCAGTAAAGGCAGCATGTTGGTGGGCAAATGTCACACAAGAATTTGGAATTCCCTACAATCCCCAAAGTCAAGGAGTAGTAGAATCTATGAATAAAGAATTAAAGAAAATTATAGGGCAGGTCAGGGATCAAGCTGAACACCTTAAGACAGCAGTACAGATGGCAGTATTCATCCACAATTTTAAAAGAAAAGGGGGGATTGGGGGGTACAGTGCAGGGGAGAGAATAATAGACATAATAGCATCAGACATACAAACTAAAGAACTACAAAAACAAATTATACAAATTCAAAATTTTCGGGTTTATTACAGGGACAGCAGAGATCCAATTTGGAAAGGACCAGCAAAACTACTCTGGAAAGGTGAAGGGGCAGTAGTAATACAGGACAATAGTGATATAAAGGTAGTACCAAGAAGAAAAGCAAAAATCATTAGGGATTATGGAAAACAGATGGCAGGTAATGATTGTGTGGCAGGTAGACAGGATGAGGAT

>AJ519488

TTTTTGGATGGAATAAGTCAGGCTCAAGATGAACATGACAAATATCACAGCAATTGGAGAGCAATGGCTAGTGATTTTAACCTACCACCTGTGGTAGCAAAAGAAATAGTAGCTAGCTGTGATAAATGTCAAATAAAAGGAGAAGCCATGCATGGACAAGTAGACTGTAGTCCAGGAATATGGCAATTAGATTGTACACATCTAGAAGGACAAATTATCCTGGTAGCAGTTCATGTAGCCAGTGGCTATATAGAAGCAGAAGTTATTCCAGCAGAAACAGGGCAGGAAACAGCATACTTTCTTTTAAAATTAGCAGGAAGATGGCCAGTAAAAACGGTGCATACAGACAATGGCAGAAATTTTACCAGTGCTACTGTTAAGGCCGCCTGTTGGTGGGCAGGTATTAAGCAGGAATTTGGAATTCCCTACAATCCCCAAAGTCAAGGAGTAGTAGAATCTATGAATAAAGAATTAAAGAAAATTATAGAACAGGTAAGAGATCAAGCTGAACATCTTAAGACAGCAGTACAAATGGCAGTATTCATTCACAATTTTAAAAGAAAAGGGGGGATTGGGGGGTACAGTGCAGGGGAAAGAATAATAGACATAATAGCAACAGACATACAAACTAGAGAATTACAAAAACAAATCACAAAAATTCAAAATTTTCGGGTTTATTACAGGGACAGCAGAGATCCAATTTGGAAAGGACCAGCAAAGCTTCTCTGGAAAGGTGAAGGGGCAGTGGTAATACAAGAAAATAGTGACATAAAGGTAGTACCAAGAAGAAAAGCAAAGATCATTAGGGATTATGGAAAACAGATGGCAGGTGATGGTTGTGTGGCAAGTAGACAGGATGAGGAT

>AJ519489

TTTTTGGATGGGATAAGTCAGGCTCAAGAGGAACATGACAAATATCACAGCAATTGGAGAGCAATGGCTAGTGATTTTAACCTACCACCTGTGGTAGCAAAAGAAATAGTAGCTAGCTGTGATAAATGTCAACTAAAAGGAGAAGCCATGCATGGACAAGTAGACTGTAGTCCAGGAATATGGCAATTAGACTGTACACATCTAGAAGGAAAAATTATCCTGGTAGCAGTCCATGTAGCCAGTGGCTATATAGAAGCAGAAGTTATTCCAGCAGAGACAGGGCAGGAAACAGCATACTTTCTTTTAAAATTAGCAGGAAGATGGCCAGTAAAAGTAGTGCATACAGACAATGGCAGCAATTTTACTAGTGCTGCCGTTAAGGCCGCCTGTTGGTGGGCAGGTATTAAGCAGGAATTCGGAATTCCCTACAATCCCCAAAGTCAAGGAGTAGTGGAATCTATGAATAAAGAATTAAAGAAAATTATAGGACAAGTAAGAGATCAAGCTGAACATCTTAAGACAGCAGTACAAATGGCAGTATTCATTCACAATTTTAAAAGAAAAGGGGGGATTGGGGGGTACAGTGCAGGGGAAAGAATAATAGACATAATAGCAACAGACATACAAACTAGAGAATTACAAAAACAAATCATAAAAATTCAAAATTTTCGGGTTTATTACAGGGACAGCAGAGATCCAATTTGGAAAGGACCAGCAAAGCTTCTCTGGAAAGGTGAAGGGGCAGTAGTAATACAAGAAAATAGTGACATAAAAGTAGTACCAAGAAGAAAGGTAAAGATCATTAAGGATTATGGAAAACAGATGGCAGGTGATGGTTGTGTGGCAAGTAGACAGGATGAGGAT

>AF164485

TTTTTAGATGGGATAGATAAGGCTCAAGAAGAACATGAAAGATATCACCGCAATTGGAGAACAATGGCTAGTGATTTTAATTTGCCACCTATAGTAGCAAAGGAAATAGTAGCCAACTGTGATAAATGTCAACTAAAAGGGGAACCTATGCATGGACAAGTAGACTGTAGTCCAGGGATATGGCAATTAGATTGCACACATCTAGAAGGAAAAGTCATCCTGGTAGCAGTCCACCTGGCCAGTGGATATATAGAAGCAGAAGTCATCCCAGCAGAAACAGGACAGGAGACAGCATACTTTCTGCTAAAATTAGCAGGAAGATGGCCAGTAAAAGTAATACACACAGACAACGGTAGCAATTTCACCAGCGCTGCAGTTAAAGCAGCCTGTTGGTGGGCCAATGTCCGACAGGAATTTGGGATCCCCTACAATCCCCAAAGTCAAGGAGTAGTAGAATCTATGAATAAGGAATTAAAGAAAATCATAGGGCAGATAAGAGAGCAAGCTGAACACCTTAAGACAGCAGTACAAATGGCAGTATTCATTCACAATTTTAAAAGAAAAGGGGGGATTGGGGGGTACAGTGCAGGGGAAAGAATAATAGACATAATAGCAACAGACATACAAACTAAAGAATTACAAAAACAAATTACAAAAATTCAAAATTTTCGGGTTTATTACAGGGACAGCAGAGACCCCATTTGGAAAGGACCAGCAAAACTACTCTGGAAAGGTGAGGGGGCAGTAGTAATACAAGACAATAGTGATATAAAAGTAATACCAAGAAGAAAAGCAAAGATCATTAGGGATTATGGAAAACAGATGGCAGGTGATGATTGTGTGGCAGGTAGACAGGATGAGGAT

>AY125894

TTTTTAGATGGGATAGATAAGGCTCAAGAAGACCATGAAAGATATCACAGCAATTGGAGAACAATGGCTAGTGATTTTAATTTACCACCTATAGTAGCAAAGGAAATAGTAGCCAACTGTGATAAATGTCAACTAAAAGGGGAAGCTATGCATGGACAAGTGGACTGCAGTCCAGGGATATGGCAATTAGATTGCACACATCTAGAAGGAAAAGTCATCCTGGTAGCAGTCCACGTGGCCAGTGGATATATAGAAGCAGAAGTTATCCCAGCAGAAACAGGACAGGAGACAGCATACTTTCTGCTAAAATTAGCAGGAAGATGGCCAGTAAAAGTAATACACACAGACAACGGTAGCAATTTCACCAGCGCTGCAGTTAAAGCAGCCTGTTGGTGGGCCAATGTCCGACAGGAATTTGGGATCCCCTACAATCCCCAAAGTCAAGGAGTAGTAGAATCTATGAATAAGGAATTAAAGAAAATCATAGGGCAGATAAGAGAGCAAGCTGAACACCTTAAGACAGCAGTACAAATGGCAGTATTCATTCACAATTTTAAAAGAAAAGGGGGGATTGGGGGGTACAGTGCGGGGGAAAGAATAATAGATATAATAGCAACAGACATACAAACTAAAGAATTACAAAAACAAATTACAAAAATTCAAAATTTTCGGGTTTATTACAGGGACAGCAGAGGCCCAATTTGGAAAGGACCAGCAAAACTACTCTGGAAAGGTGAAGGGGCAGTAGTAATACAAGACAATAGTGATATAAAAGTAGTACCAAGAAGAAAAGCAAAGATCATTAGGGATTATGGAAAACAGATGGCAGGTGATGATTGTGTGGCAGGTAGACAGGATGAGGAT

>AY322184

TTTTTAGATGGAATAGATAAGGCTCAAGAAGAACATGAAAGATATCACAGCAATTGGAGAGCAATGGCTAGTGATTTTAATCTGCCACCTATACTAGCAAAGGAAATAGTAGCCAGCTGTGATAAATGTCAACTAAAAGGGGAAGCCATGCATGGACAAGTAGACTGTAGTCCAGGGATGTGGCAATTAGATTGCACACATCTAGAAGGAAAAGTAATTCTGGTAGCAGTCCATGTAGCCAGTGGCTATATAGAAGCAGAAGTTATCCCAGCAGAAACAGGACAGGAGACAGCATACTTTCTGCTAAAATTAGCAGGAAGATGGCCAGTAAAAGTAGTACACACAGACAATGGCAGCAATTTCACCAGCGCTGCATTTAAAGCAGCCTGTTGGTGGGCAGACGTCCAACAAGAATTTGGGATTCCCTACAATCCCCAAAGTCAAGGAGTAGTGGAATCTATGAATAAGGAACTAAAGAAAATCATAGGGCAGGTAAGAGAGCAAGCTGAACACCTTAAGACAGCAGTACAAATGGCAGTATTCATTCACAATTTTAAAAGAAAAGGGGGGATTGGGGGGTACAGTGCAGGCGAAAGAATAATAGACATAATAGCAACAGACATACAAACTAAAGAATTACAAAAGAACATTACAAAAGTTCAAAATTTTCGGGTTTATTACAGGGACAGCAGAGATCCACTTTGGAAAGGACCAGCAAAACTACTCTGGAAAGGTGAAGGGGCAGTAGTAATAAAGGACAATAGTGATATAAAGGTAGTGCCAAGAAGAAAAGCAAAGATCATCAGGGATTATGGAAAACAGATGGCAGGTGATGATTGTGTGGCAGGTAGACAGGATGAGGAT

>AY322185

TTTTTAGATGGAATAGATAAGGCTCAAGAAGAACATGAAAGATATCACAGCAATTGGAGAGCAATGGCTAGTGATTTTAATCTGCCACCTATACTAGCAAAGGAAATAGTAGCCAGCTGTGATAAATGTCAACTAAAAGGGGAAGCCATACATGGACAAGTGGACTGTAGTCCAGGGATGTGGCAATTAGATTGCACACATCTAGAAGGAAAAGTAATTCTGGTAGCAGTCCATGTAGCCAGTGGCTATATAGAAGCAGAAGTTATCCCAGCAGAAACAGGACAGGAGACAGCATACTTTCTGCTAAAATTAGCAGGAAGATGGCCAGTAAAAGTAGTACACACAGACAATGGCAGCAATTTCACCAGCGCTGCATTTAAAGCAGCCTGTTGGTGGGCAGATGTCCAACAAGAATTTGGGATTCCCTACAATCCCCAAAGTCAAGGAGTAGTGGAATCTATGAATAAGGAACTAAAGAAAATCATAGGGCAGGTAAGAGAGCAAGCTGAACACCTTAAGACAGCAGTACAAATGGCAGTATTCATTCACAATTTTAAAAGAAAAGGGGGGATTGGGGGGTACAGTGCAGGAGAAAGAATAATAGACATAATAGCAACAGACATACAAACTAAAGCATTACAAAAAAACATTACAAAAATTCAAAATTTTCGGGTTTATTACAGGGACAGCAGAGATCCACTTTGGAAAGGACCAGCAAAACTACTCTGGAAAGGTGAAGGGGCAGTAGTAATAAAGGACAATAATGATATAAAGGTAGTACCAAGAAGGAAAGCAAAGATCATTAGGGATTATGGAAAACAGATGGCAGGTGATGATTGTGTGGCAGGTAGACAGGATGAGGAT

>AY322189

TTCTTGGATGGGATAGATAAGGCTCAAGAAGAACATGAGAAATACCACAACAATTGGAGAGCAATGGCTAGTGATTTTAACCTGCCACCTGTGGTAGCAAAAGAAATAGTAGCTAGCTGTGATAAATGTCAACTAAAAGGAGAAGCCATGCATGGACAAGTAGACTGTAGTCCAGGAATATGGCAATTAGATTGTACACACTTAGAAGGAAAAGTTATCCTGGTAGCAGTCCATGTAGCCAGTGGCTATATGGAAGCAGAAGTTATTCCAGCAGAAACAGGACAGGAAACAGCCTACTTTATCTTGAAGTTAGCAGGAAGATGGCCAGTAAAAATAGTACATACAGACAATGGCAGCAATTTCACCAGCGCTGCAGTTAAGGCCGCATGTTGGTGGGCAGGTGTTAAGCAGGAATTTGGAATTCCCTACAATCCCCAAAGTCAAGGAGTAGTAGAATCTACGAATAAAGAGTTAAAGAAAATTATAGGACAGGTAAGAGATCAAGCTGAACATCTTAAGACAGCAGTACAAATGGCAGTATTTATCCACAATTTTAAAAGAAAAGGGGGGATTGGGGGGTACAGTGCAGGGGAAAGAATAATAGACATAATAGCATCAGACATACAAACTAAAGAATTACAGAAACAAATCATAAAAATTCAAAATTTTCGGGTTTATTACAGGGACAGCAGAGATCCAATTTGGAAAGGACCAGCAAAGCTTCTCTGGAAAGGTGAAGGGGCAGTAGTACTACAAGACAATAGTGAAATAAAGGTAGTACCAAGAAGAAAAGCAAAGATCATTAGGGATTATGGAAAACAGATGGCAGGTGATGATTGTGTGGCAAGTAGACAGGATGAGGAT

>AY322193

TTTTTAGATGGGATAGATAAAGCTCAAGAAGAACATGAAAGGTATCACAGCAATTGGAGGACAATGGCTAGTGATTTTAATCTGCCACCTATAGTAGCAAAGGAAATAGTAGCCAGCTGTGATAAATGTCAACTAAAAGGAGAAGCCATGCATGGACAAGTAGATTGTAGTCCAGGGATGTGGCAATTAGATTGCACACATCTAGAAGGAAAAGTAATTCTAGTAGCAGTCCATGTAGCCAGTGGCTATATAGAAGCAGAAGTTATCCCAGCAGAAACAGGACAGGAGGCAGCATACTTTCTGCTAAAATTAGCAGGAAGATGGCCAGTAAAAGTAGTACACACAGATAATGGCAGCAATTTCACCAGCGCTGCATTTAAAGCAGCCTGTTGGTGGGCAAATATCCAACAGGAATTTGGGATTCCCTACAATCCCCAAAGTCAAGGAGTGGTGGAATCTATGAATAAGGAATTAAAGAAAATCATAGGACAGGTAAGAGAGCAAGCTGAACATCTTAAAACAGCAGTACAAATGGCAGTATTCATTCACAATTTTAAAAGAAAAGGGGGGATTGGGGGGTACAGTGCAGGGGAAAGAATAATAGACATAATAGCAACAGACATACAAACTAAAGAATTACAAAAACATATTACAAAAATTCAAAATTTTCGGGTTTATTACAGGGACAGCAGAGATCCAATTTGGAAAGGACCAGCAAAACTACTCTGGAAAGGTGAAGGGGCAGTAGTAATACAGGACAATAGTGATATAAAGGTAGTACCAAGAAGAAAAGCAAAGATCATTAGGGATTATGGAAAACAGATGGCAGGTGATGATTGTGTGGCAAGTAGACAGGATGAGGAT

>AY322191

TTTTTAGATGGAATAGATAAAGCTCAAGAAGAACATGAAAGATATCACAGCAATTGGAGAACAATGGCTAGTGATTTTAATCTGCCACCTATAGTAGCAAAGGAAATAGTAGCCAGCTGTGATAAATGTCAGCTAAAAGGGGAAGCCATGCATGGACAAGTAGACTGCAGTCCAGGAATATGGCAATTGGATTGCACACATCTAGAAGGAAAAGTAATTCTGGTAGCAGTTCATGTAGCCAGTGGATATATAGAAGCAGAAGTTATCCCAGCAGAAACAGGGCAAGAGACAGCATACTTTATATTAAAATTAGCAGGAAGATGGCCAGTAAAAGTAGTACACACAGACAATGGCAGCAATTTCACCAGCGCTGCAGTTAAAGCAGCCTGTTGGTGGGCAGGTATCCAACAGGAATTTGGGATTCCCTACAATCCCCAAAGTCAAGGAGTAGTAGAATCCATGAATAAAGAATTAAAGAAAATCATAGGGCAGGTAAGAGATCAAGCTGAGCACCTTAAAACAGCAGTACAAATGGCAGTATTCATTCACAATTTTAAAAGAAAAGGGGGGATTGGGGGGTACAGTGCAGGGGAAAGAATAATAGACATAATAGCAACAGACATACAAACTAAAGAATTACAAAAACATATTACAAAAGTTCAAAATTTTCGGGTTTATTACAGGGACAGCAGAGATCCACTTTGGAAAGGACCAGCCAAACTACTTTGGAAAGGTGAAGGGGCAGTAGTAATACAGGACAATAGTGATATAAAGGTAGTACCAAGAAGGAAAGCAAAGATCATCAGGGACTATGGAAAACAGATGGCAGGTGATGATTGTGTGGCAAGTAGACAGGATGAGAAT

>AY322190

TTTTTAGATGGGATAGATAAAGCTCAAGAAGAACATGAAAGATATCACAGCAATTGGAGAACAATGGCTAGTGATTTTAATCTGCCACCTATAGTAGCAAAAGAAATAGTAGCCAGCTGTGATAAATGTCAACTAAAAGGGGAAGCCATGCATGGACAAGTAGACTGTAGTCCAGGGATATGGCAATTAGATTGCACACATCTAGAAGGAAAAGTAATTCTGGTAGCAGTCCATGTAGCCAGTGGCTATATAGAAGCAGAAGTTATCCCAGCAGAAACAGGACAGGAGGCAGCATACTTTCTGCTAAAATTAGCAGGAAGATGGCCAGTAAAAGTAGTACACACAGACAATGGCAGCAATTTCACCAGCGCTGCATTTAAAGCAGCTTGTTGGTGGGCAAGTATCCAACAGGAATATGGGATCCCCTACAATCCCCAAAGTCAAGGAGTAGTGGAATCTATGAATAAGGAATTAAAGAAAATCATAGGACAGGTAAGAGAGCAAGCTGAACACCTTAAAACAGCAGTACAAATGGCAGTATTCATTCACAATTTTAAAAGAAAAGGGGGGATTGGGGGGTACAGTGCAGGGGAGAGAATAATAGACATAATAGCAACAGACATACAAACTAAAGAATTACAAAAACAAATTACAAAAATTCAAAATTTTCGGGTTTATTACAGGGACAGCAGAGATCCAATTTGGAAAGGACCAGCAAAACTACTCTGGAAAGGTGAAGGGGCAGTAGTAATACAGGACAATAGTGATATAAAAGTAGTACCAAGAAGAAAAGCAAAGATCATTAGGGATTATGGAAAACAGATGGCAGGTGATGATTGTGTGGCAGGTAGACAGGATGAGGAT

>AY322187

TTCTTAGATGGAATAGATAAGGCTCAAGAAGAACATGAGAAATACCACAACAATTGGAGAGCAATGGCTAGTGATTTTAACCTGCCACCTGTGGTAGCAAAAGAAATAGTAGCTAGCTGTGATAAATGCCAGCTAAAAGGAGAAGCCATACATGGACAAGTAGACTGTAGTCCAGGAATATGGCAATTAGATTGTACACATTTAGAAGGAAAAATTATTCTGGTAGCAGTCCATGTAGCCAGTGGCTATATAGAAGCAGAAGTTATTCCAGCAGAAACAGGACAAGAAACAGCCTATTTTCTCTTGAAATTAGCAGGAAGATGGCCAGTAAAAGTAGTACATACAGACAATGGCAGCAATTTCACCAGCGCGGCAGTTAAGGCCGCCTGTTGGTGGGCAGGCATCAAGCAGGAATTTGGAATTCCCTACAATCCCCAAAGCCAAGGAGTAGTAGAATCTATGAATAAAGAATTAAAAAAGATTATAGGACAGGTAAGAGATCAAGCTGAACATCTTAAGACAGCAGTACAAATGGCAGTATTCATCCACAATTTTAAAAGAAAAGGGGGGATTGGGGGGTACAGTGCAGGGGAAAGAATAATAGACATAATAGCAACAGACATACAAACTAAAGAATTACAAAAACAAATTATAAAAATTCGAAATTTTCGGGTTTATTACAGGGACAGCAGAGATCCAATTTGGAAAGGACCAGCAAAGCTCCTCTGGAAAGGTGAAGGGGCAGTAGTAATACAAGACAATAGTGAAATAAAGGTAGTACCAAGAAGGAAAGCAAAGATCATTAGGGATTATGGAAAACAGATGGCAGGTGATGATTGTGTGGCAAGTAGACAGGATGAGAAT

>AY169803

TTCCTAGAAGGAATAGACCAGGCACAGGAAGATCATGAAAAATATCATAGCAATTGGAGAGCATTAGCTAGTGAATTTGGACTGCCACCAATAGTAGCCAAAGAAATTATTGCTAATTGTCCTAAATGYCATATAAAAGGGGAAGCAATGCATGGTCAAGTAGACTGCAGTCCAGAAGTATGGCAAATGGACTGCACACATGCAGAAGGCAAAGTCATAATAGTTGCTGTCCATGTGGCAAGTGGGTTCATAGAAGCAGAAGTGATACCAGCAGAAACAGGACAAGAAGCTGCCTATTTCCTGTTAAAATTAGCAGCAAGATGGCCTATTAAAATATTACATACAGACAATGGGCCTAATTTCACAAGTGCAACCATGAAAGCTGCATGTTGGTGGGCCAACATAAAACATGAGTTTGGAATACCATATAACCCACAAAGTCAAGGAGTAGTAGAAGCCATGAATAAGGAATTAAAGTCAATCATACAACAAGTGAGGGACCAAGCAGAGCACTTAAGAACAGCAGTACAGATGGCAGTATTTATTCACAATTTTAAAAGAAAAGGGGGGATTGGGGGGTACACTGCAGGAGAAAGGTTAATAGACATATTAGCATCACAAATACAAACAACAGAACTACAAAAACAAATTTTAAAAATTCAAAATTTTCGGGTCTATTACAGAGACAGCAGAGACCCTATTTGGAAAGGACCGGCCCAACTCCTGTGGAAAGGTGAGGGAGCAGTAGTCATACAAGATAAAGGAGACATTAAGGTAGTACCAAGRAGAAAGGCAAAAATAATCAGGCATTATGGAAAACAGATGGCAGGTGCTGATAGTRTGGCAAGTGGACAGRCAGAA

>AY169806

TTCCTAGAAGGAATAGATCAGGCACAAGAAGATCATGAAAAATATCATAGCAATTGGAGAGCATTGGCTAGTGACTTTGGACTACCACCAGTAGTAGCCAAAGAAATTATTGCTAGCTGTCCTAAATGTCATATAAGAGGGGAAGCAATACATGGCCAGGTAGACTGCAGTCCAGAAGTATGGCAAATGGATTGTACACATTTAGAAGGCAAAATCATAATAGTAGCTGTCCATGTGGCAAGTGGGTTCATAGAAGCAGAGGTGATACCAGCAGAGACAGGACAGGAAACTGCCTATTTCCTGTTAAAATTAGCAGCAAGATGGCCTGTTAAAATAATACATACAGACAATGGGCCTAATTTTACAAGTGCAGCCATGAAAGCTGCATGCTGGTGGACAGGCATACAACATGAGTTTGGAATACCATATAACCCACAAAGTCAAGGAGTAGTAGAAGCCATGAATAAGGAATTAAAATCAATTATAGGGCAGGTGAGGGACCAAGCAGARCACTTAAAGACAGCAGTACAAATGGCAGTATTTGTTCACAATTTTAAAAGAAAAGGGGGGATTGGGAGGTATACTGCAGGAGAAAGATTAATAGACATATTAGCATCACAAATACAAACAACAGAATTACAAAAACAAATTTCAAAAATTCAAAATTTTCGGGTCTATTACAGAGACAGCAGAGACCCTATTTGGAAAGGACCGGCACAGCTCCTGTGGAAAGGTGAAGGAGCAGTAGTCATACAAGATAAAGGAGACATTAAGGTAGTACCAAGAAGAAAGGCAAAAATAATCAGGGATTATGGAAAACAGATGGCAGGTACTGATTGTATGGCAAGTGGACAGACAGAA

>AY169810

TTCCTAGAAGGAATAGATCARGCACAGGAAGATCATGAAAAATATCACAGCAATTGGAGAGCATTAGCTAGTGACTTTGGACTACCACCAGTAGTAGCCAAAGAAATTATTGCTAGTTGTCCTAAATGTCATATTAAAGGGGAAGCAATTCATGGTCAGGTAGATTACAGTCCAGAAATATGGCAAATGGATTGCACACATTTAGAAGGCAAAGTCATAATAGTTGCTGTCCATGTGGCAAGTGGATTCATAGAAGCAGAAGTGATACCAGCAGAAACAGGACAAGAAACTGCCTATTTCCTGTTAAAATTAGCAGCAAGATGGCCTATTAGAGTACTACATACAGACAATGGGCCTAATTTTACAAGTGCAACCATGAAGGCTGCATGTTGGTGGGCCAACATAAGACATGAGTTTGGAATACCATATAACCCACAAAGTCAAGGAGTAGTAGAAGCCATGAATAAGGAATTAAAGTCAATCATACAGCAGGTGAGGGACCAAGCAGAGCATTTAAAAACAGCAGTACAAATGGCAGTATTTGTTCACAATTTTAAAAGAAAAGGGGGGATTGGGGGGTGCACTGCAGGAGAGAGATTAATAGATATGCTAGCATCACAAATACAAACAACAGAACTACAAAAACACATTTTAAAAATTCAAAAATTTCAGGTCTATTACAGAGACAGCAGAGACCCTATTTGGAAAGGACCGGCACAACTCCTGTGGAAAGGTGAGGGAGCAGTAGTCATACAAGATAAAGGAGATATCAAGGTAGTACCAAGAAGAAAAGCAAAAATACTCAGAGATTATGGAAAACAGATGGCAGGTGCTGATAGTATGGCAAGTGGACAGACAGAR

>AY169813

TTCCTGGAAGGAATAGATCAGGCACAAGAAGATCATGAAAAATATCACAGCAATTGGAGAGCATTAGCTAGTGACTTTGGACTACCACCAGTAGTAGCCAAAGAAATTATTGCTAGTTGTCCTAAATGTCATACTAAAGGGGAAGCAATTCATGGTCAGGTAGATTACAGTCCAGAGATATGGCAAATGGATTGCACACATTTAGAAGGCAAAGTCATAATAGTTGCTGTCCATGTGGCAAGTGGATTCATAGAGGCAGAAGTGATACCAGCAGAAACAGGACAAGAAACTGCCTATTTCCTGTTAAAATTAGCAKCAAGATGGCCTATTAAAATATTACATACAGATAATGGGCCTAATTTTACAAGTGCAACCATGAAGGCTGCATGTTGGTGGGCYAACATAAAACATGAGTTTGGCATACCATATAACCCACAAAGTCAAGGGGTAGTAGAAGCCATGAACAAGGAATTAAAGTCAATTATACAGCAGGTGAGGGACCAAGCAGAGCATTTAAAAACAGCAGTACAAATGGCAGTATTTGTTCACAATTTTAAAAGAAAAGGGGGGATTGGGGGGTGTACTGCAGGAGAGAGATTAATAGATATGTTAGCATCACAAATACAAACAACAGAACTACAAAAACAAATTTTAAAAATTCAAAAATTTCGGGTCTATTACAGAGACAGCAGAGACCCTATTTGGAAAGGACCGGCACAACTCCTGTGGAAAGGTGAGGGAGCAGTAGTCATACAAGATAAAGGAGATATCAAGGTAGTACCAAGAAGAAAAGCAAAAATACTCAGGTGTTATGGAAAACAGATGGCAGGTGCTGATAGTATGGCAAGTGGACAGACAGAA

>AY169805

TTCCTAGARGGAATAGACCAGGCACAGGAAGATCATGAAAAATATCATAGCAATTGGAGAGCATTAGCTAGTGACTTTGGACTACCACCAGTGGTGGCCAAGGAAATCATTGCTAGCTGTCCTAAATGTCATATAAAAGGAGAAGCAATACATGGTCAGGTAGACTGCAGTCCAGAAGTATGGCAGATAGATTGCACACATGTAGAAGGCAAAGTCATAATAGTTGCTGTCCATGTGGCAAGTGGATTCATAGAAGCAGAAGTAATACCAGCAGAAACAGGACAGGAAACTGCTTACTTCCTGTTAAAATTAGCTGCAAGATGGCCTGTTAAGATAATACATACAGACAATGGGCCTAATTTTACAAGTGCAACTATGAAGGCTGCATGTTGGTGGACTAACATACAACATGAGTTTGGAATACCATATAATCCACAAAGTCAAGGAGTAGTAGAAGCCATGAATAAGGAATTAAAATCAATYATACAGCARGTGAGGGACCAAGCAGAACACTTAAGAACAGCAGTACAAATGGCAGTATTTGTTCACAATTTTAAAAGAAAAGGGGGGATTGGGGGGTACACTGCAGGAGAGAGGATAATAGACATACTAGCATCACACATACAAACAACAGAATTACAAAAACAAATTTTAAAAATTCAAAATTTTCGGGTCTATTACAGAGACAGCAGAGACCCTATTTGGAAAGGACCGGCCCAGCTCCTGTGGAAAGGTGAGGGAGCAGTAGTCATACAAGATAAAGGAGACATTAAAGTAGTACCAAGAAGAAAGGCAAAAATAATCAGAGATTATGGAAAACAGATGGCAGGTACTGATAGTATGGCAAGTAGACAGACAGAA

>AY169809

TTCCTAGAAGGAATAGACCAGGCACAAGAAGATCATGAGAAATACCATAGCAATTGGAGAGCATTAGCCAGTGACTTTGGACTACCACCAGTGGTGGCCAAGGAAATTATTGCTAATTGTCCTAAATGTCATATAAAGGGGGAAGCAATTCATGGTCAGGTAGACTGCAGTCCAGAAGTATGGCAAATGGATTGCACACATGTAGAAGGCAAAGTTATCATAGTTGCTGTCCATGTGGCAAGCGGATTCATAGAAGCAGAAGTGATACCAGCAGAAACAGGACAGGAAACTGCCTATTTCCTGTTAAAATTAGCAGCAAGATGGCCTGTTAAAGTAATACATACAGACAACGGGCCTAATTTTACAAGTGCAACCATGAAAGCTGCATGTTGGTGGACTAACATACAACATGAGTTTGGAATACCATACAATCCACAAAGTCAAGGAGTAGTAGAAGCCATGAATAAGGAATTAAAATCAATTATACAGCAAGTGAGGGACCAAGCAGAGCACTTAAGGACAGCAGTACAAATGGCAGTATTTGTTCACAATTTTAAAAGAAAAGGGGGGATTGGGGGGTACACTGCAGGAGAGAGATTAATAGACATATTAGCATCACAAATACAAACAACAGAACTACAAAAACAAATTTTGAAAATTCAAAAATTTCGGGTCTATTACAGAGACAGCAGAGACCCTATCTGGAAAGGACCGGCACAGCTCCTGTGGAAAGGTGAGGGAGCAGTAGTCATACAAGACAAAGGAGACATTAAGGTAGTACCAAGAAGAAAGGCAAAAATACTCAGGGACTATGGAAAACAGATGGCAGGTACTGATAGTATGGCAAGTGGACAGGCAGAA

>AY169804

TTCCTGGAGGGAATAGAYCAGGCACAAGAAGATCATGAAAAATATCATAGTAATTGGAGAGCATTAGCTAGTGACTTTGGACTGCCACCAGTAGTAGCAAAGGAAATCATTGCTAGCTGTCCTAAATGCCATATAAAAGGGGAAGCAACACATGGTCAAGTAGACTGCAGCCCAGAGATATGGCAGATGGATTGTACACATTTAGAAGGCAAAATCATAATAGTTGCTGTCCATGTAGCAAGTGGCTTCATAGAAGCAGAGGTGATTCCAGCAGAATCAGGACAGGAAACTGCCTATTTCCTGTTAAAATTAGCAGCAAGATGGCCTGTCAAAGTAATACATACAGACAATGGGCCTAATTTTACAAGTGCAGCCATGAAAGCTGCATGTTGGTGGACAGGCATACAACATGAGTTTGGGATACCATATAATCCACAAAGTCAAGGAGTAGTAGAATCCATGAATAAAAATTTAAAATCCATTATACAGCAAGTGAGGGACCAAGCAGAGCATTTAAAAACAGCAGTACAAATGGCAGTCTTTGTTCACAATTTTAAAAGAAAAGGGGGGATTGGGGGGTACACTGCAGGGGAAAGACTAATAGACATACTAGCATCACAAATACAAACAACAGAACTACAAAAACAAATTTTAAAAATTCAAAATTTCCGGGTTTATTACAGAGATAGCAGAGATCCTATTTGGAAAGGACCGGCACAACTCCTGTGGAAAGGTGAGGGGGCAGTAGTCATACAAGATAAAGGAGACATTAAAGTAGTACCAAGAAGAAAGGCAAAAATAATCAGGGATTATGGAAAACAGATGGCAGGTAATGATAGTATGGCAAGTAGACAGACAGAA

>AY169807

TTCCTAGAAGGAATAGACCAGGCACAGGAAGATCATGAAAAATATCATAGCAATTGGAGAGCATTAGCTAGTGACTTTGGACTACCACCAGTGGTGGCCAAGGAAATCATTGCTAGCTGTCCTAAATGTCATATAAGAGGAGAAGCAATGCATGGTCAGGTAGACTGCAGTCCAGAAGTATGGCAGATAGATTGCACACATGTAGAAGGTAAAGTCATAATAGTTGCTGTCCATGTGGCAAGTGGATTCATAGAAGCAGAAGTAATACCAGCAGAAACAGGGCAAGAAACTGCTTACTTCCTGTTAAAACTAGCTGCAAGATGGCCTGTTAAGGTAATACATACAGACAATGGGCCTAATTTTACAAGTGCAACTATGAAAGCTGCATGTTGGTGGACTAACATACAACATGAGTTTGGAATACCATATAATCCACAAAGTCAAGGAGTAGTAGAAGCCATGAATAAAGAATTAAAATCAATTATACAGCAAGTGAGGGACCAAGCAGAACATTTAAAAACAGCAGTACAAATGGCAGTATTTGTTCACAATTTTAAAAGAAAAGGGGGGATTGGGGGGTACACTGCAGGTGAGAGGATAATAGACATACTAGCATCACACATACAAACAACAGAACTACAAAAACAAATTTCAAAAATTCAAAATTTTCGGGTCTATTACAGAGACAGCAGAGACCCCATTTGGAAAGGACCGGCCCAGCTCCTGTGGAAAGGTGAGGGAGCAGTAGTCATACAAGATCAAGGAGACATTAAAGTAGTACCAAGAAGGAAGGCAAAAATAATCAGAGATTATGGAAAACAAATGGCAGGTACTGATAGTATGGCAAGTAGACAGACAGAA

>AY169811

TTCCTGGAAGGAATAGACCAGGCACAAGAAGATCATGAAAAATATCATAGTAATTGGAGAGCATTAGCTAGTGAATTTGGACTACCACCAGTGGTGGCTAAGGAAATCATTGCTAGCTGTCCTAAGTGTCATATAAGAGGGGAAGCAATTCATGGCCAGGTAGACTGCAGTCCAGAAGTGTGGCAAATGGATTGCACACATCTAGAAGGCAAAATCATAATAGTTGCTGTCCATGTGGCAAGTGGATTCATAGAAGCAGAGGTAATACCAGCAGAAACAGGACAAGAAACTGCCCACTTCCTGTTAAAACTAGCTGCAAGATGGCCTGTTAAAGTAYTACATACAGACAATGGGCCTAATTTTACAAGTGCAACTATGAAGGCTGCATGTTGGTGGACCAACATAAAACATGAGTTTGGAATACCATATAATCCACAAAGTCAAGGAGTAGTRGAAGCCATGAATAAGGAATTAAAATCAATTATACAACAGGTGAGGGACCAAGCAGAACAYTTAAGGACAGCRGTACAAATGGCAGTATTTGTTCACAATTTTAAAAGAAAAGGGGGGATTGGGGGGTACACTGCAGGAGAAAGGATAATAGACATATTAGCATCACACATACAAACAACAGAATTACAAAAACAAATTTTAAAAATTCAAAATTTTCGGGTCTATTACAGAGACAGCAGAGACCCTATTTGGAAAGGACCGGCACAGCTCCTGTGGAAAGGTGARGGAGCAGTAGTCATACAAGATAAGGGAGACATTAAAGTAGTGCCAAGAAGAAAAGCAAAAATAATCAGAGATTATGGAAAACAGATGGCAGGTRCTGATAGTATGGCAAGTGGACAGACAGAA

>AY169802

TTCCTAGAAGGGATAGATCAGGCACAGGAAGATCATGAAAAATATCATAGCMATTGGAGAGCATTAGCTAGTGACTTTGGACTGCCACCAGTAGTGGCCAAAGAAATTATTGCTAATTGTCCTAAATGTCATATAAAAGGGGAAGCMATTCATGGGCAAGTAGACTGCAGTCCAGAAGTATGGCAAATGGATTGCACACATACAGAAGGCAAAGTCATAATAGTAGCTGTCCATGTGGCAAGTGGGTTCATAGAAGCAGAAGTAATACCAGCAGAAACAGGACAAGAAACTGCCTATTTCCTGTTAAAACTAGCAGCAAGATGGCCTGTTAAAGTAATACATACAGACAATGGGCCTAATTTCACAAGTGCAACCATGAAGGCTGCATGTTGGTGGGCCAACATAWAACATGAGTTTGGAATACCATATAATCCACAAAGTCAAGGAGTAGTAGAAGCCATGAATAAGGAATTAAAGTCAATCATAGAACAAGTGAGGGACCAAGCAGAGCACTTAAGAACAGCAGTACAAATGGCAGTATTCATTCACAATTTTAAAAGAAAAGGGGGGATTGGGGGGTACACTGCAGGAGAAAGGTTAATAGACATATTAGCATCACAAATACAAACAACAGAACTACAAAAACAAATTTTAAAAATTCAAAATTTTCGGGTCTATTACAGAGACAGCAGAGACCCTATTTGGAAAGGACCGGCACAACTCCTGTGGAAAGGTGAGGGAGCAGTAGTCATACAAGATAAAGGAGACATTAAAGTAGTACCAAGAAGAAAGGCAAAAATAATCAGGCATTATGGAAAACAGATGGCAGGTGCTGATAGTATGGCAAGTGGACAGACAGAA

>AY169812

TTCYTAGAAGGAATAGACCAGGCACAAGAAGATCATGAGAAATATCATAGCAATTGGAAAGCATTAGCTAGTGACTTTGGGCTACCACCAGTAGTGGCCAAAGAAATCATTGCTAGTTGTCCTAAATGTCATATAAAAGGAGAAGCAATGCATGGTCAGGTCGACTGCAGTCCAGAAGTATGGCAAATAGATTGCACACATTTAGAAGGCAAGGTCATAATAGTTGCTGTCCATGTGGCAAGTGGATTCATAGAAGCAGAAGTAATACCAGCTGAAACAGGACAAGAAACTGCCTACTTCCTGTTAAAATTAGCTGCAAGGTGGCCTGTTAAAATTATACATACAGACAACGGGCCTAATTTTACAAGTGCAACCATGAAAGCTGCATGTTGGTGGACCAACATACAACATGAGTTTGGAATACCTTATAATCCACAAAGTCAAGGAGTAGTAGAAGCCATGAATAAGGAATTAAAATCAATTATACAGCAGGTGAGGGACCAAGCAGAACACTTAAGAACAGCAGTACAAATGGCAGTATTTGTTCACAATTTTAAAAGAAAAGGGGGGATTGGGGGGTACACTGCAGGAGAAAGGATAATAGACATACTAGCATCACAAATACAAACAACAGAATTACAAAAACAAATTTTTAAAATTCAAAAATTTCAGGTCTATTACAGAGACAGCAGAGACCCTATTTGGAAAGGACCGGCACAGCTCCTGTGGAAAGGTGAGGGAGCAGTAGTCATACAAGATAAAGGAGAAATTAAAGTAGTACCAAGAAGAAAGGCAAAAATAATCAGACATTATGGAAAACAGATGGCAGGTGCTGATAGTATGGCAAGTRGACAGACAGAA

>AY169816

TTCTTAGAAGGAATAGACCAGGCACAAGAAGATCATGAAAAGTATCATAGTAATTGGAGAGCATTAGCTAGTGAATTTGGACTACCACCAGTAGTGGCCAAGGAAATCATTGCTAGCTGTCCTAAATGTCATATAAAAGGGGAAGCAATGCATGGTCAGGTAGACTGCAGTCCAGAAGTATGGCAGATGGATTGCACACATCTAGAAGGCAAAATCATAATAGTTGCTGTCCATGTGGCAAGTGGATTCATAGAAGCAGAAGTAATACCAGCAGAAACAGGACAAGAAACTGCCTACTTCCTGTTAAAACTAGCTGCAAGATGGCCTGTTAAAATATTACACACAGACAATGGGCCTAATTTTACAAGTGCAACTATGAAGGCTGCATGTTGGTGGGCCAACATACAACATGAGTTTGGAATACCATATAATCCACAAAGCCAAGGAGTAGTAGAAGCCATGAATAAGGAATTAAAATCAATTATAGGACAGGTGAGGGACCAAGCAGAACACTTAAGAACAGCAGTACAAATGGCAGTATTTGTTCACAATTATAAAAGAAAAGGGGGGATTGGGGGGTACACTGCAGGAGAGAGGATAATAGACATATTAGCAACACAATTACAAACAACAGAATTACAAAAACAAATTTTAAAAATTCAAAATTTTCGGGTCTATTACAGAGACAGCAGAGACCCTATTTGGAAAGGACCGGCACAGCTCCTGTGGAAAGGTGAGGGAGCAGTAGTCATACAAGAYAAGGGAGACATTAAGGTAGTACCAAGAAGGAAGGCAAAAATAATCAGAGATTATGGAAAACAGATGGCAGGTACTGATAGTATGGCAAGTGGACAGACAGAA

>AY169808

TTCCTRGAAGGAATAGACCAGGCACAAGAAGATCATGAAAAATATCATAGCAATTGGAGAGCATTAGCTAGTGATTTTGGACTACCACCAGTAGTGGCCAAAGAAATCATTGCTAATTGTCCTAAATGTCATATAAAAGGGGAAGCAACTCATGGTCAGGTAGACTACAGTCCAGAAGTATGGCAAATAGATTGCACACATTTAGAAGGYAAAATCATAATAGTTGCTGTCCATGTGGCAAGTGGATTCATAGAAGCAGAAGTGATACCAGCAGARTCAGGACAAGAGACTGCCTACTTCCTGTTAAAGTTAGCTGCAAGATGGCCTGTTAAAATAATACATACAGACAACGGGCCTAATTTTACAAGTGCAATCATGAAGGCTGCCTGTTGGTGGACCAACATAAAACATGAGTTTGGAATACCATATAATCCACAAAGTCAAGGAGTAGTAGAGGCCATGAATAAGGAATTAAAATCAATTATACAGCAGGTGAGAGACCAAGCAGAACACTTAAGAACAGCAGTACAAATGGCAGTATTTGTTCACAATTTTAAAAGAAAAGGGGGATTGGGGGGGTACACTGCAGGAGAAAGGATAATAGACATATTAGCATCACAAATACAAACAACAGAATTACAAAAACAAATTTTTAAAATTCAAAAATTTCAGGTCTATTACAGAGACAGCAGAGATCCTATTTGGAAAGGACCGGCACAACTCCTGTGGAAAGGTGARGGAGCAGTAGTCATACAAGATAAAGGAGACATYAAAGTAGTACCAAGAAGAAAGGCAAAAATAATCAGACATTATGGAMAACAGATGGCAGGTCCTGATAGTATGGCAAGTGGACAGACAGAA

>AY169814

TTCCTAGAAGGAATAGATCAGGCGCAGGAAGATCATGAAAAGTATCATAGCAATTRGAGAGCATTAGCCAGTGATTTTGGATTACCACCAGTAGTAGCCAAGGAAATCATTGCTAGTTGTCCTAAATGTCATATAAGAGAGGAACCAATGCATRGTCAGGTAGACTGTAGTCCAGAAATATGGCAAATGGACTGCACACATTTAGAAGGCAAGATCATAATAGTTGCTGTCCATGTAGCAAGTGGATTCATAGAAGCAGAAGTGATACCAGCAGAAACAGGACAAGAAACTGCCTATTTCCTGTTAAAATTAGCAGCAAGATGGCCTGTTAAAATCATACATACAGATAATAGGCCTAATTTTACAAGTGCAACCATGAAAGCTGCATGTTGGTGGACAGGCATACAACATGAGTTTRGAATACCATACAATCCACAAAGTCAAGRAGTAGTAGAAGCCATGAATAAGGAATTAAAATCAATTATACAACAGGTGAGGGACCAAGCAGAGCACTTAAAAACAGCAGTACAAATGGCAGTATTTGTTCACAATTTTAAAAGAAAAGGGGGGATTGGGGGGTACACTGCAGGAGAGAGATTAATAGACATATTAGCATCACAAATACAAACAACAGAACTACAAAAACAAATTTTAAAAATTCAAAATTTTCGGGTCTATTACAGAGACAGCAGAGACCCTATTTGGAAAGGACCGGCACAGCTCCTGTGGAAAGGTGAGGGAGCAGTAGTCATACAAGATAAAGGAGAAATTAAGGTAGTACCAAGAAGAAAGGCAAAAATAATTAGGGATTATGGAAAACAGATGGCAGGTACTGATTGTATGGCAAGTGGACAGACAGAA

>AY169815

TTCCTAGAAGGKATAGACCAGGCACAAGAAGATCATGAAAAGTATCATAGCAATTGGAGAGCATTAGCTAGTGACTTTGGACTACCACCAGTGGTAGCCAAGGAAATCATTGCTAATTGTCCTAAATGTCATATAAAAGGGGAAGCAATTCATGGTCAGGTAGACTGCAGTCCAGAAGTATGGCAAATAGATTGCACACATACAGAAGGCAAAGTCATAATAGTTGCTGTTCATGTGGCAAGTGGGTTTATAGAAGCAGAAGTGATACCAGCAGAAACAGGACAAGAAACTGCCTACTTCCTGTTAAAATTAGCTGCAAGATGGCCTGTTAAAGTAATACATACAGACAACGGGCCTAATTTCACAAGTGCAACCATGAAGGCTGCATGTTGGTGGACCAACATAAAACATGAGTTTGGAATACCATATAATCCACAAAGTCAAGGAGTAGTAGAAGCCATGAACAAGGAATTAAAATCAATAATACAGCAGGTGAGGGACCAAGCAGAACACTTAAGAACAGCAGTACAAATGGCAGTATTTGTTCACAATTTTAAAAGAAAAGGGGGGATTGGGGGGTACACTGCAGGAGAAAGGATAATAGACATATTAGCATCACAAATACAAACAACAGAATTACAAAAACAAATTTTCAAAATTCAAAAATTTCAGGTCTATTACAGAGACAGCAGAGATCCTACTTGGAAAGGACCGGCACAGCTCCTGTGGAAGGGTGAAGGAGCAGTAGTCATACAAGATAAAGGAGACATTAAGGTAGTACCAAGAAGGAAGGCAAAAATAATTAGAAATTATGGAAAACAGATGGCAGGTACTGATAGTATGGCAAGTGGACAGRCAGAA

>AY532635

TTCCTAGATGGTATAGAAAAAGCCCAAGAAGATCATGARAGATATCACAGTAACTGGAGAGCAATGGCCAGTGATTTTAACTTACCCTCTGTAGTGGCAAAAGAAATAGTAGCCAGCTGTGACAAATGCCAGCTAAAAGGGGAAGCCATACATGGACAGGTCAATTGTAGTCCAGGAGTGTGGCAGCTAGATTGTACACATTTAGAAGGAAAAATCATTCTTGTAGCGGTCCATGTGGCCAGTGGCTACTTAGAAGCAGAAGTTATTCCTGCAGAAACAGGACAGGAAACAGCATATTTTATTTTAAAATTAGCTGGAAGATGGCCAGTAAAAGTTATACACACTGATAATGGACCCAATTTCACTAGTGCCACTGTAAAAGCAGCCTGTTGGTGGGCAAATATCACACAGGAATTCGGGATACCCTACAATCCTCAAAGTCAGGGAGTAGTAGAATCCATGAATAAAGAATTAAAGAAAATTATAGGACAAATCAGAGATCAAGCAGAACATCTAAAAACAGCAGTGCAAATGGCGGTGTTCATTCACAATTTTAAAAGAAAAGGGGGGATTGGGGAGTACACTGCAGGGGAAAGAATAATAGACATAATAGCAACAGACATACAAACAACAAAATTACAAACACAAATTTTAAAGATTCAAAATTTTCGGGTTTATTACAGAGACAGCAGAGATCCCATTTGGAAAGGACCAGCCAAACTTCTGTGGAAAGGAGAAGGGGCAGTGGTAATCCAAGATAACGGAGATATAAAAGTAGTCCCACGTAGGAAAGCAAAAATAATTAGGGATTATGGAAAACAGATGGCAGGTGATGGTTGTGTGGCAAGTGGACAGGCGGAGAAT

>AY093605

TTTTTAGATGGCATAGATAAAGCTCAAGAAGAACATGAAAGATATCATAGCAATTGGAGAGCAATGGCTAGTGATTTTAATCTGCCACCTGTAGTAGCAAAAGAAATAGTGGCCAGCTGTGATAAATGTCAGCTAAAAGGGGAAGCCATGCATGGACAAGTAGACTGTAGTCCAGGAATATGGCAATTAGATTGTACACATTTAGAAGGAAAAATTATTCTGGTAGCAGTCCATGTAGCCAGTGGTTATATAGAAGCAGAAGTTATCCCAGCAGAAACAGGACAAGAAACAGCATACTTTATATTAAAATTAGCAGGAAGATGGCCAGTAAAAGTGATACACACGGACAATGGCAGTAATTTCACCAGTGCTGCAGTAAAGGCAGCATGTTGGTGGGCAAATGTCACACAAGAATTTGGAATTCCCTACAATCCCCAAAGCCAAGGAGTAGTGGAATCTATGAATAAAGAATTAAAGAAAATCATAGGGCAGGTCAGGGATCAAGCTGAACACCTTAAGACAGCAGTACAGATGGCAGTATTCATTCACAATTTTAAAAGAAAAAGGGGGATTGGGGGGTACAGTGCAGGGGAAAGAATAATAGACATAATAGCATCAGATATACAAACTAAAGAACTACAAAAACAAATCACAAAAATTCAAAATTTTCGGGTTTATTACAGGGACAGCAGAGACCCAATTTGGAAAGGACCAGCAAAACTACTCTGGAAAGGTGAAGGGGCAGTAGTAATACAGGACAATAATGATATAAAGGTAGTACCAAGAAGAAAAGCAAAGATCATTAGGCATTATGGAAAACAGATGGCAGGTGATGATTGTGTGGCAGGTAGACAGGATGAGGAT

>AY093603

TTTTTAGATGGTATAGATAAAGCCCAAGAAGAGCATGAAAGATATCACAGCAACTGGAGAGCAATGGCTAGTGATTTTAATCTGCCACCTATAGTAGCAAAAGAAATAGTAGCCAGCTGTGATAAATGTCAGCTAAAAGGGGAAGCCGTGCATGGACAAGTAGACTGTAGTCCAGGAATATGGCAATTAGATTGTACACATTTAGAAGGAAAAATTATTCTGGTAGCAGTCCATGTAGCCAGTGGCTATATAGAAGCAGAAGTTATTCCAGCAGAAACAGGACAGGAAACAGCATACTTTATATTAAAATTAGCAGGAAGATGGCCAGTAAAAGTAATACACACAGACAATGGCAGCAATTTCACCAGTGCTGCAGTAAAAGCAGCATGTTGGTGGGCAGGTATCACACAAGAATTTGGAATTCCCTACAATCCCCAGAGCCAAGGAGTAGTGGAATCTATGAATAAGGAATTAAAGAAAATCATAGGGCAGGTCAGGGATCAAGCTGAACACCTTAAGACGGCAGTACAGATGGCAGTATTCATTCACAATTTTAAAAGAAAAGGGGGGATTGGGGAGTACAGTGCAGGAGAAAGAATAGTAGACATAATAGCATCAGATATACAAACTAAAGAACTACAAAAACAAATTACAAAAATTCAAAATTTTCGGGTTTATTACAGGGACAGCAGAGACCCAATTTGGAAAGGACCAGCAAAACTACTCTGGAAAGGTGAAGGGGCAGTAGTAATACAGGACAAAAGTGATATAAAGGTAGTGCCAAGAAGAAAAGCAAAAATAATTAGGCATTATGGAAAACAGATGGCAGGTGATGATTGTGTGGCAGGTAGACAGGATGAGGAT

>AY093604

TTTTTAGATGGCATAGATAAAGCCCAAGAAGAGCATGAAAGATATCACAGCAATTGGAGAGCAATGGCTAGTGATTTTAATCTGCCACCTGTAGTAGCAAAAGAAATAGTGGCCAGCTGTGATAAATGTCAGCTAAAAGGGGAAGCCATGCATGGACAAGTAGACTGTAGTCCAGGAATATGGCAATTGGATTGTACACATTTAGAAGGAAAAATTATCCTGGTAGCAGTCCATGTAGCCAGTGGCTATATAGAAGCAGAAGTTATCCCAGCAGAAACAGGACAGGAAACAGCATACTTTCTGTTAAAATTAGCAGGAAGATGGCCAGTAAAAGTAATACACACAGACAATGGCAGCAATTTCACTAGTGCTGCAGTAAAGGCAGCATGTTGGTGGGCAAATATCACCCAAGAATTTGGAATTCCCTACAATCCCCAAAGCCAAGGAGTAGTGGAATCTATGAATAAAGAATTAAAGAAAATCATAGGACAGGTCAGGGATCAAGCTGAACACCTTAAGACAGCAGTACAGATGGCAGTATTCATTCACAATTTTAAAAGAAAAGGGGGGATTGGGGGGTACAGTGCAGGGGAAAGAATAATAGACATAATAGCATCAGATATACAAACTAAAGAACTACAAAAACAAATCACAAAAATTCAAAATTTTCGGGTTTATTTCAGGGACAGCAGAGACCCAATTTGGAAAGGACCAGCAAAACTACTATGGAAAGGTGAAGGGGCAGTAGTAATACAGGACAATAATGAAATAAAGGTAGTACCAAGAAGAAAAGCAAAAATCATTAGGGACTATGGAAAACAGATGGCAGGTGATGATTGTGTGGCAGGTAGACAGGATGAGGAT

>AY093607
[truncated: 1,221,147 more chars]
