# Supplementary material for: Natural variation of HIV-1 group M integrase: Implications for a new class of antiretroviral inhibitors
Source: Retrovirology. 2008 Aug 7;5:74. doi: 10.1186/1742-4690-5-74 (PMC2546438; doi:10.1186/1742-4690-5-74)
Supplement: Additional File 2 — Variation by subtype [file 1742-4690-5-74-S2.pdf]

										10										20										30
	F	L	D	G	I	D	K	A	Q	E	E	H	E	K	Y	H	S	N	W	R	A	M	A	S	D	F	N	L	P	P
B 399			E^{1}	E^{1}		E^{5}<br>N^{3}<br>T^{1}	E^{2}<br>R^{2}<br>Q^{1}			D^{14}<br>A^{2}	D^{27}<br>G^{1}		D^{1}	R^{6}			N^{18}<br>T^{3}<br>C^{3}			K^{5}	S^{3}<br>T^{2}	I^{1}	V^{5}<br>T^{1}	G^{5}<br>N^{4}<br>A^{1}<br>D^{1}	E^{4}<br>N^{1}	Y^{1}	G^{2}	I^{9}		A^{2}
A 160			E^{16}			E^{1}				A^{3}	D^{24}		D^{1}	R^{64}			N^{3}<br>T^{1}			K^{16}	T^{37}	I^{1}	V^{6}	N^{4}<br>H^{3}				I^{3}		
AE 132				R^{2}			R^{2}				D^{14}		D^{2}	R^{96}			R^{2}			K^{2}	T^{86}		V^{10}		E^{2}					
AG 93											D^{17}			R^{89}			N^{2}			K^{4}	T^{9}		V^{3}	N^{5}	E^{4}			I^{5}		
C 432						E^{5}	R^{1}<br>Q^{1}			D^{5}<br>G^{1}	D^{16}		D^{2}	R^{17}			N^{18}			K^{1}	T^{1}			N^{13}<br>D^{1}<br>G^{1}	E^{81}			I^{1}		
D 82						S^{9}	Q^{9}<br>E^{2}			D^{9}	D^{10}		D^{11}	R^{11}			N^{70}			K^{4}					E^{4}			I^{6}		S^{2}
F 57						E^{4}				D^{5}	D^{23}			R^{4}			N^{81}<br>T^{4}						V^{5}	N^{5}				I^{11}		A^{4}
G 35											D^{34}			R^{74}			N^{11}			K^{6}				N^{9}						H^{9}

										40										50										60
	V	V	A	K	E	I	V	A	S	C	D	K	C	Q	L	K	G	E	A	M	H	G	Q	V	D	C	S	P	G	I
B<br>400	I^{23}	I^{4}		R^{2}			I^{3}		C^{8}<br>N^{2}<br>M^{1}		N^{1}				V^{4}<br>Q^{2}<br>I^{1}<br>S^{1}			Q^{1}		I^{13}<br>T^{3}<br>L^{1}				I^{1}		S^{2}	N^{1}		E^{1}<br>A^{1}	V^{1}
A<br>160	I^{77}	I^{13}<br>L^{1}		R^{2}	Q^{1}				N^{6}<br>C^{3}		N^{6}				Q^{1}<br>I^{1}			K^{3}		I^{19}										M^{24}
AE<br>132	I^{80}	I^{11}							N^{78}<br>C^{2}						V^{3}<br>Q^{2}			K^{2}	P^{2}	I^{4}					G^{2}					M^{4}<br>L^{2}
AG<br>93	I^{72}	I^{14}			Q^{2}				C^{12}<br>N^{2}						I^{2}					I^{13}<br>T^{5}<br>L^{2}							G^{20}	T^{3}		M^{3}
C<br>432	I^{76}	I^{1}		R^{6}			I^{1}		C^{3}<br>N^{2}<br>G^{1}		N^{1}	Q^{4}			Q^{7}<br>I^{2}<br>V^{2}					I^{58}<br>T^{8}<br>L^{1}				I^{1}						V^{3}<br>M^{1}
D<br>82	I^{2}	I^{5}					I^{2}		C^{5}						I^{6}<br>Q^{4}<br>V^{2}					L^{59}<br>I^{4}										
F 57	I^{12}	I^{11}					I^{5}								I^{7}<br>V^{5}					I^{9}				I^{4}						
G<br>35	I^{60}	I^{17}									N^{6}									I^{6}<br>L^{6}										

										70										80										90
	W	Q	L	D	C	T	H	L	E	G	K	I	I	L	V	A	V	H	V	A	S	G	Y	I	E	A	E	V	I	P
**B** 400								I^{1} V^{1}		E^{1}	Q^{1} R^{1} N^{1}	V^{51}	V^{2}	I^{2}			A^{1}			S^{1}	G^{1}			L^{1} V^{1}				I^{1}		S^{1}
**A** 161												V^{89}		I^{17} M^{7} V^{2}										M^{3} L^{1} V^{1}						
**AE** 132			V^{2}									V^{93}							L^{2}											
**AG** 93			I^{3}					V^{2}				V^{30}		I^{16} M^{11}										M^{2}						S^{2}
**C** 432												V^{41} T^{1}		I^{6}				R^{1}						M^{21} V^{2} L^{1}				I^{1}	T^{1}	S^{1} T^{1}
**D** 82											Q^{2}	V^{82}		I^{5} M^{2}								S^{2}		M^{4}						
**F** 57												V^{70}		I^{11}			A^{4}							L^{81}						
**G** 35														I^{11}																

										100										110										120
	A	E	T	G	Q	E	T	A	Y	F	L	L	K	L	A	G	R	W	P	V	K	T	I	H	T	D	N	G	S	N
B 400	T^{3}<br>S^{1}					D^{1}	A^{1}				I^{43}<br>V^{1}		R^{1}			A^{2}					T^{5}<br>R^{3}<br>N^{2}<br>Q^{1}	I^{6}<br>A^{4}<br>V^{3}<br>R^{1}	V^{10}						P^{11}<br>G^{7}<br>R^{7}<br>T^{5}	
A 161						D^{1}	A^{10}<br>S^{1}				I^{22}<br>V^{1}					A^{4}					R^{2}	V^{92}<br>I^{6}	V^{75}						P^{22}<br>T^{1}	
AE 132					S^{4}						I^{5}										R^{2}	V^{98}<br>A^{2}	V^{7}							
AG 93	E^{2}					D^{9}	A^{4}				I^{83}										R^{5}	V^{91}<br>I^{8}	V^{4}						P^{10}<br>T^{2}<br>R^{2}	
C 432	T^{2}<br>E^{1}					D^{2}<br>G^{1}	A^{1}			Y^{72}	I^{95}<br>V^{1}					A^{1}					R^{1}<br>Q^{1}	V^{92}<br>I^{6}	V^{1}<br>L^{1}						P^{7}<br>T^{5}<br>R^{1}	
D 82	T^{2}					D^{2}	A^{2}				I^{21}					A^{2}					R^{4}	V^{84}<br>I^{9}<br>A^{2}	V^{89}						G^{6}<br>R^{2}	
F 57	T^{7}					D^{5}					I^{86}										R^{16}	I^{11}<br>V^{7}							T^{46}<br>P^{40}	
G 35											I^{86}											V^{57}<br>I^{31}	L^{6}						P^{14}	

										130										140										150
	F	T	S	T	T	V	K	A	A	C	W	W	A	G	I	K	Q	E	F	G	I	P	Y	N	P	Q	S	Q	G	V
B<br>400		I^{17}<br>V^{1}	C^{1}	N^{23}<br>A^{21}<br>S^{2}<br>G^{1}<br>P^{1}	A^{25}<br>S^{3}<br>V^{3}	L^{1}<br>M^{1}		T^{1}					T^{1}	E^{1}<br>R^{1}	V^{7}	Q^{2}<br>N^{1}		D^{1}			V^{1}									A^{1}
A<br>161		I^{4}		A^{78}<br>S^{16}<br>N^{1}	A^{96}	F^{34}<br>M^{2}								N^{57}<br>D^{14}<br>S^{7}	V^{29}	Q^{86}			Y^{8}											
AE<br>132				A^{95}<br>N^{2}	A^{98}	F^{3}<br>M^{2}<br>L^{2}	R^{2}							N^{92}<br>D^{5}	V^{92}	R^{69}<br>Q^{25}														
AG<br>93		I^{6}		A^{96}	A^{96}	M^{6}	R^{6}							N^{81}<br>D^{12}<br>S^{2}	V^{78}	T^{82}<br>Q^{6}<br>N^{2}	H^{2}													
C<br>432		I^{2}		A^{70}<br>N^{13}<br>S^{5}<br>Q^{1}	A^{92}<br>V^{1}		R^{1}							S^{1}<br>D^{1}	V^{1}	Q^{89}<br>H^{2}					T^{1}<br>V^{1}		H^{1}							
D<br>82		I^{9}		A^{82}<br>S^{6}<br>N^{2}	A^{78}<br>V^{9}	L^{2}									V^{4}															
F 57		I^{16}<br>S^{11}		A^{70}<br>N^{5}<br>G^{4}	A^{70}<br>V^{4}				S^{4}							Q^{89}														
G<br>35		I^{14}		A^{71}<br>N^{9}<br>S^{6}	A^{86}									N^{71}<br>D^{17}<br>S^{6}	V^{9}	T^{97}														

										160										170										180
	V	E	S	M	N	K	E	L	K	K	I	I	G	Q	V	R	D	Q	A	E	H	L	K	T	A	V	Q	M	A	V
B 400	I^{10}	K^{1}		I^{6}<br>L^{2}		N^{9}<br>R^{1}	Q^{2}<br>K^{1}			T^{1}<br>Q^{1}<br>R^{1}		V^{1}	E^{4}<br>Q^{2}<br>A^{1}		I^{7}	K^{1}	E^{2}				Q^{1}<br>Y^{1}<br>L^{1}		R^{2}			L^{1}	L^{1}			
A 161				I^{1}						R^{1}					I^{5}		E^{81}						R^{4}							
AE 132		K^{2}		I^{3}			K^{2}			R^{2}					I^{14}		E^{93}													
AG 93			A^{2}				Q^{3}			Q^{2}			E^{2}		I^{5}		E^{12}													
C 432			A^{1}							R^{1}			E^{2}<br>V^{1}		I^{2}	G^{1}	E^{16}						R^{1}							
D 82						N^{5}	Q^{4}						E^{2}		I^{2}		E^{16}						R^{4}							
F 57				I^{9}									R^{16}<br>K^{14}<br>S^{7}<br>E^{4}		I^{25}		E^{9}				Y^{5}									
G 35																														

										190										200										210
	F	I	H	N	F	K	R	K	G	G	I	G	G	Y	S	A	G	E	R	I	V	D	I	I	A	T	D	I	Q	T
B 400	L^{3} Y^{1}	V^{1}			Y^{1}		K^{1}	R^{3}					E^{4} R^{2} D^{1}	T^{1}	T^{2} C^{2}			D^{1}			I^{38}		M^{3}	V^{1}	S^{1} T^{1}	S^{14} A^{1}	E^{1}	L^{5}		I^{1}
A 161	L^{2}								R^{1}				D^{3}		T^{2}						I^{98}		M^{6}			S^{6}	E^{1}	L^{2} M^{1}		
AE 132							K^{2}														I^{98}		M^{5}			S^{2}				
AG 93								R^{4}													I^{99}		M^{4}			S^{92}	E^{2}	L^{5}		
C 432	L^{2}							R^{13}					D^{2} E^{1}		C^{1}						I^{98}		M^{3}		S^{1}	S^{7}		M^{1} L^{1}		
D 82								R^{4}					E^{7}								I^{98}		M^{2}		S^{4}	S^{6}		L^{5}		
F 57																					I^{98}				S^{65}	S^{14}	E^{4}			
G 35																					I^{97}					S^{94}				

										220										230										240
	K	E	L	Q	K	Q	I	T	K	I	Q	N	F	R	V	Y	Y	R	D	S	R	D	P	L	W	K	G	P	A	K
B<br>400	R^{9}<br>Q^{2}<br>T^{1}	Q^{1}			N^{2}	H^{3}	V^{1}	I^{5}<br>S^{4}	N^{1}<br>R^{1}	L^{4}<br>V^{2}<br>M^{1}	R^{1}<br>S^{1}	K^{2}<br>H^{1}					F^{5}			N^{10}<br>G^{1}	K^{1}	E^{5}		I^{7}<br>V^{6}						
A<br>161	R^{11}	A^{1}			R^{2}	H^{8}<br>N^{2}		I^{27}<br>S^{3}	N^{5}	V^{3}	H^{1}	K^{13}					F^{4}					N^{1}		I^{70}<br>V^{6}						R^{2}
AE<br>132	R^{3}				N^{2}	H^{4}<br>N^{2}		S^{2}			T^{2}									N^{2}				I^{96}				S^{2}		
AG<br>93								I^{39}<br>S^{3}	N^{3}			K^{3}<br>H^{2}					F^{2}							I^{98}						
C<br>432	R^{12}<br>T^{1}<br>Q^{1}				N^{10}<br>R^{1}	R^{3}		I^{64}<br>L^{8}<br>M^{1}	Q^{7}<br>N^{2}	V^{2}	R^{1}	K^{2}					F^{1}			N^{1}				I^{98}<br>V^{1}						
D<br>82	R^{20}<br>T^{5}	K^{2}				H^{2}		I^{45}<br>S^{2}	N^{6}		R^{4}	K^{4}<br>H^{2}								N^{4}				I^{94}<br>V^{4}						
F 57	R^{56}				N^{4}			I^{72}<br>S^{5}			R^{7}						F^{5}			N^{5}				V^{91}<br>I^{7}						
G<br>35		A^{6}			N^{6}			I^{6}									F^{57}							I^{91}<br>V^{9}						

										250										260										270
	L	L	W	K	G	E	G	A	V	V	I	Q	D	N	S	D	I	K	V	V	P	R	R	K	A	K	I	I	R	D
B 400										I^{1}	L^{1}		E^{3}<br>H^{1}<br>Y^{1}	Q^{1}<br>K^{1}<br>S^{1}	T^{1}<br>N^{1}<br>G^{1}	E^{20}			I^{1}						V^{22}			L^{2}	K^{1}	
A 155												K^{1}		K^{2}	N^{21}<br>G^{3}<br>T^{1}	E^{5}				I^{3}					V^{11}			L^{5}	K^{3}	H^{1}<br>E^{1}
AE 132														K^{2}<br>S^{2}	N^{2}	E^{2}				I^{2}										
AG 79														K^{5}		E^{23}			I^{3}						V^{22}			L^{3}	K^{39}	
C 432											L^{6}		E^{1}	K^{2}	G^{3}<br>N^{1}	E^{2}	V^{1}			I^{2}					V^{55}				K^{57}	E^{1}
D 80											L^{8}		E^{8}	K^{4}	N^{5}	E^{57}									V^{42}				K^{3}	H^{3}
F 57															N^{5}	E^{96}									V^{4}					
G 35															N^{94}	E^{100}												L^{6}	K^{14}	H^{6}

										280																				
	Y	G	K	Q	M	A	G	D	D	C	V	A	S	R	Q	D	E	D												
B 396					V^{2}			A^{1}<br>N^{1}<br>G^{1}	G^{1}		M^{1}		G^{16}<br>N^{1}	G^{4}	R^{1}<br>P^{1}	N^{2}		N^{1}												
A 147								N^{3}	G^{2}		M^{2}		G^{73}<br>D^{1}	G^{3}		N^{7}		N^{5}												
AE 132								N^{2}	G^{2}				G^{96}	G^{3}		N^{4}		N^{2}												
AG 79					V^{3}			N^{4}	G^{3}		M^{3}		G^{84}			N^{5}<br>A^{3}		N^{15}												
C 430			R^{1}		V^{1}			A^{94}<br>T^{1}	G^{1}		M^{4}		G^{87}<br>D^{2}	G^{6}		N^{4}		N^{7}												
D 77									G^{5}		M^{4}		G^{18}		P^{3}			N^{3}												
F 57													G^{91}					N^{4}												
G 35								G^{6}					G^{94}																	
